# Development and Biological Characterization of Fluorescent Dynorphins for the Visualization of Kappa Opioid Receptors

**DOI:** 10.1021/acs.jmedchem.5c03072

**Published:** 2026-06-05

**Authors:** Predrag Kalaba, Monika Perisic Böhm, Filip D̵ikić, Nataša Tomašević, Ruth Drdla-Schutting, Simon Hasinger, Dylan M. Ines, Alexandra Wolf, Mireia Belil-Catalina, Sharon D. Bryant, Mariana Spetea, Christian W. Gruber, Markus Muttenthaler, Erik Keimpema

**Affiliations:** † Institute of Biological Chemistry, Faculty of Chemistry, 27258 University of Vienna, Vienna 1090, Austria; ‡ Vienna Doctoral School in Chemistry, University of Vienna, Vienna 1090, Austria; § Department of Molecular Neurosciences, Center for Brain Research, 27271 Medical University of Vienna, Vienna 1090, Austria; ∥ Institute of Pharmacology, Center for Pharmacology and Physiology, Medical University of Vienna, Vienna 1090, Austria; ⊥ Department of Neurophysiology, Center for Brain Research, Medical University of Vienna, Vienna 1090, Austria; # Inte:Ligand GmbH, Vienna 1070, Austria; ¶ Department of Pharmaceutical Chemistry, Institute of Pharmacy and Center for Molecular Biosciences Innsbruck (CMBI), 27255 University of Innsbruck, Innsbruck 6020, Austria; ∇ Institute for Molecular Bioscience, The University of Queensland, Brisbane 4072, Queensland, Australia

## Abstract

The kappa opioid receptor (KOR) controls a wide variety of biological processes, including pain and reward responses. However, its precise spatiotemporal location remains poorly understood due to limited selective molecular tools. We addressed this problem by developing dynorphin-based fluorescent KOR tracers that are selective for KOR over its closely related mu- and delta-family members (MOR, DOR). Our lead tracer is highly selective for KOR (>360-fold over MOR and DOR) and can effectively visualize KORs in the somatodendritic compartment of cultured primary cortical neurons, as well as in superficial layer neurons of the spinal cord dorsal horn. Furthermore, it blocked the KOR agonist U50,488-induced reduction in the excitatory drive of spinal cord neurons. The developed tracer can also be immobilized with paraformaldehyde and used for *post hoc* imaging, rendering it a highly valuable tool for mapping KOR expression in physiological and pathophysiological contexts.

## Graphical abstract

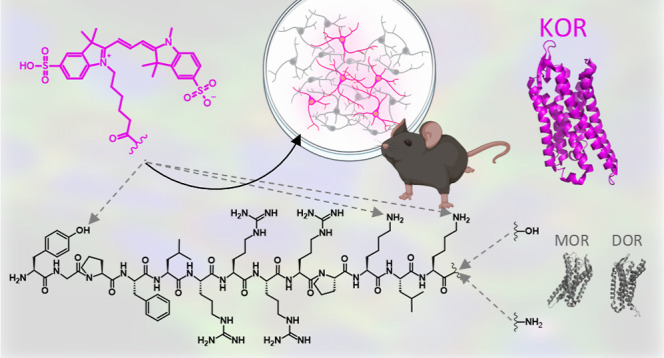

All authors commented on the manuscript and declared no conflict of interest.

## Introduction

The opioid system regulates a wide variety of physiological processes, including pain, reward, mood, and stress.^1–3^ This is accomplished through the action of the peptide hormones endorphin,^4^ enkephalin,^5^ and dynorphin (Dyn A)^6,7^ acting on their respective G protein-coupled receptors (GPCRs), the mu opioid receptor (MOR),^8^ the delta opioid receptor (DOR),^9^ and the kappa opioid receptor (KOR).^2^ In the central nervous system (CNS), activation of these opioid receptors engages Gα_i/o_ subunits, leading to inhibition of adenylyl cyclase. The resulting reduction in intracellular cyclic adenosine monophosphate (cAMP) concentration leads to the closure of voltage-gated calcium channels and, ultimately, to reduced neurotransmitter release.^10^ As such, opioid receptor signaling directly controls activity in the peripheral and central nervous systems. For instance, the well-known analgesic effects of opioids are mediated by receptor engagement in pain networks, reducing neuronal activity and diminishing pain signal processing.^11^ Although the main analgesic drug target for alleviating pain is MOR, which is, for instance, activated by morphine and fentanyl,^11^ more recent therapeutic strategies have focused on KOR to reduce addiction.^2^ While KOR agonists are efficacious in treating acute, inflammatory, and neuropathic pain,^2^ the precise anatomical and cellular receptor locations mediating these signaling events remain elusive due to a lack of selective and knockout validated antibodies, particularly for immunohistochemistry.^12^ Since antibody development for GPCRs is challenging,^13^ novel peptide-based ligands that harbor reporter tags and bind GPCRs with high affinity and selectivity represent promising alternatives for localization and signaling studies.^14,15^

Here, we addressed the lack of selective KOR antibodies by developing a series of fluorescently labeled dynorphin analogs incorporating strategic sequence modifications to enhance KOR selectivity.^16^ The study explored 1) Dyn A­(1-8), Dyn A­(1-11), and Dyn A­(1-13) peptides as scaffolds for fluorophore attachment, 2) optimal fluorophore attachment sites, and 3) the impact of C-terminal modifications on KOR selectivity. Tracer binding affinities were characterized across KOR, DOR, and MOR; tracer functional properties were verified using [^35^S]-GTPγS, cAMP, and β-arrestin recruitment assays; and molecular modeling was performed. The applicability of our tracers for KOR imaging was investigated in cell models expressing green fluorescent protein (GFP)-tagged opioid receptors, followed by paraformaldehyde (PFA)-based cross-linking to fix the tracers for *post hoc* confocal laser-scanning microscopy imaging. This was expanded to KOR-expressing mouse primary cortical neurons to verify our tracers’ imaging capacity in endogenously expressing systems. The tracers were further evaluated in *ex vivo* rat spinal cord slices *via* patch-clamp electrophysiology. Taken together, this work reports new structure–activity relationship data for dynorphin-based ligands, resulting in bright, selective fluorescent KOR tracers suitable for *post hoc* imaging studies.

## Results

### Tracer Design and Synthesis

Opioid peptides contain a “message sequence”(residues 1-4), essential for binding across all opioid receptors, as well as an “address sequence” (residues 5-11), responsible for mediating receptor subtype preference.^17^ Modifications to these sequences can alter the affinity for specific receptor subtypes. The endogenous opioid peptide dynorphin A activates KOR with high affinity (*K*_i_ = 0.1 nM) and consists of 17 residues (Dyn A­(1-17)-OH: H_2_N-YGGFLRRIRPKLKWDNQ-CO­OH).^6,7,18^ C-terminal truncations are well-tolerated with P^3^ and R^8^ modifications enhancing KOR selectivity, and the P^3^ modification resulting in an agonist-to-antagonist switch.^19^ [P^3^,R^8^]­Dyn A­(1-11)-NH_2_ binds with picomolar affinity and >5000-fold selectivity to KOR.^16^ Considering this potent and selective KOR pharmacology, we used [P^3^,R^8^]­Dyn A­(1-11)-NH_2_ as the starting point for our tracer design and carried out a structure−affinity-relationship (SAR) study of fluorescent tracers with different dynorphin peptide lengths (1-8, 1-11 or 1-13 residues), different fluorophore attachment positions (Y^1^, K^11^, K^13^), different C-terminal modifications (acid *vs* amide), and ether-based spacers between the peptide and fluorophore (Figure 1). Tracers were synthesized via manual Fmoc-SPPS (fluorenylmethyloxycarbonyl-solid-phase peptide synthesis), and the fluorophore (Cy3_s_-alkyne) was attached in solution via copper-catalyzed azide–alkyne cycloaddition (CuAAC). Rink amide resin was used to produce C-terminal amides, and preloaded Wang resins were used to produce carboxylic acids. HATU (hexafluorophosphate azabenzotriazole tetramethyl uronium) was used as a coupling reagent. Tracers (**1–4**) were designed with an azide modification at the phenol -OH of the Y^1^ side chain (tyrosine­(CH_2_CH_2_N_3_)) for fluorophore attachment and lacked an additional spacer. A short AEEA (2-[2-(2-azidoethoxy)­ethoxy]­acetic acid) spacer was introduced to the side chain amine at K^11^ of tracers (**6–8**) and (**10**), and at K^13^ of tracers (**5**) and (**9**) on-resin. Fully assembled peptide constructs, including spacers, were cleaved off the resin with trifluoroacetic acid (TFA) and purified by C_18_ reversed-phase high-performance liquid chromatography (C_18_-RP-HPLC), followed by in-solution fluorophore attachment and a second purification (Figure 1a). Ligands were characterized by analytical C_18_-RP-HPLC and high-resolution electrospray ionization mass spectrometry (HR-ESI-MS), and all final compounds had a purity of >95% (Table S1, Figure S1).

**Figure 1:**
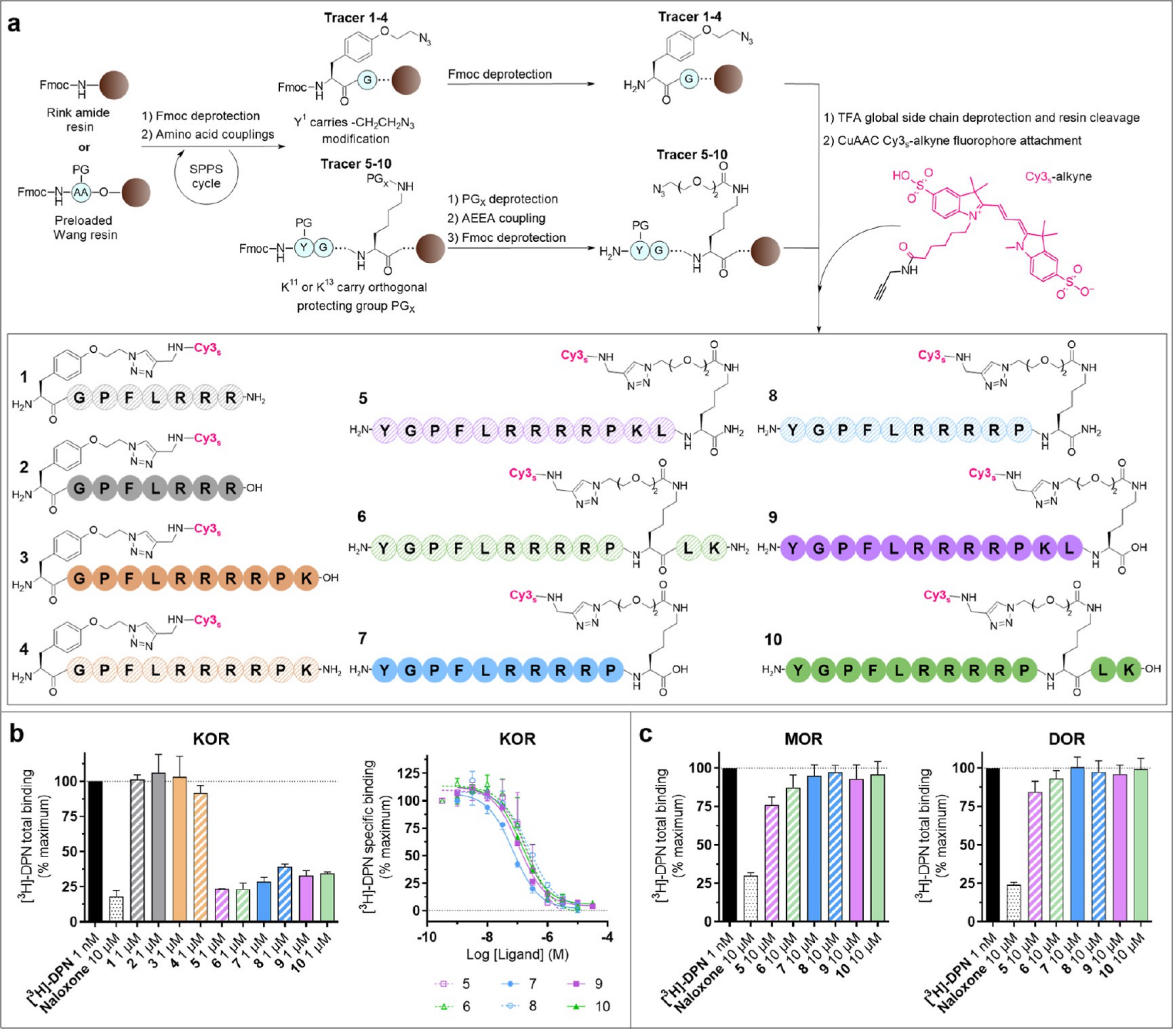
Peptide tracer synthesis, sequences, and binding affinities. (a) Synthetic approach for KOR tracer production and overview of peptide sequences and fluorophore attachment points. Peptide assembly was carried out via Fmoc-SPPS, followed by TFA cleavage and in-solution attachment of Cy3_s_-alkyne to peptide-azide precursors. 8-Residue peptides are colored gray, 11-residue peptides with a fluorophore at position 1 brown, 11-residue peptides with a fluorophore at position 11 blue, 13-residue peptides with a fluorophore at position 13 purple, and 13-residue peptides with a fluorophore at position 11 green. Corresponding C-terminal amides are striped. (b) Radioligand displacement assays at KOR using membrane preparations of HEK293 cells stably expressing mKOR-GFP and tritiated diprenorphine ([^3^H]-DPN, 1 nM). One-point bindings were carried out with 10 μM of fluorescent tracers and revealed that tracers (**1–4**) did not displace the radioligand, whereas tracers (**5–10**) showed full radioligand displacement similar to the test ligand naloxone. KOR binding affinities of tracers (**5–10**) were evaluated in full dose–response curves, and the obtained *K*_i_ values were in the range of 25–100 nM (Table 1). Specific binding in full dose–response curves was obtained by subtracting nonspecific binding at 10 μM naloxone from total binding. (c) One-point bindings at MOR and DOR applying 10 μM of each tracer and [^3^H]-DPN (1 nM). Membranes were obtained from HEK293 cells stably expressing mMOR-GFP and mDOR-GFP, respectively. None of the tested tracers showed radioligand displacement to the level of the positive control naloxone at either MOR or DOR. Data are presented as mean ± SD from three independent experiments (*N* = 3) in technical duplicates (*n* = 2).

### Pharmacological Characterization

Binding affinities were determined through radioligand displacement assays on HEK293 cells stably overexpressing the murine opioid receptor-GFP fusion constructs mKOR-GFP, mMOR-GFP, and mDOR-GFP (Figure 1b,c, Table 1). With a sequence homology of 94%, mouse and human KOR are considered highly similar, except for some nonconserved GPCR kinase/arrestin residues in human KOR.^20^ Since experimental work was primarily directed at rodent tissues, we characterized the ligands against the mKOR, mMOR, and mDOR variants, unless stated otherwise. Tracers (**1**–**4**), with Cy3_s_ attached to the Y^1^ side chain, did not bind to KOR and were thus not further characterized at MOR and DOR (Figure 1b, Table 1). Upon changing the labeling position from Y^1^ to K^11^ or K^13^ and introducing an AEEA spacer to increase the distance between peptide and fluorophore (tracers **5**–**10**), nanomolar KOR affinity was obtained (Figure 1b). Importantly, tracers (**5**–**10**) did not bind to MOR or DOR, resulting in high selectivity (>100-fold), with tracer (**7**) showing the highest selectivity for KOR with >368-fold (Figure 1c, Table 1). Slight differences in KOR affinities were observed depending on the presence of the C-terminal carboxylic acid vs. amide, with the acid consistently having the better affinity (2–3-fold).

**Table 1:** Generated Compounds and Their Affinities for Mouse KOR, MOR, and DOR^d^

**tracer ID**	***K*_i_ (mKOR, nM)**	$\text{Selectivity ratio}\;\frac{K_{i}\left(mMOR,mDOR\right)}{K_{i}\left(mKOR\right)}$	
1 [Y^1^(C_2_H_4_-Cy3_s_),P,^3^R^8^]Dyn A(1-8)-NH_2_ ^a^	>10,000	–	
2 [Y^1^(C_2_H_4_-Cy3_s_),P,^3^R^8^]Dyn A(1-8)-OH^a^	>10,000	–	
3 [Y^1^(C_2_H_4_-Cy3_s_),P,^3^R^8^]Dyn A(1-11)-OH^a^	>10,000	–	
4 [Y^1^(C_2_H_4_-Cy3_s_),P,^3^R^8^]Dyn A(1-11)-NH_2_ ^a^	>10,000	–	
5 [P^3^,R^8^,K^13^(AEEA-Cy3_s_)]Dyn A(1-13)-NH_2_ ^b^	95.4 ± 38.2	105	
6 [P^3^,R^8^,K^11^(AEEA-Cy3_s_)]Dyn A(1-13)-NH_2_ ^b^	86.3 ± 43.1	116	
**7 [P^3^,R^8^,K^11^(AEEA-Cy3_s_)]Dyn A(1-11)-OH** ^b^	**27.2 ± 5.8**	**368**	
8 [P^3^,R^8^,K^11^(AEEA-Cy3_s_)]Dyn A(1-11)-NH_2_ ^b^	95.5 ± 8.2	105	
9 [P^3^,R^8^,K^13^(AEEA-Cy3_s_)]Dyn A(1-13)-OH^b^	42.7 ± 4.8	234	
10 [P^3^,R^8^,K^11^(AEEA-Cy3_s_)]Dyn A(1-13)-OH^b^	54.2 ± 9.1	185	
			
**Control peptides**	***K*_i_ or EC_50_ (nM)**		
[P^3^,R^8^]Dyn A(1-11)-OH	*K*_i_ = 27.6 ± 11.7 nM^c^		
Dyn A(1-13)-OH	*K*_i_ = 0.3 ± 0.03 nM^21^		
DAMGO	EC_50_ = 6.5 ± 4.7 nM^22^		
DADLE	EC_50_ = 10.4 ± 3.7 nM^22^		

a *K*_i_ values at mMOR and mDOR not determined;

b *K*_i_ values at mMOR and mDOR >10,000 nM;

c *K*_i_ at hKOR. DADLE, [d-Ala^2^,d-Leu^5^]-enkephalin; DAMGO, [d-Ala^2^,*N*-MePhe^4^,Gly-ol]-enkephalin. Values represent means ± SD of at least three independent experiments (*N* = 3) performed in duplicates (*n* = 2, radioligand displacement assay) or triplicates (*n* = 3, cAMP assay).

d Values were calculated from radioligand displacement assays applying membrane preparations of HEK293 cells stably expressing murine KOR, MOR, or DOR and [^3^H]-diprenorphine ([^3^H]-DPN), or CHO cells stably expressing human KOR and [^3^H]-U69,593 (only for the *K*_i_ value of the parent peptide of tracer (**7**), [P^3^,R^8^]­Dyn A­(1-11)-OH). *K*_i_ values > 10 μM were assigned to compounds that displaced <25% of the radioligand at 10 μM. Selectivity ratios express the fold binding selectivity for KOR and were obtained by dividing respective MOR and DOR affinities by the KOR affinity. EC_50_ values were determined from cAMP assays using HEK293 cell lines stably expressing murine opioid receptors. *K*_i_ or EC_50_ values of standard agonists at applied stable cell lines (Dyn A­(1-13)-OH for KOR, DAMGO for MOR, and DADLE for DOR) were validated previously, and the obtained values are indicated at the bottom.

Next, we evaluated the functional performance of lead tracer (**7**) in a FRET-based (fluorescence resonance energy transfer) cAMP accumulation assay. While the fluorescent tracer (**7**), similar to Cy3_s_ alone, could not be analyzed using the cAMP assay (Figure S2a), its nonfluorescent parent peptide [P^3^,R^8^]­Dyn A­(1-11)-OH revealed minor receptor activation at micromolar concentration (*E*_max_ = 18%, Table 2) compared to control agonist U50,488, supporting weak partial agonism (Figure S2b). To confirm partial agonism of our lead tracer (**7**), we additionally performed a [^35^S]-GTPγS binding assay. Both [P^3^,R^8^]­Dyn A­(1-11)-OH and tracer (**7**) induced [^35^S]-GTPγS binding and exhibited weak partial agonism at concentrations around 1 μM (Table 2 and Figure S2c), similar to the value obtained for the parent peptide in the cAMP assay and to previous studies.^16^ We next evaluated β-arrestin-2 recruitment as an indicator of KOR-mediated internalization in a BRET-based (bioluminescence resonance energy transfer) assay, where [P^3^,R^8^]­Dyn A­(1-11)-OH did not recruit β-arrestin-2, while KOR agonist U50,488 recruited β-arrestin-2 as expected (Figure S2d).^23^

**Table 2:** Overview of Compound Potencies and Efficacies at KOR^b^

	EC_50_ (nM)	*E*_max_ (%)
U69,593	20.6 ± 3.5	100 ± 1
[P^3^,R^8^,K^11^(AEEA-Cy3_s_)]Dyn A(1-11)-OH (**7**)	1189 ± 245	17 ± 3
[P^3^,R^8^]Dyn A(1-11)-OH^a^	1190 ± 35 (1176 ± 760)	18 ± 2 (18 ± 4)

a Values in parentheses represent EC_50_ and *E*_max_ as measured in cAMP assays at murine KOR. Values represent means ± SD of at least three independent experiments (*N* ≥ 3) performed in duplicate (*n* = 2, [^35^S]-GTPγS binding assay) or triplicate (*n* = 3, cAMP assay).

b A radioactivity-based [^35^S]-GTPγS binding assay was used for the evaluation of compound activity at human KOR. U69,593, a standard full KOR agonist, was applied as a positive control.

### Molecular Modeling and Chemical Feature Interaction Analysis

Starting from the respective cryo-EM structures of KOR in complex with the agonist Dyn A­(1-8) (PDB 8F7W)^24^ and the antagonist norbinaltorphimine (NorBNI; PDB 8VVE),^25^ we derived the 3D-pharmacophores of Dyn A­(1-8) and NorBNI with KOR using LigandScout XT (Inte:Ligand).^26^ 3D-pharmacophores are abstractions of chemical feature interaction patterns related to a biological outcome, used in drug discovery to interpret SAR data, perform virtual screening, and optimize leads.^26^ It should be noted that in the original article reporting the cryo-EM resolved structure of dynorphin and KOR, the electron microscopy maps enabled the authors only to model in coordinates of the first eight residues of Dyn A­(1-13) in the KOR binding site, and only those corresponding coordinates for the first eight residues of dynorphin were deposited to the protein databank along with the other macromolecular data (PDB 8F7W).^24^ Therefore, the opioid ligand available for analysis in our study is herein referred to as Dyn A­(1-8) rather than Dyn A­(1-13). The pharmacophores of Dyn A­(1-8) agonist and NorBNI antagonist at KOR are shown in Figures 2 and 3. Dyn A­(1-8) and NorBNI displayed similar key interactions with the message domain of KOR’s orthosteric binding site, including positive ionizable interactions with respective amino groups and D138 (3.32) (Ballesteros-Weinstein numbering), hydrogen bond acceptors between Y139 (3.33) and the carbonyl oxygen of [Y^1^] of Dyn A­(1-8) and the oxygen of the furan moiety of NorBNI, as well as hydrophobic interactions with Y139 (3.33), M142 (3.36), and I294 (6.55). H304 (7.40) was the only common interaction partner in the receptor address region in both cryo-EM complexes. Other interactions included those linked to NorBNI-KOR selectivity, such as hydrogen bonds with S211 (ECL2) and V108 (2.53) in the message region of the KOR binding pocket.^25^ Other unique interactions of the NorBNI pharmacophore compared to the Dyn A­(1-8) pharmacophore included C210 (ECL2), I316 (7.52), and I290 (6.51), as well as Y312 (7.48) and Y320 (7.56), likely involved in stabilizing an inactive conformation of KOR (Figure 3).^25^ Unique KOR interactions with the agonist Dyn A­(1-8) included hydrophobic interactions with T111 (2.56), V134 (3.28), I135 (3.29), and V230 (5.42) in the message region. Positive ionizable hydrophobic interactions observed between the address region [R^6^,R^7^] of the peptide and E209 (ECL2) and E297 (ECL3) of KOR have been linked to KOR selectivity (Figure 2).^24^

**Figure 2:**
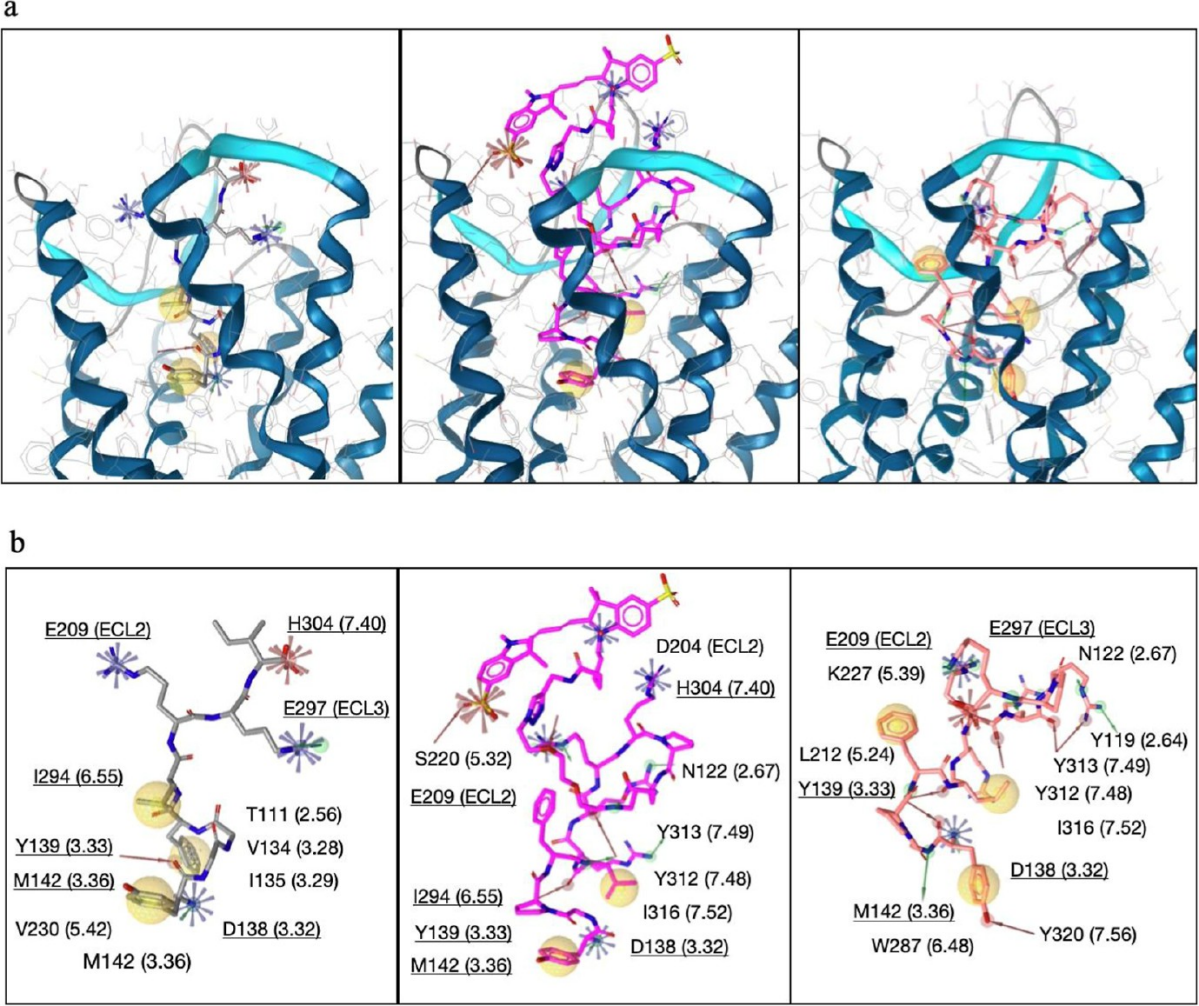
Comparison of Dyn A­(1-8), tracer [P^3^,R^8^,K^11^(AEEA-Cy3_s_)]­Dyn A­(1-11)-OH (7), and parent peptide [P^3^,R^8^]­Dyn A­(1-11)-OH in the KOR-Dyn A­(1-8) agonist binding site. (a) Side-by-side depictions of the 3D-conformations and interaction patterns of the agonist Dyn A­(1-8) (gray) derived from the cryo-EM resolved KOR-Dyn A­(1-8) complex (PDB ID: 8F7W, chains R and P), and tracer (**7**) (magenta) and [P^3^,R^8^]­Dyn A­(1-11)-OH (peach) pharmacophore based docking and molecular docking, prioritized poses with the agonist KOR binding site derived from the cryo-EM resolved structure (PDB ID: 8F7W, chain R). (b) KOR amino acids forming chemical feature interactions with Dyn A­(1-8) (gray), tracer (**7**) (magenta), or [P^3^,R^8^]­Dyn A­(1-11)-OH (peach). The chemical interaction features (structure-based 3D pharmacophores in the depictions) were derived using LigandScout XT.^26^ The interaction feature types depicted are hydrophobic (yellow spheres), hydrogen-bond donors and acceptors (green and red arrows, respectively), and positive and negative ionizable features (blue and red stars, respectively). Single-letter codes and numbering correspond to the cryo-EM resolved structure in the Protein Data Bank. Ballesteros-Weinstein numbers are shown in parentheses. Amino acid interaction partners that are in common with those observed in Dyn A­(1-8)-KOR cryo-EM resolved structure (PDB ID: 8F7W) are underlined. Hydrogen atoms are not displayed.

**Figure 3:**
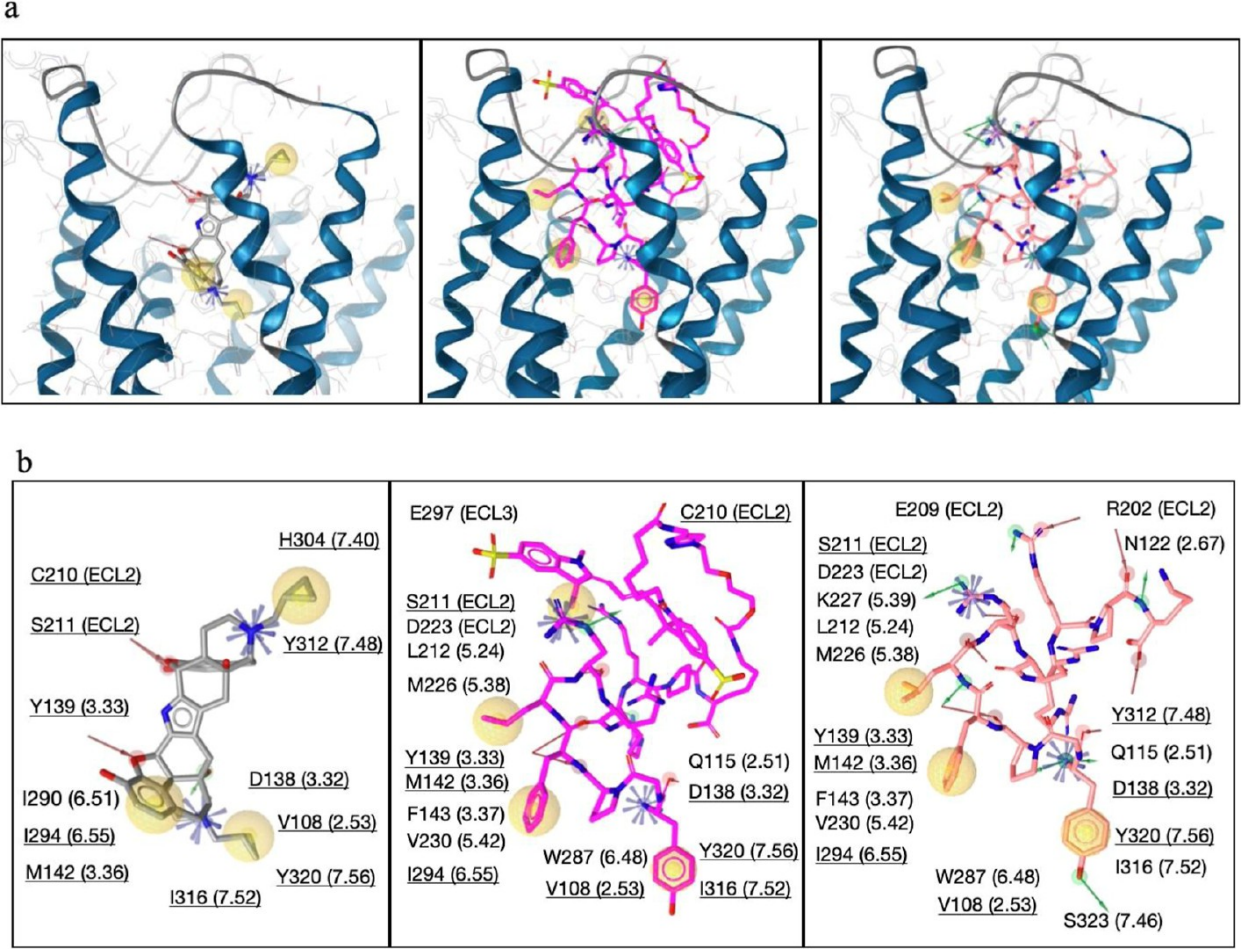
Comparison of NorBNI, tracer [P^3^,R^8^,K^11^(AEEA-Cy3_s_)]­Dyn A­(1-11)-OH (7), and parent peptide [P^3^,R^8^]­Dyn A­(1-11)-OH in the KOR-NorBNI antagonist binding site. (a) Side-by-side depictions of the 3D-conformations and interaction patterns of the antagonist NorBNI (gray) derived from the cryo-EM resolved KOR-NorBNI complex (PDB ID: 8VVE, chains A and F), and tracer (**7**) (magenta) and [P^3^,R^8^]­Dyn A­(1-11)-OH (peach) derived from pharmacophore based docking and molecular docking into the antagonist KOR binding site derived from the cryo-EM resolved structure (PDB ID: 8VVE, chain A). (b) Overview of receptor amino acids forming chemical feature interactions (structure-based 3D-pharmacophores in the depictions) with NorBNI (gray), tracer (**7**) (magenta), and [P^3^,R^8^]­Dyn A­(1-11)-OH (peach) were derived using LigandScout XT.^26^ The interaction feature types depicted are hydrophobic (yellow spheres), hydrogen-bond donors and acceptors (green and red arrows, respectively), and positive and negative ionizable features (blue and red stars, respectively). Single-letter codes and numbering correspond to the cryo-EM resolved structure in the Protein Data Bank. Ballesteros-Weinstein numbers are shown in parentheses. Amino acid interaction partners that are in common with those from the KOR-NorBNI cryo-EM resolved structure are underlined. Hydrogen atoms are not displayed.

With this biophysical data in hand, we performed docking and 3D-pharmacophore alignment experiments with fluorescent dynorphin tracers (**1–10**) and the parent peptide of tracer (**7**), [P^3^,R^8^]­Dyn A­(1-11)-OH, in both agonist- and antagonist-resolved binding sites and compared it to observed interactions of Dyn A­(1-8) and NorBNI (overlaid structures shown in Figures S3 and S4). For the parent peptide [P^3^,R^8^]­Dyn A­(1-11)-OH, the observed interactions were similar to those at the KOR-NorBNI site and the NorBNI antagonist pharmacophore. Those included S211 (ECL2) and V108 (2.53) linked to KOR selectivity, Y312 (7.48) and Y320 (7.56) linked to KOR antagonism, and hydrophobic interactions with I294 (6.55) and I316 (7.52) (Figure 3). Interactions with E209 (ECL2) and E297 (ECL3), which were also observed for Dyn A­(1-8) and are involved in KOR selectivity,^24^ were also observed in the docked poses of [P^3^,R^8^]­Dyn A­(1-11)-OH (Figure 2). Other common interaction partners of the KOR-Dyn A­(1-8) pharmacophore with the KOR message domain were D138 (3.32), Y139 (3.33), and M142 (3.36) (Figure 2). Compared to the parent peptide, tracers (**1–4**) did not fit well into KOR’s orthosteric site due to the fluorophore attachment at the hydroxyl group of [Y^1^] of the N-terminal message domain, aligning with the lack of KOR affinity. The N-terminal amine of the peptide ligand should be positioned within the message region of the KOR binding site as delineated by the agonist and antagonist 3D-pharmacophores. Despite several alignment experiments with >200 low-energy conformations of tracers (**1–4**), none of the chemical features could be aligned to the 3D-pharmacophores of KOR-Dyn A­(1-8) or KOR-NorBNI. Docking studies of tracers (**1–4**) also did not yield any suitable poses, likely due to the incompatibility of charged, bulky fluorophores with the receptor message region, which is situated deep within the binding pocket. Therefore, the attachment of fluorophores to the peptide message domain is not amenable to KOR tracers due to their size and charge, resulting in a lack of the essential chemical features required for KOR interactions. Conversely, tracers (**5–10**) with the fluorophore attached to the address region all yielded poses similar to those of the parent peptide and were compatible with KOR binding. Interactions observed between the lead tracer (**7**) and V108 (2.53), S211 (ECL2), and E209 (ECL2) are consistent with pharmacological results exhibiting selectivity for KOR. Interactions with other key amino acids in the KOR message and address domains, including D138 (3.32), Y139 (3.33), M142 (3.36), I316 (7.52), Y320 (7.56), W287(6.48), I294 (6.55), C210 (ECL2), and E297 (ECL3) were also obtained (Figure 3).

### KOR Selectivity of Fluorescent Tracers

To confirm the selectivity and applicability of our tracers as *post hoc* imaging tools in biological systems (after PFA cross-linking), we cultured HEK293 cells stably expressing the mKOR-GFP, mDOR-GFP, or mMOR-GFP.^22^ Application of tracers (**5**) or (**6**) at 100 nM resulted in robust accumulation and internalization of overlapping GFP and Cy3_s_ signals when exposed to KOR-GFP cells for 60 min (Figure S5a,a_1_), but not with 10 nM, data not shown. Signal accumulation between (**5**) and (**6**) was not visibly different, indicating that Cy3_s_ on K^11^ or K^13^ did not differentially affect tracer binding. Sites of high intensities of red fluorescence, signifying a high local concentration of tracer and receptor, are representatively marked across figures using arrows. Stimulation of parent HEK293 cells with 100 nM tracer did not display any cellular labeling, supporting a KOR-dependent binding mechanism (Figure S5b,b_1_).

Next, we treated KOR-GFP cells with 100 nM of tracers (**5**), (**6**), (**7**), or (**8**) to see if Dyn A­(1-13) would change specificity as compared to truncated Dyn A­(1-11). Of the four tracers tested, tracer (**7**) had the highest signal-to-noise ratio, and tracer (**8**) had the weakest fluorescent signal (Figures 4a,b,c,d and S6). Internalization was again observed across all tested tracers. Pretreatment with KOR antagonist LY-2456302 ^27^ completely abolished labeling, confirming KOR specificity (Figure 4a_1_,b_1_,c_1_,d_1_). Given the lack of β-arrestin-2 recruitment of the parent peptide (Figure S2d), we further examined the internalization of tracer (**7**) in KOR-GFP cells in the presence of barbadin, a β-arrestin/β2-adaptin interaction inhibitor, and pitstop-2, a clathrin inhibitor to block endocytosis. While tracer (**7**) internalization was somewhat reduced when pretreated with barbadin (50 μM for 30 min; Figure S7a,a_1_),^28^ pitstop 2 (10 μM for 15 min)^29^ prevented intracellular accumulation, leading to strong membrane labeling instead (Figure S7a,a_2_). Hence, the above findings indicate that tracer (**7**) is a weak partial agonist at micromolar concentrations and internalizes predominantly via clathrin-mediated endocytosis in heterologous KOR overexpression systems, consistent with the literature.^16,30^

**Figure 4:**
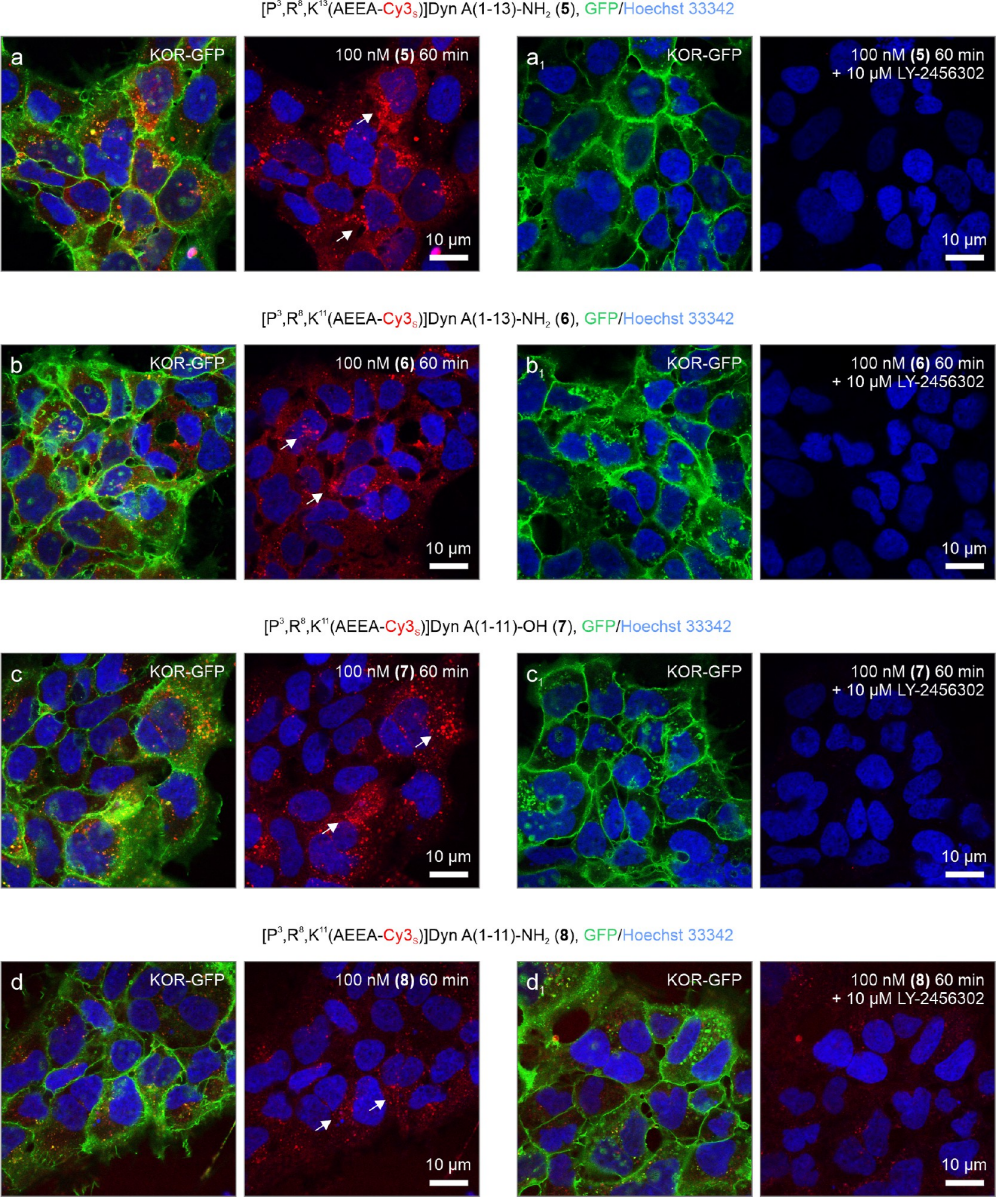
Tracers [P^3^,R^8^,K^13^(AEEA-Cy3_s_)]­Dyn A­(1-13)-NH_2_ (**5**), [P^3^,R,^8^K^11^(AEEA-Cy3_s_)]­Dyn A­(1-13)-NH_2_ (**6**), [P^3^,R,^8^K^11^(AEEA-Cy3_s_)]­Dyn A­(1-11)-OH (**7**), and [P^3^,R^8^,K^11^(AEEA-Cy3_s_)]­Dyn A­(1-11)-NH_2_ (**8**) labeling comparison in mKOR-GFP-expressing HEK293 cells. (a–d) Incubation of cells with 100 nM (**5**), (**6**), (**7**), and (**8**), respectively, confirmed robust receptor labeling (red, excitation 560 nm) after PFA fixation (arrows mark sites of high tracer accumulation). (a_1_–d_1_) This was prevented by a 30 min pretreatment of cells with 10 μM KOR antagonist LY-2456302, confirming receptor-dependent activity. Hoechst 33342 was used to label cellular nuclei (blue, excitation 405 nm).

Since tracer (**7**) had the best signal-to-noise ratio, binding affinity, and selectivity (Table 1), it was selected as the lead tracer for further characterization.

Subsequently, we dose–response tested tracer (**7**) for KOR selectivity in our cell models. At 10 nM, only minor amounts of fluorescent signals were detected up to 1 h of stimulation. However, at 100 nM, the Cy3_s_ signal was robustly detected as early as 5 min post-stimulation, with 30 min reaching intensities similar to 60 min (Figures 4c and 5a,b). No labeling was detected when stimulating either MOR-GFP or DOR-GFP-containing cells with 100 nM of (**7**), reconfirming KOR selectivity (Figure 5a_1_−b_2_). While 1 μM of (**7**) revealed even more robust fluorescent signals than 100 nM (Figure S8), minor amounts of fluorescence were detected in MOR-GFP and DOR-GFP cells, even though our pharmacology did not detect binding at MOR and KOR at this concentration on membrane fractions (Table 1). We verified this discrepancy by exposing HEK293 cells, without overexpressing receptors, to 1 μM of (**7**) and observed that at these concentrations our tracer became detectable on cell somata, but mostly at cellular debris sites. Pretreatment with 10 μM of KOR antagonist LY-2456302 (also inhibiting MOR and DOR at this concentration^27^) did not prevent tracer accumulation, suggesting that nonspecific background labeling occurred at concentrations ≥1 μM (Figure S9). To verify whether the observed cellular debris was due to the cytotoxicity of our compound, we performed an MTT assay. However, we observed no increased cytotoxicity at tracer (**7**) concentrations up to 10 μM compared to the positive control (1% Triton X-100; Figure S10a). Since we could not exclude interaction between the fluorophore moiety and the signal readout, we performed *post hoc* labeling for cleaved caspase-3, an apoptosis marker.^31^ Neither did we observe any nuclear fragmentation nor increased cleaved caspase-3 immunoreactivity at tracer concentrations up to 10 μM, confirming that tracer (**7**) did not induce cell death (Figure S10b-c_2_). Since tracer (**7**) contained a C-terminal carboxyl group, we also investigated whether C-terminal amide tracers (**5**) and (**6**) could be improved by switching to a C-terminal carboxylic acid. Surprisingly, both C-terminal acid tracers (**9**) and (**10**) labeled less robustly, with tracer (**10**) labeling considerably weaker as compared to its amide counterpart (Figures S11 and S12).

**Figure 5:**
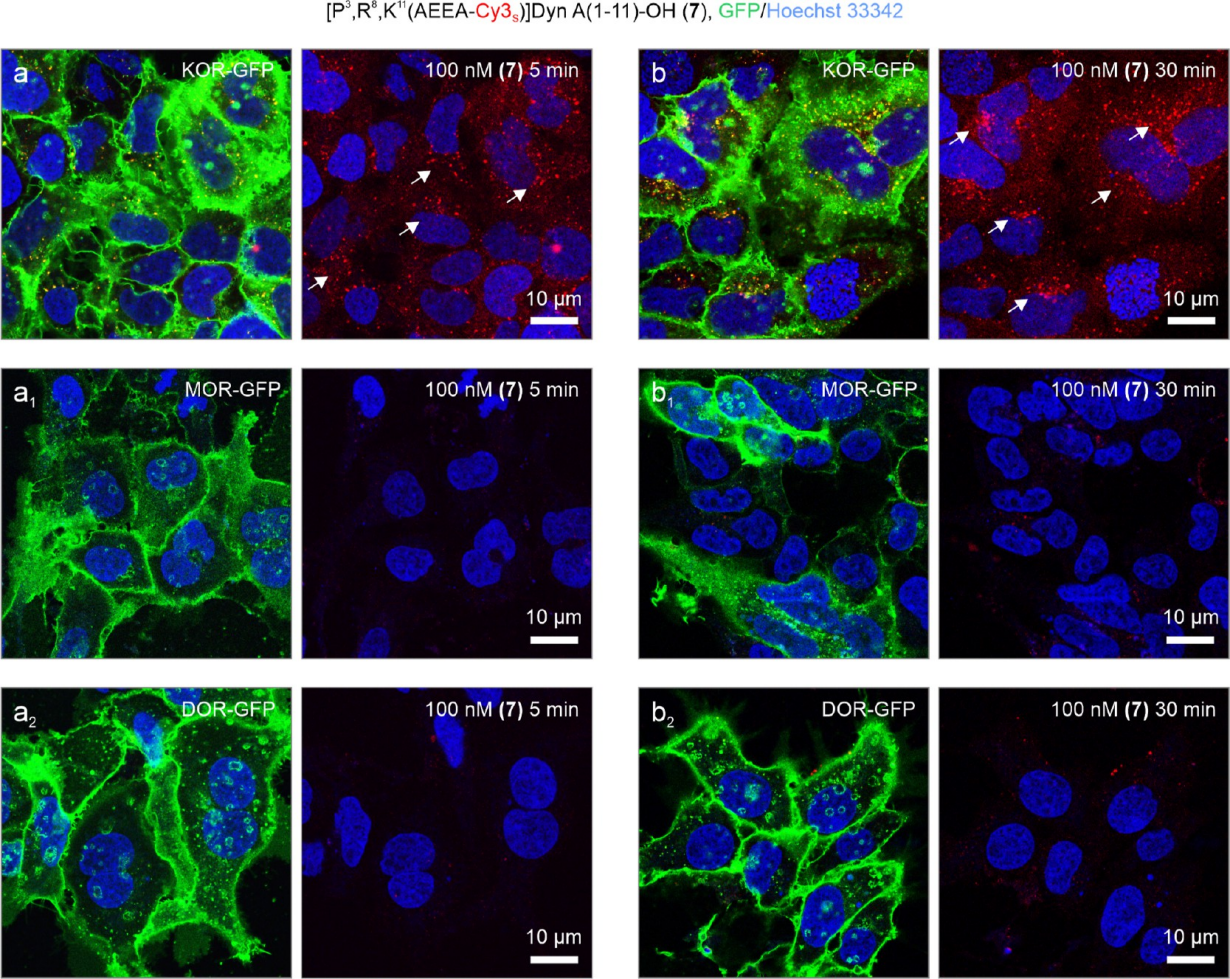
Selective and time-dependent accumulation of lead tracer [P^3^,R^8^,K^11^(AEEA-Cy3_s_)]­Dyn A­(1-11) (**7**) in HEK293 cells stably expressing KOR, but not MOR or DOR. (a,b) Accumulation of 100 nM tracer (**7**) could be detected as early as 5 min post stimulation, with 30 min revealing strong fluorescent signals (red, excitation 560 nm) in KOR-GFP cells, overlapping with mKOR-GFP signals (green, excitation 480 nm). Arrows are marking sites of high tracer accumulation. (a_1_-b_2_) Conversely, tracer (**7**) did not label cells overexpressing either mMOR-GFP or mDOR-GFP, confirming its mKOR selectivity at this concentration. Hoechst 33342 was used as a nuclear counterstain (blue, excitation 405 nm).

### Application in Cell Systems Natively Expressing KOR

We next examined the physiological effects and labeling capacity of tracer (**7**) in biological systems natively expressing KOR. Primary mouse cortical neurons, which express KOR throughout the deep cortical layers,^32^ were cultured and examined at 12 days. Tracer (**7**) stimulation predominantly labeled dendrites in a subpopulation of neurons (Figure 6a,a_1_), with only minor somatic labeling, consistent with a lack of internalization and preserved antagonistic properties. Compared with overexpressing HEK293 cells, higher tracer (**7**) concentrations were required to achieve robust KOR labeling (100 nM vs 500 nM), presumably because of lower endogenous receptor expression levels. Pretreatment with 10 μM KOR antagonist LY-2456302 blocked tracer binding (Figure 6b,b_1_). However, similarly to the KOR-overexpressing HEK293 cultures (Figure S8), we observed tracer accumulation on cellular debris separated from healthy cells, including fragmented nuclei and damaged somas (Figure S13), underscoring the need for caution when interpreting labeled structures in vitro.

**Figure 6:**
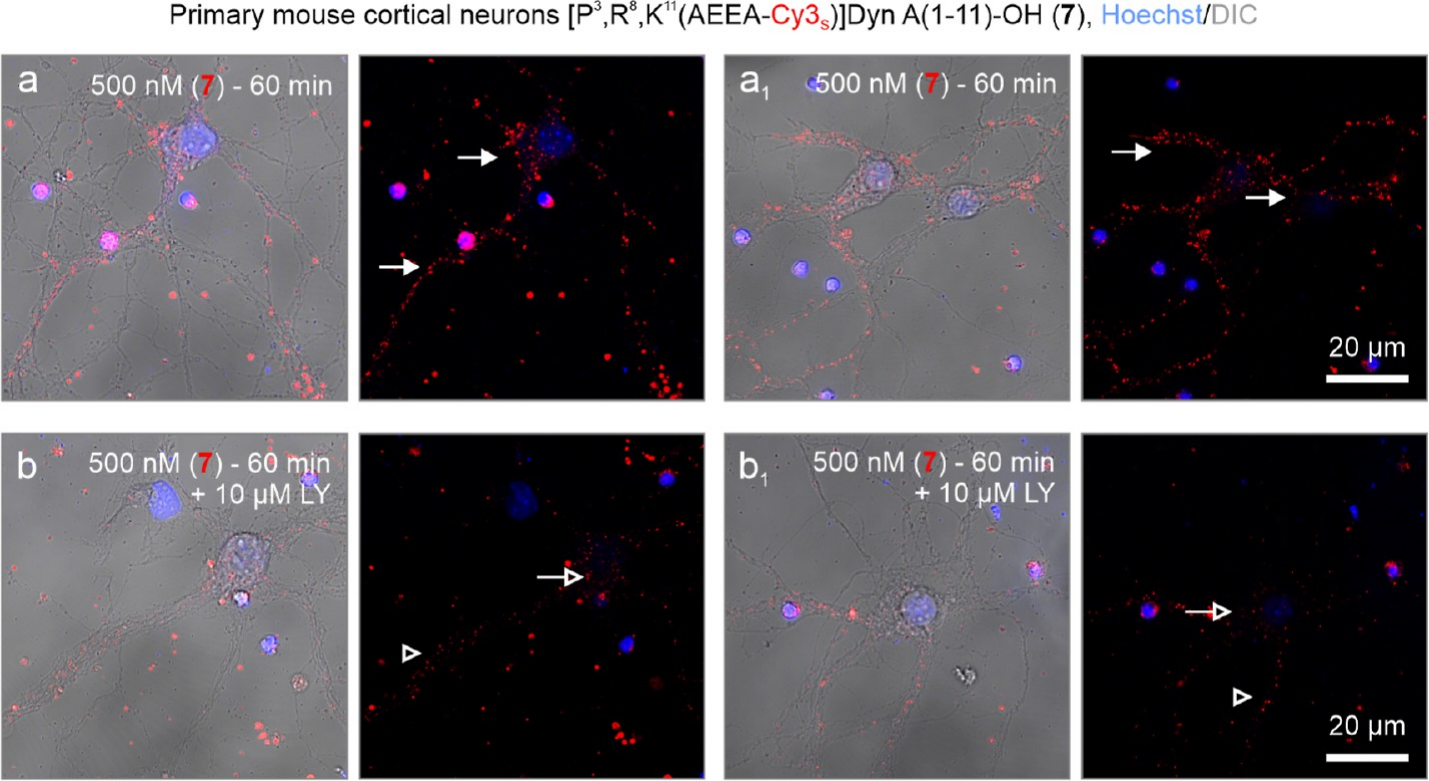
Labeling endogenous mKOR in primary mouse cortical neurons with lead tracer (**7**) [P^3^,R,^8^K^11^(AEEA-Cy3_s_)]­Dyn A­(1-11)-OH. (a-a_1_) Stimulation of primary mouse cortical neurons at 12 days with 500 nM tracer (**7**) revealed somatodendritic labeling in a subpopulation of neurons (red, excitation 560 nm). Images show two representative neurons with arrows marking sites of tracer accumulation. (b-b_1_) Pretreatment with 10 μM KOR antagonist LY-2456302 markedly inhibited tracer binding, with minimal labeling remaining detectable along cell bodies (open arrows) and dendrites (open arrowheads). Note tracer accumulation on cell debris and fragmented nuclei (dense blue labeling, excitation 405 nm), which can be recognized by a lack of cellular structures in the DIC (differential interference contrast; gray in left figure panels). Cellular components remain attached to poly-d-lysine-coated tissue culture surfaces after cell death. Whole cells were visualized with DIC to reveal cellular structures.

Finally, we tested whether our tracer retained its antagonistic properties in the rat superficial spinal cord dorsal horn, where KOR is present on dendrites and somata of laminae I and II neurons,^33^ as well as on rat primary afferent fibers, originating from dorsal root ganglia.^34^ Using whole-cell patch-clamp recordings from unidentified lamina I and II neurons in spinal cord slices, we measured spontaneous excitatory postsynaptic currents (sEPSCs) to assess the tracer’s ability to block KOR activation by KOR-selective agonist U50,488. Under control conditions, sEPSC amplitudes and event rates remained stable across preapplication, application, and postapplication windows (Figures 7a,a_1_, S14a,a_1_), indicating retained slice viability over our experimental time frame. Next, we superfused 10 μM of KOR agonist U50,488 over fresh slices and detected a significant reduction in both sEPSC amplitude (*P* = 0.005 and 0.002) and event rate (*P* = 0.004 and 0.003) (Figure 7b,b_1_), indicative of KOR-mediated reduction of neuronal activity through both pre- and postsynaptic signaling sites. A 30-min pretreatment with 500 nM of tracer (**7**) prevented U50,488-induced reduction in sEPSC event rates, but less so their amplitude, indicating more reliance on presynaptic KOR activity and confirming maintained antagonistic properties of our tracer *ex vivo* (Figure 7c,c_1_). *Post hoc* fixation and subsequent imaging revealed faint somatodendritic labeling of neuronal-like cells in the superficial laminae of the dorsal horn, but not in the control sections (Figure 7d), highlighting its capacity to label cells endogenously expressing KOR.

**Figure 7:**
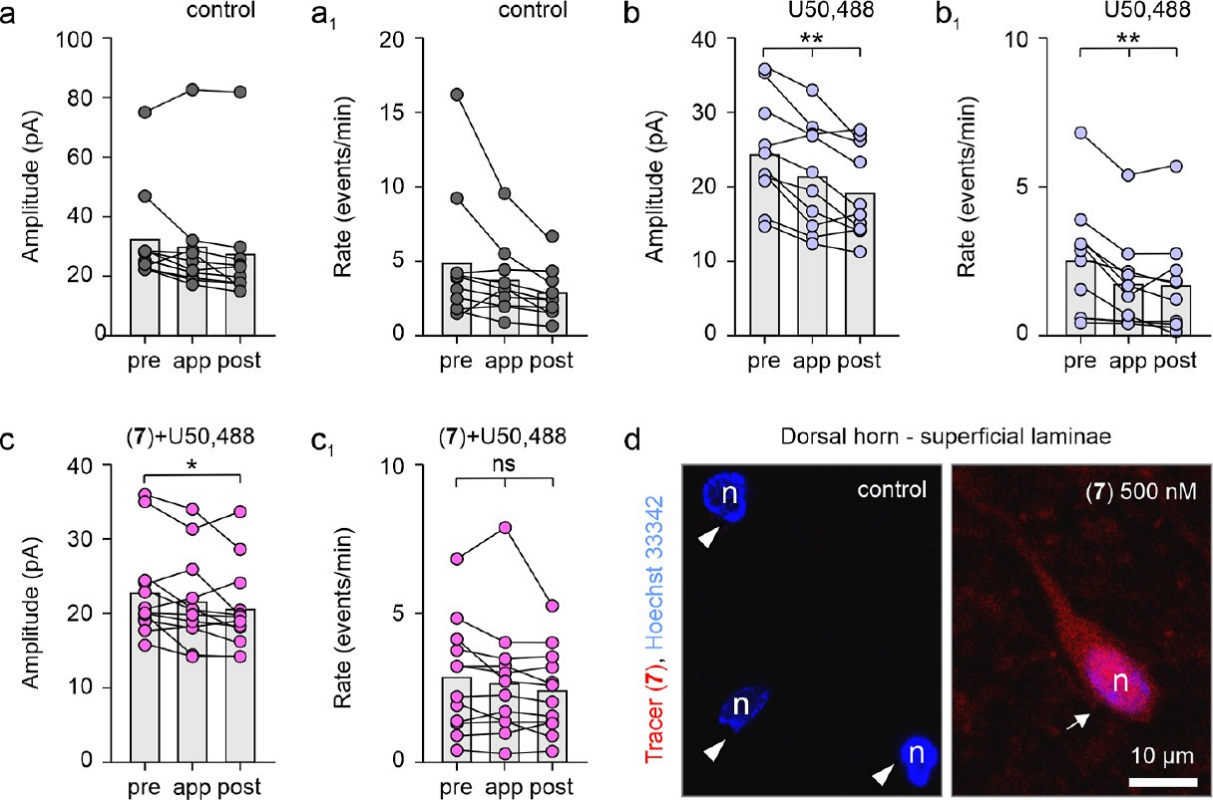
Measurements of spontaneous excitatory postsynaptic currents in neurons of the superficial spinal cord dorsal horn. (a-a_1_) Stable amplitudes and event rates during the preapplication (pre), application (app), and postapplication (post) periods under control settings (*n* ≈ 10 cells per condition). (b-c_1_) Bath application of the KOR agonist U50,488 (10 μM) reduced sEPSCs, which was prevented by pretreatment with 500 nM [P^3^,R^8^,K^11^(AEEA-Cy3_s_)]­Dyn A­(1-11)-OH (**7**). (**d**) *Post hoc* imaging of spinal cord sections revealed somatodendritic labeling (red, excitation 560 nm; arrow marking high tracer accumulation) in superficial laminae neurons. In control sections, tracer was absent (arrowheads marking sites of cell bodies). Sections were counterstained with Hoechst 33342 to reveal nuclei (*n*, blue, excitation 405 nm). *P < 0.05; **P < 0.001; ns, not significant.

## Discussion and Conclusions

The significance of KOR in controlling brain network states, especially for pain management, is gaining traction.^2^ However, selective tools for visualizing receptor localization and trafficking are lacking. For instance, while commercial antibodies can occasionally detect KOR protein in Western blotting, their specificity in immunohistochemistry remains poor.^12^ Alternative approaches focused on genetically fusing a fluorescent tdTomato protein to KOR for anatomical studies and receptor trafficking, but this restricts experimental applicability to native tissues of genetically modified mice.^35^ To circumvent reliance on difficult-to-produce, specific antibodies for GPCRs^13^ and transgenic mouse models,^35^ fluorescent ligands offer an excellent alternative for direct, selective visualization of receptor localization, activity, and movement.^14,15,36^ For example, the fluorescein-labeled KOR agonist arylacetamide visualized the receptor in mouse microglial cells,^37^ while a partial agonist tetrapeptide containing sulforhodamine B at the N-terminal amine, selectively labeled hamster ovarian cells overexpressing KOR, maintaining biological activity.^38^ Attachment of the polarity-sensitive fluorophore aladan to varying amino acid residues of Dyn A resulted in a potent KOR tracer,^39^ but with differing emission spectra depending on the binding location. Dyn A and Big Dyn (a Dyn A precursor) N-terminally labeled with 5-carboxytetramethylrhodamine penetrate plasma membranes and accumulate in the cytosol of both neuronal and non-neuronal cells in a receptor-independent manner.^40^ Opioid receptor affinities of those peptides were not reported. While most of the above compounds are selective for KOR, newer generations of fluorophores, including carbocyanine dyes with increased photostability, pH stability, water solubility, and applicability for high-resolution single-molecule tracking, are considered superior for *post hoc* imaging, especially since fluorescein is highly pH dependent and readily bleaches, and sulforhodamine can aggregate and form complexes.^41,42^ Newer tracer generations use small molecules labeled with Cy3 and Cy5, suitable for high-resolution single-molecule detection microscopy of KOR, but with poor selectivity over MOR and DOR.^43^ The above-mentioned tracers furthermore lack reactive groups for cross-linking, rendering them unusable for prolonged *post hoc* imaging, as they are easily washed away.^36^ Since peptides have sufficient surface area and chirality to provide selectivity and can contain reactive groups for PFA cross-linking, we based our tracer development on dynorphin-based parent peptides and attached a bright carbocyanine dye for easy and reliable *post hoc* fluorescent imaging. We opted for Cy3_s_ due to its enhanced water solubility,^41^ brightness, and peak excitation wavelength of 550 nm, which provides a high signal-to-noise ratio in biological tissues. In addition, Cy3_s_ is spectrally compatible with GFP and allows visual detection with the naked eye (peak emission 570 nm), enabling direct observation with most commercially available epifluorescence and confocal-scanning laser microscopes.

We first attached Cy3_s_ directly to the N-terminal amino acid of [P^3^,R^8^]­Dyn A, inspired by commercially available biotin-Dyn A­(1-17). However, this modification abolished binding to KOR, as determined by both pharmacology and molecular modeling. Attachment of Cy3_s_ to a free lysine at positions 11 or 13 retained KOR binding, resulting in our selective lead tracer (**7**) with nanomolar affinity (*K*_i_ = 27.2 nM) and 368-fold KOR selectivity. [^35^S]-GTPγS binding assays with lead tracer (**7**) revealed weak partial KOR agonism at micromolar concentrations (EC_50_ = 1.2 μM), which was also reported previously for the parent compound (*E*_max_ 21.5%; vs 18% present work).^16^ However, this was considered an artifact of heterologous receptor overexpression as the partial agonism was not observable in endogenous systems.^19^ Similarly, while our lead tracer was internalized via clathrin-mediated endocytosis in HEK293 cells overexpressing KOR, tracer application on cortical primary neurons and spinal cord slices pointed toward retained antagonistic properties of tracer (**7**). Hence, depending on the sample, different tracer properties can be obtained. In silico studies revealed 3D-pharmacophore interactions of both tracer (**7**) and the parent peptide, [P^3^,R^8^]­Dyn A­(1-11)-OH, corresponded well with the pharmacophores derived from cryo-EM structures and are linked to KOR selectivity (e.g., S211 (ECL2) and V108 (2.53))^25^ and KOR antagonism (e.g., Y312 (7.48) and Y320 (7.56)).^25^

Superior imaging compared to small-molecule tracers was demonstrated by its continued, reliable detection over multiple days after PFA fixation, subsequent PBS washes, and nuclear counterstaining.^36^ A decline in fluorescent signals was observed after standard immunohistochemistry protocols, which we ascribed to the presence of detergent (e.g., Triton X-100) in the blocking and antibody solution. Therefore, we suggest detergent titration and shorter incubation times to identify suitable conditions that yield maximum fluorescent signals while still allowing antibody-based labeling in cell cultures or tissue sections. While our tracers were highly selective and readily detectable in overexpressing systems, we also observed that at concentrations ≥1 μM, they accumulated at cellular debris sites and in fragmented nuclei. We attributed this to increased local accumulation of reactive groups upon cell shrinkage and death, especially from sticky cell–surface adhesion and DNA molecules. Aggregates of such reactive molecules, e.g., glycoprotein,^44^ could capture peptide-based tracers, especially when cultures are stimulated in the absence of protein-rich serum to occlude reactive sites, as in primary neuronal cultures. Because endogenously expressed systems have lower KOR levels, increasing tracer concentration would be necessary to detect lower-expressed receptor levels more robustly, at the cost of labeling more cellular debris. In addition, fixation-induced artifacts might occur, as PFA has been linked to loss of membrane integrity^45^ and the dissociation of protein–protein interactions,^46^ leading to misinterpretations of cellular protein localization. We thus caution that, for any in vitro or *ex vivo* system, it is important to examine tracer serial dilutions and to use suitable fixation protocols–since different fixatives affect fluorescent signals^47^–to support the identification of the best signal-to-noise ratio and reliable detection of true signals.

In conclusion, we developed [P^3^,R^8^,K^11^(AEEA-Cy3_s_)]­Dyn A­(1-11)-OH (**7**), a noncytotoxic, bright, water-soluble, and easily detectable fluorescent tracer that can selectively visualize and distinguish KOR from its closely related family members MOR and DOR. Its binding properties are excellent for detecting KOR in cellular membranes without inducing membrane trafficking in native systems, enabling precise localization studies of dynorphin binding sites. The presence of free arginine moieties enables convenient PFA cross-linking, preserving tracer accumulation in biological tissues for *post hoc* imaging analysis. We anticipate that our lead tracer will be a valuable new tool for mapping KOR expression at high-resolution in both physiological and pathophysiological environments.

## Experimental Section

### Ethics

C57BL/6JRj mice and Sprague–Dawley rats (Janvier) were kept under standard housing conditions with a 12/12 h dark/light cycle in humidity- and temperature-controlled rooms. Food and water were provided *ad libitum*. Tissue collection was approved by the Austrian Ministry for Science and Research (2024–0.518.937).

### Synthesis Materials

Fmoc-protected l-amino acids were purchased from Sigma-Aldrich, Merck (Tyr, Gly, Phe, Leu, Arg, Ile, Pro, Lys). AEEA-linker (N_3_-O_2_OC-OH*CHA) and 2-azidoethyl tyrosine (Fmoc- *l* -Tyr­(2-azidoethyl)-OH) were purchased from Iris Biotech. Cy3_s_-alkyne was purchased from Cayman Chemical. SPPS reagents and solvents were purchased from Merck (CuSO_4_, *N,N*-diisopropylethylamine (DIPEA), piperidine, phenylsilane, sodium ascorbate, triisopropylsilane (TIPS)), palladium tretrakis triphenylphosphine was purchased from TCI, diethyl ether and dimethylformamide (DMF) were purchased from VWR Chemicals, HATU from Apollo Scientific, and dichloromethane (DCM) from Thermo Fisher Scientific. Tris­(3-hydroxypropyltriazolylmethyl)­amine (THPTA) was purchased from BLD Pharm, Fmoc-Rink amide aminomethyl (AM) resin (100–200 mesh, loading: 0.74 mmol/g, 1% divinylbenzene (DVB) cross-linking) and Wang resin (100–200 mesh, loading: 0.68 mmol/g, 1% DVB cross-linking) were purchased from Iris Biotech GmbH. Preloaded Wang resins were purchased from Iris Biotech (Wang-Lys­(Boc), Wang-Lys­(Alloc)) and Novabiochem (Wang-Lys­(Mtt)).

### RP-HPLC­(-MS) Analysis and Purification Materials

Acetonitrile (ACN), TFA, and formic acid (FA) were purchased from VWR Chemicals. 2-Propanol was purchased from Sigma-Aldrich, Merck, and methanol was purchased from Honeywell.

### Materials for Pharmacological Assays at Murine Opioid Receptors and Associated Cell Culture

Dulbecco’s Modified Eagle Medium (DMEM, high glucose) and Hank’s Balanced Salt Solution (HBSS) were purchased from Thermo Fisher Scientific, while fetal bovine serum (FBS), G-418 disulfate salt powder, penicillin–streptomycin (P/S), phosphate buffer saline (PBS) tablets, trypsin-ethylenediaminetetraacetic acid (EDTA), 3-isobutyl-1-methylxanthin (IBMX), forskolin, and 2-[4-(2-hydroxyethyl)­piperazin-1-yl]­ethanesulfonic acid (HEPES) were purchased from Sigma-Aldrich. For membrane preparation, albumin standard 2 mg/mL, Pierce bicinchoninic acid protein assay kit (BCA protein assay kit), and Pierce protease inhibitor tablets EDTA-free were purchased from Thermo Fisher Scientific, and a 25 cm cell scraper from Sarstedt. For binding assays, glass microfiber filters (MGB grade, 140 g/m^2^) were purchased from Sartorius Stedim, MgCl_2_*6 H_2_O from Sigma-Aldrich, polyethylenimine from Honeywell, and Rotiszint eco plus LSC Universalcocktail and tris­(hydroxymethyl) aminomethane hydrochloride (TRIS) were purchased from Carl Roth. [^3^H]-DPN and the cAMP detection kit were purchased from Revvity. JetPRIME transfection reagent was purchased from Polyplus, and furimazine was purchased from Promega.

### Materials for Pharmacological Assays at Human KOR and Associated Cell Culture

Guanosine diphosphate (GDP), guanosine 5′-*O*-[gamma-thio]­triphosphate (GTPγS), U69,593, HEPES, bovine serum albumin (BSA), TRIS, and cell culture media and supplements were obtained from Sigma-Aldrich. [^3^H]-U69,593 and [^35^S]-GTPγS were purchased from Revvity.

### Materials for Imaging and Associated Cell Culture and Sample Preparation

DMEM, FBS, HBSS, P/S, sodium pyruvate, trypsin–EDTA, Geneticin, Neurobasal A, 40 μm nylon mesh, GlutaMax, and B27 supplement were purchased from Thermo Fisher Scientific. Poly-d-lysine, PBS, PFA, barbadin, pitstop 2, Hoechst 33342, Triton X-100, DNase, and DMSO were purchased from Sigma-Aldrich. 1,4-diazabicyclo[2.2.2]­octane (DABCO) was purchased from Carl Roth. Normal donkey serum (NDS), antigoat Cy2-conjugated secondary antibody, and antirabbit Cy5-conjugated secondary antibody were purchased from Jackson ImmunoResearch. Fluorescein isothiocyanate (FITC)-conjugated goat-anti-GFP (#AB6662) and MTT solution (#AB146345) were purchased from Abcam. Rabbit-anticleaved caspase-3 was purchased from Cell Signaling Technology (#9661).

### SPPS and General Synthetic Procedure

Peptides were obtained through manual Fmoc-SPPS and labeled in solution following established protocols.^48^ In brief, the respective resins (depending on the desired C-terminus, Rink amide or preloaded Wang resin) were swollen in DMF overnight. 0.1 mmol of synthetic scale was prepared per peptide and assembled from C- to N-terminus using 4 eq. Fmoc-protected amino acids, HATU- and DIPEA-mediated activation and coupling for 15 min, followed by 1 min DMF flow-wash, 2 × 1 min Fmoc deprotection (50% piperidine in DMF), 1 min DMF flow-wash, and the next coupling cycle until the desired amino acid sequence was assembled. All amino acids carried standard Fmoc-SPPS protecting groups, except at the spacer and fluorophore attachment points. To enable coupling via CuAAC, an azide moiety was introduced either directly at Y^1^ (in case of tracers (**1–4**) through Fmoc-Tyr­(CH_2_CH_2_N_3_)), or through application of amino acids with orthogonal protecting groups at K^11^ or K^13^ (in case of tracers (**5**), (**6**), (**8**), and (**10**) through Fmoc-Lys­(Alloc)-OH, and case of tracers (**7**) and (**9**) through Fmoc-Lys­(Mtt)-Wang resin) and subsequent selective deprotection and introduction of azide-carrying spacer N_3_–O_2_OC–OH*CHA. Mtt deprotection was performed by incubating with Mtt deprotection solution (1% TFA and 3% TIPS in DCM) for 5 min, then repeating with fresh solution ∼10 times. Alloc deprotection was carried out with 24 eq phenylsilane and 0.25 eq tretrakis­(triphenylphosphine) palladium in DCM under an inert atmosphere, with incubation for 10 min, followed by a second incubation with a fresh solution. The AEEA spacer was purchased as a cyclohexylamine salt, which required extraction from aqueous hydrochloric acid (pH 2) with DCM. Peptide cleavage and global side chain deprotection were performed by incubating the resin with 90% TFA, 5% TIPS, and 5% ddH_2_O for 2 h, followed by diethyl ether precipitation and lyophilization, yielding crude peptide powder.

Following SPPS, peptides were purified via RP-HPLC on a Waters Auto Purification HPLC-UV system using ddH_2_O with 0.1% TFA (solution A) and ACN with 0.08% TFA (solution B) as the liquid phase. Either a preparative (20–100 mg peptide, Kromasil Classic C_18_ 21.2 × 250 mm, 300 Å, 10 μm) or a semipreparative (1–20 mg peptide, Kromasil Classic C_18_ 10 × 250 mm, 300 Å, 10 μm) column was used with a flow rate of 20 mL/min or 10 mL/min, respectively. Solvents A and B were used as eluents at a linear standard gradient of 5–45% B in 50 min. Fractions were collected based on UV absorption at 214 nm. Coupling of Cy3_s_-alkyne to purified peptides was performed through CuAAC in solution, applying 6 eq THPTA, 6.5 eq CuSO_4_, 10 eq sodium ascorbate, 1 eq peptide-azide, and 1.5 eq Cy3_s_-alkyne in ddH_2_O. The reaction was completed within 1 h and followed by a second RP-HPLC purification to a purity of >95% (Table S1).

### Reaction Monitoring and Quality Control

Reactions were monitored by analytical RP-HPLC-UV-MS at a Thermo Fisher Scientific Dionex Ultimate 3000 system equipped with a Waters XSelect CSH UPLC C_18_ XP column (3.0 × 75 mm, 130 Å, 2.5 μm), UV detector (measurement at 214 and 280 nm), and a Thermo Fisher Scientific MSQ Plus ESI-MS unit in positive ionization mode. Solvents A and B (in case of MS measurements, FA was used instead of TFA) were applied at a flow rate of 1 mL/min with a gradient of 1–61% solvent B in 6 min. Analytic RP-HPLC was employed to determine final product purity on a Thermo Fisher Scientific Vanquish Horizon UHPLC system equipped with a Kromasil Classic C_18_ column (4.6 × 150 mm, 300 Å, 5 μm) at a 1 mL/min solvent flow rate and a linear gradient of 5–65% solvent B in 30 min. UV detection at 214 nm and peak integration, followed by comparison of the product and side-peak peak areas, provided information on the final product’s purity.

### Determination of Peptide Concentration

A Thermo Fisher Scientific Vanquish Horizon UHPLC system equipped with a designated quality control column (Kromasil Classic C_18_ 2.1 × 100 mm, 100 Å, 5 μm) was used to determine the product concentration, following established protocols.^48,49^ A solvent gradient from 5% to 65% B over 10 min at a flow rate of 1 mL/min was applied.

### Cell Culture, Membrane Preparation, and Radioligand Binding Assay using Stable HEK293 Cell Lines Expressing Murine Opioid Receptors

Cells were cultured in DMEM high glucose supplemented with 10% FBS and Geneticin (0.5 mg/mL) in a humidified atmosphere containing 5% CO_2_ at 37 °C. Membranes for radioligand binding assays were isolated from HEK293 cells stably expressing GFP-tagged mKOR, mMOR, or mDOR as described.^22^ In short, cells were detached from the dish into PBS (pH 7.4), then centrifuged and resuspended in TRIS buffer (50 mM TRIS, 5 mM MgCl_2_, 0.1% BSA, pH 7.4 at 37 °C) containing protease inhibitor. The resuspended cells were disrupted in a water bath sonicator, and the membranes isolated by centrifugation at 52,192 x *g* for 20 min, followed by homogenization with an ultrasonication stick. Membrane aliquots were stored at −80 °C, and the protein concentration was determined via BCA assay. Radioligand binding assays were carried out according to established procedures.^22^ Competing ligand, radioligand, and membrane preparation were incubated in TRIS buffer (end volume 300 μL) for 1 h at 37 °C. Afterward, membranes were filtered on a Skatron cell harvester using 0.1% polyethylenimine-coated glass fiber sheets and a TRIS washing solution (10 mM TRIS, 1 mM MgCl_2_, pH 7.4 at 4 °C). Radioactivity counting was performed on a Tri-Carb liquid scintillation analyzer. Specific binding was obtained by subtracting nonspecific binding at 10 μM naloxone (nonselective opioid antagonist) from total binding. The nonselective opioid antagonist [^3^H]-DPN was applied at 1 nM at all three receptors, and membrane concentrations were 7, 40, and 20 μg/vial for bindings at mKOR, mMOR, or mDOR, respectively. The *K*_d_ and *B*_max_ values at mKOR (0.87 nM and 7.2 pmol/mg) were determined,^21^ while *K*_d_ values at mMOR (0.81 nM) and mDOR (1.7 nM) were taken from the literature.^22^ Radioligand binding assays were performed in duplicates (*n* = 2) across at least three independent experiments (*N* ≥ 3). The *K*_i_ values were obtained from fitting serial dilutions to a three-parameter logistic Hill equation and applying the Cheng-Prusoff approximation.^50^

### Cell Culture, Membrane Preparation, and Radioligand Binding Assay using Stable CHO Cell Lines Expressing the Human KOR

The CHO cell line stably expressing the human KOR (CHO-hKOR) was kindly provided by Dr. Lawrence Toll (SRI International). The CHO-hKOR cell line was maintained in DMEM supplemented with 10% FBS, 0.1% P/S, 2 mM l-glutamine, and Geneticin (0.4 mg/mL). Cells were maintained at 37 °C in 5% CO_2_ humidified air. Cell membranes were prepared as previously described.^51^ Briefly, cells were scraped into 50 mM TRIS buffer (pH 7.7), homogenized with a Dounce glass homogenizer, centrifuged once, and then washed by an additional centrifugation at 27,000 x *g* for 15 min at 4 °C. The final pellet was resuspended in 50 mM TRIS buffer (pH 7.7) and stored at −80 °C until use. The protein content of cell membrane preparations was determined by the Bradford method using BSA as the standard.^52^ Radioligand competitive binding assays were conducted on a stable hKOR cell line according to the published procedure.^51^ Assays were performed in 50 mM TRIS buffer (pH 7.4) in a final volume of 1 mL. Cell membranes (20 μg) were incubated with various concentrations of test compounds and [^3^H]-U69,593 (1 nM) for 60 min at 25 °C. Nonspecific binding was determined using 10 μM of U69,593. After incubation, reactions were terminated by rapid filtration through Whatman GF/C glass fiber filters using a Brandel M24R cell harvester. Radioactivity was counted by liquid scintillation counting using a Beckman Coulter LS6500. Inhibition constant (*K*_i_, nM) values were determined by the method of Cheng and Prusoff from concentration–response curves by nonlinear regression analysis.^50^ All experiments were performed in duplicate (*n* = 2) and repeated at least three times (*N* ≥ 3) with independently prepared samples.

### cAMP Assay

G_i_ protein activation was determined using established protocols^53^ and a commercially available cAMP detection kit. Briefly, cells were seeded at 2000 cells/well in 384-well plates using stimulation buffer (5 mM HEPES and 0.1% BSA in HBSS, pH 7.4) supplemented with IBMX (0.5 mM). Test ligand dilutions were prepared in stimulation buffer supplemented with IBMX (0.5 mM) and forskolin (10 μM end concentration), added to wells containing cell suspension, and incubated for 30 min at 37 °C. Afterward, labeled cAMP and labeled anti-cAMP antibody were diluted with lysis buffer, added to the wells, and the plate was incubated for 1 h at 25 °C. Cellular cAMP levels were measured through homogeneous time-resolved fluorescence (HRTF) on a Tecan Spark plate reader. Curves were fitted to a three-parameter nonlinear regression and normalized to a saturating concentration (1 μM) of control agonist U50,488. Data correspond to at least three independent (*N* ≥ 3) measurements in technical triplicate (*n* = 3).

### [^35^S]-GTPγS Binding Assay

Binding of [^35^S]-GTPγS to membranes from CHO cells stably expressing hKOR was conducted according to a published procedure.^51^ In short, cell membranes (10–15 μg) in Buffer A (20 mM HEPES, 10 mM MgCl_2,_ and 100 mM NaCl, pH 7.4) were incubated with 0.05 nM [^35^S]-GTPγS, 3 μM GDP, and test compounds in a final volume of 1 mL, for 60 min at 25 °C. Nonspecific binding was determined using 10 μM GTPγS, and the basal binding was determined in the absence of the test compound. Samples were filtered over Whatman GF/B glass fiber filters using a Brandel M24R cell harvester (Brandel). Radioactivity was counted by liquid scintillation using a Beckman Coulter LS6500 (Beckman Coulter). The obtained concentration–response curves were subjected to nonlinear regression analysis. Experiments were performed in duplicate (*n* = 2) and repeated at least three times (*N* ≥ 3).

### β-arrestin Recruitment Assay

HEK293 cells were cotransfected with 0.2 μg of a pcDNA3.1 (+) vector carrying human β-arrestin-2 C-terminally tagged with nanoluciferase and 1.8 μg of pEGFP-N1 vector containing mKOR with a C-terminal GFP tag. Transfection was performed using jetPRIME transfection reagent according to the manufacturer’s protocol. After 6 h of incubation, the transfected cells were transferred into white, clear-bottom 96-well plates containing DMEM supplemented with 10% FBS and 1% P/S at 10,000 cells per well. After 12 h of incubation, the cells were serum-starved for 1 h prior to the addition of diluted furimazine (50-fold diluted in HBSS). After 5 min incubation at 37 °C, test compounds were added, and BRET was recorded for a total of 25 min at 37 °C and 460 nM (nanoluciferase) and 510 nM (GFP) using a FlexStation 3 (Molecular Devices). BRET ratios were calculated (acceptor/donor), and the assay was carried out as technical duplicate (*n* = 2).

### Molecular Modeling

Cryo-EM resolved structures of Dyn A­(1-8) and NorBNI (PDB IDs: 8F7W^24^ and 8VVE,^25^ respectively) were used for the calculation of chemical feature-based pharmacophore interactions using LigandScout XT.^26^ 3D-pharmacophores were generated using the program default settings. LigandScout default distance cutoff settings, hydrogen bonds, and positive ionizable-to-negative ionizable interactions are described in the LigandScout manual. Tracers (**1–10**) were docked into each binding site using AutoDock Vina,^54,55^ as implemented in LigandScout XT (default settings). Poses were prioritized using the multiparameter based scoring as implemented in LigandScout XT, which is based on the total number of 3D-interaction features of each resulting docked pose with binding site amino acids, the geometric 3D-pharmacophore interaction feature alignment score of each pose to the cryo-EM derived 3D-interaction features of Dyn A­(1-8) and NorBNI, in each binding site respectively, the AutoDock Vina score and the relative binding affinity score calculated by LigandScout XT. The docked poses of tracers (**5–10**) with the best combined scores were prioritized for the 3D-chemical feature analysis done with default settings in LigandScout XT. In addition, 3D-pharmacophore feature alignments were performed by calculating low energy conformations of tracers (**1–10**) (default settings using iCon and Conforge conformation generation algorithms in LigandScout XT) and aligning the pharmacophore features of the ligands to the Dyn A­(1-8) and NorBNI pharmacophores derived from the cryo-EM resolved structures to evaluate reasonable low energy conformations to represent KOR binding. The best-aligned conformations were evaluated in both cryo-EM-resolved KOR binding sites and in new interaction models generated. This was done to overcome limitations in conformational sampling in docking programs and is known as pharmacophore docking.^26^

### Cell Culture and Imaging

Stably expressing mouse KOR-GFP, MOR-GFP, or DOR-GFP HEK293 ^22^ cells were grown in DMEM, 10% FBS, 1 mM sodium pyruvate, and Geneticin (500 μg/mL), at 37 °C and 5% CO_2_. Parent HEK293 cells were grown in the above medium without Geneticin, but with P/S as an alternative antibiotic. For experiments, trypsin/EDTA (0.1%) dissociated cells were plated at a 10,000 cells/well density on poly-d-lysine-coated black glass-bottom 96-well plates (Cellvis) and grown overnight in a full growth medium. The next day, the medium was replaced with serum-free medium to synchronize the cell cycle and enhance sensitivity to external stimuli. Cells were treated with concentrations ranging from 10 nM to 10 μM of tracers up to 60 min (*n* = 2–3 wells per condition) in the absence or presence of the KOR antagonist LY-2456302 (10 μM).^27^ For endocytosis inhibition, we pretreated the cells with barbadin (50 μM for 30 min)^28^ or pitstop 2 (10 μM for 15 min).^29^

Subsequently, cells were washed and fixed in 4% PFA in PBS for 30 min on ice. Afterward, another washed with PBS, counterstained with the nuclear dye Hoechst 33342, and either mounted with Mowiol mounting medium, containing the antifading agent DABCO, or directly imaged in PBS. For immunohistochemistry, cells were incubated with 5% NDS, 2% BSA, and 0.2% Triton X-100 in PBS at room temperature (25 °C) for 1 h to block nonspecific antibody binding. Next, cells were incubated in 2% NDS, 0.1% BSA, 0.2% Triton X-100 in PBS with FITC conjugated goat-anti-GFP (1:1000) and rabbit-anticleaved caspase-3 (1:300) primary antibodies in PBS overnight at 4 °C. The following day, the cells were washed with PBS and incubated with 2% BSA in PBS containing antigoat Cy2 and antirabbit Cy5 (1:500) conjugated secondary antibodies for 2 h at room temperature. Finally, cells were washed and counterstained with Hoechst 33342 (1:10,000) before mounting in PBS. Cells were then directly imaged on a Zeiss LSM 780 or 880 confocal microscope (Zeiss) with excitation of 405 nm to excite Hoechst 33342, 480 nm for GFP, 560 nm to visualize tracers and 647 nm for cleaved caspase-3. Images were analyzed (fluorescent intensity) and equally contrasted for visualization with ZEN Pro (Zeiss) and collated in CorelDraw 2019 (v.21.0.0.593).

### MTT Assay

A 3-(4,5-dimethylthiazol-2-yl)–2,5-diphenyltetrazolium bromide colorimetric assay was performed either 30 or 60 min post-stimulation at tracer (**7**) concentrations up to 10 μM (*n* = 3 cells per condition). After stimulation, cells were washed and incubated with MTT solution at a final concentration of 0.5 mg/mL for 3 h. Cells were subsequently washed and 100 μL of 100% DMSO was added to each well to dissolve the precipitated formazan crystals. After a brief shake, absorbance was determined spectrophotometrically at 570 nm to detect solubilized formazan.

### Primary Mouse Cortical Cultures

Fetal cortices from embryonic day E17.5 pregnant C57BL/6JRj mice were dissected and mechanically dissociated with flamed Pasteur pipettes in ice-cold HBSS. Tissues were further enzymatically dissociated in 0.1% trypsin/EDTA in Neurobasal A containing 1000 U/mL DNase for 5 min at 37 °C.^56^ Neurons were subsequently triturated with flamed Pasteur pipettes and filtered through a 40 μm nylon mesh before centrifugation at 200 x *g* for 5 min. Next, cells were washed up to three times in Neurobasal A containing penicillin/streptomycin, GlutaMax, and B27 supplement. Neurons were plated at a density of 25,000 cells/well on poly-d-lysine-coated glass-bottom black 96-well microscopy plates (Cellvis) and grown at 37 °C with 5% CO_2_ until 12 days in vitro. Half of the medium was replaced every 3–4 days. Mature cultures were exposed to 500 nM tracer for 1 h (*n* = 3 wells per condition), with or without a 30 min pretreatment with 10 μM of the KOR antagonist LY-2456302. After stimulation, cells were washed with ice-cold PBS, fixed with 4% PFA for 30 min on ice, washed, and counterstained with the nuclear marker Hoechst 33342 (1:5000 in PBS) for 10 min. Cultures were washed with PBS and directly imaged on a Zeiss LSM880 confocal microscope.

### Spinal Cord Slice Preparation and Patch-Clamp Electrophysiology

Transversal spinal cord slices (500 μm) were prepared from male and female SpragueDawley rats aged 35–55 days, as described previously.^57^ Slices were transferred to a recording chamber and continuously perfused with oxygenated recording solution containing: 127 mM NaCl, 1.8 mM KCl, 1.2 mM KH_2_PO_4_, 2.4 mM CaCl_2_, 1.3 mM MgSO_4_, 26 mM NaHCO_3_, 15 mM glucose, a pH of 7.4, and a measured osmolarity of 310–320 milliosmoles per liter. Cells were recorded at room temperature in the whole-cell patch-clamp configuration using glass pipettes (resistance = 2–4 MΩ) filled with internal solution containing: 120 mM potassium gluconate, 20 mM KCl, 2 mM MgCl_2_, 20 mM HEPES, 0.5 mM Na-GTP, 0.5 mM Na_4_-EGTA, 2 mM Na_2_-ATP, 7.5 mM phosphocreatine disodium salt hydrate, pH 7.4 adjusted with KOH, and measured osmolarity 310 milliosmoles per liter. Spontaneous excitatory postsynaptic currents (sEPSCs) were recorded at a holding potential of −70 mV from unidentified neurons in laminae I and II using an Axopatch B700 patch-clamp amplifier (Axon Instruments) and pClamp10 software. Signals were low-pass filtered at 2–10 kHz, digitized at 20 kHz, and analyzed offline. No correction was made for the liquid junction potential. Drugs were bath-applied (application period, *app*) at final concentrations of 500 nM (fluorescent tracer), 10 μM (U50,488), or 10 μM (DAMGO) following a 5 min baseline period (predrug period, pre), followed by washout of the drug (*n* = 8–11 cells per condition; postdrug period, post). To verify our system, we bath applied 10 μM DAMGO, a selective MOR agonist, and observed reduced amplitude and event rate, consistent with the well-established postsynaptic and presynaptic inhibition,^58^ confirming the reliability of our approach (*n* = 6 cells per condition; Supporting Information Figure S14). Next, for experiments verifying the antagonistic properties of the tracer, slices were preincubated for ≥30 min in recording solution containing 500 nM tracer before being transferred to the recording chamber. During recordings, slices were superfused with the same tracer-containing solution. After recordings, sections were fixed with 4% PFA in PBS, counterstained with Hoechst 33342, and mounted with Fluorescent Mounting Medium before imaging on a Zeiss LSM880 microscope.

### Data Analysis and Statistics

Experimental data from pharmacological assays were analyzed and graphically displayed using GraphPad Prism (GraphPad Prism Software Inc.). Data are presented as means ± SD or SEM where indicated. sEPSC amplitudes and event rates across the pre-, app-, and postdrug periods were analyzed using one-way repeated-measures ANOVA followed by Dunnett’s multiple comparisons test in GraphPad Prism. Fluorescent intensities were tested with Student *t* tests, and the cytotoxicity assays with a one-way ANOVA (Tukey’s *post hoc* test).

## Supplementary Material







## Notes

Docking pose of selected tracer (ZIP)
Molecular formula strings of the final tracer with biological activity data (CSV)
Synthesis summary for all compounds. Structures, purities, radioligand displacement assays and HRMS. Tracer selectivity comparisons and analysis. KOR-mediated downstream signaling events. Concentration-dependent background labeling in HEK293 cells. Labeling of cellular debris. Cytotoxicity assays. Endocytosis inhibition. Positive controls for spinal cord measurements (PDF)

## Abbreviations

ACN: acetonitrile
AEEA: (2-[2-(2-azidoethoxy)­ethoxy]­acetic acid)
AM: aminomethyl
BCA: bicinchoninic acid
BRET: bioluminescence resonance energy transfer
BSA: bovine serum albumin
cAMP: 3′,5′-cyclic adenosine monophosphate
CHO: chinese hamster ovary cells
CNS: central nervous system
CuAAC: copper-catalyzed azide–alkyne cycloaddition
Cy2/3/5: carbocyanine dye
Cy2/3/5_s_: sulfonated carbocyanine dye
DABCO: 1,4-diazabicyclo­[2.2.2]­octane
DADLE: [d-Ala^2^,d-Leu^5^]-enkephalin
DAMGO: [d-Ala^2^,*N*-MePhe^4^,Gly-ol]-enkephalin
DCM: dichloromethane
DIC: differential interference contrast
DIPEA: *N,N*-diisopropylethylamine
DMF: dimethylformamide
DOR: delta opioid receptor
DPN: diprenorphine
DVB: divinylbenzene
Dyn: dynorphin
FA: formic acid
FBS: fetal bovine serum
FITC: fluorescein isothiocyanate
Fmoc-SPPS: 9-fluorenylmethoxycarbonyl-solid-phase peptide synthesis
FRET: fluorescence resonance energy transfer
GDP: guanosine diphosphate
GFP: green fluorescent protein
GPCR: G protein-coupled receptor
GTPγS: guanosine 5′-*O*-[gamma-thio]­triphosphate
HATU: hexafluorophosphate azabenzotriazole tetramethyl uronium
HBSS: hank’s balanced salt solution
HEK293: human embryonic kidney cells
HEPES: 2-[4-(2-hydroxyethyl)­piperazin-1-yl]­ethanesulfonic acid
HR-ESI-MS: high-resolution electrospray mass spectrometry
HRTF: homogeneous time-resolved fluorescence
IBMX: 3-isobutyl-1-methylxanthin
KOR: kappa opioid receptor
LY-2456302: aticaprant—KOR antagonist
MOR: mu opioid receptor
NDS: normal donkey serum
NorBNI: norbinaltorphimine
PDL: poly-d-lysine
PFA: paraformaldehyde
RP-HPLC: reversed-phase high-performance liquid chromatography
SAR: structure−affinity-relationship
sEPSCs: spontaneous excitatory postsynaptic currents
TFA: trifluoroacetic acid
THPTA: tris­(3-hydroxypropyltriazolylmethyl)­amine
TIPS: triisopropylsilane
U50,488: KOR agonist
U69,593: KOR agonist.
