# Supplementary material for: Development and Biological Characterization of Fluorescent Dynorphins for the Visualization of Kappa Opioid Receptors
Source: J Med Chem. 2026 Jun 5;69(12):14098–113. doi: 10.1021/acs.jmedchem.5c03072 (PMC13312453; doi:10.1021/acs.jmedchem.5c03072)
Supplement: Supplementary file 1 [file jm5c03072_si_001.zip › Manuscript-PDB-Files-Fig2-Fig3/SI-PDB-text-information.docx]

**SI PDB Files Text for Models Reported in Figures 2 and 3.**

**1. PDB Text: Figure 2 KOR (8f7w) with Compound 7**

**CRYST1 0.000 0.000 0.000 90.00 90.00 90.00 P 1 1**

**ATOM 1 CA TYR R 66 125.416 111.307 129.813 1.00 0.00 C**

**ATOM 2 C TYR R 66 125.567 112.260 128.626 1.00 0.00 C**

**ATOM 3 O TYR R 66 124.699 112.297 127.745 1.00 0.00 O**

**ATOM 4 N TYR R 66 126.660 110.597 130.131 1.00 0.00 N**

**ATOM 5 CB TYR R 66 124.900 112.079 131.031 1.00 0.00 C**

**ATOM 6 CG TYR R 66 124.024 111.281 131.978 1.00 0.00 C**

**ATOM 7 CD1 TYR R 66 122.678 111.082 131.693 1.00 0.00 C**

**ATOM 8 CE1 TYR R 66 121.857 110.372 132.554 1.00 0.00 C**

**ATOM 9 CZ TYR R 66 122.380 109.864 133.723 1.00 0.00 C**

**ATOM 10 OH TYR R 66 121.567 109.154 134.580 1.00 0.00 O**

**ATOM 11 CE2 TYR R 66 123.711 110.057 134.040 1.00 0.00 C**

**ATOM 12 CD2 TYR R 66 124.523 110.771 133.169 1.00 0.00 C**

**ATOM 13 CA TYR R 87 115.508 112.609 101.104 1.00 0.00 C**

**ATOM 14 C TYR R 87 114.531 111.549 100.598 1.00 0.00 C**

**ATOM 15 O TYR R 87 114.539 111.212 99.409 1.00 0.00 O**

**ATOM 16 N TYR R 87 114.884 113.574 102.013 1.00 0.00 N**

**ATOM 17 CB TYR R 87 116.713 111.953 101.774 1.00 0.00 C**

**ATOM 18 CG TYR R 87 117.926 112.852 101.845 1.00 0.00 C**

**ATOM 19 CD1 TYR R 87 118.883 112.834 100.835 1.00 0.00 C**

**ATOM 20 CE1 TYR R 87 119.995 113.655 100.889 1.00 0.00 C**

**ATOM 21 CZ TYR R 87 120.159 114.509 101.960 1.00 0.00 C**

**ATOM 22 OH TYR R 87 121.264 115.327 102.016 1.00 0.00 O**

**ATOM 23 CE2 TYR R 87 119.222 114.549 102.975 1.00 0.00 C**

**ATOM 24 CD2 TYR R 87 118.113 113.723 102.912 1.00 0.00 C**

**ATOM 25 CA TYR R 97 106.940 104.103 111.643 1.00 0.00 C**

**ATOM 26 C TYR R 97 108.311 104.595 112.109 1.00 0.00 C**

**ATOM 27 O TYR R 97 108.471 104.983 113.274 1.00 0.00 O**

**ATOM 28 N TYR R 97 106.585 104.622 110.318 1.00 0.00 N**

**ATOM 29 CB TYR R 97 106.882 102.574 111.664 1.00 0.00 C**

**ATOM 30 CG TYR R 97 105.487 101.996 111.792 1.00 0.00 C**

**ATOM 31 CD1 TYR R 97 104.835 101.991 113.020 1.00 0.00 C**

**ATOM 32 CE1 TYR R 97 103.565 101.459 113.157 1.00 0.00 C**

**ATOM 33 CZ TYR R 97 102.941 100.902 112.060 1.00 0.00 C**

**ATOM 34 OH TYR R 97 101.681 100.365 112.190 1.00 0.00 O**

**ATOM 35 CE2 TYR R 97 103.573 100.878 110.832 1.00 0.00 C**

**ATOM 36 CD2 TYR R 97 104.843 101.416 110.708 1.00 0.00 C**

**ATOM 37 CA TYR R 119 121.729 114.543 139.772 1.00 0.00 C**

**ATOM 38 C TYR R 119 122.878 115.333 140.398 1.00 0.00 C**

**ATOM 39 O TYR R 119 123.213 115.133 141.571 1.00 0.00 O**

**ATOM 40 N TYR R 119 120.754 115.414 139.116 1.00 0.00 N**

**ATOM 41 CB TYR R 119 122.272 113.510 138.785 1.00 0.00 C**

**ATOM 42 CG TYR R 119 123.464 112.752 139.329 1.00 0.00 C**

**ATOM 43 CD1 TYR R 119 123.323 111.883 140.406 1.00 0.00 C**

**ATOM 44 CE1 TYR R 119 124.407 111.189 140.914 1.00 0.00 C**

**ATOM 45 CZ TYR R 119 125.652 111.357 140.341 1.00 0.00 C**

**ATOM 46 OH TYR R 119 126.731 110.666 140.844 1.00 0.00 O**

**ATOM 47 CE2 TYR R 119 125.820 112.213 139.269 1.00 0.00 C**

**ATOM 48 CD2 TYR R 119 124.727 112.904 138.769 1.00 0.00 C**

**ATOM 49 CA TYR R 139 106.459 110.053 130.061 1.00 0.00 C**

**ATOM 50 C TYR R 139 105.717 109.750 128.760 1.00 0.00 C**

**ATOM 51 O TYR R 139 105.679 108.592 128.309 1.00 0.00 O**

**ATOM 52 N TYR R 139 107.367 111.195 129.917 1.00 0.00 N**

**ATOM 53 CB TYR R 139 105.468 110.296 131.203 1.00 0.00 C**

**ATOM 54 CG TYR R 139 106.027 110.038 132.588 1.00 0.00 C**

**ATOM 55 CD1 TYR R 139 105.659 108.901 133.299 1.00 0.00 C**

**ATOM 56 CE1 TYR R 139 106.159 108.654 134.566 1.00 0.00 C**

**ATOM 57 CZ TYR R 139 107.034 109.553 135.140 1.00 0.00 C**

**ATOM 58 OH TYR R 139 107.532 109.309 136.400 1.00 0.00 O**

**ATOM 59 CE2 TYR R 139 107.412 110.693 134.459 1.00 0.00 C**

**ATOM 60 CD2 TYR R 139 106.907 110.931 133.189 1.00 0.00 C**

**ATOM 61 CA TYR R 140 104.303 110.705 126.953 1.00 0.00 C**

**ATOM 62 C TYR R 140 105.105 110.066 125.820 1.00 0.00 C**

**ATOM 63 O TYR R 140 104.701 109.043 125.254 1.00 0.00 O**

**ATOM 64 N TYR R 140 105.058 110.776 128.200 1.00 0.00 N**

**ATOM 65 CB TYR R 140 103.875 112.134 126.604 1.00 0.00 C**

**ATOM 66 CG TYR R 140 103.084 112.344 125.340 1.00 0.00 C**

**ATOM 67 CD1 TYR R 140 101.708 112.180 125.338 1.00 0.00 C**

**ATOM 68 CE1 TYR R 140 100.969 112.399 124.201 1.00 0.00 C**

**ATOM 69 CZ TYR R 140 101.588 112.810 123.050 1.00 0.00 C**

**ATOM 70 OH TYR R 140 100.812 113.024 121.937 1.00 0.00 O**

**ATOM 71 CE2 TYR R 140 102.955 113.009 123.024 1.00 0.00 C**

**ATOM 72 CD2 TYR R 140 103.696 112.784 124.171 1.00 0.00 C**

**ATOM 73 CA TYR R 157 104.831 89.714 112.263 1.00 0.00 C**

**ATOM 74 C TYR R 157 103.683 89.380 111.317 1.00 0.00 C**

**ATOM 75 O TYR R 157 103.620 88.273 110.771 1.00 0.00 O**

**ATOM 76 N TYR R 157 105.361 91.050 111.994 1.00 0.00 N**

**ATOM 77 CB TYR R 157 104.376 89.620 113.721 1.00 0.00 C**

**ATOM 78 CG TYR R 157 103.531 88.405 114.048 1.00 0.00 C**

**ATOM 79 CD1 TYR R 157 104.110 87.151 114.204 1.00 0.00 C**

**ATOM 80 CE1 TYR R 157 103.340 86.042 114.514 1.00 0.00 C**

**ATOM 81 CZ TYR R 157 101.975 86.181 114.671 1.00 0.00 C**

**ATOM 82 OH TYR R 157 101.203 85.084 114.973 1.00 0.00 O**

**ATOM 83 CE2 TYR R 157 101.376 87.415 114.526 1.00 0.00 C**

**ATOM 84 CD2 TYR R 157 102.154 88.519 114.222 1.00 0.00 C**

**ATOM 85 CA TYR R 219 101.031 108.686 148.819 1.00 0.00 C**

**ATOM 86 C TYR R 219 101.804 107.431 148.439 1.00 0.00 C**

**ATOM 87 O TYR R 219 102.017 107.171 147.247 1.00 0.00 O**

**ATOM 88 N TYR R 219 100.029 108.415 149.846 1.00 0.00 N**

**ATOM 89 CB TYR R 219 102.002 109.766 149.303 1.00 0.00 C**

**ATOM 90 CG TYR R 219 103.034 110.172 148.269 1.00 0.00 C**

**ATOM 91 CD1 TYR R 219 102.708 111.045 147.242 1.00 0.00 C**

**ATOM 92 CE1 TYR R 219 103.643 111.414 146.290 1.00 0.00 C**

**ATOM 93 CZ TYR R 219 104.923 110.904 146.359 1.00 0.00 C**

**ATOM 94 OH TYR R 219 105.857 111.268 145.416 1.00 0.00 O**

**ATOM 95 CE2 TYR R 219 105.274 110.030 147.369 1.00 0.00 C**

**ATOM 96 CD2 TYR R 219 104.333 109.672 148.317 1.00 0.00 C**

**ATOM 97 CA TYR R 246 109.707 87.440 116.493 1.00 0.00 C**

**ATOM 98 C TYR R 246 109.994 85.941 116.542 1.00 0.00 C**

**ATOM 99 O TYR R 246 110.587 85.411 115.595 1.00 0.00 O**

**ATOM 100 N TYR R 246 108.466 87.803 117.176 1.00 0.00 N**

**ATOM 101 CB TYR R 246 110.885 88.237 117.047 1.00 0.00 C**

**ATOM 102 CG TYR R 246 110.888 89.682 116.597 1.00 0.00 C**

**ATOM 103 CD1 TYR R 246 110.989 90.003 115.247 1.00 0.00 C**

**ATOM 104 CE1 TYR R 246 110.995 91.320 114.822 1.00 0.00 C**

**ATOM 105 CZ TYR R 246 110.907 92.334 115.753 1.00 0.00 C**

**ATOM 106 OH TYR R 246 110.915 93.646 115.338 1.00 0.00 O**

**ATOM 107 CE2 TYR R 246 110.811 92.041 117.098 1.00 0.00 C**

**ATOM 108 CD2 TYR R 246 110.806 90.719 117.511 1.00 0.00 C**

**ATOM 109 CA TYR R 312 120.532 104.023 139.757 1.00 0.00 C**

**ATOM 110 C TYR R 312 120.565 104.941 138.539 1.00 0.00 C**

**ATOM 111 O TYR R 312 119.884 104.687 137.532 1.00 0.00 O**

**ATOM 112 N TYR R 312 121.876 103.596 140.140 1.00 0.00 N**

**ATOM 113 CB TYR R 312 119.874 104.710 140.956 1.00 0.00 C**

**ATOM 114 CG TYR R 312 118.520 105.333 140.715 1.00 0.00 C**

**ATOM 115 CD1 TYR R 312 117.377 104.548 140.635 1.00 0.00 C**

**ATOM 116 CE1 TYR R 312 116.133 105.114 140.437 1.00 0.00 C**

**ATOM 117 CZ TYR R 312 116.018 106.485 140.342 1.00 0.00 C**

**ATOM 118 OH TYR R 312 114.781 107.050 140.150 1.00 0.00 O**

**ATOM 119 CE2 TYR R 312 117.136 107.292 140.444 1.00 0.00 C**

**ATOM 120 CD2 TYR R 312 118.376 106.713 140.634 1.00 0.00 C**

**ATOM 121 CA TYR R 313 121.480 106.873 137.431 1.00 0.00 C**

**ATOM 122 C TYR R 313 122.231 106.241 136.262 1.00 0.00 C**

**ATOM 123 O TYR R 313 122.008 106.643 135.117 1.00 0.00 O**

**ATOM 124 N TYR R 313 121.395 105.987 138.588 1.00 0.00 N**

**ATOM 125 CB TYR R 313 122.108 108.211 137.823 1.00 0.00 C**

**ATOM 126 CG TYR R 313 121.136 109.114 138.550 1.00 0.00 C**

**ATOM 127 CD1 TYR R 313 120.221 109.887 137.839 1.00 0.00 C**

**ATOM 128 CE1 TYR R 313 119.316 110.707 138.493 1.00 0.00 C**

**ATOM 129 CZ TYR R 313 119.316 110.756 139.872 1.00 0.00 C**

**ATOM 130 OH TYR R 313 118.422 111.569 140.532 1.00 0.00 O**

**ATOM 131 CE2 TYR R 313 120.209 109.995 140.598 1.00 0.00 C**

**ATOM 132 CD2 TYR R 313 121.109 109.175 139.937 1.00 0.00 C**

**ATOM 133 CA TYR R 320 118.111 106.452 127.573 1.00 0.00 C**

**ATOM 134 C TYR R 320 119.100 106.067 126.471 1.00 0.00 C**

**ATOM 135 O TYR R 320 118.962 106.533 125.331 1.00 0.00 O**

**ATOM 136 N TYR R 320 118.035 105.448 128.633 1.00 0.00 N**

**ATOM 137 CB TYR R 320 118.470 107.811 128.166 1.00 0.00 C**

**ATOM 138 CG TYR R 320 117.327 108.484 128.893 1.00 0.00 C**

**ATOM 139 CD1 TYR R 320 116.342 109.168 128.190 1.00 0.00 C**

**ATOM 140 CE1 TYR R 320 115.295 109.787 128.850 1.00 0.00 C**

**ATOM 141 CZ TYR R 320 115.221 109.721 130.223 1.00 0.00 C**

**ATOM 142 OH TYR R 320 114.180 110.333 130.882 1.00 0.00 O**

**ATOM 143 CE2 TYR R 320 116.184 109.048 130.942 1.00 0.00 C**

**ATOM 144 CD2 TYR R 320 117.230 108.436 130.277 1.00 0.00 C**

**ATOM 145 CA TYR R 330 116.530 100.688 113.037 1.00 0.00 C**

**ATOM 146 C TYR R 330 116.674 101.359 111.680 1.00 0.00 C**

**ATOM 147 O TYR R 330 116.139 100.841 110.702 1.00 0.00 O**

**ATOM 148 N TYR R 330 117.713 100.747 113.890 1.00 0.00 N**

**ATOM 149 CB TYR R 330 115.325 101.290 113.767 1.00 0.00 C**

**ATOM 150 CG TYR R 330 114.704 100.344 114.767 1.00 0.00 C**

**ATOM 151 CD1 TYR R 330 113.722 99.443 114.370 1.00 0.00 C**

**ATOM 152 CE1 TYR R 330 113.146 98.571 115.272 1.00 0.00 C**

**ATOM 153 CZ TYR R 330 113.563 98.579 116.585 1.00 0.00 C**

**ATOM 154 OH TYR R 330 112.993 97.706 117.481 1.00 0.00 O**

**ATOM 155 CE2 TYR R 330 114.544 99.458 117.006 1.00 0.00 C**

**ATOM 156 CD2 TYR R 330 115.111 100.333 116.096 1.00 0.00 C**

**ATOM 157 CA VAL R 60 132.940 113.494 135.847 1.00 0.00 C**

**ATOM 158 C VAL R 60 132.721 112.443 134.762 1.00 0.00 C**

**ATOM 159 O VAL R 60 132.238 112.758 133.667 1.00 0.00 O**

**ATOM 160 N VAL R 60 132.456 113.012 137.138 1.00 0.00 N**

**ATOM 161 CB VAL R 60 134.422 113.898 135.955 1.00 0.00 C**

**ATOM 162 CG1 VAL R 60 134.956 114.376 134.611 1.00 0.00 C**

**ATOM 163 CG2 VAL R 60 134.600 114.972 137.017 1.00 0.00 C**

**ATOM 164 CA VAL R 65 128.665 109.292 129.605 1.00 0.00 C**

**ATOM 165 C VAL R 65 127.308 109.868 129.207 1.00 0.00 C**

**ATOM 166 O VAL R 65 126.826 109.635 128.089 1.00 0.00 O**

**ATOM 167 N VAL R 65 129.488 110.315 130.256 1.00 0.00 N**

**ATOM 168 CB VAL R 65 128.523 108.052 130.510 1.00 0.00 C**

**ATOM 169 CG1 VAL R 65 127.535 107.056 129.924 1.00 0.00 C**

**ATOM 170 CG2 VAL R 65 129.862 107.355 130.647 1.00 0.00 C**

**ATOM 171 CA VAL R 68 128.018 111.266 124.955 1.00 0.00 C**

**ATOM 172 C VAL R 68 126.689 110.723 124.437 1.00 0.00 C**

**ATOM 173 O VAL R 68 126.377 110.853 123.247 1.00 0.00 O**

**ATOM 174 N VAL R 68 127.814 112.072 126.163 1.00 0.00 N**

**ATOM 175 CB VAL R 68 129.034 110.137 125.212 1.00 0.00 C**

**ATOM 176 CG1 VAL R 68 129.065 109.166 124.043 1.00 0.00 C**

**ATOM 177 CG2 VAL R 68 130.424 110.721 125.381 1.00 0.00 C**

**ATOM 178 CA VAL R 69 124.605 109.519 124.954 1.00 0.00 C**

**ATOM 179 C VAL R 69 123.680 110.616 124.422 1.00 0.00 C**

**ATOM 180 O VAL R 69 122.994 110.421 123.409 1.00 0.00 O**

**ATOM 181 N VAL R 69 125.923 110.055 125.315 1.00 0.00 N**

**ATOM 182 CB VAL R 69 123.985 108.772 126.152 1.00 0.00 C**

**ATOM 183 CG1 VAL R 69 122.601 108.252 125.813 1.00 0.00 C**

**ATOM 184 CG2 VAL R 69 124.855 107.588 126.529 1.00 0.00 C**

**ATOM 185 CA VAL R 71 124.979 114.157 121.666 1.00 0.00 C**

**ATOM 186 C VAL R 71 124.735 113.124 120.567 1.00 0.00 C**

**ATOM 187 O VAL R 71 124.207 113.459 119.498 1.00 0.00 O**

**ATOM 188 N VAL R 71 124.527 113.658 122.967 1.00 0.00 N**

**ATOM 189 CB VAL R 71 126.458 114.584 121.735 1.00 0.00 C**

**ATOM 190 CG1 VAL R 71 126.978 114.962 120.358 1.00 0.00 C**

**ATOM 191 CG2 VAL R 71 126.612 115.779 122.662 1.00 0.00 C**

**ATOM 192 CA VAL R 72 124.943 110.775 119.843 1.00 0.00 C**

**ATOM 193 C VAL R 72 123.464 110.607 119.496 1.00 0.00 C**

**ATOM 194 O VAL R 72 123.096 110.527 118.315 1.00 0.00 O**

**ATOM 195 N VAL R 72 125.150 111.868 120.803 1.00 0.00 N**

**ATOM 196 CB VAL R 72 125.557 109.467 120.379 1.00 0.00 C**

**ATOM 197 CG1 VAL R 72 125.098 108.269 119.560 1.00 0.00 C**

**ATOM 198 CG2 VAL R 72 127.075 109.547 120.332 1.00 0.00 C**

**ATOM 199 CA VAL R 75 122.622 113.817 116.090 1.00 0.00 C**

**ATOM 200 C VAL R 75 121.976 112.710 115.261 1.00 0.00 C**

**ATOM 201 O VAL R 75 121.556 112.941 114.118 1.00 0.00 O**

**ATOM 202 N VAL R 75 122.115 113.799 117.464 1.00 0.00 N**

**ATOM 203 CB VAL R 75 124.161 113.726 116.081 1.00 0.00 C**

**ATOM 204 CG1 VAL R 75 124.694 113.570 114.665 1.00 0.00 C**

**ATOM 205 CG2 VAL R 75 124.759 114.987 116.683 1.00 0.00 C**

**ATOM 206 CA VAL R 80 117.127 110.308 110.517 1.00 0.00 C**

**ATOM 207 C VAL R 80 115.874 111.117 110.191 1.00 0.00 C**

**ATOM 208 O VAL R 80 115.247 110.914 109.138 1.00 0.00 O**

**ATOM 209 N VAL R 80 118.212 111.181 110.976 1.00 0.00 N**

**ATOM 210 CB VAL R 80 116.847 109.204 111.557 1.00 0.00 C**

**ATOM 211 CG1 VAL R 80 115.621 108.390 111.171 1.00 0.00 C**

**ATOM 212 CG2 VAL R 80 118.042 108.271 111.664 1.00 0.00 C**

**ATOM 213 CA VAL R 83 116.329 112.182 105.731 1.00 0.00 C**

**ATOM 214 C VAL R 83 114.879 111.822 105.408 1.00 0.00 C**

**ATOM 215 O VAL R 83 114.470 111.843 104.239 1.00 0.00 O**

**ATOM 216 N VAL R 83 116.407 113.064 106.898 1.00 0.00 N**

**ATOM 217 CB VAL R 83 117.208 110.931 105.942 1.00 0.00 C**

**ATOM 218 CG1 VAL R 83 116.953 109.888 104.868 1.00 0.00 C**

**ATOM 219 CG2 VAL R 83 118.676 111.316 105.896 1.00 0.00 C**

**ATOM 220 CA VAL R 108 112.935 111.654 124.849 1.00 0.00 C**

**ATOM 221 C VAL R 108 114.371 112.063 125.201 1.00 0.00 C**

**ATOM 222 O VAL R 108 114.688 112.278 126.380 1.00 0.00 O**

**ATOM 223 N VAL R 108 112.489 112.293 123.612 1.00 0.00 N**

**ATOM 224 CB VAL R 108 112.754 110.124 124.723 1.00 0.00 C**

**ATOM 225 CG1 VAL R 108 113.553 109.354 125.753 1.00 0.00 C**

**ATOM 226 CG2 VAL R 108 111.292 109.764 124.914 1.00 0.00 C**

**ATOM 227 CA VAL R 118 119.074 117.198 139.062 1.00 0.00 C**

**ATOM 228 C VAL R 118 120.019 116.272 139.828 1.00 0.00 C**

**ATOM 229 O VAL R 118 120.096 116.326 141.061 1.00 0.00 O**

**ATOM 230 N VAL R 118 119.694 117.670 137.820 1.00 0.00 N**

**ATOM 231 CB VAL R 118 117.717 116.528 138.751 1.00 0.00 C**

**ATOM 232 CG1 VAL R 118 117.141 115.795 139.959 1.00 0.00 C**

**ATOM 233 CG2 VAL R 118 116.718 117.574 138.310 1.00 0.00 C**

**ATOM 234 CA VAL R 129 106.032 125.233 133.928 1.00 0.00 C**

**ATOM 235 C VAL R 129 107.003 124.288 133.228 1.00 0.00 C**

**ATOM 236 O VAL R 129 106.590 123.332 132.552 1.00 0.00 O**

**ATOM 237 N VAL R 129 106.470 125.506 135.298 1.00 0.00 N**

**ATOM 238 CB VAL R 129 105.867 126.557 133.157 1.00 0.00 C**

**ATOM 239 CG1 VAL R 129 105.512 126.305 131.697 1.00 0.00 C**

**ATOM 240 CG2 VAL R 129 104.818 127.432 133.825 1.00 0.00 C**

**ATOM 241 CA VAL R 134 109.542 118.070 130.807 1.00 0.00 C**

**ATOM 242 C VAL R 134 109.040 116.709 131.296 1.00 0.00 C**

**ATOM 243 O VAL R 134 109.287 115.681 130.646 1.00 0.00 O**

**ATOM 244 N VAL R 134 108.504 119.097 130.934 1.00 0.00 N**

**ATOM 245 CB VAL R 134 110.833 118.520 131.524 1.00 0.00 C**

**ATOM 246 CG1 VAL R 134 111.847 117.403 131.587 1.00 0.00 C**

**ATOM 247 CG2 VAL R 134 111.472 119.666 130.755 1.00 0.00 C**

**ATOM 248 CA VAL R 154 103.349 93.336 115.194 1.00 0.00 C**

**ATOM 249 C VAL R 154 103.158 93.479 113.683 1.00 0.00 C**

**ATOM 250 O VAL R 154 102.671 92.555 113.015 1.00 0.00 O**

**ATOM 251 N VAL R 154 104.606 93.956 115.621 1.00 0.00 N**

**ATOM 252 CB VAL R 154 102.167 93.934 115.987 1.00 0.00 C**

**ATOM 253 CG1 VAL R 154 100.825 93.397 115.501 1.00 0.00 C**

**ATOM 254 CG2 VAL R 154 102.313 93.614 117.456 1.00 0.00 C**

**ATOM 255 CA VAL R 160 105.634 87.529 107.470 1.00 0.00 C**

**ATOM 256 C VAL R 160 104.681 86.345 107.609 1.00 0.00 C**

**ATOM 257 O VAL R 160 104.618 85.486 106.729 1.00 0.00 O**

**ATOM 258 N VAL R 160 104.971 88.802 107.758 1.00 0.00 N**

**ATOM 259 CB VAL R 160 106.882 87.357 108.366 1.00 0.00 C**

**ATOM 260 CG1 VAL R 160 107.539 86.015 108.114 1.00 0.00 C**

**ATOM 261 CG2 VAL R 160 107.881 88.457 108.078 1.00 0.00 C**

**ATOM 262 CA VAL R 164 97.297 87.700 102.429 1.00 0.00 C**

**ATOM 263 C VAL R 164 96.657 88.873 103.166 1.00 0.00 C**

**ATOM 264 O VAL R 164 96.106 89.789 102.540 1.00 0.00 O**

**ATOM 265 N VAL R 164 98.513 87.277 103.118 1.00 0.00 N**

**ATOM 266 CB VAL R 164 96.337 86.500 102.293 1.00 0.00 C**

**ATOM 267 CG1 VAL R 164 95.051 86.884 101.565 1.00 0.00 C**

**ATOM 268 CG2 VAL R 164 97.030 85.345 101.584 1.00 0.00 C**

**ATOM 269 CA VAL R 189 99.110 109.660 130.466 1.00 0.00 C**

**ATOM 270 C VAL R 189 99.753 111.037 130.347 1.00 0.00 C**

**ATOM 271 O VAL R 189 100.215 111.604 131.345 1.00 0.00 O**

**ATOM 272 N VAL R 189 98.048 109.522 129.476 1.00 0.00 N**

**ATOM 273 CB VAL R 189 100.131 108.516 130.300 1.00 0.00 C**

**ATOM 274 CG1 VAL R 189 101.186 108.546 131.401 1.00 0.00 C**

**ATOM 275 CG2 VAL R 189 99.418 107.171 130.272 1.00 0.00 C**

**ATOM 276 CA VAL R 195 97.570 117.734 135.730 1.00 0.00 C**

**ATOM 277 C VAL R 195 98.281 117.440 137.051 1.00 0.00 C**

**ATOM 278 O VAL R 195 98.270 118.267 137.971 1.00 0.00 O**

**ATOM 279 N VAL R 195 98.407 117.370 134.590 1.00 0.00 N**

**ATOM 280 CB VAL R 195 96.206 117.023 135.632 1.00 0.00 C**

**ATOM 281 CG1 VAL R 195 95.377 117.243 136.888 1.00 0.00 C**

**ATOM 282 CG2 VAL R 195 95.436 117.539 134.427 1.00 0.00 C**

**ATOM 283 CA VAL R 201 109.685 122.912 148.148 1.00 0.00 C**

**ATOM 284 C VAL R 201 109.647 122.126 149.461 1.00 0.00 C**

**ATOM 285 O VAL R 201 108.587 121.965 150.079 1.00 0.00 O**

**ATOM 286 N VAL R 201 108.508 122.583 147.347 1.00 0.00 N**

**ATOM 287 CB VAL R 201 109.772 124.441 148.373 1.00 0.00 C**

**ATOM 288 CG1 VAL R 201 108.492 125.002 149.001 1.00 0.00 C**

**ATOM 289 CG2 VAL R 201 110.999 124.824 149.203 1.00 0.00 C**

**ATOM 290 CA VAL R 205 116.029 119.626 153.624 1.00 0.00 C**

**ATOM 291 C VAL R 205 116.447 120.913 152.920 1.00 0.00 C**

**ATOM 292 O VAL R 205 117.608 121.029 152.497 1.00 0.00 O**

**ATOM 293 N VAL R 205 114.854 119.860 154.457 1.00 0.00 N**

**ATOM 294 CB VAL R 205 115.660 118.435 152.707 1.00 0.00 C**

**ATOM 295 CG1 VAL R 205 116.711 118.123 151.633 1.00 0.00 C**

**ATOM 296 CG2 VAL R 205 115.380 117.193 153.544 1.00 0.00 C**

**ATOM 297 CA VAL R 207 115.685 121.897 148.687 1.00 0.00 C**

**ATOM 298 C VAL R 207 114.322 121.760 148.014 1.00 0.00 C**

**ATOM 299 O VAL R 207 113.338 121.380 148.644 1.00 0.00 O**

**ATOM 300 N VAL R 207 115.406 122.199 150.091 1.00 0.00 N**

**ATOM 301 CB VAL R 207 116.554 120.588 148.538 1.00 0.00 C**

**ATOM 302 CG1 VAL R 207 116.794 120.154 147.099 1.00 0.00 C**

**ATOM 303 CG2 VAL R 207 117.938 120.710 149.188 1.00 0.00 C**

**ATOM 304 CA VAL R 230 103.489 101.651 134.430 1.00 0.00 C**

**ATOM 305 C VAL R 230 104.392 100.480 134.063 1.00 0.00 C**

**ATOM 306 O VAL R 230 104.621 100.216 132.876 1.00 0.00 O**

**ATOM 307 N VAL R 230 102.497 101.249 135.428 1.00 0.00 N**

**ATOM 308 CB VAL R 230 104.295 102.868 134.922 1.00 0.00 C**

**ATOM 309 CG1 VAL R 230 105.460 103.159 133.991 1.00 0.00 C**

**ATOM 310 CG2 VAL R 230 103.406 104.098 134.962 1.00 0.00 C**

**ATOM 311 CA VAL R 236 105.385 92.955 130.504 1.00 0.00 C**

**ATOM 312 C VAL R 236 104.689 92.676 129.176 1.00 0.00 C**

**ATOM 313 O VAL R 236 105.025 91.716 128.475 1.00 0.00 O**

**ATOM 314 N VAL R 236 105.789 94.359 130.606 1.00 0.00 N**

**ATOM 315 CB VAL R 236 104.508 92.565 131.709 1.00 0.00 C**

**ATOM 316 CG1 VAL R 236 103.901 91.183 131.517 1.00 0.00 C**

**ATOM 317 CG2 VAL R 236 105.345 92.592 132.973 1.00 0.00 C**

**ATOM 318 CA VAL R 239 108.286 92.217 125.879 1.00 0.00 C**

**ATOM 319 C VAL R 239 107.686 90.901 125.395 1.00 0.00 C**

**ATOM 320 O VAL R 239 108.215 90.282 124.467 1.00 0.00 O**

**ATOM 321 N VAL R 239 107.256 93.255 125.952 1.00 0.00 N**

**ATOM 322 CB VAL R 239 109.011 92.060 127.231 1.00 0.00 C**

**ATOM 323 CG1 VAL R 239 109.759 90.735 127.312 1.00 0.00 C**

**ATOM 324 CG2 VAL R 239 109.995 93.203 127.426 1.00 0.00 C**

**ATOM 325 CA VAL R 244 105.976 85.840 120.586 1.00 0.00 C**

**ATOM 326 C VAL R 244 106.036 86.154 119.091 1.00 0.00 C**

**ATOM 327 O VAL R 244 106.157 85.244 118.258 1.00 0.00 O**

**ATOM 328 N VAL R 244 106.900 86.694 121.339 1.00 0.00 N**

**ATOM 329 CB VAL R 244 104.546 85.972 121.143 1.00 0.00 C**

**ATOM 330 CG1 VAL R 244 103.538 85.306 120.220 1.00 0.00 C**

**ATOM 331 CG2 VAL R 244 104.462 85.330 122.518 1.00 0.00 C**

**ATOM 332 CA VAL R 256 110.300 78.955 103.641 1.00 0.00 C**

**ATOM 333 C VAL R 256 111.299 78.272 102.717 1.00 0.00 C**

**ATOM 334 O VAL R 256 112.484 78.151 103.052 1.00 0.00 O**

**ATOM 335 N VAL R 256 110.373 78.319 104.953 1.00 0.00 N**

**ATOM 336 CB VAL R 256 110.574 80.467 103.741 1.00 0.00 C**

**ATOM 337 CG1 VAL R 256 110.810 81.073 102.364 1.00 0.00 C**

**ATOM 338 CG2 VAL R 256 109.421 81.166 104.442 1.00 0.00 C**

**ATOM 339 CA VAL R 276 116.353 90.216 115.454 1.00 0.00 C**

**ATOM 340 C VAL R 276 116.878 89.999 116.873 1.00 0.00 C**

**ATOM 341 O VAL R 276 116.426 90.653 117.827 1.00 0.00 O**

**ATOM 342 N VAL R 276 117.458 90.386 114.505 1.00 0.00 N**

**ATOM 343 CB VAL R 276 115.440 89.061 115.001 1.00 0.00 C**

**ATOM 344 CG1 VAL R 276 114.467 88.671 116.099 1.00 0.00 C**

**ATOM 345 CG2 VAL R 276 114.676 89.457 113.747 1.00 0.00 C**

**ATOM 346 CA VAL R 278 120.462 92.033 118.569 1.00 0.00 C**

**ATOM 347 C VAL R 278 119.431 93.028 119.092 1.00 0.00 C**

**ATOM 348 O VAL R 278 119.591 93.577 120.189 1.00 0.00 O**

**ATOM 349 N VAL R 278 119.813 90.807 118.096 1.00 0.00 N**

**ATOM 350 CB VAL R 278 121.347 92.646 117.464 1.00 0.00 C**

**ATOM 351 CG1 VAL R 278 121.750 94.069 117.813 1.00 0.00 C**

**ATOM 352 CG2 VAL R 278 122.621 91.834 117.313 1.00 0.00 C**

**ATOM 353 CA VAL R 279 117.343 94.260 118.695 1.00 0.00 C**

**ATOM 354 C VAL R 279 116.660 93.844 120.001 1.00 0.00 C**

**ATOM 355 O VAL R 279 116.499 94.660 120.927 1.00 0.00 O**

**ATOM 356 N VAL R 279 118.381 93.299 118.300 1.00 0.00 N**

**ATOM 357 CB VAL R 279 116.326 94.436 117.549 1.00 0.00 C**

**ATOM 358 CG1 VAL R 279 115.111 95.214 118.017 1.00 0.00 C**

**ATOM 359 CG2 VAL R 279 116.975 95.151 116.374 1.00 0.00 C**

**ATOM 360 CA VAL R 280 115.643 92.088 121.335 1.00 0.00 C**

**ATOM 361 C VAL R 280 116.583 92.079 122.540 1.00 0.00 C**

**ATOM 362 O VAL R 280 116.185 92.454 123.655 1.00 0.00 O**

**ATOM 363 N VAL R 280 116.340 92.552 120.136 1.00 0.00 N**

**ATOM 364 CB VAL R 280 115.014 90.704 121.074 1.00 0.00 C**

**ATOM 365 CG1 VAL R 280 114.388 90.127 122.333 1.00 0.00 C**

**ATOM 366 CG2 VAL R 280 113.955 90.809 119.989 1.00 0.00 C**

**ATOM 367 CA VAL R 282 119.362 95.515 123.411 1.00 0.00 C**

**ATOM 368 C VAL R 282 118.159 96.013 124.203 1.00 0.00 C**

**ATOM 369 O VAL R 282 118.321 96.634 125.263 1.00 0.00 O**

**ATOM 370 N VAL R 282 119.170 94.123 122.999 1.00 0.00 N**

**ATOM 371 CB VAL R 282 119.665 96.409 122.190 1.00 0.00 C**

**ATOM 372 CG1 VAL R 282 119.633 97.881 122.572 1.00 0.00 C**

**ATOM 373 CG2 VAL R 282 121.045 96.092 121.643 1.00 0.00 C**

**ATOM 374 CA VAL R 284 115.882 93.584 127.299 1.00 0.00 C**

**ATOM 375 C VAL R 284 116.980 94.072 128.243 1.00 0.00 C**

**ATOM 376 O VAL R 284 116.696 94.481 129.378 1.00 0.00 O**

**ATOM 377 N VAL R 284 115.979 94.245 125.997 1.00 0.00 N**

**ATOM 378 CB VAL R 284 115.911 92.054 127.129 1.00 0.00 C**

**ATOM 379 CG1 VAL R 284 116.043 91.365 128.477 1.00 0.00 C**

**ATOM 380 CG2 VAL R 284 114.641 91.585 126.440 1.00 0.00 C**

**ATOM 381 CA VAL R 285 119.399 94.492 128.567 1.00 0.00 C**

**ATOM 382 C VAL R 285 119.259 95.954 128.995 1.00 0.00 C**

**ATOM 383 O VAL R 285 119.698 96.332 130.089 1.00 0.00 O**

**ATOM 384 N VAL R 285 118.242 94.043 127.783 1.00 0.00 N**

**ATOM 385 CB VAL R 285 120.696 94.211 127.765 1.00 0.00 C**

**ATOM 386 CG1 VAL R 285 121.929 94.867 128.375 1.00 0.00 C**

**ATOM 387 CG2 VAL R 285 120.953 92.710 127.719 1.00 0.00 C**

**ATOM 388 CA VAL R 296 112.697 96.582 144.492 1.00 0.00 C**

**ATOM 389 C VAL R 296 112.729 97.723 145.503 1.00 0.00 C**

**ATOM 390 O VAL R 296 112.342 97.548 146.666 1.00 0.00 O**

**ATOM 391 N VAL R 296 112.106 97.028 143.226 1.00 0.00 N**

**ATOM 392 CB VAL R 296 114.095 95.976 144.259 1.00 0.00 C**

**ATOM 393 CG1 VAL R 296 114.802 95.715 145.577 1.00 0.00 C**

**ATOM 394 CG2 VAL R 296 113.972 94.656 143.519 1.00 0.00 C**

**ATOM 395 CA THR R 63 128.273 113.236 133.319 1.00 0.00 C**

**ATOM 396 C THR R 63 128.863 113.193 131.909 1.00 0.00 C**

**ATOM 397 O THR R 63 128.208 113.633 130.959 1.00 0.00 O**

**ATOM 398 N THR R 63 128.786 112.146 134.150 1.00 0.00 N**

**ATOM 399 CB THR R 63 128.488 114.605 133.990 1.00 0.00 C**

**ATOM 400 OG1 THR R 63 127.702 115.593 133.311 1.00 0.00 O**

**ATOM 401 CG2 THR R 63 129.939 115.058 133.965 1.00 0.00 C**

**ATOM 402 CA THR R 88 112.774 109.935 101.099 1.00 0.00 C**

**ATOM 403 C THR R 88 111.347 110.328 101.446 1.00 0.00 C**

**ATOM 404 O THR R 88 111.038 110.581 102.614 1.00 0.00 O**

**ATOM 405 N THR R 88 113.686 111.010 101.475 1.00 0.00 N**

**ATOM 406 CB THR R 88 113.147 108.629 101.806 1.00 0.00 C**

**ATOM 407 OG1 THR R 88 114.458 108.221 101.399 1.00 0.00 O**

**ATOM 408 CG2 THR R 88 112.151 107.533 101.460 1.00 0.00 C**

**ATOM 409 CA THR R 92 105.557 103.342 102.563 1.00 0.00 C**

**ATOM 410 C THR R 92 105.158 103.515 104.026 1.00 0.00 C**

**ATOM 411 O THR R 92 105.228 104.608 104.591 1.00 0.00 O**

**ATOM 412 N THR R 92 105.702 104.643 101.932 1.00 0.00 N**

**ATOM 413 CB THR R 92 106.858 102.539 102.455 1.00 0.00 C**

**ATOM 414 OG1 THR R 92 107.919 103.260 103.094 1.00 0.00 O**

**ATOM 415 CG2 THR R 92 107.220 102.305 100.996 1.00 0.00 C**

**ATOM 416 CA THR R 94 107.817 101.457 107.326 1.00 0.00 C**

**ATOM 417 C THR R 94 108.487 102.800 107.621 1.00 0.00 C**

**ATOM 418 O THR R 94 108.897 103.050 108.759 1.00 0.00 O**

**ATOM 419 N THR R 94 106.590 101.616 106.547 1.00 0.00 N**

**ATOM 420 CB THR R 94 108.761 100.456 106.632 1.00 0.00 C**

**ATOM 421 OG1 THR R 94 109.977 100.321 107.382 1.00 0.00 O**

**ATOM 422 CG2 THR R 94 109.038 100.775 105.148 1.00 0.00 C**

**ATOM 423 CA THR R 109 116.630 112.629 124.433 1.00 0.00 C**

**ATOM 424 C THR R 109 116.767 114.051 124.995 1.00 0.00 C**

**ATOM 425 O THR R 109 117.740 114.341 125.704 1.00 0.00 O**

**ATOM 426 N THR R 109 115.234 112.260 124.194 1.00 0.00 N**

**ATOM 427 CB THR R 109 117.397 112.449 123.107 1.00 0.00 C**

**ATOM 428 OG1 THR R 109 117.260 111.091 122.672 1.00 0.00 O**

**ATOM 429 CG2 THR R 109 118.896 112.729 123.234 1.00 0.00 C**

**ATOM 430 CA THR R 110 115.746 116.286 125.256 1.00 0.00 C**

**ATOM 431 C THR R 110 115.776 116.356 126.787 1.00 0.00 C**

**ATOM 432 O THR R 110 116.366 117.282 127.359 1.00 0.00 O**

**ATOM 433 N THR R 110 115.766 114.909 124.756 1.00 0.00 N**

**ATOM 434 CB THR R 110 114.502 116.990 124.691 1.00 0.00 C**

**ATOM 435 OG1 THR R 110 114.538 116.930 123.260 1.00 0.00 O**

**ATOM 436 CG2 THR R 110 114.428 118.461 125.100 1.00 0.00 C**

**ATOM 437 CA THR R 111 115.030 115.394 128.915 1.00 0.00 C**

**ATOM 438 C THR R 111 116.360 115.205 129.659 1.00 0.00 C**

**ATOM 439 O THR R 111 116.521 115.723 130.773 1.00 0.00 O**

**ATOM 440 N THR R 111 115.194 115.361 127.462 1.00 0.00 N**

**ATOM 441 CB THR R 111 114.000 114.314 129.292 1.00 0.00 C**

**ATOM 442 OG1 THR R 111 112.763 114.594 128.624 1.00 0.00 O**

**ATOM 443 CG2 THR R 111 113.710 114.254 130.787 1.00 0.00 C**

**ATOM 444 CA THR R 117 121.379 118.815 136.465 1.00 0.00 C**

**ATOM 445 C THR R 117 120.805 118.417 137.824 1.00 0.00 C**

**ATOM 446 O THR R 117 121.342 118.799 138.870 1.00 0.00 O**

**ATOM 447 N THR R 117 121.401 117.681 135.541 1.00 0.00 N**

**ATOM 448 CB THR R 117 120.579 119.965 135.846 1.00 0.00 C**

**ATOM 449 OG1 THR R 117 121.173 120.341 134.598 1.00 0.00 O**

**ATOM 450 CG2 THR R 117 120.563 121.181 136.765 1.00 0.00 C**

**ATOM 451 CA THR R 144 104.956 106.516 122.483 1.00 0.00 C**

**ATOM 452 C THR R 144 106.053 105.733 121.765 1.00 0.00 C**

**ATOM 453 O THR R 144 105.775 104.998 120.810 1.00 0.00 O**

**ATOM 454 N THR R 144 105.127 106.464 123.933 1.00 0.00 N**

**ATOM 455 CB THR R 144 104.941 107.975 122.021 1.00 0.00 C**

**ATOM 456 OG1 THR R 144 103.892 108.678 122.697 1.00 0.00 O**

**ATOM 457 CG2 THR R 144 104.710 108.075 120.521 1.00 0.00 C**

**ATOM 458 CA THR R 148 105.808 102.338 118.342 1.00 0.00 C**

**ATOM 459 C THR R 148 106.667 101.122 118.015 1.00 0.00 C**

**ATOM 460 O THR R 148 106.466 100.482 116.974 1.00 0.00 O**

**ATOM 461 N THR R 148 105.391 102.326 119.741 1.00 0.00 N**

**ATOM 462 CB THR R 148 106.561 103.637 118.038 1.00 0.00 C**

**ATOM 463 OG1 THR R 148 105.711 104.757 118.319 1.00 0.00 O**

**ATOM 464 CG2 THR R 148 106.984 103.697 116.577 1.00 0.00 C**

**ATOM 465 CA THR R 150 105.690 97.095 119.638 1.00 0.00 C**

**ATOM 466 C THR R 150 104.806 96.959 118.403 1.00 0.00 C**

**ATOM 467 O THR R 150 104.643 95.855 117.868 1.00 0.00 O**

**ATOM 468 N THR R 150 106.527 98.287 119.554 1.00 0.00 N**

**ATOM 469 CB THR R 150 104.842 97.155 120.911 1.00 0.00 C**

**ATOM 470 OG1 THR R 150 105.703 97.291 122.047 1.00 0.00 O**

**ATOM 471 CG2 THR R 150 104.008 95.895 121.076 1.00 0.00 C**

**ATOM 472 CA THR R 171 98.328 100.560 103.756 1.00 0.00 C**

**ATOM 473 C THR R 171 98.943 101.761 104.473 1.00 0.00 C**

**ATOM 474 O THR R 171 99.078 101.742 105.702 1.00 0.00 O**

**ATOM 475 N THR R 171 98.728 99.355 104.458 1.00 0.00 N**

**ATOM 476 CB THR R 171 96.794 100.662 103.751 1.00 0.00 C**

**ATOM 477 OG1 THR R 171 96.243 99.474 103.170 1.00 0.00 O**

**ATOM 478 CG2 THR R 171 96.273 101.867 102.971 1.00 0.00 C**

**ATOM 479 CA THR R 199 107.031 121.515 141.861 1.00 0.00 C**

**ATOM 480 C THR R 199 106.641 121.746 143.323 1.00 0.00 C**

**ATOM 481 O THR R 199 105.660 122.442 143.601 1.00 0.00 O**

**ATOM 482 N THR R 199 105.856 121.203 141.061 1.00 0.00 N**

**ATOM 483 CB THR R 199 107.702 122.758 141.265 1.00 0.00 C**

**ATOM 484 OG1 THR R 199 108.031 122.501 139.893 1.00 0.00 O**

**ATOM 485 CG2 THR R 199 108.958 123.094 141.976 1.00 0.00 C**

**ATOM 486 CA THR R 247 109.772 83.792 117.662 1.00 0.00 C**

**ATOM 487 C THR R 247 108.954 83.083 116.589 1.00 0.00 C**

**ATOM 488 O THR R 247 109.485 82.247 115.843 1.00 0.00 O**

**ATOM 489 N THR R 247 109.579 85.239 117.604 1.00 0.00 N**

**ATOM 490 CB THR R 247 109.407 83.272 119.053 1.00 0.00 C**

**ATOM 491 OG1 THR R 247 110.222 83.925 120.035 1.00 0.00 O**

**ATOM 492 CG2 THR R 247 109.635 81.771 119.138 1.00 0.00 C**

**ATOM 493 CA THR R 273 118.092 86.287 112.660 1.00 0.00 C**

**ATOM 494 C THR R 273 118.982 87.008 113.668 1.00 0.00 C**

**ATOM 495 O THR R 273 118.539 87.331 114.779 1.00 0.00 O**

**ATOM 496 N THR R 273 118.229 86.874 111.329 1.00 0.00 N**

**ATOM 497 CB THR R 273 118.427 84.793 112.602 1.00 0.00 C**

**ATOM 498 OG1 THR R 273 117.511 84.133 111.721 1.00 0.00 O**

**ATOM 499 CG2 THR R 273 118.331 84.155 113.981 1.00 0.00 C**

**ATOM 500 CA THR R 288 115.083 95.177 132.563 1.00 0.00 C**

**ATOM 501 C THR R 288 115.752 95.657 133.871 1.00 0.00 C**

**ATOM 502 O THR R 288 115.073 95.594 134.917 1.00 0.00 O**

**ATOM 503 N THR R 288 115.215 96.040 131.385 1.00 0.00 N**

**ATOM 504 CB THR R 288 115.573 93.776 132.146 1.00 0.00 C**

**ATOM 505 OG1 THR R 288 114.842 93.345 130.991 1.00 0.00 O**

**ATOM 506 CG2 THR R 288 115.374 92.767 133.242 1.00 0.00 C**

**ATOM 507 CA THR R 302 114.235 101.419 151.043 1.00 0.00 C**

**ATOM 508 C THR R 302 115.608 101.725 151.628 1.00 0.00 C**

**ATOM 509 O THR R 302 115.860 101.449 152.806 1.00 0.00 O**

**ATOM 510 N THR R 302 113.917 100.015 151.268 1.00 0.00 N**

**ATOM 511 CB THR R 302 113.177 102.329 151.672 1.00 0.00 C**

**ATOM 512 OG1 THR R 302 111.884 101.988 151.158 1.00 0.00 O**

**ATOM 513 CG2 THR R 302 113.470 103.790 151.358 1.00 0.00 C**

**ATOM 514 CA THR R 306 126.213 106.617 147.151 1.00 0.00 C**

**ATOM 515 C THR R 306 126.365 105.730 145.915 1.00 0.00 C**

**ATOM 516 O THR R 306 126.004 106.129 144.799 1.00 0.00 O**

**ATOM 517 N THR R 306 125.455 105.961 148.220 1.00 0.00 N**

**ATOM 518 CB THR R 306 127.586 107.043 147.676 1.00 0.00 C**

**ATOM 519 OG1 THR R 306 128.325 105.886 148.088 1.00 0.00 O**

**ATOM 520 CG2 THR R 306 127.436 107.991 148.857 1.00 0.00 C**

**ATOM 521 CA THR R 321 121.067 104.775 125.805 1.00 0.00 C**

**ATOM 522 C THR R 321 120.391 103.910 124.749 1.00 0.00 C**

**ATOM 523 O THR R 321 120.783 103.936 123.578 1.00 0.00 O**

**ATOM 524 N THR R 321 120.095 105.234 126.792 1.00 0.00 N**

**ATOM 525 CB THR R 321 122.205 104.022 126.509 1.00 0.00 C**

**ATOM 526 OG1 THR R 321 122.806 104.878 127.487 1.00 0.00 O**

**ATOM 527 CG2 THR R 321 123.299 103.585 125.535 1.00 0.00 C**

**ATOM 528 CA GLY R 73 121.170 110.434 120.262 1.00 0.00 C**

**ATOM 529 C GLY R 73 120.549 111.601 119.518 1.00 0.00 C**

**ATOM 530 O GLY R 73 119.721 111.401 118.623 1.00 0.00 O**

**ATOM 531 N GLY R 73 122.593 110.604 120.513 1.00 0.00 N**

**ATOM 532 CA GLY R 76 121.216 110.375 115.187 1.00 0.00 C**

**ATOM 533 C GLY R 76 119.770 110.644 114.814 1.00 0.00 C**

**ATOM 534 O GLY R 76 119.397 110.505 113.639 1.00 0.00 O**

**ATOM 535 N GLY R 76 121.896 111.494 115.817 1.00 0.00 N**

**ATOM 536 CA GLY R 127 110.110 127.204 137.215 1.00 0.00 C**

**ATOM 537 C GLY R 127 109.291 125.927 137.192 1.00 0.00 C**

**ATOM 538 O GLY R 127 109.770 124.837 136.877 1.00 0.00 O**

**ATOM 539 N GLY R 127 111.510 126.994 136.918 1.00 0.00 N**

**ATOM 540 CA GLY R 190 100.274 112.962 128.958 1.00 0.00 C**

**ATOM 541 C GLY R 190 99.457 114.000 129.711 1.00 0.00 C**

**ATOM 542 O GLY R 190 100.009 114.850 130.415 1.00 0.00 O**

**ATOM 543 N GLY R 190 99.774 111.602 129.135 1.00 0.00 N**

**ATOM 544 CA GLY R 197 103.096 117.071 137.586 1.00 0.00 C**

**ATOM 545 C GLY R 197 103.179 118.464 138.190 1.00 0.00 C**

**ATOM 546 O GLY R 197 102.323 119.319 137.950 1.00 0.00 O**

**ATOM 547 N GLY R 197 101.724 116.592 137.490 1.00 0.00 N**

**ATOM 548 CA GLY R 198 104.411 119.994 139.591 1.00 0.00 C**

**ATOM 549 C GLY R 198 105.657 120.037 140.451 1.00 0.00 C**

**ATOM 550 O GLY R 198 106.435 119.076 140.526 1.00 0.00 O**

**ATOM 551 N GLY R 198 104.223 118.690 138.981 1.00 0.00 N**

**ATOM 552 CA GLY R 261 117.106 77.027 98.812 1.00 0.00 C**

**ATOM 553 C GLY R 261 118.600 77.003 98.543 1.00 0.00 C**

**ATOM 554 O GLY R 261 119.106 76.084 97.894 1.00 0.00 O**

**ATOM 555 N GLY R 261 116.756 77.457 100.158 1.00 0.00 N**

**ATOM 556 CA GLY R 300 111.205 95.325 148.819 1.00 0.00 C**

**ATOM 557 C GLY R 300 111.970 95.978 149.958 1.00 0.00 C**

**ATOM 558 O GLY R 300 111.885 95.536 151.107 1.00 0.00 O**

**ATOM 559 N GLY R 300 110.382 96.242 148.034 1.00 0.00 N**

**ATOM 560 CA GLY R 319 117.478 103.244 129.499 1.00 0.00 C**

**ATOM 561 C GLY R 319 117.384 104.304 128.418 1.00 0.00 C**

**ATOM 562 O GLY R 319 116.729 104.103 127.392 1.00 0.00 O**

**ATOM 563 N GLY R 319 118.816 103.125 130.063 1.00 0.00 N**

**ATOM 564 CA PHE R 70 122.872 112.923 124.610 1.00 0.00 C**

**ATOM 565 C PHE R 70 123.235 113.395 123.205 1.00 0.00 C**

**ATOM 566 O PHE R 70 122.356 113.533 122.343 1.00 0.00 O**

**ATOM 567 N PHE R 70 123.715 111.806 125.041 1.00 0.00 N**

**ATOM 568 CB PHE R 70 122.987 114.079 125.607 1.00 0.00 C**

**ATOM 569 CG PHE R 70 122.249 115.321 125.191 1.00 0.00 C**

**ATOM 570 CD1 PHE R 70 120.863 115.372 125.254 1.00 0.00 C**

**ATOM 571 CE1 PHE R 70 120.178 116.515 124.877 1.00 0.00 C**

**ATOM 572 CZ PHE R 70 120.879 117.625 124.438 1.00 0.00 C**

**ATOM 573 CE2 PHE R 70 122.261 117.589 124.377 1.00 0.00 C**

**ATOM 574 CD2 PHE R 70 122.939 116.443 124.755 1.00 0.00 C**

**ATOM 575 CA PHE R 82 115.978 115.124 108.155 1.00 0.00 C**

**ATOM 576 C PHE R 82 115.873 114.295 106.876 1.00 0.00 C**

**ATOM 577 O PHE R 82 115.324 114.766 105.868 1.00 0.00 O**

**ATOM 578 N PHE R 82 115.719 114.318 109.347 1.00 0.00 N**

**ATOM 579 CB PHE R 82 117.355 115.778 108.264 1.00 0.00 C**

**ATOM 580 CG PHE R 82 117.655 116.752 107.162 1.00 0.00 C**

**ATOM 581 CD1 PHE R 82 117.007 117.977 107.110 1.00 0.00 C**

**ATOM 582 CE1 PHE R 82 117.282 118.881 106.098 1.00 0.00 C**

**ATOM 583 CZ PHE R 82 118.216 118.565 105.127 1.00 0.00 C**

**ATOM 584 CE2 PHE R 82 118.873 117.347 105.170 1.00 0.00 C**

**ATOM 585 CD2 PHE R 82 118.592 116.448 106.185 1.00 0.00 C**

**ATOM 586 CA PHE R 99 109.579 108.718 111.405 1.00 0.00 C**

**ATOM 587 C PHE R 99 109.028 108.979 112.803 1.00 0.00 C**

**ATOM 588 O PHE R 99 109.562 109.812 113.548 1.00 0.00 O**

**ATOM 589 N PHE R 99 109.753 107.287 111.158 1.00 0.00 N**

**ATOM 590 CB PHE R 99 108.652 109.305 110.336 1.00 0.00 C**

**ATOM 591 CG PHE R 99 108.378 110.778 110.489 1.00 0.00 C**

**ATOM 592 CD1 PHE R 99 109.290 111.718 110.038 1.00 0.00 C**

**ATOM 593 CE1 PHE R 99 109.031 113.069 110.159 1.00 0.00 C**

**ATOM 594 CZ PHE R 99 107.848 113.497 110.733 1.00 0.00 C**

**ATOM 595 CE2 PHE R 99 106.923 112.572 111.178 1.00 0.00 C**

**ATOM 596 CD2 PHE R 99 107.185 111.221 111.047 1.00 0.00 C**

**ATOM 597 CA PHE R 114 117.738 118.227 133.101 1.00 0.00 C**

**ATOM 598 C PHE R 114 117.937 117.196 134.205 1.00 0.00 C**

**ATOM 599 O PHE R 114 118.128 117.558 135.371 1.00 0.00 O**

**ATOM 600 N PHE R 114 118.464 117.849 131.892 1.00 0.00 N**

**ATOM 601 CB PHE R 114 116.255 118.407 132.787 1.00 0.00 C**

**ATOM 602 CG PHE R 114 115.972 119.529 131.834 1.00 0.00 C**

**ATOM 603 CD1 PHE R 114 115.976 120.844 132.275 1.00 0.00 C**

**ATOM 604 CE1 PHE R 114 115.718 121.884 131.402 1.00 0.00 C**

**ATOM 605 CZ PHE R 114 115.447 121.616 130.072 1.00 0.00 C**

**ATOM 606 CE2 PHE R 114 115.437 120.308 129.619 1.00 0.00 C**

**ATOM 607 CD2 PHE R 114 115.698 119.273 130.499 1.00 0.00 C**

**ATOM 608 CA PHE R 126 113.824 126.422 137.381 1.00 0.00 C**

**ATOM 609 C PHE R 126 112.382 126.610 137.850 1.00 0.00 C**

**ATOM 610 O PHE R 126 112.051 126.417 139.023 1.00 0.00 O**

**ATOM 611 N PHE R 126 114.639 125.874 138.452 1.00 0.00 N**

**ATOM 612 CB PHE R 126 113.907 125.526 136.144 1.00 0.00 C**

**ATOM 613 CG PHE R 126 115.309 125.243 135.695 1.00 0.00 C**

**ATOM 614 CD1 PHE R 126 116.089 126.252 135.150 1.00 0.00 C**

**ATOM 615 CE1 PHE R 126 117.383 126.000 134.731 1.00 0.00 C**

**ATOM 616 CZ PHE R 126 117.909 124.727 134.853 1.00 0.00 C**

**ATOM 617 CE2 PHE R 126 117.141 123.710 135.394 1.00 0.00 C**

**ATOM 618 CD2 PHE R 126 115.847 123.970 135.811 1.00 0.00 C**

**ATOM 619 CA PHE R 143 105.273 105.346 126.106 1.00 0.00 C**

**ATOM 620 C PHE R 143 105.026 105.308 124.601 1.00 0.00 C**

**ATOM 621 O PHE R 143 104.754 104.236 124.043 1.00 0.00 O**

**ATOM 622 N PHE R 143 106.325 106.302 126.451 1.00 0.00 N**

**ATOM 623 CB PHE R 143 103.978 105.669 126.852 1.00 0.00 C**

**ATOM 624 CG PHE R 143 104.034 105.380 128.325 1.00 0.00 C**

**ATOM 625 CD1 PHE R 143 104.951 104.473 128.836 1.00 0.00 C**

**ATOM 626 CE1 PHE R 143 104.994 104.199 130.190 1.00 0.00 C**

**ATOM 627 CZ PHE R 143 104.113 104.824 131.047 1.00 0.00 C**

**ATOM 628 CE2 PHE R 143 103.191 105.719 130.552 1.00 0.00 C**

**ATOM 629 CD2 PHE R 143 103.150 105.994 129.197 1.00 0.00 C**

**ATOM 630 CA PHE R 147 104.270 101.350 121.695 1.00 0.00 C**

**ATOM 631 C PHE R 147 104.556 101.384 120.197 1.00 0.00 C**

**ATOM 632 O PHE R 147 104.040 100.539 119.456 1.00 0.00 O**

**ATOM 633 N PHE R 147 105.402 101.842 122.476 1.00 0.00 N**

**ATOM 634 CB PHE R 147 103.011 102.152 122.023 1.00 0.00 C**

**ATOM 635 CG PHE R 147 102.509 101.950 123.427 1.00 0.00 C**

**ATOM 636 CD1 PHE R 147 102.753 100.762 124.103 1.00 0.00 C**

**ATOM 637 CE1 PHE R 147 102.292 100.573 125.394 1.00 0.00 C**

**ATOM 638 CZ PHE R 147 101.578 101.577 126.024 1.00 0.00 C**

**ATOM 639 CE2 PHE R 147 101.327 102.766 125.362 1.00 0.00 C**

**ATOM 640 CD2 PHE R 147 101.791 102.947 124.069 1.00 0.00 C**

**ATOM 641 CA PHE R 169 97.060 95.787 106.464 1.00 0.00 C**

**ATOM 642 C PHE R 169 98.192 96.793 106.278 1.00 0.00 C**

**ATOM 643 O PHE R 169 98.332 97.725 107.077 1.00 0.00 O**

**ATOM 644 N PHE R 169 96.611 95.248 105.186 1.00 0.00 N**

**ATOM 645 CB PHE R 169 97.493 94.647 107.394 1.00 0.00 C**

**ATOM 646 CG PHE R 169 97.918 95.101 108.765 1.00 0.00 C**

**ATOM 647 CD1 PHE R 169 96.970 95.347 109.747 1.00 0.00 C**

**ATOM 648 CE1 PHE R 169 97.349 95.759 111.013 1.00 0.00 C**

**ATOM 649 CZ PHE R 169 98.691 95.915 111.314 1.00 0.00 C**

**ATOM 650 CE2 PHE R 169 99.650 95.661 110.348 1.00 0.00 C**

**ATOM 651 CD2 PHE R 169 99.263 95.250 109.085 1.00 0.00 C**

**ATOM 652 CA PHE R 214 99.664 112.717 144.947 1.00 0.00 C**

**ATOM 653 C PHE R 214 99.279 113.157 146.356 1.00 0.00 C**

**ATOM 654 O PHE R 214 100.098 113.773 147.050 1.00 0.00 O**

**ATOM 655 N PHE R 214 100.800 113.536 144.561 1.00 0.00 N**

**ATOM 656 CB PHE R 214 100.022 111.219 144.933 1.00 0.00 C**

**ATOM 657 CG PHE R 214 100.384 110.676 143.579 1.00 0.00 C**

**ATOM 658 CD1 PHE R 214 99.900 111.260 142.418 1.00 0.00 C**

**ATOM 659 CE1 PHE R 214 100.238 110.756 141.180 1.00 0.00 C**

**ATOM 660 CZ PHE R 214 101.070 109.658 141.092 1.00 0.00 C**

**ATOM 661 CE2 PHE R 214 101.561 109.064 142.238 1.00 0.00 C**

**ATOM 662 CD2 PHE R 214 101.218 109.574 143.473 1.00 0.00 C**

**ATOM 663 CA PHE R 225 99.962 101.444 142.299 1.00 0.00 C**

**ATOM 664 C PHE R 225 100.329 101.968 140.913 1.00 0.00 C**

**ATOM 665 O PHE R 225 100.263 101.223 139.924 1.00 0.00 O**

**ATOM 666 N PHE R 225 100.959 101.815 143.304 1.00 0.00 N**

**ATOM 667 CB PHE R 225 98.583 101.952 142.728 1.00 0.00 C**

**ATOM 668 CG PHE R 225 97.477 101.615 141.763 1.00 0.00 C**

**ATOM 669 CD1 PHE R 225 96.893 100.356 141.773 1.00 0.00 C**

**ATOM 670 CE1 PHE R 225 95.870 100.042 140.893 1.00 0.00 C**

**ATOM 671 CZ PHE R 225 95.410 100.996 140.002 1.00 0.00 C**

**ATOM 672 CE2 PHE R 225 95.974 102.259 139.990 1.00 0.00 C**

**ATOM 673 CD2 PHE R 225 96.996 102.566 140.872 1.00 0.00 C**

**ATOM 674 CA PHE R 231 105.799 98.626 134.814 1.00 0.00 C**

**ATOM 675 C PHE R 231 105.113 97.534 134.001 1.00 0.00 C**

**ATOM 676 O PHE R 231 105.672 97.068 133.003 1.00 0.00 O**

**ATOM 677 N PHE R 231 104.902 99.753 135.072 1.00 0.00 N**

**ATOM 678 CB PHE R 231 106.350 98.057 136.129 1.00 0.00 C**

**ATOM 679 CG PHE R 231 107.032 96.710 135.977 1.00 0.00 C**

**ATOM 680 CD1 PHE R 231 108.355 96.640 135.565 1.00 0.00 C**

**ATOM 681 CE1 PHE R 231 108.994 95.416 135.426 1.00 0.00 C**

**ATOM 682 CZ PHE R 231 108.319 94.245 135.730 1.00 0.00 C**

**ATOM 683 CE2 PHE R 231 107.005 94.299 136.165 1.00 0.00 C**

**ATOM 684 CD2 PHE R 231 106.373 95.524 136.304 1.00 0.00 C**

**ATOM 685 CA PHE R 233 101.786 98.062 130.727 1.00 0.00 C**

**ATOM 686 C PHE R 233 102.950 98.398 129.800 1.00 0.00 C**

**ATOM 687 O PHE R 233 102.879 98.124 128.596 1.00 0.00 O**

**ATOM 688 N PHE R 233 102.231 97.651 132.059 1.00 0.00 N**

**ATOM 689 CB PHE R 233 100.811 99.237 130.828 1.00 0.00 C**

**ATOM 690 CG PHE R 233 99.427 98.838 131.267 1.00 0.00 C**

**ATOM 691 CD1 PHE R 233 98.707 97.899 130.541 1.00 0.00 C**

**ATOM 692 CE1 PHE R 233 97.432 97.525 130.931 1.00 0.00 C**

**ATOM 693 CZ PHE R 233 96.858 98.098 132.051 1.00 0.00 C**

**ATOM 694 CE2 PHE R 233 97.562 99.042 132.781 1.00 0.00 C**

**ATOM 695 CD2 PHE R 233 98.837 99.411 132.385 1.00 0.00 C**

**ATOM 696 CA PHE R 235 107.252 96.270 130.085 1.00 0.00 C**

**ATOM 697 C PHE R 235 106.738 94.867 129.806 1.00 0.00 C**

**ATOM 698 O PHE R 235 107.219 94.232 128.865 1.00 0.00 O**

**ATOM 699 N PHE R 235 106.197 97.268 130.163 1.00 0.00 N**

**ATOM 700 CB PHE R 235 108.076 96.289 131.385 1.00 0.00 C**

**ATOM 701 CG PHE R 235 109.269 95.372 131.367 1.00 0.00 C**

**ATOM 702 CD1 PHE R 235 110.436 95.758 130.721 1.00 0.00 C**

**ATOM 703 CE1 PHE R 235 111.542 94.929 130.702 1.00 0.00 C**

**ATOM 704 CZ PHE R 235 111.496 93.703 131.341 1.00 0.00 C**

**ATOM 705 CE2 PHE R 235 110.342 93.309 131.997 1.00 0.00 C**

**ATOM 706 CD2 PHE R 235 109.238 94.145 132.012 1.00 0.00 C**

**ATOM 707 CA PHE R 283 115.736 96.134 124.452 1.00 0.00 C**

**ATOM 708 C PHE R 283 115.634 95.530 125.855 1.00 0.00 C**

**ATOM 709 O PHE R 283 115.345 96.248 126.825 1.00 0.00 O**

**ATOM 710 N PHE R 283 116.939 95.680 123.749 1.00 0.00 N**

**ATOM 711 CB PHE R 283 114.497 95.786 123.624 1.00 0.00 C**

**ATOM 712 CG PHE R 283 113.217 96.384 124.146 1.00 0.00 C**

**ATOM 713 CD1 PHE R 283 112.868 97.688 123.827 1.00 0.00 C**

**ATOM 714 CE1 PHE R 283 111.687 98.242 124.295 1.00 0.00 C**

**ATOM 715 CZ PHE R 283 110.840 97.489 125.088 1.00 0.00 C**

**ATOM 716 CE2 PHE R 283 111.172 96.185 125.410 1.00 0.00 C**

**ATOM 717 CD2 PHE R 283 112.352 95.636 124.935 1.00 0.00 C**

**ATOM 718 CA PHE R 293 115.626 98.331 140.535 1.00 0.00 C**

**ATOM 719 C PHE R 293 114.563 99.234 141.162 1.00 0.00 C**

**ATOM 720 O PHE R 293 114.461 99.330 142.396 1.00 0.00 O**

**ATOM 721 N PHE R 293 115.066 97.488 139.478 1.00 0.00 N**

**ATOM 722 CB PHE R 293 116.780 99.150 139.953 1.00 0.00 C**

**ATOM 723 CG PHE R 293 117.706 99.721 140.981 1.00 0.00 C**

**ATOM 724 CD1 PHE R 293 118.583 98.899 141.672 1.00 0.00 C**

**ATOM 725 CE1 PHE R 293 119.449 99.423 142.613 1.00 0.00 C**

**ATOM 726 CZ PHE R 293 119.454 100.780 142.861 1.00 0.00 C**

**ATOM 727 CE2 PHE R 293 118.592 101.607 142.168 1.00 0.00 C**

**ATOM 728 CD2 PHE R 293 117.727 101.080 141.233 1.00 0.00 C**

**ATOM 729 CA PHE R 314 123.650 104.467 135.408 1.00 0.00 C**

**ATOM 730 C PHE R 314 122.614 103.617 134.679 1.00 0.00 C**

**ATOM 731 O PHE R 314 122.649 103.508 133.449 1.00 0.00 O**

**ATOM 732 N PHE R 314 123.042 105.208 136.508 1.00 0.00 N**

**ATOM 733 CB PHE R 314 124.800 103.608 135.938 1.00 0.00 C**

**ATOM 734 CG PHE R 314 125.506 102.807 134.880 1.00 0.00 C**

**ATOM 735 CD1 PHE R 314 126.435 103.413 134.046 1.00 0.00 C**

**ATOM 736 CE1 PHE R 314 127.100 102.685 133.075 1.00 0.00 C**

**ATOM 737 CZ PHE R 314 126.852 101.331 132.939 1.00 0.00 C**

**ATOM 738 CE2 PHE R 314 125.939 100.709 133.773 1.00 0.00 C**

**ATOM 739 CD2 PHE R 314 125.274 101.445 134.741 1.00 0.00 C**

**ATOM 740 CA PHE R 332 121.172 102.493 109.541 1.00 0.00 C**

**ATOM 741 C PHE R 332 121.448 100.995 109.386 1.00 0.00 C**

**ATOM 742 O PHE R 332 122.609 100.614 109.194 1.00 0.00 O**

**ATOM 743 N PHE R 332 119.892 102.788 110.175 1.00 0.00 N**

**ATOM 744 CB PHE R 332 122.307 103.153 110.329 1.00 0.00 C**

**ATOM 745 CG PHE R 332 122.274 104.653 110.294 1.00 0.00 C**

**ATOM 746 CD1 PHE R 332 122.740 105.346 109.186 1.00 0.00 C**

**ATOM 747 CE1 PHE R 332 122.708 106.730 109.150 1.00 0.00 C**

**ATOM 748 CZ PHE R 332 122.204 107.435 110.230 1.00 0.00 C**

**ATOM 749 CE2 PHE R 332 121.735 106.756 111.341 1.00 0.00 C**

**ATOM 750 CD2 PHE R 332 121.771 105.372 111.368 1.00 0.00 C**

**ATOM 751 CA PHE R 337 117.259 102.929 103.946 1.00 0.00 C**

**ATOM 752 C PHE R 337 118.674 103.175 103.445 1.00 0.00 C**

**ATOM 753 O PHE R 337 119.049 104.325 103.197 1.00 0.00 O**

**ATOM 754 N PHE R 337 116.791 101.610 103.537 1.00 0.00 N**

**ATOM 755 CB PHE R 337 117.206 103.062 105.469 1.00 0.00 C**

**ATOM 756 CG PHE R 337 117.492 104.454 105.976 1.00 0.00 C**

**ATOM 757 CD1 PHE R 337 116.485 105.405 106.000 1.00 0.00 C**

**ATOM 758 CE1 PHE R 337 116.728 106.682 106.467 1.00 0.00 C**

**ATOM 759 CZ PHE R 337 117.996 107.031 106.900 1.00 0.00 C**

**ATOM 760 CE2 PHE R 337 119.014 106.097 106.873 1.00 0.00 C**

**ATOM 761 CD2 PHE R 337 118.762 104.818 106.411 1.00 0.00 C**

**ATOM 762 CA PHE R 341 120.883 107.015 101.542 1.00 0.00 C**

**ATOM 763 C PHE R 341 121.967 107.119 100.476 1.00 0.00 C**

**ATOM 764 O PHE R 341 122.501 108.208 100.231 1.00 0.00 O**

**ATOM 765 N PHE R 341 119.797 106.138 101.114 1.00 0.00 N**

**ATOM 766 CB PHE R 341 121.466 106.502 102.860 1.00 0.00 C**

**ATOM 767 CG PHE R 341 122.553 107.369 103.430 1.00 0.00 C**

**ATOM 768 CD1 PHE R 341 122.247 108.577 104.040 1.00 0.00 C**

**ATOM 769 CE1 PHE R 341 123.247 109.376 104.571 1.00 0.00 C**

**ATOM 770 CZ PHE R 341 124.567 108.966 104.501 1.00 0.00 C**

**ATOM 771 CE2 PHE R 341 124.885 107.760 103.900 1.00 0.00 C**

**ATOM 772 CD2 PHE R 341 123.880 106.967 103.371 1.00 0.00 C**

**ATOM 773 CA MET R 81 114.351 112.921 110.823 1.00 0.00 C**

**ATOM 774 C MET R 81 114.519 113.766 109.567 1.00 0.00 C**

**ATOM 775 O MET R 81 113.578 113.894 108.780 1.00 0.00 O**

**ATOM 776 N MET R 81 115.518 112.075 111.062 1.00 0.00 N**

**ATOM 777 CB MET R 81 114.094 113.812 112.040 1.00 0.00 C**

**ATOM 778 CG MET R 81 113.359 113.127 113.185 1.00 0.00 C**

**ATOM 779 SD MET R 81 111.632 112.785 112.825 1.00 0.00 S**

**ATOM 780 CE MET R 81 110.992 114.455 112.821 1.00 0.00 C**

**ATOM 781 CA MET R 90 106.723 108.936 103.109 1.00 0.00 C**

**ATOM 782 C MET R 90 105.580 108.277 102.334 1.00 0.00 C**

**ATOM 783 O MET R 90 104.398 108.570 102.520 1.00 0.00 O**

**ATOM 784 N MET R 90 107.434 109.897 102.262 1.00 0.00 N**

**ATOM 785 CB MET R 90 106.208 109.617 104.370 1.00 0.00 C**

**ATOM 786 CG MET R 90 107.295 110.176 105.269 1.00 0.00 C**

**ATOM 787 SD MET R 90 106.639 110.794 106.829 1.00 0.00 S**

**ATOM 788 CE MET R 90 105.533 112.075 106.244 1.00 0.00 C**

**ATOM 789 CA MET R 112 118.594 114.109 129.644 1.00 0.00 C**

**ATOM 790 C MET R 112 119.418 115.178 130.383 1.00 0.00 C**

**ATOM 791 O MET R 112 119.860 114.885 131.509 1.00 0.00 O**

**ATOM 792 N MET R 112 117.336 114.544 129.021 1.00 0.00 N**

**ATOM 793 CB MET R 112 119.452 113.407 128.579 1.00 0.00 C**

**ATOM 794 CG MET R 112 120.574 112.567 129.145 1.00 0.00 C**

**ATOM 795 SD MET R 112 121.464 111.695 127.849 1.00 0.00 S**

**ATOM 796 CE MET R 112 120.127 110.847 127.026 1.00 0.00 C**

**ATOM 797 CA MET R 121 123.181 120.074 141.617 1.00 0.00 C**

**ATOM 798 C MET R 121 122.408 119.667 142.864 1.00 0.00 C**

**ATOM 799 O MET R 121 122.507 120.352 143.892 1.00 0.00 O**

**ATOM 800 N MET R 121 123.492 118.934 140.758 1.00 0.00 N**

**ATOM 801 CB MET R 121 122.396 121.123 140.830 1.00 0.00 C**

**ATOM 802 CG MET R 121 123.156 121.718 139.655 1.00 0.00 C**

**ATOM 803 SD MET R 121 124.643 122.601 140.164 1.00 0.00 S**

**ATOM 804 CE MET R 121 123.927 124.024 140.983 1.00 0.00 C**

**ATOM 805 CA MET R 142 108.593 107.204 126.329 1.00 0.00 C**

**ATOM 806 C MET R 142 107.584 106.098 126.035 1.00 0.00 C**

**ATOM 807 O MET R 142 107.924 105.075 125.417 1.00 0.00 O**

**ATOM 808 N MET R 142 108.035 108.515 126.006 1.00 0.00 N**

**ATOM 809 CB MET R 142 109.040 107.168 127.791 1.00 0.00 C**

**ATOM 810 CG MET R 142 109.645 105.843 128.225 1.00 0.00 C**

**ATOM 811 SD MET R 142 109.897 105.739 130.008 1.00 0.00 S**

**ATOM 812 CE MET R 142 108.208 105.786 130.592 1.00 0.00 C**

**ATOM 813 CA MET R 151 103.494 98.042 116.670 1.00 0.00 C**

**ATOM 814 C MET R 151 104.356 97.711 115.457 1.00 0.00 C**

**ATOM 815 O MET R 151 103.856 97.084 114.520 1.00 0.00 O**

**ATOM 816 N MET R 151 104.266 98.078 117.908 1.00 0.00 N**

**ATOM 817 CB MET R 151 102.731 99.347 116.478 1.00 0.00 C**

**ATOM 818 CG MET R 151 101.452 99.409 117.313 1.00 0.00 C**

**ATOM 819 SD MET R 151 100.290 98.056 117.006 1.00 0.00 S**

**ATOM 820 CE MET R 151 100.425 97.093 118.515 1.00 0.00 C**

**ATOM 821 CA MET R 152 106.506 97.714 114.339 1.00 0.00 C**

**ATOM 822 C MET R 152 106.757 96.211 114.378 1.00 0.00 C**

**ATOM 823 O MET R 152 106.801 95.556 113.324 1.00 0.00 O**

**ATOM 824 N MET R 152 105.628 98.131 115.435 1.00 0.00 N**

**ATOM 825 CB MET R 152 107.836 98.467 114.380 1.00 0.00 C**

**ATOM 826 CG MET R 152 107.740 99.939 114.057 1.00 0.00 C**

**ATOM 827 SD MET R 152 109.321 100.792 114.207 1.00 0.00 S**

**ATOM 828 CE MET R 152 110.184 100.157 112.773 1.00 0.00 C**

**ATOM 829 CA MET R 226 101.117 103.824 139.541 1.00 0.00 C**

**ATOM 830 C MET R 226 102.371 103.130 139.016 1.00 0.00 C**

**ATOM 831 O MET R 226 102.475 102.867 137.813 1.00 0.00 O**

**ATOM 832 N MET R 226 100.682 103.261 140.819 1.00 0.00 N**

**ATOM 833 CB MET R 226 101.354 105.332 139.658 1.00 0.00 C**

**ATOM 834 CG MET R 226 101.772 105.964 138.328 1.00 0.00 C**

**ATOM 835 SD MET R 226 101.883 107.755 138.262 1.00 0.00 S**

**ATOM 836 CE MET R 226 102.537 107.976 136.610 1.00 0.00 C**

**ATOM 837 CA MET R 249 108.415 84.996 112.677 1.00 0.00 C**

**ATOM 838 C MET R 249 109.584 84.138 112.209 1.00 0.00 C**

**ATOM 839 O MET R 249 109.629 83.717 111.048 1.00 0.00 O**

**ATOM 840 N MET R 249 107.878 84.513 113.946 1.00 0.00 N**

**ATOM 841 CB MET R 249 108.856 86.452 112.822 1.00 0.00 C**

**ATOM 842 CG MET R 249 109.316 87.104 111.531 1.00 0.00 C**

**ATOM 843 SD MET R 249 110.071 88.710 111.842 1.00 0.00 S**

**ATOM 844 CE MET R 249 108.663 89.629 112.451 1.00 0.00 C**

**ATOM 845 CA GLU R 203 111.644 122.981 154.182 1.00 0.00 C**

**ATOM 846 C GLU R 203 112.802 122.412 155.000 1.00 0.00 C**

**ATOM 847 O GLU R 203 113.819 123.090 155.184 1.00 0.00 O**

**ATOM 848 N GLU R 203 111.100 122.035 153.207 1.00 0.00 N**

**ATOM 849 CB GLU R 203 110.530 123.451 155.117 1.00 0.00 C**

**ATOM 850 CG GLU R 203 109.432 124.247 154.438 1.00 0.00 C**

**ATOM 851 CD GLU R 203 108.414 124.784 155.424 1.00 0.00 C**

**ATOM 852 OE1 GLU R 203 108.544 124.492 156.632 1.00 0.00 O**

**ATOM 853 OE2 GLU R 203 107.483 125.496 154.992 1.00 0.00 O**

**ATOM 854 CA GLU R 209 111.859 118.865 144.125 1.00 0.00 C**

**ATOM 855 C GLU R 209 110.752 119.027 143.084 1.00 0.00 C**

**ATOM 856 O GLU R 209 109.718 119.657 143.335 1.00 0.00 O**

**ATOM 857 N GLU R 209 111.978 120.026 145.001 1.00 0.00 N**

**ATOM 858 CB GLU R 209 111.651 117.582 144.944 1.00 0.00 C**

**ATOM 859 CG GLU R 209 110.344 117.480 145.699 1.00 0.00 C**

**ATOM 860 CD GLU R 209 110.202 116.164 146.439 1.00 0.00 C**

**ATOM 861 OE1 GLU R 209 111.117 115.320 146.336 1.00 0.00 O**

**ATOM 862 OE2 GLU R 209 109.177 115.973 147.126 1.00 0.00 O**

**ATOM 863 CA GLU R 264 123.852 82.498 100.310 1.00 0.00 C**

**ATOM 864 C GLU R 264 122.553 83.054 100.888 1.00 0.00 C**

**ATOM 865 O GLU R 264 122.573 83.978 101.714 1.00 0.00 O**

**ATOM 866 N GLU R 264 123.817 81.039 100.194 1.00 0.00 N**

**ATOM 867 CB GLU R 264 124.142 83.122 98.944 1.00 0.00 C**

**ATOM 868 CG GLU R 264 125.515 82.783 98.375 1.00 0.00 C**

**ATOM 869 CD GLU R 264 126.661 83.247 99.259 1.00 0.00 C**

**ATOM 870 OE1 GLU R 264 126.563 84.347 99.844 1.00 0.00 O**

**ATOM 871 OE2 GLU R 264 127.663 82.510 99.366 1.00 0.00 O**

**ATOM 872 CA GLU R 297 113.185 100.057 145.981 1.00 0.00 C**

**ATOM 873 C GLU R 297 111.764 100.480 146.352 1.00 0.00 C**

**ATOM 874 O GLU R 297 111.530 100.962 147.467 1.00 0.00 O**

**ATOM 875 N GLU R 297 113.201 98.903 145.082 1.00 0.00 N**

**ATOM 876 CB GLU R 297 113.975 101.220 145.392 1.00 0.00 C**

**ATOM 877 CG GLU R 297 115.467 100.964 145.417 1.00 0.00 C**

**ATOM 878 CD GLU R 297 116.271 102.189 145.068 1.00 0.00 C**

**ATOM 879 OE1 GLU R 297 115.663 103.252 144.823 1.00 0.00 O**

**ATOM 880 OE2 GLU R 297 117.515 102.108 145.093 1.00 0.00 O**

**ATOM 881 CA GLU R 335 118.502 97.417 103.438 1.00 0.00 C**

**ATOM 882 C GLU R 335 117.869 98.396 102.451 1.00 0.00 C**

**ATOM 883 O GLU R 335 118.448 98.637 101.387 1.00 0.00 O**

**ATOM 884 N GLU R 335 117.934 97.504 104.781 1.00 0.00 N**

**ATOM 885 CB GLU R 335 118.371 95.988 102.909 1.00 0.00 C**

**ATOM 886 CG GLU R 335 119.232 94.975 103.649 1.00 0.00 C**

**ATOM 887 CD GLU R 335 120.716 95.189 103.422 1.00 0.00 C**

**ATOM 888 OE1 GLU R 335 121.096 95.616 102.311 1.00 0.00 O**

**ATOM 889 OE2 GLU R 335 121.502 94.935 104.358 1.00 0.00 O**

**ATOM 890 CA ARG R 86 113.439 115.495 102.544 1.00 0.00 C**

**ATOM 891 C ARG R 86 113.926 114.409 101.594 1.00 0.00 C**

**ATOM 892 O ARG R 86 113.441 114.344 100.456 1.00 0.00 O**

**ATOM 893 N ARG R 86 113.121 115.028 103.891 1.00 0.00 N**

**ATOM 894 CB ARG R 86 114.485 116.622 102.618 1.00 0.00 C**

**ATOM 895 CG ARG R 86 114.861 117.204 101.262 1.00 0.00 C**

**ATOM 896 CD ARG R 86 113.709 117.998 100.664 1.00 0.00 C**

**ATOM 897 NE ARG R 86 114.061 118.598 99.379 1.00 0.00 N**

**ATOM 898 CZ ARG R 86 113.238 119.349 98.654 1.00 0.00 C**

**ATOM 899 NH2 ARG R 86 113.643 119.851 97.496 1.00 0.00 N**

**ATOM 900 NH1 ARG R 86 112.010 119.599 99.088 1.00 0.00 N**

**ATOM 901 CA ARG R 156 106.424 92.774 110.606 1.00 0.00 C**

**ATOM 902 C ARG R 156 105.990 91.328 110.845 1.00 0.00 C**

**ATOM 903 O ARG R 156 106.151 90.480 109.953 1.00 0.00 O**

**ATOM 904 N ARG R 156 105.566 93.722 111.315 1.00 0.00 N**

**ATOM 905 CB ARG R 156 107.885 92.991 111.003 1.00 0.00 C**

**ATOM 906 CG ARG R 156 108.474 94.270 110.420 1.00 0.00 C**

**ATOM 907 CD ARG R 156 109.979 94.371 110.623 1.00 0.00 C**

**ATOM 908 NE ARG R 156 110.326 94.659 112.012 1.00 0.00 N**

**ATOM 909 CZ ARG R 156 111.540 95.009 112.422 1.00 0.00 C**

**ATOM 910 NH2 ARG R 156 111.760 95.254 113.706 1.00 0.00 N**

**ATOM 911 NH1 ARG R 156 112.532 95.124 111.549 1.00 0.00 N**

**ATOM 912 CA ARG R 170 100.149 97.510 105.008 1.00 0.00 C**

**ATOM 913 C ARG R 170 99.773 98.675 104.085 1.00 0.00 C**

**ATOM 914 O ARG R 170 100.353 98.919 103.025 1.00 0.00 O**

**ATOM 915 N ARG R 170 99.001 96.628 105.234 1.00 0.00 N**

**ATOM 916 CB ARG R 170 101.341 96.707 104.487 1.00 0.00 C**

**ATOM 917 CG ARG R 170 102.694 97.445 104.519 1.00 0.00 C**

**ATOM 918 CD ARG R 170 103.859 96.550 104.155 1.00 0.00 C**

**ATOM 919 NE ARG R 170 104.254 95.724 105.290 1.00 0.00 N**

**ATOM 920 CZ ARG R 170 105.122 96.116 106.216 1.00 0.00 C**

**ATOM 921 NH2 ARG R 170 105.432 95.313 107.223 1.00 0.00 N**

**ATOM 922 NH1 ARG R 170 105.683 97.315 106.135 1.00 0.00 N**

**ATOM 923 CA ARG R 202 110.965 120.830 151.101 1.00 0.00 C**

**ATOM 924 C ARG R 202 111.751 121.695 152.084 1.00 0.00 C**

**ATOM 925 O ARG R 202 112.915 122.043 151.823 1.00 0.00 O**

**ATOM 926 N ARG R 202 110.798 121.579 149.862 1.00 0.00 N**

**ATOM 927 CB ARG R 202 111.679 119.507 150.826 1.00 0.00 C**

**ATOM 928 CG ARG R 202 111.748 118.582 152.010 1.00 0.00 C**

**ATOM 929 CD ARG R 202 110.401 117.949 152.321 1.00 0.00 C**

**ATOM 930 NE ARG R 202 110.481 117.017 153.443 1.00 0.00 N**

**ATOM 931 CZ ARG R 202 110.852 115.744 153.343 1.00 0.00 C**

**ATOM 932 NH2 ARG R 202 111.181 115.233 152.164 1.00 0.00 N**

**ATOM 933 NH1 ARG R 202 110.894 114.979 154.425 1.00 0.00 N**

**ATOM 934 CA ARG R 252 107.754 80.713 109.952 1.00 0.00 C**

**ATOM 935 C ARG R 252 108.799 80.798 108.841 1.00 0.00 C**

**ATOM 936 O ARG R 252 108.678 80.113 107.817 1.00 0.00 O**

**ATOM 937 N ARG R 252 108.381 80.687 111.270 1.00 0.00 N**

**ATOM 938 CB ARG R 252 106.768 81.879 109.863 1.00 0.00 C**

**ATOM 939 CG ARG R 252 106.093 82.018 108.508 1.00 0.00 C**

**ATOM 940 CD ARG R 252 105.243 80.786 108.228 1.00 0.00 C**

**ATOM 941 NE ARG R 252 104.500 80.875 106.974 1.00 0.00 N**

**ATOM 942 CZ ARG R 252 104.956 80.437 105.805 1.00 0.00 C**

**ATOM 943 NH2 ARG R 252 104.211 80.552 104.715 1.00 0.00 N**

**ATOM 944 NH1 ARG R 252 106.155 79.876 105.728 1.00 0.00 N**

**ATOM 945 CA ARG R 257 111.639 77.074 100.607 1.00 0.00 C**

**ATOM 946 C ARG R 257 112.719 77.978 100.014 1.00 0.00 C**

**ATOM 947 O ARG R 257 113.828 77.514 99.723 1.00 0.00 O**

**ATOM 948 N ARG R 257 110.805 77.798 101.566 1.00 0.00 N**

**ATOM 949 CB ARG R 257 110.744 76.452 99.525 1.00 0.00 C**

**ATOM 950 CG ARG R 257 111.400 75.439 98.563 1.00 0.00 C**

**ATOM 951 CD ARG R 257 112.004 76.045 97.293 1.00 0.00 C**

**ATOM 952 NE ARG R 257 112.598 75.039 96.411 1.00 0.00 N**

**ATOM 953 CZ ARG R 257 111.915 74.228 95.606 1.00 0.00 C**

**ATOM 954 NH2 ARG R 257 112.563 73.353 94.849 1.00 0.00 N**

**ATOM 955 NH1 ARG R 257 110.590 74.282 95.551 1.00 0.00 N**

**ATOM 956 CA ARG R 263 123.699 78.746 101.053 1.00 0.00 C**

**ATOM 957 C ARG R 263 123.743 80.257 101.277 1.00 0.00 C**

**ATOM 958 O ARG R 263 123.738 80.717 102.425 1.00 0.00 O**

**ATOM 959 N ARG R 263 122.823 78.382 99.942 1.00 0.00 N**

**ATOM 960 CB ARG R 263 125.102 78.183 100.805 1.00 0.00 C**

**ATOM 961 CG ARG R 263 126.177 78.666 101.770 1.00 0.00 C**

**ATOM 962 CD ARG R 263 125.929 78.120 103.170 1.00 0.00 C**

**ATOM 963 NE ARG R 263 126.955 78.536 104.123 1.00 0.00 N**

**ATOM 964 CZ ARG R 263 128.096 77.886 104.323 1.00 0.00 C**

**ATOM 965 NH2 ARG R 263 128.362 76.781 103.640 1.00 0.00 N**

**ATOM 966 NH1 ARG R 263 128.971 78.337 105.212 1.00 0.00 N**

**ATOM 967 CA ARG R 267 123.401 83.392 105.115 1.00 0.00 C**

**ATOM 968 C ARG R 267 122.600 84.670 105.355 1.00 0.00 C**

**ATOM 969 O ARG R 267 122.723 85.303 106.412 1.00 0.00 O**

**ATOM 970 N ARG R 267 122.584 82.361 104.473 1.00 0.00 N**

**ATOM 971 CB ARG R 267 124.639 83.678 104.261 1.00 0.00 C**

**ATOM 972 CG ARG R 267 125.555 84.764 104.806 1.00 0.00 C**

**ATOM 973 CD ARG R 267 126.774 84.950 103.915 1.00 0.00 C**

**ATOM 974 NE ARG R 267 127.669 85.988 104.419 1.00 0.00 N**

**ATOM 975 CZ ARG R 267 128.816 86.331 103.840 1.00 0.00 C**

**ATOM 976 NH2 ARG R 267 129.566 87.289 104.368 1.00 0.00 N**

**ATOM 977 NH1 ARG R 267 129.213 85.717 102.734 1.00 0.00 N**

**ATOM 978 CA ARG R 270 121.144 84.095 109.408 1.00 0.00 C**

**ATOM 979 C ARG R 270 121.388 85.541 109.830 1.00 0.00 C**

**ATOM 980 O ARG R 270 121.353 85.857 111.023 1.00 0.00 O**

**ATOM 981 N ARG R 270 120.320 84.020 108.203 1.00 0.00 N**

**ATOM 982 CB ARG R 270 122.471 83.373 109.168 1.00 0.00 C**

**ATOM 983 CG ARG R 270 123.425 83.376 110.350 1.00 0.00 C**

**ATOM 984 CD ARG R 270 122.872 82.557 111.502 1.00 0.00 C**

**ATOM 985 NE ARG R 270 122.689 81.156 111.131 1.00 0.00 N**

**ATOM 986 CZ ARG R 270 123.636 80.227 111.216 1.00 0.00 C**

**ATOM 987 NH2 ARG R 270 124.844 80.544 111.661 1.00 0.00 N**

**ATOM 988 NH1 ARG R 270 123.374 78.978 110.855 1.00 0.00 N**

**ATOM 989 CA ARG R 271 121.833 87.847 109.148 1.00 0.00 C**

**ATOM 990 C ARG R 271 120.561 88.479 109.709 1.00 0.00 C**

**ATOM 991 O ARG R 271 120.622 89.281 110.651 1.00 0.00 O**

**ATOM 992 N ARG R 271 121.643 86.425 108.859 1.00 0.00 N**

**ATOM 993 CB ARG R 271 122.287 88.580 107.885 1.00 0.00 C**

**ATOM 994 CG ARG R 271 122.553 90.064 108.080 1.00 0.00 C**

**ATOM 995 CD ARG R 271 122.922 90.743 106.769 1.00 0.00 C**

**ATOM 996 NE ARG R 271 121.825 90.712 105.807 1.00 0.00 N**

**ATOM 997 CZ ARG R 271 121.897 91.207 104.575 1.00 0.00 C**

**ATOM 998 NH2 ARG R 271 120.848 91.136 103.767 1.00 0.00 N**

**ATOM 999 NH1 ARG R 271 123.018 91.775 104.152 1.00 0.00 N**

**ATOM 1000 CA ARG R 274 121.203 87.980 114.114 1.00 0.00 C**

**ATOM 1001 C ARG R 274 120.664 89.371 114.428 1.00 0.00 C**

**ATOM 1002 O ARG R 274 120.781 89.840 115.563 1.00 0.00 O**

**ATOM 1003 N ARG R 274 120.263 87.196 113.313 1.00 0.00 N**

**ATOM 1004 CB ARG R 274 122.547 88.103 113.391 1.00 0.00 C**

**ATOM 1005 CG ARG R 274 123.397 86.842 113.357 1.00 0.00 C**

**ATOM 1006 CD ARG R 274 123.885 86.429 114.732 1.00 0.00 C**

**ATOM 1007 NE ARG R 274 124.647 85.185 114.671 1.00 0.00 N**

**ATOM 1008 CZ ARG R 274 124.139 83.981 114.914 1.00 0.00 C**

**ATOM 1009 NH2 ARG R 274 124.911 82.906 114.832 1.00 0.00 N**

**ATOM 1010 NH1 ARG R 274 122.860 83.851 115.238 1.00 0.00 N**

**ATOM 1011 CA ARG R 339 119.697 102.377 99.104 1.00 0.00 C**

**ATOM 1012 C ARG R 339 119.030 103.732 98.913 1.00 0.00 C**

**ATOM 1013 O ARG R 339 119.243 104.371 97.878 1.00 0.00 O**

**ATOM 1014 N ARG R 339 119.845 102.022 100.512 1.00 0.00 N**

**ATOM 1015 CB ARG R 339 118.891 101.307 98.366 1.00 0.00 C**

**ATOM 1016 CG ARG R 339 119.554 99.944 98.299 1.00 0.00 C**

**ATOM 1017 CD ARG R 339 118.657 98.949 97.579 1.00 0.00 C**

**ATOM 1018 NE ARG R 339 119.239 97.612 97.528 1.00 0.00 N**

**ATOM 1019 CZ ARG R 339 118.630 96.556 96.999 1.00 0.00 C**

**ATOM 1020 NH2 ARG R 339 119.232 95.374 96.994 1.00 0.00 N**

**ATOM 1021 NH1 ARG R 339 117.420 96.681 96.471 1.00 0.00 N**

**ATOM 1022 CA ARG R 342 123.334 105.992 98.803 1.00 0.00 C**

**ATOM 1023 C ARG R 342 122.823 106.615 97.508 1.00 0.00 C**

**ATOM 1024 O ARG R 342 123.193 107.736 97.159 1.00 0.00 O**

**ATOM 1025 N ARG R 342 122.306 106.004 99.838 1.00 0.00 N**

**ATOM 1026 CB ARG R 342 123.819 104.563 98.545 1.00 0.00 C**

**ATOM 1027 CG ARG R 342 124.563 103.938 99.716 1.00 0.00 C**

**ATOM 1028 CD ARG R 342 125.045 102.536 99.382 1.00 0.00 C**

**ATOM 1029 NE ARG R 342 123.936 101.606 99.192 1.00 0.00 N**

**ATOM 1030 CZ ARG R 342 124.073 100.357 98.761 1.00 0.00 C**

**ATOM 1031 NH2 ARG R 342 123.007 99.581 98.618 1.00 0.00 N**

**ATOM 1032 NH1 ARG R 342 125.277 99.881 98.474 1.00 0.00 N**

**ATOM 1033 C N.A X -1 109.531 104.094 133.585 1.00 0.00 C**

**ATOM 1034 O N.A X -1 108.980 102.892 133.896 1.00 0.00 O**

**ATOM 1035 C N.A X -1 110.839 104.104 133.126 1.00 0.00 C**

**ATOM 1036 C N.A X -1 111.438 105.323 132.828 1.00 0.00 C**

**ATOM 1037 C N.A X -1 110.708 106.521 132.956 1.00 0.00 C**

**ATOM 1038 C N.A X -1 109.402 106.494 133.458 1.00 0.00 C**

**ATOM 1039 C N.A X -1 108.808 105.273 133.758 1.00 0.00 C**

**ATOM 1040 C N.A X -1 111.300 107.809 132.458 1.00 0.00 C**

**ATOM 1041 C N.A X -1 111.936 108.634 133.559 1.00 0.00 C**

**ATOM 1042 N N.A X -1 112.616 109.778 132.908 1.00 0.00 N**

**ATOM 1043 C N.A X -1 112.976 107.802 134.324 1.00 0.00 C**

**ATOM 1044 O N.A X -1 114.128 107.704 133.892 1.00 0.00 O**

**ATOM 1045 N N.A X -1 112.644 107.198 135.501 1.00 0.00 N**

**ATOM 1046 C N.A X -1 111.362 107.135 136.205 1.00 0.00 C**

**ATOM 1047 C N.A X -1 110.852 105.703 136.286 1.00 0.00 C**

**ATOM 1048 O N.A X -1 111.567 104.783 135.887 1.00 0.00 O**

**ATOM 1049 N N.A X -1 109.601 105.486 136.833 1.00 0.00 N**

**ATOM 1050 C N.A X -1 108.618 106.479 137.247 1.00 0.00 C**

**ATOM 1051 C N.A X -1 108.835 106.767 138.727 1.00 0.00 C**

**ATOM 1052 O N.A X -1 108.628 105.907 139.578 1.00 0.00 O**

**ATOM 1053 N N.A X -1 109.373 108.002 139.003 1.00 0.00 N**

**ATOM 1054 C N.A X -1 109.992 108.345 140.290 1.00 0.00 C**

**ATOM 1055 C N.A X -1 111.390 107.706 140.182 1.00 0.00 C**

**ATOM 1056 O N.A X -1 111.618 106.553 140.553 1.00 0.00 O**

**ATOM 1057 N N.A X -1 112.363 108.382 139.473 1.00 0.00 N**

**ATOM 1058 C N.A X -1 112.452 109.826 139.247 1.00 0.00 C**

**ATOM 1059 C N.A X -1 111.354 110.408 138.337 1.00 0.00 C**

**ATOM 1060 O N.A X -1 110.674 109.727 137.565 1.00 0.00 O**

**ATOM 1061 N N.A X -1 111.097 111.744 138.397 1.00 0.00 N**

**ATOM 1062 C N.A X -1 111.569 112.741 139.361 1.00 0.00 C**

**ATOM 1063 C N.A X -1 110.585 112.687 140.540 1.00 0.00 C**

**ATOM 1064 O N.A X -1 109.376 112.521 140.353 1.00 0.00 O**

**ATOM 1065 N N.A X -1 111.045 112.775 141.824 1.00 0.00 N**

**ATOM 1066 C N.A X -1 112.352 113.126 142.425 1.00 0.00 C**

**ATOM 1067 C N.A X -1 112.178 112.898 143.939 1.00 0.00 C**

**ATOM 1068 O N.A X -1 111.069 113.049 144.452 1.00 0.00 O**

**ATOM 1069 N N.A X -1 113.335 112.635 144.657 1.00 0.00 N**

**ATOM 1070 C N.A X -1 113.346 112.742 146.118 1.00 0.00 C**

**ATOM 1071 C N.A X -1 114.675 113.252 146.740 1.00 0.00 C**

**ATOM 1072 O N.A X -1 115.016 114.440 146.636 1.00 0.00 O**

**ATOM 1073 N N.A X -1 115.469 112.321 147.374 1.00 0.00 N**

**ATOM 1074 C N.A X -1 116.540 112.674 148.313 1.00 0.00 C**

**ATOM 1075 C N.A X -1 117.368 111.426 148.664 1.00 0.00 C**

**ATOM 1076 O N.A X -1 116.934 110.611 149.497 1.00 0.00 O**

**ATOM 1077 N N.A X -1 118.627 111.265 148.116 1.00 0.00 N**

**ATOM 1078 C N.A X -1 119.368 112.159 147.202 1.00 0.00 C**

**ATOM 1079 C N.A X -1 119.005 111.953 145.717 1.00 0.00 C**

**ATOM 1080 O N.A X -1 119.364 112.733 144.833 1.00 0.00 O**

**ATOM 1081 N N.A X -1 118.289 110.801 145.486 1.00 0.00 N**

**ATOM 1082 C N.A X -1 117.476 110.553 144.297 1.00 0.00 C**

**ATOM 1083 C N.A X -1 115.989 110.815 144.598 1.00 0.00 C**

**ATOM 1084 O N.A X -1 115.325 111.422 143.700 1.00 0.00 O**

**ATOM 1085 O N.A X -1 115.628 110.512 145.780 1.00 0.00 O**

**ATOM 1086 C N.A X -1 117.620 109.140 143.721 1.00 0.00 C**

**ATOM 1087 C N.A X -1 117.812 107.986 144.722 1.00 0.00 C**

**ATOM 1088 C N.A X -1 116.544 107.202 145.046 1.00 0.00 C**

**ATOM 1089 C N.A X -1 116.122 106.336 143.865 1.00 0.00 C**

**ATOM 1090 N N.A X -1 114.997 105.504 144.189 1.00 0.00 N**

**ATOM 1091 C N.A X -1 113.700 105.933 144.022 1.00 0.00 C**

**ATOM 1092 O N.A X -1 113.390 107.016 143.526 1.00 0.00 O**

**ATOM 1093 C N.A X -1 112.661 104.966 144.551 1.00 0.00 C**

**ATOM 1094 O N.A X -1 112.396 105.334 145.898 1.00 0.00 O**

**ATOM 1095 C N.A X -1 111.592 104.383 146.584 1.00 0.00 C**

**ATOM 1096 C N.A X -1 111.661 104.685 148.075 1.00 0.00 C**

**ATOM 1097 O N.A X -1 110.905 103.761 148.852 1.00 0.00 O**

**ATOM 1098 C N.A X -1 109.494 103.977 148.776 1.00 0.00 C**

**ATOM 1099 C N.A X -1 108.913 104.073 150.182 1.00 0.00 C**

**ATOM 1100 N N.A X -1 109.455 105.183 150.937 1.00 0.00 N**

**ATOM 1101 N N.A X -1 109.539 106.415 150.402 1.00 0.00 N**

**ATOM 1102 N N.A X -1 110.154 107.191 151.285 1.00 0.00 N**

**ATOM 1103 C N.A X -1 110.440 106.432 152.392 1.00 0.00 C**

**ATOM 1104 C N.A X -1 109.984 105.148 152.192 1.00 0.00 C**

**ATOM 1105 C N.A X -1 111.150 107.009 153.549 1.00 0.00 C**

**ATOM 1106 N N.A X -1 112.390 107.613 153.104 1.00 0.00 N**

**ATOM 1107 C N.A X -1 113.115 108.446 153.921 1.00 0.00 C**

**ATOM 1108 O N.A X -1 112.763 108.785 155.047 1.00 0.00 O**

**ATOM 1109 C N.A X -1 114.410 108.929 153.309 1.00 0.00 C**

**ATOM 1110 C N.A X -1 114.236 110.132 152.383 1.00 0.00 C**

**ATOM 1111 C N.A X -1 113.790 111.455 153.025 1.00 0.00 C**

**ATOM 1112 C N.A X -1 114.378 111.744 154.409 1.00 0.00 C**

**ATOM 1113 C N.A X -1 114.078 113.176 154.872 1.00 0.00 C**

**ATOM 1114 N N.A X -1 114.373 113.331 156.316 1.00 0.00 N**

**ATOM 1115 C N.A X -1 115.717 113.350 156.763 1.00 0.00 C**

**ATOM 1116 C N.A X -1 115.754 113.403 158.154 1.00 0.00 C**

**ATOM 1117 C N.A X -1 116.959 113.327 158.833 1.00 0.00 C**

**ATOM 1118 C N.A X -1 118.124 113.232 158.064 1.00 0.00 C**

**ATOM 1119 S N.A X -1 119.727 112.983 158.859 1.00 0.00 S**

**ATOM 1120 O N.A X -1 119.364 112.430 160.159 1.00 0.00 O**

**ATOM 1121 O N.A X -1 120.373 112.047 157.941 1.00 0.00 O**

**ATOM 1122 O N.A X -1 120.261 114.340 158.863 1.00 0.00 O**

**ATOM 1123 C N.A X -1 118.091 113.247 156.660 1.00 0.00 C**

**ATOM 1124 C N.A X -1 116.871 113.309 155.994 1.00 0.00 C**

**ATOM 1125 C N.A X -1 114.347 113.536 158.663 1.00 0.00 C**

**ATOM 1126 C N.A X -1 113.564 113.476 157.329 1.00 0.00 C**

**ATOM 1127 C N.A X -1 112.090 113.618 157.287 1.00 0.00 C**

**ATOM 1128 C N.A X -1 111.276 112.641 156.857 1.00 0.00 C**

**ATOM 1129 C N.A X -1 109.836 112.823 156.753 1.00 0.00 C**

**ATOM 1130 C N.A X -1 108.923 112.045 156.125 1.00 0.00 C**

**ATOM 1131 N N.A X -1 107.546 112.323 156.015 1.00 0.00 N**

**ATOM 1132 C N.A X -1 106.925 111.430 155.128 1.00 0.00 C**

**ATOM 1133 C N.A X -1 107.843 110.466 154.682 1.00 0.00 C**

**ATOM 1134 C N.A X -1 107.489 109.493 153.753 1.00 0.00 C**

**ATOM 1135 C N.A X -1 106.184 109.504 153.256 1.00 0.00 C**

**ATOM 1136 S N.A X -1 105.765 108.314 151.961 1.00 0.00 S**

**ATOM 1137 O N.A X -1 106.483 108.873 150.818 1.00 0.00 O**

**ATOM 1138 O N.A X -1 106.290 107.060 152.492 1.00 0.00 O**

**ATOM 1139 O N.A X -1 104.308 108.372 151.862 1.00 0.00 O**

**ATOM 1140 C N.A X -1 105.243 110.440 153.707 1.00 0.00 C**

**ATOM 1141 C N.A X -1 105.618 111.410 154.651 1.00 0.00 C**

**ATOM 1142 C N.A X -1 109.197 110.725 155.332 1.00 0.00 C**

**ATOM 1143 C N.A X -1 110.239 110.899 154.224 1.00 0.00 C**

**ATOM 1144 C N.A X -1 109.529 109.575 156.292 1.00 0.00 C**

**ATOM 1145 C N.A X -1 106.792 113.432 156.563 1.00 0.00 C**

**ATOM 1146 C N.A X -1 113.959 112.367 159.570 1.00 0.00 C**

**ATOM 1147 C N.A X -1 114.139 114.881 159.363 1.00 0.00 C**

**ATOM 1148 C N.A X -1 120.832 111.810 147.468 1.00 0.00 C**

**ATOM 1149 C N.A X -1 120.796 110.339 147.867 1.00 0.00 C**

**ATOM 1150 C N.A X -1 119.485 110.197 148.613 1.00 0.00 C**

**ATOM 1151 C N.A X -1 115.908 113.215 149.624 1.00 0.00 C**

**ATOM 1152 C N.A X -1 116.846 113.880 150.646 1.00 0.00 C**

**ATOM 1153 C N.A X -1 118.044 113.096 151.190 1.00 0.00 C**

**ATOM 1154 N N.A X -1 117.717 111.723 151.603 1.00 0.00 N**

**ATOM 1155 C N.A X -1 118.179 111.062 152.690 1.00 0.00 C**

**ATOM 1156 N N.A X -1 118.999 111.633 153.596 1.00 0.00 N**

**ATOM 1157 N N.A X -1 117.818 109.775 152.874 1.00 0.00 N**

**ATOM 1158 C N.A X -1 112.698 111.527 146.791 1.00 0.00 C**

**ATOM 1159 C N.A X -1 112.437 111.640 148.295 1.00 0.00 C**

**ATOM 1160 C N.A X -1 111.870 112.973 148.766 1.00 0.00 C**

**ATOM 1161 N N.A X -1 110.732 113.464 147.990 1.00 0.00 N**

**ATOM 1162 C N.A X -1 109.440 113.105 148.202 1.00 0.00 C**

**ATOM 1163 N N.A X -1 109.113 111.990 148.897 1.00 0.00 N**

**ATOM 1164 N N.A X -1 108.439 113.878 147.750 1.00 0.00 N**

**ATOM 1165 C N.A X -1 112.640 114.616 142.162 1.00 0.00 C**

**ATOM 1166 C N.A X -1 113.659 115.314 143.051 1.00 0.00 C**

**ATOM 1167 C N.A X -1 115.074 114.780 142.942 1.00 0.00 C**

**ATOM 1168 N N.A X -1 115.732 114.907 144.230 1.00 0.00 N**

**ATOM 1169 C N.A X -1 117.037 114.746 144.472 1.00 0.00 C**

**ATOM 1170 N N.A X -1 117.912 114.352 143.538 1.00 0.00 N**

**ATOM 1171 N N.A X -1 117.475 114.988 145.711 1.00 0.00 N**

**ATOM 1172 C N.A X -1 111.620 114.095 138.657 1.00 0.00 C**

**ATOM 1173 C N.A X -1 112.680 114.195 137.550 1.00 0.00 C**

**ATOM 1174 C N.A X -1 113.972 114.851 138.025 1.00 0.00 C**

**ATOM 1175 N N.A X -1 115.170 114.010 137.849 1.00 0.00 N**

**ATOM 1176 C N.A X -1 115.808 113.326 138.832 1.00 0.00 C**

**ATOM 1177 N N.A X -1 115.226 113.040 140.010 1.00 0.00 N**

**ATOM 1178 N N.A X -1 117.093 112.974 138.666 1.00 0.00 N**

**ATOM 1179 C N.A X -1 113.861 110.256 138.795 1.00 0.00 C**

**ATOM 1180 C N.A X -1 114.396 109.788 137.432 1.00 0.00 C**

**ATOM 1181 C N.A X -1 115.847 110.258 137.287 1.00 0.00 C**

**ATOM 1182 C N.A X -1 113.655 110.364 136.234 1.00 0.00 C**

**ATOM 1183 C N.A X -1 109.199 107.866 141.506 1.00 0.00 C**

**ATOM 1184 C N.A X -1 109.322 108.795 142.683 1.00 0.00 C**

**ATOM 1185 C N.A X -1 108.556 109.968 142.724 1.00 0.00 C**

**ATOM 1186 C N.A X -1 108.627 110.812 143.826 1.00 0.00 C**

**ATOM 1187 C N.A X -1 109.445 110.479 144.902 1.00 0.00 C**

**ATOM 1188 C N.A X -1 110.227 109.326 144.860 1.00 0.00 C**

**ATOM 1189 C N.A X -1 110.177 108.492 143.747 1.00 0.00 C**

**ATOM 1190 C N.A X -1 107.277 105.780 137.045 1.00 0.00 C**

**ATOM 1191 C N.A X -1 107.580 104.283 137.107 1.00 0.00 C**

**ATOM 1192 C N.A X -1 109.094 104.153 137.089 1.00 0.00 C**

**ATOM 1193 CA ALA R 57 136.376 111.055 139.465 1.00 0.00 C**

**ATOM 1194 C ALA R 57 135.110 110.437 138.885 1.00 0.00 C**

**ATOM 1195 O ALA R 57 134.871 110.508 137.679 1.00 0.00 O**

**ATOM 1196 N ALA R 57 136.113 111.637 140.776 1.00 0.00 N**

**ATOM 1197 CB ALA R 57 137.482 110.015 139.558 1.00 0.00 C**

**ATOM 1198 CA ALA R 64 130.632 112.472 130.406 1.00 0.00 C**

**ATOM 1199 C ALA R 64 129.873 111.421 129.605 1.00 0.00 C**

**ATOM 1200 O ALA R 64 129.633 111.607 128.406 1.00 0.00 O**

**ATOM 1201 N ALA R 64 130.065 112.621 131.741 1.00 0.00 N**

**ATOM 1202 CB ALA R 64 132.115 112.115 130.501 1.00 0.00 C**

**ATOM 1203 CA ALA R 93 104.351 102.440 106.047 1.00 0.00 C**

**ATOM 1204 C ALA R 93 105.561 102.363 106.969 1.00 0.00 C**

**ATOM 1205 O ALA R 93 105.545 102.957 108.058 1.00 0.00 O**

**ATOM 1206 N ALA R 93 104.732 102.410 104.639 1.00 0.00 N**

**ATOM 1207 CB ALA R 93 103.387 101.293 106.354 1.00 0.00 C**

**ATOM 1208 CA ALA R 102 112.724 109.029 115.490 1.00 0.00 C**

**ATOM 1209 C ALA R 102 112.234 110.281 116.208 1.00 0.00 C**

**ATOM 1210 O ALA R 102 112.981 110.883 116.987 1.00 0.00 O**

**ATOM 1211 N ALA R 102 111.683 108.008 115.423 1.00 0.00 N**

**ATOM 1212 CB ALA R 102 113.212 109.372 114.083 1.00 0.00 C**

**ATOM 1213 CA ALA R 104 109.696 109.988 119.935 1.00 0.00 C**

**ATOM 1214 C ALA R 104 111.050 109.950 120.636 1.00 0.00 C**

**ATOM 1215 O ALA R 104 111.188 110.467 121.749 1.00 0.00 O**

**ATOM 1216 N ALA R 104 109.831 110.345 118.527 1.00 0.00 N**

**ATOM 1217 CB ALA R 104 108.984 108.642 120.069 1.00 0.00 C**

**ATOM 1218 CA ALA R 106 114.248 112.956 119.689 1.00 0.00 C**

**ATOM 1219 C ALA R 106 113.617 113.779 120.799 1.00 0.00 C**

**ATOM 1220 O ALA R 106 114.262 114.680 121.347 1.00 0.00 O**

**ATOM 1221 N ALA R 106 113.719 111.598 119.674 1.00 0.00 N**

**ATOM 1222 CB ALA R 106 114.020 113.630 118.336 1.00 0.00 C**

**ATOM 1223 CA ALA R 159 103.852 90.894 107.158 1.00 0.00 C**

**ATOM 1224 C ALA R 159 104.438 89.535 106.787 1.00 0.00 C**

**ATOM 1225 O ALA R 159 104.411 89.144 105.614 1.00 0.00 O**

**ATOM 1226 N ALA R 159 103.201 90.894 108.470 1.00 0.00 N**

**ATOM 1227 CB ALA R 159 104.941 91.962 107.118 1.00 0.00 C**

**ATOM 1228 CA ALA R 166 99.447 92.083 104.731 1.00 0.00 C**

**ATOM 1229 C ALA R 166 99.049 93.020 103.598 1.00 0.00 C**

**ATOM 1230 O ALA R 166 99.348 94.219 103.653 1.00 0.00 O**

**ATOM 1231 N ALA R 166 98.488 90.993 104.897 1.00 0.00 N**

**ATOM 1232 CB ALA R 166 100.845 91.519 104.486 1.00 0.00 C**

**ATOM 1233 CA ALA R 175 99.467 102.980 109.277 1.00 0.00 C**

**ATOM 1234 C ALA R 175 99.360 104.324 109.990 1.00 0.00 C**

**ATOM 1235 O ALA R 175 99.675 104.427 111.185 1.00 0.00 O**

**ATOM 1236 N ALA R 175 98.301 102.717 108.432 1.00 0.00 N**

**ATOM 1237 CB ALA R 175 100.742 102.922 108.438 1.00 0.00 C**

**ATOM 1238 CA ALA R 193 101.276 114.207 133.791 1.00 0.00 C**

**ATOM 1239 C ALA R 193 101.209 115.729 133.792 1.00 0.00 C**

**ATOM 1240 O ALA R 193 101.929 116.374 134.561 1.00 0.00 O**

**ATOM 1241 N ALA R 193 100.015 113.605 133.374 1.00 0.00 N**

**ATOM 1242 CB ALA R 193 102.413 113.731 132.886 1.00 0.00 C**

**ATOM 1243 CA ALA R 234 105.132 99.383 129.464 1.00 0.00 C**

**ATOM 1244 C ALA R 234 106.214 98.319 129.341 1.00 0.00 C**

**ATOM 1245 O ALA R 234 107.088 98.458 128.479 1.00 0.00 O**

**ATOM 1246 N ALA R 234 104.024 98.994 130.319 1.00 0.00 N**

**ATOM 1247 CB ALA R 234 105.766 100.677 129.980 1.00 0.00 C**

**ATOM 1248 CA ALA R 281 118.851 91.725 123.391 1.00 0.00 C**

**ATOM 1249 C ALA R 281 119.083 93.141 123.904 1.00 0.00 C**

**ATOM 1250 O ALA R 281 119.169 93.351 125.118 1.00 0.00 O**

**ATOM 1251 N ALA R 281 117.848 91.686 122.331 1.00 0.00 N**

**ATOM 1252 CB ALA R 281 120.161 91.117 122.893 1.00 0.00 C**

**ATOM 1253 CA ALA R 298 109.430 100.715 145.686 1.00 0.00 C**

**ATOM 1254 C ALA R 298 108.794 99.830 146.754 1.00 0.00 C**

**ATOM 1255 O ALA R 298 108.052 100.326 147.610 1.00 0.00 O**

**ATOM 1256 N ALA R 298 110.817 100.347 145.419 1.00 0.00 N**

**ATOM 1257 CB ALA R 298 108.618 100.657 144.392 1.00 0.00 C**

**ATOM 1258 CA ALA R 307 127.121 103.573 145.019 1.00 0.00 C**

**ATOM 1259 C ALA R 307 125.769 103.167 144.440 1.00 0.00 C**

**ATOM 1260 O ALA R 307 125.643 102.965 143.225 1.00 0.00 O**

**ATOM 1261 N ALA R 307 126.983 104.554 146.096 1.00 0.00 N**

**ATOM 1262 CB ALA R 307 127.874 102.343 145.526 1.00 0.00 C**

**ATOM 1263 CA ALA R 308 123.400 102.748 144.814 1.00 0.00 C**

**ATOM 1264 C ALA R 308 122.836 103.898 143.990 1.00 0.00 C**

**ATOM 1265 O ALA R 308 122.128 103.661 143.007 1.00 0.00 O**

**ATOM 1266 N ALA R 308 124.746 103.043 145.296 1.00 0.00 N**

**ATOM 1267 CB ALA R 308 122.472 102.428 145.986 1.00 0.00 C**

**ATOM 1268 CA ALA R 317 121.518 106.276 131.456 1.00 0.00 C**

**ATOM 1269 C ALA R 317 121.446 105.155 130.426 1.00 0.00 C**

**ATOM 1270 O ALA R 317 121.431 105.421 129.225 1.00 0.00 O**

**ATOM 1271 N ALA R 317 120.702 105.987 132.632 1.00 0.00 N**

**ATOM 1272 CB ALA R 317 122.966 106.525 131.875 1.00 0.00 C**

**ATOM 1273 CA ALA R 331 117.476 103.157 110.269 1.00 0.00 C**

**ATOM 1274 C ALA R 331 118.735 102.757 109.514 1.00 0.00 C**

**ATOM 1275 O ALA R 331 118.658 102.354 108.352 1.00 0.00 O**

**ATOM 1276 N ALA R 331 117.371 102.488 111.558 1.00 0.00 N**

**ATOM 1277 CB ALA R 331 117.438 104.678 110.459 1.00 0.00 C**

**ATOM 1278 CA GLN R 115 118.196 114.843 134.805 1.00 0.00 C**

**ATOM 1279 C GLN R 115 119.638 114.919 135.292 1.00 0.00 C**

**ATOM 1280 O GLN R 115 119.911 114.650 136.468 1.00 0.00 O**

**ATOM 1281 N GLN R 115 117.893 115.907 133.851 1.00 0.00 N**

**ATOM 1282 CB GLN R 115 117.904 113.479 134.188 1.00 0.00 C**

**ATOM 1283 CG GLN R 115 116.435 113.254 133.904 1.00 0.00 C**

**ATOM 1284 CD GLN R 115 116.169 111.894 133.310 1.00 0.00 C**

**ATOM 1285 OE1 GLN R 115 117.003 110.994 133.397 1.00 0.00 O**

**ATOM 1286 NE2 GLN R 115 114.995 111.727 132.716 1.00 0.00 N**

**ATOM 1287 CA GLN R 213 102.206 115.159 143.473 1.00 0.00 C**

**ATOM 1288 C GLN R 213 100.923 114.336 143.511 1.00 0.00 C**

**ATOM 1289 O GLN R 213 100.067 114.441 142.627 1.00 0.00 O**

**ATOM 1290 N GLN R 213 102.918 115.028 142.203 1.00 0.00 N**

**ATOM 1291 CB GLN R 213 101.868 116.618 143.786 1.00 0.00 C**

**ATOM 1292 CG GLN R 213 103.040 117.530 144.076 1.00 0.00 C**

**ATOM 1293 CD GLN R 213 103.779 117.955 142.850 1.00 0.00 C**

**ATOM 1294 OE1 GLN R 213 103.235 117.929 141.752 1.00 0.00 O**

**ATOM 1295 NE2 GLN R 213 105.043 118.326 143.022 1.00 0.00 N**

**ATOM 1296 CA PRO R 59 130.742 112.288 138.734 1.00 0.00 C**

**ATOM 1297 C PRO R 59 131.155 112.812 137.367 1.00 0.00 C**

**ATOM 1298 O PRO R 59 130.284 112.995 136.507 1.00 0.00 O**

**ATOM 1299 N PRO R 59 131.753 111.415 139.372 1.00 0.00 N**

**ATOM 1300 CD PRO R 59 132.306 112.123 140.553 1.00 0.00 C**

**ATOM 1301 CG PRO R 59 131.821 113.523 140.414 1.00 0.00 C**

**ATOM 1302 CB PRO R 59 130.562 113.433 139.738 1.00 0.00 C**

**ATOM 1303 CA PRO R 113 120.500 117.354 130.616 1.00 0.00 C**

**ATOM 1304 C PRO R 113 119.800 117.831 131.878 1.00 0.00 C**

**ATOM 1305 O PRO R 113 120.479 118.183 132.850 1.00 0.00 O**

**ATOM 1306 N PRO R 113 119.726 116.374 129.820 1.00 0.00 N**

**ATOM 1307 CD PRO R 113 119.418 116.949 128.491 1.00 0.00 C**

**ATOM 1308 CG PRO R 113 119.659 118.408 128.665 1.00 0.00 C**

**ATOM 1309 CB PRO R 113 120.732 118.512 129.636 1.00 0.00 C**

**ATOM 1310 CA PRO R 125 116.276 125.959 140.262 1.00 0.00 C**

**ATOM 1311 C PRO R 125 115.417 126.642 139.207 1.00 0.00 C**

**ATOM 1312 O PRO R 125 115.458 127.870 139.077 1.00 0.00 O**

**ATOM 1313 N PRO R 125 116.398 124.506 140.087 1.00 0.00 N**

**ATOM 1314 CD PRO R 125 117.604 124.139 139.326 1.00 0.00 C**

**ATOM 1315 CG PRO R 125 118.410 125.419 139.298 1.00 0.00 C**

**ATOM 1316 CB PRO R 125 117.730 126.435 140.180 1.00 0.00 C**

**ATOM 1317 CA PRO R 163 100.871 87.547 103.770 1.00 0.00 C**

**ATOM 1318 C PRO R 163 99.623 88.016 103.035 1.00 0.00 C**

**ATOM 1319 O PRO R 163 99.673 89.060 102.374 1.00 0.00 O**

**ATOM 1320 N PRO R 163 100.601 86.739 104.973 1.00 0.00 N**

**ATOM 1321 CD PRO R 163 100.964 85.324 104.776 1.00 0.00 C**

**ATOM 1322 CG PRO R 163 101.268 85.248 103.310 1.00 0.00 C**

**ATOM 1323 CB PRO R 163 101.711 86.602 102.902 1.00 0.00 C**

**ATOM 1324 CA PRO R 172 99.874 104.007 104.451 1.00 0.00 C**

**ATOM 1325 C PRO R 172 98.800 104.720 105.256 1.00 0.00 C**

**ATOM 1326 O PRO R 172 99.095 105.238 106.342 1.00 0.00 O**

**ATOM 1327 N PRO R 172 99.381 102.805 103.752 1.00 0.00 N**

**ATOM 1328 CD PRO R 172 99.699 102.846 102.314 1.00 0.00 C**

**ATOM 1329 CG PRO R 172 99.852 104.309 102.053 1.00 0.00 C**

**ATOM 1330 CB PRO R 172 100.428 104.877 103.311 1.00 0.00 C**

**ATOM 1331 CA PRO R 215 97.700 113.250 148.192 1.00 0.00 C**

**ATOM 1332 C PRO R 215 98.469 112.377 149.175 1.00 0.00 C**

**ATOM 1333 O PRO R 215 98.598 111.164 148.995 1.00 0.00 O**

**ATOM 1334 N PRO R 215 98.035 112.922 146.799 1.00 0.00 N**

**ATOM 1335 CD PRO R 215 96.863 112.400 146.066 1.00 0.00 C**

**ATOM 1336 CG PRO R 215 95.867 112.113 147.148 1.00 0.00 C**

**ATOM 1337 CB PRO R 215 96.188 113.009 148.272 1.00 0.00 C**

**ATOM 1338 CA PRO R 238 105.577 94.776 124.990 1.00 0.00 C**

**ATOM 1339 C PRO R 238 106.639 93.696 124.850 1.00 0.00 C**

**ATOM 1340 O PRO R 238 106.887 93.250 123.724 1.00 0.00 O**

**ATOM 1341 N PRO R 238 104.729 94.662 126.199 1.00 0.00 N**

**ATOM 1342 CD PRO R 238 104.839 95.913 126.986 1.00 0.00 C**

**ATOM 1343 CG PRO R 238 105.330 96.907 126.003 1.00 0.00 C**

**ATOM 1344 CB PRO R 238 106.216 96.164 125.132 1.00 0.00 C**

**ATOM 1345 CA PRO R 289 117.602 96.528 135.209 1.00 0.00 C**

**ATOM 1346 C PRO R 289 116.926 97.685 135.920 1.00 0.00 C**

**ATOM 1347 O PRO R 289 116.721 97.601 137.136 1.00 0.00 O**

**ATOM 1348 N PRO R 289 117.010 96.168 133.901 1.00 0.00 N**

**ATOM 1349 CD PRO R 289 118.036 96.307 132.847 1.00 0.00 C**

**ATOM 1350 CG PRO R 289 119.322 96.254 133.588 1.00 0.00 C**

**ATOM 1351 CB PRO R 289 119.059 96.874 134.865 1.00 0.00 C**

**ATOM 1352 CA PRO R 327 118.451 105.178 115.131 1.00 0.00 C**

**ATOM 1353 C PRO R 327 119.445 104.274 114.418 1.00 0.00 C**

**ATOM 1354 O PRO R 327 119.200 103.904 113.266 1.00 0.00 O**

**ATOM 1355 N PRO R 327 118.190 104.809 116.539 1.00 0.00 N**

**ATOM 1356 CD PRO R 327 118.383 105.989 117.411 1.00 0.00 C**

**ATOM 1357 CG PRO R 327 118.399 107.133 116.457 1.00 0.00 C**

**ATOM 1358 CB PRO R 327 118.980 106.615 115.234 1.00 0.00 C**

**ATOM 1359 CA ASP R 105 113.395 109.348 120.599 1.00 0.00 C**

**ATOM 1360 C ASP R 105 114.052 110.724 120.631 1.00 0.00 C**

**ATOM 1361 O ASP R 105 114.812 111.021 121.562 1.00 0.00 O**

**ATOM 1362 N ASP R 105 112.072 109.390 119.982 1.00 0.00 N**

**ATOM 1363 CB ASP R 105 114.285 108.348 119.861 1.00 0.00 C**

**ATOM 1364 CG ASP R 105 113.785 106.921 119.985 1.00 0.00 C**

**ATOM 1365 OD1 ASP R 105 113.237 106.571 121.052 1.00 0.00 O**

**ATOM 1366 OD2 ASP R 105 113.940 106.149 119.015 1.00 0.00 O**

**ATOM 1367 CA ASP R 128 107.092 124.951 137.602 1.00 0.00 C**

**ATOM 1368 C ASP R 128 106.603 124.540 136.216 1.00 0.00 C**

**ATOM 1369 O ASP R 128 106.336 123.352 135.988 1.00 0.00 O**

**ATOM 1370 N ASP R 128 108.012 126.082 137.543 1.00 0.00 N**

**ATOM 1371 CB ASP R 128 105.904 125.308 138.502 1.00 0.00 C**

**ATOM 1372 CG ASP R 128 105.150 124.087 139.004 1.00 0.00 C**

**ATOM 1373 OD1 ASP R 128 105.560 122.950 138.699 1.00 0.00 O**

**ATOM 1374 OD2 ASP R 128 104.138 124.269 139.712 1.00 0.00 O**

**ATOM 1375 CA ASP R 138 109.324 112.365 129.016 1.00 0.00 C**

**ATOM 1376 C ASP R 138 108.438 111.125 129.114 1.00 0.00 C**

**ATOM 1377 O ASP R 138 108.668 110.138 128.402 1.00 0.00 O**

**ATOM 1378 N ASP R 138 108.549 113.571 128.721 1.00 0.00 N**

**ATOM 1379 CB ASP R 138 110.115 112.567 130.309 1.00 0.00 C**

**ATOM 1380 CG ASP R 138 111.197 111.523 130.503 1.00 0.00 C**

**ATOM 1381 OD1 ASP R 138 111.544 110.827 129.525 1.00 0.00 O**

**ATOM 1382 OD2 ASP R 138 111.705 111.403 131.637 1.00 0.00 O**

**ATOM 1383 CA ASP R 155 103.434 94.870 111.688 1.00 0.00 C**

**ATOM 1384 C ASP R 155 104.309 93.907 110.892 1.00 0.00 C**

**ATOM 1385 O ASP R 155 103.853 93.314 109.909 1.00 0.00 O**

**ATOM 1386 N ASP R 155 103.556 94.628 113.123 1.00 0.00 N**

**ATOM 1387 CB ASP R 155 103.801 96.325 111.378 1.00 0.00 C**

**ATOM 1388 CG ASP R 155 103.449 96.744 109.953 1.00 0.00 C**

**ATOM 1389 OD1 ASP R 155 102.820 95.961 109.209 1.00 0.00 O**

**ATOM 1390 OD2 ASP R 155 103.810 97.877 109.572 1.00 0.00 O**

**ATOM 1391 CA ASP R 168 94.915 94.564 103.555 1.00 0.00 C**

**ATOM 1392 C ASP R 168 95.316 95.217 104.871 1.00 0.00 C**

**ATOM 1393 O ASP R 168 94.444 95.689 105.608 1.00 0.00 O**

**ATOM 1394 N ASP R 168 95.999 93.790 102.969 1.00 0.00 N**

**ATOM 1395 CB ASP R 168 93.690 93.673 103.773 1.00 0.00 C**

**ATOM 1396 CG ASP R 168 93.025 93.269 102.475 1.00 0.00 C**

**ATOM 1397 OD1 ASP R 168 93.269 93.936 101.449 1.00 0.00 O**

**ATOM 1398 OD2 ASP R 168 92.261 92.280 102.479 1.00 0.00 O**

**ATOM 1399 CA ASP R 204 113.679 120.659 156.444 1.00 0.00 C**

**ATOM 1400 C ASP R 204 114.970 120.366 155.686 1.00 0.00 C**

**ATOM 1401 O ASP R 204 116.069 120.594 156.206 1.00 0.00 O**

**ATOM 1402 N ASP R 204 112.644 121.202 155.558 1.00 0.00 N**

**ATOM 1403 CB ASP R 204 113.167 119.427 157.207 1.00 0.00 C**

**ATOM 1404 CG ASP R 204 112.651 118.319 156.304 1.00 0.00 C**

**ATOM 1405 OD1 ASP R 204 112.676 118.468 155.073 1.00 0.00 O**

**ATOM 1406 OD2 ASP R 204 112.213 117.280 156.842 1.00 0.00 O**

**ATOM 1407 CA ASP R 206 115.776 123.234 152.267 1.00 0.00 C**

**ATOM 1408 C ASP R 206 116.130 123.080 150.792 1.00 0.00 C**

**ATOM 1409 O ASP R 206 117.032 123.748 150.280 1.00 0.00 O**

**ATOM 1410 N ASP R 206 115.560 121.926 152.910 1.00 0.00 N**

**ATOM 1411 CB ASP R 206 116.836 124.074 152.994 1.00 0.00 C**

**ATOM 1412 CG ASP R 206 116.263 124.903 154.126 1.00 0.00 C**

**ATOM 1413 OD1 ASP R 206 115.099 125.342 154.013 1.00 0.00 O**

**ATOM 1414 OD2 ASP R 206 116.976 125.115 155.129 1.00 0.00 O**

**ATOM 1415 CA ASP R 216 99.953 112.409 151.124 1.00 0.00 C**

**ATOM 1416 C ASP R 216 99.335 111.433 152.115 1.00 0.00 C**

**ATOM 1417 O ASP R 216 100.086 110.731 152.802 1.00 0.00 O**

**ATOM 1418 N ASP R 216 98.983 113.021 150.225 1.00 0.00 N**

**ATOM 1419 CB ASP R 216 100.710 113.499 151.887 1.00 0.00 C**

**ATOM 1420 CG ASP R 216 101.554 114.369 150.975 1.00 0.00 C**

**ATOM 1421 OD1 ASP R 216 102.010 113.868 149.925 1.00 0.00 O**

**ATOM 1422 OD2 ASP R 216 101.758 115.556 151.305 1.00 0.00 O**

**ATOM 1423 CA ASP R 217 97.327 110.440 153.091 1.00 0.00 C**

**ATOM 1424 C ASP R 217 97.614 109.017 152.622 1.00 0.00 C**

**ATOM 1425 O ASP R 217 97.864 108.123 153.438 1.00 0.00 O**

**ATOM 1426 N ASP R 217 98.004 111.427 152.251 1.00 0.00 N**

**ATOM 1427 CB ASP R 217 95.823 110.710 153.103 1.00 0.00 C**

**ATOM 1428 CG ASP R 217 95.071 109.776 154.028 1.00 0.00 C**

**ATOM 1429 OD1 ASP R 217 95.145 109.972 155.259 1.00 0.00 O**

**ATOM 1430 OD2 ASP R 217 94.407 108.847 153.524 1.00 0.00 O**

**ATOM 1431 CA ASP R 218 97.926 107.516 150.684 1.00 0.00 C**

**ATOM 1432 C ASP R 218 98.914 107.745 149.537 1.00 0.00 C**

**ATOM 1433 O ASP R 218 98.686 107.339 148.399 1.00 0.00 O**

**ATOM 1434 N ASP R 218 97.603 108.800 151.306 1.00 0.00 N**

**ATOM 1435 CB ASP R 218 96.670 106.807 150.180 1.00 0.00 C**

**ATOM 1436 CG ASP R 218 95.797 106.284 151.305 1.00 0.00 C**

**ATOM 1437 OD1 ASP R 218 96.342 105.922 152.368 1.00 0.00 O**

**ATOM 1438 OD2 ASP R 218 94.562 106.239 151.126 1.00 0.00 O**

**ATOM 1439 CA ASP R 223 102.631 105.773 144.061 1.00 0.00 C**

**ATOM 1440 C ASP R 223 103.040 104.361 143.664 1.00 0.00 C**

**ATOM 1441 O ASP R 223 103.466 104.135 142.524 1.00 0.00 O**

**ATOM 1442 N ASP R 223 101.364 105.761 144.784 1.00 0.00 N**

**ATOM 1443 CB ASP R 223 103.712 106.436 144.919 1.00 0.00 C**

**ATOM 1444 CG ASP R 223 105.039 106.598 144.188 1.00 0.00 C**

**ATOM 1445 OD1 ASP R 223 105.059 106.569 142.938 1.00 0.00 O**

**ATOM 1446 OD2 ASP R 223 106.071 106.757 144.872 1.00 0.00 O**

**ATOM 1447 CA ASP R 266 120.642 80.894 104.209 1.00 0.00 C**

**ATOM 1448 C ASP R 266 121.479 81.864 105.044 1.00 0.00 C**

**ATOM 1449 O ASP R 266 121.141 82.155 106.196 1.00 0.00 O**

**ATOM 1450 N ASP R 266 120.581 81.342 102.818 1.00 0.00 N**

**ATOM 1451 CB ASP R 266 121.217 79.471 104.254 1.00 0.00 C**

**ATOM 1452 CG ASP R 266 121.209 78.844 105.652 1.00 0.00 C**

**ATOM 1453 OD1 ASP R 266 120.606 79.394 106.598 1.00 0.00 O**

**ATOM 1454 OD2 ASP R 266 121.823 77.767 105.802 1.00 0.00 O**

**ATOM 1455 CA ASP R 334 117.483 98.527 106.939 1.00 0.00 C**

**ATOM 1456 C ASP R 334 118.105 98.584 105.550 1.00 0.00 C**

**ATOM 1457 O ASP R 334 118.737 99.580 105.184 1.00 0.00 O**

**ATOM 1458 N ASP R 334 118.455 98.941 107.944 1.00 0.00 N**

**ATOM 1459 CB ASP R 334 116.233 99.406 106.987 1.00 0.00 C**

**ATOM 1460 CG ASP R 334 115.363 99.125 108.193 1.00 0.00 C**

**ATOM 1461 OD1 ASP R 334 115.530 98.055 108.815 1.00 0.00 O**

**ATOM 1462 OD2 ASP R 334 114.491 99.964 108.503 1.00 0.00 O**

**ATOM 1463 CA ASN R 77 117.551 111.341 115.582 1.00 0.00 C**

**ATOM 1464 C ASN R 77 117.392 112.512 114.618 1.00 0.00 C**

**ATOM 1465 O ASN R 77 116.472 112.501 113.796 1.00 0.00 O**

**ATOM 1466 N ASN R 77 118.952 110.999 115.819 1.00 0.00 N**

**ATOM 1467 CB ASN R 77 116.859 111.661 116.908 1.00 0.00 C**

**ATOM 1468 CG ASN R 77 116.609 110.427 117.752 1.00 0.00 C**

**ATOM 1469 OD1 ASN R 77 116.159 109.399 117.250 1.00 0.00 O**

**ATOM 1470 ND2 ASN R 77 116.904 110.524 119.043 1.00 0.00 N**

**ATOM 1471 CA ASN R 95 109.220 104.984 106.804 1.00 0.00 C**

**ATOM 1472 C ASN R 95 108.383 105.847 107.745 1.00 0.00 C**

**ATOM 1473 O ASN R 95 108.940 106.655 108.493 1.00 0.00 O**

**ATOM 1474 N ASN R 95 108.636 103.660 106.604 1.00 0.00 N**

**ATOM 1475 CB ASN R 95 109.400 105.687 105.458 1.00 0.00 C**

**ATOM 1476 CG ASN R 95 110.455 105.027 104.593 1.00 0.00 C**

**ATOM 1477 OD1 ASN R 95 111.399 104.421 105.100 1.00 0.00 O**

**ATOM 1478 ND2 ASN R 95 110.300 105.140 103.279 1.00 0.00 N**

**ATOM 1479 CA ASN R 100 107.348 108.470 114.488 1.00 0.00 C**

**ATOM 1480 C ASN R 100 108.273 107.995 115.606 1.00 0.00 C**

**ATOM 1481 O ASN R 100 108.322 108.619 116.675 1.00 0.00 O**

**ATOM 1482 N ASN R 100 107.944 108.284 113.165 1.00 0.00 N**

**ATOM 1483 CB ASN R 100 106.007 107.745 114.562 1.00 0.00 C**

**ATOM 1484 CG ASN R 100 104.966 108.355 113.646 1.00 0.00 C**

**ATOM 1485 OD1 ASN R 100 104.740 109.565 113.666 1.00 0.00 O**

**ATOM 1486 ND2 ASN R 100 104.336 107.523 112.828 1.00 0.00 N**

**ATOM 1487 CA ASN R 122 120.629 118.069 143.732 1.00 0.00 C**

**ATOM 1488 C ASN R 122 119.439 119.014 143.935 1.00 0.00 C**

**ATOM 1489 O ASN R 122 118.565 118.746 144.763 1.00 0.00 O**

**ATOM 1490 N ASN R 122 121.584 118.614 142.756 1.00 0.00 N**

**ATOM 1491 CB ASN R 122 121.318 117.702 145.066 1.00 0.00 C**

**ATOM 1492 CG ASN R 122 120.481 116.792 145.959 1.00 0.00 C**

**ATOM 1493 OD1 ASN R 122 119.551 116.130 145.498 1.00 0.00 O**

**ATOM 1494 ND2 ASN R 122 120.812 116.760 147.244 1.00 0.00 N**

**ATOM 1495 CA ASN R 141 107.048 110.115 124.389 1.00 0.00 C**

**ATOM 1496 C ASN R 141 107.822 108.844 124.723 1.00 0.00 C**

**ATOM 1497 O ASN R 141 108.168 108.107 123.795 1.00 0.00 O**

**ATOM 1498 N ASN R 141 106.269 110.633 125.500 1.00 0.00 N**

**ATOM 1499 CB ASN R 141 107.977 111.214 123.883 1.00 0.00 C**

**ATOM 1500 CG ASN R 141 107.219 112.298 123.144 1.00 0.00 C**

**ATOM 1501 OD1 ASN R 141 106.444 112.012 122.231 1.00 0.00 O**

**ATOM 1502 ND2 ASN R 141 107.376 113.535 123.585 1.00 0.00 N**

**ATOM 1503 CA ASN R 179 99.928 105.892 114.701 1.00 0.00 C**

**ATOM 1504 C ASN R 179 99.222 107.029 115.442 1.00 0.00 C**

**ATOM 1505 O ASN R 179 99.624 107.369 116.563 1.00 0.00 O**

**ATOM 1506 N ASN R 179 98.994 105.017 113.989 1.00 0.00 N**

**ATOM 1507 CB ASN R 179 100.963 106.444 113.724 1.00 0.00 C**

**ATOM 1508 CG ASN R 179 101.953 105.388 113.277 1.00 0.00 C**

**ATOM 1509 OD1 ASN R 179 102.478 104.630 114.092 1.00 0.00 O**

**ATOM 1510 ND2 ASN R 179 102.202 105.322 111.976 1.00 0.00 N**

**ATOM 1511 CA ASN R 268 120.955 86.268 104.509 1.00 0.00 C**

**ATOM 1512 C ASN R 268 119.924 86.066 105.621 1.00 0.00 C**

**ATOM 1513 O ASN R 268 119.655 86.987 106.407 1.00 0.00 O**

**ATOM 1514 N ASN R 268 121.818 85.093 104.353 1.00 0.00 N**

**ATOM 1515 CB ASN R 268 120.258 86.586 103.186 1.00 0.00 C**

**ATOM 1516 CG ASN R 268 121.224 87.046 102.114 1.00 0.00 C**

**ATOM 1517 OD1 ASN R 268 122.308 87.547 102.412 1.00 0.00 O**

**ATOM 1518 ND2 ASN R 268 120.836 86.876 100.855 1.00 0.00 N**

**ATOM 1519 CA ASN R 322 118.581 102.365 124.196 1.00 0.00 C**

**ATOM 1520 C ASN R 322 117.920 103.306 123.188 1.00 0.00 C**

**ATOM 1521 O ASN R 322 117.912 103.034 121.981 1.00 0.00 O**

**ATOM 1522 N ASN R 322 119.399 103.109 125.160 1.00 0.00 N**

**ATOM 1523 CB ASN R 322 117.513 101.542 124.911 1.00 0.00 C**

**ATOM 1524 CG ASN R 322 116.718 100.645 123.960 1.00 0.00 C**

**ATOM 1525 OD1 ASN R 322 117.128 100.384 122.827 1.00 0.00 O**

**ATOM 1526 ND2 ASN R 322 115.580 100.165 124.427 1.00 0.00 N**

**ATOM 1527 CA ASN R 326 117.512 103.038 118.228 1.00 0.00 C**

**ATOM 1528 C ASN R 326 117.686 103.576 116.800 1.00 0.00 C**

**ATOM 1529 O ASN R 326 117.279 102.856 115.870 1.00 0.00 O**

**ATOM 1530 N ASN R 326 118.611 103.306 119.161 1.00 0.00 N**

**ATOM 1531 CB ASN R 326 116.181 103.531 118.823 1.00 0.00 C**

**ATOM 1532 CG ASN R 326 115.634 102.597 119.883 1.00 0.00 C**

**ATOM 1533 OD1 ASN R 326 115.784 101.379 119.790 1.00 0.00 O**

**ATOM 1534 ND2 ASN R 326 114.977 103.162 120.889 1.00 0.00 N**

**ATOM 1535 CA ASN R 336 116.065 99.942 101.903 1.00 0.00 C**

**ATOM 1536 C ASN R 336 116.495 101.360 102.257 1.00 0.00 C**

**ATOM 1537 O ASN R 336 116.617 102.213 101.366 1.00 0.00 O**

**ATOM 1538 N ASN R 336 116.705 98.963 102.773 1.00 0.00 N**

**ATOM 1539 CB ASN R 336 114.539 99.809 102.008 1.00 0.00 C**

**ATOM 1540 CG ASN R 336 113.795 100.503 100.873 1.00 0.00 C**

**ATOM 1541 OD1 ASN R 336 114.396 101.084 99.969 1.00 0.00 O**

**ATOM 1542 ND2 ASN R 336 112.469 100.439 100.920 1.00 0.00 N**

**ATOM 1543 CA LYS R 89 109.072 110.663 100.643 1.00 0.00 C**

**ATOM 1544 C LYS R 89 108.389 109.530 101.403 1.00 0.00 C**

**ATOM 1545 O LYS R 89 108.715 108.352 101.226 1.00 0.00 O**

**ATOM 1546 N LYS R 89 110.485 110.373 100.433 1.00 0.00 N**

**ATOM 1547 CB LYS R 89 108.385 110.902 99.299 1.00 0.00 C**

**ATOM 1548 CG LYS R 89 108.698 109.850 98.247 1.00 0.00 C**

**ATOM 1549 CD LYS R 89 108.106 110.221 96.899 1.00 0.00 C**

**ATOM 1550 CE LYS R 89 108.413 109.162 95.855 1.00 0.00 C**

**ATOM 1551 NZ LYS R 89 109.880 108.999 95.655 1.00 0.00 N**

**ATOM 1552 CA LYS R 91 104.972 106.611 100.674 1.00 0.00 C**

**ATOM 1553 C LYS R 91 104.736 105.210 101.219 1.00 0.00 C**

**ATOM 1554 O LYS R 91 103.664 104.643 100.980 1.00 0.00 O**

**ATOM 1555 N LYS R 91 105.957 107.360 101.442 1.00 0.00 N**

**ATOM 1556 CB LYS R 91 105.391 106.523 99.202 1.00 0.00 C**

**ATOM 1557 CG LYS R 91 105.569 107.880 98.546 1.00 0.00 C**

**ATOM 1558 CD LYS R 91 104.305 108.720 98.669 1.00 0.00 C**

**ATOM 1559 CE LYS R 91 104.489 110.099 98.056 1.00 0.00 C**

**ATOM 1560 NZ LYS R 91 103.272 110.942 98.220 1.00 0.00 N**

**ATOM 1561 CA LYS R 132 105.383 120.068 134.114 1.00 0.00 C**

**ATOM 1562 C LYS R 132 105.579 119.621 132.666 1.00 0.00 C**

**ATOM 1563 O LYS R 132 105.242 118.478 132.317 1.00 0.00 O**

**ATOM 1564 N LYS R 132 106.570 120.731 134.656 1.00 0.00 N**

**ATOM 1565 CB LYS R 132 104.177 121.002 134.225 1.00 0.00 C**

**ATOM 1566 CG LYS R 132 103.795 121.356 135.656 1.00 0.00 C**

**ATOM 1567 CD LYS R 132 102.740 122.453 135.701 1.00 0.00 C**

**ATOM 1568 CE LYS R 132 101.406 121.988 135.155 1.00 0.00 C**

**ATOM 1569 NZ LYS R 132 100.778 120.981 136.053 1.00 0.00 N**

**ATOM 1570 CA LYS R 165 96.271 90.023 105.278 1.00 0.00 C**

**ATOM 1571 C LYS R 165 97.207 91.222 105.215 1.00 0.00 C**

**ATOM 1572 O LYS R 165 96.769 92.347 105.490 1.00 0.00 O**

**ATOM 1573 N LYS R 165 96.771 88.900 104.494 1.00 0.00 N**

**ATOM 1574 CB LYS R 165 96.034 89.619 106.742 1.00 0.00 C**

**ATOM 1575 CG LYS R 165 94.704 88.890 107.064 1.00 0.00 C**

**ATOM 1576 CD LYS R 165 94.527 87.520 106.412 1.00 0.00 C**

**ATOM 1577 CE LYS R 165 93.198 86.889 106.792 1.00 0.00 C**

**ATOM 1578 NZ LYS R 165 93.076 86.696 108.263 1.00 0.00 N**

**ATOM 1579 CA LYS R 174 95.913 102.484 107.946 1.00 0.00 C**

**ATOM 1580 C LYS R 174 97.060 102.662 108.936 1.00 0.00 C**

**ATOM 1581 O LYS R 174 96.831 102.717 110.150 1.00 0.00 O**

**ATOM 1582 N LYS R 174 96.109 103.289 106.742 1.00 0.00 N**

**ATOM 1583 CB LYS R 174 95.755 101.013 107.566 1.00 0.00 C**

**ATOM 1584 CG LYS R 174 95.096 100.160 108.634 1.00 0.00 C**

**ATOM 1585 CD LYS R 174 94.760 98.779 108.097 1.00 0.00 C**

**ATOM 1586 CE LYS R 174 94.253 97.865 109.199 1.00 0.00 C**

**ATOM 1587 NZ LYS R 174 92.955 98.332 109.758 1.00 0.00 N**

**ATOM 1588 CA LYS R 176 98.714 106.673 109.888 1.00 0.00 C**

**ATOM 1589 C LYS R 176 97.624 106.623 110.953 1.00 0.00 C**

**ATOM 1590 O LYS R 176 97.772 107.224 112.021 1.00 0.00 O**

**ATOM 1591 N LYS R 176 98.916 105.363 109.271 1.00 0.00 N**

**ATOM 1592 CB LYS R 176 98.387 107.727 108.829 1.00 0.00 C**

**ATOM 1593 CG LYS R 176 99.535 108.043 107.884 1.00 0.00 C**

**ATOM 1594 CD LYS R 176 99.120 109.042 106.817 1.00 0.00 C**

**ATOM 1595 CE LYS R 176 100.259 109.313 105.849 1.00 0.00 C**

**ATOM 1596 NZ LYS R 176 99.859 110.252 104.767 1.00 0.00 N**

**ATOM 1597 CA LYS R 200 107.179 121.407 145.689 1.00 0.00 C**

**ATOM 1598 C LYS R 200 108.501 121.621 146.422 1.00 0.00 C**

**ATOM 1599 O LYS R 200 109.498 120.951 146.141 1.00 0.00 O**

**ATOM 1600 N LYS R 200 107.402 121.165 144.263 1.00 0.00 N**

**ATOM 1601 CB LYS R 200 106.385 120.280 146.387 1.00 0.00 C**

**ATOM 1602 CG LYS R 200 107.035 118.906 146.412 1.00 0.00 C**

**ATOM 1603 CD LYS R 200 106.402 118.006 147.467 1.00 0.00 C**

**ATOM 1604 CE LYS R 200 104.961 117.679 147.162 1.00 0.00 C**

**ATOM 1605 NZ LYS R 200 104.392 116.712 148.139 1.00 0.00 N**

**ATOM 1606 CA LYS R 227 104.538 102.125 139.451 1.00 0.00 C**

**ATOM 1607 C LYS R 227 104.252 100.715 138.939 1.00 0.00 C**

**ATOM 1608 O LYS R 227 104.854 100.294 137.944 1.00 0.00 O**

**ATOM 1609 N LYS R 227 103.325 102.807 139.901 1.00 0.00 N**

**ATOM 1610 CB LYS R 227 105.577 102.080 140.572 1.00 0.00 C**

**ATOM 1611 CG LYS R 227 106.208 103.427 140.891 1.00 0.00 C**

**ATOM 1612 CD LYS R 227 107.160 103.323 142.071 1.00 0.00 C**

**ATOM 1613 CE LYS R 227 107.861 104.644 142.341 1.00 0.00 C**

**ATOM 1614 NZ LYS R 227 108.773 104.555 143.516 1.00 0.00 N**

**ATOM 1615 CA LYS R 254 112.558 78.448 108.695 1.00 0.00 C**

**ATOM 1616 C LYS R 254 111.816 77.474 107.783 1.00 0.00 C**

**ATOM 1617 O LYS R 254 112.445 76.567 107.228 1.00 0.00 O**

**ATOM 1618 N LYS R 254 111.851 79.719 108.859 1.00 0.00 N**

**ATOM 1619 CB LYS R 254 112.817 77.779 110.048 1.00 0.00 C**

**ATOM 1620 CG LYS R 254 113.783 78.526 110.950 1.00 0.00 C**

**ATOM 1621 CD LYS R 254 115.185 78.553 110.365 1.00 0.00 C**

**ATOM 1622 CE LYS R 254 116.156 79.251 111.303 1.00 0.00 C**

**ATOM 1623 NZ LYS R 254 117.525 79.336 110.727 1.00 0.00 N**

**ATOM 1624 CA LYS R 265 120.129 82.925 101.012 1.00 0.00 C**

**ATOM 1625 C LYS R 265 120.030 82.515 102.478 1.00 0.00 C**

**ATOM 1626 O LYS R 265 119.430 83.229 103.292 1.00 0.00 O**

**ATOM 1627 N LYS R 265 121.412 82.520 100.444 1.00 0.00 N**

**ATOM 1628 CB LYS R 265 118.983 82.336 100.193 1.00 0.00 C**

**ATOM 1629 CG LYS R 265 117.647 83.006 100.445 1.00 0.00 C**

**ATOM 1630 CD LYS R 265 117.630 84.408 99.854 1.00 0.00 C**

**ATOM 1631 CE LYS R 265 117.583 84.367 98.333 1.00 0.00 C**

**ATOM 1632 NZ LYS R 265 117.664 85.730 97.738 1.00 0.00 N**

**ATOM 1633 CA LYS R 338 120.810 102.216 102.748 1.00 0.00 C**

**ATOM 1634 C LYS R 338 120.732 102.651 101.288 1.00 0.00 C**

**ATOM 1635 O LYS R 338 121.465 103.548 100.856 1.00 0.00 O**

**ATOM 1636 N LYS R 338 119.479 102.111 103.338 1.00 0.00 N**

**ATOM 1637 CB LYS R 338 121.543 100.880 102.872 1.00 0.00 C**

**ATOM 1638 CG LYS R 338 121.858 100.471 104.299 1.00 0.00 C**

**ATOM 1639 CD LYS R 338 122.495 99.093 104.345 1.00 0.00 C**

**ATOM 1640 CE LYS R 338 122.772 98.658 105.774 1.00 0.00 C**

**ATOM 1641 NZ LYS R 338 123.346 97.285 105.834 1.00 0.00 N**

**ATOM 1642 CA CYS R 131 108.890 120.894 135.395 1.00 0.00 C**

**ATOM 1643 C CYS R 131 107.745 120.097 134.775 1.00 0.00 C**

**ATOM 1644 O CYS R 131 107.924 118.934 134.381 1.00 0.00 O**

**ATOM 1645 N CYS R 131 109.098 122.173 134.713 1.00 0.00 N**

**ATOM 1646 CB CYS R 131 108.624 121.165 136.879 1.00 0.00 C**

**ATOM 1647 SG CYS R 131 108.253 119.751 137.948 1.00 0.00 S**

**ATOM 1648 CA CYS R 161 103.078 85.119 108.953 1.00 0.00 C**

**ATOM 1649 C CYS R 161 101.730 85.136 108.243 1.00 0.00 C**

**ATOM 1650 O CYS R 161 101.189 84.066 107.941 1.00 0.00 O**

**ATOM 1651 N CYS R 161 103.898 86.296 108.685 1.00 0.00 N**

**ATOM 1652 CB CYS R 161 102.858 84.963 110.460 1.00 0.00 C**

**ATOM 1653 SG CYS R 161 104.378 84.675 111.389 1.00 0.00 S**

**ATOM 1654 CA CYS R 181 95.730 105.924 117.858 1.00 0.00 C**

**ATOM 1655 C CYS R 181 96.784 105.716 118.947 1.00 0.00 C**

**ATOM 1656 O CYS R 181 96.466 105.796 120.138 1.00 0.00 O**

**ATOM 1657 N CYS R 181 96.281 106.628 116.698 1.00 0.00 N**

**ATOM 1658 CB CYS R 181 95.115 104.586 117.445 1.00 0.00 C**

**ATOM 1659 SG CYS R 181 93.645 104.713 116.401 1.00 0.00 S**

**ATOM 1660 CA CYS R 210 110.047 118.385 140.803 1.00 0.00 C**

**ATOM 1661 C CYS R 210 109.536 116.948 140.725 1.00 0.00 C**

**ATOM 1662 O CYS R 210 110.314 116.037 140.422 1.00 0.00 O**

**ATOM 1663 N CYS R 210 111.007 118.480 141.898 1.00 0.00 N**

**ATOM 1664 CB CYS R 210 110.742 118.801 139.503 1.00 0.00 C**

**ATOM 1665 SG CYS R 210 109.901 118.574 137.915 1.00 0.00 S**

**ATOM 1666 CA CYS R 229 100.607 99.970 136.303 1.00 0.00 C**

**ATOM 1667 C CYS R 229 101.573 100.313 135.171 1.00 0.00 C**

**ATOM 1668 O CYS R 229 101.504 99.721 134.083 1.00 0.00 O**

**ATOM 1669 N CYS R 229 101.314 99.773 137.572 1.00 0.00 N**

**ATOM 1670 CB CYS R 229 99.539 101.057 136.450 1.00 0.00 C**

**ATOM 1671 SG CYS R 229 98.157 100.623 137.535 1.00 0.00 S**

**ATOM 1672 CA CYS R 245 106.012 87.867 117.337 1.00 0.00 C**

**ATOM 1673 C CYS R 245 107.295 87.458 116.620 1.00 0.00 C**

**ATOM 1674 O CYS R 245 107.238 86.816 115.566 1.00 0.00 O**

**ATOM 1675 N CYS R 245 106.002 87.448 118.738 1.00 0.00 N**

**ATOM 1676 CB CYS R 245 105.793 89.378 117.234 1.00 0.00 C**

**ATOM 1677 SG CYS R 245 104.090 89.911 117.550 1.00 0.00 S**

**ATOM 1678 CA CYS R 286 118.471 98.177 128.594 1.00 0.00 C**

**ATOM 1679 C CYS R 286 117.271 98.440 129.503 1.00 0.00 C**

**ATOM 1680 O CYS R 286 117.384 99.250 130.429 1.00 0.00 O**

**ATOM 1681 N CYS R 286 118.601 96.786 128.188 1.00 0.00 N**

**ATOM 1682 CB CYS R 286 118.406 99.078 127.367 1.00 0.00 C**

**ATOM 1683 SG CYS R 286 119.950 99.164 126.426 1.00 0.00 S**

**ATOM 1684 CA CYS R 315 120.597 102.267 134.779 1.00 0.00 C**

**ATOM 1685 C CYS R 315 119.642 103.202 134.044 1.00 0.00 C**

**ATOM 1686 O CYS R 315 119.106 102.833 132.989 1.00 0.00 O**

**ATOM 1687 N CYS R 315 121.683 103.004 135.422 1.00 0.00 N**

**ATOM 1688 CB CYS R 315 119.842 101.423 135.807 1.00 0.00 C**

**ATOM 1689 SG CYS R 315 120.810 100.100 136.571 1.00 0.00 S**

**ATOM 1690 CA CYS R 340 117.776 105.570 99.857 1.00 0.00 C**

**ATOM 1691 C CYS R 340 118.901 106.538 100.209 1.00 0.00 C**

**ATOM 1692 O CYS R 340 118.956 107.647 99.666 1.00 0.00 O**

**ATOM 1693 N CYS R 340 118.231 104.184 99.883 1.00 0.00 N**

**ATOM 1694 CB CYS R 340 116.596 105.758 100.810 1.00 0.00 C**

**ATOM 1695 SG CYS R 340 115.080 104.914 100.302 1.00 0.00 S**

**ATOM 1696 CA SER R 67 126.924 113.940 127.470 1.00 0.00 C**

**ATOM 1697 C SER R 67 127.084 113.194 126.148 1.00 0.00 C**

**ATOM 1698 O SER R 67 126.554 113.632 125.118 1.00 0.00 O**

**ATOM 1699 N SER R 67 126.665 113.028 128.586 1.00 0.00 N**

**ATOM 1700 CB SER R 67 128.168 114.779 127.761 1.00 0.00 C**

**ATOM 1701 OG SER R 67 127.960 115.630 128.875 1.00 0.00 O**

**ATOM 1702 CA SER R 78 118.208 114.624 113.744 1.00 0.00 C**

**ATOM 1703 C SER R 78 118.486 114.239 112.297 1.00 0.00 C**

**ATOM 1704 O SER R 78 117.764 114.662 111.385 1.00 0.00 O**

**ATOM 1705 N SER R 78 118.331 113.466 114.622 1.00 0.00 N**

**ATOM 1706 CB SER R 78 119.154 115.733 114.202 1.00 0.00 C**

**ATOM 1707 OG SER R 78 118.862 116.135 115.530 1.00 0.00 O**

**ATOM 1708 CA SER R 116 121.974 115.439 134.765 1.00 0.00 C**

**ATOM 1709 C SER R 116 122.096 116.565 135.791 1.00 0.00 C**

**ATOM 1710 O SER R 116 122.842 116.450 136.774 1.00 0.00 O**

**ATOM 1711 N SER R 116 120.578 115.208 134.381 1.00 0.00 N**

**ATOM 1712 CB SER R 116 122.811 115.747 133.523 1.00 0.00 C**

**ATOM 1713 OG SER R 116 124.170 115.954 133.864 1.00 0.00 O**

**ATOM 1714 CA SER R 123 118.241 121.032 143.238 1.00 0.00 C**

**ATOM 1715 C SER R 123 117.691 121.344 141.852 1.00 0.00 C**

**ATOM 1716 O SER R 123 118.401 121.249 140.847 1.00 0.00 O**

**ATOM 1717 N SER R 123 119.336 120.079 143.141 1.00 0.00 N**

**ATOM 1718 CB SER R 123 118.684 122.331 143.922 1.00 0.00 C**

**ATOM 1719 OG SER R 123 117.621 123.265 143.969 1.00 0.00 O**

**ATOM 1720 CA SER R 136 104.798 115.244 130.372 1.00 0.00 C**

**ATOM 1721 C SER R 136 105.413 114.735 129.073 1.00 0.00 C**

**ATOM 1722 O SER R 136 105.015 113.675 128.575 1.00 0.00 O**

**ATOM 1723 N SER R 136 105.815 115.716 131.309 1.00 0.00 N**

**ATOM 1724 CB SER R 136 103.795 116.361 130.086 1.00 0.00 C**

**ATOM 1725 OG SER R 136 102.814 115.938 129.156 1.00 0.00 O**

**ATOM 1726 CA SER R 145 108.410 105.068 121.700 1.00 0.00 C**

**ATOM 1727 C SER R 145 108.211 103.567 121.878 1.00 0.00 C**

**ATOM 1728 O SER R 145 108.412 102.788 120.935 1.00 0.00 O**

**ATOM 1729 N SER R 145 107.294 105.826 122.262 1.00 0.00 N**

**ATOM 1730 CB SER R 145 109.720 105.520 122.345 1.00 0.00 C**

**ATOM 1731 OG SER R 145 110.810 104.726 121.910 1.00 0.00 O**

**ATOM 1732 CA SER R 153 107.042 94.201 115.720 1.00 0.00 C**

**ATOM 1733 C SER R 153 105.792 93.461 115.245 1.00 0.00 C**

**ATOM 1734 O SER R 153 105.892 92.464 114.517 1.00 0.00 O**

**ATOM 1735 N SER R 153 106.895 95.650 115.586 1.00 0.00 N**

**ATOM 1736 CB SER R 153 107.360 93.841 117.171 1.00 0.00 C**

**ATOM 1737 OG SER R 153 107.520 92.442 117.323 1.00 0.00 O**

**ATOM 1738 CA SER R 186 99.718 108.104 125.096 1.00 0.00 C**

**ATOM 1739 C SER R 186 99.209 109.399 125.725 1.00 0.00 C**

**ATOM 1740 O SER R 186 99.637 109.761 126.830 1.00 0.00 O**

**ATOM 1741 N SER R 186 98.712 107.499 124.227 1.00 0.00 N**

**ATOM 1742 CB SER R 186 101.006 108.362 124.316 1.00 0.00 C**

**ATOM 1743 OG SER R 186 101.560 107.152 123.828 1.00 0.00 O**

**ATOM 1744 CA SER R 187 97.804 111.409 125.472 1.00 0.00 C**

**ATOM 1745 C SER R 187 97.055 111.322 126.800 1.00 0.00 C**

**ATOM 1746 O SER R 187 96.981 112.333 127.500 1.00 0.00 O**

**ATOM 1747 N SER R 187 98.355 110.137 125.001 1.00 0.00 N**

**ATOM 1748 CB SER R 187 96.882 112.009 124.408 1.00 0.00 C**

**ATOM 1749 OG SER R 187 96.336 113.242 124.843 1.00 0.00 O**

**ATOM 1750 CA SER R 188 95.877 110.018 128.484 1.00 0.00 C**

**ATOM 1751 C SER R 188 96.896 110.185 129.607 1.00 0.00 C**

**ATOM 1752 O SER R 188 96.683 110.959 130.553 1.00 0.00 O**

**ATOM 1753 N SER R 188 96.507 110.158 127.170 1.00 0.00 N**

**ATOM 1754 CB SER R 188 95.178 108.662 128.585 1.00 0.00 C**

**ATOM 1755 OG SER R 188 96.117 107.601 128.546 1.00 0.00 O**

**ATOM 1756 CA SER R 192 97.666 112.974 133.642 1.00 0.00 C**

**ATOM 1757 C SER R 192 98.962 113.566 134.194 1.00 0.00 C**

**ATOM 1758 O SER R 192 99.008 114.015 135.345 1.00 0.00 O**

**ATOM 1759 N SER R 192 97.486 113.291 132.224 1.00 0.00 N**

**ATOM 1760 CB SER R 192 97.640 111.459 133.846 1.00 0.00 C**

**ATOM 1761 OG SER R 192 96.374 110.922 133.504 1.00 0.00 O**

**ATOM 1762 CA SER R 211 107.710 115.377 140.990 1.00 0.00 C**

**ATOM 1763 C SER R 211 106.196 115.360 140.800 1.00 0.00 C**

**ATOM 1764 O SER R 211 105.531 116.395 140.794 1.00 0.00 O**

**ATOM 1765 N SER R 211 108.232 116.743 140.963 1.00 0.00 N**

**ATOM 1766 CB SER R 211 108.069 114.668 142.304 1.00 0.00 C**

**ATOM 1767 OG SER R 211 107.408 115.267 143.404 1.00 0.00 O**

**ATOM 1768 CA SER R 220 103.005 105.434 149.156 1.00 0.00 C**

**ATOM 1769 C SER R 220 102.169 104.390 148.427 1.00 0.00 C**

**ATOM 1770 O SER R 220 102.678 103.712 147.525 1.00 0.00 O**

**ATOM 1771 N SER R 220 102.247 106.652 149.434 1.00 0.00 N**

**ATOM 1772 CB SER R 220 103.555 104.855 150.460 1.00 0.00 C**

**ATOM 1773 OG SER R 220 104.297 103.672 150.222 1.00 0.00 O**

**ATOM 1774 CA SER R 255 109.700 76.788 106.748 1.00 0.00 C**

**ATOM 1775 C SER R 255 109.528 77.369 105.346 1.00 0.00 C**

**ATOM 1776 O SER R 255 108.620 76.950 104.619 1.00 0.00 O**

**ATOM 1777 N SER R 255 110.503 77.638 107.615 1.00 0.00 N**

**ATOM 1778 CB SER R 255 108.330 76.536 107.383 1.00 0.00 C**

**ATOM 1779 OG SER R 255 108.459 75.862 108.623 1.00 0.00 O**

**ATOM 1780 CA SER R 260 116.668 77.248 102.595 1.00 0.00 C**

**ATOM 1781 C SER R 260 117.105 76.736 101.229 1.00 0.00 C**

**ATOM 1782 O SER R 260 117.731 75.673 101.153 1.00 0.00 O**

**ATOM 1783 N SER R 260 115.826 78.432 102.496 1.00 0.00 N**

**ATOM 1784 CB SER R 260 115.934 76.137 103.354 1.00 0.00 C**

**ATOM 1785 OG SER R 260 114.745 75.760 102.681 1.00 0.00 O**

**ATOM 1786 CA SER R 262 120.750 78.112 98.747 1.00 0.00 C**

**ATOM 1787 C SER R 262 121.502 78.595 99.978 1.00 0.00 C**

**ATOM 1788 O SER R 262 120.915 79.114 100.931 1.00 0.00 O**

**ATOM 1789 N SER R 262 119.327 78.005 99.034 1.00 0.00 N**

**ATOM 1790 CB SER R 262 121.015 79.056 97.565 1.00 0.00 C**

**ATOM 1791 OG SER R 262 122.404 79.172 97.306 1.00 0.00 O**

**ATOM 1792 CA SER R 301 113.515 97.701 150.691 1.00 0.00 C**

**ATOM 1793 C SER R 301 113.805 99.136 150.276 1.00 0.00 C**

**ATOM 1794 O SER R 301 113.942 99.433 149.090 1.00 0.00 O**

**ATOM 1795 N SER R 301 112.714 97.044 149.664 1.00 0.00 N**

**ATOM 1796 CB SER R 301 114.831 96.954 150.943 1.00 0.00 C**

**ATOM 1797 OG SER R 301 115.633 96.926 149.776 1.00 0.00 O**

**ATOM 1798 CA SER R 303 117.836 102.643 151.238 1.00 0.00 C**

**ATOM 1799 C SER R 303 118.306 103.856 150.444 1.00 0.00 C**

**ATOM 1800 O SER R 303 117.623 104.327 149.530 1.00 0.00 O**

**ATOM 1801 N SER R 303 116.490 102.292 150.806 1.00 0.00 N**

**ATOM 1802 CB SER R 303 118.792 101.458 151.072 1.00 0.00 C**

**ATOM 1803 OG SER R 303 120.103 101.796 151.489 1.00 0.00 O**

**ATOM 1804 CA SER R 305 123.708 104.642 149.257 1.00 0.00 C**

**ATOM 1805 C SER R 305 124.270 105.362 148.037 1.00 0.00 C**

**ATOM 1806 O SER R 305 123.605 105.442 146.998 1.00 0.00 O**

**ATOM 1807 N SER R 305 122.259 104.778 149.300 1.00 0.00 N**

**ATOM 1808 CB SER R 305 124.122 103.168 149.235 1.00 0.00 C**

**ATOM 1809 OG SER R 305 123.673 102.496 150.399 1.00 0.00 O**

**ATOM 1810 CA SER R 310 125.434 105.962 140.912 1.00 0.00 C**

**ATOM 1811 C SER R 310 124.903 104.852 140.014 1.00 0.00 C**

**ATOM 1812 O SER R 310 124.742 105.056 138.806 1.00 0.00 O**

**ATOM 1813 N SER R 310 124.700 106.024 142.171 1.00 0.00 N**

**ATOM 1814 CB SER R 310 126.925 105.764 141.180 1.00 0.00 C**

**ATOM 1815 OG SER R 310 127.642 105.634 139.966 1.00 0.00 O**

**ATOM 1816 CA SER R 311 124.072 102.569 139.805 1.00 0.00 C**

**ATOM 1817 C SER R 311 122.661 102.889 139.318 1.00 0.00 C**

**ATOM 1818 O SER R 311 122.274 102.485 138.215 1.00 0.00 O**

**ATOM 1819 N SER R 311 124.636 103.670 140.584 1.00 0.00 N**

**ATOM 1820 CB SER R 311 124.071 101.283 140.630 1.00 0.00 C**

**ATOM 1821 OG SER R 311 123.255 101.412 141.778 1.00 0.00 O**

**ATOM 1822 CA SER R 323 116.736 105.405 122.794 1.00 0.00 C**

**ATOM 1823 C SER R 323 117.784 106.071 121.910 1.00 0.00 C**

**ATOM 1824 O SER R 323 117.477 106.489 120.788 1.00 0.00 O**

**ATOM 1825 N SER R 323 117.334 104.406 123.678 1.00 0.00 N**

**ATOM 1826 CB SER R 323 115.988 106.455 123.616 1.00 0.00 C**

**ATOM 1827 OG SER R 323 115.010 105.851 124.446 1.00 0.00 O**

**ATOM 1828 CA SER R 324 120.087 106.760 121.588 1.00 0.00 C**

**ATOM 1829 C SER R 324 120.612 105.781 120.541 1.00 0.00 C**

**ATOM 1830 O SER R 324 121.115 106.208 119.496 1.00 0.00 O**

**ATOM 1831 N SER R 324 119.021 106.179 122.398 1.00 0.00 N**

**ATOM 1832 CB SER R 324 121.235 107.218 122.489 1.00 0.00 C**

**ATOM 1833 OG SER R 324 122.312 107.738 121.731 1.00 0.00 O**

**ATOM 1834 CA TRP R 124 115.748 122.176 140.594 1.00 0.00 C**

**ATOM 1835 C TRP R 124 115.523 123.682 140.677 1.00 0.00 C**

**ATOM 1836 O TRP R 124 114.540 124.129 141.288 1.00 0.00 O**

**ATOM 1837 N TRP R 124 116.414 121.726 141.814 1.00 0.00 N**

**ATOM 1838 CB TRP R 124 114.419 121.440 140.393 1.00 0.00 C**

**ATOM 1839 CG TRP R 124 113.661 121.855 139.157 1.00 0.00 C**

**ATOM 1840 CD1 TRP R 124 112.717 122.839 139.067 1.00 0.00 C**

**ATOM 1841 NE1 TRP R 124 112.251 122.933 137.779 1.00 0.00 N**

**ATOM 1842 CE2 TRP R 124 112.893 122.003 137.005 1.00 0.00 C**

**ATOM 1843 CD2 TRP R 124 113.791 121.305 137.839 1.00 0.00 C**

**ATOM 1844 CE3 TRP R 124 114.577 120.292 137.283 1.00 0.00 C**

**ATOM 1845 CZ3 TRP R 124 114.443 120.011 135.934 1.00 0.00 C**

**ATOM 1846 CH2 TRP R 124 113.542 120.722 135.132 1.00 0.00 C**

**ATOM 1847 CZ2 TRP R 124 112.761 121.719 135.647 1.00 0.00 C**

**ATOM 1848 CA TRP R 183 99.648 109.040 120.131 1.00 0.00 C**

**ATOM 1849 C TRP R 183 98.480 109.446 121.035 1.00 0.00 C**

**ATOM 1850 O TRP R 183 98.697 110.012 122.113 1.00 0.00 O**

**ATOM 1851 N TRP R 183 99.424 107.728 119.521 1.00 0.00 N**

**ATOM 1852 CB TRP R 183 99.941 110.075 119.038 1.00 0.00 C**

**ATOM 1853 CG TRP R 183 101.382 109.991 118.498 1.00 0.00 C**

**ATOM 1854 CD1 TRP R 183 101.856 109.100 117.580 1.00 0.00 C**

**ATOM 1855 NE1 TRP R 183 103.181 109.329 117.322 1.00 0.00 N**

**ATOM 1856 CE2 TRP R 183 103.595 110.407 118.056 1.00 0.00 C**

**ATOM 1857 CD2 TRP R 183 102.488 110.858 118.805 1.00 0.00 C**

**ATOM 1858 CE3 TRP R 183 102.653 111.960 119.644 1.00 0.00 C**

**ATOM 1859 CZ3 TRP R 183 103.898 112.567 119.709 1.00 0.00 C**

**ATOM 1860 CH2 TRP R 183 104.975 112.094 118.956 1.00 0.00 C**

**ATOM 1861 CZ2 TRP R 183 104.845 111.018 118.125 1.00 0.00 C**

**ATOM 1862 CA TRP R 221 100.011 103.360 148.049 1.00 0.00 C**

**ATOM 1863 C TRP R 221 99.698 103.728 146.607 1.00 0.00 C**

**ATOM 1864 O TRP R 221 99.825 102.887 145.712 1.00 0.00 O**

**ATOM 1865 N TRP R 221 100.857 104.371 148.670 1.00 0.00 N**

**ATOM 1866 CB TRP R 221 98.719 103.173 148.845 1.00 0.00 C**

**ATOM 1867 CG TRP R 221 97.843 102.105 148.269 1.00 0.00 C**

**ATOM 1868 CD1 TRP R 221 98.099 100.765 148.237 1.00 0.00 C**

**ATOM 1869 NE1 TRP R 221 97.070 100.100 147.618 1.00 0.00 N**

**ATOM 1870 CE2 TRP R 221 96.125 101.012 147.226 1.00 0.00 C**

**ATOM 1871 CD2 TRP R 221 96.581 102.287 147.616 1.00 0.00 C**

**ATOM 1872 CE3 TRP R 221 95.789 103.403 147.330 1.00 0.00 C**

**ATOM 1873 CZ3 TRP R 221 94.586 103.212 146.673 1.00 0.00 C**

**ATOM 1874 CH2 TRP R 221 94.160 101.932 146.298 1.00 0.00 C**

**ATOM 1875 CZ2 TRP R 221 94.913 100.822 146.565 1.00 0.00 C**

**ATOM 1876 CA TRP R 222 98.950 105.381 145.003 1.00 0.00 C**

**ATOM 1877 C TRP R 222 100.211 105.498 144.153 1.00 0.00 C**

**ATOM 1878 O TRP R 222 100.157 105.241 142.943 1.00 0.00 O**

**ATOM 1879 N TRP R 222 99.256 104.966 146.366 1.00 0.00 N**

**ATOM 1880 CB TRP R 222 98.209 106.719 145.021 1.00 0.00 C**

**ATOM 1881 CG TRP R 222 96.746 106.609 145.335 1.00 0.00 C**

**ATOM 1882 CD1 TRP R 222 96.152 106.802 146.549 1.00 0.00 C**

**ATOM 1883 NE1 TRP R 222 94.794 106.626 146.446 1.00 0.00 N**

**ATOM 1884 CE2 TRP R 222 94.484 106.327 145.145 1.00 0.00 C**

**ATOM 1885 CD2 TRP R 222 95.690 106.312 144.415 1.00 0.00 C**

**ATOM 1886 CE3 TRP R 222 95.647 106.028 143.047 1.00 0.00 C**

**ATOM 1887 CZ3 TRP R 222 94.421 105.772 142.460 1.00 0.00 C**

**ATOM 1888 CH2 TRP R 222 93.239 105.793 143.212 1.00 0.00 C**

**ATOM 1889 CZ2 TRP R 222 93.250 106.068 144.552 1.00 0.00 C**

**ATOM 1890 CA TRP R 287 114.970 98.143 130.122 1.00 0.00 C**

**ATOM 1891 C TRP R 287 114.832 97.318 131.397 1.00 0.00 C**

**ATOM 1892 O TRP R 287 114.345 97.852 132.399 1.00 0.00 O**

**ATOM 1893 N TRP R 287 116.118 97.792 129.287 1.00 0.00 N**

**ATOM 1894 CB TRP R 287 113.664 98.041 129.331 1.00 0.00 C**

**ATOM 1895 CG TRP R 287 113.465 99.141 128.343 1.00 0.00 C**

**ATOM 1896 CD1 TRP R 287 113.632 99.074 126.995 1.00 0.00 C**

**ATOM 1897 NE1 TRP R 287 113.347 100.289 126.422 1.00 0.00 N**

**ATOM 1898 CE2 TRP R 287 112.999 101.175 127.406 1.00 0.00 C**

**ATOM 1899 CD2 TRP R 287 113.060 100.486 128.632 1.00 0.00 C**

**ATOM 1900 CE3 TRP R 287 112.742 101.172 129.808 1.00 0.00 C**

**ATOM 1901 CZ3 TRP R 287 112.381 102.505 129.722 1.00 0.00 C**

**ATOM 1902 CH2 TRP R 287 112.330 103.162 128.486 1.00 0.00 C**

**ATOM 1903 CZ2 TRP R 287 112.634 102.516 127.320 1.00 0.00 C**

**ATOM 1904 CA LEU R 74 120.390 113.999 119.194 1.00 0.00 C**

**ATOM 1905 C LEU R 74 120.835 114.087 117.737 1.00 0.00 C**

**ATOM 1906 O LEU R 74 120.010 114.352 116.854 1.00 0.00 O**

**ATOM 1907 N LEU R 74 120.962 112.830 119.853 1.00 0.00 N**

**ATOM 1908 CB LEU R 74 120.773 115.270 119.952 1.00 0.00 C**

**ATOM 1909 CG LEU R 74 119.981 116.527 119.584 1.00 0.00 C**

**ATOM 1910 CD1 LEU R 74 118.517 116.366 119.967 1.00 0.00 C**

**ATOM 1911 CD2 LEU R 74 120.580 117.760 120.242 1.00 0.00 C**

**ATOM 1912 CA LEU R 79 119.863 112.977 110.728 1.00 0.00 C**

**ATOM 1913 C LEU R 79 118.753 112.099 110.159 1.00 0.00 C**

**ATOM 1914 O LEU R 79 118.365 112.263 108.993 1.00 0.00 O**

**ATOM 1915 N LEU R 79 119.541 113.446 112.073 1.00 0.00 N**

**ATOM 1916 CB LEU R 79 121.191 112.220 110.741 1.00 0.00 C**

**ATOM 1917 CG LEU R 79 121.734 111.767 109.383 1.00 0.00 C**

**ATOM 1918 CD1 LEU R 79 121.805 112.934 108.408 1.00 0.00 C**

**ATOM 1919 CD2 LEU R 79 123.099 111.112 109.538 1.00 0.00 C**

**ATOM 1920 CA LEU R 101 110.024 106.464 116.339 1.00 0.00 C**

**ATOM 1921 C LEU R 101 111.160 107.464 116.529 1.00 0.00 C**

**ATOM 1922 O LEU R 101 111.595 107.712 117.661 1.00 0.00 O**

**ATOM 1923 N LEU R 101 109.055 106.939 115.355 1.00 0.00 N**

**ATOM 1924 CB LEU R 101 110.568 105.099 115.919 1.00 0.00 C**

**ATOM 1925 CG LEU R 101 111.587 104.468 116.867 1.00 0.00 C**

**ATOM 1926 CD1 LEU R 101 110.920 104.104 118.182 1.00 0.00 C**

**ATOM 1927 CD2 LEU R 101 112.228 103.247 116.228 1.00 0.00 C**

**ATOM 1928 CA LEU R 103 110.408 111.829 116.670 1.00 0.00 C**

**ATOM 1929 C LEU R 103 110.268 111.558 118.165 1.00 0.00 C**

**ATOM 1930 O LEU R 103 110.580 112.430 118.993 1.00 0.00 O**

**ATOM 1931 N LEU R 103 110.977 110.678 115.968 1.00 0.00 N**

**ATOM 1932 CB LEU R 103 109.052 112.193 116.064 1.00 0.00 C**

**ATOM 1933 CG LEU R 103 108.284 113.336 116.732 1.00 0.00 C**

**ATOM 1934 CD1 LEU R 103 109.066 114.634 116.641 1.00 0.00 C**

**ATOM 1935 CD2 LEU R 103 106.907 113.490 116.108 1.00 0.00 C**

**ATOM 1936 CA LEU R 107 111.733 114.189 122.252 1.00 0.00 C**

**ATOM 1937 C LEU R 107 112.044 113.551 123.602 1.00 0.00 C**

**ATOM 1938 O LEU R 107 111.878 114.209 124.635 1.00 0.00 O**

**ATOM 1939 N LEU R 107 112.364 113.488 121.144 1.00 0.00 N**

**ATOM 1940 CB LEU R 107 110.221 114.255 122.038 1.00 0.00 C**

**ATOM 1941 CG LEU R 107 109.762 115.113 120.858 1.00 0.00 C**

**ATOM 1942 CD1 LEU R 107 108.298 114.861 120.533 1.00 0.00 C**

**ATOM 1943 CD2 LEU R 107 110.002 116.585 121.154 1.00 0.00 C**

**ATOM 1944 CA LEU R 120 124.702 116.907 140.113 1.00 0.00 C**

**ATOM 1945 C LEU R 120 124.405 118.026 141.108 1.00 0.00 C**

**ATOM 1946 O LEU R 120 125.005 118.078 142.188 1.00 0.00 O**

**ATOM 1947 N LEU R 120 123.492 116.239 139.632 1.00 0.00 N**

**ATOM 1948 CB LEU R 120 125.500 117.454 138.928 1.00 0.00 C**

**ATOM 1949 CG LEU R 120 126.069 116.424 137.955 1.00 0.00 C**

**ATOM 1950 CD1 LEU R 120 126.609 117.105 136.710 1.00 0.00 C**

**ATOM 1951 CD2 LEU R 120 127.166 115.625 138.638 1.00 0.00 C**

**ATOM 1952 CA LEU R 130 109.329 123.677 132.804 1.00 0.00 C**

**ATOM 1953 C LEU R 130 109.299 122.272 133.394 1.00 0.00 C**

**ATOM 1954 O LEU R 130 109.444 121.288 132.660 1.00 0.00 O**

**ATOM 1955 N LEU R 130 108.308 124.525 133.409 1.00 0.00 N**

**ATOM 1956 CB LEU R 130 110.708 124.311 132.981 1.00 0.00 C**

**ATOM 1957 CG LEU R 130 111.870 123.607 132.281 1.00 0.00 C**

**ATOM 1958 CD1 LEU R 130 111.681 123.641 130.772 1.00 0.00 C**

**ATOM 1959 CD2 LEU R 130 113.191 124.249 132.669 1.00 0.00 C**

**ATOM 1960 CA LEU R 149 108.428 99.577 118.731 1.00 0.00 C**

**ATOM 1961 C LEU R 149 107.566 98.317 118.713 1.00 0.00 C**

**ATOM 1962 O LEU R 149 107.783 97.408 117.892 1.00 0.00 O**

**ATOM 1963 N LEU R 149 107.616 100.785 118.902 1.00 0.00 N**

**ATOM 1964 CB LEU R 149 109.462 99.478 119.853 1.00 0.00 C**

**ATOM 1965 CG LEU R 149 110.585 100.513 119.917 1.00 0.00 C**

**ATOM 1966 CD1 LEU R 149 111.403 100.335 121.188 1.00 0.00 C**

**ATOM 1967 CD2 LEU R 149 111.462 100.433 118.694 1.00 0.00 C**

**ATOM 1968 CA LEU R 167 97.879 93.327 101.480 1.00 0.00 C**

**ATOM 1969 C LEU R 167 96.777 94.249 101.990 1.00 0.00 C**

**ATOM 1970 O LEU R 167 96.631 95.375 101.501 1.00 0.00 O**

**ATOM 1971 N LEU R 167 98.409 92.487 102.551 1.00 0.00 N**

**ATOM 1972 CB LEU R 167 97.358 92.454 100.338 1.00 0.00 C**

**ATOM 1973 CG LEU R 167 98.383 91.550 99.648 1.00 0.00 C**

**ATOM 1974 CD1 LEU R 167 97.687 90.517 98.776 1.00 0.00 C**

**ATOM 1975 CD2 LEU R 167 99.361 92.375 98.826 1.00 0.00 C**

**ATOM 1976 CA LEU R 173 96.437 105.339 105.463 1.00 0.00 C**

**ATOM 1977 C LEU R 173 96.194 104.624 106.788 1.00 0.00 C**

**ATOM 1978 O LEU R 173 96.044 105.267 107.835 1.00 0.00 O**

**ATOM 1979 N LEU R 173 97.565 104.753 104.741 1.00 0.00 N**

**ATOM 1980 CB LEU R 173 95.182 105.296 104.590 1.00 0.00 C**

**ATOM 1981 CG LEU R 173 93.905 105.859 105.215 1.00 0.00 C**

**ATOM 1982 CD1 LEU R 173 94.020 107.362 105.406 1.00 0.00 C**

**ATOM 1983 CD2 LEU R 173 92.690 105.510 104.368 1.00 0.00 C**

**ATOM 1984 CA LEU R 184 96.072 109.416 121.468 1.00 0.00 C**

**ATOM 1985 C LEU R 184 96.096 108.559 122.732 1.00 0.00 C**

**ATOM 1986 O LEU R 184 95.710 109.030 123.808 1.00 0.00 O**

**ATOM 1987 N LEU R 184 97.237 109.169 120.614 1.00 0.00 N**

**ATOM 1988 CB LEU R 184 94.769 109.193 120.697 1.00 0.00 C**

**ATOM 1989 CG LEU R 184 94.407 110.220 119.621 1.00 0.00 C**

**ATOM 1990 CD1 LEU R 184 93.265 109.715 118.753 1.00 0.00 C**

**ATOM 1991 CD2 LEU R 184 94.053 111.557 120.255 1.00 0.00 C**

**ATOM 1992 CA LEU R 185 96.574 106.400 123.773 1.00 0.00 C**

**ATOM 1993 C LEU R 185 97.635 106.904 124.746 1.00 0.00 C**

**ATOM 1994 O LEU R 185 97.478 106.767 125.965 1.00 0.00 O**

**ATOM 1995 N LEU R 185 96.457 107.274 122.605 1.00 0.00 N**

**ATOM 1996 CB LEU R 185 96.888 104.971 123.324 1.00 0.00 C**

**ATOM 1997 CG LEU R 185 96.969 103.885 124.399 1.00 0.00 C**

**ATOM 1998 CD1 LEU R 185 95.624 103.719 125.090 1.00 0.00 C**

**ATOM 1999 CD2 LEU R 185 97.435 102.568 123.803 1.00 0.00 C**

**ATOM 2000 CA LEU R 196 99.565 115.896 138.422 1.00 0.00 C**

**ATOM 2001 C LEU R 196 101.001 116.380 138.589 1.00 0.00 C**

**ATOM 2002 O LEU R 196 101.462 116.512 139.730 1.00 0.00 O**

**ATOM 2003 N LEU R 196 98.952 116.292 137.158 1.00 0.00 N**

**ATOM 2004 CB LEU R 196 99.520 114.373 138.569 1.00 0.00 C**

**ATOM 2005 CG LEU R 196 98.122 113.771 138.726 1.00 0.00 C**

**ATOM 2006 CD1 LEU R 196 98.156 112.262 138.546 1.00 0.00 C**

**ATOM 2007 CD2 LEU R 196 97.528 114.140 140.078 1.00 0.00 C**

**ATOM 2008 CA LEU R 212 104.239 113.850 140.519 1.00 0.00 C**

**ATOM 2009 C LEU R 212 103.552 113.900 141.882 1.00 0.00 C**

**ATOM 2010 O LEU R 212 103.598 112.933 142.646 1.00 0.00 O**

**ATOM 2011 N LEU R 212 105.670 114.146 140.630 1.00 0.00 N**

**ATOM 2012 CB LEU R 212 104.048 112.486 139.865 1.00 0.00 C**

**ATOM 2013 CG LEU R 212 104.466 112.406 138.395 1.00 0.00 C**

**ATOM 2014 CD1 LEU R 212 104.399 110.975 137.895 1.00 0.00 C**

**ATOM 2015 CD2 LEU R 212 103.600 113.315 137.538 1.00 0.00 C**

**ATOM 2016 CA LEU R 224 103.194 102.003 144.275 1.00 0.00 C**

**ATOM 2017 C LEU R 224 102.251 101.481 143.196 1.00 0.00 C**

**ATOM 2018 O LEU R 224 102.682 100.760 142.290 1.00 0.00 O**

**ATOM 2019 N LEU R 224 102.914 103.400 144.592 1.00 0.00 N**

**ATOM 2020 CB LEU R 224 103.093 101.150 145.539 1.00 0.00 C**

**ATOM 2021 CG LEU R 224 103.348 99.650 145.382 1.00 0.00 C**

**ATOM 2022 CD1 LEU R 224 104.808 99.386 145.049 1.00 0.00 C**

**ATOM 2023 CD2 LEU R 224 102.936 98.904 146.639 1.00 0.00 C**

**ATOM 2024 CA LEU R 240 105.933 89.204 125.634 1.00 0.00 C**

**ATOM 2025 C LEU R 240 105.443 89.266 124.188 1.00 0.00 C**

**ATOM 2026 O LEU R 240 105.563 88.277 123.452 1.00 0.00 O**

**ATOM 2027 N LEU R 240 106.606 90.439 126.044 1.00 0.00 N**

**ATOM 2028 CB LEU R 240 104.772 88.887 126.578 1.00 0.00 C**

**ATOM 2029 CG LEU R 240 105.136 88.474 128.006 1.00 0.00 C**

**ATOM 2030 CD1 LEU R 240 103.886 88.347 128.863 1.00 0.00 C**

**ATOM 2031 CD2 LEU R 240 105.921 87.172 128.004 1.00 0.00 C**

**ATOM 2032 CA LEU R 248 106.826 82.862 115.424 1.00 0.00 C**

**ATOM 2033 C LEU R 248 107.295 83.312 114.045 1.00 0.00 C**

**ATOM 2034 O LEU R 248 107.153 82.560 113.075 1.00 0.00 O**

**ATOM 2035 N LEU R 248 107.659 83.416 116.488 1.00 0.00 N**

**ATOM 2036 CB LEU R 248 105.361 83.239 115.639 1.00 0.00 C**

**ATOM 2037 CG LEU R 248 104.677 82.633 116.868 1.00 0.00 C**

**ATOM 2038 CD1 LEU R 248 103.256 83.156 116.999 1.00 0.00 C**

**ATOM 2039 CD2 LEU R 248 104.697 81.115 116.813 1.00 0.00 C**

**ATOM 2040 CA LEU R 251 109.799 79.710 113.018 1.00 0.00 C**

**ATOM 2041 C LEU R 251 109.098 79.628 111.663 1.00 0.00 C**

**ATOM 2042 O LEU R 251 109.185 78.602 110.977 1.00 0.00 O**

**ATOM 2043 N LEU R 251 110.346 81.045 113.281 1.00 0.00 N**

**ATOM 2044 CB LEU R 251 108.845 79.294 114.138 1.00 0.00 C**

**ATOM 2045 CG LEU R 251 109.478 78.977 115.495 1.00 0.00 C**

**ATOM 2046 CD1 LEU R 251 108.407 78.706 116.542 1.00 0.00 C**

**ATOM 2047 CD2 LEU R 251 110.434 77.801 115.379 1.00 0.00 C**

**ATOM 2048 CA LEU R 253 110.865 81.803 108.003 1.00 0.00 C**

**ATOM 2049 C LEU R 253 111.702 80.542 107.815 1.00 0.00 C**

**ATOM 2050 O LEU R 253 112.239 80.324 106.722 1.00 0.00 O**

**ATOM 2051 N LEU R 253 109.827 81.640 109.023 1.00 0.00 N**

**ATOM 2052 CB LEU R 253 111.775 82.988 108.331 1.00 0.00 C**

**ATOM 2053 CG LEU R 253 111.194 84.403 108.278 1.00 0.00 C**

**ATOM 2054 CD1 LEU R 253 112.170 85.408 108.869 1.00 0.00 C**

**ATOM 2055 CD2 LEU R 253 110.836 84.780 106.851 1.00 0.00 C**

**ATOM 2056 CA LEU R 258 113.353 80.205 99.217 1.00 0.00 C**

**ATOM 2057 C LEU R 258 114.550 80.418 100.135 1.00 0.00 C**

**ATOM 2058 O LEU R 258 115.693 80.477 99.667 1.00 0.00 O**

**ATOM 2059 N LEU R 258 112.390 79.256 99.778 1.00 0.00 N**

**ATOM 2060 CB LEU R 258 112.675 81.547 98.936 1.00 0.00 C**

**ATOM 2061 CG LEU R 258 111.577 81.624 97.881 1.00 0.00 C**

**ATOM 2062 CD1 LEU R 258 110.914 82.987 97.907 1.00 0.00 C**

**ATOM 2063 CD2 LEU R 258 112.220 81.397 96.532 1.00 0.00 C**

**ATOM 2064 CA LEU R 259 115.366 80.830 102.401 1.00 0.00 C**

**ATOM 2065 C LEU R 259 116.336 79.659 102.523 1.00 0.00 C**

**ATOM 2066 O LEU R 259 117.550 79.863 102.640 1.00 0.00 O**

**ATOM 2067 N LEU R 259 114.299 80.557 101.440 1.00 0.00 N**

**ATOM 2068 CB LEU R 259 114.756 81.194 103.750 1.00 0.00 C**

**ATOM 2069 CG LEU R 259 114.049 82.553 103.706 1.00 0.00 C**

**ATOM 2070 CD1 LEU R 259 113.555 82.937 105.073 1.00 0.00 C**

**ATOM 2071 CD2 LEU R 259 114.956 83.646 103.146 1.00 0.00 C**

**ATOM 2072 CA LEU R 269 118.413 84.516 106.769 1.00 0.00 C**

**ATOM 2073 C LEU R 269 119.106 84.577 108.126 1.00 0.00 C**

**ATOM 2074 O LEU R 269 118.526 85.050 109.111 1.00 0.00 O**

**ATOM 2075 N LEU R 269 119.334 84.867 105.688 1.00 0.00 N**

**ATOM 2076 CB LEU R 269 117.821 83.127 106.524 1.00 0.00 C**

**ATOM 2077 CG LEU R 269 116.931 82.521 107.617 1.00 0.00 C**

**ATOM 2078 CD1 LEU R 269 115.781 83.446 107.998 1.00 0.00 C**

**ATOM 2079 CD2 LEU R 269 116.403 81.164 107.177 1.00 0.00 C**

**ATOM 2080 CA LEU R 275 119.503 91.375 113.614 1.00 0.00 C**

**ATOM 2081 C LEU R 275 118.369 91.359 114.636 1.00 0.00 C**

**ATOM 2082 O LEU R 275 118.296 92.235 115.512 1.00 0.00 O**

**ATOM 2083 N LEU R 275 120.085 90.044 113.424 1.00 0.00 N**

**ATOM 2084 CB LEU R 275 119.002 91.913 112.273 1.00 0.00 C**

**ATOM 2085 CG LEU R 275 118.496 93.354 112.262 1.00 0.00 C**

**ATOM 2086 CD1 LEU R 275 119.613 94.307 112.630 1.00 0.00 C**

**ATOM 2087 CD2 LEU R 275 117.909 93.705 110.903 1.00 0.00 C**

**ATOM 2088 CA LEU R 277 118.451 88.778 118.320 1.00 0.00 C**

**ATOM 2089 C LEU R 277 119.114 90.016 118.921 1.00 0.00 C**

**ATOM 2090 O LEU R 277 119.003 90.250 120.129 1.00 0.00 O**

**ATOM 2091 N LEU R 277 117.820 89.055 117.027 1.00 0.00 N**

**ATOM 2092 CB LEU R 277 119.466 87.642 118.189 1.00 0.00 C**

**ATOM 2093 CG LEU R 277 118.921 86.251 117.858 1.00 0.00 C**

**ATOM 2094 CD1 LEU R 277 120.061 85.270 117.629 1.00 0.00 C**

**ATOM 2095 CD2 LEU R 277 117.996 85.758 118.959 1.00 0.00 C**

**ATOM 2096 CA LEU R 295 110.323 97.887 141.777 1.00 0.00 C**

**ATOM 2097 C LEU R 295 110.834 97.441 143.148 1.00 0.00 C**

**ATOM 2098 O LEU R 295 110.083 97.476 144.134 1.00 0.00 O**

**ATOM 2099 N LEU R 295 111.294 98.723 141.062 1.00 0.00 N**

**ATOM 2100 CB LEU R 295 109.949 96.664 140.945 1.00 0.00 C**

**ATOM 2101 CG LEU R 295 109.034 96.891 139.748 1.00 0.00 C**

**ATOM 2102 CD1 LEU R 295 108.796 95.570 139.061 1.00 0.00 C**

**ATOM 2103 CD2 LEU R 295 107.728 97.528 140.179 1.00 0.00 C**

**ATOM 2104 CA LEU R 299 108.390 97.648 147.687 1.00 0.00 C**

**ATOM 2105 C LEU R 299 109.328 96.855 148.586 1.00 0.00 C**

**ATOM 2106 O LEU R 299 109.072 96.769 149.794 1.00 0.00 O**

**ATOM 2107 N LEU R 299 109.066 98.522 146.732 1.00 0.00 N**

**ATOM 2108 CB LEU R 299 107.421 96.693 146.957 1.00 0.00 C**

**ATOM 2109 CG LEU R 299 107.851 95.773 145.811 1.00 0.00 C**

**ATOM 2110 CD1 LEU R 299 108.129 94.358 146.301 1.00 0.00 C**

**ATOM 2111 CD2 LEU R 299 106.804 95.769 144.707 1.00 0.00 C**

**ATOM 2112 CA LEU R 309 122.730 106.298 143.574 1.00 0.00 C**

**ATOM 2113 C LEU R 309 123.404 106.352 142.208 1.00 0.00 C**

**ATOM 2114 O LEU R 309 122.754 106.650 141.191 1.00 0.00 O**

**ATOM 2115 N LEU R 309 123.192 105.140 144.334 1.00 0.00 N**

**ATOM 2116 CB LEU R 309 123.030 107.572 144.366 1.00 0.00 C**

**ATOM 2117 CG LEU R 309 122.528 108.940 143.903 1.00 0.00 C**

**ATOM 2118 CD1 LEU R 309 121.023 109.012 143.977 1.00 0.00 C**

**ATOM 2119 CD2 LEU R 309 123.163 110.047 144.732 1.00 0.00 C**

**ATOM 2120 CA LEU R 318 121.231 102.768 129.957 1.00 0.00 C**

**ATOM 2121 C LEU R 318 119.856 102.783 129.291 1.00 0.00 C**

**ATOM 2122 O LEU R 318 119.729 102.464 128.096 1.00 0.00 O**

**ATOM 2123 N LEU R 318 121.404 103.896 130.877 1.00 0.00 N**

**ATOM 2124 CB LEU R 318 121.444 101.446 130.693 1.00 0.00 C**

**ATOM 2125 CG LEU R 318 122.869 101.096 131.120 1.00 0.00 C**

**ATOM 2126 CD1 LEU R 318 122.858 99.882 132.034 1.00 0.00 C**

**ATOM 2127 CD2 LEU R 318 123.755 100.856 129.908 1.00 0.00 C**

**ATOM 2128 CA LEU R 325 120.948 103.443 119.878 1.00 0.00 C**

**ATOM 2129 C LEU R 325 119.889 103.057 118.856 1.00 0.00 C**

**ATOM 2130 O LEU R 325 120.242 102.460 117.832 1.00 0.00 O**

**ATOM 2131 N LEU R 325 120.505 104.479 120.805 1.00 0.00 N**

**ATOM 2132 CB LEU R 325 121.390 102.183 120.632 1.00 0.00 C**

**ATOM 2133 CG LEU R 325 122.659 102.260 121.484 1.00 0.00 C**

**ATOM 2134 CD1 LEU R 325 122.857 100.970 122.265 1.00 0.00 C**

**ATOM 2135 CD2 LEU R 325 123.869 102.557 120.615 1.00 0.00 C**

**ATOM 2136 CA LEU R 329 119.573 99.744 115.143 1.00 0.00 C**

**ATOM 2137 C LEU R 329 118.446 99.657 114.121 1.00 0.00 C**

**ATOM 2138 O LEU R 329 118.235 98.590 113.532 1.00 0.00 O**

**ATOM 2139 N LEU R 329 120.139 101.086 115.229 1.00 0.00 N**

**ATOM 2140 CB LEU R 329 119.074 99.297 116.516 1.00 0.00 C**

**ATOM 2141 CG LEU R 329 120.153 99.037 117.566 1.00 0.00 C**

**ATOM 2142 CD1 LEU R 329 119.518 98.754 118.914 1.00 0.00 C**

**ATOM 2143 CD2 LEU R 329 121.046 97.883 117.139 1.00 0.00 C**

**ATOM 2144 CA LEU R 333 120.570 98.721 109.186 1.00 0.00 C**

**ATOM 2145 C LEU R 333 119.486 98.164 108.273 1.00 0.00 C**

**ATOM 2146 O LEU R 333 119.591 97.001 107.868 1.00 0.00 O**

**ATOM 2147 N LEU R 333 120.424 100.142 109.463 1.00 0.00 N**

**ATOM 2148 CB LEU R 333 120.590 97.919 110.498 1.00 0.00 C**

**ATOM 2149 CG LEU R 333 121.870 98.061 111.329 1.00 0.00 C**

**ATOM 2150 CD1 LEU R 333 121.753 97.352 112.665 1.00 0.00 C**

**ATOM 2151 CD2 LEU R 333 123.070 97.542 110.551 1.00 0.00 C**

**ATOM 2152 CA ILE R 58 133.042 109.224 139.321 1.00 0.00 C**

**ATOM 2153 C ILE R 58 132.005 110.216 138.783 1.00 0.00 C**

**ATOM 2154 O ILE R 58 131.389 109.899 137.751 1.00 0.00 O**

**ATOM 2155 N ILE R 58 134.315 109.811 139.760 1.00 0.00 N**

**ATOM 2156 CB ILE R 58 132.504 108.262 140.394 1.00 0.00 C**

**ATOM 2157 CG2 ILE R 58 132.498 108.820 141.826 1.00 0.00 C**

**ATOM 2158 CG1 ILE R 58 131.192 107.581 139.973 1.00 0.00 C**

**ATOM 2159 CD1 ILE R 58 130.795 106.430 140.870 1.00 0.00 C**

**ATOM 2160 CA ILE R 61 132.842 110.122 134.062 1.00 0.00 C**

**ATOM 2161 C ILE R 61 131.346 109.878 133.836 1.00 0.00 C**

**ATOM 2162 O ILE R 61 130.929 109.616 132.703 1.00 0.00 O**

**ATOM 2163 N ILE R 61 133.062 111.181 135.047 1.00 0.00 N**

**ATOM 2164 CB ILE R 61 133.623 108.835 134.418 1.00 0.00 C**

**ATOM 2165 CG2 ILE R 61 135.114 109.131 134.512 1.00 0.00 C**

**ATOM 2166 CG1 ILE R 61 133.143 108.158 135.701 1.00 0.00 C**

**ATOM 2167 CD1 ILE R 61 133.741 106.786 135.927 1.00 0.00 C**

**ATOM 2168 CA ILE R 62 129.073 109.771 134.725 1.00 0.00 C**

**ATOM 2169 C ILE R 62 128.463 110.875 133.858 1.00 0.00 C**

**ATOM 2170 O ILE R 62 127.704 110.593 132.917 1.00 0.00 O**

**ATOM 2171 N ILE R 62 130.519 109.964 134.892 1.00 0.00 N**

**ATOM 2172 CB ILE R 62 128.373 109.656 136.094 1.00 0.00 C**

**ATOM 2173 CG2 ILE R 62 126.860 109.747 135.958 1.00 0.00 C**

**ATOM 2174 CG1 ILE R 62 128.764 108.347 136.786 1.00 0.00 C**

**ATOM 2175 CD1 ILE R 62 128.392 107.105 136.001 1.00 0.00 C**

**ATOM 2176 CA ILE R 84 112.658 111.209 106.178 1.00 0.00 C**

**ATOM 2177 C ILE R 84 111.903 112.455 105.696 1.00 0.00 C**

**ATOM 2178 O ILE R 84 111.008 112.357 104.847 1.00 0.00 O**

**ATOM 2179 N ILE R 84 114.071 111.513 106.432 1.00 0.00 N**

**ATOM 2180 CB ILE R 84 111.999 110.562 107.414 1.00 0.00 C**

**ATOM 2181 CG2 ILE R 84 110.579 110.105 107.110 1.00 0.00 C**

**ATOM 2182 CG1 ILE R 84 112.783 109.325 107.855 1.00 0.00 C**

**ATOM 2183 CD1 ILE R 84 112.857 108.228 106.811 1.00 0.00 C**

**ATOM 2184 CA ILE R 85 111.583 114.878 105.804 1.00 0.00 C**

**ATOM 2185 C ILE R 85 111.874 115.194 104.336 1.00 0.00 C**

**ATOM 2186 O ILE R 85 110.959 115.533 103.574 1.00 0.00 O**

**ATOM 2187 N ILE R 85 112.239 113.637 106.230 1.00 0.00 N**

**ATOM 2188 CB ILE R 85 111.973 116.047 106.736 1.00 0.00 C**

**ATOM 2189 CG2 ILE R 85 111.696 117.410 106.099 1.00 0.00 C**

**ATOM 2190 CG1 ILE R 85 111.243 115.936 108.078 1.00 0.00 C**

**ATOM 2191 CD1 ILE R 85 111.607 117.029 109.059 1.00 0.00 C**

**ATOM 2192 CA ILE R 96 106.181 106.383 108.668 1.00 0.00 C**

**ATOM 2193 C ILE R 96 106.460 105.940 110.105 1.00 0.00 C**

**ATOM 2194 O ILE R 96 106.549 106.769 111.025 1.00 0.00 O**

**ATOM 2195 N ILE R 96 107.056 105.680 107.729 1.00 0.00 N**

**ATOM 2196 CB ILE R 96 104.705 106.175 108.277 1.00 0.00 C**

**ATOM 2197 CG2 ILE R 96 103.769 106.752 109.323 1.00 0.00 C**

**ATOM 2198 CG1 ILE R 96 104.418 106.842 106.931 1.00 0.00 C**

**ATOM 2199 CD1 ILE R 96 103.050 106.523 106.369 1.00 0.00 C**

**ATOM 2200 CA ILE R 98 110.659 105.044 111.549 1.00 0.00 C**

**ATOM 2201 C ILE R 98 110.608 106.540 111.865 1.00 0.00 C**

**ATOM 2202 O ILE R 98 111.309 107.020 112.765 1.00 0.00 O**

**ATOM 2203 N ILE R 98 109.322 104.528 111.230 1.00 0.00 N**

**ATOM 2204 CB ILE R 98 111.653 104.718 110.413 1.00 0.00 C**

**ATOM 2205 CG2 ILE R 98 113.049 105.250 110.708 1.00 0.00 C**

**ATOM 2206 CG1 ILE R 98 111.751 103.206 110.206 1.00 0.00 C**

**ATOM 2207 CD1 ILE R 98 112.533 102.805 108.975 1.00 0.00 C**

**ATOM 2208 CA ILE R 133 106.319 120.125 130.410 1.00 0.00 C**

**ATOM 2209 C ILE R 133 107.354 119.010 130.243 1.00 0.00 C**

**ATOM 2210 O ILE R 133 107.138 118.085 129.454 1.00 0.00 O**

**ATOM 2211 N ILE R 133 106.158 120.485 131.820 1.00 0.00 N**

**ATOM 2212 CB ILE R 133 106.602 121.356 129.516 1.00 0.00 C**

**ATOM 2213 CG2 ILE R 133 105.424 122.327 129.566 1.00 0.00 C**

**ATOM 2214 CG1 ILE R 133 107.908 122.083 129.837 1.00 0.00 C**

**ATOM 2215 CD1 ILE R 133 108.239 123.200 128.869 1.00 0.00 C**

**ATOM 2216 CA ILE R 135 107.676 115.429 132.873 1.00 0.00 C**

**ATOM 2217 C ILE R 135 106.692 114.858 131.853 1.00 0.00 C**

**ATOM 2218 O ILE R 135 106.733 113.657 131.531 1.00 0.00 O**

**ATOM 2219 N ILE R 135 108.261 116.687 132.392 1.00 0.00 N**

**ATOM 2220 CB ILE R 135 107.008 115.625 134.248 1.00 0.00 C**

**ATOM 2221 CG2 ILE R 135 106.112 114.447 134.616 1.00 0.00 C**

**ATOM 2222 CG1 ILE R 135 108.049 115.838 135.345 1.00 0.00 C**

**ATOM 2223 CD1 ILE R 135 107.430 116.205 136.673 1.00 0.00 C**

**ATOM 2224 CA ILE R 137 107.114 115.003 127.337 1.00 0.00 C**

**ATOM 2225 C ILE R 137 107.863 113.697 127.573 1.00 0.00 C**

**ATOM 2226 O ILE R 137 107.784 112.778 126.747 1.00 0.00 O**

**ATOM 2227 N ILE R 137 106.424 115.439 128.550 1.00 0.00 N**

**ATOM 2228 CB ILE R 137 108.041 116.120 126.811 1.00 0.00 C**

**ATOM 2229 CG2 ILE R 137 108.970 115.624 125.707 1.00 0.00 C**

**ATOM 2230 CG1 ILE R 137 107.222 117.325 126.337 1.00 0.00 C**

**ATOM 2231 CD1 ILE R 137 108.064 118.523 125.954 1.00 0.00 C**

**ATOM 2232 CA ILE R 146 107.684 101.711 123.364 1.00 0.00 C**

**ATOM 2233 C ILE R 146 106.522 101.115 122.567 1.00 0.00 C**

**ATOM 2234 O ILE R 146 106.646 100.029 121.976 1.00 0.00 O**

**ATOM 2235 N ILE R 146 107.823 103.143 123.089 1.00 0.00 N**

**ATOM 2236 CB ILE R 146 107.527 101.477 124.880 1.00 0.00 C**

**ATOM 2237 CG2 ILE R 146 107.235 100.019 125.187 1.00 0.00 C**

**ATOM 2238 CG1 ILE R 146 108.795 101.906 125.620 1.00 0.00 C**

**ATOM 2239 CD1 ILE R 146 108.651 101.907 127.126 1.00 0.00 C**

**ATOM 2240 CA ILE R 158 101.645 90.143 110.206 1.00 0.00 C**

**ATOM 2241 C ILE R 158 102.181 90.076 108.776 1.00 0.00 C**

**ATOM 2242 O ILE R 158 101.694 89.288 107.952 1.00 0.00 O**

**ATOM 2243 N ILE R 158 102.741 90.312 111.159 1.00 0.00 N**

**ATOM 2244 CB ILE R 158 100.616 91.278 110.396 1.00 0.00 C**

**ATOM 2245 CG2 ILE R 158 99.572 91.305 109.284 1.00 0.00 C**

**ATOM 2246 CG1 ILE R 158 99.911 91.129 111.746 1.00 0.00 C**

**ATOM 2247 CD1 ILE R 158 98.942 92.250 112.058 1.00 0.00 C**

**ATOM 2248 CA ILE R 177 95.406 105.814 111.619 1.00 0.00 C**

**ATOM 2249 C ILE R 177 95.882 105.127 112.899 1.00 0.00 C**

**ATOM 2250 O ILE R 177 95.601 105.591 114.014 1.00 0.00 O**

**ATOM 2251 N ILE R 177 96.506 105.948 110.655 1.00 0.00 N**

**ATOM 2252 CB ILE R 177 94.219 105.066 110.980 1.00 0.00 C**

**ATOM 2253 CG2 ILE R 177 93.173 104.685 112.022 1.00 0.00 C**

**ATOM 2254 CG1 ILE R 177 93.585 105.910 109.871 1.00 0.00 C**

**ATOM 2255 CD1 ILE R 177 92.510 105.185 109.093 1.00 0.00 C**

**ATOM 2256 CA ILE R 178 97.217 103.323 113.894 1.00 0.00 C**

**ATOM 2257 C ILE R 178 98.169 104.222 114.683 1.00 0.00 C**

**ATOM 2258 O ILE R 178 98.139 104.236 115.921 1.00 0.00 O**

**ATOM 2259 N ILE R 178 96.639 104.032 112.749 1.00 0.00 N**

**ATOM 2260 CB ILE R 178 97.902 102.022 113.426 1.00 0.00 C**

**ATOM 2261 CG2 ILE R 178 98.711 101.369 114.541 1.00 0.00 C**

**ATOM 2262 CG1 ILE R 178 96.861 101.028 112.910 1.00 0.00 C**

**ATOM 2263 CD1 ILE R 178 97.460 99.837 112.195 1.00 0.00 C**

**ATOM 2264 CA ILE R 180 97.328 108.557 115.601 1.00 0.00 C**

**ATOM 2265 C ILE R 180 96.674 107.903 116.815 1.00 0.00 C**

**ATOM 2266 O ILE R 180 96.584 108.518 117.885 1.00 0.00 O**

**ATOM 2267 N ILE R 180 98.148 107.587 114.869 1.00 0.00 N**

**ATOM 2268 CB ILE R 180 96.319 109.260 114.659 1.00 0.00 C**

**ATOM 2269 CG2 ILE R 180 94.994 109.618 115.341 1.00 0.00 C**

**ATOM 2270 CG1 ILE R 180 96.958 110.504 114.013 1.00 0.00 C**

**ATOM 2271 CD1 ILE R 180 97.581 110.345 112.666 1.00 0.00 C**

**ATOM 2272 CA ILE R 182 99.118 105.304 119.538 1.00 0.00 C**

**ATOM 2273 C ILE R 182 99.393 106.618 120.267 1.00 0.00 C**

**ATOM 2274 O ILE R 182 99.530 106.649 121.501 1.00 0.00 O**

**ATOM 2275 N ILE R 182 98.039 105.463 118.558 1.00 0.00 N**

**ATOM 2276 CB ILE R 182 100.398 104.802 118.840 1.00 0.00 C**

**ATOM 2277 CG2 ILE R 182 101.591 104.804 119.791 1.00 0.00 C**

**ATOM 2278 CG1 ILE R 182 100.217 103.412 118.248 1.00 0.00 C**

**ATOM 2279 CD1 ILE R 182 101.357 103.048 117.339 1.00 0.00 C**

**ATOM 2280 CA ILE R 191 97.241 114.829 130.326 1.00 0.00 C**

**ATOM 2281 C ILE R 191 97.322 114.562 131.829 1.00 0.00 C**

**ATOM 2282 O ILE R 191 97.281 115.500 132.638 1.00 0.00 O**

**ATOM 2283 N ILE R 191 98.127 113.927 129.591 1.00 0.00 N**

**ATOM 2284 CB ILE R 191 95.801 114.714 129.780 1.00 0.00 C**

**ATOM 2285 CG2 ILE R 191 94.798 115.517 130.603 1.00 0.00 C**

**ATOM 2286 CG1 ILE R 191 95.744 115.192 128.327 1.00 0.00 C**

**ATOM 2287 CD1 ILE R 191 94.424 114.914 127.643 1.00 0.00 C**

**ATOM 2288 CA ILE R 194 100.192 117.768 132.966 1.00 0.00 C**

**ATOM 2289 C ILE R 194 99.398 118.174 134.205 1.00 0.00 C**

**ATOM 2290 O ILE R 194 99.710 119.176 134.860 1.00 0.00 O**

**ATOM 2291 N ILE R 194 100.371 116.318 132.934 1.00 0.00 N**

**ATOM 2292 CB ILE R 194 99.529 118.259 131.660 1.00 0.00 C**

**ATOM 2293 CG2 ILE R 194 99.140 119.732 131.733 1.00 0.00 C**

**ATOM 2294 CG1 ILE R 194 100.461 118.041 130.468 1.00 0.00 C**

**ATOM 2295 CD1 ILE R 194 99.819 118.328 129.127 1.00 0.00 C**

**ATOM 2296 CA ILE R 208 113.016 122.013 145.963 1.00 0.00 C**

**ATOM 2297 C ILE R 208 113.074 120.786 145.057 1.00 0.00 C**

**ATOM 2298 O ILE R 208 114.101 120.532 144.417 1.00 0.00 O**

**ATOM 2299 N ILE R 208 114.262 122.057 146.716 1.00 0.00 N**

**ATOM 2300 CB ILE R 208 112.764 123.303 145.145 1.00 0.00 C**

**ATOM 2301 CG2 ILE R 208 111.375 123.312 144.636 1.00 0.00 C**

**ATOM 2302 CG1 ILE R 208 112.878 124.617 145.953 1.00 0.00 C**

**ATOM 2303 CD1 ILE R 208 114.279 125.233 146.173 1.00 0.00 C**

**ATOM 2304 CA ILE R 228 102.939 98.678 139.051 1.00 0.00 C**

**ATOM 2305 C ILE R 228 102.204 98.780 137.718 1.00 0.00 C**

**ATOM 2306 O ILE R 228 102.476 98.002 136.796 1.00 0.00 O**

**ATOM 2307 N ILE R 228 103.294 100.008 139.546 1.00 0.00 N**

**ATOM 2308 CB ILE R 228 102.121 97.892 140.099 1.00 0.00 C**

**ATOM 2309 CG2 ILE R 228 101.700 96.524 139.575 1.00 0.00 C**

**ATOM 2310 CG1 ILE R 228 102.913 97.708 141.394 1.00 0.00 C**

**ATOM 2311 CD1 ILE R 228 104.192 96.930 141.223 1.00 0.00 C**

**ATOM 2312 CA ILE R 232 103.186 96.076 133.677 1.00 0.00 C**

**ATOM 2313 C ILE R 232 102.864 96.481 132.244 1.00 0.00 C**

**ATOM 2314 O ILE R 232 103.304 95.809 131.302 1.00 0.00 O**

**ATOM 2315 N ILE R 232 103.884 97.153 134.381 1.00 0.00 N**

**ATOM 2316 CB ILE R 232 101.913 95.652 134.437 1.00 0.00 C**

**ATOM 2317 CG2 ILE R 232 101.055 94.734 133.577 1.00 0.00 C**

**ATOM 2318 CG1 ILE R 232 102.255 94.980 135.771 1.00 0.00 C**

**ATOM 2319 CD1 ILE R 232 103.038 93.706 135.645 1.00 0.00 C**

**ATOM 2320 CA ILE R 237 103.011 93.350 127.539 1.00 0.00 C**

**ATOM 2321 C ILE R 237 103.914 93.576 126.317 1.00 0.00 C**

**ATOM 2322 O ILE R 237 103.905 92.699 125.434 1.00 0.00 O**

**ATOM 2323 N ILE R 237 103.733 93.518 128.801 1.00 0.00 N**

**ATOM 2324 CB ILE R 237 101.745 94.231 127.510 1.00 0.00 C**

**ATOM 2325 CG2 ILE R 237 100.986 94.058 126.208 1.00 0.00 C**

**ATOM 2326 CG1 ILE R 237 100.834 93.895 128.692 1.00 0.00 C**

**ATOM 2327 CD1 ILE R 237 100.385 92.450 128.729 1.00 0.00 C**

**ATOM 2328 CA ILE R 241 104.493 90.560 122.360 1.00 0.00 C**

**ATOM 2329 C ILE R 241 105.687 90.471 121.409 1.00 0.00 C**

**ATOM 2330 O ILE R 241 105.620 89.776 120.389 1.00 0.00 O**

**ATOM 2331 N ILE R 241 104.918 90.422 123.755 1.00 0.00 N**

**ATOM 2332 CB ILE R 241 103.674 91.852 122.159 1.00 0.00 C**

**ATOM 2333 CG2 ILE R 241 103.506 92.192 120.687 1.00 0.00 C**

**ATOM 2334 CG1 ILE R 241 102.300 91.720 122.824 1.00 0.00 C**

**ATOM 2335 CD1 ILE R 241 101.425 92.947 122.672 1.00 0.00 C**

**ATOM 2336 CA ILE R 242 107.983 91.079 120.872 1.00 0.00 C**

**ATOM 2337 C ILE R 242 108.606 89.681 120.821 1.00 0.00 C**

**ATOM 2338 O ILE R 242 108.993 89.213 119.737 1.00 0.00 O**

**ATOM 2339 N ILE R 242 106.809 91.117 121.748 1.00 0.00 N**

**ATOM 2340 CB ILE R 242 108.986 92.164 121.326 1.00 0.00 C**

**ATOM 2341 CG2 ILE R 242 110.402 91.911 120.835 1.00 0.00 C**

**ATOM 2342 CG1 ILE R 242 108.488 93.558 120.927 1.00 0.00 C**

**ATOM 2343 CD1 ILE R 242 109.382 94.684 121.398 1.00 0.00 C**

**ATOM 2344 CA ILE R 243 109.104 87.576 121.955 1.00 0.00 C**

**ATOM 2345 C ILE R 243 108.224 86.650 121.124 1.00 0.00 C**

**ATOM 2346 O ILE R 243 108.733 85.902 120.282 1.00 0.00 O**

**ATOM 2347 N ILE R 243 108.582 88.945 121.939 1.00 0.00 N**

**ATOM 2348 CB ILE R 243 109.272 87.055 123.397 1.00 0.00 C**

**ATOM 2349 CG2 ILE R 243 109.684 85.588 123.407 1.00 0.00 C**

**ATOM 2350 CG1 ILE R 243 110.296 87.886 124.169 1.00 0.00 C**

**ATOM 2351 CD1 ILE R 243 111.670 87.877 123.554 1.00 0.00 C**

**ATOM 2352 CA ILE R 250 111.691 83.034 112.760 1.00 0.00 C**

**ATOM 2353 C ILE R 250 111.258 81.598 112.465 1.00 0.00 C**

**ATOM 2354 O ILE R 250 111.748 80.980 111.509 1.00 0.00 O**

**ATOM 2355 N ILE R 250 110.537 83.864 113.107 1.00 0.00 N**

**ATOM 2356 CB ILE R 250 112.760 83.107 113.871 1.00 0.00 C**

**ATOM 2357 CG2 ILE R 250 113.931 82.176 113.587 1.00 0.00 C**

**ATOM 2358 CG1 ILE R 250 113.278 84.539 114.022 1.00 0.00 C**

**ATOM 2359 CD1 ILE R 250 113.930 85.091 112.772 1.00 0.00 C**

**ATOM 2360 CA ILE R 272 118.114 88.622 109.634 1.00 0.00 C**

**ATOM 2361 C ILE R 272 117.871 88.135 111.062 1.00 0.00 C**

**ATOM 2362 O ILE R 272 117.407 88.892 111.931 1.00 0.00 O**

**ATOM 2363 N ILE R 272 119.403 88.129 109.135 1.00 0.00 N**

**ATOM 2364 CB ILE R 272 116.984 88.190 108.677 1.00 0.00 C**

**ATOM 2365 CG2 ILE R 272 115.605 88.451 109.275 1.00 0.00 C**

**ATOM 2366 CG1 ILE R 272 117.123 88.899 107.329 1.00 0.00 C**

**ATOM 2367 CD1 ILE R 272 116.054 88.520 106.328 1.00 0.00 C**

**ATOM 2368 CA ILE R 290 116.026 99.931 135.882 1.00 0.00 C**

**ATOM 2369 C ILE R 290 114.604 99.648 136.361 1.00 0.00 C**

**ATOM 2370 O ILE R 290 114.209 100.094 137.443 1.00 0.00 O**

**ATOM 2371 N ILE R 290 116.586 98.763 135.209 1.00 0.00 N**

**ATOM 2372 CB ILE R 290 116.124 101.182 134.980 1.00 0.00 C**

**ATOM 2373 CG2 ILE R 290 115.388 101.011 133.643 1.00 0.00 C**

**ATOM 2374 CG1 ILE R 290 115.696 102.443 135.745 1.00 0.00 C**

**ATOM 2375 CD1 ILE R 290 116.589 102.795 136.911 1.00 0.00 C**

**ATOM 2376 CA ILE R 292 113.606 95.766 138.521 1.00 0.00 C**

**ATOM 2377 C ILE R 292 114.107 96.586 139.712 1.00 0.00 C**

**ATOM 2378 O ILE R 292 113.612 96.428 140.834 1.00 0.00 O**

**ATOM 2379 N ILE R 292 113.477 96.601 137.323 1.00 0.00 N**

**ATOM 2380 CB ILE R 292 114.501 94.538 138.246 1.00 0.00 C**

**ATOM 2381 CG2 ILE R 292 114.866 93.781 139.522 1.00 0.00 C**

**ATOM 2382 CG1 ILE R 292 113.817 93.579 137.275 1.00 0.00 C**

**ATOM 2383 CD1 ILE R 292 114.717 92.461 136.825 1.00 0.00 C**

**ATOM 2384 CA ILE R 294 112.667 100.750 140.787 1.00 0.00 C**

**ATOM 2385 C ILE R 294 111.641 99.923 141.557 1.00 0.00 C**

**ATOM 2386 O ILE R 294 111.161 100.358 142.609 1.00 0.00 O**

**ATOM 2387 N ILE R 294 113.773 99.914 140.317 1.00 0.00 N**

**ATOM 2388 CB ILE R 294 112.038 101.529 139.611 1.00 0.00 C**

**ATOM 2389 CG2 ILE R 294 110.729 102.208 140.005 1.00 0.00 C**

**ATOM 2390 CG1 ILE R 294 113.020 102.583 139.093 1.00 0.00 C**

**ATOM 2391 CD1 ILE R 294 112.546 103.309 137.855 1.00 0.00 C**

**ATOM 2392 CA ILE R 316 118.658 105.422 133.840 1.00 0.00 C**

**ATOM 2393 C ILE R 316 119.377 105.811 132.550 1.00 0.00 C**

**ATOM 2394 O ILE R 316 118.754 105.946 131.484 1.00 0.00 O**

**ATOM 2395 N ILE R 316 119.433 104.416 134.571 1.00 0.00 N**

**ATOM 2396 CB ILE R 316 118.384 106.647 134.737 1.00 0.00 C**

**ATOM 2397 CG2 ILE R 316 117.706 107.766 133.963 1.00 0.00 C**

**ATOM 2398 CG1 ILE R 316 117.521 106.257 135.936 1.00 0.00 C**

**ATOM 2399 CD1 ILE R 316 117.219 107.407 136.863 1.00 0.00 C**

**ATOM 2400 CA ILE R 328 121.489 102.967 114.457 1.00 0.00 C**

**ATOM 2401 C ILE R 328 120.928 101.561 114.264 1.00 0.00 C**

**ATOM 2402 O ILE R 328 121.165 100.917 113.235 1.00 0.00 O**

**ATOM 2403 N ILE R 328 120.498 103.824 115.100 1.00 0.00 N**

**ATOM 2404 CB ILE R 328 122.789 102.966 115.286 1.00 0.00 C**

**ATOM 2405 CG2 ILE R 328 123.851 102.067 114.658 1.00 0.00 C**

**ATOM 2406 CG1 ILE R 328 123.334 104.390 115.416 1.00 0.00 C**

**ATOM 2407 CD1 ILE R 328 124.605 104.492 116.237 1.00 0.00 C**

**ATOM 2408 CA HIS R 162 99.874 86.449 107.302 1.00 0.00 C**

**ATOM 2409 C HIS R 162 100.016 87.298 106.042 1.00 0.00 C**

**ATOM 2410 O HIS R 162 99.629 88.475 106.031 1.00 0.00 O**

**ATOM 2411 N HIS R 162 101.170 86.315 107.968 1.00 0.00 N**

**ATOM 2412 CB HIS R 162 98.833 87.044 108.247 1.00 0.00 C**

**ATOM 2413 CG HIS R 162 98.620 86.238 109.489 1.00 0.00 C**

**ATOM 2414 ND1 HIS R 162 97.752 85.169 109.542 1.00 0.00 N**

**ATOM 2415 CE1 HIS R 162 97.770 84.649 110.756 1.00 0.00 C**

**ATOM 2416 NE2 HIS R 162 98.620 85.341 111.493 1.00 0.00 N**

**ATOM 2417 CD2 HIS R 162 99.166 86.340 110.724 1.00 0.00 C**

**ATOM 2418 CA HIS R 291 112.502 98.458 136.044 1.00 0.00 C**

**ATOM 2419 C HIS R 291 112.562 97.584 137.294 1.00 0.00 C**

**ATOM 2420 O HIS R 291 111.809 97.815 138.252 1.00 0.00 O**

**ATOM 2421 N HIS R 291 113.836 98.859 135.601 1.00 0.00 N**

**ATOM 2422 CB HIS R 291 111.752 97.763 134.907 1.00 0.00 C**

**ATOM 2423 CG HIS R 291 111.143 98.716 133.925 1.00 0.00 C**

**ATOM 2424 ND1 HIS R 291 109.929 99.332 134.146 1.00 0.00 N**

**ATOM 2425 CE1 HIS R 291 109.641 100.116 133.123 1.00 0.00 C**

**ATOM 2426 NE2 HIS R 291 110.627 100.036 132.248 1.00 0.00 N**

**ATOM 2427 CD2 HIS R 291 111.580 99.170 132.727 1.00 0.00 C**

**ATOM 2428 CA HIS R 304 120.091 105.501 150.120 1.00 0.00 C**

**ATOM 2429 C HIS R 304 121.619 105.491 150.222 1.00 0.00 C**

**ATOM 2430 O HIS R 304 122.219 106.110 151.108 1.00 0.00 O**

**ATOM 2431 N HIS R 304 119.497 104.350 150.792 1.00 0.00 N**

**ATOM 2432 CB HIS R 304 119.457 106.801 150.655 1.00 0.00 C**

**ATOM 2433 CG HIS R 304 119.561 106.983 152.140 1.00 0.00 C**

**ATOM 2434 ND1 HIS R 304 120.583 107.691 152.735 1.00 0.00 N**

**ATOM 2435 CE1 HIS R 304 120.415 107.687 154.045 1.00 0.00 C**

**ATOM 2436 NE2 HIS R 304 119.320 107.001 154.322 1.00 0.00 N**

**ATOM 2437 CD2 HIS R 304 118.767 106.551 153.148 1.00 0.00 C**

**END**

*********************************************************************************************************

**2. PDB Text: Figure 2 KOR (8f7w) with [P3,R8]-Dynorphin A1-11-OH**

**CRYST1 0.000 0.000 0.000 90.00 90.00 90.00 P 1 1**

**ATOM 1 CA TYR R 66 125.416 111.307 129.813 1.00 0.00 C**

**ATOM 2 C TYR R 66 125.567 112.260 128.626 1.00 0.00 C**

**ATOM 3 O TYR R 66 124.699 112.297 127.745 1.00 0.00 O**

**ATOM 4 N TYR R 66 126.660 110.597 130.131 1.00 0.00 N**

**ATOM 5 CB TYR R 66 124.900 112.079 131.031 1.00 0.00 C**

**ATOM 6 CG TYR R 66 124.024 111.281 131.978 1.00 0.00 C**

**ATOM 7 CD1 TYR R 66 122.678 111.082 131.693 1.00 0.00 C**

**ATOM 8 CE1 TYR R 66 121.857 110.372 132.554 1.00 0.00 C**

**ATOM 9 CZ TYR R 66 122.380 109.864 133.723 1.00 0.00 C**

**ATOM 10 OH TYR R 66 121.567 109.154 134.580 1.00 0.00 O**

**ATOM 11 CE2 TYR R 66 123.711 110.057 134.040 1.00 0.00 C**

**ATOM 12 CD2 TYR R 66 124.523 110.771 133.169 1.00 0.00 C**

**ATOM 13 CA TYR R 87 115.508 112.609 101.104 1.00 0.00 C**

**ATOM 14 C TYR R 87 114.531 111.549 100.598 1.00 0.00 C**

**ATOM 15 O TYR R 87 114.539 111.212 99.409 1.00 0.00 O**

**ATOM 16 N TYR R 87 114.884 113.574 102.013 1.00 0.00 N**

**ATOM 17 CB TYR R 87 116.713 111.953 101.774 1.00 0.00 C**

**ATOM 18 CG TYR R 87 117.926 112.852 101.845 1.00 0.00 C**

**ATOM 19 CD1 TYR R 87 118.883 112.834 100.835 1.00 0.00 C**

**ATOM 20 CE1 TYR R 87 119.995 113.655 100.889 1.00 0.00 C**

**ATOM 21 CZ TYR R 87 120.159 114.509 101.960 1.00 0.00 C**

**ATOM 22 OH TYR R 87 121.264 115.327 102.016 1.00 0.00 O**

**ATOM 23 CE2 TYR R 87 119.222 114.549 102.975 1.00 0.00 C**

**ATOM 24 CD2 TYR R 87 118.113 113.723 102.912 1.00 0.00 C**

**ATOM 25 CA TYR R 97 106.940 104.103 111.643 1.00 0.00 C**

**ATOM 26 C TYR R 97 108.311 104.595 112.109 1.00 0.00 C**

**ATOM 27 O TYR R 97 108.471 104.983 113.274 1.00 0.00 O**

**ATOM 28 N TYR R 97 106.585 104.622 110.318 1.00 0.00 N**

**ATOM 29 CB TYR R 97 106.882 102.574 111.664 1.00 0.00 C**

**ATOM 30 CG TYR R 97 105.487 101.996 111.792 1.00 0.00 C**

**ATOM 31 CD1 TYR R 97 104.835 101.991 113.020 1.00 0.00 C**

**ATOM 32 CE1 TYR R 97 103.565 101.459 113.157 1.00 0.00 C**

**ATOM 33 CZ TYR R 97 102.941 100.902 112.060 1.00 0.00 C**

**ATOM 34 OH TYR R 97 101.681 100.365 112.190 1.00 0.00 O**

**ATOM 35 CE2 TYR R 97 103.573 100.878 110.832 1.00 0.00 C**

**ATOM 36 CD2 TYR R 97 104.843 101.416 110.708 1.00 0.00 C**

**ATOM 37 CA TYR R 119 121.729 114.543 139.772 1.00 0.00 C**

**ATOM 38 C TYR R 119 122.878 115.333 140.398 1.00 0.00 C**

**ATOM 39 O TYR R 119 123.213 115.133 141.571 1.00 0.00 O**

**ATOM 40 N TYR R 119 120.754 115.414 139.116 1.00 0.00 N**

**ATOM 41 CB TYR R 119 122.272 113.510 138.785 1.00 0.00 C**

**ATOM 42 CG TYR R 119 123.464 112.752 139.329 1.00 0.00 C**

**ATOM 43 CD1 TYR R 119 123.323 111.883 140.406 1.00 0.00 C**

**ATOM 44 CE1 TYR R 119 124.407 111.189 140.914 1.00 0.00 C**

**ATOM 45 CZ TYR R 119 125.652 111.357 140.341 1.00 0.00 C**

**ATOM 46 OH TYR R 119 126.731 110.666 140.844 1.00 0.00 O**

**ATOM 47 CE2 TYR R 119 125.820 112.213 139.269 1.00 0.00 C**

**ATOM 48 CD2 TYR R 119 124.727 112.904 138.769 1.00 0.00 C**

**ATOM 49 CA TYR R 139 106.459 110.053 130.061 1.00 0.00 C**

**ATOM 50 C TYR R 139 105.717 109.750 128.760 1.00 0.00 C**

**ATOM 51 O TYR R 139 105.679 108.592 128.309 1.00 0.00 O**

**ATOM 52 N TYR R 139 107.367 111.195 129.917 1.00 0.00 N**

**ATOM 53 CB TYR R 139 105.468 110.296 131.203 1.00 0.00 C**

**ATOM 54 CG TYR R 139 106.027 110.038 132.588 1.00 0.00 C**

**ATOM 55 CD1 TYR R 139 105.659 108.901 133.299 1.00 0.00 C**

**ATOM 56 CE1 TYR R 139 106.159 108.654 134.566 1.00 0.00 C**

**ATOM 57 CZ TYR R 139 107.034 109.553 135.140 1.00 0.00 C**

**ATOM 58 OH TYR R 139 107.532 109.309 136.400 1.00 0.00 O**

**ATOM 59 CE2 TYR R 139 107.412 110.693 134.459 1.00 0.00 C**

**ATOM 60 CD2 TYR R 139 106.907 110.931 133.189 1.00 0.00 C**

**ATOM 61 CA TYR R 140 104.303 110.705 126.953 1.00 0.00 C**

**ATOM 62 C TYR R 140 105.105 110.066 125.820 1.00 0.00 C**

**ATOM 63 O TYR R 140 104.701 109.043 125.254 1.00 0.00 O**

**ATOM 64 N TYR R 140 105.058 110.776 128.200 1.00 0.00 N**

**ATOM 65 CB TYR R 140 103.875 112.134 126.604 1.00 0.00 C**

**ATOM 66 CG TYR R 140 103.084 112.344 125.340 1.00 0.00 C**

**ATOM 67 CD1 TYR R 140 101.708 112.180 125.338 1.00 0.00 C**

**ATOM 68 CE1 TYR R 140 100.969 112.399 124.201 1.00 0.00 C**

**ATOM 69 CZ TYR R 140 101.588 112.810 123.050 1.00 0.00 C**

**ATOM 70 OH TYR R 140 100.812 113.024 121.937 1.00 0.00 O**

**ATOM 71 CE2 TYR R 140 102.955 113.009 123.024 1.00 0.00 C**

**ATOM 72 CD2 TYR R 140 103.696 112.784 124.171 1.00 0.00 C**

**ATOM 73 CA TYR R 157 104.831 89.714 112.263 1.00 0.00 C**

**ATOM 74 C TYR R 157 103.683 89.380 111.317 1.00 0.00 C**

**ATOM 75 O TYR R 157 103.620 88.273 110.771 1.00 0.00 O**

**ATOM 76 N TYR R 157 105.361 91.050 111.994 1.00 0.00 N**

**ATOM 77 CB TYR R 157 104.376 89.620 113.721 1.00 0.00 C**

**ATOM 78 CG TYR R 157 103.531 88.405 114.048 1.00 0.00 C**

**ATOM 79 CD1 TYR R 157 104.110 87.151 114.204 1.00 0.00 C**

**ATOM 80 CE1 TYR R 157 103.340 86.042 114.514 1.00 0.00 C**

**ATOM 81 CZ TYR R 157 101.975 86.181 114.671 1.00 0.00 C**

**ATOM 82 OH TYR R 157 101.203 85.084 114.973 1.00 0.00 O**

**ATOM 83 CE2 TYR R 157 101.376 87.415 114.526 1.00 0.00 C**

**ATOM 84 CD2 TYR R 157 102.154 88.519 114.222 1.00 0.00 C**

**ATOM 85 CA TYR R 219 101.031 108.686 148.819 1.00 0.00 C**

**ATOM 86 C TYR R 219 101.804 107.431 148.439 1.00 0.00 C**

**ATOM 87 O TYR R 219 102.017 107.171 147.247 1.00 0.00 O**

**ATOM 88 N TYR R 219 100.029 108.415 149.846 1.00 0.00 N**

**ATOM 89 CB TYR R 219 102.002 109.766 149.303 1.00 0.00 C**

**ATOM 90 CG TYR R 219 103.034 110.172 148.269 1.00 0.00 C**

**ATOM 91 CD1 TYR R 219 102.708 111.045 147.242 1.00 0.00 C**

**ATOM 92 CE1 TYR R 219 103.643 111.414 146.290 1.00 0.00 C**

**ATOM 93 CZ TYR R 219 104.923 110.904 146.359 1.00 0.00 C**

**ATOM 94 OH TYR R 219 105.857 111.268 145.416 1.00 0.00 O**

**ATOM 95 CE2 TYR R 219 105.274 110.030 147.369 1.00 0.00 C**

**ATOM 96 CD2 TYR R 219 104.333 109.672 148.317 1.00 0.00 C**

**ATOM 97 CA TYR R 246 109.707 87.440 116.493 1.00 0.00 C**

**ATOM 98 C TYR R 246 109.994 85.941 116.542 1.00 0.00 C**

**ATOM 99 O TYR R 246 110.587 85.411 115.595 1.00 0.00 O**

**ATOM 100 N TYR R 246 108.466 87.803 117.176 1.00 0.00 N**

**ATOM 101 CB TYR R 246 110.885 88.237 117.047 1.00 0.00 C**

**ATOM 102 CG TYR R 246 110.888 89.682 116.597 1.00 0.00 C**

**ATOM 103 CD1 TYR R 246 110.989 90.003 115.247 1.00 0.00 C**

**ATOM 104 CE1 TYR R 246 110.995 91.320 114.822 1.00 0.00 C**

**ATOM 105 CZ TYR R 246 110.907 92.334 115.753 1.00 0.00 C**

**ATOM 106 OH TYR R 246 110.915 93.646 115.338 1.00 0.00 O**

**ATOM 107 CE2 TYR R 246 110.811 92.041 117.098 1.00 0.00 C**

**ATOM 108 CD2 TYR R 246 110.806 90.719 117.511 1.00 0.00 C**

**ATOM 109 CA TYR R 312 120.532 104.023 139.757 1.00 0.00 C**

**ATOM 110 C TYR R 312 120.565 104.941 138.539 1.00 0.00 C**

**ATOM 111 O TYR R 312 119.884 104.687 137.532 1.00 0.00 O**

**ATOM 112 N TYR R 312 121.876 103.596 140.140 1.00 0.00 N**

**ATOM 113 CB TYR R 312 119.874 104.710 140.956 1.00 0.00 C**

**ATOM 114 CG TYR R 312 118.520 105.333 140.715 1.00 0.00 C**

**ATOM 115 CD1 TYR R 312 117.377 104.548 140.635 1.00 0.00 C**

**ATOM 116 CE1 TYR R 312 116.133 105.114 140.437 1.00 0.00 C**

**ATOM 117 CZ TYR R 312 116.018 106.485 140.342 1.00 0.00 C**

**ATOM 118 OH TYR R 312 114.781 107.050 140.150 1.00 0.00 O**

**ATOM 119 CE2 TYR R 312 117.136 107.292 140.444 1.00 0.00 C**

**ATOM 120 CD2 TYR R 312 118.376 106.713 140.634 1.00 0.00 C**

**ATOM 121 CA TYR R 313 121.480 106.873 137.431 1.00 0.00 C**

**ATOM 122 C TYR R 313 122.231 106.241 136.262 1.00 0.00 C**

**ATOM 123 O TYR R 313 122.008 106.643 135.117 1.00 0.00 O**

**ATOM 124 N TYR R 313 121.395 105.987 138.588 1.00 0.00 N**

**ATOM 125 CB TYR R 313 122.108 108.211 137.823 1.00 0.00 C**

**ATOM 126 CG TYR R 313 121.136 109.114 138.550 1.00 0.00 C**

**ATOM 127 CD1 TYR R 313 120.221 109.887 137.839 1.00 0.00 C**

**ATOM 128 CE1 TYR R 313 119.316 110.707 138.493 1.00 0.00 C**

**ATOM 129 CZ TYR R 313 119.316 110.756 139.872 1.00 0.00 C**

**ATOM 130 OH TYR R 313 118.422 111.569 140.532 1.00 0.00 O**

**ATOM 131 CE2 TYR R 313 120.209 109.995 140.598 1.00 0.00 C**

**ATOM 132 CD2 TYR R 313 121.109 109.175 139.937 1.00 0.00 C**

**ATOM 133 CA TYR R 320 118.111 106.452 127.573 1.00 0.00 C**

**ATOM 134 C TYR R 320 119.100 106.067 126.471 1.00 0.00 C**

**ATOM 135 O TYR R 320 118.962 106.533 125.331 1.00 0.00 O**

**ATOM 136 N TYR R 320 118.035 105.448 128.633 1.00 0.00 N**

**ATOM 137 CB TYR R 320 118.470 107.811 128.166 1.00 0.00 C**

**ATOM 138 CG TYR R 320 117.327 108.484 128.893 1.00 0.00 C**

**ATOM 139 CD1 TYR R 320 116.342 109.168 128.190 1.00 0.00 C**

**ATOM 140 CE1 TYR R 320 115.295 109.787 128.850 1.00 0.00 C**

**ATOM 141 CZ TYR R 320 115.221 109.721 130.223 1.00 0.00 C**

**ATOM 142 OH TYR R 320 114.180 110.333 130.882 1.00 0.00 O**

**ATOM 143 CE2 TYR R 320 116.184 109.048 130.942 1.00 0.00 C**

**ATOM 144 CD2 TYR R 320 117.230 108.436 130.277 1.00 0.00 C**

**ATOM 145 CA TYR R 330 116.530 100.688 113.037 1.00 0.00 C**

**ATOM 146 C TYR R 330 116.674 101.359 111.680 1.00 0.00 C**

**ATOM 147 O TYR R 330 116.139 100.841 110.702 1.00 0.00 O**

**ATOM 148 N TYR R 330 117.713 100.747 113.890 1.00 0.00 N**

**ATOM 149 CB TYR R 330 115.325 101.290 113.767 1.00 0.00 C**

**ATOM 150 CG TYR R 330 114.704 100.344 114.767 1.00 0.00 C**

**ATOM 151 CD1 TYR R 330 113.722 99.443 114.370 1.00 0.00 C**

**ATOM 152 CE1 TYR R 330 113.146 98.571 115.272 1.00 0.00 C**

**ATOM 153 CZ TYR R 330 113.563 98.579 116.585 1.00 0.00 C**

**ATOM 154 OH TYR R 330 112.993 97.706 117.481 1.00 0.00 O**

**ATOM 155 CE2 TYR R 330 114.544 99.458 117.006 1.00 0.00 C**

**ATOM 156 CD2 TYR R 330 115.111 100.333 116.096 1.00 0.00 C**

**ATOM 157 CA VAL R 60 132.940 113.494 135.847 1.00 0.00 C**

**ATOM 158 C VAL R 60 132.721 112.443 134.762 1.00 0.00 C**

**ATOM 159 O VAL R 60 132.238 112.758 133.667 1.00 0.00 O**

**ATOM 160 N VAL R 60 132.456 113.012 137.138 1.00 0.00 N**

**ATOM 161 CB VAL R 60 134.422 113.898 135.955 1.00 0.00 C**

**ATOM 162 CG1 VAL R 60 134.956 114.376 134.611 1.00 0.00 C**

**ATOM 163 CG2 VAL R 60 134.600 114.972 137.017 1.00 0.00 C**

**ATOM 164 CA VAL R 65 128.665 109.292 129.605 1.00 0.00 C**

**ATOM 165 C VAL R 65 127.308 109.868 129.207 1.00 0.00 C**

**ATOM 166 O VAL R 65 126.826 109.635 128.089 1.00 0.00 O**

**ATOM 167 N VAL R 65 129.488 110.315 130.256 1.00 0.00 N**

**ATOM 168 CB VAL R 65 128.523 108.052 130.510 1.00 0.00 C**

**ATOM 169 CG1 VAL R 65 127.535 107.056 129.924 1.00 0.00 C**

**ATOM 170 CG2 VAL R 65 129.862 107.355 130.647 1.00 0.00 C**

**ATOM 171 CA VAL R 68 128.018 111.266 124.955 1.00 0.00 C**

**ATOM 172 C VAL R 68 126.689 110.723 124.437 1.00 0.00 C**

**ATOM 173 O VAL R 68 126.377 110.853 123.247 1.00 0.00 O**

**ATOM 174 N VAL R 68 127.814 112.072 126.163 1.00 0.00 N**

**ATOM 175 CB VAL R 68 129.034 110.137 125.212 1.00 0.00 C**

**ATOM 176 CG1 VAL R 68 129.065 109.166 124.043 1.00 0.00 C**

**ATOM 177 CG2 VAL R 68 130.424 110.721 125.381 1.00 0.00 C**

**ATOM 178 CA VAL R 69 124.605 109.519 124.954 1.00 0.00 C**

**ATOM 179 C VAL R 69 123.680 110.616 124.422 1.00 0.00 C**

**ATOM 180 O VAL R 69 122.994 110.421 123.409 1.00 0.00 O**

**ATOM 181 N VAL R 69 125.923 110.055 125.315 1.00 0.00 N**

**ATOM 182 CB VAL R 69 123.985 108.772 126.152 1.00 0.00 C**

**ATOM 183 CG1 VAL R 69 122.601 108.252 125.813 1.00 0.00 C**

**ATOM 184 CG2 VAL R 69 124.855 107.588 126.529 1.00 0.00 C**

**ATOM 185 CA VAL R 71 124.979 114.157 121.666 1.00 0.00 C**

**ATOM 186 C VAL R 71 124.735 113.124 120.567 1.00 0.00 C**

**ATOM 187 O VAL R 71 124.207 113.459 119.498 1.00 0.00 O**

**ATOM 188 N VAL R 71 124.527 113.658 122.967 1.00 0.00 N**

**ATOM 189 CB VAL R 71 126.458 114.584 121.735 1.00 0.00 C**

**ATOM 190 CG1 VAL R 71 126.978 114.962 120.358 1.00 0.00 C**

**ATOM 191 CG2 VAL R 71 126.612 115.779 122.662 1.00 0.00 C**

**ATOM 192 CA VAL R 72 124.943 110.775 119.843 1.00 0.00 C**

**ATOM 193 C VAL R 72 123.464 110.607 119.496 1.00 0.00 C**

**ATOM 194 O VAL R 72 123.096 110.527 118.315 1.00 0.00 O**

**ATOM 195 N VAL R 72 125.150 111.868 120.803 1.00 0.00 N**

**ATOM 196 CB VAL R 72 125.557 109.467 120.379 1.00 0.00 C**

**ATOM 197 CG1 VAL R 72 125.098 108.269 119.560 1.00 0.00 C**

**ATOM 198 CG2 VAL R 72 127.075 109.547 120.332 1.00 0.00 C**

**ATOM 199 CA VAL R 75 122.622 113.817 116.090 1.00 0.00 C**

**ATOM 200 C VAL R 75 121.976 112.710 115.261 1.00 0.00 C**

**ATOM 201 O VAL R 75 121.556 112.941 114.118 1.00 0.00 O**

**ATOM 202 N VAL R 75 122.115 113.799 117.464 1.00 0.00 N**

**ATOM 203 CB VAL R 75 124.161 113.726 116.081 1.00 0.00 C**

**ATOM 204 CG1 VAL R 75 124.694 113.570 114.665 1.00 0.00 C**

**ATOM 205 CG2 VAL R 75 124.759 114.987 116.683 1.00 0.00 C**

**ATOM 206 CA VAL R 80 117.127 110.308 110.517 1.00 0.00 C**

**ATOM 207 C VAL R 80 115.874 111.117 110.191 1.00 0.00 C**

**ATOM 208 O VAL R 80 115.247 110.914 109.138 1.00 0.00 O**

**ATOM 209 N VAL R 80 118.212 111.181 110.976 1.00 0.00 N**

**ATOM 210 CB VAL R 80 116.847 109.204 111.557 1.00 0.00 C**

**ATOM 211 CG1 VAL R 80 115.621 108.390 111.171 1.00 0.00 C**

**ATOM 212 CG2 VAL R 80 118.042 108.271 111.664 1.00 0.00 C**

**ATOM 213 CA VAL R 83 116.329 112.182 105.731 1.00 0.00 C**

**ATOM 214 C VAL R 83 114.879 111.822 105.408 1.00 0.00 C**

**ATOM 215 O VAL R 83 114.470 111.843 104.239 1.00 0.00 O**

**ATOM 216 N VAL R 83 116.407 113.064 106.898 1.00 0.00 N**

**ATOM 217 CB VAL R 83 117.208 110.931 105.942 1.00 0.00 C**

**ATOM 218 CG1 VAL R 83 116.953 109.888 104.868 1.00 0.00 C**

**ATOM 219 CG2 VAL R 83 118.676 111.316 105.896 1.00 0.00 C**

**ATOM 220 CA VAL R 108 112.935 111.654 124.849 1.00 0.00 C**

**ATOM 221 C VAL R 108 114.371 112.063 125.201 1.00 0.00 C**

**ATOM 222 O VAL R 108 114.688 112.278 126.380 1.00 0.00 O**

**ATOM 223 N VAL R 108 112.489 112.293 123.612 1.00 0.00 N**

**ATOM 224 CB VAL R 108 112.754 110.124 124.723 1.00 0.00 C**

**ATOM 225 CG1 VAL R 108 113.553 109.354 125.753 1.00 0.00 C**

**ATOM 226 CG2 VAL R 108 111.292 109.764 124.914 1.00 0.00 C**

**ATOM 227 CA VAL R 118 119.074 117.198 139.062 1.00 0.00 C**

**ATOM 228 C VAL R 118 120.019 116.272 139.828 1.00 0.00 C**

**ATOM 229 O VAL R 118 120.096 116.326 141.061 1.00 0.00 O**

**ATOM 230 N VAL R 118 119.694 117.670 137.820 1.00 0.00 N**

**ATOM 231 CB VAL R 118 117.717 116.528 138.751 1.00 0.00 C**

**ATOM 232 CG1 VAL R 118 117.141 115.795 139.959 1.00 0.00 C**

**ATOM 233 CG2 VAL R 118 116.718 117.574 138.310 1.00 0.00 C**

**ATOM 234 CA VAL R 129 106.032 125.233 133.928 1.00 0.00 C**

**ATOM 235 C VAL R 129 107.003 124.288 133.228 1.00 0.00 C**

**ATOM 236 O VAL R 129 106.590 123.332 132.552 1.00 0.00 O**

**ATOM 237 N VAL R 129 106.470 125.506 135.298 1.00 0.00 N**

**ATOM 238 CB VAL R 129 105.867 126.557 133.157 1.00 0.00 C**

**ATOM 239 CG1 VAL R 129 105.512 126.305 131.697 1.00 0.00 C**

**ATOM 240 CG2 VAL R 129 104.818 127.432 133.825 1.00 0.00 C**

**ATOM 241 CA VAL R 134 109.542 118.070 130.807 1.00 0.00 C**

**ATOM 242 C VAL R 134 109.040 116.709 131.296 1.00 0.00 C**

**ATOM 243 O VAL R 134 109.287 115.681 130.646 1.00 0.00 O**

**ATOM 244 N VAL R 134 108.504 119.097 130.934 1.00 0.00 N**

**ATOM 245 CB VAL R 134 110.833 118.520 131.524 1.00 0.00 C**

**ATOM 246 CG1 VAL R 134 111.847 117.403 131.587 1.00 0.00 C**

**ATOM 247 CG2 VAL R 134 111.472 119.666 130.755 1.00 0.00 C**

**ATOM 248 CA VAL R 154 103.349 93.336 115.194 1.00 0.00 C**

**ATOM 249 C VAL R 154 103.158 93.479 113.683 1.00 0.00 C**

**ATOM 250 O VAL R 154 102.671 92.555 113.015 1.00 0.00 O**

**ATOM 251 N VAL R 154 104.606 93.956 115.621 1.00 0.00 N**

**ATOM 252 CB VAL R 154 102.167 93.934 115.987 1.00 0.00 C**

**ATOM 253 CG1 VAL R 154 100.825 93.397 115.501 1.00 0.00 C**

**ATOM 254 CG2 VAL R 154 102.313 93.614 117.456 1.00 0.00 C**

**ATOM 255 CA VAL R 160 105.634 87.529 107.470 1.00 0.00 C**

**ATOM 256 C VAL R 160 104.681 86.345 107.609 1.00 0.00 C**

**ATOM 257 O VAL R 160 104.618 85.486 106.729 1.00 0.00 O**

**ATOM 258 N VAL R 160 104.971 88.802 107.758 1.00 0.00 N**

**ATOM 259 CB VAL R 160 106.882 87.357 108.366 1.00 0.00 C**

**ATOM 260 CG1 VAL R 160 107.539 86.015 108.114 1.00 0.00 C**

**ATOM 261 CG2 VAL R 160 107.881 88.457 108.078 1.00 0.00 C**

**ATOM 262 CA VAL R 164 97.297 87.700 102.429 1.00 0.00 C**

**ATOM 263 C VAL R 164 96.657 88.873 103.166 1.00 0.00 C**

**ATOM 264 O VAL R 164 96.106 89.789 102.540 1.00 0.00 O**

**ATOM 265 N VAL R 164 98.513 87.277 103.118 1.00 0.00 N**

**ATOM 266 CB VAL R 164 96.337 86.500 102.293 1.00 0.00 C**

**ATOM 267 CG1 VAL R 164 95.051 86.884 101.565 1.00 0.00 C**

**ATOM 268 CG2 VAL R 164 97.030 85.345 101.584 1.00 0.00 C**

**ATOM 269 CA VAL R 189 99.110 109.660 130.466 1.00 0.00 C**

**ATOM 270 C VAL R 189 99.753 111.037 130.347 1.00 0.00 C**

**ATOM 271 O VAL R 189 100.215 111.604 131.345 1.00 0.00 O**

**ATOM 272 N VAL R 189 98.048 109.522 129.476 1.00 0.00 N**

**ATOM 273 CB VAL R 189 100.131 108.516 130.300 1.00 0.00 C**

**ATOM 274 CG1 VAL R 189 101.186 108.546 131.401 1.00 0.00 C**

**ATOM 275 CG2 VAL R 189 99.418 107.171 130.272 1.00 0.00 C**

**ATOM 276 CA VAL R 195 97.570 117.734 135.730 1.00 0.00 C**

**ATOM 277 C VAL R 195 98.281 117.440 137.051 1.00 0.00 C**

**ATOM 278 O VAL R 195 98.270 118.267 137.971 1.00 0.00 O**

**ATOM 279 N VAL R 195 98.407 117.370 134.590 1.00 0.00 N**

**ATOM 280 CB VAL R 195 96.206 117.023 135.632 1.00 0.00 C**

**ATOM 281 CG1 VAL R 195 95.377 117.243 136.888 1.00 0.00 C**

**ATOM 282 CG2 VAL R 195 95.436 117.539 134.427 1.00 0.00 C**

**ATOM 283 CA VAL R 201 109.685 122.912 148.148 1.00 0.00 C**

**ATOM 284 C VAL R 201 109.647 122.126 149.461 1.00 0.00 C**

**ATOM 285 O VAL R 201 108.587 121.965 150.079 1.00 0.00 O**

**ATOM 286 N VAL R 201 108.508 122.583 147.347 1.00 0.00 N**

**ATOM 287 CB VAL R 201 109.772 124.441 148.373 1.00 0.00 C**

**ATOM 288 CG1 VAL R 201 108.492 125.002 149.001 1.00 0.00 C**

**ATOM 289 CG2 VAL R 201 110.999 124.824 149.203 1.00 0.00 C**

**ATOM 290 CA VAL R 205 116.029 119.626 153.624 1.00 0.00 C**

**ATOM 291 C VAL R 205 116.447 120.913 152.920 1.00 0.00 C**

**ATOM 292 O VAL R 205 117.608 121.029 152.497 1.00 0.00 O**

**ATOM 293 N VAL R 205 114.854 119.860 154.457 1.00 0.00 N**

**ATOM 294 CB VAL R 205 115.660 118.435 152.707 1.00 0.00 C**

**ATOM 295 CG1 VAL R 205 116.711 118.123 151.633 1.00 0.00 C**

**ATOM 296 CG2 VAL R 205 115.380 117.193 153.544 1.00 0.00 C**

**ATOM 297 CA VAL R 207 115.685 121.897 148.687 1.00 0.00 C**

**ATOM 298 C VAL R 207 114.322 121.760 148.014 1.00 0.00 C**

**ATOM 299 O VAL R 207 113.338 121.380 148.644 1.00 0.00 O**

**ATOM 300 N VAL R 207 115.406 122.199 150.091 1.00 0.00 N**

**ATOM 301 CB VAL R 207 116.554 120.588 148.538 1.00 0.00 C**

**ATOM 302 CG1 VAL R 207 116.794 120.154 147.099 1.00 0.00 C**

**ATOM 303 CG2 VAL R 207 117.938 120.710 149.188 1.00 0.00 C**

**ATOM 304 CA VAL R 230 103.489 101.651 134.430 1.00 0.00 C**

**ATOM 305 C VAL R 230 104.392 100.480 134.063 1.00 0.00 C**

**ATOM 306 O VAL R 230 104.621 100.216 132.876 1.00 0.00 O**

**ATOM 307 N VAL R 230 102.497 101.249 135.428 1.00 0.00 N**

**ATOM 308 CB VAL R 230 104.295 102.868 134.922 1.00 0.00 C**

**ATOM 309 CG1 VAL R 230 105.460 103.159 133.991 1.00 0.00 C**

**ATOM 310 CG2 VAL R 230 103.406 104.098 134.962 1.00 0.00 C**

**ATOM 311 CA VAL R 236 105.385 92.955 130.504 1.00 0.00 C**

**ATOM 312 C VAL R 236 104.689 92.676 129.176 1.00 0.00 C**

**ATOM 313 O VAL R 236 105.025 91.716 128.475 1.00 0.00 O**

**ATOM 314 N VAL R 236 105.789 94.359 130.606 1.00 0.00 N**

**ATOM 315 CB VAL R 236 104.508 92.565 131.709 1.00 0.00 C**

**ATOM 316 CG1 VAL R 236 103.901 91.183 131.517 1.00 0.00 C**

**ATOM 317 CG2 VAL R 236 105.345 92.592 132.973 1.00 0.00 C**

**ATOM 318 CA VAL R 239 108.286 92.217 125.879 1.00 0.00 C**

**ATOM 319 C VAL R 239 107.686 90.901 125.395 1.00 0.00 C**

**ATOM 320 O VAL R 239 108.215 90.282 124.467 1.00 0.00 O**

**ATOM 321 N VAL R 239 107.256 93.255 125.952 1.00 0.00 N**

**ATOM 322 CB VAL R 239 109.011 92.060 127.231 1.00 0.00 C**

**ATOM 323 CG1 VAL R 239 109.759 90.735 127.312 1.00 0.00 C**

**ATOM 324 CG2 VAL R 239 109.995 93.203 127.426 1.00 0.00 C**

**ATOM 325 CA VAL R 244 105.976 85.840 120.586 1.00 0.00 C**

**ATOM 326 C VAL R 244 106.036 86.154 119.091 1.00 0.00 C**

**ATOM 327 O VAL R 244 106.157 85.244 118.258 1.00 0.00 O**

**ATOM 328 N VAL R 244 106.900 86.694 121.339 1.00 0.00 N**

**ATOM 329 CB VAL R 244 104.546 85.972 121.143 1.00 0.00 C**

**ATOM 330 CG1 VAL R 244 103.538 85.306 120.220 1.00 0.00 C**

**ATOM 331 CG2 VAL R 244 104.462 85.330 122.518 1.00 0.00 C**

**ATOM 332 CA VAL R 256 110.300 78.955 103.641 1.00 0.00 C**

**ATOM 333 C VAL R 256 111.299 78.272 102.717 1.00 0.00 C**

**ATOM 334 O VAL R 256 112.484 78.151 103.052 1.00 0.00 O**

**ATOM 335 N VAL R 256 110.373 78.319 104.953 1.00 0.00 N**

**ATOM 336 CB VAL R 256 110.574 80.467 103.741 1.00 0.00 C**

**ATOM 337 CG1 VAL R 256 110.810 81.073 102.364 1.00 0.00 C**

**ATOM 338 CG2 VAL R 256 109.421 81.166 104.442 1.00 0.00 C**

**ATOM 339 CA VAL R 276 116.353 90.216 115.454 1.00 0.00 C**

**ATOM 340 C VAL R 276 116.878 89.999 116.873 1.00 0.00 C**

**ATOM 341 O VAL R 276 116.426 90.653 117.827 1.00 0.00 O**

**ATOM 342 N VAL R 276 117.458 90.386 114.505 1.00 0.00 N**

**ATOM 343 CB VAL R 276 115.440 89.061 115.001 1.00 0.00 C**

**ATOM 344 CG1 VAL R 276 114.467 88.671 116.099 1.00 0.00 C**

**ATOM 345 CG2 VAL R 276 114.676 89.457 113.747 1.00 0.00 C**

**ATOM 346 CA VAL R 278 120.462 92.033 118.569 1.00 0.00 C**

**ATOM 347 C VAL R 278 119.431 93.028 119.092 1.00 0.00 C**

**ATOM 348 O VAL R 278 119.591 93.577 120.189 1.00 0.00 O**

**ATOM 349 N VAL R 278 119.813 90.807 118.096 1.00 0.00 N**

**ATOM 350 CB VAL R 278 121.347 92.646 117.464 1.00 0.00 C**

**ATOM 351 CG1 VAL R 278 121.750 94.069 117.813 1.00 0.00 C**

**ATOM 352 CG2 VAL R 278 122.621 91.834 117.313 1.00 0.00 C**

**ATOM 353 CA VAL R 279 117.343 94.260 118.695 1.00 0.00 C**

**ATOM 354 C VAL R 279 116.660 93.844 120.001 1.00 0.00 C**

**ATOM 355 O VAL R 279 116.499 94.660 120.927 1.00 0.00 O**

**ATOM 356 N VAL R 279 118.381 93.299 118.300 1.00 0.00 N**

**ATOM 357 CB VAL R 279 116.326 94.436 117.549 1.00 0.00 C**

**ATOM 358 CG1 VAL R 279 115.111 95.214 118.017 1.00 0.00 C**

**ATOM 359 CG2 VAL R 279 116.975 95.151 116.374 1.00 0.00 C**

**ATOM 360 CA VAL R 280 115.643 92.088 121.335 1.00 0.00 C**

**ATOM 361 C VAL R 280 116.583 92.079 122.540 1.00 0.00 C**

**ATOM 362 O VAL R 280 116.185 92.454 123.655 1.00 0.00 O**

**ATOM 363 N VAL R 280 116.340 92.552 120.136 1.00 0.00 N**

**ATOM 364 CB VAL R 280 115.014 90.704 121.074 1.00 0.00 C**

**ATOM 365 CG1 VAL R 280 114.388 90.127 122.333 1.00 0.00 C**

**ATOM 366 CG2 VAL R 280 113.955 90.809 119.989 1.00 0.00 C**

**ATOM 367 CA VAL R 282 119.362 95.515 123.411 1.00 0.00 C**

**ATOM 368 C VAL R 282 118.159 96.013 124.203 1.00 0.00 C**

**ATOM 369 O VAL R 282 118.321 96.634 125.263 1.00 0.00 O**

**ATOM 370 N VAL R 282 119.170 94.123 122.999 1.00 0.00 N**

**ATOM 371 CB VAL R 282 119.665 96.409 122.190 1.00 0.00 C**

**ATOM 372 CG1 VAL R 282 119.633 97.881 122.572 1.00 0.00 C**

**ATOM 373 CG2 VAL R 282 121.045 96.092 121.643 1.00 0.00 C**

**ATOM 374 CA VAL R 284 115.882 93.584 127.299 1.00 0.00 C**

**ATOM 375 C VAL R 284 116.980 94.072 128.243 1.00 0.00 C**

**ATOM 376 O VAL R 284 116.696 94.481 129.378 1.00 0.00 O**

**ATOM 377 N VAL R 284 115.979 94.245 125.997 1.00 0.00 N**

**ATOM 378 CB VAL R 284 115.911 92.054 127.129 1.00 0.00 C**

**ATOM 379 CG1 VAL R 284 116.043 91.365 128.477 1.00 0.00 C**

**ATOM 380 CG2 VAL R 284 114.641 91.585 126.440 1.00 0.00 C**

**ATOM 381 CA VAL R 285 119.399 94.492 128.567 1.00 0.00 C**

**ATOM 382 C VAL R 285 119.259 95.954 128.995 1.00 0.00 C**

**ATOM 383 O VAL R 285 119.698 96.332 130.089 1.00 0.00 O**

**ATOM 384 N VAL R 285 118.242 94.043 127.783 1.00 0.00 N**

**ATOM 385 CB VAL R 285 120.696 94.211 127.765 1.00 0.00 C**

**ATOM 386 CG1 VAL R 285 121.929 94.867 128.375 1.00 0.00 C**

**ATOM 387 CG2 VAL R 285 120.953 92.710 127.719 1.00 0.00 C**

**ATOM 388 CA VAL R 296 112.697 96.582 144.492 1.00 0.00 C**

**ATOM 389 C VAL R 296 112.729 97.723 145.503 1.00 0.00 C**

**ATOM 390 O VAL R 296 112.342 97.548 146.666 1.00 0.00 O**

**ATOM 391 N VAL R 296 112.106 97.028 143.226 1.00 0.00 N**

**ATOM 392 CB VAL R 296 114.095 95.976 144.259 1.00 0.00 C**

**ATOM 393 CG1 VAL R 296 114.802 95.715 145.577 1.00 0.00 C**

**ATOM 394 CG2 VAL R 296 113.972 94.656 143.519 1.00 0.00 C**

**ATOM 395 CA THR R 63 128.273 113.236 133.319 1.00 0.00 C**

**ATOM 396 C THR R 63 128.863 113.193 131.909 1.00 0.00 C**

**ATOM 397 O THR R 63 128.208 113.633 130.959 1.00 0.00 O**

**ATOM 398 N THR R 63 128.786 112.146 134.150 1.00 0.00 N**

**ATOM 399 CB THR R 63 128.488 114.605 133.990 1.00 0.00 C**

**ATOM 400 OG1 THR R 63 127.702 115.593 133.311 1.00 0.00 O**

**ATOM 401 CG2 THR R 63 129.939 115.058 133.965 1.00 0.00 C**

**ATOM 402 CA THR R 88 112.774 109.935 101.099 1.00 0.00 C**

**ATOM 403 C THR R 88 111.347 110.328 101.446 1.00 0.00 C**

**ATOM 404 O THR R 88 111.038 110.581 102.614 1.00 0.00 O**

**ATOM 405 N THR R 88 113.686 111.010 101.475 1.00 0.00 N**

**ATOM 406 CB THR R 88 113.147 108.629 101.806 1.00 0.00 C**

**ATOM 407 OG1 THR R 88 114.458 108.221 101.399 1.00 0.00 O**

**ATOM 408 CG2 THR R 88 112.151 107.533 101.460 1.00 0.00 C**

**ATOM 409 CA THR R 92 105.557 103.342 102.563 1.00 0.00 C**

**ATOM 410 C THR R 92 105.158 103.515 104.026 1.00 0.00 C**

**ATOM 411 O THR R 92 105.228 104.608 104.591 1.00 0.00 O**

**ATOM 412 N THR R 92 105.702 104.643 101.932 1.00 0.00 N**

**ATOM 413 CB THR R 92 106.858 102.539 102.455 1.00 0.00 C**

**ATOM 414 OG1 THR R 92 107.919 103.260 103.094 1.00 0.00 O**

**ATOM 415 CG2 THR R 92 107.220 102.305 100.996 1.00 0.00 C**

**ATOM 416 CA THR R 94 107.817 101.457 107.326 1.00 0.00 C**

**ATOM 417 C THR R 94 108.487 102.800 107.621 1.00 0.00 C**

**ATOM 418 O THR R 94 108.897 103.050 108.759 1.00 0.00 O**

**ATOM 419 N THR R 94 106.590 101.616 106.547 1.00 0.00 N**

**ATOM 420 CB THR R 94 108.761 100.456 106.632 1.00 0.00 C**

**ATOM 421 OG1 THR R 94 109.977 100.321 107.382 1.00 0.00 O**

**ATOM 422 CG2 THR R 94 109.038 100.775 105.148 1.00 0.00 C**

**ATOM 423 CA THR R 109 116.630 112.629 124.433 1.00 0.00 C**

**ATOM 424 C THR R 109 116.767 114.051 124.995 1.00 0.00 C**

**ATOM 425 O THR R 109 117.740 114.341 125.704 1.00 0.00 O**

**ATOM 426 N THR R 109 115.234 112.260 124.194 1.00 0.00 N**

**ATOM 427 CB THR R 109 117.397 112.449 123.107 1.00 0.00 C**

**ATOM 428 OG1 THR R 109 117.260 111.091 122.672 1.00 0.00 O**

**ATOM 429 CG2 THR R 109 118.896 112.729 123.234 1.00 0.00 C**

**ATOM 430 CA THR R 110 115.746 116.286 125.256 1.00 0.00 C**

**ATOM 431 C THR R 110 115.776 116.356 126.787 1.00 0.00 C**

**ATOM 432 O THR R 110 116.366 117.282 127.359 1.00 0.00 O**

**ATOM 433 N THR R 110 115.766 114.909 124.756 1.00 0.00 N**

**ATOM 434 CB THR R 110 114.502 116.990 124.691 1.00 0.00 C**

**ATOM 435 OG1 THR R 110 114.538 116.930 123.260 1.00 0.00 O**

**ATOM 436 CG2 THR R 110 114.428 118.461 125.100 1.00 0.00 C**

**ATOM 437 CA THR R 111 115.030 115.394 128.915 1.00 0.00 C**

**ATOM 438 C THR R 111 116.360 115.205 129.659 1.00 0.00 C**

**ATOM 439 O THR R 111 116.521 115.723 130.773 1.00 0.00 O**

**ATOM 440 N THR R 111 115.194 115.361 127.462 1.00 0.00 N**

**ATOM 441 CB THR R 111 114.000 114.314 129.292 1.00 0.00 C**

**ATOM 442 OG1 THR R 111 112.763 114.594 128.624 1.00 0.00 O**

**ATOM 443 CG2 THR R 111 113.710 114.254 130.787 1.00 0.00 C**

**ATOM 444 CA THR R 117 121.379 118.815 136.465 1.00 0.00 C**

**ATOM 445 C THR R 117 120.805 118.417 137.824 1.00 0.00 C**

**ATOM 446 O THR R 117 121.342 118.799 138.870 1.00 0.00 O**

**ATOM 447 N THR R 117 121.401 117.681 135.541 1.00 0.00 N**

**ATOM 448 CB THR R 117 120.579 119.965 135.846 1.00 0.00 C**

**ATOM 449 OG1 THR R 117 121.173 120.341 134.598 1.00 0.00 O**

**ATOM 450 CG2 THR R 117 120.563 121.181 136.765 1.00 0.00 C**

**ATOM 451 CA THR R 144 104.956 106.516 122.483 1.00 0.00 C**

**ATOM 452 C THR R 144 106.053 105.733 121.765 1.00 0.00 C**

**ATOM 453 O THR R 144 105.775 104.998 120.810 1.00 0.00 O**

**ATOM 454 N THR R 144 105.127 106.464 123.933 1.00 0.00 N**

**ATOM 455 CB THR R 144 104.941 107.975 122.021 1.00 0.00 C**

**ATOM 456 OG1 THR R 144 103.892 108.678 122.697 1.00 0.00 O**

**ATOM 457 CG2 THR R 144 104.710 108.075 120.521 1.00 0.00 C**

**ATOM 458 CA THR R 148 105.808 102.338 118.342 1.00 0.00 C**

**ATOM 459 C THR R 148 106.667 101.122 118.015 1.00 0.00 C**

**ATOM 460 O THR R 148 106.466 100.482 116.974 1.00 0.00 O**

**ATOM 461 N THR R 148 105.391 102.326 119.741 1.00 0.00 N**

**ATOM 462 CB THR R 148 106.561 103.637 118.038 1.00 0.00 C**

**ATOM 463 OG1 THR R 148 105.711 104.757 118.319 1.00 0.00 O**

**ATOM 464 CG2 THR R 148 106.984 103.697 116.577 1.00 0.00 C**

**ATOM 465 CA THR R 150 105.690 97.095 119.638 1.00 0.00 C**

**ATOM 466 C THR R 150 104.806 96.959 118.403 1.00 0.00 C**

**ATOM 467 O THR R 150 104.643 95.855 117.868 1.00 0.00 O**

**ATOM 468 N THR R 150 106.527 98.287 119.554 1.00 0.00 N**

**ATOM 469 CB THR R 150 104.842 97.155 120.911 1.00 0.00 C**

**ATOM 470 OG1 THR R 150 105.703 97.291 122.047 1.00 0.00 O**

**ATOM 471 CG2 THR R 150 104.008 95.895 121.076 1.00 0.00 C**

**ATOM 472 CA THR R 171 98.328 100.560 103.756 1.00 0.00 C**

**ATOM 473 C THR R 171 98.943 101.761 104.473 1.00 0.00 C**

**ATOM 474 O THR R 171 99.078 101.742 105.702 1.00 0.00 O**

**ATOM 475 N THR R 171 98.728 99.355 104.458 1.00 0.00 N**

**ATOM 476 CB THR R 171 96.794 100.662 103.751 1.00 0.00 C**

**ATOM 477 OG1 THR R 171 96.243 99.474 103.170 1.00 0.00 O**

**ATOM 478 CG2 THR R 171 96.273 101.867 102.971 1.00 0.00 C**

**ATOM 479 CA THR R 199 107.031 121.515 141.861 1.00 0.00 C**

**ATOM 480 C THR R 199 106.641 121.746 143.323 1.00 0.00 C**

**ATOM 481 O THR R 199 105.660 122.442 143.601 1.00 0.00 O**

**ATOM 482 N THR R 199 105.856 121.203 141.061 1.00 0.00 N**

**ATOM 483 CB THR R 199 107.702 122.758 141.265 1.00 0.00 C**

**ATOM 484 OG1 THR R 199 108.031 122.501 139.893 1.00 0.00 O**

**ATOM 485 CG2 THR R 199 108.958 123.094 141.976 1.00 0.00 C**

**ATOM 486 CA THR R 247 109.772 83.792 117.662 1.00 0.00 C**

**ATOM 487 C THR R 247 108.954 83.083 116.589 1.00 0.00 C**

**ATOM 488 O THR R 247 109.485 82.247 115.843 1.00 0.00 O**

**ATOM 489 N THR R 247 109.579 85.239 117.604 1.00 0.00 N**

**ATOM 490 CB THR R 247 109.407 83.272 119.053 1.00 0.00 C**

**ATOM 491 OG1 THR R 247 110.222 83.925 120.035 1.00 0.00 O**

**ATOM 492 CG2 THR R 247 109.635 81.771 119.138 1.00 0.00 C**

**ATOM 493 CA THR R 273 118.092 86.287 112.660 1.00 0.00 C**

**ATOM 494 C THR R 273 118.982 87.008 113.668 1.00 0.00 C**

**ATOM 495 O THR R 273 118.539 87.331 114.779 1.00 0.00 O**

**ATOM 496 N THR R 273 118.229 86.874 111.329 1.00 0.00 N**

**ATOM 497 CB THR R 273 118.427 84.793 112.602 1.00 0.00 C**

**ATOM 498 OG1 THR R 273 117.511 84.133 111.721 1.00 0.00 O**

**ATOM 499 CG2 THR R 273 118.331 84.155 113.981 1.00 0.00 C**

**ATOM 500 CA THR R 288 115.083 95.177 132.563 1.00 0.00 C**

**ATOM 501 C THR R 288 115.752 95.657 133.871 1.00 0.00 C**

**ATOM 502 O THR R 288 115.073 95.594 134.917 1.00 0.00 O**

**ATOM 503 N THR R 288 115.215 96.040 131.385 1.00 0.00 N**

**ATOM 504 CB THR R 288 115.573 93.776 132.146 1.00 0.00 C**

**ATOM 505 OG1 THR R 288 114.842 93.345 130.991 1.00 0.00 O**

**ATOM 506 CG2 THR R 288 115.374 92.767 133.242 1.00 0.00 C**

**ATOM 507 CA THR R 302 114.235 101.419 151.043 1.00 0.00 C**

**ATOM 508 C THR R 302 115.608 101.725 151.628 1.00 0.00 C**

**ATOM 509 O THR R 302 115.860 101.449 152.806 1.00 0.00 O**

**ATOM 510 N THR R 302 113.917 100.015 151.268 1.00 0.00 N**

**ATOM 511 CB THR R 302 113.177 102.329 151.672 1.00 0.00 C**

**ATOM 512 OG1 THR R 302 111.884 101.988 151.158 1.00 0.00 O**

**ATOM 513 CG2 THR R 302 113.470 103.790 151.358 1.00 0.00 C**

**ATOM 514 CA THR R 306 126.213 106.617 147.151 1.00 0.00 C**

**ATOM 515 C THR R 306 126.365 105.730 145.915 1.00 0.00 C**

**ATOM 516 O THR R 306 126.004 106.129 144.799 1.00 0.00 O**

**ATOM 517 N THR R 306 125.455 105.961 148.220 1.00 0.00 N**

**ATOM 518 CB THR R 306 127.586 107.043 147.676 1.00 0.00 C**

**ATOM 519 OG1 THR R 306 128.325 105.886 148.088 1.00 0.00 O**

**ATOM 520 CG2 THR R 306 127.436 107.991 148.857 1.00 0.00 C**

**ATOM 521 CA THR R 321 121.067 104.775 125.805 1.00 0.00 C**

**ATOM 522 C THR R 321 120.391 103.910 124.749 1.00 0.00 C**

**ATOM 523 O THR R 321 120.783 103.936 123.578 1.00 0.00 O**

**ATOM 524 N THR R 321 120.095 105.234 126.792 1.00 0.00 N**

**ATOM 525 CB THR R 321 122.205 104.022 126.509 1.00 0.00 C**

**ATOM 526 OG1 THR R 321 122.806 104.878 127.487 1.00 0.00 O**

**ATOM 527 CG2 THR R 321 123.299 103.585 125.535 1.00 0.00 C**

**ATOM 528 CA GLY R 73 121.170 110.434 120.262 1.00 0.00 C**

**ATOM 529 C GLY R 73 120.549 111.601 119.518 1.00 0.00 C**

**ATOM 530 O GLY R 73 119.721 111.401 118.623 1.00 0.00 O**

**ATOM 531 N GLY R 73 122.593 110.604 120.513 1.00 0.00 N**

**ATOM 532 CA GLY R 76 121.216 110.375 115.187 1.00 0.00 C**

**ATOM 533 C GLY R 76 119.770 110.644 114.814 1.00 0.00 C**

**ATOM 534 O GLY R 76 119.397 110.505 113.639 1.00 0.00 O**

**ATOM 535 N GLY R 76 121.896 111.494 115.817 1.00 0.00 N**

**ATOM 536 CA GLY R 127 110.110 127.204 137.215 1.00 0.00 C**

**ATOM 537 C GLY R 127 109.291 125.927 137.192 1.00 0.00 C**

**ATOM 538 O GLY R 127 109.770 124.837 136.877 1.00 0.00 O**

**ATOM 539 N GLY R 127 111.510 126.994 136.918 1.00 0.00 N**

**ATOM 540 CA GLY R 190 100.274 112.962 128.958 1.00 0.00 C**

**ATOM 541 C GLY R 190 99.457 114.000 129.711 1.00 0.00 C**

**ATOM 542 O GLY R 190 100.009 114.850 130.415 1.00 0.00 O**

**ATOM 543 N GLY R 190 99.774 111.602 129.135 1.00 0.00 N**

**ATOM 544 CA GLY R 197 103.096 117.071 137.586 1.00 0.00 C**

**ATOM 545 C GLY R 197 103.179 118.464 138.190 1.00 0.00 C**

**ATOM 546 O GLY R 197 102.323 119.319 137.950 1.00 0.00 O**

**ATOM 547 N GLY R 197 101.724 116.592 137.490 1.00 0.00 N**

**ATOM 548 CA GLY R 198 104.411 119.994 139.591 1.00 0.00 C**

**ATOM 549 C GLY R 198 105.657 120.037 140.451 1.00 0.00 C**

**ATOM 550 O GLY R 198 106.435 119.076 140.526 1.00 0.00 O**

**ATOM 551 N GLY R 198 104.223 118.690 138.981 1.00 0.00 N**

**ATOM 552 CA GLY R 261 117.106 77.027 98.812 1.00 0.00 C**

**ATOM 553 C GLY R 261 118.600 77.003 98.543 1.00 0.00 C**

**ATOM 554 O GLY R 261 119.106 76.084 97.894 1.00 0.00 O**

**ATOM 555 N GLY R 261 116.756 77.457 100.158 1.00 0.00 N**

**ATOM 556 CA GLY R 300 111.205 95.325 148.819 1.00 0.00 C**

**ATOM 557 C GLY R 300 111.970 95.978 149.958 1.00 0.00 C**

**ATOM 558 O GLY R 300 111.885 95.536 151.107 1.00 0.00 O**

**ATOM 559 N GLY R 300 110.382 96.242 148.034 1.00 0.00 N**

**ATOM 560 CA GLY R 319 117.478 103.244 129.499 1.00 0.00 C**

**ATOM 561 C GLY R 319 117.384 104.304 128.418 1.00 0.00 C**

**ATOM 562 O GLY R 319 116.729 104.103 127.392 1.00 0.00 O**

**ATOM 563 N GLY R 319 118.816 103.125 130.063 1.00 0.00 N**

**ATOM 564 CA PHE R 70 122.872 112.923 124.610 1.00 0.00 C**

**ATOM 565 C PHE R 70 123.235 113.395 123.205 1.00 0.00 C**

**ATOM 566 O PHE R 70 122.356 113.533 122.343 1.00 0.00 O**

**ATOM 567 N PHE R 70 123.715 111.806 125.041 1.00 0.00 N**

**ATOM 568 CB PHE R 70 122.987 114.079 125.607 1.00 0.00 C**

**ATOM 569 CG PHE R 70 122.249 115.321 125.191 1.00 0.00 C**

**ATOM 570 CD1 PHE R 70 120.863 115.372 125.254 1.00 0.00 C**

**ATOM 571 CE1 PHE R 70 120.178 116.515 124.877 1.00 0.00 C**

**ATOM 572 CZ PHE R 70 120.879 117.625 124.438 1.00 0.00 C**

**ATOM 573 CE2 PHE R 70 122.261 117.589 124.377 1.00 0.00 C**

**ATOM 574 CD2 PHE R 70 122.939 116.443 124.755 1.00 0.00 C**

**ATOM 575 CA PHE R 82 115.978 115.124 108.155 1.00 0.00 C**

**ATOM 576 C PHE R 82 115.873 114.295 106.876 1.00 0.00 C**

**ATOM 577 O PHE R 82 115.324 114.766 105.868 1.00 0.00 O**

**ATOM 578 N PHE R 82 115.719 114.318 109.347 1.00 0.00 N**

**ATOM 579 CB PHE R 82 117.355 115.778 108.264 1.00 0.00 C**

**ATOM 580 CG PHE R 82 117.655 116.752 107.162 1.00 0.00 C**

**ATOM 581 CD1 PHE R 82 117.007 117.977 107.110 1.00 0.00 C**

**ATOM 582 CE1 PHE R 82 117.282 118.881 106.098 1.00 0.00 C**

**ATOM 583 CZ PHE R 82 118.216 118.565 105.127 1.00 0.00 C**

**ATOM 584 CE2 PHE R 82 118.873 117.347 105.170 1.00 0.00 C**

**ATOM 585 CD2 PHE R 82 118.592 116.448 106.185 1.00 0.00 C**

**ATOM 586 CA PHE R 99 109.579 108.718 111.405 1.00 0.00 C**

**ATOM 587 C PHE R 99 109.028 108.979 112.803 1.00 0.00 C**

**ATOM 588 O PHE R 99 109.562 109.812 113.548 1.00 0.00 O**

**ATOM 589 N PHE R 99 109.753 107.287 111.158 1.00 0.00 N**

**ATOM 590 CB PHE R 99 108.652 109.305 110.336 1.00 0.00 C**

**ATOM 591 CG PHE R 99 108.378 110.778 110.489 1.00 0.00 C**

**ATOM 592 CD1 PHE R 99 109.290 111.718 110.038 1.00 0.00 C**

**ATOM 593 CE1 PHE R 99 109.031 113.069 110.159 1.00 0.00 C**

**ATOM 594 CZ PHE R 99 107.848 113.497 110.733 1.00 0.00 C**

**ATOM 595 CE2 PHE R 99 106.923 112.572 111.178 1.00 0.00 C**

**ATOM 596 CD2 PHE R 99 107.185 111.221 111.047 1.00 0.00 C**

**ATOM 597 CA PHE R 114 117.738 118.227 133.101 1.00 0.00 C**

**ATOM 598 C PHE R 114 117.937 117.196 134.205 1.00 0.00 C**

**ATOM 599 O PHE R 114 118.128 117.558 135.371 1.00 0.00 O**

**ATOM 600 N PHE R 114 118.464 117.849 131.892 1.00 0.00 N**

**ATOM 601 CB PHE R 114 116.255 118.407 132.787 1.00 0.00 C**

**ATOM 602 CG PHE R 114 115.972 119.529 131.834 1.00 0.00 C**

**ATOM 603 CD1 PHE R 114 115.976 120.844 132.275 1.00 0.00 C**

**ATOM 604 CE1 PHE R 114 115.718 121.884 131.402 1.00 0.00 C**

**ATOM 605 CZ PHE R 114 115.447 121.616 130.072 1.00 0.00 C**

**ATOM 606 CE2 PHE R 114 115.437 120.308 129.619 1.00 0.00 C**

**ATOM 607 CD2 PHE R 114 115.698 119.273 130.499 1.00 0.00 C**

**ATOM 608 CA PHE R 126 113.824 126.422 137.381 1.00 0.00 C**

**ATOM 609 C PHE R 126 112.382 126.610 137.850 1.00 0.00 C**

**ATOM 610 O PHE R 126 112.051 126.417 139.023 1.00 0.00 O**

**ATOM 611 N PHE R 126 114.639 125.874 138.452 1.00 0.00 N**

**ATOM 612 CB PHE R 126 113.907 125.526 136.144 1.00 0.00 C**

**ATOM 613 CG PHE R 126 115.309 125.243 135.695 1.00 0.00 C**

**ATOM 614 CD1 PHE R 126 116.089 126.252 135.150 1.00 0.00 C**

**ATOM 615 CE1 PHE R 126 117.383 126.000 134.731 1.00 0.00 C**

**ATOM 616 CZ PHE R 126 117.909 124.727 134.853 1.00 0.00 C**

**ATOM 617 CE2 PHE R 126 117.141 123.710 135.394 1.00 0.00 C**

**ATOM 618 CD2 PHE R 126 115.847 123.970 135.811 1.00 0.00 C**

**ATOM 619 CA PHE R 143 105.273 105.346 126.106 1.00 0.00 C**

**ATOM 620 C PHE R 143 105.026 105.308 124.601 1.00 0.00 C**

**ATOM 621 O PHE R 143 104.754 104.236 124.043 1.00 0.00 O**

**ATOM 622 N PHE R 143 106.325 106.302 126.451 1.00 0.00 N**

**ATOM 623 CB PHE R 143 103.978 105.669 126.852 1.00 0.00 C**

**ATOM 624 CG PHE R 143 104.034 105.380 128.325 1.00 0.00 C**

**ATOM 625 CD1 PHE R 143 104.951 104.473 128.836 1.00 0.00 C**

**ATOM 626 CE1 PHE R 143 104.994 104.199 130.190 1.00 0.00 C**

**ATOM 627 CZ PHE R 143 104.113 104.824 131.047 1.00 0.00 C**

**ATOM 628 CE2 PHE R 143 103.191 105.719 130.552 1.00 0.00 C**

**ATOM 629 CD2 PHE R 143 103.150 105.994 129.197 1.00 0.00 C**

**ATOM 630 CA PHE R 147 104.270 101.350 121.695 1.00 0.00 C**

**ATOM 631 C PHE R 147 104.556 101.384 120.197 1.00 0.00 C**

**ATOM 632 O PHE R 147 104.040 100.539 119.456 1.00 0.00 O**

**ATOM 633 N PHE R 147 105.402 101.842 122.476 1.00 0.00 N**

**ATOM 634 CB PHE R 147 103.011 102.152 122.023 1.00 0.00 C**

**ATOM 635 CG PHE R 147 102.509 101.950 123.427 1.00 0.00 C**

**ATOM 636 CD1 PHE R 147 102.753 100.762 124.103 1.00 0.00 C**

**ATOM 637 CE1 PHE R 147 102.292 100.573 125.394 1.00 0.00 C**

**ATOM 638 CZ PHE R 147 101.578 101.577 126.024 1.00 0.00 C**

**ATOM 639 CE2 PHE R 147 101.327 102.766 125.362 1.00 0.00 C**

**ATOM 640 CD2 PHE R 147 101.791 102.947 124.069 1.00 0.00 C**

**ATOM 641 CA PHE R 169 97.060 95.787 106.464 1.00 0.00 C**

**ATOM 642 C PHE R 169 98.192 96.793 106.278 1.00 0.00 C**

**ATOM 643 O PHE R 169 98.332 97.725 107.077 1.00 0.00 O**

**ATOM 644 N PHE R 169 96.611 95.248 105.186 1.00 0.00 N**

**ATOM 645 CB PHE R 169 97.493 94.647 107.394 1.00 0.00 C**

**ATOM 646 CG PHE R 169 97.918 95.101 108.765 1.00 0.00 C**

**ATOM 647 CD1 PHE R 169 96.970 95.347 109.747 1.00 0.00 C**

**ATOM 648 CE1 PHE R 169 97.349 95.759 111.013 1.00 0.00 C**

**ATOM 649 CZ PHE R 169 98.691 95.915 111.314 1.00 0.00 C**

**ATOM 650 CE2 PHE R 169 99.650 95.661 110.348 1.00 0.00 C**

**ATOM 651 CD2 PHE R 169 99.263 95.250 109.085 1.00 0.00 C**

**ATOM 652 CA PHE R 214 99.664 112.717 144.947 1.00 0.00 C**

**ATOM 653 C PHE R 214 99.279 113.157 146.356 1.00 0.00 C**

**ATOM 654 O PHE R 214 100.098 113.773 147.050 1.00 0.00 O**

**ATOM 655 N PHE R 214 100.800 113.536 144.561 1.00 0.00 N**

**ATOM 656 CB PHE R 214 100.022 111.219 144.933 1.00 0.00 C**

**ATOM 657 CG PHE R 214 100.384 110.676 143.579 1.00 0.00 C**

**ATOM 658 CD1 PHE R 214 99.900 111.260 142.418 1.00 0.00 C**

**ATOM 659 CE1 PHE R 214 100.238 110.756 141.180 1.00 0.00 C**

**ATOM 660 CZ PHE R 214 101.070 109.658 141.092 1.00 0.00 C**

**ATOM 661 CE2 PHE R 214 101.561 109.064 142.238 1.00 0.00 C**

**ATOM 662 CD2 PHE R 214 101.218 109.574 143.473 1.00 0.00 C**

**ATOM 663 CA PHE R 225 99.962 101.444 142.299 1.00 0.00 C**

**ATOM 664 C PHE R 225 100.329 101.968 140.913 1.00 0.00 C**

**ATOM 665 O PHE R 225 100.263 101.223 139.924 1.00 0.00 O**

**ATOM 666 N PHE R 225 100.959 101.815 143.304 1.00 0.00 N**

**ATOM 667 CB PHE R 225 98.583 101.952 142.728 1.00 0.00 C**

**ATOM 668 CG PHE R 225 97.477 101.615 141.763 1.00 0.00 C**

**ATOM 669 CD1 PHE R 225 96.893 100.356 141.773 1.00 0.00 C**

**ATOM 670 CE1 PHE R 225 95.870 100.042 140.893 1.00 0.00 C**

**ATOM 671 CZ PHE R 225 95.410 100.996 140.002 1.00 0.00 C**

**ATOM 672 CE2 PHE R 225 95.974 102.259 139.990 1.00 0.00 C**

**ATOM 673 CD2 PHE R 225 96.996 102.566 140.872 1.00 0.00 C**

**ATOM 674 CA PHE R 231 105.799 98.626 134.814 1.00 0.00 C**

**ATOM 675 C PHE R 231 105.113 97.534 134.001 1.00 0.00 C**

**ATOM 676 O PHE R 231 105.672 97.068 133.003 1.00 0.00 O**

**ATOM 677 N PHE R 231 104.902 99.753 135.072 1.00 0.00 N**

**ATOM 678 CB PHE R 231 106.350 98.057 136.129 1.00 0.00 C**

**ATOM 679 CG PHE R 231 107.032 96.710 135.977 1.00 0.00 C**

**ATOM 680 CD1 PHE R 231 108.355 96.640 135.565 1.00 0.00 C**

**ATOM 681 CE1 PHE R 231 108.994 95.416 135.426 1.00 0.00 C**

**ATOM 682 CZ PHE R 231 108.319 94.245 135.730 1.00 0.00 C**

**ATOM 683 CE2 PHE R 231 107.005 94.299 136.165 1.00 0.00 C**

**ATOM 684 CD2 PHE R 231 106.373 95.524 136.304 1.00 0.00 C**

**ATOM 685 CA PHE R 233 101.786 98.062 130.727 1.00 0.00 C**

**ATOM 686 C PHE R 233 102.950 98.398 129.800 1.00 0.00 C**

**ATOM 687 O PHE R 233 102.879 98.124 128.596 1.00 0.00 O**

**ATOM 688 N PHE R 233 102.231 97.651 132.059 1.00 0.00 N**

**ATOM 689 CB PHE R 233 100.811 99.237 130.828 1.00 0.00 C**

**ATOM 690 CG PHE R 233 99.427 98.838 131.267 1.00 0.00 C**

**ATOM 691 CD1 PHE R 233 98.707 97.899 130.541 1.00 0.00 C**

**ATOM 692 CE1 PHE R 233 97.432 97.525 130.931 1.00 0.00 C**

**ATOM 693 CZ PHE R 233 96.858 98.098 132.051 1.00 0.00 C**

**ATOM 694 CE2 PHE R 233 97.562 99.042 132.781 1.00 0.00 C**

**ATOM 695 CD2 PHE R 233 98.837 99.411 132.385 1.00 0.00 C**

**ATOM 696 CA PHE R 235 107.252 96.270 130.085 1.00 0.00 C**

**ATOM 697 C PHE R 235 106.738 94.867 129.806 1.00 0.00 C**

**ATOM 698 O PHE R 235 107.219 94.232 128.865 1.00 0.00 O**

**ATOM 699 N PHE R 235 106.197 97.268 130.163 1.00 0.00 N**

**ATOM 700 CB PHE R 235 108.076 96.289 131.385 1.00 0.00 C**

**ATOM 701 CG PHE R 235 109.269 95.372 131.367 1.00 0.00 C**

**ATOM 702 CD1 PHE R 235 110.436 95.758 130.721 1.00 0.00 C**

**ATOM 703 CE1 PHE R 235 111.542 94.929 130.702 1.00 0.00 C**

**ATOM 704 CZ PHE R 235 111.496 93.703 131.341 1.00 0.00 C**

**ATOM 705 CE2 PHE R 235 110.342 93.309 131.997 1.00 0.00 C**

**ATOM 706 CD2 PHE R 235 109.238 94.145 132.012 1.00 0.00 C**

**ATOM 707 CA PHE R 283 115.736 96.134 124.452 1.00 0.00 C**

**ATOM 708 C PHE R 283 115.634 95.530 125.855 1.00 0.00 C**

**ATOM 709 O PHE R 283 115.345 96.248 126.825 1.00 0.00 O**

**ATOM 710 N PHE R 283 116.939 95.680 123.749 1.00 0.00 N**

**ATOM 711 CB PHE R 283 114.497 95.786 123.624 1.00 0.00 C**

**ATOM 712 CG PHE R 283 113.217 96.384 124.146 1.00 0.00 C**

**ATOM 713 CD1 PHE R 283 112.868 97.688 123.827 1.00 0.00 C**

**ATOM 714 CE1 PHE R 283 111.687 98.242 124.295 1.00 0.00 C**

**ATOM 715 CZ PHE R 283 110.840 97.489 125.088 1.00 0.00 C**

**ATOM 716 CE2 PHE R 283 111.172 96.185 125.410 1.00 0.00 C**

**ATOM 717 CD2 PHE R 283 112.352 95.636 124.935 1.00 0.00 C**

**ATOM 718 CA PHE R 293 115.626 98.331 140.535 1.00 0.00 C**

**ATOM 719 C PHE R 293 114.563 99.234 141.162 1.00 0.00 C**

**ATOM 720 O PHE R 293 114.461 99.330 142.396 1.00 0.00 O**

**ATOM 721 N PHE R 293 115.066 97.488 139.478 1.00 0.00 N**

**ATOM 722 CB PHE R 293 116.780 99.150 139.953 1.00 0.00 C**

**ATOM 723 CG PHE R 293 117.706 99.721 140.981 1.00 0.00 C**

**ATOM 724 CD1 PHE R 293 118.583 98.899 141.672 1.00 0.00 C**

**ATOM 725 CE1 PHE R 293 119.449 99.423 142.613 1.00 0.00 C**

**ATOM 726 CZ PHE R 293 119.454 100.780 142.861 1.00 0.00 C**

**ATOM 727 CE2 PHE R 293 118.592 101.607 142.168 1.00 0.00 C**

**ATOM 728 CD2 PHE R 293 117.727 101.080 141.233 1.00 0.00 C**

**ATOM 729 CA PHE R 314 123.650 104.467 135.408 1.00 0.00 C**

**ATOM 730 C PHE R 314 122.614 103.617 134.679 1.00 0.00 C**

**ATOM 731 O PHE R 314 122.649 103.508 133.449 1.00 0.00 O**

**ATOM 732 N PHE R 314 123.042 105.208 136.508 1.00 0.00 N**

**ATOM 733 CB PHE R 314 124.800 103.608 135.938 1.00 0.00 C**

**ATOM 734 CG PHE R 314 125.506 102.807 134.880 1.00 0.00 C**

**ATOM 735 CD1 PHE R 314 126.435 103.413 134.046 1.00 0.00 C**

**ATOM 736 CE1 PHE R 314 127.100 102.685 133.075 1.00 0.00 C**

**ATOM 737 CZ PHE R 314 126.852 101.331 132.939 1.00 0.00 C**

**ATOM 738 CE2 PHE R 314 125.939 100.709 133.773 1.00 0.00 C**

**ATOM 739 CD2 PHE R 314 125.274 101.445 134.741 1.00 0.00 C**

**ATOM 740 CA PHE R 332 121.172 102.493 109.541 1.00 0.00 C**

**ATOM 741 C PHE R 332 121.448 100.995 109.386 1.00 0.00 C**

**ATOM 742 O PHE R 332 122.609 100.614 109.194 1.00 0.00 O**

**ATOM 743 N PHE R 332 119.892 102.788 110.175 1.00 0.00 N**

**ATOM 744 CB PHE R 332 122.307 103.153 110.329 1.00 0.00 C**

**ATOM 745 CG PHE R 332 122.274 104.653 110.294 1.00 0.00 C**

**ATOM 746 CD1 PHE R 332 122.740 105.346 109.186 1.00 0.00 C**

**ATOM 747 CE1 PHE R 332 122.708 106.730 109.150 1.00 0.00 C**

**ATOM 748 CZ PHE R 332 122.204 107.435 110.230 1.00 0.00 C**

**ATOM 749 CE2 PHE R 332 121.735 106.756 111.341 1.00 0.00 C**

**ATOM 750 CD2 PHE R 332 121.771 105.372 111.368 1.00 0.00 C**

**ATOM 751 CA PHE R 337 117.259 102.929 103.946 1.00 0.00 C**

**ATOM 752 C PHE R 337 118.674 103.175 103.445 1.00 0.00 C**

**ATOM 753 O PHE R 337 119.049 104.325 103.197 1.00 0.00 O**

**ATOM 754 N PHE R 337 116.791 101.610 103.537 1.00 0.00 N**

**ATOM 755 CB PHE R 337 117.206 103.062 105.469 1.00 0.00 C**

**ATOM 756 CG PHE R 337 117.492 104.454 105.976 1.00 0.00 C**

**ATOM 757 CD1 PHE R 337 116.485 105.405 106.000 1.00 0.00 C**

**ATOM 758 CE1 PHE R 337 116.728 106.682 106.467 1.00 0.00 C**

**ATOM 759 CZ PHE R 337 117.996 107.031 106.900 1.00 0.00 C**

**ATOM 760 CE2 PHE R 337 119.014 106.097 106.873 1.00 0.00 C**

**ATOM 761 CD2 PHE R 337 118.762 104.818 106.411 1.00 0.00 C**

**ATOM 762 CA PHE R 341 120.883 107.015 101.542 1.00 0.00 C**

**ATOM 763 C PHE R 341 121.967 107.119 100.476 1.00 0.00 C**

**ATOM 764 O PHE R 341 122.501 108.208 100.231 1.00 0.00 O**

**ATOM 765 N PHE R 341 119.797 106.138 101.114 1.00 0.00 N**

**ATOM 766 CB PHE R 341 121.466 106.502 102.860 1.00 0.00 C**

**ATOM 767 CG PHE R 341 122.553 107.369 103.430 1.00 0.00 C**

**ATOM 768 CD1 PHE R 341 122.247 108.577 104.040 1.00 0.00 C**

**ATOM 769 CE1 PHE R 341 123.247 109.376 104.571 1.00 0.00 C**

**ATOM 770 CZ PHE R 341 124.567 108.966 104.501 1.00 0.00 C**

**ATOM 771 CE2 PHE R 341 124.885 107.760 103.900 1.00 0.00 C**

**ATOM 772 CD2 PHE R 341 123.880 106.967 103.371 1.00 0.00 C**

**ATOM 773 CA MET R 81 114.351 112.921 110.823 1.00 0.00 C**

**ATOM 774 C MET R 81 114.519 113.766 109.567 1.00 0.00 C**

**ATOM 775 O MET R 81 113.578 113.894 108.780 1.00 0.00 O**

**ATOM 776 N MET R 81 115.518 112.075 111.062 1.00 0.00 N**

**ATOM 777 CB MET R 81 114.094 113.812 112.040 1.00 0.00 C**

**ATOM 778 CG MET R 81 113.359 113.127 113.185 1.00 0.00 C**

**ATOM 779 SD MET R 81 111.632 112.785 112.825 1.00 0.00 S**

**ATOM 780 CE MET R 81 110.992 114.455 112.821 1.00 0.00 C**

**ATOM 781 CA MET R 90 106.723 108.936 103.109 1.00 0.00 C**

**ATOM 782 C MET R 90 105.580 108.277 102.334 1.00 0.00 C**

**ATOM 783 O MET R 90 104.398 108.570 102.520 1.00 0.00 O**

**ATOM 784 N MET R 90 107.434 109.897 102.262 1.00 0.00 N**

**ATOM 785 CB MET R 90 106.208 109.617 104.370 1.00 0.00 C**

**ATOM 786 CG MET R 90 107.295 110.176 105.269 1.00 0.00 C**

**ATOM 787 SD MET R 90 106.639 110.794 106.829 1.00 0.00 S**

**ATOM 788 CE MET R 90 105.533 112.075 106.244 1.00 0.00 C**

**ATOM 789 CA MET R 112 118.594 114.109 129.644 1.00 0.00 C**

**ATOM 790 C MET R 112 119.418 115.178 130.383 1.00 0.00 C**

**ATOM 791 O MET R 112 119.860 114.885 131.509 1.00 0.00 O**

**ATOM 792 N MET R 112 117.336 114.544 129.021 1.00 0.00 N**

**ATOM 793 CB MET R 112 119.452 113.407 128.579 1.00 0.00 C**

**ATOM 794 CG MET R 112 120.574 112.567 129.145 1.00 0.00 C**

**ATOM 795 SD MET R 112 121.464 111.695 127.849 1.00 0.00 S**

**ATOM 796 CE MET R 112 120.127 110.847 127.026 1.00 0.00 C**

**ATOM 797 CA MET R 121 123.181 120.074 141.617 1.00 0.00 C**

**ATOM 798 C MET R 121 122.408 119.667 142.864 1.00 0.00 C**

**ATOM 799 O MET R 121 122.507 120.352 143.892 1.00 0.00 O**

**ATOM 800 N MET R 121 123.492 118.934 140.758 1.00 0.00 N**

**ATOM 801 CB MET R 121 122.396 121.123 140.830 1.00 0.00 C**

**ATOM 802 CG MET R 121 123.156 121.718 139.655 1.00 0.00 C**

**ATOM 803 SD MET R 121 124.643 122.601 140.164 1.00 0.00 S**

**ATOM 804 CE MET R 121 123.927 124.024 140.983 1.00 0.00 C**

**ATOM 805 CA MET R 142 108.593 107.204 126.329 1.00 0.00 C**

**ATOM 806 C MET R 142 107.584 106.098 126.035 1.00 0.00 C**

**ATOM 807 O MET R 142 107.924 105.075 125.417 1.00 0.00 O**

**ATOM 808 N MET R 142 108.035 108.515 126.006 1.00 0.00 N**

**ATOM 809 CB MET R 142 109.040 107.168 127.791 1.00 0.00 C**

**ATOM 810 CG MET R 142 109.645 105.843 128.225 1.00 0.00 C**

**ATOM 811 SD MET R 142 109.897 105.739 130.008 1.00 0.00 S**

**ATOM 812 CE MET R 142 108.208 105.786 130.592 1.00 0.00 C**

**ATOM 813 CA MET R 151 103.494 98.042 116.670 1.00 0.00 C**

**ATOM 814 C MET R 151 104.356 97.711 115.457 1.00 0.00 C**

**ATOM 815 O MET R 151 103.856 97.084 114.520 1.00 0.00 O**

**ATOM 816 N MET R 151 104.266 98.078 117.908 1.00 0.00 N**

**ATOM 817 CB MET R 151 102.731 99.347 116.478 1.00 0.00 C**

**ATOM 818 CG MET R 151 101.452 99.409 117.313 1.00 0.00 C**

**ATOM 819 SD MET R 151 100.290 98.056 117.006 1.00 0.00 S**

**ATOM 820 CE MET R 151 100.425 97.093 118.515 1.00 0.00 C**

**ATOM 821 CA MET R 152 106.506 97.714 114.339 1.00 0.00 C**

**ATOM 822 C MET R 152 106.757 96.211 114.378 1.00 0.00 C**

**ATOM 823 O MET R 152 106.801 95.556 113.324 1.00 0.00 O**

**ATOM 824 N MET R 152 105.628 98.131 115.435 1.00 0.00 N**

**ATOM 825 CB MET R 152 107.836 98.467 114.380 1.00 0.00 C**

**ATOM 826 CG MET R 152 107.740 99.939 114.057 1.00 0.00 C**

**ATOM 827 SD MET R 152 109.321 100.792 114.207 1.00 0.00 S**

**ATOM 828 CE MET R 152 110.184 100.157 112.773 1.00 0.00 C**

**ATOM 829 CA MET R 226 101.117 103.824 139.541 1.00 0.00 C**

**ATOM 830 C MET R 226 102.371 103.130 139.016 1.00 0.00 C**

**ATOM 831 O MET R 226 102.475 102.867 137.813 1.00 0.00 O**

**ATOM 832 N MET R 226 100.682 103.261 140.819 1.00 0.00 N**

**ATOM 833 CB MET R 226 101.354 105.332 139.658 1.00 0.00 C**

**ATOM 834 CG MET R 226 101.772 105.964 138.328 1.00 0.00 C**

**ATOM 835 SD MET R 226 101.883 107.755 138.262 1.00 0.00 S**

**ATOM 836 CE MET R 226 102.537 107.976 136.610 1.00 0.00 C**

**ATOM 837 CA MET R 249 108.415 84.996 112.677 1.00 0.00 C**

**ATOM 838 C MET R 249 109.584 84.138 112.209 1.00 0.00 C**

**ATOM 839 O MET R 249 109.629 83.717 111.048 1.00 0.00 O**

**ATOM 840 N MET R 249 107.878 84.513 113.946 1.00 0.00 N**

**ATOM 841 CB MET R 249 108.856 86.452 112.822 1.00 0.00 C**

**ATOM 842 CG MET R 249 109.316 87.104 111.531 1.00 0.00 C**

**ATOM 843 SD MET R 249 110.071 88.710 111.842 1.00 0.00 S**

**ATOM 844 CE MET R 249 108.663 89.629 112.451 1.00 0.00 C**

**ATOM 845 CA GLU R 203 111.644 122.981 154.182 1.00 0.00 C**

**ATOM 846 C GLU R 203 112.802 122.412 155.000 1.00 0.00 C**

**ATOM 847 O GLU R 203 113.819 123.090 155.184 1.00 0.00 O**

**ATOM 848 N GLU R 203 111.100 122.035 153.207 1.00 0.00 N**

**ATOM 849 CB GLU R 203 110.530 123.451 155.117 1.00 0.00 C**

**ATOM 850 CG GLU R 203 109.432 124.247 154.438 1.00 0.00 C**

**ATOM 851 CD GLU R 203 108.414 124.784 155.424 1.00 0.00 C**

**ATOM 852 OE1 GLU R 203 108.544 124.492 156.632 1.00 0.00 O**

**ATOM 853 OE2 GLU R 203 107.483 125.496 154.992 1.00 0.00 O**

**ATOM 854 CA GLU R 209 111.859 118.865 144.125 1.00 0.00 C**

**ATOM 855 C GLU R 209 110.752 119.027 143.084 1.00 0.00 C**

**ATOM 856 O GLU R 209 109.718 119.657 143.335 1.00 0.00 O**

**ATOM 857 N GLU R 209 111.978 120.026 145.001 1.00 0.00 N**

**ATOM 858 CB GLU R 209 111.651 117.582 144.944 1.00 0.00 C**

**ATOM 859 CG GLU R 209 110.344 117.480 145.699 1.00 0.00 C**

**ATOM 860 CD GLU R 209 110.202 116.164 146.439 1.00 0.00 C**

**ATOM 861 OE1 GLU R 209 111.117 115.320 146.336 1.00 0.00 O**

**ATOM 862 OE2 GLU R 209 109.177 115.973 147.126 1.00 0.00 O**

**ATOM 863 CA GLU R 264 123.852 82.498 100.310 1.00 0.00 C**

**ATOM 864 C GLU R 264 122.553 83.054 100.888 1.00 0.00 C**

**ATOM 865 O GLU R 264 122.573 83.978 101.714 1.00 0.00 O**

**ATOM 866 N GLU R 264 123.817 81.039 100.194 1.00 0.00 N**

**ATOM 867 CB GLU R 264 124.142 83.122 98.944 1.00 0.00 C**

**ATOM 868 CG GLU R 264 125.515 82.783 98.375 1.00 0.00 C**

**ATOM 869 CD GLU R 264 126.661 83.247 99.259 1.00 0.00 C**

**ATOM 870 OE1 GLU R 264 126.563 84.347 99.844 1.00 0.00 O**

**ATOM 871 OE2 GLU R 264 127.663 82.510 99.366 1.00 0.00 O**

**ATOM 872 CA GLU R 297 113.185 100.057 145.981 1.00 0.00 C**

**ATOM 873 C GLU R 297 111.764 100.480 146.352 1.00 0.00 C**

**ATOM 874 O GLU R 297 111.530 100.962 147.467 1.00 0.00 O**

**ATOM 875 N GLU R 297 113.201 98.903 145.082 1.00 0.00 N**

**ATOM 876 CB GLU R 297 113.975 101.220 145.392 1.00 0.00 C**

**ATOM 877 CG GLU R 297 115.467 100.964 145.417 1.00 0.00 C**

**ATOM 878 CD GLU R 297 116.271 102.189 145.068 1.00 0.00 C**

**ATOM 879 OE1 GLU R 297 115.663 103.252 144.823 1.00 0.00 O**

**ATOM 880 OE2 GLU R 297 117.515 102.108 145.093 1.00 0.00 O**

**ATOM 881 CA GLU R 335 118.502 97.417 103.438 1.00 0.00 C**

**ATOM 882 C GLU R 335 117.869 98.396 102.451 1.00 0.00 C**

**ATOM 883 O GLU R 335 118.448 98.637 101.387 1.00 0.00 O**

**ATOM 884 N GLU R 335 117.934 97.504 104.781 1.00 0.00 N**

**ATOM 885 CB GLU R 335 118.371 95.988 102.909 1.00 0.00 C**

**ATOM 886 CG GLU R 335 119.232 94.975 103.649 1.00 0.00 C**

**ATOM 887 CD GLU R 335 120.716 95.189 103.422 1.00 0.00 C**

**ATOM 888 OE1 GLU R 335 121.096 95.616 102.311 1.00 0.00 O**

**ATOM 889 OE2 GLU R 335 121.502 94.935 104.358 1.00 0.00 O**

**ATOM 890 CA ARG R 86 113.439 115.495 102.544 1.00 0.00 C**

**ATOM 891 C ARG R 86 113.926 114.409 101.594 1.00 0.00 C**

**ATOM 892 O ARG R 86 113.441 114.344 100.456 1.00 0.00 O**

**ATOM 893 N ARG R 86 113.121 115.028 103.891 1.00 0.00 N**

**ATOM 894 CB ARG R 86 114.485 116.622 102.618 1.00 0.00 C**

**ATOM 895 CG ARG R 86 114.861 117.204 101.262 1.00 0.00 C**

**ATOM 896 CD ARG R 86 113.709 117.998 100.664 1.00 0.00 C**

**ATOM 897 NE ARG R 86 114.061 118.598 99.379 1.00 0.00 N**

**ATOM 898 CZ ARG R 86 113.238 119.349 98.654 1.00 0.00 C**

**ATOM 899 NH2 ARG R 86 113.643 119.851 97.496 1.00 0.00 N**

**ATOM 900 NH1 ARG R 86 112.010 119.599 99.088 1.00 0.00 N**

**ATOM 901 CA ARG R 156 106.424 92.774 110.606 1.00 0.00 C**

**ATOM 902 C ARG R 156 105.990 91.328 110.845 1.00 0.00 C**

**ATOM 903 O ARG R 156 106.151 90.480 109.953 1.00 0.00 O**

**ATOM 904 N ARG R 156 105.566 93.722 111.315 1.00 0.00 N**

**ATOM 905 CB ARG R 156 107.885 92.991 111.003 1.00 0.00 C**

**ATOM 906 CG ARG R 156 108.474 94.270 110.420 1.00 0.00 C**

**ATOM 907 CD ARG R 156 109.979 94.371 110.623 1.00 0.00 C**

**ATOM 908 NE ARG R 156 110.326 94.659 112.012 1.00 0.00 N**

**ATOM 909 CZ ARG R 156 111.540 95.009 112.422 1.00 0.00 C**

**ATOM 910 NH2 ARG R 156 111.760 95.254 113.706 1.00 0.00 N**

**ATOM 911 NH1 ARG R 156 112.532 95.124 111.549 1.00 0.00 N**

**ATOM 912 CA ARG R 170 100.149 97.510 105.008 1.00 0.00 C**

**ATOM 913 C ARG R 170 99.773 98.675 104.085 1.00 0.00 C**

**ATOM 914 O ARG R 170 100.353 98.919 103.025 1.00 0.00 O**

**ATOM 915 N ARG R 170 99.001 96.628 105.234 1.00 0.00 N**

**ATOM 916 CB ARG R 170 101.341 96.707 104.487 1.00 0.00 C**

**ATOM 917 CG ARG R 170 102.694 97.445 104.519 1.00 0.00 C**

**ATOM 918 CD ARG R 170 103.859 96.550 104.155 1.00 0.00 C**

**ATOM 919 NE ARG R 170 104.254 95.724 105.290 1.00 0.00 N**

**ATOM 920 CZ ARG R 170 105.122 96.116 106.216 1.00 0.00 C**

**ATOM 921 NH2 ARG R 170 105.432 95.313 107.223 1.00 0.00 N**

**ATOM 922 NH1 ARG R 170 105.683 97.315 106.135 1.00 0.00 N**

**ATOM 923 CA ARG R 202 110.965 120.830 151.101 1.00 0.00 C**

**ATOM 924 C ARG R 202 111.751 121.695 152.084 1.00 0.00 C**

**ATOM 925 O ARG R 202 112.915 122.043 151.823 1.00 0.00 O**

**ATOM 926 N ARG R 202 110.798 121.579 149.862 1.00 0.00 N**

**ATOM 927 CB ARG R 202 111.679 119.507 150.826 1.00 0.00 C**

**ATOM 928 CG ARG R 202 111.748 118.582 152.010 1.00 0.00 C**

**ATOM 929 CD ARG R 202 110.401 117.949 152.321 1.00 0.00 C**

**ATOM 930 NE ARG R 202 110.481 117.017 153.443 1.00 0.00 N**

**ATOM 931 CZ ARG R 202 110.852 115.744 153.343 1.00 0.00 C**

**ATOM 932 NH2 ARG R 202 111.181 115.233 152.164 1.00 0.00 N**

**ATOM 933 NH1 ARG R 202 110.894 114.979 154.425 1.00 0.00 N**

**ATOM 934 CA ARG R 252 107.754 80.713 109.952 1.00 0.00 C**

**ATOM 935 C ARG R 252 108.799 80.798 108.841 1.00 0.00 C**

**ATOM 936 O ARG R 252 108.678 80.113 107.817 1.00 0.00 O**

**ATOM 937 N ARG R 252 108.381 80.687 111.270 1.00 0.00 N**

**ATOM 938 CB ARG R 252 106.768 81.879 109.863 1.00 0.00 C**

**ATOM 939 CG ARG R 252 106.093 82.018 108.508 1.00 0.00 C**

**ATOM 940 CD ARG R 252 105.243 80.786 108.228 1.00 0.00 C**

**ATOM 941 NE ARG R 252 104.500 80.875 106.974 1.00 0.00 N**

**ATOM 942 CZ ARG R 252 104.956 80.437 105.805 1.00 0.00 C**

**ATOM 943 NH2 ARG R 252 104.211 80.552 104.715 1.00 0.00 N**

**ATOM 944 NH1 ARG R 252 106.155 79.876 105.728 1.00 0.00 N**

**ATOM 945 CA ARG R 257 111.639 77.074 100.607 1.00 0.00 C**

**ATOM 946 C ARG R 257 112.719 77.978 100.014 1.00 0.00 C**

**ATOM 947 O ARG R 257 113.828 77.514 99.723 1.00 0.00 O**

**ATOM 948 N ARG R 257 110.805 77.798 101.566 1.00 0.00 N**

**ATOM 949 CB ARG R 257 110.744 76.452 99.525 1.00 0.00 C**

**ATOM 950 CG ARG R 257 111.400 75.439 98.563 1.00 0.00 C**

**ATOM 951 CD ARG R 257 112.004 76.045 97.293 1.00 0.00 C**

**ATOM 952 NE ARG R 257 112.598 75.039 96.411 1.00 0.00 N**

**ATOM 953 CZ ARG R 257 111.915 74.228 95.606 1.00 0.00 C**

**ATOM 954 NH2 ARG R 257 112.563 73.353 94.849 1.00 0.00 N**

**ATOM 955 NH1 ARG R 257 110.590 74.282 95.551 1.00 0.00 N**

**ATOM 956 CA ARG R 263 123.699 78.746 101.053 1.00 0.00 C**

**ATOM 957 C ARG R 263 123.743 80.257 101.277 1.00 0.00 C**

**ATOM 958 O ARG R 263 123.738 80.717 102.425 1.00 0.00 O**

**ATOM 959 N ARG R 263 122.823 78.382 99.942 1.00 0.00 N**

**ATOM 960 CB ARG R 263 125.102 78.183 100.805 1.00 0.00 C**

**ATOM 961 CG ARG R 263 126.177 78.666 101.770 1.00 0.00 C**

**ATOM 962 CD ARG R 263 125.929 78.120 103.170 1.00 0.00 C**

**ATOM 963 NE ARG R 263 126.955 78.536 104.123 1.00 0.00 N**

**ATOM 964 CZ ARG R 263 128.096 77.886 104.323 1.00 0.00 C**

**ATOM 965 NH2 ARG R 263 128.362 76.781 103.640 1.00 0.00 N**

**ATOM 966 NH1 ARG R 263 128.971 78.337 105.212 1.00 0.00 N**

**ATOM 967 CA ARG R 267 123.401 83.392 105.115 1.00 0.00 C**

**ATOM 968 C ARG R 267 122.600 84.670 105.355 1.00 0.00 C**

**ATOM 969 O ARG R 267 122.723 85.303 106.412 1.00 0.00 O**

**ATOM 970 N ARG R 267 122.584 82.361 104.473 1.00 0.00 N**

**ATOM 971 CB ARG R 267 124.639 83.678 104.261 1.00 0.00 C**

**ATOM 972 CG ARG R 267 125.555 84.764 104.806 1.00 0.00 C**

**ATOM 973 CD ARG R 267 126.774 84.950 103.915 1.00 0.00 C**

**ATOM 974 NE ARG R 267 127.669 85.988 104.419 1.00 0.00 N**

**ATOM 975 CZ ARG R 267 128.816 86.331 103.840 1.00 0.00 C**

**ATOM 976 NH2 ARG R 267 129.566 87.289 104.368 1.00 0.00 N**

**ATOM 977 NH1 ARG R 267 129.213 85.717 102.734 1.00 0.00 N**

**ATOM 978 CA ARG R 270 121.144 84.095 109.408 1.00 0.00 C**

**ATOM 979 C ARG R 270 121.388 85.541 109.830 1.00 0.00 C**

**ATOM 980 O ARG R 270 121.353 85.857 111.023 1.00 0.00 O**

**ATOM 981 N ARG R 270 120.320 84.020 108.203 1.00 0.00 N**

**ATOM 982 CB ARG R 270 122.471 83.373 109.168 1.00 0.00 C**

**ATOM 983 CG ARG R 270 123.425 83.376 110.350 1.00 0.00 C**

**ATOM 984 CD ARG R 270 122.872 82.557 111.502 1.00 0.00 C**

**ATOM 985 NE ARG R 270 122.689 81.156 111.131 1.00 0.00 N**

**ATOM 986 CZ ARG R 270 123.636 80.227 111.216 1.00 0.00 C**

**ATOM 987 NH2 ARG R 270 124.844 80.544 111.661 1.00 0.00 N**

**ATOM 988 NH1 ARG R 270 123.374 78.978 110.855 1.00 0.00 N**

**ATOM 989 CA ARG R 271 121.833 87.847 109.148 1.00 0.00 C**

**ATOM 990 C ARG R 271 120.561 88.479 109.709 1.00 0.00 C**

**ATOM 991 O ARG R 271 120.622 89.281 110.651 1.00 0.00 O**

**ATOM 992 N ARG R 271 121.643 86.425 108.859 1.00 0.00 N**

**ATOM 993 CB ARG R 271 122.287 88.580 107.885 1.00 0.00 C**

**ATOM 994 CG ARG R 271 122.553 90.064 108.080 1.00 0.00 C**

**ATOM 995 CD ARG R 271 122.922 90.743 106.769 1.00 0.00 C**

**ATOM 996 NE ARG R 271 121.825 90.712 105.807 1.00 0.00 N**

**ATOM 997 CZ ARG R 271 121.897 91.207 104.575 1.00 0.00 C**

**ATOM 998 NH2 ARG R 271 120.848 91.136 103.767 1.00 0.00 N**

**ATOM 999 NH1 ARG R 271 123.018 91.775 104.152 1.00 0.00 N**

**ATOM 1000 CA ARG R 274 121.203 87.980 114.114 1.00 0.00 C**

**ATOM 1001 C ARG R 274 120.664 89.371 114.428 1.00 0.00 C**

**ATOM 1002 O ARG R 274 120.781 89.840 115.563 1.00 0.00 O**

**ATOM 1003 N ARG R 274 120.263 87.196 113.313 1.00 0.00 N**

**ATOM 1004 CB ARG R 274 122.547 88.103 113.391 1.00 0.00 C**

**ATOM 1005 CG ARG R 274 123.397 86.842 113.357 1.00 0.00 C**

**ATOM 1006 CD ARG R 274 123.885 86.429 114.732 1.00 0.00 C**

**ATOM 1007 NE ARG R 274 124.647 85.185 114.671 1.00 0.00 N**

**ATOM 1008 CZ ARG R 274 124.139 83.981 114.914 1.00 0.00 C**

**ATOM 1009 NH2 ARG R 274 124.911 82.906 114.832 1.00 0.00 N**

**ATOM 1010 NH1 ARG R 274 122.860 83.851 115.238 1.00 0.00 N**

**ATOM 1011 CA ARG R 339 119.697 102.377 99.104 1.00 0.00 C**

**ATOM 1012 C ARG R 339 119.030 103.732 98.913 1.00 0.00 C**

**ATOM 1013 O ARG R 339 119.243 104.371 97.878 1.00 0.00 O**

**ATOM 1014 N ARG R 339 119.845 102.022 100.512 1.00 0.00 N**

**ATOM 1015 CB ARG R 339 118.891 101.307 98.366 1.00 0.00 C**

**ATOM 1016 CG ARG R 339 119.554 99.944 98.299 1.00 0.00 C**

**ATOM 1017 CD ARG R 339 118.657 98.949 97.579 1.00 0.00 C**

**ATOM 1018 NE ARG R 339 119.239 97.612 97.528 1.00 0.00 N**

**ATOM 1019 CZ ARG R 339 118.630 96.556 96.999 1.00 0.00 C**

**ATOM 1020 NH2 ARG R 339 119.232 95.374 96.994 1.00 0.00 N**

**ATOM 1021 NH1 ARG R 339 117.420 96.681 96.471 1.00 0.00 N**

**ATOM 1022 CA ARG R 342 123.334 105.992 98.803 1.00 0.00 C**

**ATOM 1023 C ARG R 342 122.823 106.615 97.508 1.00 0.00 C**

**ATOM 1024 O ARG R 342 123.193 107.736 97.159 1.00 0.00 O**

**ATOM 1025 N ARG R 342 122.306 106.004 99.838 1.00 0.00 N**

**ATOM 1026 CB ARG R 342 123.819 104.563 98.545 1.00 0.00 C**

**ATOM 1027 CG ARG R 342 124.563 103.938 99.716 1.00 0.00 C**

**ATOM 1028 CD ARG R 342 125.045 102.536 99.382 1.00 0.00 C**

**ATOM 1029 NE ARG R 342 123.936 101.606 99.192 1.00 0.00 N**

**ATOM 1030 CZ ARG R 342 124.073 100.357 98.761 1.00 0.00 C**

**ATOM 1031 NH2 ARG R 342 123.007 99.581 98.618 1.00 0.00 N**

**ATOM 1032 NH1 ARG R 342 125.277 99.881 98.474 1.00 0.00 N**

**ATOM 1033 N N.A X -1 114.422 110.696 143.268 1.00 0.00 N**

**ATOM 1034 C N.A X -1 113.659 109.772 142.579 1.00 0.00 C**

**ATOM 1035 O N.A X -1 114.104 109.037 141.695 1.00 0.00 O**

**ATOM 1036 C N.A X -1 112.164 109.965 142.912 1.00 0.00 C**

**ATOM 1037 N N.A X -1 111.593 110.342 141.626 1.00 0.00 N**

**ATOM 1038 C N.A X -1 111.371 111.665 141.298 1.00 0.00 C**

**ATOM 1039 O N.A X -1 111.074 112.513 142.139 1.00 0.00 O**

**ATOM 1040 C N.A X -1 111.685 112.032 139.835 1.00 0.00 C**

**ATOM 1041 N N.A X -1 110.718 111.505 138.883 1.00 0.00 N**

**ATOM 1042 C N.A X -1 110.956 110.673 137.822 1.00 0.00 C**

**ATOM 1043 O N.A X -1 110.186 110.763 136.860 1.00 0.00 O**

**ATOM 1044 C N.A X -1 111.954 109.506 138.006 1.00 0.00 C**

**ATOM 1045 N N.A X -1 110.971 108.412 138.014 1.00 0.00 N**

**ATOM 1046 C N.A X -1 110.301 108.115 139.185 1.00 0.00 C**

**ATOM 1047 O N.A X -1 110.837 108.271 140.281 1.00 0.00 O**

**ATOM 1048 C N.A X -1 108.781 107.976 139.008 1.00 0.00 C**

**ATOM 1049 N N.A X -1 108.425 107.175 137.847 1.00 0.00 N**

**ATOM 1050 C N.A X -1 108.580 105.809 137.841 1.00 0.00 C**

**ATOM 1051 O N.A X -1 109.197 105.210 138.721 1.00 0.00 O**

**ATOM 1052 C N.A X -1 107.856 105.096 136.691 1.00 0.00 C**

**ATOM 1053 N N.A X -1 108.763 104.329 135.846 1.00 0.00 N**

**ATOM 1054 C N.A X -1 109.402 104.758 134.698 1.00 0.00 C**

**ATOM 1055 O N.A X -1 110.227 104.059 134.105 1.00 0.00 O**

**ATOM 1056 C N.A X -1 108.915 106.080 134.116 1.00 0.00 C**

**ATOM 1057 N N.A X -1 109.951 106.851 133.486 1.00 0.00 N**

**ATOM 1058 C N.A X -1 110.581 107.850 134.202 1.00 0.00 C**

**ATOM 1059 O N.A X -1 110.414 107.990 135.414 1.00 0.00 O**

**ATOM 1060 C N.A X -1 111.459 108.754 133.329 1.00 0.00 C**

**ATOM 1061 N N.A X -1 111.325 110.153 133.767 1.00 0.00 N**

**ATOM 1062 C N.A X -1 112.920 108.313 133.443 1.00 0.00 C**

**ATOM 1063 C N.A X -1 113.309 107.201 132.507 1.00 0.00 C**

**ATOM 1064 C N.A X -1 113.129 107.348 131.124 1.00 0.00 C**

**ATOM 1065 C N.A X -1 113.621 106.392 130.249 1.00 0.00 C**

**ATOM 1066 C N.A X -1 114.258 105.275 130.762 1.00 0.00 C**

**ATOM 1067 O N.A X -1 114.710 104.357 129.870 1.00 0.00 O**

**ATOM 1068 C N.A X -1 114.423 105.086 132.129 1.00 0.00 C**

**ATOM 1069 C N.A X -1 113.952 106.059 133.004 1.00 0.00 C**

**ATOM 1070 C N.A X -1 108.859 102.947 136.287 1.00 0.00 C**

**ATOM 1071 C N.A X -1 107.846 102.806 137.416 1.00 0.00 C**

**ATOM 1072 C N.A X -1 106.949 104.029 137.266 1.00 0.00 C**

**ATOM 1073 C N.A X -1 108.045 107.549 140.286 1.00 0.00 C**

**ATOM 1074 C N.A X -1 107.386 108.709 140.995 1.00 0.00 C**

**ATOM 1075 C N.A X -1 108.161 109.721 141.564 1.00 0.00 C**

**ATOM 1076 C N.A X -1 107.583 110.764 142.279 1.00 0.00 C**

**ATOM 1077 C N.A X -1 106.207 110.803 142.433 1.00 0.00 C**

**ATOM 1078 C N.A X -1 105.417 109.800 141.882 1.00 0.00 C**

**ATOM 1079 C N.A X -1 106.000 108.768 141.147 1.00 0.00 C**

**ATOM 1080 C N.A X -1 112.957 109.272 136.884 1.00 0.00 C**

**ATOM 1081 C N.A X -1 114.272 110.069 136.877 1.00 0.00 C**

**ATOM 1082 C N.A X -1 114.064 111.497 136.406 1.00 0.00 C**

**ATOM 1083 C N.A X -1 115.020 110.034 138.205 1.00 0.00 C**

**ATOM 1084 C N.A X -1 111.726 113.563 139.667 1.00 0.00 C**

**ATOM 1085 C N.A X -1 112.412 113.999 138.374 1.00 0.00 C**

**ATOM 1086 C N.A X -1 112.297 115.495 138.104 1.00 0.00 C**

**ATOM 1087 N N.A X -1 113.105 115.868 136.957 1.00 0.00 N**

**ATOM 1088 C N.A X -1 112.795 115.566 135.670 1.00 0.00 C**

**ATOM 1089 N N.A X -1 111.765 114.884 135.305 1.00 0.00 N**

**ATOM 1090 N N.A X -1 113.695 116.050 134.787 1.00 0.00 N**

**ATOM 1091 C N.A X -1 111.530 108.751 143.576 1.00 0.00 C**

**ATOM 1092 C N.A X -1 110.305 109.124 144.433 1.00 0.00 C**

**ATOM 1093 C N.A X -1 110.604 109.518 145.883 1.00 0.00 C**

**ATOM 1094 N N.A X -1 111.361 110.746 146.005 1.00 0.00 N**

**ATOM 1095 C N.A X -1 110.809 111.990 146.054 1.00 0.00 C**

**ATOM 1096 N N.A X -1 109.544 112.238 145.951 1.00 0.00 N**

**ATOM 1097 N N.A X -1 111.717 112.972 146.235 1.00 0.00 N**

**ATOM 1098 C N.A X -1 115.636 111.264 142.721 1.00 0.00 C**

**ATOM 1099 C N.A X -1 116.895 110.664 143.342 1.00 0.00 C**

**ATOM 1100 O N.A X -1 118.009 110.923 142.873 1.00 0.00 O**

**ATOM 1101 N N.A X -1 116.717 110.024 144.548 1.00 0.00 N**

**ATOM 1102 C N.A X -1 117.757 110.078 145.559 1.00 0.00 C**

**ATOM 1103 C N.A X -1 117.825 108.883 146.543 1.00 0.00 C**

**ATOM 1104 O N.A X -1 118.198 109.088 147.708 1.00 0.00 O**

**ATOM 1105 N N.A X -1 117.712 107.578 146.087 1.00 0.00 N**

**ATOM 1106 C N.A X -1 117.194 107.016 144.820 1.00 0.00 C**

**ATOM 1107 C N.A X -1 115.642 106.936 144.918 1.00 0.00 C**

**ATOM 1108 O N.A X -1 115.006 107.997 144.860 1.00 0.00 O**

**ATOM 1109 N N.A X -1 115.057 105.722 145.228 1.00 0.00 N**

**ATOM 1110 C N.A X -1 113.651 105.487 145.591 1.00 0.00 C**

**ATOM 1111 C N.A X -1 112.746 105.136 144.402 1.00 0.00 C**

**ATOM 1112 O N.A X -1 112.923 105.467 143.238 1.00 0.00 O**

**ATOM 1113 O N.A X -1 111.621 104.448 144.775 1.00 0.00 O**

**ATOM 1114 C N.A X -1 112.976 106.564 146.455 1.00 0.00 C**

**ATOM 1115 C N.A X -1 113.179 106.368 147.953 1.00 0.00 C**

**ATOM 1116 C N.A X -1 111.988 105.687 148.632 1.00 0.00 C**

**ATOM 1117 C N.A X -1 111.849 104.197 148.339 1.00 0.00 C**

**ATOM 1118 N N.A X -1 111.177 103.884 147.058 1.00 0.00 N**

**ATOM 1119 C N.A X -1 117.956 105.703 144.688 1.00 0.00 C**

**ATOM 1120 C N.A X -1 118.121 105.229 146.128 1.00 0.00 C**

**ATOM 1121 C N.A X -1 118.230 106.512 146.932 1.00 0.00 C**

**ATOM 1122 C N.A X -1 117.612 111.448 146.283 1.00 0.00 C**

**ATOM 1123 C N.A X -1 118.851 112.043 146.967 1.00 0.00 C**

**ATOM 1124 C N.A X -1 120.067 112.284 146.077 1.00 0.00 C**

**ATOM 1125 N N.A X -1 119.856 113.304 145.064 1.00 0.00 N**

**ATOM 1126 C N.A X -1 120.526 113.267 143.881 1.00 0.00 C**

**ATOM 1127 N N.A X -1 120.347 112.364 142.983 1.00 0.00 N**

**ATOM 1128 N N.A X -1 121.449 114.235 143.744 1.00 0.00 N**

**ATOM 1129 C N.A X -1 115.641 112.776 143.020 1.00 0.00 C**

**ATOM 1130 C N.A X -1 114.468 113.526 142.372 1.00 0.00 C**

**ATOM 1131 C N.A X -1 114.097 114.801 143.132 1.00 0.00 C**

**ATOM 1132 N N.A X -1 115.213 115.710 143.117 1.00 0.00 N**

**ATOM 1133 C N.A X -1 115.632 116.542 144.100 1.00 0.00 C**

**ATOM 1134 N N.A X -1 115.043 116.806 145.212 1.00 0.00 N**

**ATOM 1135 N N.A X -1 116.810 117.105 143.781 1.00 0.00 N**

**ATOM 1136 CA ALA R 57 136.376 111.055 139.465 1.00 0.00 C**

**ATOM 1137 C ALA R 57 135.110 110.437 138.885 1.00 0.00 C**

**ATOM 1138 O ALA R 57 134.871 110.508 137.679 1.00 0.00 O**

**ATOM 1139 N ALA R 57 136.113 111.637 140.776 1.00 0.00 N**

**ATOM 1140 CB ALA R 57 137.482 110.015 139.558 1.00 0.00 C**

**ATOM 1141 CA ALA R 64 130.632 112.472 130.406 1.00 0.00 C**

**ATOM 1142 C ALA R 64 129.873 111.421 129.605 1.00 0.00 C**

**ATOM 1143 O ALA R 64 129.633 111.607 128.406 1.00 0.00 O**

**ATOM 1144 N ALA R 64 130.065 112.621 131.741 1.00 0.00 N**

**ATOM 1145 CB ALA R 64 132.115 112.115 130.501 1.00 0.00 C**

**ATOM 1146 CA ALA R 93 104.351 102.440 106.047 1.00 0.00 C**

**ATOM 1147 C ALA R 93 105.561 102.363 106.969 1.00 0.00 C**

**ATOM 1148 O ALA R 93 105.545 102.957 108.058 1.00 0.00 O**

**ATOM 1149 N ALA R 93 104.732 102.410 104.639 1.00 0.00 N**

**ATOM 1150 CB ALA R 93 103.387 101.293 106.354 1.00 0.00 C**

**ATOM 1151 CA ALA R 102 112.724 109.029 115.490 1.00 0.00 C**

**ATOM 1152 C ALA R 102 112.234 110.281 116.208 1.00 0.00 C**

**ATOM 1153 O ALA R 102 112.981 110.883 116.987 1.00 0.00 O**

**ATOM 1154 N ALA R 102 111.683 108.008 115.423 1.00 0.00 N**

**ATOM 1155 CB ALA R 102 113.212 109.372 114.083 1.00 0.00 C**

**ATOM 1156 CA ALA R 104 109.696 109.988 119.935 1.00 0.00 C**

**ATOM 1157 C ALA R 104 111.050 109.950 120.636 1.00 0.00 C**

**ATOM 1158 O ALA R 104 111.188 110.467 121.749 1.00 0.00 O**

**ATOM 1159 N ALA R 104 109.831 110.345 118.527 1.00 0.00 N**

**ATOM 1160 CB ALA R 104 108.984 108.642 120.069 1.00 0.00 C**

**ATOM 1161 CA ALA R 106 114.248 112.956 119.689 1.00 0.00 C**

**ATOM 1162 C ALA R 106 113.617 113.779 120.799 1.00 0.00 C**

**ATOM 1163 O ALA R 106 114.262 114.680 121.347 1.00 0.00 O**

**ATOM 1164 N ALA R 106 113.719 111.598 119.674 1.00 0.00 N**

**ATOM 1165 CB ALA R 106 114.020 113.630 118.336 1.00 0.00 C**

**ATOM 1166 CA ALA R 159 103.852 90.894 107.158 1.00 0.00 C**

**ATOM 1167 C ALA R 159 104.438 89.535 106.787 1.00 0.00 C**

**ATOM 1168 O ALA R 159 104.411 89.144 105.614 1.00 0.00 O**

**ATOM 1169 N ALA R 159 103.201 90.894 108.470 1.00 0.00 N**

**ATOM 1170 CB ALA R 159 104.941 91.962 107.118 1.00 0.00 C**

**ATOM 1171 CA ALA R 166 99.447 92.083 104.731 1.00 0.00 C**

**ATOM 1172 C ALA R 166 99.049 93.020 103.598 1.00 0.00 C**

**ATOM 1173 O ALA R 166 99.348 94.219 103.653 1.00 0.00 O**

**ATOM 1174 N ALA R 166 98.488 90.993 104.897 1.00 0.00 N**

**ATOM 1175 CB ALA R 166 100.845 91.519 104.486 1.00 0.00 C**

**ATOM 1176 CA ALA R 175 99.467 102.980 109.277 1.00 0.00 C**

**ATOM 1177 C ALA R 175 99.360 104.324 109.990 1.00 0.00 C**

**ATOM 1178 O ALA R 175 99.675 104.427 111.185 1.00 0.00 O**

**ATOM 1179 N ALA R 175 98.301 102.717 108.432 1.00 0.00 N**

**ATOM 1180 CB ALA R 175 100.742 102.922 108.438 1.00 0.00 C**

**ATOM 1181 CA ALA R 193 101.276 114.207 133.791 1.00 0.00 C**

**ATOM 1182 C ALA R 193 101.209 115.729 133.792 1.00 0.00 C**

**ATOM 1183 O ALA R 193 101.929 116.374 134.561 1.00 0.00 O**

**ATOM 1184 N ALA R 193 100.015 113.605 133.374 1.00 0.00 N**

**ATOM 1185 CB ALA R 193 102.413 113.731 132.886 1.00 0.00 C**

**ATOM 1186 CA ALA R 234 105.132 99.383 129.464 1.00 0.00 C**

**ATOM 1187 C ALA R 234 106.214 98.319 129.341 1.00 0.00 C**

**ATOM 1188 O ALA R 234 107.088 98.458 128.479 1.00 0.00 O**

**ATOM 1189 N ALA R 234 104.024 98.994 130.319 1.00 0.00 N**

**ATOM 1190 CB ALA R 234 105.766 100.677 129.980 1.00 0.00 C**

**ATOM 1191 CA ALA R 281 118.851 91.725 123.391 1.00 0.00 C**

**ATOM 1192 C ALA R 281 119.083 93.141 123.904 1.00 0.00 C**

**ATOM 1193 O ALA R 281 119.169 93.351 125.118 1.00 0.00 O**

**ATOM 1194 N ALA R 281 117.848 91.686 122.331 1.00 0.00 N**

**ATOM 1195 CB ALA R 281 120.161 91.117 122.893 1.00 0.00 C**

**ATOM 1196 CA ALA R 298 109.430 100.715 145.686 1.00 0.00 C**

**ATOM 1197 C ALA R 298 108.794 99.830 146.754 1.00 0.00 C**

**ATOM 1198 O ALA R 298 108.052 100.326 147.610 1.00 0.00 O**

**ATOM 1199 N ALA R 298 110.817 100.347 145.419 1.00 0.00 N**

**ATOM 1200 CB ALA R 298 108.618 100.657 144.392 1.00 0.00 C**

**ATOM 1201 CA ALA R 307 127.121 103.573 145.019 1.00 0.00 C**

**ATOM 1202 C ALA R 307 125.769 103.167 144.440 1.00 0.00 C**

**ATOM 1203 O ALA R 307 125.643 102.965 143.225 1.00 0.00 O**

**ATOM 1204 N ALA R 307 126.983 104.554 146.096 1.00 0.00 N**

**ATOM 1205 CB ALA R 307 127.874 102.343 145.526 1.00 0.00 C**

**ATOM 1206 CA ALA R 308 123.400 102.748 144.814 1.00 0.00 C**

**ATOM 1207 C ALA R 308 122.836 103.898 143.990 1.00 0.00 C**

**ATOM 1208 O ALA R 308 122.128 103.661 143.007 1.00 0.00 O**

**ATOM 1209 N ALA R 308 124.746 103.043 145.296 1.00 0.00 N**

**ATOM 1210 CB ALA R 308 122.472 102.428 145.986 1.00 0.00 C**

**ATOM 1211 CA ALA R 317 121.518 106.276 131.456 1.00 0.00 C**

**ATOM 1212 C ALA R 317 121.446 105.155 130.426 1.00 0.00 C**

**ATOM 1213 O ALA R 317 121.431 105.421 129.225 1.00 0.00 O**

**ATOM 1214 N ALA R 317 120.702 105.987 132.632 1.00 0.00 N**

**ATOM 1215 CB ALA R 317 122.966 106.525 131.875 1.00 0.00 C**

**ATOM 1216 CA ALA R 331 117.476 103.157 110.269 1.00 0.00 C**

**ATOM 1217 C ALA R 331 118.735 102.757 109.514 1.00 0.00 C**

**ATOM 1218 O ALA R 331 118.658 102.354 108.352 1.00 0.00 O**

**ATOM 1219 N ALA R 331 117.371 102.488 111.558 1.00 0.00 N**

**ATOM 1220 CB ALA R 331 117.438 104.678 110.459 1.00 0.00 C**

**ATOM 1221 CA GLN R 115 118.196 114.843 134.805 1.00 0.00 C**

**ATOM 1222 C GLN R 115 119.638 114.919 135.292 1.00 0.00 C**

**ATOM 1223 O GLN R 115 119.911 114.650 136.468 1.00 0.00 O**

**ATOM 1224 N GLN R 115 117.893 115.907 133.851 1.00 0.00 N**

**ATOM 1225 CB GLN R 115 117.904 113.479 134.188 1.00 0.00 C**

**ATOM 1226 CG GLN R 115 116.435 113.254 133.904 1.00 0.00 C**

**ATOM 1227 CD GLN R 115 116.169 111.894 133.310 1.00 0.00 C**

**ATOM 1228 OE1 GLN R 115 117.003 110.994 133.397 1.00 0.00 O**

**ATOM 1229 NE2 GLN R 115 114.995 111.727 132.716 1.00 0.00 N**

**ATOM 1230 CA GLN R 213 102.206 115.159 143.473 1.00 0.00 C**

**ATOM 1231 C GLN R 213 100.923 114.336 143.511 1.00 0.00 C**

**ATOM 1232 O GLN R 213 100.067 114.441 142.627 1.00 0.00 O**

**ATOM 1233 N GLN R 213 102.918 115.028 142.203 1.00 0.00 N**

**ATOM 1234 CB GLN R 213 101.868 116.618 143.786 1.00 0.00 C**

**ATOM 1235 CG GLN R 213 103.040 117.530 144.076 1.00 0.00 C**

**ATOM 1236 CD GLN R 213 103.779 117.955 142.850 1.00 0.00 C**

**ATOM 1237 OE1 GLN R 213 103.235 117.929 141.752 1.00 0.00 O**

**ATOM 1238 NE2 GLN R 213 105.043 118.326 143.022 1.00 0.00 N**

**ATOM 1239 CA PRO R 59 130.742 112.288 138.734 1.00 0.00 C**

**ATOM 1240 C PRO R 59 131.155 112.812 137.367 1.00 0.00 C**

**ATOM 1241 O PRO R 59 130.284 112.995 136.507 1.00 0.00 O**

**ATOM 1242 N PRO R 59 131.753 111.415 139.372 1.00 0.00 N**

**ATOM 1243 CD PRO R 59 132.306 112.123 140.553 1.00 0.00 C**

**ATOM 1244 CG PRO R 59 131.821 113.523 140.414 1.00 0.00 C**

**ATOM 1245 CB PRO R 59 130.562 113.433 139.738 1.00 0.00 C**

**ATOM 1246 CA PRO R 113 120.500 117.354 130.616 1.00 0.00 C**

**ATOM 1247 C PRO R 113 119.800 117.831 131.878 1.00 0.00 C**

**ATOM 1248 O PRO R 113 120.479 118.183 132.850 1.00 0.00 O**

**ATOM 1249 N PRO R 113 119.726 116.374 129.820 1.00 0.00 N**

**ATOM 1250 CD PRO R 113 119.418 116.949 128.491 1.00 0.00 C**

**ATOM 1251 CG PRO R 113 119.659 118.408 128.665 1.00 0.00 C**

**ATOM 1252 CB PRO R 113 120.732 118.512 129.636 1.00 0.00 C**

**ATOM 1253 CA PRO R 125 116.276 125.959 140.262 1.00 0.00 C**

**ATOM 1254 C PRO R 125 115.417 126.642 139.207 1.00 0.00 C**

**ATOM 1255 O PRO R 125 115.458 127.870 139.077 1.00 0.00 O**

**ATOM 1256 N PRO R 125 116.398 124.506 140.087 1.00 0.00 N**

**ATOM 1257 CD PRO R 125 117.604 124.139 139.326 1.00 0.00 C**

**ATOM 1258 CG PRO R 125 118.410 125.419 139.298 1.00 0.00 C**

**ATOM 1259 CB PRO R 125 117.730 126.435 140.180 1.00 0.00 C**

**ATOM 1260 CA PRO R 163 100.871 87.547 103.770 1.00 0.00 C**

**ATOM 1261 C PRO R 163 99.623 88.016 103.035 1.00 0.00 C**

**ATOM 1262 O PRO R 163 99.673 89.060 102.374 1.00 0.00 O**

**ATOM 1263 N PRO R 163 100.601 86.739 104.973 1.00 0.00 N**

**ATOM 1264 CD PRO R 163 100.964 85.324 104.776 1.00 0.00 C**

**ATOM 1265 CG PRO R 163 101.268 85.248 103.310 1.00 0.00 C**

**ATOM 1266 CB PRO R 163 101.711 86.602 102.902 1.00 0.00 C**

**ATOM 1267 CA PRO R 172 99.874 104.007 104.451 1.00 0.00 C**

**ATOM 1268 C PRO R 172 98.800 104.720 105.256 1.00 0.00 C**

**ATOM 1269 O PRO R 172 99.095 105.238 106.342 1.00 0.00 O**

**ATOM 1270 N PRO R 172 99.381 102.805 103.752 1.00 0.00 N**

**ATOM 1271 CD PRO R 172 99.699 102.846 102.314 1.00 0.00 C**

**ATOM 1272 CG PRO R 172 99.852 104.309 102.053 1.00 0.00 C**

**ATOM 1273 CB PRO R 172 100.428 104.877 103.311 1.00 0.00 C**

**ATOM 1274 CA PRO R 215 97.700 113.250 148.192 1.00 0.00 C**

**ATOM 1275 C PRO R 215 98.469 112.377 149.175 1.00 0.00 C**

**ATOM 1276 O PRO R 215 98.598 111.164 148.995 1.00 0.00 O**

**ATOM 1277 N PRO R 215 98.035 112.922 146.799 1.00 0.00 N**

**ATOM 1278 CD PRO R 215 96.863 112.400 146.066 1.00 0.00 C**

**ATOM 1279 CG PRO R 215 95.867 112.113 147.148 1.00 0.00 C**

**ATOM 1280 CB PRO R 215 96.188 113.009 148.272 1.00 0.00 C**

**ATOM 1281 CA PRO R 238 105.577 94.776 124.990 1.00 0.00 C**

**ATOM 1282 C PRO R 238 106.639 93.696 124.850 1.00 0.00 C**

**ATOM 1283 O PRO R 238 106.887 93.250 123.724 1.00 0.00 O**

**ATOM 1284 N PRO R 238 104.729 94.662 126.199 1.00 0.00 N**

**ATOM 1285 CD PRO R 238 104.839 95.913 126.986 1.00 0.00 C**

**ATOM 1286 CG PRO R 238 105.330 96.907 126.003 1.00 0.00 C**

**ATOM 1287 CB PRO R 238 106.216 96.164 125.132 1.00 0.00 C**

**ATOM 1288 CA PRO R 289 117.602 96.528 135.209 1.00 0.00 C**

**ATOM 1289 C PRO R 289 116.926 97.685 135.920 1.00 0.00 C**

**ATOM 1290 O PRO R 289 116.721 97.601 137.136 1.00 0.00 O**

**ATOM 1291 N PRO R 289 117.010 96.168 133.901 1.00 0.00 N**

**ATOM 1292 CD PRO R 289 118.036 96.307 132.847 1.00 0.00 C**

**ATOM 1293 CG PRO R 289 119.322 96.254 133.588 1.00 0.00 C**

**ATOM 1294 CB PRO R 289 119.059 96.874 134.865 1.00 0.00 C**

**ATOM 1295 CA PRO R 327 118.451 105.178 115.131 1.00 0.00 C**

**ATOM 1296 C PRO R 327 119.445 104.274 114.418 1.00 0.00 C**

**ATOM 1297 O PRO R 327 119.200 103.904 113.266 1.00 0.00 O**

**ATOM 1298 N PRO R 327 118.190 104.809 116.539 1.00 0.00 N**

**ATOM 1299 CD PRO R 327 118.383 105.989 117.411 1.00 0.00 C**

**ATOM 1300 CG PRO R 327 118.399 107.133 116.457 1.00 0.00 C**

**ATOM 1301 CB PRO R 327 118.980 106.615 115.234 1.00 0.00 C**

**ATOM 1302 CA ASP R 105 113.395 109.348 120.599 1.00 0.00 C**

**ATOM 1303 C ASP R 105 114.052 110.724 120.631 1.00 0.00 C**

**ATOM 1304 O ASP R 105 114.812 111.021 121.562 1.00 0.00 O**

**ATOM 1305 N ASP R 105 112.072 109.390 119.982 1.00 0.00 N**

**ATOM 1306 CB ASP R 105 114.285 108.348 119.861 1.00 0.00 C**

**ATOM 1307 CG ASP R 105 113.785 106.921 119.985 1.00 0.00 C**

**ATOM 1308 OD1 ASP R 105 113.237 106.571 121.052 1.00 0.00 O**

**ATOM 1309 OD2 ASP R 105 113.940 106.149 119.015 1.00 0.00 O**

**ATOM 1310 CA ASP R 128 107.092 124.951 137.602 1.00 0.00 C**

**ATOM 1311 C ASP R 128 106.603 124.540 136.216 1.00 0.00 C**

**ATOM 1312 O ASP R 128 106.336 123.352 135.988 1.00 0.00 O**

**ATOM 1313 N ASP R 128 108.012 126.082 137.543 1.00 0.00 N**

**ATOM 1314 CB ASP R 128 105.904 125.308 138.502 1.00 0.00 C**

**ATOM 1315 CG ASP R 128 105.150 124.087 139.004 1.00 0.00 C**

**ATOM 1316 OD1 ASP R 128 105.560 122.950 138.699 1.00 0.00 O**

**ATOM 1317 OD2 ASP R 128 104.138 124.269 139.712 1.00 0.00 O**

**ATOM 1318 CA ASP R 138 109.324 112.365 129.016 1.00 0.00 C**

**ATOM 1319 C ASP R 138 108.438 111.125 129.114 1.00 0.00 C**

**ATOM 1320 O ASP R 138 108.668 110.138 128.402 1.00 0.00 O**

**ATOM 1321 N ASP R 138 108.549 113.571 128.721 1.00 0.00 N**

**ATOM 1322 CB ASP R 138 110.115 112.567 130.309 1.00 0.00 C**

**ATOM 1323 CG ASP R 138 111.197 111.523 130.503 1.00 0.00 C**

**ATOM 1324 OD1 ASP R 138 111.544 110.827 129.525 1.00 0.00 O**

**ATOM 1325 OD2 ASP R 138 111.705 111.403 131.637 1.00 0.00 O**

**ATOM 1326 CA ASP R 155 103.434 94.870 111.688 1.00 0.00 C**

**ATOM 1327 C ASP R 155 104.309 93.907 110.892 1.00 0.00 C**

**ATOM 1328 O ASP R 155 103.853 93.314 109.909 1.00 0.00 O**

**ATOM 1329 N ASP R 155 103.556 94.628 113.123 1.00 0.00 N**

**ATOM 1330 CB ASP R 155 103.801 96.325 111.378 1.00 0.00 C**

**ATOM 1331 CG ASP R 155 103.449 96.744 109.953 1.00 0.00 C**

**ATOM 1332 OD1 ASP R 155 102.820 95.961 109.209 1.00 0.00 O**

**ATOM 1333 OD2 ASP R 155 103.810 97.877 109.572 1.00 0.00 O**

**ATOM 1334 CA ASP R 168 94.915 94.564 103.555 1.00 0.00 C**

**ATOM 1335 C ASP R 168 95.316 95.217 104.871 1.00 0.00 C**

**ATOM 1336 O ASP R 168 94.444 95.689 105.608 1.00 0.00 O**

**ATOM 1337 N ASP R 168 95.999 93.790 102.969 1.00 0.00 N**

**ATOM 1338 CB ASP R 168 93.690 93.673 103.773 1.00 0.00 C**

**ATOM 1339 CG ASP R 168 93.025 93.269 102.475 1.00 0.00 C**

**ATOM 1340 OD1 ASP R 168 93.269 93.936 101.449 1.00 0.00 O**

**ATOM 1341 OD2 ASP R 168 92.261 92.280 102.479 1.00 0.00 O**

**ATOM 1342 CA ASP R 204 113.679 120.659 156.444 1.00 0.00 C**

**ATOM 1343 C ASP R 204 114.970 120.366 155.686 1.00 0.00 C**

**ATOM 1344 O ASP R 204 116.069 120.594 156.206 1.00 0.00 O**

**ATOM 1345 N ASP R 204 112.644 121.202 155.558 1.00 0.00 N**

**ATOM 1346 CB ASP R 204 113.167 119.427 157.207 1.00 0.00 C**

**ATOM 1347 CG ASP R 204 112.651 118.319 156.304 1.00 0.00 C**

**ATOM 1348 OD1 ASP R 204 112.676 118.468 155.073 1.00 0.00 O**

**ATOM 1349 OD2 ASP R 204 112.213 117.280 156.842 1.00 0.00 O**

**ATOM 1350 CA ASP R 206 115.776 123.234 152.267 1.00 0.00 C**

**ATOM 1351 C ASP R 206 116.130 123.080 150.792 1.00 0.00 C**

**ATOM 1352 O ASP R 206 117.032 123.748 150.280 1.00 0.00 O**

**ATOM 1353 N ASP R 206 115.560 121.926 152.910 1.00 0.00 N**

**ATOM 1354 CB ASP R 206 116.836 124.074 152.994 1.00 0.00 C**

**ATOM 1355 CG ASP R 206 116.263 124.903 154.126 1.00 0.00 C**

**ATOM 1356 OD1 ASP R 206 115.099 125.342 154.013 1.00 0.00 O**

**ATOM 1357 OD2 ASP R 206 116.976 125.115 155.129 1.00 0.00 O**

**ATOM 1358 CA ASP R 216 99.953 112.409 151.124 1.00 0.00 C**

**ATOM 1359 C ASP R 216 99.335 111.433 152.115 1.00 0.00 C**

**ATOM 1360 O ASP R 216 100.086 110.731 152.802 1.00 0.00 O**

**ATOM 1361 N ASP R 216 98.983 113.021 150.225 1.00 0.00 N**

**ATOM 1362 CB ASP R 216 100.710 113.499 151.887 1.00 0.00 C**

**ATOM 1363 CG ASP R 216 101.554 114.369 150.975 1.00 0.00 C**

**ATOM 1364 OD1 ASP R 216 102.010 113.868 149.925 1.00 0.00 O**

**ATOM 1365 OD2 ASP R 216 101.758 115.556 151.305 1.00 0.00 O**

**ATOM 1366 CA ASP R 217 97.327 110.440 153.091 1.00 0.00 C**

**ATOM 1367 C ASP R 217 97.614 109.017 152.622 1.00 0.00 C**

**ATOM 1368 O ASP R 217 97.864 108.123 153.438 1.00 0.00 O**

**ATOM 1369 N ASP R 217 98.004 111.427 152.251 1.00 0.00 N**

**ATOM 1370 CB ASP R 217 95.823 110.710 153.103 1.00 0.00 C**

**ATOM 1371 CG ASP R 217 95.071 109.776 154.028 1.00 0.00 C**

**ATOM 1372 OD1 ASP R 217 95.145 109.972 155.259 1.00 0.00 O**

**ATOM 1373 OD2 ASP R 217 94.407 108.847 153.524 1.00 0.00 O**

**ATOM 1374 CA ASP R 218 97.926 107.516 150.684 1.00 0.00 C**

**ATOM 1375 C ASP R 218 98.914 107.745 149.537 1.00 0.00 C**

**ATOM 1376 O ASP R 218 98.686 107.339 148.399 1.00 0.00 O**

**ATOM 1377 N ASP R 218 97.603 108.800 151.306 1.00 0.00 N**

**ATOM 1378 CB ASP R 218 96.670 106.807 150.180 1.00 0.00 C**

**ATOM 1379 CG ASP R 218 95.797 106.284 151.305 1.00 0.00 C**

**ATOM 1380 OD1 ASP R 218 96.342 105.922 152.368 1.00 0.00 O**

**ATOM 1381 OD2 ASP R 218 94.562 106.239 151.126 1.00 0.00 O**

**ATOM 1382 CA ASP R 223 102.631 105.773 144.061 1.00 0.00 C**

**ATOM 1383 C ASP R 223 103.040 104.361 143.664 1.00 0.00 C**

**ATOM 1384 O ASP R 223 103.466 104.135 142.524 1.00 0.00 O**

**ATOM 1385 N ASP R 223 101.364 105.761 144.784 1.00 0.00 N**

**ATOM 1386 CB ASP R 223 103.712 106.436 144.919 1.00 0.00 C**

**ATOM 1387 CG ASP R 223 105.039 106.598 144.188 1.00 0.00 C**

**ATOM 1388 OD1 ASP R 223 105.059 106.569 142.938 1.00 0.00 O**

**ATOM 1389 OD2 ASP R 223 106.071 106.757 144.872 1.00 0.00 O**

**ATOM 1390 CA ASP R 266 120.642 80.894 104.209 1.00 0.00 C**

**ATOM 1391 C ASP R 266 121.479 81.864 105.044 1.00 0.00 C**

**ATOM 1392 O ASP R 266 121.141 82.155 106.196 1.00 0.00 O**

**ATOM 1393 N ASP R 266 120.581 81.342 102.818 1.00 0.00 N**

**ATOM 1394 CB ASP R 266 121.217 79.471 104.254 1.00 0.00 C**

**ATOM 1395 CG ASP R 266 121.209 78.844 105.652 1.00 0.00 C**

**ATOM 1396 OD1 ASP R 266 120.606 79.394 106.598 1.00 0.00 O**

**ATOM 1397 OD2 ASP R 266 121.823 77.767 105.802 1.00 0.00 O**

**ATOM 1398 CA ASP R 334 117.483 98.527 106.939 1.00 0.00 C**

**ATOM 1399 C ASP R 334 118.105 98.584 105.550 1.00 0.00 C**

**ATOM 1400 O ASP R 334 118.737 99.580 105.184 1.00 0.00 O**

**ATOM 1401 N ASP R 334 118.455 98.941 107.944 1.00 0.00 N**

**ATOM 1402 CB ASP R 334 116.233 99.406 106.987 1.00 0.00 C**

**ATOM 1403 CG ASP R 334 115.363 99.125 108.193 1.00 0.00 C**

**ATOM 1404 OD1 ASP R 334 115.530 98.055 108.815 1.00 0.00 O**

**ATOM 1405 OD2 ASP R 334 114.491 99.964 108.503 1.00 0.00 O**

**ATOM 1406 CA ASN R 77 117.551 111.341 115.582 1.00 0.00 C**

**ATOM 1407 C ASN R 77 117.392 112.512 114.618 1.00 0.00 C**

**ATOM 1408 O ASN R 77 116.472 112.501 113.796 1.00 0.00 O**

**ATOM 1409 N ASN R 77 118.952 110.999 115.819 1.00 0.00 N**

**ATOM 1410 CB ASN R 77 116.859 111.661 116.908 1.00 0.00 C**

**ATOM 1411 CG ASN R 77 116.609 110.427 117.752 1.00 0.00 C**

**ATOM 1412 OD1 ASN R 77 116.159 109.399 117.250 1.00 0.00 O**

**ATOM 1413 ND2 ASN R 77 116.904 110.524 119.043 1.00 0.00 N**

**ATOM 1414 CA ASN R 95 109.220 104.984 106.804 1.00 0.00 C**

**ATOM 1415 C ASN R 95 108.383 105.847 107.745 1.00 0.00 C**

**ATOM 1416 O ASN R 95 108.940 106.655 108.493 1.00 0.00 O**

**ATOM 1417 N ASN R 95 108.636 103.660 106.604 1.00 0.00 N**

**ATOM 1418 CB ASN R 95 109.400 105.687 105.458 1.00 0.00 C**

**ATOM 1419 CG ASN R 95 110.455 105.027 104.593 1.00 0.00 C**

**ATOM 1420 OD1 ASN R 95 111.399 104.421 105.100 1.00 0.00 O**

**ATOM 1421 ND2 ASN R 95 110.300 105.140 103.279 1.00 0.00 N**

**ATOM 1422 CA ASN R 100 107.348 108.470 114.488 1.00 0.00 C**

**ATOM 1423 C ASN R 100 108.273 107.995 115.606 1.00 0.00 C**

**ATOM 1424 O ASN R 100 108.322 108.619 116.675 1.00 0.00 O**

**ATOM 1425 N ASN R 100 107.944 108.284 113.165 1.00 0.00 N**

**ATOM 1426 CB ASN R 100 106.007 107.745 114.562 1.00 0.00 C**

**ATOM 1427 CG ASN R 100 104.966 108.355 113.646 1.00 0.00 C**

**ATOM 1428 OD1 ASN R 100 104.740 109.565 113.666 1.00 0.00 O**

**ATOM 1429 ND2 ASN R 100 104.336 107.523 112.828 1.00 0.00 N**

**ATOM 1430 CA ASN R 122 120.629 118.069 143.732 1.00 0.00 C**

**ATOM 1431 C ASN R 122 119.439 119.014 143.935 1.00 0.00 C**

**ATOM 1432 O ASN R 122 118.565 118.746 144.763 1.00 0.00 O**

**ATOM 1433 N ASN R 122 121.584 118.614 142.756 1.00 0.00 N**

**ATOM 1434 CB ASN R 122 121.318 117.702 145.066 1.00 0.00 C**

**ATOM 1435 CG ASN R 122 120.481 116.792 145.959 1.00 0.00 C**

**ATOM 1436 OD1 ASN R 122 119.551 116.130 145.498 1.00 0.00 O**

**ATOM 1437 ND2 ASN R 122 120.812 116.760 147.244 1.00 0.00 N**

**ATOM 1438 CA ASN R 141 107.048 110.115 124.389 1.00 0.00 C**

**ATOM 1439 C ASN R 141 107.822 108.844 124.723 1.00 0.00 C**

**ATOM 1440 O ASN R 141 108.168 108.107 123.795 1.00 0.00 O**

**ATOM 1441 N ASN R 141 106.269 110.633 125.500 1.00 0.00 N**

**ATOM 1442 CB ASN R 141 107.977 111.214 123.883 1.00 0.00 C**

**ATOM 1443 CG ASN R 141 107.219 112.298 123.144 1.00 0.00 C**

**ATOM 1444 OD1 ASN R 141 106.444 112.012 122.231 1.00 0.00 O**

**ATOM 1445 ND2 ASN R 141 107.376 113.535 123.585 1.00 0.00 N**

**ATOM 1446 CA ASN R 179 99.928 105.892 114.701 1.00 0.00 C**

**ATOM 1447 C ASN R 179 99.222 107.029 115.442 1.00 0.00 C**

**ATOM 1448 O ASN R 179 99.624 107.369 116.563 1.00 0.00 O**

**ATOM 1449 N ASN R 179 98.994 105.017 113.989 1.00 0.00 N**

**ATOM 1450 CB ASN R 179 100.963 106.444 113.724 1.00 0.00 C**

**ATOM 1451 CG ASN R 179 101.953 105.388 113.277 1.00 0.00 C**

**ATOM 1452 OD1 ASN R 179 102.478 104.630 114.092 1.00 0.00 O**

**ATOM 1453 ND2 ASN R 179 102.202 105.322 111.976 1.00 0.00 N**

**ATOM 1454 CA ASN R 268 120.955 86.268 104.509 1.00 0.00 C**

**ATOM 1455 C ASN R 268 119.924 86.066 105.621 1.00 0.00 C**

**ATOM 1456 O ASN R 268 119.655 86.987 106.407 1.00 0.00 O**

**ATOM 1457 N ASN R 268 121.818 85.093 104.353 1.00 0.00 N**

**ATOM 1458 CB ASN R 268 120.258 86.586 103.186 1.00 0.00 C**

**ATOM 1459 CG ASN R 268 121.224 87.046 102.114 1.00 0.00 C**

**ATOM 1460 OD1 ASN R 268 122.308 87.547 102.412 1.00 0.00 O**

**ATOM 1461 ND2 ASN R 268 120.836 86.876 100.855 1.00 0.00 N**

**ATOM 1462 CA ASN R 322 118.581 102.365 124.196 1.00 0.00 C**

**ATOM 1463 C ASN R 322 117.920 103.306 123.188 1.00 0.00 C**

**ATOM 1464 O ASN R 322 117.912 103.034 121.981 1.00 0.00 O**

**ATOM 1465 N ASN R 322 119.399 103.109 125.160 1.00 0.00 N**

**ATOM 1466 CB ASN R 322 117.513 101.542 124.911 1.00 0.00 C**

**ATOM 1467 CG ASN R 322 116.718 100.645 123.960 1.00 0.00 C**

**ATOM 1468 OD1 ASN R 322 117.128 100.384 122.827 1.00 0.00 O**

**ATOM 1469 ND2 ASN R 322 115.580 100.165 124.427 1.00 0.00 N**

**ATOM 1470 CA ASN R 326 117.512 103.038 118.228 1.00 0.00 C**

**ATOM 1471 C ASN R 326 117.686 103.576 116.800 1.00 0.00 C**

**ATOM 1472 O ASN R 326 117.279 102.856 115.870 1.00 0.00 O**

**ATOM 1473 N ASN R 326 118.611 103.306 119.161 1.00 0.00 N**

**ATOM 1474 CB ASN R 326 116.181 103.531 118.823 1.00 0.00 C**

**ATOM 1475 CG ASN R 326 115.634 102.597 119.883 1.00 0.00 C**

**ATOM 1476 OD1 ASN R 326 115.784 101.379 119.790 1.00 0.00 O**

**ATOM 1477 ND2 ASN R 326 114.977 103.162 120.889 1.00 0.00 N**

**ATOM 1478 CA ASN R 336 116.065 99.942 101.903 1.00 0.00 C**

**ATOM 1479 C ASN R 336 116.495 101.360 102.257 1.00 0.00 C**

**ATOM 1480 O ASN R 336 116.617 102.213 101.366 1.00 0.00 O**

**ATOM 1481 N ASN R 336 116.705 98.963 102.773 1.00 0.00 N**

**ATOM 1482 CB ASN R 336 114.539 99.809 102.008 1.00 0.00 C**

**ATOM 1483 CG ASN R 336 113.795 100.503 100.873 1.00 0.00 C**

**ATOM 1484 OD1 ASN R 336 114.396 101.084 99.969 1.00 0.00 O**

**ATOM 1485 ND2 ASN R 336 112.469 100.439 100.920 1.00 0.00 N**

**ATOM 1486 CA LYS R 89 109.072 110.663 100.643 1.00 0.00 C**

**ATOM 1487 C LYS R 89 108.389 109.530 101.403 1.00 0.00 C**

**ATOM 1488 O LYS R 89 108.715 108.352 101.226 1.00 0.00 O**

**ATOM 1489 N LYS R 89 110.485 110.373 100.433 1.00 0.00 N**

**ATOM 1490 CB LYS R 89 108.385 110.902 99.299 1.00 0.00 C**

**ATOM 1491 CG LYS R 89 108.698 109.850 98.247 1.00 0.00 C**

**ATOM 1492 CD LYS R 89 108.106 110.221 96.899 1.00 0.00 C**

**ATOM 1493 CE LYS R 89 108.413 109.162 95.855 1.00 0.00 C**

**ATOM 1494 NZ LYS R 89 109.880 108.999 95.655 1.00 0.00 N**

**ATOM 1495 CA LYS R 91 104.972 106.611 100.674 1.00 0.00 C**

**ATOM 1496 C LYS R 91 104.736 105.210 101.219 1.00 0.00 C**

**ATOM 1497 O LYS R 91 103.664 104.643 100.980 1.00 0.00 O**

**ATOM 1498 N LYS R 91 105.957 107.360 101.442 1.00 0.00 N**

**ATOM 1499 CB LYS R 91 105.391 106.523 99.202 1.00 0.00 C**

**ATOM 1500 CG LYS R 91 105.569 107.880 98.546 1.00 0.00 C**

**ATOM 1501 CD LYS R 91 104.305 108.720 98.669 1.00 0.00 C**

**ATOM 1502 CE LYS R 91 104.489 110.099 98.056 1.00 0.00 C**

**ATOM 1503 NZ LYS R 91 103.272 110.942 98.220 1.00 0.00 N**

**ATOM 1504 CA LYS R 132 105.383 120.068 134.114 1.00 0.00 C**

**ATOM 1505 C LYS R 132 105.579 119.621 132.666 1.00 0.00 C**

**ATOM 1506 O LYS R 132 105.242 118.478 132.317 1.00 0.00 O**

**ATOM 1507 N LYS R 132 106.570 120.731 134.656 1.00 0.00 N**

**ATOM 1508 CB LYS R 132 104.177 121.002 134.225 1.00 0.00 C**

**ATOM 1509 CG LYS R 132 103.795 121.356 135.656 1.00 0.00 C**

**ATOM 1510 CD LYS R 132 102.740 122.453 135.701 1.00 0.00 C**

**ATOM 1511 CE LYS R 132 101.406 121.988 135.155 1.00 0.00 C**

**ATOM 1512 NZ LYS R 132 100.778 120.981 136.053 1.00 0.00 N**

**ATOM 1513 CA LYS R 165 96.271 90.023 105.278 1.00 0.00 C**

**ATOM 1514 C LYS R 165 97.207 91.222 105.215 1.00 0.00 C**

**ATOM 1515 O LYS R 165 96.769 92.347 105.490 1.00 0.00 O**

**ATOM 1516 N LYS R 165 96.771 88.900 104.494 1.00 0.00 N**

**ATOM 1517 CB LYS R 165 96.034 89.619 106.742 1.00 0.00 C**

**ATOM 1518 CG LYS R 165 94.704 88.890 107.064 1.00 0.00 C**

**ATOM 1519 CD LYS R 165 94.527 87.520 106.412 1.00 0.00 C**

**ATOM 1520 CE LYS R 165 93.198 86.889 106.792 1.00 0.00 C**

**ATOM 1521 NZ LYS R 165 93.076 86.696 108.263 1.00 0.00 N**

**ATOM 1522 CA LYS R 174 95.913 102.484 107.946 1.00 0.00 C**

**ATOM 1523 C LYS R 174 97.060 102.662 108.936 1.00 0.00 C**

**ATOM 1524 O LYS R 174 96.831 102.717 110.150 1.00 0.00 O**

**ATOM 1525 N LYS R 174 96.109 103.289 106.742 1.00 0.00 N**

**ATOM 1526 CB LYS R 174 95.755 101.013 107.566 1.00 0.00 C**

**ATOM 1527 CG LYS R 174 95.096 100.160 108.634 1.00 0.00 C**

**ATOM 1528 CD LYS R 174 94.760 98.779 108.097 1.00 0.00 C**

**ATOM 1529 CE LYS R 174 94.253 97.865 109.199 1.00 0.00 C**

**ATOM 1530 NZ LYS R 174 92.955 98.332 109.758 1.00 0.00 N**

**ATOM 1531 CA LYS R 176 98.714 106.673 109.888 1.00 0.00 C**

**ATOM 1532 C LYS R 176 97.624 106.623 110.953 1.00 0.00 C**

**ATOM 1533 O LYS R 176 97.772 107.224 112.021 1.00 0.00 O**

**ATOM 1534 N LYS R 176 98.916 105.363 109.271 1.00 0.00 N**

**ATOM 1535 CB LYS R 176 98.387 107.727 108.829 1.00 0.00 C**

**ATOM 1536 CG LYS R 176 99.535 108.043 107.884 1.00 0.00 C**

**ATOM 1537 CD LYS R 176 99.120 109.042 106.817 1.00 0.00 C**

**ATOM 1538 CE LYS R 176 100.259 109.313 105.849 1.00 0.00 C**

**ATOM 1539 NZ LYS R 176 99.859 110.252 104.767 1.00 0.00 N**

**ATOM 1540 CA LYS R 200 107.179 121.407 145.689 1.00 0.00 C**

**ATOM 1541 C LYS R 200 108.501 121.621 146.422 1.00 0.00 C**

**ATOM 1542 O LYS R 200 109.498 120.951 146.141 1.00 0.00 O**

**ATOM 1543 N LYS R 200 107.402 121.165 144.263 1.00 0.00 N**

**ATOM 1544 CB LYS R 200 106.385 120.280 146.387 1.00 0.00 C**

**ATOM 1545 CG LYS R 200 107.035 118.906 146.412 1.00 0.00 C**

**ATOM 1546 CD LYS R 200 106.402 118.006 147.467 1.00 0.00 C**

**ATOM 1547 CE LYS R 200 104.961 117.679 147.162 1.00 0.00 C**

**ATOM 1548 NZ LYS R 200 104.392 116.712 148.139 1.00 0.00 N**

**ATOM 1549 CA LYS R 227 104.538 102.125 139.451 1.00 0.00 C**

**ATOM 1550 C LYS R 227 104.252 100.715 138.939 1.00 0.00 C**

**ATOM 1551 O LYS R 227 104.854 100.294 137.944 1.00 0.00 O**

**ATOM 1552 N LYS R 227 103.325 102.807 139.901 1.00 0.00 N**

**ATOM 1553 CB LYS R 227 105.577 102.080 140.572 1.00 0.00 C**

**ATOM 1554 CG LYS R 227 106.208 103.427 140.891 1.00 0.00 C**

**ATOM 1555 CD LYS R 227 107.160 103.323 142.071 1.00 0.00 C**

**ATOM 1556 CE LYS R 227 107.861 104.644 142.341 1.00 0.00 C**

**ATOM 1557 NZ LYS R 227 108.773 104.555 143.516 1.00 0.00 N**

**ATOM 1558 CA LYS R 254 112.558 78.448 108.695 1.00 0.00 C**

**ATOM 1559 C LYS R 254 111.816 77.474 107.783 1.00 0.00 C**

**ATOM 1560 O LYS R 254 112.445 76.567 107.228 1.00 0.00 O**

**ATOM 1561 N LYS R 254 111.851 79.719 108.859 1.00 0.00 N**

**ATOM 1562 CB LYS R 254 112.817 77.779 110.048 1.00 0.00 C**

**ATOM 1563 CG LYS R 254 113.783 78.526 110.950 1.00 0.00 C**

**ATOM 1564 CD LYS R 254 115.185 78.553 110.365 1.00 0.00 C**

**ATOM 1565 CE LYS R 254 116.156 79.251 111.303 1.00 0.00 C**

**ATOM 1566 NZ LYS R 254 117.525 79.336 110.727 1.00 0.00 N**

**ATOM 1567 CA LYS R 265 120.129 82.925 101.012 1.00 0.00 C**

**ATOM 1568 C LYS R 265 120.030 82.515 102.478 1.00 0.00 C**

**ATOM 1569 O LYS R 265 119.430 83.229 103.292 1.00 0.00 O**

**ATOM 1570 N LYS R 265 121.412 82.520 100.444 1.00 0.00 N**

**ATOM 1571 CB LYS R 265 118.983 82.336 100.193 1.00 0.00 C**

**ATOM 1572 CG LYS R 265 117.647 83.006 100.445 1.00 0.00 C**

**ATOM 1573 CD LYS R 265 117.630 84.408 99.854 1.00 0.00 C**

**ATOM 1574 CE LYS R 265 117.583 84.367 98.333 1.00 0.00 C**

**ATOM 1575 NZ LYS R 265 117.664 85.730 97.738 1.00 0.00 N**

**ATOM 1576 CA LYS R 338 120.810 102.216 102.748 1.00 0.00 C**

**ATOM 1577 C LYS R 338 120.732 102.651 101.288 1.00 0.00 C**

**ATOM 1578 O LYS R 338 121.465 103.548 100.856 1.00 0.00 O**

**ATOM 1579 N LYS R 338 119.479 102.111 103.338 1.00 0.00 N**

**ATOM 1580 CB LYS R 338 121.543 100.880 102.872 1.00 0.00 C**

**ATOM 1581 CG LYS R 338 121.858 100.471 104.299 1.00 0.00 C**

**ATOM 1582 CD LYS R 338 122.495 99.093 104.345 1.00 0.00 C**

**ATOM 1583 CE LYS R 338 122.772 98.658 105.774 1.00 0.00 C**

**ATOM 1584 NZ LYS R 338 123.346 97.285 105.834 1.00 0.00 N**

**ATOM 1585 CA CYS R 131 108.890 120.894 135.395 1.00 0.00 C**

**ATOM 1586 C CYS R 131 107.745 120.097 134.775 1.00 0.00 C**

**ATOM 1587 O CYS R 131 107.924 118.934 134.381 1.00 0.00 O**

**ATOM 1588 N CYS R 131 109.098 122.173 134.713 1.00 0.00 N**

**ATOM 1589 CB CYS R 131 108.624 121.165 136.879 1.00 0.00 C**

**ATOM 1590 SG CYS R 131 108.253 119.751 137.948 1.00 0.00 S**

**ATOM 1591 CA CYS R 161 103.078 85.119 108.953 1.00 0.00 C**

**ATOM 1592 C CYS R 161 101.730 85.136 108.243 1.00 0.00 C**

**ATOM 1593 O CYS R 161 101.189 84.066 107.941 1.00 0.00 O**

**ATOM 1594 N CYS R 161 103.898 86.296 108.685 1.00 0.00 N**

**ATOM 1595 CB CYS R 161 102.858 84.963 110.460 1.00 0.00 C**

**ATOM 1596 SG CYS R 161 104.378 84.675 111.389 1.00 0.00 S**

**ATOM 1597 CA CYS R 181 95.730 105.924 117.858 1.00 0.00 C**

**ATOM 1598 C CYS R 181 96.784 105.716 118.947 1.00 0.00 C**

**ATOM 1599 O CYS R 181 96.466 105.796 120.138 1.00 0.00 O**

**ATOM 1600 N CYS R 181 96.281 106.628 116.698 1.00 0.00 N**

**ATOM 1601 CB CYS R 181 95.115 104.586 117.445 1.00 0.00 C**

**ATOM 1602 SG CYS R 181 93.645 104.713 116.401 1.00 0.00 S**

**ATOM 1603 CA CYS R 210 110.047 118.385 140.803 1.00 0.00 C**

**ATOM 1604 C CYS R 210 109.536 116.948 140.725 1.00 0.00 C**

**ATOM 1605 O CYS R 210 110.314 116.037 140.422 1.00 0.00 O**

**ATOM 1606 N CYS R 210 111.007 118.480 141.898 1.00 0.00 N**

**ATOM 1607 CB CYS R 210 110.742 118.801 139.503 1.00 0.00 C**

**ATOM 1608 SG CYS R 210 109.901 118.574 137.915 1.00 0.00 S**

**ATOM 1609 CA CYS R 229 100.607 99.970 136.303 1.00 0.00 C**

**ATOM 1610 C CYS R 229 101.573 100.313 135.171 1.00 0.00 C**

**ATOM 1611 O CYS R 229 101.504 99.721 134.083 1.00 0.00 O**

**ATOM 1612 N CYS R 229 101.314 99.773 137.572 1.00 0.00 N**

**ATOM 1613 CB CYS R 229 99.539 101.057 136.450 1.00 0.00 C**

**ATOM 1614 SG CYS R 229 98.157 100.623 137.535 1.00 0.00 S**

**ATOM 1615 CA CYS R 245 106.012 87.867 117.337 1.00 0.00 C**

**ATOM 1616 C CYS R 245 107.295 87.458 116.620 1.00 0.00 C**

**ATOM 1617 O CYS R 245 107.238 86.816 115.566 1.00 0.00 O**

**ATOM 1618 N CYS R 245 106.002 87.448 118.738 1.00 0.00 N**

**ATOM 1619 CB CYS R 245 105.793 89.378 117.234 1.00 0.00 C**

**ATOM 1620 SG CYS R 245 104.090 89.911 117.550 1.00 0.00 S**

**ATOM 1621 CA CYS R 286 118.471 98.177 128.594 1.00 0.00 C**

**ATOM 1622 C CYS R 286 117.271 98.440 129.503 1.00 0.00 C**

**ATOM 1623 O CYS R 286 117.384 99.250 130.429 1.00 0.00 O**

**ATOM 1624 N CYS R 286 118.601 96.786 128.188 1.00 0.00 N**

**ATOM 1625 CB CYS R 286 118.406 99.078 127.367 1.00 0.00 C**

**ATOM 1626 SG CYS R 286 119.950 99.164 126.426 1.00 0.00 S**

**ATOM 1627 CA CYS R 315 120.597 102.267 134.779 1.00 0.00 C**

**ATOM 1628 C CYS R 315 119.642 103.202 134.044 1.00 0.00 C**

**ATOM 1629 O CYS R 315 119.106 102.833 132.989 1.00 0.00 O**

**ATOM 1630 N CYS R 315 121.683 103.004 135.422 1.00 0.00 N**

**ATOM 1631 CB CYS R 315 119.842 101.423 135.807 1.00 0.00 C**

**ATOM 1632 SG CYS R 315 120.810 100.100 136.571 1.00 0.00 S**

**ATOM 1633 CA CYS R 340 117.776 105.570 99.857 1.00 0.00 C**

**ATOM 1634 C CYS R 340 118.901 106.538 100.209 1.00 0.00 C**

**ATOM 1635 O CYS R 340 118.956 107.647 99.666 1.00 0.00 O**

**ATOM 1636 N CYS R 340 118.231 104.184 99.883 1.00 0.00 N**

**ATOM 1637 CB CYS R 340 116.596 105.758 100.810 1.00 0.00 C**

**ATOM 1638 SG CYS R 340 115.080 104.914 100.302 1.00 0.00 S**

**ATOM 1639 CA SER R 67 126.924 113.940 127.470 1.00 0.00 C**

**ATOM 1640 C SER R 67 127.084 113.194 126.148 1.00 0.00 C**

**ATOM 1641 O SER R 67 126.554 113.632 125.118 1.00 0.00 O**

**ATOM 1642 N SER R 67 126.665 113.028 128.586 1.00 0.00 N**

**ATOM 1643 CB SER R 67 128.168 114.779 127.761 1.00 0.00 C**

**ATOM 1644 OG SER R 67 127.960 115.630 128.875 1.00 0.00 O**

**ATOM 1645 CA SER R 78 118.208 114.624 113.744 1.00 0.00 C**

**ATOM 1646 C SER R 78 118.486 114.239 112.297 1.00 0.00 C**

**ATOM 1647 O SER R 78 117.764 114.662 111.385 1.00 0.00 O**

**ATOM 1648 N SER R 78 118.331 113.466 114.622 1.00 0.00 N**

**ATOM 1649 CB SER R 78 119.154 115.733 114.202 1.00 0.00 C**

**ATOM 1650 OG SER R 78 118.862 116.135 115.530 1.00 0.00 O**

**ATOM 1651 CA SER R 116 121.974 115.439 134.765 1.00 0.00 C**

**ATOM 1652 C SER R 116 122.096 116.565 135.791 1.00 0.00 C**

**ATOM 1653 O SER R 116 122.842 116.450 136.774 1.00 0.00 O**

**ATOM 1654 N SER R 116 120.578 115.208 134.381 1.00 0.00 N**

**ATOM 1655 CB SER R 116 122.811 115.747 133.523 1.00 0.00 C**

**ATOM 1656 OG SER R 116 124.170 115.954 133.864 1.00 0.00 O**

**ATOM 1657 CA SER R 123 118.241 121.032 143.238 1.00 0.00 C**

**ATOM 1658 C SER R 123 117.691 121.344 141.852 1.00 0.00 C**

**ATOM 1659 O SER R 123 118.401 121.249 140.847 1.00 0.00 O**

**ATOM 1660 N SER R 123 119.336 120.079 143.141 1.00 0.00 N**

**ATOM 1661 CB SER R 123 118.684 122.331 143.922 1.00 0.00 C**

**ATOM 1662 OG SER R 123 117.621 123.265 143.969 1.00 0.00 O**

**ATOM 1663 CA SER R 136 104.798 115.244 130.372 1.00 0.00 C**

**ATOM 1664 C SER R 136 105.413 114.735 129.073 1.00 0.00 C**

**ATOM 1665 O SER R 136 105.015 113.675 128.575 1.00 0.00 O**

**ATOM 1666 N SER R 136 105.815 115.716 131.309 1.00 0.00 N**

**ATOM 1667 CB SER R 136 103.795 116.361 130.086 1.00 0.00 C**

**ATOM 1668 OG SER R 136 102.814 115.938 129.156 1.00 0.00 O**

**ATOM 1669 CA SER R 145 108.410 105.068 121.700 1.00 0.00 C**

**ATOM 1670 C SER R 145 108.211 103.567 121.878 1.00 0.00 C**

**ATOM 1671 O SER R 145 108.412 102.788 120.935 1.00 0.00 O**

**ATOM 1672 N SER R 145 107.294 105.826 122.262 1.00 0.00 N**

**ATOM 1673 CB SER R 145 109.720 105.520 122.345 1.00 0.00 C**

**ATOM 1674 OG SER R 145 110.810 104.726 121.910 1.00 0.00 O**

**ATOM 1675 CA SER R 153 107.042 94.201 115.720 1.00 0.00 C**

**ATOM 1676 C SER R 153 105.792 93.461 115.245 1.00 0.00 C**

**ATOM 1677 O SER R 153 105.892 92.464 114.517 1.00 0.00 O**

**ATOM 1678 N SER R 153 106.895 95.650 115.586 1.00 0.00 N**

**ATOM 1679 CB SER R 153 107.360 93.841 117.171 1.00 0.00 C**

**ATOM 1680 OG SER R 153 107.520 92.442 117.323 1.00 0.00 O**

**ATOM 1681 CA SER R 186 99.718 108.104 125.096 1.00 0.00 C**

**ATOM 1682 C SER R 186 99.209 109.399 125.725 1.00 0.00 C**

**ATOM 1683 O SER R 186 99.637 109.761 126.830 1.00 0.00 O**

**ATOM 1684 N SER R 186 98.712 107.499 124.227 1.00 0.00 N**

**ATOM 1685 CB SER R 186 101.006 108.362 124.316 1.00 0.00 C**

**ATOM 1686 OG SER R 186 101.560 107.152 123.828 1.00 0.00 O**

**ATOM 1687 CA SER R 187 97.804 111.409 125.472 1.00 0.00 C**

**ATOM 1688 C SER R 187 97.055 111.322 126.800 1.00 0.00 C**

**ATOM 1689 O SER R 187 96.981 112.333 127.500 1.00 0.00 O**

**ATOM 1690 N SER R 187 98.355 110.137 125.001 1.00 0.00 N**

**ATOM 1691 CB SER R 187 96.882 112.009 124.408 1.00 0.00 C**

**ATOM 1692 OG SER R 187 96.336 113.242 124.843 1.00 0.00 O**

**ATOM 1693 CA SER R 188 95.877 110.018 128.484 1.00 0.00 C**

**ATOM 1694 C SER R 188 96.896 110.185 129.607 1.00 0.00 C**

**ATOM 1695 O SER R 188 96.683 110.959 130.553 1.00 0.00 O**

**ATOM 1696 N SER R 188 96.507 110.158 127.170 1.00 0.00 N**

**ATOM 1697 CB SER R 188 95.178 108.662 128.585 1.00 0.00 C**

**ATOM 1698 OG SER R 188 96.117 107.601 128.546 1.00 0.00 O**

**ATOM 1699 CA SER R 192 97.666 112.974 133.642 1.00 0.00 C**

**ATOM 1700 C SER R 192 98.962 113.566 134.194 1.00 0.00 C**

**ATOM 1701 O SER R 192 99.008 114.015 135.345 1.00 0.00 O**

**ATOM 1702 N SER R 192 97.486 113.291 132.224 1.00 0.00 N**

**ATOM 1703 CB SER R 192 97.640 111.459 133.846 1.00 0.00 C**

**ATOM 1704 OG SER R 192 96.374 110.922 133.504 1.00 0.00 O**

**ATOM 1705 CA SER R 211 107.710 115.377 140.990 1.00 0.00 C**

**ATOM 1706 C SER R 211 106.196 115.360 140.800 1.00 0.00 C**

**ATOM 1707 O SER R 211 105.531 116.395 140.794 1.00 0.00 O**

**ATOM 1708 N SER R 211 108.232 116.743 140.963 1.00 0.00 N**

**ATOM 1709 CB SER R 211 108.069 114.668 142.304 1.00 0.00 C**

**ATOM 1710 OG SER R 211 107.408 115.267 143.404 1.00 0.00 O**

**ATOM 1711 CA SER R 220 103.005 105.434 149.156 1.00 0.00 C**

**ATOM 1712 C SER R 220 102.169 104.390 148.427 1.00 0.00 C**

**ATOM 1713 O SER R 220 102.678 103.712 147.525 1.00 0.00 O**

**ATOM 1714 N SER R 220 102.247 106.652 149.434 1.00 0.00 N**

**ATOM 1715 CB SER R 220 103.555 104.855 150.460 1.00 0.00 C**

**ATOM 1716 OG SER R 220 104.297 103.672 150.222 1.00 0.00 O**

**ATOM 1717 CA SER R 255 109.700 76.788 106.748 1.00 0.00 C**

**ATOM 1718 C SER R 255 109.528 77.369 105.346 1.00 0.00 C**

**ATOM 1719 O SER R 255 108.620 76.950 104.619 1.00 0.00 O**

**ATOM 1720 N SER R 255 110.503 77.638 107.615 1.00 0.00 N**

**ATOM 1721 CB SER R 255 108.330 76.536 107.383 1.00 0.00 C**

**ATOM 1722 OG SER R 255 108.459 75.862 108.623 1.00 0.00 O**

**ATOM 1723 CA SER R 260 116.668 77.248 102.595 1.00 0.00 C**

**ATOM 1724 C SER R 260 117.105 76.736 101.229 1.00 0.00 C**

**ATOM 1725 O SER R 260 117.731 75.673 101.153 1.00 0.00 O**

**ATOM 1726 N SER R 260 115.826 78.432 102.496 1.00 0.00 N**

**ATOM 1727 CB SER R 260 115.934 76.137 103.354 1.00 0.00 C**

**ATOM 1728 OG SER R 260 114.745 75.760 102.681 1.00 0.00 O**

**ATOM 1729 CA SER R 262 120.750 78.112 98.747 1.00 0.00 C**

**ATOM 1730 C SER R 262 121.502 78.595 99.978 1.00 0.00 C**

**ATOM 1731 O SER R 262 120.915 79.114 100.931 1.00 0.00 O**

**ATOM 1732 N SER R 262 119.327 78.005 99.034 1.00 0.00 N**

**ATOM 1733 CB SER R 262 121.015 79.056 97.565 1.00 0.00 C**

**ATOM 1734 OG SER R 262 122.404 79.172 97.306 1.00 0.00 O**

**ATOM 1735 CA SER R 301 113.515 97.701 150.691 1.00 0.00 C**

**ATOM 1736 C SER R 301 113.805 99.136 150.276 1.00 0.00 C**

**ATOM 1737 O SER R 301 113.942 99.433 149.090 1.00 0.00 O**

**ATOM 1738 N SER R 301 112.714 97.044 149.664 1.00 0.00 N**

**ATOM 1739 CB SER R 301 114.831 96.954 150.943 1.00 0.00 C**

**ATOM 1740 OG SER R 301 115.633 96.926 149.776 1.00 0.00 O**

**ATOM 1741 CA SER R 303 117.836 102.643 151.238 1.00 0.00 C**

**ATOM 1742 C SER R 303 118.306 103.856 150.444 1.00 0.00 C**

**ATOM 1743 O SER R 303 117.623 104.327 149.530 1.00 0.00 O**

**ATOM 1744 N SER R 303 116.490 102.292 150.806 1.00 0.00 N**

**ATOM 1745 CB SER R 303 118.792 101.458 151.072 1.00 0.00 C**

**ATOM 1746 OG SER R 303 120.103 101.796 151.489 1.00 0.00 O**

**ATOM 1747 CA SER R 305 123.708 104.642 149.257 1.00 0.00 C**

**ATOM 1748 C SER R 305 124.270 105.362 148.037 1.00 0.00 C**

**ATOM 1749 O SER R 305 123.605 105.442 146.998 1.00 0.00 O**

**ATOM 1750 N SER R 305 122.259 104.778 149.300 1.00 0.00 N**

**ATOM 1751 CB SER R 305 124.122 103.168 149.235 1.00 0.00 C**

**ATOM 1752 OG SER R 305 123.673 102.496 150.399 1.00 0.00 O**

**ATOM 1753 CA SER R 310 125.434 105.962 140.912 1.00 0.00 C**

**ATOM 1754 C SER R 310 124.903 104.852 140.014 1.00 0.00 C**

**ATOM 1755 O SER R 310 124.742 105.056 138.806 1.00 0.00 O**

**ATOM 1756 N SER R 310 124.700 106.024 142.171 1.00 0.00 N**

**ATOM 1757 CB SER R 310 126.925 105.764 141.180 1.00 0.00 C**

**ATOM 1758 OG SER R 310 127.642 105.634 139.966 1.00 0.00 O**

**ATOM 1759 CA SER R 311 124.072 102.569 139.805 1.00 0.00 C**

**ATOM 1760 C SER R 311 122.661 102.889 139.318 1.00 0.00 C**

**ATOM 1761 O SER R 311 122.274 102.485 138.215 1.00 0.00 O**

**ATOM 1762 N SER R 311 124.636 103.670 140.584 1.00 0.00 N**

**ATOM 1763 CB SER R 311 124.071 101.283 140.630 1.00 0.00 C**

**ATOM 1764 OG SER R 311 123.255 101.412 141.778 1.00 0.00 O**

**ATOM 1765 CA SER R 323 116.736 105.405 122.794 1.00 0.00 C**

**ATOM 1766 C SER R 323 117.784 106.071 121.910 1.00 0.00 C**

**ATOM 1767 O SER R 323 117.477 106.489 120.788 1.00 0.00 O**

**ATOM 1768 N SER R 323 117.334 104.406 123.678 1.00 0.00 N**

**ATOM 1769 CB SER R 323 115.988 106.455 123.616 1.00 0.00 C**

**ATOM 1770 OG SER R 323 115.010 105.851 124.446 1.00 0.00 O**

**ATOM 1771 CA SER R 324 120.087 106.760 121.588 1.00 0.00 C**

**ATOM 1772 C SER R 324 120.612 105.781 120.541 1.00 0.00 C**

**ATOM 1773 O SER R 324 121.115 106.208 119.496 1.00 0.00 O**

**ATOM 1774 N SER R 324 119.021 106.179 122.398 1.00 0.00 N**

**ATOM 1775 CB SER R 324 121.235 107.218 122.489 1.00 0.00 C**

**ATOM 1776 OG SER R 324 122.312 107.738 121.731 1.00 0.00 O**

**ATOM 1777 CA TRP R 124 115.748 122.176 140.594 1.00 0.00 C**

**ATOM 1778 C TRP R 124 115.523 123.682 140.677 1.00 0.00 C**

**ATOM 1779 O TRP R 124 114.540 124.129 141.288 1.00 0.00 O**

**ATOM 1780 N TRP R 124 116.414 121.726 141.814 1.00 0.00 N**

**ATOM 1781 CB TRP R 124 114.419 121.440 140.393 1.00 0.00 C**

**ATOM 1782 CG TRP R 124 113.661 121.855 139.157 1.00 0.00 C**

**ATOM 1783 CD1 TRP R 124 112.717 122.839 139.067 1.00 0.00 C**

**ATOM 1784 NE1 TRP R 124 112.251 122.933 137.779 1.00 0.00 N**

**ATOM 1785 CE2 TRP R 124 112.893 122.003 137.005 1.00 0.00 C**

**ATOM 1786 CD2 TRP R 124 113.791 121.305 137.839 1.00 0.00 C**

**ATOM 1787 CE3 TRP R 124 114.577 120.292 137.283 1.00 0.00 C**

**ATOM 1788 CZ3 TRP R 124 114.443 120.011 135.934 1.00 0.00 C**

**ATOM 1789 CH2 TRP R 124 113.542 120.722 135.132 1.00 0.00 C**

**ATOM 1790 CZ2 TRP R 124 112.761 121.719 135.647 1.00 0.00 C**

**ATOM 1791 CA TRP R 183 99.648 109.040 120.131 1.00 0.00 C**

**ATOM 1792 C TRP R 183 98.480 109.446 121.035 1.00 0.00 C**

**ATOM 1793 O TRP R 183 98.697 110.012 122.113 1.00 0.00 O**

**ATOM 1794 N TRP R 183 99.424 107.728 119.521 1.00 0.00 N**

**ATOM 1795 CB TRP R 183 99.941 110.075 119.038 1.00 0.00 C**

**ATOM 1796 CG TRP R 183 101.382 109.991 118.498 1.00 0.00 C**

**ATOM 1797 CD1 TRP R 183 101.856 109.100 117.580 1.00 0.00 C**

**ATOM 1798 NE1 TRP R 183 103.181 109.329 117.322 1.00 0.00 N**

**ATOM 1799 CE2 TRP R 183 103.595 110.407 118.056 1.00 0.00 C**

**ATOM 1800 CD2 TRP R 183 102.488 110.858 118.805 1.00 0.00 C**

**ATOM 1801 CE3 TRP R 183 102.653 111.960 119.644 1.00 0.00 C**

**ATOM 1802 CZ3 TRP R 183 103.898 112.567 119.709 1.00 0.00 C**

**ATOM 1803 CH2 TRP R 183 104.975 112.094 118.956 1.00 0.00 C**

**ATOM 1804 CZ2 TRP R 183 104.845 111.018 118.125 1.00 0.00 C**

**ATOM 1805 CA TRP R 221 100.011 103.360 148.049 1.00 0.00 C**

**ATOM 1806 C TRP R 221 99.698 103.728 146.607 1.00 0.00 C**

**ATOM 1807 O TRP R 221 99.825 102.887 145.712 1.00 0.00 O**

**ATOM 1808 N TRP R 221 100.857 104.371 148.670 1.00 0.00 N**

**ATOM 1809 CB TRP R 221 98.719 103.173 148.845 1.00 0.00 C**

**ATOM 1810 CG TRP R 221 97.843 102.105 148.269 1.00 0.00 C**

**ATOM 1811 CD1 TRP R 221 98.099 100.765 148.237 1.00 0.00 C**

**ATOM 1812 NE1 TRP R 221 97.070 100.100 147.618 1.00 0.00 N**

**ATOM 1813 CE2 TRP R 221 96.125 101.012 147.226 1.00 0.00 C**

**ATOM 1814 CD2 TRP R 221 96.581 102.287 147.616 1.00 0.00 C**

**ATOM 1815 CE3 TRP R 221 95.789 103.403 147.330 1.00 0.00 C**

**ATOM 1816 CZ3 TRP R 221 94.586 103.212 146.673 1.00 0.00 C**

**ATOM 1817 CH2 TRP R 221 94.160 101.932 146.298 1.00 0.00 C**

**ATOM 1818 CZ2 TRP R 221 94.913 100.822 146.565 1.00 0.00 C**

**ATOM 1819 CA TRP R 222 98.950 105.381 145.003 1.00 0.00 C**

**ATOM 1820 C TRP R 222 100.211 105.498 144.153 1.00 0.00 C**

**ATOM 1821 O TRP R 222 100.157 105.241 142.943 1.00 0.00 O**

**ATOM 1822 N TRP R 222 99.256 104.966 146.366 1.00 0.00 N**

**ATOM 1823 CB TRP R 222 98.209 106.719 145.021 1.00 0.00 C**

**ATOM 1824 CG TRP R 222 96.746 106.609 145.335 1.00 0.00 C**

**ATOM 1825 CD1 TRP R 222 96.152 106.802 146.549 1.00 0.00 C**

**ATOM 1826 NE1 TRP R 222 94.794 106.626 146.446 1.00 0.00 N**

**ATOM 1827 CE2 TRP R 222 94.484 106.327 145.145 1.00 0.00 C**

**ATOM 1828 CD2 TRP R 222 95.690 106.312 144.415 1.00 0.00 C**

**ATOM 1829 CE3 TRP R 222 95.647 106.028 143.047 1.00 0.00 C**

**ATOM 1830 CZ3 TRP R 222 94.421 105.772 142.460 1.00 0.00 C**

**ATOM 1831 CH2 TRP R 222 93.239 105.793 143.212 1.00 0.00 C**

**ATOM 1832 CZ2 TRP R 222 93.250 106.068 144.552 1.00 0.00 C**

**ATOM 1833 CA TRP R 287 114.970 98.143 130.122 1.00 0.00 C**

**ATOM 1834 C TRP R 287 114.832 97.318 131.397 1.00 0.00 C**

**ATOM 1835 O TRP R 287 114.345 97.852 132.399 1.00 0.00 O**

**ATOM 1836 N TRP R 287 116.118 97.792 129.287 1.00 0.00 N**

**ATOM 1837 CB TRP R 287 113.664 98.041 129.331 1.00 0.00 C**

**ATOM 1838 CG TRP R 287 113.465 99.141 128.343 1.00 0.00 C**

**ATOM 1839 CD1 TRP R 287 113.632 99.074 126.995 1.00 0.00 C**

**ATOM 1840 NE1 TRP R 287 113.347 100.289 126.422 1.00 0.00 N**

**ATOM 1841 CE2 TRP R 287 112.999 101.175 127.406 1.00 0.00 C**

**ATOM 1842 CD2 TRP R 287 113.060 100.486 128.632 1.00 0.00 C**

**ATOM 1843 CE3 TRP R 287 112.742 101.172 129.808 1.00 0.00 C**

**ATOM 1844 CZ3 TRP R 287 112.381 102.505 129.722 1.00 0.00 C**

**ATOM 1845 CH2 TRP R 287 112.330 103.162 128.486 1.00 0.00 C**

**ATOM 1846 CZ2 TRP R 287 112.634 102.516 127.320 1.00 0.00 C**

**ATOM 1847 CA LEU R 74 120.390 113.999 119.194 1.00 0.00 C**

**ATOM 1848 C LEU R 74 120.835 114.087 117.737 1.00 0.00 C**

**ATOM 1849 O LEU R 74 120.010 114.352 116.854 1.00 0.00 O**

**ATOM 1850 N LEU R 74 120.962 112.830 119.853 1.00 0.00 N**

**ATOM 1851 CB LEU R 74 120.773 115.270 119.952 1.00 0.00 C**

**ATOM 1852 CG LEU R 74 119.981 116.527 119.584 1.00 0.00 C**

**ATOM 1853 CD1 LEU R 74 118.517 116.366 119.967 1.00 0.00 C**

**ATOM 1854 CD2 LEU R 74 120.580 117.760 120.242 1.00 0.00 C**

**ATOM 1855 CA LEU R 79 119.863 112.977 110.728 1.00 0.00 C**

**ATOM 1856 C LEU R 79 118.753 112.099 110.159 1.00 0.00 C**

**ATOM 1857 O LEU R 79 118.365 112.263 108.993 1.00 0.00 O**

**ATOM 1858 N LEU R 79 119.541 113.446 112.073 1.00 0.00 N**

**ATOM 1859 CB LEU R 79 121.191 112.220 110.741 1.00 0.00 C**

**ATOM 1860 CG LEU R 79 121.734 111.767 109.383 1.00 0.00 C**

**ATOM 1861 CD1 LEU R 79 121.805 112.934 108.408 1.00 0.00 C**

**ATOM 1862 CD2 LEU R 79 123.099 111.112 109.538 1.00 0.00 C**

**ATOM 1863 CA LEU R 101 110.024 106.464 116.339 1.00 0.00 C**

**ATOM 1864 C LEU R 101 111.160 107.464 116.529 1.00 0.00 C**

**ATOM 1865 O LEU R 101 111.595 107.712 117.661 1.00 0.00 O**

**ATOM 1866 N LEU R 101 109.055 106.939 115.355 1.00 0.00 N**

**ATOM 1867 CB LEU R 101 110.568 105.099 115.919 1.00 0.00 C**

**ATOM 1868 CG LEU R 101 111.587 104.468 116.867 1.00 0.00 C**

**ATOM 1869 CD1 LEU R 101 110.920 104.104 118.182 1.00 0.00 C**

**ATOM 1870 CD2 LEU R 101 112.228 103.247 116.228 1.00 0.00 C**

**ATOM 1871 CA LEU R 103 110.408 111.829 116.670 1.00 0.00 C**

**ATOM 1872 C LEU R 103 110.268 111.558 118.165 1.00 0.00 C**

**ATOM 1873 O LEU R 103 110.580 112.430 118.993 1.00 0.00 O**

**ATOM 1874 N LEU R 103 110.977 110.678 115.968 1.00 0.00 N**

**ATOM 1875 CB LEU R 103 109.052 112.193 116.064 1.00 0.00 C**

**ATOM 1876 CG LEU R 103 108.284 113.336 116.732 1.00 0.00 C**

**ATOM 1877 CD1 LEU R 103 109.066 114.634 116.641 1.00 0.00 C**

**ATOM 1878 CD2 LEU R 103 106.907 113.490 116.108 1.00 0.00 C**

**ATOM 1879 CA LEU R 107 111.733 114.189 122.252 1.00 0.00 C**

**ATOM 1880 C LEU R 107 112.044 113.551 123.602 1.00 0.00 C**

**ATOM 1881 O LEU R 107 111.878 114.209 124.635 1.00 0.00 O**

**ATOM 1882 N LEU R 107 112.364 113.488 121.144 1.00 0.00 N**

**ATOM 1883 CB LEU R 107 110.221 114.255 122.038 1.00 0.00 C**

**ATOM 1884 CG LEU R 107 109.762 115.113 120.858 1.00 0.00 C**

**ATOM 1885 CD1 LEU R 107 108.298 114.861 120.533 1.00 0.00 C**

**ATOM 1886 CD2 LEU R 107 110.002 116.585 121.154 1.00 0.00 C**

**ATOM 1887 CA LEU R 120 124.702 116.907 140.113 1.00 0.00 C**

**ATOM 1888 C LEU R 120 124.405 118.026 141.108 1.00 0.00 C**

**ATOM 1889 O LEU R 120 125.005 118.078 142.188 1.00 0.00 O**

**ATOM 1890 N LEU R 120 123.492 116.239 139.632 1.00 0.00 N**

**ATOM 1891 CB LEU R 120 125.500 117.454 138.928 1.00 0.00 C**

**ATOM 1892 CG LEU R 120 126.069 116.424 137.955 1.00 0.00 C**

**ATOM 1893 CD1 LEU R 120 126.609 117.105 136.710 1.00 0.00 C**

**ATOM 1894 CD2 LEU R 120 127.166 115.625 138.638 1.00 0.00 C**

**ATOM 1895 CA LEU R 130 109.329 123.677 132.804 1.00 0.00 C**

**ATOM 1896 C LEU R 130 109.299 122.272 133.394 1.00 0.00 C**

**ATOM 1897 O LEU R 130 109.444 121.288 132.660 1.00 0.00 O**

**ATOM 1898 N LEU R 130 108.308 124.525 133.409 1.00 0.00 N**

**ATOM 1899 CB LEU R 130 110.708 124.311 132.981 1.00 0.00 C**

**ATOM 1900 CG LEU R 130 111.870 123.607 132.281 1.00 0.00 C**

**ATOM 1901 CD1 LEU R 130 111.681 123.641 130.772 1.00 0.00 C**

**ATOM 1902 CD2 LEU R 130 113.191 124.249 132.669 1.00 0.00 C**

**ATOM 1903 CA LEU R 149 108.428 99.577 118.731 1.00 0.00 C**

**ATOM 1904 C LEU R 149 107.566 98.317 118.713 1.00 0.00 C**

**ATOM 1905 O LEU R 149 107.783 97.408 117.892 1.00 0.00 O**

**ATOM 1906 N LEU R 149 107.616 100.785 118.902 1.00 0.00 N**

**ATOM 1907 CB LEU R 149 109.462 99.478 119.853 1.00 0.00 C**

**ATOM 1908 CG LEU R 149 110.585 100.513 119.917 1.00 0.00 C**

**ATOM 1909 CD1 LEU R 149 111.403 100.335 121.188 1.00 0.00 C**

**ATOM 1910 CD2 LEU R 149 111.462 100.433 118.694 1.00 0.00 C**

**ATOM 1911 CA LEU R 167 97.879 93.327 101.480 1.00 0.00 C**

**ATOM 1912 C LEU R 167 96.777 94.249 101.990 1.00 0.00 C**

**ATOM 1913 O LEU R 167 96.631 95.375 101.501 1.00 0.00 O**

**ATOM 1914 N LEU R 167 98.409 92.487 102.551 1.00 0.00 N**

**ATOM 1915 CB LEU R 167 97.358 92.454 100.338 1.00 0.00 C**

**ATOM 1916 CG LEU R 167 98.383 91.550 99.648 1.00 0.00 C**

**ATOM 1917 CD1 LEU R 167 97.687 90.517 98.776 1.00 0.00 C**

**ATOM 1918 CD2 LEU R 167 99.361 92.375 98.826 1.00 0.00 C**

**ATOM 1919 CA LEU R 173 96.437 105.339 105.463 1.00 0.00 C**

**ATOM 1920 C LEU R 173 96.194 104.624 106.788 1.00 0.00 C**

**ATOM 1921 O LEU R 173 96.044 105.267 107.835 1.00 0.00 O**

**ATOM 1922 N LEU R 173 97.565 104.753 104.741 1.00 0.00 N**

**ATOM 1923 CB LEU R 173 95.182 105.296 104.590 1.00 0.00 C**

**ATOM 1924 CG LEU R 173 93.905 105.859 105.215 1.00 0.00 C**

**ATOM 1925 CD1 LEU R 173 94.020 107.362 105.406 1.00 0.00 C**

**ATOM 1926 CD2 LEU R 173 92.690 105.510 104.368 1.00 0.00 C**

**ATOM 1927 CA LEU R 184 96.072 109.416 121.468 1.00 0.00 C**

**ATOM 1928 C LEU R 184 96.096 108.559 122.732 1.00 0.00 C**

**ATOM 1929 O LEU R 184 95.710 109.030 123.808 1.00 0.00 O**

**ATOM 1930 N LEU R 184 97.237 109.169 120.614 1.00 0.00 N**

**ATOM 1931 CB LEU R 184 94.769 109.193 120.697 1.00 0.00 C**

**ATOM 1932 CG LEU R 184 94.407 110.220 119.621 1.00 0.00 C**

**ATOM 1933 CD1 LEU R 184 93.265 109.715 118.753 1.00 0.00 C**

**ATOM 1934 CD2 LEU R 184 94.053 111.557 120.255 1.00 0.00 C**

**ATOM 1935 CA LEU R 185 96.574 106.400 123.773 1.00 0.00 C**

**ATOM 1936 C LEU R 185 97.635 106.904 124.746 1.00 0.00 C**

**ATOM 1937 O LEU R 185 97.478 106.767 125.965 1.00 0.00 O**

**ATOM 1938 N LEU R 185 96.457 107.274 122.605 1.00 0.00 N**

**ATOM 1939 CB LEU R 185 96.888 104.971 123.324 1.00 0.00 C**

**ATOM 1940 CG LEU R 185 96.969 103.885 124.399 1.00 0.00 C**

**ATOM 1941 CD1 LEU R 185 95.624 103.719 125.090 1.00 0.00 C**

**ATOM 1942 CD2 LEU R 185 97.435 102.568 123.803 1.00 0.00 C**

**ATOM 1943 CA LEU R 196 99.565 115.896 138.422 1.00 0.00 C**

**ATOM 1944 C LEU R 196 101.001 116.380 138.589 1.00 0.00 C**

**ATOM 1945 O LEU R 196 101.462 116.512 139.730 1.00 0.00 O**

**ATOM 1946 N LEU R 196 98.952 116.292 137.158 1.00 0.00 N**

**ATOM 1947 CB LEU R 196 99.520 114.373 138.569 1.00 0.00 C**

**ATOM 1948 CG LEU R 196 98.122 113.771 138.726 1.00 0.00 C**

**ATOM 1949 CD1 LEU R 196 98.156 112.262 138.546 1.00 0.00 C**

**ATOM 1950 CD2 LEU R 196 97.528 114.140 140.078 1.00 0.00 C**

**ATOM 1951 CA LEU R 212 104.239 113.850 140.519 1.00 0.00 C**

**ATOM 1952 C LEU R 212 103.552 113.900 141.882 1.00 0.00 C**

**ATOM 1953 O LEU R 212 103.598 112.933 142.646 1.00 0.00 O**

**ATOM 1954 N LEU R 212 105.670 114.146 140.630 1.00 0.00 N**

**ATOM 1955 CB LEU R 212 104.048 112.486 139.865 1.00 0.00 C**

**ATOM 1956 CG LEU R 212 104.466 112.406 138.395 1.00 0.00 C**

**ATOM 1957 CD1 LEU R 212 104.399 110.975 137.895 1.00 0.00 C**

**ATOM 1958 CD2 LEU R 212 103.600 113.315 137.538 1.00 0.00 C**

**ATOM 1959 CA LEU R 224 103.194 102.003 144.275 1.00 0.00 C**

**ATOM 1960 C LEU R 224 102.251 101.481 143.196 1.00 0.00 C**

**ATOM 1961 O LEU R 224 102.682 100.760 142.290 1.00 0.00 O**

**ATOM 1962 N LEU R 224 102.914 103.400 144.592 1.00 0.00 N**

**ATOM 1963 CB LEU R 224 103.093 101.150 145.539 1.00 0.00 C**

**ATOM 1964 CG LEU R 224 103.348 99.650 145.382 1.00 0.00 C**

**ATOM 1965 CD1 LEU R 224 104.808 99.386 145.049 1.00 0.00 C**

**ATOM 1966 CD2 LEU R 224 102.936 98.904 146.639 1.00 0.00 C**

**ATOM 1967 CA LEU R 240 105.933 89.204 125.634 1.00 0.00 C**

**ATOM 1968 C LEU R 240 105.443 89.266 124.188 1.00 0.00 C**

**ATOM 1969 O LEU R 240 105.563 88.277 123.452 1.00 0.00 O**

**ATOM 1970 N LEU R 240 106.606 90.439 126.044 1.00 0.00 N**

**ATOM 1971 CB LEU R 240 104.772 88.887 126.578 1.00 0.00 C**

**ATOM 1972 CG LEU R 240 105.136 88.474 128.006 1.00 0.00 C**

**ATOM 1973 CD1 LEU R 240 103.886 88.347 128.863 1.00 0.00 C**

**ATOM 1974 CD2 LEU R 240 105.921 87.172 128.004 1.00 0.00 C**

**ATOM 1975 CA LEU R 248 106.826 82.862 115.424 1.00 0.00 C**

**ATOM 1976 C LEU R 248 107.295 83.312 114.045 1.00 0.00 C**

**ATOM 1977 O LEU R 248 107.153 82.560 113.075 1.00 0.00 O**

**ATOM 1978 N LEU R 248 107.659 83.416 116.488 1.00 0.00 N**

**ATOM 1979 CB LEU R 248 105.361 83.239 115.639 1.00 0.00 C**

**ATOM 1980 CG LEU R 248 104.677 82.633 116.868 1.00 0.00 C**

**ATOM 1981 CD1 LEU R 248 103.256 83.156 116.999 1.00 0.00 C**

**ATOM 1982 CD2 LEU R 248 104.697 81.115 116.813 1.00 0.00 C**

**ATOM 1983 CA LEU R 251 109.799 79.710 113.018 1.00 0.00 C**

**ATOM 1984 C LEU R 251 109.098 79.628 111.663 1.00 0.00 C**

**ATOM 1985 O LEU R 251 109.185 78.602 110.977 1.00 0.00 O**

**ATOM 1986 N LEU R 251 110.346 81.045 113.281 1.00 0.00 N**

**ATOM 1987 CB LEU R 251 108.845 79.294 114.138 1.00 0.00 C**

**ATOM 1988 CG LEU R 251 109.478 78.977 115.495 1.00 0.00 C**

**ATOM 1989 CD1 LEU R 251 108.407 78.706 116.542 1.00 0.00 C**

**ATOM 1990 CD2 LEU R 251 110.434 77.801 115.379 1.00 0.00 C**

**ATOM 1991 CA LEU R 253 110.865 81.803 108.003 1.00 0.00 C**

**ATOM 1992 C LEU R 253 111.702 80.542 107.815 1.00 0.00 C**

**ATOM 1993 O LEU R 253 112.239 80.324 106.722 1.00 0.00 O**

**ATOM 1994 N LEU R 253 109.827 81.640 109.023 1.00 0.00 N**

**ATOM 1995 CB LEU R 253 111.775 82.988 108.331 1.00 0.00 C**

**ATOM 1996 CG LEU R 253 111.194 84.403 108.278 1.00 0.00 C**

**ATOM 1997 CD1 LEU R 253 112.170 85.408 108.869 1.00 0.00 C**

**ATOM 1998 CD2 LEU R 253 110.836 84.780 106.851 1.00 0.00 C**

**ATOM 1999 CA LEU R 258 113.353 80.205 99.217 1.00 0.00 C**

**ATOM 2000 C LEU R 258 114.550 80.418 100.135 1.00 0.00 C**

**ATOM 2001 O LEU R 258 115.693 80.477 99.667 1.00 0.00 O**

**ATOM 2002 N LEU R 258 112.390 79.256 99.778 1.00 0.00 N**

**ATOM 2003 CB LEU R 258 112.675 81.547 98.936 1.00 0.00 C**

**ATOM 2004 CG LEU R 258 111.577 81.624 97.881 1.00 0.00 C**

**ATOM 2005 CD1 LEU R 258 110.914 82.987 97.907 1.00 0.00 C**

**ATOM 2006 CD2 LEU R 258 112.220 81.397 96.532 1.00 0.00 C**

**ATOM 2007 CA LEU R 259 115.366 80.830 102.401 1.00 0.00 C**

**ATOM 2008 C LEU R 259 116.336 79.659 102.523 1.00 0.00 C**

**ATOM 2009 O LEU R 259 117.550 79.863 102.640 1.00 0.00 O**

**ATOM 2010 N LEU R 259 114.299 80.557 101.440 1.00 0.00 N**

**ATOM 2011 CB LEU R 259 114.756 81.194 103.750 1.00 0.00 C**

**ATOM 2012 CG LEU R 259 114.049 82.553 103.706 1.00 0.00 C**

**ATOM 2013 CD1 LEU R 259 113.555 82.937 105.073 1.00 0.00 C**

**ATOM 2014 CD2 LEU R 259 114.956 83.646 103.146 1.00 0.00 C**

**ATOM 2015 CA LEU R 269 118.413 84.516 106.769 1.00 0.00 C**

**ATOM 2016 C LEU R 269 119.106 84.577 108.126 1.00 0.00 C**

**ATOM 2017 O LEU R 269 118.526 85.050 109.111 1.00 0.00 O**

**ATOM 2018 N LEU R 269 119.334 84.867 105.688 1.00 0.00 N**

**ATOM 2019 CB LEU R 269 117.821 83.127 106.524 1.00 0.00 C**

**ATOM 2020 CG LEU R 269 116.931 82.521 107.617 1.00 0.00 C**

**ATOM 2021 CD1 LEU R 269 115.781 83.446 107.998 1.00 0.00 C**

**ATOM 2022 CD2 LEU R 269 116.403 81.164 107.177 1.00 0.00 C**

**ATOM 2023 CA LEU R 275 119.503 91.375 113.614 1.00 0.00 C**

**ATOM 2024 C LEU R 275 118.369 91.359 114.636 1.00 0.00 C**

**ATOM 2025 O LEU R 275 118.296 92.235 115.512 1.00 0.00 O**

**ATOM 2026 N LEU R 275 120.085 90.044 113.424 1.00 0.00 N**

**ATOM 2027 CB LEU R 275 119.002 91.913 112.273 1.00 0.00 C**

**ATOM 2028 CG LEU R 275 118.496 93.354 112.262 1.00 0.00 C**

**ATOM 2029 CD1 LEU R 275 119.613 94.307 112.630 1.00 0.00 C**

**ATOM 2030 CD2 LEU R 275 117.909 93.705 110.903 1.00 0.00 C**

**ATOM 2031 CA LEU R 277 118.451 88.778 118.320 1.00 0.00 C**

**ATOM 2032 C LEU R 277 119.114 90.016 118.921 1.00 0.00 C**

**ATOM 2033 O LEU R 277 119.003 90.250 120.129 1.00 0.00 O**

**ATOM 2034 N LEU R 277 117.820 89.055 117.027 1.00 0.00 N**

**ATOM 2035 CB LEU R 277 119.466 87.642 118.189 1.00 0.00 C**

**ATOM 2036 CG LEU R 277 118.921 86.251 117.858 1.00 0.00 C**

**ATOM 2037 CD1 LEU R 277 120.061 85.270 117.629 1.00 0.00 C**

**ATOM 2038 CD2 LEU R 277 117.996 85.758 118.959 1.00 0.00 C**

**ATOM 2039 CA LEU R 295 110.323 97.887 141.777 1.00 0.00 C**

**ATOM 2040 C LEU R 295 110.834 97.441 143.148 1.00 0.00 C**

**ATOM 2041 O LEU R 295 110.083 97.476 144.134 1.00 0.00 O**

**ATOM 2042 N LEU R 295 111.294 98.723 141.062 1.00 0.00 N**

**ATOM 2043 CB LEU R 295 109.949 96.664 140.945 1.00 0.00 C**

**ATOM 2044 CG LEU R 295 109.034 96.891 139.748 1.00 0.00 C**

**ATOM 2045 CD1 LEU R 295 108.796 95.570 139.061 1.00 0.00 C**

**ATOM 2046 CD2 LEU R 295 107.728 97.528 140.179 1.00 0.00 C**

**ATOM 2047 CA LEU R 299 108.390 97.648 147.687 1.00 0.00 C**

**ATOM 2048 C LEU R 299 109.328 96.855 148.586 1.00 0.00 C**

**ATOM 2049 O LEU R 299 109.072 96.769 149.794 1.00 0.00 O**

**ATOM 2050 N LEU R 299 109.066 98.522 146.732 1.00 0.00 N**

**ATOM 2051 CB LEU R 299 107.421 96.693 146.957 1.00 0.00 C**

**ATOM 2052 CG LEU R 299 107.851 95.773 145.811 1.00 0.00 C**

**ATOM 2053 CD1 LEU R 299 108.129 94.358 146.301 1.00 0.00 C**

**ATOM 2054 CD2 LEU R 299 106.804 95.769 144.707 1.00 0.00 C**

**ATOM 2055 CA LEU R 309 122.730 106.298 143.574 1.00 0.00 C**

**ATOM 2056 C LEU R 309 123.404 106.352 142.208 1.00 0.00 C**

**ATOM 2057 O LEU R 309 122.754 106.650 141.191 1.00 0.00 O**

**ATOM 2058 N LEU R 309 123.192 105.140 144.334 1.00 0.00 N**

**ATOM 2059 CB LEU R 309 123.030 107.572 144.366 1.00 0.00 C**

**ATOM 2060 CG LEU R 309 122.528 108.940 143.903 1.00 0.00 C**

**ATOM 2061 CD1 LEU R 309 121.023 109.012 143.977 1.00 0.00 C**

**ATOM 2062 CD2 LEU R 309 123.163 110.047 144.732 1.00 0.00 C**

**ATOM 2063 CA LEU R 318 121.231 102.768 129.957 1.00 0.00 C**

**ATOM 2064 C LEU R 318 119.856 102.783 129.291 1.00 0.00 C**

**ATOM 2065 O LEU R 318 119.729 102.464 128.096 1.00 0.00 O**

**ATOM 2066 N LEU R 318 121.404 103.896 130.877 1.00 0.00 N**

**ATOM 2067 CB LEU R 318 121.444 101.446 130.693 1.00 0.00 C**

**ATOM 2068 CG LEU R 318 122.869 101.096 131.120 1.00 0.00 C**

**ATOM 2069 CD1 LEU R 318 122.858 99.882 132.034 1.00 0.00 C**

**ATOM 2070 CD2 LEU R 318 123.755 100.856 129.908 1.00 0.00 C**

**ATOM 2071 CA LEU R 325 120.948 103.443 119.878 1.00 0.00 C**

**ATOM 2072 C LEU R 325 119.889 103.057 118.856 1.00 0.00 C**

**ATOM 2073 O LEU R 325 120.242 102.460 117.832 1.00 0.00 O**

**ATOM 2074 N LEU R 325 120.505 104.479 120.805 1.00 0.00 N**

**ATOM 2075 CB LEU R 325 121.390 102.183 120.632 1.00 0.00 C**

**ATOM 2076 CG LEU R 325 122.659 102.260 121.484 1.00 0.00 C**

**ATOM 2077 CD1 LEU R 325 122.857 100.970 122.265 1.00 0.00 C**

**ATOM 2078 CD2 LEU R 325 123.869 102.557 120.615 1.00 0.00 C**

**ATOM 2079 CA LEU R 329 119.573 99.744 115.143 1.00 0.00 C**

**ATOM 2080 C LEU R 329 118.446 99.657 114.121 1.00 0.00 C**

**ATOM 2081 O LEU R 329 118.235 98.590 113.532 1.00 0.00 O**

**ATOM 2082 N LEU R 329 120.139 101.086 115.229 1.00 0.00 N**

**ATOM 2083 CB LEU R 329 119.074 99.297 116.516 1.00 0.00 C**

**ATOM 2084 CG LEU R 329 120.153 99.037 117.566 1.00 0.00 C**

**ATOM 2085 CD1 LEU R 329 119.518 98.754 118.914 1.00 0.00 C**

**ATOM 2086 CD2 LEU R 329 121.046 97.883 117.139 1.00 0.00 C**

**ATOM 2087 CA LEU R 333 120.570 98.721 109.186 1.00 0.00 C**

**ATOM 2088 C LEU R 333 119.486 98.164 108.273 1.00 0.00 C**

**ATOM 2089 O LEU R 333 119.591 97.001 107.868 1.00 0.00 O**

**ATOM 2090 N LEU R 333 120.424 100.142 109.463 1.00 0.00 N**

**ATOM 2091 CB LEU R 333 120.590 97.919 110.498 1.00 0.00 C**

**ATOM 2092 CG LEU R 333 121.870 98.061 111.329 1.00 0.00 C**

**ATOM 2093 CD1 LEU R 333 121.753 97.352 112.665 1.00 0.00 C**

**ATOM 2094 CD2 LEU R 333 123.070 97.542 110.551 1.00 0.00 C**

**ATOM 2095 CA ILE R 58 133.042 109.224 139.321 1.00 0.00 C**

**ATOM 2096 C ILE R 58 132.005 110.216 138.783 1.00 0.00 C**

**ATOM 2097 O ILE R 58 131.389 109.899 137.751 1.00 0.00 O**

**ATOM 2098 N ILE R 58 134.315 109.811 139.760 1.00 0.00 N**

**ATOM 2099 CB ILE R 58 132.504 108.262 140.394 1.00 0.00 C**

**ATOM 2100 CG2 ILE R 58 132.498 108.820 141.826 1.00 0.00 C**

**ATOM 2101 CG1 ILE R 58 131.192 107.581 139.973 1.00 0.00 C**

**ATOM 2102 CD1 ILE R 58 130.795 106.430 140.870 1.00 0.00 C**

**ATOM 2103 CA ILE R 61 132.842 110.122 134.062 1.00 0.00 C**

**ATOM 2104 C ILE R 61 131.346 109.878 133.836 1.00 0.00 C**

**ATOM 2105 O ILE R 61 130.929 109.616 132.703 1.00 0.00 O**

**ATOM 2106 N ILE R 61 133.062 111.181 135.047 1.00 0.00 N**

**ATOM 2107 CB ILE R 61 133.623 108.835 134.418 1.00 0.00 C**

**ATOM 2108 CG2 ILE R 61 135.114 109.131 134.512 1.00 0.00 C**

**ATOM 2109 CG1 ILE R 61 133.143 108.158 135.701 1.00 0.00 C**

**ATOM 2110 CD1 ILE R 61 133.741 106.786 135.927 1.00 0.00 C**

**ATOM 2111 CA ILE R 62 129.073 109.771 134.725 1.00 0.00 C**

**ATOM 2112 C ILE R 62 128.463 110.875 133.858 1.00 0.00 C**

**ATOM 2113 O ILE R 62 127.704 110.593 132.917 1.00 0.00 O**

**ATOM 2114 N ILE R 62 130.519 109.964 134.892 1.00 0.00 N**

**ATOM 2115 CB ILE R 62 128.373 109.656 136.094 1.00 0.00 C**

**ATOM 2116 CG2 ILE R 62 126.860 109.747 135.958 1.00 0.00 C**

**ATOM 2117 CG1 ILE R 62 128.764 108.347 136.786 1.00 0.00 C**

**ATOM 2118 CD1 ILE R 62 128.392 107.105 136.001 1.00 0.00 C**

**ATOM 2119 CA ILE R 84 112.658 111.209 106.178 1.00 0.00 C**

**ATOM 2120 C ILE R 84 111.903 112.455 105.696 1.00 0.00 C**

**ATOM 2121 O ILE R 84 111.008 112.357 104.847 1.00 0.00 O**

**ATOM 2122 N ILE R 84 114.071 111.513 106.432 1.00 0.00 N**

**ATOM 2123 CB ILE R 84 111.999 110.562 107.414 1.00 0.00 C**

**ATOM 2124 CG2 ILE R 84 110.579 110.105 107.110 1.00 0.00 C**

**ATOM 2125 CG1 ILE R 84 112.783 109.325 107.855 1.00 0.00 C**

**ATOM 2126 CD1 ILE R 84 112.857 108.228 106.811 1.00 0.00 C**

**ATOM 2127 CA ILE R 85 111.583 114.878 105.804 1.00 0.00 C**

**ATOM 2128 C ILE R 85 111.874 115.194 104.336 1.00 0.00 C**

**ATOM 2129 O ILE R 85 110.959 115.533 103.574 1.00 0.00 O**

**ATOM 2130 N ILE R 85 112.239 113.637 106.230 1.00 0.00 N**

**ATOM 2131 CB ILE R 85 111.973 116.047 106.736 1.00 0.00 C**

**ATOM 2132 CG2 ILE R 85 111.696 117.410 106.099 1.00 0.00 C**

**ATOM 2133 CG1 ILE R 85 111.243 115.936 108.078 1.00 0.00 C**

**ATOM 2134 CD1 ILE R 85 111.607 117.029 109.059 1.00 0.00 C**

**ATOM 2135 CA ILE R 96 106.181 106.383 108.668 1.00 0.00 C**

**ATOM 2136 C ILE R 96 106.460 105.940 110.105 1.00 0.00 C**

**ATOM 2137 O ILE R 96 106.549 106.769 111.025 1.00 0.00 O**

**ATOM 2138 N ILE R 96 107.056 105.680 107.729 1.00 0.00 N**

**ATOM 2139 CB ILE R 96 104.705 106.175 108.277 1.00 0.00 C**

**ATOM 2140 CG2 ILE R 96 103.769 106.752 109.323 1.00 0.00 C**

**ATOM 2141 CG1 ILE R 96 104.418 106.842 106.931 1.00 0.00 C**

**ATOM 2142 CD1 ILE R 96 103.050 106.523 106.369 1.00 0.00 C**

**ATOM 2143 CA ILE R 98 110.659 105.044 111.549 1.00 0.00 C**

**ATOM 2144 C ILE R 98 110.608 106.540 111.865 1.00 0.00 C**

**ATOM 2145 O ILE R 98 111.309 107.020 112.765 1.00 0.00 O**

**ATOM 2146 N ILE R 98 109.322 104.528 111.230 1.00 0.00 N**

**ATOM 2147 CB ILE R 98 111.653 104.718 110.413 1.00 0.00 C**

**ATOM 2148 CG2 ILE R 98 113.049 105.250 110.708 1.00 0.00 C**

**ATOM 2149 CG1 ILE R 98 111.751 103.206 110.206 1.00 0.00 C**

**ATOM 2150 CD1 ILE R 98 112.533 102.805 108.975 1.00 0.00 C**

**ATOM 2151 CA ILE R 133 106.319 120.125 130.410 1.00 0.00 C**

**ATOM 2152 C ILE R 133 107.354 119.010 130.243 1.00 0.00 C**

**ATOM 2153 O ILE R 133 107.138 118.085 129.454 1.00 0.00 O**

**ATOM 2154 N ILE R 133 106.158 120.485 131.820 1.00 0.00 N**

**ATOM 2155 CB ILE R 133 106.602 121.356 129.516 1.00 0.00 C**

**ATOM 2156 CG2 ILE R 133 105.424 122.327 129.566 1.00 0.00 C**

**ATOM 2157 CG1 ILE R 133 107.908 122.083 129.837 1.00 0.00 C**

**ATOM 2158 CD1 ILE R 133 108.239 123.200 128.869 1.00 0.00 C**

**ATOM 2159 CA ILE R 135 107.676 115.429 132.873 1.00 0.00 C**

**ATOM 2160 C ILE R 135 106.692 114.858 131.853 1.00 0.00 C**

**ATOM 2161 O ILE R 135 106.733 113.657 131.531 1.00 0.00 O**

**ATOM 2162 N ILE R 135 108.261 116.687 132.392 1.00 0.00 N**

**ATOM 2163 CB ILE R 135 107.008 115.625 134.248 1.00 0.00 C**

**ATOM 2164 CG2 ILE R 135 106.112 114.447 134.616 1.00 0.00 C**

**ATOM 2165 CG1 ILE R 135 108.049 115.838 135.345 1.00 0.00 C**

**ATOM 2166 CD1 ILE R 135 107.430 116.205 136.673 1.00 0.00 C**

**ATOM 2167 CA ILE R 137 107.114 115.003 127.337 1.00 0.00 C**

**ATOM 2168 C ILE R 137 107.863 113.697 127.573 1.00 0.00 C**

**ATOM 2169 O ILE R 137 107.784 112.778 126.747 1.00 0.00 O**

**ATOM 2170 N ILE R 137 106.424 115.439 128.550 1.00 0.00 N**

**ATOM 2171 CB ILE R 137 108.041 116.120 126.811 1.00 0.00 C**

**ATOM 2172 CG2 ILE R 137 108.970 115.624 125.707 1.00 0.00 C**

**ATOM 2173 CG1 ILE R 137 107.222 117.325 126.337 1.00 0.00 C**

**ATOM 2174 CD1 ILE R 137 108.064 118.523 125.954 1.00 0.00 C**

**ATOM 2175 CA ILE R 146 107.684 101.711 123.364 1.00 0.00 C**

**ATOM 2176 C ILE R 146 106.522 101.115 122.567 1.00 0.00 C**

**ATOM 2177 O ILE R 146 106.646 100.029 121.976 1.00 0.00 O**

**ATOM 2178 N ILE R 146 107.823 103.143 123.089 1.00 0.00 N**

**ATOM 2179 CB ILE R 146 107.527 101.477 124.880 1.00 0.00 C**

**ATOM 2180 CG2 ILE R 146 107.235 100.019 125.187 1.00 0.00 C**

**ATOM 2181 CG1 ILE R 146 108.795 101.906 125.620 1.00 0.00 C**

**ATOM 2182 CD1 ILE R 146 108.651 101.907 127.126 1.00 0.00 C**

**ATOM 2183 CA ILE R 158 101.645 90.143 110.206 1.00 0.00 C**

**ATOM 2184 C ILE R 158 102.181 90.076 108.776 1.00 0.00 C**

**ATOM 2185 O ILE R 158 101.694 89.288 107.952 1.00 0.00 O**

**ATOM 2186 N ILE R 158 102.741 90.312 111.159 1.00 0.00 N**

**ATOM 2187 CB ILE R 158 100.616 91.278 110.396 1.00 0.00 C**

**ATOM 2188 CG2 ILE R 158 99.572 91.305 109.284 1.00 0.00 C**

**ATOM 2189 CG1 ILE R 158 99.911 91.129 111.746 1.00 0.00 C**

**ATOM 2190 CD1 ILE R 158 98.942 92.250 112.058 1.00 0.00 C**

**ATOM 2191 CA ILE R 177 95.406 105.814 111.619 1.00 0.00 C**

**ATOM 2192 C ILE R 177 95.882 105.127 112.899 1.00 0.00 C**

**ATOM 2193 O ILE R 177 95.601 105.591 114.014 1.00 0.00 O**

**ATOM 2194 N ILE R 177 96.506 105.948 110.655 1.00 0.00 N**

**ATOM 2195 CB ILE R 177 94.219 105.066 110.980 1.00 0.00 C**

**ATOM 2196 CG2 ILE R 177 93.173 104.685 112.022 1.00 0.00 C**

**ATOM 2197 CG1 ILE R 177 93.585 105.910 109.871 1.00 0.00 C**

**ATOM 2198 CD1 ILE R 177 92.510 105.185 109.093 1.00 0.00 C**

**ATOM 2199 CA ILE R 178 97.217 103.323 113.894 1.00 0.00 C**

**ATOM 2200 C ILE R 178 98.169 104.222 114.683 1.00 0.00 C**

**ATOM 2201 O ILE R 178 98.139 104.236 115.921 1.00 0.00 O**

**ATOM 2202 N ILE R 178 96.639 104.032 112.749 1.00 0.00 N**

**ATOM 2203 CB ILE R 178 97.902 102.022 113.426 1.00 0.00 C**

**ATOM 2204 CG2 ILE R 178 98.711 101.369 114.541 1.00 0.00 C**

**ATOM 2205 CG1 ILE R 178 96.861 101.028 112.910 1.00 0.00 C**

**ATOM 2206 CD1 ILE R 178 97.460 99.837 112.195 1.00 0.00 C**

**ATOM 2207 CA ILE R 180 97.328 108.557 115.601 1.00 0.00 C**

**ATOM 2208 C ILE R 180 96.674 107.903 116.815 1.00 0.00 C**

**ATOM 2209 O ILE R 180 96.584 108.518 117.885 1.00 0.00 O**

**ATOM 2210 N ILE R 180 98.148 107.587 114.869 1.00 0.00 N**

**ATOM 2211 CB ILE R 180 96.319 109.260 114.659 1.00 0.00 C**

**ATOM 2212 CG2 ILE R 180 94.994 109.618 115.341 1.00 0.00 C**

**ATOM 2213 CG1 ILE R 180 96.958 110.504 114.013 1.00 0.00 C**

**ATOM 2214 CD1 ILE R 180 97.581 110.345 112.666 1.00 0.00 C**

**ATOM 2215 CA ILE R 182 99.118 105.304 119.538 1.00 0.00 C**

**ATOM 2216 C ILE R 182 99.393 106.618 120.267 1.00 0.00 C**

**ATOM 2217 O ILE R 182 99.530 106.649 121.501 1.00 0.00 O**

**ATOM 2218 N ILE R 182 98.039 105.463 118.558 1.00 0.00 N**

**ATOM 2219 CB ILE R 182 100.398 104.802 118.840 1.00 0.00 C**

**ATOM 2220 CG2 ILE R 182 101.591 104.804 119.791 1.00 0.00 C**

**ATOM 2221 CG1 ILE R 182 100.217 103.412 118.248 1.00 0.00 C**

**ATOM 2222 CD1 ILE R 182 101.357 103.048 117.339 1.00 0.00 C**

**ATOM 2223 CA ILE R 191 97.241 114.829 130.326 1.00 0.00 C**

**ATOM 2224 C ILE R 191 97.322 114.562 131.829 1.00 0.00 C**

**ATOM 2225 O ILE R 191 97.281 115.500 132.638 1.00 0.00 O**

**ATOM 2226 N ILE R 191 98.127 113.927 129.591 1.00 0.00 N**

**ATOM 2227 CB ILE R 191 95.801 114.714 129.780 1.00 0.00 C**

**ATOM 2228 CG2 ILE R 191 94.798 115.517 130.603 1.00 0.00 C**

**ATOM 2229 CG1 ILE R 191 95.744 115.192 128.327 1.00 0.00 C**

**ATOM 2230 CD1 ILE R 191 94.424 114.914 127.643 1.00 0.00 C**

**ATOM 2231 CA ILE R 194 100.192 117.768 132.966 1.00 0.00 C**

**ATOM 2232 C ILE R 194 99.398 118.174 134.205 1.00 0.00 C**

**ATOM 2233 O ILE R 194 99.710 119.176 134.860 1.00 0.00 O**

**ATOM 2234 N ILE R 194 100.371 116.318 132.934 1.00 0.00 N**

**ATOM 2235 CB ILE R 194 99.529 118.259 131.660 1.00 0.00 C**

**ATOM 2236 CG2 ILE R 194 99.140 119.732 131.733 1.00 0.00 C**

**ATOM 2237 CG1 ILE R 194 100.461 118.041 130.468 1.00 0.00 C**

**ATOM 2238 CD1 ILE R 194 99.819 118.328 129.127 1.00 0.00 C**

**ATOM 2239 CA ILE R 208 113.016 122.013 145.963 1.00 0.00 C**

**ATOM 2240 C ILE R 208 113.074 120.786 145.057 1.00 0.00 C**

**ATOM 2241 O ILE R 208 114.101 120.532 144.417 1.00 0.00 O**

**ATOM 2242 N ILE R 208 114.262 122.057 146.716 1.00 0.00 N**

**ATOM 2243 CB ILE R 208 112.764 123.303 145.145 1.00 0.00 C**

**ATOM 2244 CG2 ILE R 208 111.375 123.312 144.636 1.00 0.00 C**

**ATOM 2245 CG1 ILE R 208 112.878 124.617 145.953 1.00 0.00 C**

**ATOM 2246 CD1 ILE R 208 114.279 125.233 146.173 1.00 0.00 C**

**ATOM 2247 CA ILE R 228 102.939 98.678 139.051 1.00 0.00 C**

**ATOM 2248 C ILE R 228 102.204 98.780 137.718 1.00 0.00 C**

**ATOM 2249 O ILE R 228 102.476 98.002 136.796 1.00 0.00 O**

**ATOM 2250 N ILE R 228 103.294 100.008 139.546 1.00 0.00 N**

**ATOM 2251 CB ILE R 228 102.121 97.892 140.099 1.00 0.00 C**

**ATOM 2252 CG2 ILE R 228 101.700 96.524 139.575 1.00 0.00 C**

**ATOM 2253 CG1 ILE R 228 102.913 97.708 141.394 1.00 0.00 C**

**ATOM 2254 CD1 ILE R 228 104.192 96.930 141.223 1.00 0.00 C**

**ATOM 2255 CA ILE R 232 103.186 96.076 133.677 1.00 0.00 C**

**ATOM 2256 C ILE R 232 102.864 96.481 132.244 1.00 0.00 C**

**ATOM 2257 O ILE R 232 103.304 95.809 131.302 1.00 0.00 O**

**ATOM 2258 N ILE R 232 103.884 97.153 134.381 1.00 0.00 N**

**ATOM 2259 CB ILE R 232 101.913 95.652 134.437 1.00 0.00 C**

**ATOM 2260 CG2 ILE R 232 101.055 94.734 133.577 1.00 0.00 C**

**ATOM 2261 CG1 ILE R 232 102.255 94.980 135.771 1.00 0.00 C**

**ATOM 2262 CD1 ILE R 232 103.038 93.706 135.645 1.00 0.00 C**

**ATOM 2263 CA ILE R 237 103.011 93.350 127.539 1.00 0.00 C**

**ATOM 2264 C ILE R 237 103.914 93.576 126.317 1.00 0.00 C**

**ATOM 2265 O ILE R 237 103.905 92.699 125.434 1.00 0.00 O**

**ATOM 2266 N ILE R 237 103.733 93.518 128.801 1.00 0.00 N**

**ATOM 2267 CB ILE R 237 101.745 94.231 127.510 1.00 0.00 C**

**ATOM 2268 CG2 ILE R 237 100.986 94.058 126.208 1.00 0.00 C**

**ATOM 2269 CG1 ILE R 237 100.834 93.895 128.692 1.00 0.00 C**

**ATOM 2270 CD1 ILE R 237 100.385 92.450 128.729 1.00 0.00 C**

**ATOM 2271 CA ILE R 241 104.493 90.560 122.360 1.00 0.00 C**

**ATOM 2272 C ILE R 241 105.687 90.471 121.409 1.00 0.00 C**

**ATOM 2273 O ILE R 241 105.620 89.776 120.389 1.00 0.00 O**

**ATOM 2274 N ILE R 241 104.918 90.422 123.755 1.00 0.00 N**

**ATOM 2275 CB ILE R 241 103.674 91.852 122.159 1.00 0.00 C**

**ATOM 2276 CG2 ILE R 241 103.506 92.192 120.687 1.00 0.00 C**

**ATOM 2277 CG1 ILE R 241 102.300 91.720 122.824 1.00 0.00 C**

**ATOM 2278 CD1 ILE R 241 101.425 92.947 122.672 1.00 0.00 C**

**ATOM 2279 CA ILE R 242 107.983 91.079 120.872 1.00 0.00 C**

**ATOM 2280 C ILE R 242 108.606 89.681 120.821 1.00 0.00 C**

**ATOM 2281 O ILE R 242 108.993 89.213 119.737 1.00 0.00 O**

**ATOM 2282 N ILE R 242 106.809 91.117 121.748 1.00 0.00 N**

**ATOM 2283 CB ILE R 242 108.986 92.164 121.326 1.00 0.00 C**

**ATOM 2284 CG2 ILE R 242 110.402 91.911 120.835 1.00 0.00 C**

**ATOM 2285 CG1 ILE R 242 108.488 93.558 120.927 1.00 0.00 C**

**ATOM 2286 CD1 ILE R 242 109.382 94.684 121.398 1.00 0.00 C**

**ATOM 2287 CA ILE R 243 109.104 87.576 121.955 1.00 0.00 C**

**ATOM 2288 C ILE R 243 108.224 86.650 121.124 1.00 0.00 C**

**ATOM 2289 O ILE R 243 108.733 85.902 120.282 1.00 0.00 O**

**ATOM 2290 N ILE R 243 108.582 88.945 121.939 1.00 0.00 N**

**ATOM 2291 CB ILE R 243 109.272 87.055 123.397 1.00 0.00 C**

**ATOM 2292 CG2 ILE R 243 109.684 85.588 123.407 1.00 0.00 C**

**ATOM 2293 CG1 ILE R 243 110.296 87.886 124.169 1.00 0.00 C**

**ATOM 2294 CD1 ILE R 243 111.670 87.877 123.554 1.00 0.00 C**

**ATOM 2295 CA ILE R 250 111.691 83.034 112.760 1.00 0.00 C**

**ATOM 2296 C ILE R 250 111.258 81.598 112.465 1.00 0.00 C**

**ATOM 2297 O ILE R 250 111.748 80.980 111.509 1.00 0.00 O**

**ATOM 2298 N ILE R 250 110.537 83.864 113.107 1.00 0.00 N**

**ATOM 2299 CB ILE R 250 112.760 83.107 113.871 1.00 0.00 C**

**ATOM 2300 CG2 ILE R 250 113.931 82.176 113.587 1.00 0.00 C**

**ATOM 2301 CG1 ILE R 250 113.278 84.539 114.022 1.00 0.00 C**

**ATOM 2302 CD1 ILE R 250 113.930 85.091 112.772 1.00 0.00 C**

**ATOM 2303 CA ILE R 272 118.114 88.622 109.634 1.00 0.00 C**

**ATOM 2304 C ILE R 272 117.871 88.135 111.062 1.00 0.00 C**

**ATOM 2305 O ILE R 272 117.407 88.892 111.931 1.00 0.00 O**

**ATOM 2306 N ILE R 272 119.403 88.129 109.135 1.00 0.00 N**

**ATOM 2307 CB ILE R 272 116.984 88.190 108.677 1.00 0.00 C**

**ATOM 2308 CG2 ILE R 272 115.605 88.451 109.275 1.00 0.00 C**

**ATOM 2309 CG1 ILE R 272 117.123 88.899 107.329 1.00 0.00 C**

**ATOM 2310 CD1 ILE R 272 116.054 88.520 106.328 1.00 0.00 C**

**ATOM 2311 CA ILE R 290 116.026 99.931 135.882 1.00 0.00 C**

**ATOM 2312 C ILE R 290 114.604 99.648 136.361 1.00 0.00 C**

**ATOM 2313 O ILE R 290 114.209 100.094 137.443 1.00 0.00 O**

**ATOM 2314 N ILE R 290 116.586 98.763 135.209 1.00 0.00 N**

**ATOM 2315 CB ILE R 290 116.124 101.182 134.980 1.00 0.00 C**

**ATOM 2316 CG2 ILE R 290 115.388 101.011 133.643 1.00 0.00 C**

**ATOM 2317 CG1 ILE R 290 115.696 102.443 135.745 1.00 0.00 C**

**ATOM 2318 CD1 ILE R 290 116.589 102.795 136.911 1.00 0.00 C**

**ATOM 2319 CA ILE R 292 113.606 95.766 138.521 1.00 0.00 C**

**ATOM 2320 C ILE R 292 114.107 96.586 139.712 1.00 0.00 C**

**ATOM 2321 O ILE R 292 113.612 96.428 140.834 1.00 0.00 O**

**ATOM 2322 N ILE R 292 113.477 96.601 137.323 1.00 0.00 N**

**ATOM 2323 CB ILE R 292 114.501 94.538 138.246 1.00 0.00 C**

**ATOM 2324 CG2 ILE R 292 114.866 93.781 139.522 1.00 0.00 C**

**ATOM 2325 CG1 ILE R 292 113.817 93.579 137.275 1.00 0.00 C**

**ATOM 2326 CD1 ILE R 292 114.717 92.461 136.825 1.00 0.00 C**

**ATOM 2327 CA ILE R 294 112.667 100.750 140.787 1.00 0.00 C**

**ATOM 2328 C ILE R 294 111.641 99.923 141.557 1.00 0.00 C**

**ATOM 2329 O ILE R 294 111.161 100.358 142.609 1.00 0.00 O**

**ATOM 2330 N ILE R 294 113.773 99.914 140.317 1.00 0.00 N**

**ATOM 2331 CB ILE R 294 112.038 101.529 139.611 1.00 0.00 C**

**ATOM 2332 CG2 ILE R 294 110.729 102.208 140.005 1.00 0.00 C**

**ATOM 2333 CG1 ILE R 294 113.020 102.583 139.093 1.00 0.00 C**

**ATOM 2334 CD1 ILE R 294 112.546 103.309 137.855 1.00 0.00 C**

**ATOM 2335 CA ILE R 316 118.658 105.422 133.840 1.00 0.00 C**

**ATOM 2336 C ILE R 316 119.377 105.811 132.550 1.00 0.00 C**

**ATOM 2337 O ILE R 316 118.754 105.946 131.484 1.00 0.00 O**

**ATOM 2338 N ILE R 316 119.433 104.416 134.571 1.00 0.00 N**

**ATOM 2339 CB ILE R 316 118.384 106.647 134.737 1.00 0.00 C**

**ATOM 2340 CG2 ILE R 316 117.706 107.766 133.963 1.00 0.00 C**

**ATOM 2341 CG1 ILE R 316 117.521 106.257 135.936 1.00 0.00 C**

**ATOM 2342 CD1 ILE R 316 117.219 107.407 136.863 1.00 0.00 C**

**ATOM 2343 CA ILE R 328 121.489 102.967 114.457 1.00 0.00 C**

**ATOM 2344 C ILE R 328 120.928 101.561 114.264 1.00 0.00 C**

**ATOM 2345 O ILE R 328 121.165 100.917 113.235 1.00 0.00 O**

**ATOM 2346 N ILE R 328 120.498 103.824 115.100 1.00 0.00 N**

**ATOM 2347 CB ILE R 328 122.789 102.966 115.286 1.00 0.00 C**

**ATOM 2348 CG2 ILE R 328 123.851 102.067 114.658 1.00 0.00 C**

**ATOM 2349 CG1 ILE R 328 123.334 104.390 115.416 1.00 0.00 C**

**ATOM 2350 CD1 ILE R 328 124.605 104.492 116.237 1.00 0.00 C**

**ATOM 2351 CA HIS R 162 99.874 86.449 107.302 1.00 0.00 C**

**ATOM 2352 C HIS R 162 100.016 87.298 106.042 1.00 0.00 C**

**ATOM 2353 O HIS R 162 99.629 88.475 106.031 1.00 0.00 O**

**ATOM 2354 N HIS R 162 101.170 86.315 107.968 1.00 0.00 N**

**ATOM 2355 CB HIS R 162 98.833 87.044 108.247 1.00 0.00 C**

**ATOM 2356 CG HIS R 162 98.620 86.238 109.489 1.00 0.00 C**

**ATOM 2357 ND1 HIS R 162 97.752 85.169 109.542 1.00 0.00 N**

**ATOM 2358 CE1 HIS R 162 97.770 84.649 110.756 1.00 0.00 C**

**ATOM 2359 NE2 HIS R 162 98.620 85.341 111.493 1.00 0.00 N**

**ATOM 2360 CD2 HIS R 162 99.166 86.340 110.724 1.00 0.00 C**

**ATOM 2361 CA HIS R 291 112.502 98.458 136.044 1.00 0.00 C**

**ATOM 2362 C HIS R 291 112.562 97.584 137.294 1.00 0.00 C**

**ATOM 2363 O HIS R 291 111.809 97.815 138.252 1.00 0.00 O**

**ATOM 2364 N HIS R 291 113.836 98.859 135.601 1.00 0.00 N**

**ATOM 2365 CB HIS R 291 111.752 97.763 134.907 1.00 0.00 C**

**ATOM 2366 CG HIS R 291 111.143 98.716 133.925 1.00 0.00 C**

**ATOM 2367 ND1 HIS R 291 109.929 99.332 134.146 1.00 0.00 N**

**ATOM 2368 CE1 HIS R 291 109.641 100.116 133.123 1.00 0.00 C**

**ATOM 2369 NE2 HIS R 291 110.627 100.036 132.248 1.00 0.00 N**

**ATOM 2370 CD2 HIS R 291 111.580 99.170 132.727 1.00 0.00 C**

**ATOM 2371 CA HIS R 304 120.091 105.501 150.120 1.00 0.00 C**

**ATOM 2372 C HIS R 304 121.619 105.491 150.222 1.00 0.00 C**

**ATOM 2373 O HIS R 304 122.219 106.110 151.108 1.00 0.00 O**

**ATOM 2374 N HIS R 304 119.497 104.350 150.792 1.00 0.00 N**

**ATOM 2375 CB HIS R 304 119.457 106.801 150.655 1.00 0.00 C**

**ATOM 2376 CG HIS R 304 119.561 106.983 152.140 1.00 0.00 C**

**ATOM 2377 ND1 HIS R 304 120.583 107.691 152.735 1.00 0.00 N**

**ATOM 2378 CE1 HIS R 304 120.415 107.687 154.045 1.00 0.00 C**

**ATOM 2379 NE2 HIS R 304 119.320 107.001 154.322 1.00 0.00 N**

**ATOM 2380 CD2 HIS R 304 118.767 106.551 153.148 1.00 0.00 C**

**END**

*********************************************************************************************************

**3. PDB Text: Figure 3 KOR (8vve) with [P3,R8]-Dynorphin A1-11-OH**

**CRYST1 0.000 0.000 0.000 90.00 90.00 90.00 P 1 1**

**ATOM 1 CA TYR A 66 182.466 166.215 131.137 1.00 0.00 C**

**ATOM 2 C TYR A 66 183.377 166.612 132.290 1.00 0.00 C**

**ATOM 3 O TYR A 66 183.224 166.100 133.403 1.00 0.00 O**

**ATOM 4 N TYR A 66 182.269 167.320 130.203 1.00 0.00 N**

**ATOM 5 CB TYR A 66 183.032 165.002 130.391 1.00 0.00 C**

**ATOM 6 CG TYR A 66 183.861 164.055 131.237 1.00 0.00 C**

**ATOM 7 CD1 TYR A 66 183.257 163.185 132.137 1.00 0.00 C**

**ATOM 8 CE1 TYR A 66 184.009 162.321 132.911 1.00 0.00 C**

**ATOM 9 CZ TYR A 66 185.383 162.316 132.787 1.00 0.00 C**

**ATOM 10 OH TYR A 66 186.135 161.456 133.554 1.00 0.00 O**

**ATOM 11 CE2 TYR A 66 186.008 163.167 131.899 1.00 0.00 C**

**ATOM 12 CD2 TYR A 66 185.248 164.028 131.129 1.00 0.00 C**

**ATOM 13 CA TYR A 87 175.965 172.066 160.208 1.00 0.00 C**

**ATOM 14 C TYR A 87 174.541 172.173 160.734 1.00 0.00 C**

**ATOM 15 O TYR A 87 174.214 173.103 161.478 1.00 0.00 O**

**ATOM 16 N TYR A 87 176.338 170.675 159.989 1.00 0.00 N**

**ATOM 17 CB TYR A 87 176.115 172.868 158.914 1.00 0.00 C**

**ATOM 18 CA TYR A 97 163.414 166.886 151.344 1.00 0.00 C**

**ATOM 19 C TYR A 97 164.678 167.442 150.705 1.00 0.00 C**

**ATOM 20 O TYR A 97 165.066 167.016 149.610 1.00 0.00 O**

**ATOM 21 N TYR A 97 163.580 166.740 152.785 1.00 0.00 N**

**ATOM 22 CB TYR A 97 162.215 167.781 151.043 1.00 0.00 C**

**ATOM 23 CG TYR A 97 160.892 167.179 151.448 1.00 0.00 C**

**ATOM 24 CD1 TYR A 97 160.691 165.806 151.410 1.00 0.00 C**

**ATOM 25 CE1 TYR A 97 159.482 165.252 151.783 1.00 0.00 C**

**ATOM 26 CZ TYR A 97 158.456 166.073 152.201 1.00 0.00 C**

**ATOM 27 OH TYR A 97 157.250 165.529 152.573 1.00 0.00 O**

**ATOM 28 CE2 TYR A 97 158.631 167.438 152.249 1.00 0.00 C**

**ATOM 29 CD2 TYR A 97 159.843 167.983 151.874 1.00 0.00 C**

**ATOM 30 CA TYR A 119 178.442 158.912 124.463 1.00 0.00 C**

**ATOM 31 C TYR A 119 179.690 158.980 123.595 1.00 0.00 C**

**ATOM 32 O TYR A 119 179.588 158.948 122.364 1.00 0.00 O**

**ATOM 33 N TYR A 119 178.578 157.918 125.519 1.00 0.00 N**

**ATOM 34 CB TYR A 119 178.148 160.283 125.071 1.00 0.00 C**

**ATOM 35 CA TYR A 139 164.761 156.006 135.926 1.00 0.00 C**

**ATOM 36 C TYR A 139 164.327 156.352 137.346 1.00 0.00 C**

**ATOM 37 O TYR A 139 163.597 157.331 137.546 1.00 0.00 O**

**ATOM 38 N TYR A 139 166.216 155.920 135.795 1.00 0.00 N**

**ATOM 39 CB TYR A 139 164.101 154.690 135.513 1.00 0.00 C**

**ATOM 40 CG TYR A 139 164.033 154.432 134.029 1.00 0.00 C**

**ATOM 41 CD1 TYR A 139 164.236 155.450 133.113 1.00 0.00 C**

**ATOM 42 CE1 TYR A 139 164.161 155.208 131.756 1.00 0.00 C**

**ATOM 43 CZ TYR A 139 163.880 153.939 131.299 1.00 0.00 C**

**ATOM 44 OH TYR A 139 163.808 153.698 129.946 1.00 0.00 O**

**ATOM 45 CE2 TYR A 139 163.670 152.912 132.189 1.00 0.00 C**

**ATOM 46 CD2 TYR A 139 163.745 153.164 133.544 1.00 0.00 C**

**ATOM 47 CA TYR A 140 164.464 155.725 139.743 1.00 0.00 C**

**ATOM 48 C TYR A 140 164.784 157.120 140.255 1.00 0.00 C**

**ATOM 49 O TYR A 140 163.963 157.750 140.928 1.00 0.00 O**

**ATOM 50 N TYR A 140 164.786 155.573 138.330 1.00 0.00 N**

**ATOM 51 CB TYR A 140 165.245 154.670 140.528 1.00 0.00 C**

**ATOM 52 CG TYR A 140 165.065 154.676 142.023 1.00 0.00 C**

**ATOM 53 CD1 TYR A 140 163.901 154.202 142.604 1.00 0.00 C**

**ATOM 54 CE1 TYR A 140 163.747 154.190 143.971 1.00 0.00 C**

**ATOM 55 CZ TYR A 140 164.766 154.631 144.778 1.00 0.00 C**

**ATOM 56 OH TYR A 140 164.595 154.602 146.139 1.00 0.00 O**

**ATOM 57 CE2 TYR A 140 165.943 155.093 144.229 1.00 0.00 C**

**ATOM 58 CD2 TYR A 140 166.088 155.107 142.857 1.00 0.00 C**

**ATOM 59 CA TYR A 157 150.755 174.964 149.495 1.00 0.00 C**

**ATOM 60 C TYR A 157 149.948 174.689 150.756 1.00 0.00 C**

**ATOM 61 O TYR A 157 149.169 175.539 151.199 1.00 0.00 O**

**ATOM 62 N TYR A 157 152.147 174.553 149.652 1.00 0.00 N**

**ATOM 63 CB TYR A 157 150.120 174.258 148.298 1.00 0.00 C**

**ATOM 64 CG TYR A 157 148.606 174.205 148.353 1.00 0.00 C**

**ATOM 65 CD1 TYR A 157 147.843 175.316 148.026 1.00 0.00 C**

**ATOM 66 CE1 TYR A 157 146.462 175.272 148.071 1.00 0.00 C**

**ATOM 67 CZ TYR A 157 145.828 174.108 148.444 1.00 0.00 C**

**ATOM 68 OH TYR A 157 144.455 174.063 148.489 1.00 0.00 O**

**ATOM 69 CE2 TYR A 157 146.560 172.989 148.769 1.00 0.00 C**

**ATOM 70 CD2 TYR A 157 147.939 173.038 148.717 1.00 0.00 C**

**ATOM 71 CA TYR A 219 155.376 146.104 119.474 1.00 0.00 C**

**ATOM 72 C TYR A 219 155.668 147.541 119.880 1.00 0.00 C**

**ATOM 73 O TYR A 219 156.264 147.795 120.932 1.00 0.00 O**

**ATOM 74 N TYR A 219 155.444 145.186 120.607 1.00 0.00 N**

**ATOM 75 CB TYR A 219 154.007 146.018 118.793 1.00 0.00 C**

**ATOM 76 CA TYR A 246 152.298 177.696 143.780 1.00 0.00 C**

**ATOM 77 C TYR A 246 151.503 178.953 143.440 1.00 0.00 C**

**ATOM 78 O TYR A 246 151.634 179.966 144.137 1.00 0.00 O**

**ATOM 79 N TYR A 246 151.542 176.470 143.556 1.00 0.00 N**

**ATOM 80 CB TYR A 246 153.610 177.649 143.001 1.00 0.00 C**

**ATOM 81 CG TYR A 246 154.666 176.809 143.682 1.00 0.00 C**

**ATOM 82 CD1 TYR A 246 154.541 176.457 145.021 1.00 0.00 C**

**ATOM 83 CE1 TYR A 246 155.505 175.693 145.655 1.00 0.00 C**

**ATOM 84 CZ TYR A 246 156.612 175.270 144.949 1.00 0.00 C**

**ATOM 85 OH TYR A 246 157.573 174.508 145.573 1.00 0.00 O**

**ATOM 86 CE2 TYR A 246 156.760 175.606 143.620 1.00 0.00 C**

**ATOM 87 CD2 TYR A 246 155.788 176.369 142.992 1.00 0.00 C**

**ATOM 88 CA TYR A 312 171.302 164.962 122.139 1.00 0.00 C**

**ATOM 89 C TYR A 312 172.263 165.281 123.279 1.00 0.00 C**

**ATOM 90 O TYR A 312 171.859 165.316 124.449 1.00 0.00 O**

**ATOM 91 N TYR A 312 171.486 165.885 121.022 1.00 0.00 N**

**ATOM 92 CB TYR A 312 171.483 163.524 121.655 1.00 0.00 C**

**ATOM 93 CG TYR A 312 171.856 162.528 122.728 1.00 0.00 C**

**ATOM 94 CD1 TYR A 312 170.955 162.178 123.726 1.00 0.00 C**

**ATOM 95 CE1 TYR A 312 171.296 161.260 124.703 1.00 0.00 C**

**ATOM 96 CZ TYR A 312 172.547 160.679 124.687 1.00 0.00 C**

**ATOM 97 OH TYR A 312 172.892 159.766 125.657 1.00 0.00 O**

**ATOM 98 CE2 TYR A 312 173.456 161.006 123.703 1.00 0.00 C**

**ATOM 99 CD2 TYR A 312 173.106 161.923 122.731 1.00 0.00 C**

**ATOM 100 CA TYR A 313 174.502 165.939 123.970 1.00 0.00 C**

**ATOM 101 C TYR A 313 174.130 167.278 124.591 1.00 0.00 C**

**ATOM 102 O TYR A 313 174.119 167.423 125.819 1.00 0.00 O**

**ATOM 103 N TYR A 313 173.534 165.543 122.951 1.00 0.00 N**

**ATOM 104 CB TYR A 313 175.911 166.012 123.374 1.00 0.00 C**

**ATOM 105 CG TYR A 313 176.385 164.754 122.683 1.00 0.00 C**

**ATOM 106 CD1 TYR A 313 176.175 163.505 123.250 1.00 0.00 C**

**ATOM 107 CE1 TYR A 313 176.616 162.354 122.623 1.00 0.00 C**

**ATOM 108 CZ TYR A 313 177.289 162.444 121.423 1.00 0.00 C**

**ATOM 109 OH TYR A 313 177.730 161.299 120.800 1.00 0.00 O**

**ATOM 110 CE2 TYR A 313 177.520 163.675 120.844 1.00 0.00 C**

**ATOM 111 CD2 TYR A 313 177.074 164.821 121.478 1.00 0.00 C**

**ATOM 112 CA TYR A 320 173.152 167.599 133.925 1.00 0.00 C**

**ATOM 113 C TYR A 320 173.511 168.941 134.559 1.00 0.00 C**

**ATOM 114 O TYR A 320 173.965 168.984 135.707 1.00 0.00 O**

**ATOM 115 N TYR A 320 171.979 167.704 133.061 1.00 0.00 N**

**ATOM 116 CB TYR A 320 174.334 167.027 133.144 1.00 0.00 C**

**ATOM 117 CG TYR A 320 174.191 165.550 132.851 1.00 0.00 C**

**ATOM 118 CD1 TYR A 320 173.797 164.662 133.846 1.00 0.00 C**

**ATOM 119 CE1 TYR A 320 173.661 163.310 133.586 1.00 0.00 C**

**ATOM 120 CZ TYR A 320 173.924 162.830 132.321 1.00 0.00 C**

**ATOM 121 OH TYR A 320 173.792 161.486 132.059 1.00 0.00 O**

**ATOM 122 CE2 TYR A 320 174.319 163.690 131.316 1.00 0.00 C**

**ATOM 123 CD2 TYR A 320 174.452 165.041 131.585 1.00 0.00 C**

**ATOM 124 CA TYR A 330 168.722 175.576 146.988 1.00 0.00 C**

**ATOM 125 C TYR A 330 169.519 175.891 148.250 1.00 0.00 C**

**ATOM 126 O TYR A 330 169.049 176.660 149.095 1.00 0.00 O**

**ATOM 127 N TYR A 330 169.497 175.764 145.766 1.00 0.00 N**

**ATOM 128 CB TYR A 330 168.166 174.151 147.051 1.00 0.00 C**

**ATOM 129 CG TYR A 330 166.822 173.985 146.369 1.00 0.00 C**

**ATOM 130 CD1 TYR A 330 166.548 174.608 145.157 1.00 0.00 C**

**ATOM 131 CE1 TYR A 330 165.319 174.460 144.537 1.00 0.00 C**

**ATOM 132 CZ TYR A 330 164.347 173.682 145.132 1.00 0.00 C**

**ATOM 133 OH TYR A 330 163.124 173.528 144.523 1.00 0.00 O**

**ATOM 134 CE2 TYR A 330 164.594 173.057 146.335 1.00 0.00 C**

**ATOM 135 CD2 TYR A 330 165.824 173.212 146.946 1.00 0.00 C**

**ATOM 136 CA VAL A 60 186.670 169.113 121.781 1.00 0.00 C**

**ATOM 137 C VAL A 60 185.771 169.624 122.903 1.00 0.00 C**

**ATOM 138 O VAL A 60 186.287 170.024 123.958 1.00 0.00 O**

**ATOM 139 N VAL A 60 185.946 168.317 120.798 1.00 0.00 N**

**ATOM 140 CB VAL A 60 187.395 170.296 121.107 1.00 0.00 C**

**ATOM 141 CG1 VAL A 60 188.530 169.791 120.230 1.00 0.00 C**

**ATOM 142 CG2 VAL A 60 186.414 171.127 120.294 1.00 0.00 C**

**ATOM 143 CA VAL A 65 181.493 169.551 129.518 1.00 0.00 C**

**ATOM 144 C VAL A 65 181.636 168.449 130.567 1.00 0.00 C**

**ATOM 145 O VAL A 65 181.239 168.634 131.727 1.00 0.00 O**

**ATOM 146 N VAL A 65 182.719 169.678 128.733 1.00 0.00 N**

**ATOM 147 CB VAL A 65 180.259 169.354 128.606 1.00 0.00 C**

**ATOM 148 CG1 VAL A 65 180.354 168.109 127.733 1.00 0.00 C**

**ATOM 149 CG2 VAL A 65 178.982 169.324 129.434 1.00 0.00 C**

**ATOM 150 CA VAL A 68 183.226 171.223 134.168 1.00 0.00 C**

**ATOM 151 C VAL A 68 181.915 170.881 134.867 1.00 0.00 C**

**ATOM 152 O VAL A 68 181.774 171.096 136.078 1.00 0.00 O**

**ATOM 153 N VAL A 68 183.731 170.086 133.399 1.00 0.00 N**

**ATOM 154 CB VAL A 68 183.075 172.454 133.257 1.00 0.00 C**

**ATOM 155 CG1 VAL A 68 182.372 173.582 133.996 1.00 0.00 C**

**ATOM 156 CG2 VAL A 68 184.436 172.913 132.762 1.00 0.00 C**

**ATOM 157 CA VAL A 69 179.615 170.129 134.699 1.00 0.00 C**

**ATOM 158 C VAL A 69 179.671 169.049 135.773 1.00 0.00 C**

**ATOM 159 O VAL A 69 179.072 169.190 136.849 1.00 0.00 O**

**ATOM 160 N VAL A 69 180.939 170.347 134.123 1.00 0.00 N**

**ATOM 161 CB VAL A 69 178.607 169.792 133.585 1.00 0.00 C**

**ATOM 162 CG1 VAL A 69 177.289 169.294 134.166 1.00 0.00 C**

**ATOM 163 CG2 VAL A 69 178.373 171.009 132.705 1.00 0.00 C**

**ATOM 164 CA VAL A 71 183.077 168.559 138.824 1.00 0.00 C**

**ATOM 165 C VAL A 71 182.248 169.567 139.616 1.00 0.00 C**

**ATOM 166 O VAL A 71 182.248 169.531 140.853 1.00 0.00 O**

**ATOM 167 N VAL A 71 182.365 168.149 137.616 1.00 0.00 N**

**ATOM 168 CB VAL A 71 184.499 169.086 138.513 1.00 0.00 C**

**ATOM 169 CG1 VAL A 71 184.498 170.434 137.792 1.00 0.00 C**

**ATOM 170 CG2 VAL A 71 185.336 169.158 139.788 1.00 0.00 C**

**ATOM 171 CA VAL A 72 180.624 171.380 139.635 1.00 0.00 C**

**ATOM 172 C VAL A 72 179.524 170.642 140.389 1.00 0.00 C**

**ATOM 173 O VAL A 72 179.275 170.919 141.570 1.00 0.00 O**

**ATOM 174 N VAL A 72 181.488 170.434 138.934 1.00 0.00 N**

**ATOM 175 CB VAL A 72 180.052 172.415 138.647 1.00 0.00 C**

**ATOM 176 CG1 VAL A 72 178.845 173.128 139.238 1.00 0.00 C**

**ATOM 177 CG2 VAL A 72 181.122 173.431 138.275 1.00 0.00 C**

**ATOM 178 CA VAL A 75 181.630 169.460 144.468 1.00 0.00 C**

**ATOM 179 C VAL A 75 180.442 170.073 145.200 1.00 0.00 C**

**ATOM 180 O VAL A 75 180.418 170.118 146.437 1.00 0.00 O**

**ATOM 181 N VAL A 75 181.178 168.580 143.394 1.00 0.00 N**

**ATOM 182 CB VAL A 75 182.581 170.533 143.902 1.00 0.00 C**

**ATOM 183 CG1 VAL A 75 182.692 171.729 144.839 1.00 0.00 C**

**ATOM 184 CG2 VAL A 75 183.956 169.934 143.649 1.00 0.00 C**

**ATOM 185 CA VAL A 80 175.720 170.272 150.564 1.00 0.00 C**

**ATOM 186 C VAL A 80 175.536 169.125 151.552 1.00 0.00 C**

**ATOM 187 O VAL A 80 174.987 169.314 152.645 1.00 0.00 O**

**ATOM 188 N VAL A 80 177.060 170.240 149.983 1.00 0.00 N**

**ATOM 189 CB VAL A 80 174.653 170.252 149.452 1.00 0.00 C**

**ATOM 190 CG1 VAL A 80 173.276 169.924 150.014 1.00 0.00 C**

**ATOM 191 CG2 VAL A 80 174.613 171.593 148.745 1.00 0.00 C**

**ATOM 192 CA VAL A 83 177.014 170.961 155.629 1.00 0.00 C**

**ATOM 193 C VAL A 83 175.683 170.490 156.210 1.00 0.00 C**

**ATOM 194 O VAL A 83 175.314 170.902 157.312 1.00 0.00 O**

**ATOM 195 N VAL A 83 177.598 169.950 154.752 1.00 0.00 N**

**ATOM 196 CB VAL A 83 176.873 172.321 154.918 1.00 0.00 C**

**ATOM 197 CG1 VAL A 83 178.231 172.813 154.431 1.00 0.00 C**

**ATOM 198 CG2 VAL A 83 175.866 172.262 153.778 1.00 0.00 C**

**ATOM 199 CA VAL A 108 172.626 161.558 138.813 1.00 0.00 C**

**ATOM 200 C VAL A 108 173.896 162.239 138.304 1.00 0.00 C**

**ATOM 201 O VAL A 108 174.252 162.101 137.126 1.00 0.00 O**

**ATOM 202 N VAL A 108 172.716 161.304 140.251 1.00 0.00 N**

**ATOM 203 CB VAL A 108 171.346 162.355 138.482 1.00 0.00 C**

**ATOM 204 CG1 VAL A 108 171.404 163.749 139.044 1.00 0.00 C**

**ATOM 205 CG2 VAL A 108 171.066 162.369 136.979 1.00 0.00 C**

**ATOM 206 CA VAL A 118 178.998 155.642 126.332 1.00 0.00 C**

**ATOM 207 C VAL A 118 178.608 156.613 125.231 1.00 0.00 C**

**ATOM 208 O VAL A 118 178.328 156.178 124.107 1.00 0.00 O**

**ATOM 209 N VAL A 118 179.714 156.307 127.420 1.00 0.00 N**

**ATOM 210 CB VAL A 118 177.747 154.894 126.837 1.00 0.00 C**

**ATOM 211 CG1 VAL A 118 178.125 153.933 127.945 1.00 0.00 C**

**ATOM 212 CG2 VAL A 118 176.658 155.843 127.291 1.00 0.00 C**

**ATOM 213 CA VAL A 129 174.612 143.877 134.834 1.00 0.00 C**

**ATOM 214 C VAL A 129 174.632 145.331 135.288 1.00 0.00 C**

**ATOM 215 O VAL A 129 173.612 145.875 135.727 1.00 0.00 O**

**ATOM 216 N VAL A 129 175.143 143.754 133.477 1.00 0.00 N**

**ATOM 217 CB VAL A 129 175.399 142.979 135.805 1.00 0.00 C**

**ATOM 218 CG1 VAL A 129 174.731 142.967 137.171 1.00 0.00 C**

**ATOM 219 CG2 VAL A 129 175.511 141.568 135.253 1.00 0.00 C**

**ATOM 220 CA VAL A 134 172.573 152.427 135.695 1.00 0.00 C**

**ATOM 221 C VAL A 134 171.265 152.859 135.035 1.00 0.00 C**

**ATOM 222 O VAL A 134 170.801 153.979 135.269 1.00 0.00 O**

**ATOM 223 N VAL A 134 172.594 150.985 135.942 1.00 0.00 N**

**ATOM 224 CB VAL A 134 173.806 152.899 134.891 1.00 0.00 C**

**ATOM 225 CG1 VAL A 134 175.090 152.622 135.662 1.00 0.00 C**

**ATOM 226 CG2 VAL A 134 173.861 152.288 133.507 1.00 0.00 C**

**ATOM 227 CA VAL A 154 152.257 170.445 147.983 1.00 0.00 C**

**ATOM 228 C VAL A 154 152.413 170.784 149.462 1.00 0.00 C**

**ATOM 229 O VAL A 154 151.483 171.309 150.088 1.00 0.00 O**

**ATOM 230 N VAL A 154 153.510 170.684 147.272 1.00 0.00 N**

**ATOM 231 CB VAL A 154 151.795 168.990 147.765 1.00 0.00 C**

**ATOM 232 CG1 VAL A 154 150.561 168.664 148.597 1.00 0.00 C**

**ATOM 233 CG2 VAL A 154 151.504 168.749 146.297 1.00 0.00 C**

**ATOM 234 CA VAL A 160 150.756 178.732 153.401 1.00 0.00 C**

**ATOM 235 C VAL A 160 149.262 178.925 153.160 1.00 0.00 C**

**ATOM 236 O VAL A 160 148.620 179.740 153.821 1.00 0.00 O**

**ATOM 237 N VAL A 160 151.109 177.319 153.535 1.00 0.00 N**

**ATOM 238 CB VAL A 160 151.599 179.392 152.287 1.00 0.00 C**

**ATOM 239 CG1 VAL A 160 150.971 180.700 151.841 1.00 0.00 C**

**ATOM 240 CG2 VAL A 160 153.019 179.624 152.772 1.00 0.00 C**

**ATOM 241 CA VAL A 164 144.593 174.739 160.107 1.00 0.00 C**

**ATOM 242 C VAL A 164 145.216 173.353 160.019 1.00 0.00 C**

**ATOM 243 O VAL A 164 145.603 172.764 161.033 1.00 0.00 O**

**ATOM 244 N VAL A 164 145.140 175.620 159.081 1.00 0.00 N**

**ATOM 245 CB VAL A 164 143.059 174.677 159.988 1.00 0.00 C**

**ATOM 246 CG1 VAL A 164 142.465 173.893 161.146 1.00 0.00 C**

**ATOM 247 CG2 VAL A 164 142.474 176.077 159.927 1.00 0.00 C**

**ATOM 248 CA VAL A 189 158.684 151.497 138.878 1.00 0.00 C**

**ATOM 249 C VAL A 189 160.100 151.105 138.457 1.00 0.00 C**

**ATOM 250 O VAL A 189 160.316 150.584 137.353 1.00 0.00 O**

**ATOM 251 N VAL A 189 158.484 151.236 140.304 1.00 0.00 N**

**ATOM 252 CB VAL A 189 158.363 152.966 138.545 1.00 0.00 C**

**ATOM 253 CG1 VAL A 189 158.439 153.194 137.049 1.00 0.00 C**

**ATOM 254 CG2 VAL A 189 156.976 153.326 139.043 1.00 0.00 C**

**ATOM 255 CA VAL A 195 162.640 143.213 135.075 1.00 0.00 C**

**ATOM 256 C VAL A 195 162.191 143.332 133.626 1.00 0.00 C**

**ATOM 257 O VAL A 195 161.924 142.303 132.986 1.00 0.00 O**

**ATOM 258 N VAL A 195 163.141 144.477 135.615 1.00 0.00 N**

**ATOM 259 CB VAL A 195 161.481 142.629 135.911 1.00 0.00 C**

**ATOM 260 CG1 VAL A 195 161.904 142.446 137.363 1.00 0.00 C**

**ATOM 261 CG2 VAL A 195 160.237 143.491 135.798 1.00 0.00 C**

**ATOM 262 CA VAL A 201 173.105 144.146 119.895 1.00 0.00 C**

**ATOM 263 C VAL A 201 172.498 144.725 118.626 1.00 0.00 C**

**ATOM 264 O VAL A 201 171.586 144.127 118.043 1.00 0.00 O**

**ATOM 265 N VAL A 201 172.032 143.951 120.863 1.00 0.00 N**

**ATOM 266 CB VAL A 201 173.844 142.830 119.590 1.00 0.00 C**

**ATOM 267 CG1 VAL A 201 174.865 143.037 118.480 1.00 0.00 C**

**ATOM 268 CG2 VAL A 201 174.503 142.280 120.843 1.00 0.00 C**

**ATOM 269 CA VAL A 205 176.708 148.834 113.516 1.00 0.00 C**

**ATOM 270 C VAL A 205 177.448 147.576 113.968 1.00 0.00 C**

**ATOM 271 O VAL A 205 178.692 147.565 113.973 1.00 0.00 O**

**ATOM 272 N VAL A 205 175.337 148.524 113.130 1.00 0.00 N**

**ATOM 273 CB VAL A 205 176.710 149.929 114.613 1.00 0.00 C**

**ATOM 274 CG1 VAL A 205 178.112 150.470 114.916 1.00 0.00 C**

**ATOM 275 CG2 VAL A 205 175.777 151.066 114.222 1.00 0.00 C**

**ATOM 276 CA VAL A 207 177.973 146.604 118.298 1.00 0.00 C**

**ATOM 277 C VAL A 207 176.815 146.358 119.253 1.00 0.00 C**

**ATOM 278 O VAL A 207 175.643 146.402 118.873 1.00 0.00 O**

**ATOM 279 N VAL A 207 177.486 146.379 116.946 1.00 0.00 N**

**ATOM 280 CB VAL A 207 178.549 148.023 118.497 1.00 0.00 C**

**ATOM 281 CG1 VAL A 207 179.714 148.264 117.548 1.00 0.00 C**

**ATOM 282 CG2 VAL A 207 177.468 149.069 118.296 1.00 0.00 C**

**ATOM 283 CA VAL A 230 155.264 157.979 132.242 1.00 0.00 C**

**ATOM 284 C VAL A 230 155.462 159.447 131.872 1.00 0.00 C**

**ATOM 285 O VAL A 230 155.825 160.261 132.728 1.00 0.00 O**

**ATOM 286 N VAL A 230 154.218 157.388 131.419 1.00 0.00 N**

**ATOM 287 CB VAL A 230 156.565 157.155 132.150 1.00 0.00 C**

**ATOM 288 CG1 VAL A 230 157.242 157.291 130.799 1.00 0.00 C**

**ATOM 289 CG2 VAL A 230 157.508 157.537 133.262 1.00 0.00 C**

**ATOM 290 CA VAL A 236 151.595 166.662 132.977 1.00 0.00 C**

**ATOM 291 C VAL A 236 151.050 166.841 134.386 1.00 0.00 C**

**ATOM 292 O VAL A 236 150.774 167.964 134.820 1.00 0.00 O**

**ATOM 293 N VAL A 236 152.907 166.031 133.073 1.00 0.00 N**

**ATOM 294 CB VAL A 236 150.629 165.842 132.104 1.00 0.00 C**

**ATOM 295 CG1 VAL A 236 149.207 166.370 132.233 1.00 0.00 C**

**ATOM 296 CG2 VAL A 236 151.080 165.871 130.653 1.00 0.00 C**

**ATOM 297 CA VAL A 239 153.729 170.409 136.347 1.00 0.00 C**

**ATOM 298 C VAL A 239 152.384 171.040 136.695 1.00 0.00 C**

**ATOM 299 O VAL A 239 152.328 172.158 137.225 1.00 0.00 O**

**ATOM 300 N VAL A 239 153.730 168.985 136.673 1.00 0.00 N**

**ATOM 301 CB VAL A 239 154.106 170.619 134.868 1.00 0.00 C**

**ATOM 302 CG1 VAL A 239 153.921 172.069 134.450 1.00 0.00 C**

**ATOM 303 CG2 VAL A 239 155.550 170.206 134.644 1.00 0.00 C**

**ATOM 304 CA VAL A 244 148.126 175.060 140.789 1.00 0.00 C**

**ATOM 305 C VAL A 244 148.349 175.071 142.292 1.00 0.00 C**

**ATOM 306 O VAL A 244 147.458 175.494 143.035 1.00 0.00 O**

**ATOM 307 N VAL A 244 149.335 174.733 140.039 1.00 0.00 N**

**ATOM 308 CB VAL A 244 146.955 174.107 140.470 1.00 0.00 C**

**ATOM 309 CG1 VAL A 244 146.717 174.025 138.968 1.00 0.00 C**

**ATOM 310 CG2 VAL A 244 147.188 172.730 141.082 1.00 0.00 C**

**ATOM 311 CA VAL A 256 146.236 187.839 154.188 1.00 0.00 C**

**ATOM 312 C VAL A 256 146.722 189.259 154.464 1.00 0.00 C**

**ATOM 313 O VAL A 256 147.415 189.876 153.644 1.00 0.00 O**

**ATOM 314 N VAL A 256 146.100 187.622 152.752 1.00 0.00 N**

**ATOM 315 CB VAL A 256 147.179 186.792 154.810 1.00 0.00 C**

**ATOM 316 CG1 VAL A 256 146.532 185.419 154.772 1.00 0.00 C**

**ATOM 317 CG2 VAL A 256 148.500 186.759 154.066 1.00 0.00 C**

**ATOM 318 CA VAL A 276 159.828 180.398 143.372 1.00 0.00 C**

**ATOM 319 C VAL A 276 159.543 180.337 141.870 1.00 0.00 C**

**ATOM 320 O VAL A 276 159.528 179.247 141.276 1.00 0.00 O**

**ATOM 321 N VAL A 276 160.616 181.583 143.698 1.00 0.00 N**

**ATOM 322 CB VAL A 276 158.549 180.336 144.237 1.00 0.00 C**

**ATOM 323 CG1 VAL A 276 157.549 181.424 143.883 1.00 0.00 C**

**ATOM 324 CG2 VAL A 276 157.909 178.971 144.137 1.00 0.00 C**

**ATOM 325 CA VAL A 278 162.826 181.029 138.799 1.00 0.00 C**

**ATOM 326 C VAL A 278 163.135 179.557 139.077 1.00 0.00 C**

**ATOM 327 O VAL A 278 163.715 178.879 138.223 1.00 0.00 O**

**ATOM 328 N VAL A 278 161.623 181.447 139.510 1.00 0.00 N**

**ATOM 329 CB VAL A 278 164.019 181.955 139.122 1.00 0.00 C**

**ATOM 330 CG1 VAL A 278 164.593 181.702 140.505 1.00 0.00 C**

**ATOM 331 CG2 VAL A 278 165.102 181.835 138.055 1.00 0.00 C**

**ATOM 332 CA VAL A 279 162.811 177.575 140.454 1.00 0.00 C**

**ATOM 333 C VAL A 279 161.910 176.826 139.476 1.00 0.00 C**

**ATOM 334 O VAL A 279 162.333 175.854 138.829 1.00 0.00 O**

**ATOM 335 N VAL A 279 162.717 179.021 140.232 1.00 0.00 N**

**ATOM 336 CB VAL A 279 162.475 177.227 141.916 1.00 0.00 C**

**ATOM 337 CG1 VAL A 279 162.319 175.726 142.084 1.00 0.00 C**

**ATOM 338 CG2 VAL A 279 163.574 177.717 142.837 1.00 0.00 C**

**ATOM 339 CA VAL A 280 159.743 176.681 138.371 1.00 0.00 C**

**ATOM 340 C VAL A 280 160.284 176.834 136.952 1.00 0.00 C**

**ATOM 341 O VAL A 280 160.197 175.909 136.134 1.00 0.00 O**

**ATOM 342 N VAL A 280 160.661 177.285 139.337 1.00 0.00 N**

**ATOM 343 CB VAL A 280 158.336 177.294 138.525 1.00 0.00 C**

**ATOM 344 CG1 VAL A 280 157.417 176.874 137.386 1.00 0.00 C**

**ATOM 345 CG2 VAL A 280 157.731 176.887 139.856 1.00 0.00 C**

**ATOM 346 CA VAL A 282 164.671 176.270 135.606 1.00 0.00 C**

**ATOM 347 C VAL A 282 164.222 174.815 135.530 1.00 0.00 C**

**ATOM 348 O VAL A 282 164.719 174.060 134.684 1.00 0.00 O**

**ATOM 349 N VAL A 282 163.540 177.144 135.909 1.00 0.00 N**

**ATOM 350 CB VAL A 282 165.842 176.471 136.595 1.00 0.00 C**

**ATOM 351 CG1 VAL A 282 166.373 177.896 136.504 1.00 0.00 C**

**ATOM 352 CG2 VAL A 282 165.461 176.125 138.026 1.00 0.00 C**

**ATOM 353 CA VAL A 284 160.568 173.714 133.141 1.00 0.00 C**

**ATOM 354 C VAL A 284 161.561 173.685 131.985 1.00 0.00 C**

**ATOM 355 O VAL A 284 161.498 172.805 131.122 1.00 0.00 O**

**ATOM 356 N VAL A 284 161.266 173.834 134.418 1.00 0.00 N**

**ATOM 357 CB VAL A 284 159.540 174.851 132.984 1.00 0.00 C**

**ATOM 358 CG1 VAL A 284 159.158 175.044 131.523 1.00 0.00 C**

**ATOM 359 CG2 VAL A 284 158.300 174.554 133.814 1.00 0.00 C**

**ATOM 360 CA VAL A 285 163.461 174.661 130.849 1.00 0.00 C**

**ATOM 361 C VAL A 285 164.511 173.564 130.916 1.00 0.00 C**

**ATOM 362 O VAL A 285 165.261 173.387 129.950 1.00 0.00 O**

**ATOM 363 N VAL A 285 162.516 174.619 131.961 1.00 0.00 N**

**ATOM 364 CB VAL A 285 164.178 176.023 130.741 1.00 0.00 C**

**ATOM 365 CG1 VAL A 285 163.169 177.162 130.694 1.00 0.00 C**

**ATOM 366 CG2 VAL A 285 165.176 176.201 131.876 1.00 0.00 C**

**ATOM 367 CA VAL A 296 158.733 163.468 118.971 1.00 0.00 C**

**ATOM 368 C VAL A 296 159.421 162.262 118.341 1.00 0.00 C**

**ATOM 369 O VAL A 296 158.955 161.724 117.329 1.00 0.00 O**

**ATOM 370 N VAL A 296 158.664 163.313 120.421 1.00 0.00 N**

**ATOM 371 CB VAL A 296 159.435 164.794 118.617 1.00 0.00 C**

**ATOM 372 CG1 VAL A 296 159.879 164.814 117.168 1.00 0.00 C**

**ATOM 373 CG2 VAL A 296 158.501 165.967 118.876 1.00 0.00 C**

**ATOM 374 CA THR A 63 185.870 166.746 127.385 1.00 0.00 C**

**ATOM 375 C THR A 63 185.615 167.951 128.279 1.00 0.00 C**

**ATOM 376 O THR A 63 185.661 167.839 129.509 1.00 0.00 O**

**ATOM 377 N THR A 63 184.952 166.728 126.251 1.00 0.00 N**

**ATOM 378 CB THR A 63 187.316 166.744 126.891 1.00 0.00 C**

**ATOM 379 OG1 THR A 63 187.611 168.001 126.268 1.00 0.00 O**

**ATOM 380 CG2 THR A 63 187.533 165.625 125.885 1.00 0.00 C**

**ATOM 381 CA THR A 88 172.295 171.213 160.772 1.00 0.00 C**

**ATOM 382 C THR A 88 171.908 169.770 161.074 1.00 0.00 C**

**ATOM 383 O THR A 88 172.764 168.886 161.177 1.00 0.00 O**

**ATOM 384 N THR A 88 173.689 171.225 160.353 1.00 0.00 N**

**ATOM 385 CB THR A 88 171.395 171.851 159.706 1.00 0.00 C**

**ATOM 386 OG1 THR A 88 170.023 171.714 160.093 1.00 0.00 O**

**ATOM 387 CG2 THR A 88 171.607 171.183 158.362 1.00 0.00 C**

**ATOM 388 CA THR A 92 162.183 170.150 160.048 1.00 0.00 C**

**ATOM 389 C THR A 92 161.678 169.165 158.997 1.00 0.00 C**

**ATOM 390 O THR A 92 162.334 168.177 158.656 1.00 0.00 O**

**ATOM 391 N THR A 92 163.371 169.640 160.714 1.00 0.00 N**

**ATOM 392 CB THR A 92 162.467 171.505 159.402 1.00 0.00 C**

**ATOM 393 OG1 THR A 92 163.478 171.355 158.398 1.00 0.00 O**

**ATOM 394 CG2 THR A 92 162.942 172.501 160.447 1.00 0.00 C**

**ATOM 395 CA THR A 94 161.794 170.287 154.594 1.00 0.00 C**

**ATOM 396 C THR A 94 163.272 169.930 154.551 1.00 0.00 C**

**ATOM 397 O THR A 94 163.833 169.819 153.454 1.00 0.00 O**

**ATOM 398 N THR A 94 161.167 169.986 155.874 1.00 0.00 N**

**ATOM 399 CB THR A 94 161.637 171.773 154.253 1.00 0.00 C**

**ATOM 400 OG1 THR A 94 162.269 172.567 155.265 1.00 0.00 O**

**ATOM 401 CG2 THR A 94 160.167 172.145 154.160 1.00 0.00 C**

**ATOM 402 CA THR A 109 175.941 163.438 138.793 1.00 0.00 C**

**ATOM 403 C THR A 109 176.946 162.296 138.706 1.00 0.00 C**

**ATOM 404 O THR A 109 177.862 162.338 137.877 1.00 0.00 O**

**ATOM 405 N THR A 109 174.622 162.949 139.173 1.00 0.00 N**

**ATOM 406 CB THR A 109 176.416 164.497 139.791 1.00 0.00 C**

**ATOM 407 OG1 THR A 109 175.339 165.395 140.083 1.00 0.00 O**

**ATOM 408 CG2 THR A 109 177.579 165.296 139.217 1.00 0.00 C**

**ATOM 409 CA THR A 110 177.659 160.102 139.484 1.00 0.00 C**

**ATOM 410 C THR A 110 177.428 159.315 138.198 1.00 0.00 C**

**ATOM 411 O THR A 110 178.375 158.769 137.620 1.00 0.00 O**

**ATOM 412 N THR A 110 176.789 161.273 139.552 1.00 0.00 N**

**ATOM 413 CB THR A 110 177.437 159.214 140.709 1.00 0.00 C**

**ATOM 414 OG1 THR A 110 177.480 160.018 141.894 1.00 0.00 O**

**ATOM 415 CG2 THR A 110 178.514 158.142 140.805 1.00 0.00 C**

**ATOM 416 CA THR A 111 175.902 158.558 136.472 1.00 0.00 C**

**ATOM 417 C THR A 111 176.280 159.376 135.240 1.00 0.00 C**

**ATOM 418 O THR A 111 176.251 158.836 134.125 1.00 0.00 O**

**ATOM 419 N THR A 111 176.180 159.245 137.731 1.00 0.00 N**

**ATOM 420 CB THR A 111 174.423 158.175 136.390 1.00 0.00 C**

**ATOM 421 OG1 THR A 111 173.619 159.361 136.382 1.00 0.00 O**

**ATOM 422 CG2 THR A 111 174.031 157.306 137.575 1.00 0.00 C**

**ATOM 423 CA THR A 117 181.748 157.147 128.497 1.00 0.00 C**

**ATOM 424 C THR A 117 181.052 156.304 127.431 1.00 0.00 C**

**ATOM 425 O THR A 117 181.708 155.659 126.605 1.00 0.00 O**

**ATOM 426 N THR A 117 180.853 158.180 129.007 1.00 0.00 N**

**ATOM 427 CB THR A 117 182.244 156.282 129.661 1.00 0.00 C**

**ATOM 428 OG1 THR A 117 182.666 157.134 130.732 1.00 0.00 O**

**ATOM 429 CG2 THR A 117 183.443 155.434 129.246 1.00 0.00 C**

**ATOM 430 CA THR A 144 162.078 160.397 142.789 1.00 0.00 C**

**ATOM 431 C THR A 144 162.420 161.877 142.926 1.00 0.00 C**

**ATOM 432 O THR A 144 161.904 162.552 143.822 1.00 0.00 O**

**ATOM 433 N THR A 144 162.137 159.972 141.396 1.00 0.00 N**

**ATOM 434 CB THR A 144 162.978 159.520 143.670 1.00 0.00 C**

**ATOM 435 OG1 THR A 144 162.674 159.772 145.046 1.00 0.00 O**

**ATOM 436 CG2 THR A 144 164.463 159.784 143.438 1.00 0.00 C**

**ATOM 437 CA THR A 148 160.108 165.141 145.808 1.00 0.00 C**

**ATOM 438 C THR A 148 159.869 166.617 145.515 1.00 0.00 C**

**ATOM 439 O THR A 148 159.379 167.344 146.387 1.00 0.00 O**

**ATOM 440 N THR A 148 159.752 164.290 144.677 1.00 0.00 N**

**ATOM 441 CB THR A 148 161.568 164.929 146.233 1.00 0.00 C**

**ATOM 442 OG1 THR A 148 162.437 165.099 145.106 1.00 0.00 O**

**ATOM 443 CG2 THR A 148 161.765 163.558 146.870 1.00 0.00 C**

**ATOM 444 CA THR A 150 156.142 168.042 143.696 1.00 0.00 C**

**ATOM 445 C THR A 150 155.582 167.908 145.107 1.00 0.00 C**

**ATOM 446 O THR A 150 154.682 168.666 145.488 1.00 0.00 O**

**ATOM 447 N THR A 150 157.577 167.787 143.633 1.00 0.00 N**

**ATOM 448 CB THR A 150 155.387 167.099 142.762 1.00 0.00 C**

**ATOM 449 OG1 THR A 150 155.706 165.741 143.088 1.00 0.00 O**

**ATOM 450 CG2 THR A 150 155.769 167.382 141.335 1.00 0.00 C**

**ATOM 451 CA THR A 171 155.063 166.649 161.438 1.00 0.00 C**

**ATOM 452 C THR A 171 156.193 165.921 160.711 1.00 0.00 C**

**ATOM 453 O THR A 171 156.039 165.532 159.549 1.00 0.00 O**

**ATOM 454 N THR A 171 154.188 167.286 160.470 1.00 0.00 N**

**ATOM 455 CB THR A 171 154.279 165.664 162.303 1.00 0.00 C**

**ATOM 456 OG1 THR A 171 153.503 164.801 161.464 1.00 0.00 O**

**ATOM 457 CG2 THR A 171 153.353 166.408 163.250 1.00 0.00 C**

**ATOM 458 CA THR A 199 170.776 144.693 126.640 1.00 0.00 C**

**ATOM 459 C THR A 199 170.401 144.145 125.271 1.00 0.00 C**

**ATOM 460 O THR A 199 169.582 143.230 125.149 1.00 0.00 O**

**ATOM 461 N THR A 199 169.744 144.325 127.599 1.00 0.00 N**

**ATOM 462 CB THR A 199 172.157 144.175 127.068 1.00 0.00 C**

**ATOM 463 OG1 THR A 199 173.154 144.652 126.156 1.00 0.00 O**

**ATOM 464 CG2 THR A 199 172.181 142.652 127.094 1.00 0.00 C**

**ATOM 465 CA THR A 247 149.892 180.131 142.103 1.00 0.00 C**

**ATOM 466 C THR A 247 148.808 180.360 143.155 1.00 0.00 C**

**ATOM 467 O THR A 247 148.509 181.512 143.488 1.00 0.00 O**

**ATOM 468 N THR A 247 150.665 178.926 142.395 1.00 0.00 N**

**ATOM 469 CB THR A 247 149.313 180.088 140.677 1.00 0.00 C**

**ATOM 470 OG1 THR A 247 148.917 181.408 140.284 1.00 0.00 O**

**ATOM 471 CG2 THR A 247 148.103 179.164 140.549 1.00 0.00 C**

**ATOM 472 CA THR A 273 157.825 185.149 144.292 1.00 0.00 C**

**ATOM 473 C THR A 273 158.785 184.968 143.122 1.00 0.00 C**

**ATOM 474 O THR A 273 158.630 184.037 142.326 1.00 0.00 O**

**ATOM 475 N THR A 273 158.535 185.537 145.506 1.00 0.00 N**

**ATOM 476 CB THR A 273 156.754 186.187 143.960 1.00 0.00 C**

**ATOM 477 OG1 THR A 273 155.952 186.432 145.121 1.00 0.00 O**

**ATOM 478 CG2 THR A 273 155.852 185.690 142.840 1.00 0.00 C**

**ATOM 479 CA THR A 288 160.873 170.268 128.861 1.00 0.00 C**

**ATOM 480 C THR A 288 161.513 169.691 127.597 1.00 0.00 C**

**ATOM 481 O THR A 288 160.895 168.797 126.980 1.00 0.00 O**

**ATOM 482 N THR A 288 161.742 170.183 130.035 1.00 0.00 N**

**ATOM 483 CB THR A 288 160.424 171.725 128.670 1.00 0.00 C**

**ATOM 484 OG1 THR A 288 159.727 172.180 129.836 1.00 0.00 O**

**ATOM 485 CG2 THR A 288 159.540 171.886 127.455 1.00 0.00 C**

**ATOM 486 CA THR A 302 160.724 160.990 113.023 1.00 0.00 C**

**ATOM 487 C THR A 302 162.156 160.695 112.597 1.00 0.00 C**

**ATOM 488 O THR A 302 162.708 161.412 111.755 1.00 0.00 O**

**ATOM 489 N THR A 302 160.074 159.798 113.565 1.00 0.00 N**

**ATOM 490 CB THR A 302 160.711 162.148 114.027 1.00 0.00 C**

**ATOM 491 OG1 THR A 302 161.283 161.726 115.273 1.00 0.00 O**

**ATOM 492 CG2 THR A 302 159.300 162.686 114.234 1.00 0.00 C**

**ATOM 493 CA THR A 306 173.492 164.599 112.038 1.00 0.00 C**

**ATOM 494 C THR A 306 173.216 165.656 113.099 1.00 0.00 C**

**ATOM 495 O THR A 306 174.045 165.888 113.985 1.00 0.00 O**

**ATOM 496 N THR A 306 172.250 164.036 111.513 1.00 0.00 N**

**ATOM 497 CB THR A 306 174.332 165.205 110.911 1.00 0.00 C**

**ATOM 498 OG1 THR A 306 173.616 166.289 110.307 1.00 0.00 O**

**ATOM 499 CG2 THR A 306 174.643 164.157 109.856 1.00 0.00 C**

**ATOM 500 CA THR A 321 173.494 171.370 134.424 1.00 0.00 C**

**ATOM 501 C THR A 321 172.455 171.658 135.502 1.00 0.00 C**

**ATOM 502 O THR A 321 172.777 172.277 136.525 1.00 0.00 O**

**ATOM 503 N THR A 321 173.295 170.048 133.843 1.00 0.00 N**

**ATOM 504 CB THR A 321 173.446 172.427 133.314 1.00 0.00 C**

**ATOM 505 OG1 THR A 321 174.475 172.152 132.354 1.00 0.00 O**

**ATOM 506 CG2 THR A 321 173.668 173.826 133.867 1.00 0.00 C**

**ATOM 507 CA GLY A 73 177.838 168.903 140.404 1.00 0.00 C**

**ATOM 508 C GLY A 73 178.349 168.088 141.577 1.00 0.00 C**

**ATOM 509 O GLY A 73 177.733 168.071 142.647 1.00 0.00 O**

**ATOM 510 N GLY A 73 178.884 169.664 139.739 1.00 0.00 N**

**ATOM 511 CA GLY A 76 178.249 171.097 145.088 1.00 0.00 C**

**ATOM 512 C GLY A 76 177.488 170.101 145.940 1.00 0.00 C**

**ATOM 513 O GLY A 76 177.102 170.410 147.070 1.00 0.00 O**

**ATOM 514 N GLY A 76 179.425 170.523 144.454 1.00 0.00 N**

**ATOM 515 CA GLY A 127 178.851 144.864 131.307 1.00 0.00 C**

**ATOM 516 C GLY A 127 177.351 145.092 131.441 1.00 0.00 C**

**ATOM 517 O GLY A 127 176.878 146.170 131.825 1.00 0.00 O**

**ATOM 518 N GLY A 127 179.545 146.062 130.891 1.00 0.00 N**

**ATOM 519 CA GLY A 190 162.441 150.888 139.040 1.00 0.00 C**

**ATOM 520 C GLY A 190 162.609 149.381 138.952 1.00 0.00 C**

**ATOM 521 O GLY A 190 163.383 148.884 138.130 1.00 0.00 O**

**ATOM 522 N GLY A 190 161.080 151.305 139.342 1.00 0.00 N**

**ATOM 523 CA GLY A 197 165.196 146.222 130.766 1.00 0.00 C**

**ATOM 524 C GLY A 197 166.156 145.117 130.374 1.00 0.00 C**

**ATOM 525 O GLY A 197 165.840 143.927 130.436 1.00 0.00 O**

**ATOM 526 N GLY A 197 164.042 145.719 131.481 1.00 0.00 N**

**ATOM 527 CA GLY A 198 168.374 144.569 129.582 1.00 0.00 C**

**ATOM 528 C GLY A 198 169.429 145.102 128.634 1.00 0.00 C**

**ATOM 529 O GLY A 198 169.959 146.200 128.832 1.00 0.00 O**

**ATOM 530 N GLY A 198 167.353 145.527 129.961 1.00 0.00 N**

**ATOM 531 CA GLY A 261 152.801 195.088 151.247 1.00 0.00 C**

**ATOM 532 C GLY A 261 153.128 196.225 152.191 1.00 0.00 C**

**ATOM 533 O GLY A 261 152.260 196.737 152.906 1.00 0.00 O**

**ATOM 534 N GLY A 261 152.122 193.942 151.816 1.00 0.00 N**

**ATOM 535 CA GLY A 300 154.893 157.202 114.893 1.00 0.00 C**

**ATOM 536 C GLY A 300 156.342 156.990 114.503 1.00 0.00 C**

**ATOM 537 O GLY A 300 156.887 155.904 114.730 1.00 0.00 O**

**ATOM 538 N GLY A 300 154.700 157.968 116.109 1.00 0.00 N**

**ATOM 539 CA GLY A 319 169.603 168.059 132.648 1.00 0.00 C**

**ATOM 540 C GLY A 319 170.774 167.911 133.599 1.00 0.00 C**

**ATOM 541 O GLY A 319 170.603 168.001 134.819 1.00 0.00 O**

**ATOM 542 N GLY A 319 169.928 168.813 131.450 1.00 0.00 N**

**ATOM 543 CA PHE A 70 180.553 166.908 136.499 1.00 0.00 C**

**ATOM 544 C PHE A 70 181.286 167.364 137.756 1.00 0.00 C**

**ATOM 545 O PHE A 70 180.871 167.015 138.868 1.00 0.00 O**

**ATOM 546 N PHE A 70 180.432 167.982 135.520 1.00 0.00 N**

**ATOM 547 CB PHE A 70 181.251 165.700 135.879 1.00 0.00 C**

**ATOM 548 CG PHE A 70 181.642 164.646 136.875 1.00 0.00 C**

**ATOM 549 CD1 PHE A 70 180.675 163.897 137.525 1.00 0.00 C**

**ATOM 550 CE1 PHE A 70 181.032 162.924 138.441 1.00 0.00 C**

**ATOM 551 CZ PHE A 70 182.367 162.689 138.713 1.00 0.00 C**

**ATOM 552 CE2 PHE A 70 183.341 163.428 138.069 1.00 0.00 C**

**ATOM 553 CD2 PHE A 70 182.977 164.400 137.155 1.00 0.00 C**

**ATOM 554 CA PHE A 82 178.812 167.855 154.370 1.00 0.00 C**

**ATOM 555 C PHE A 82 178.187 168.886 155.304 1.00 0.00 C**

**ATOM 556 O PHE A 82 178.219 168.717 156.529 1.00 0.00 O**

**ATOM 557 N PHE A 82 177.961 167.586 153.215 1.00 0.00 N**

**ATOM 558 CB PHE A 82 180.193 168.319 153.907 1.00 0.00 C**

**ATOM 559 CA PHE A 99 168.637 165.842 151.962 1.00 0.00 C**

**ATOM 560 C PHE A 99 168.400 164.829 150.841 1.00 0.00 C**

**ATOM 561 O PHE A 99 169.350 164.388 150.176 1.00 0.00 O**

**ATOM 562 N PHE A 99 167.682 166.947 151.899 1.00 0.00 N**

**ATOM 563 CB PHE A 99 168.541 165.185 153.341 1.00 0.00 C**

**ATOM 564 CG PHE A 99 169.218 163.851 153.443 1.00 0.00 C**

**ATOM 565 CD1 PHE A 99 170.580 163.727 153.229 1.00 0.00 C**

**ATOM 566 CE1 PHE A 99 171.199 162.498 153.336 1.00 0.00 C**

**ATOM 567 CZ PHE A 99 170.459 161.378 153.669 1.00 0.00 C**

**ATOM 568 CE2 PHE A 99 169.102 161.491 153.890 1.00 0.00 C**

**ATOM 569 CD2 PHE A 99 168.490 162.722 153.781 1.00 0.00 C**

**ATOM 570 CA PHE A 114 178.860 156.727 132.972 1.00 0.00 C**

**ATOM 571 C PHE A 114 178.150 156.914 131.637 1.00 0.00 C**

**ATOM 572 O PHE A 114 178.333 156.115 130.712 1.00 0.00 O**

**ATOM 573 N PHE A 114 179.278 158.008 133.533 1.00 0.00 N**

**ATOM 574 CB PHE A 114 177.959 155.988 133.961 1.00 0.00 C**

**ATOM 575 CA PHE A 126 181.236 147.811 131.097 1.00 0.00 C**

**ATOM 576 C PHE A 126 180.679 146.444 131.456 1.00 0.00 C**

**ATOM 577 O PHE A 126 181.288 145.724 132.257 1.00 0.00 O**

**ATOM 578 N PHE A 126 181.503 147.970 129.672 1.00 0.00 N**

**ATOM 579 CB PHE A 126 180.261 148.898 131.574 1.00 0.00 C**

**ATOM 580 CG PHE A 126 180.778 150.293 131.405 1.00 0.00 C**

**ATOM 581 CD1 PHE A 126 182.004 150.660 131.935 1.00 0.00 C**

**ATOM 582 CE1 PHE A 126 182.482 151.948 131.782 1.00 0.00 C**

**ATOM 583 CZ PHE A 126 181.730 152.884 131.099 1.00 0.00 C**

**ATOM 584 CE2 PHE A 126 180.505 152.531 130.568 1.00 0.00 C**

**ATOM 585 CD2 PHE A 126 180.034 151.242 130.724 1.00 0.00 C**

**ATOM 586 CA PHE A 143 161.215 159.803 139.137 1.00 0.00 C**

**ATOM 587 C PHE A 143 161.161 160.340 140.562 1.00 0.00 C**

**ATOM 588 O PHE A 143 160.251 161.100 140.914 1.00 0.00 O**

**ATOM 589 N PHE A 143 162.566 159.889 138.589 1.00 0.00 N**

**ATOM 590 CB PHE A 143 160.710 158.359 139.101 1.00 0.00 C**

**ATOM 591 CG PHE A 143 160.583 157.789 137.719 1.00 0.00 C**

**ATOM 592 CD1 PHE A 143 160.413 158.616 136.621 1.00 0.00 C**

**ATOM 593 CE1 PHE A 143 160.311 158.087 135.355 1.00 0.00 C**

**ATOM 594 CZ PHE A 143 160.384 156.721 135.168 1.00 0.00 C**

**ATOM 595 CE2 PHE A 143 160.559 155.889 136.248 1.00 0.00 C**

**ATOM 596 CD2 PHE A 143 160.652 156.419 137.517 1.00 0.00 C**

**ATOM 597 CA PHE A 147 158.064 163.377 143.095 1.00 0.00 C**

**ATOM 598 C PHE A 147 158.470 164.027 144.416 1.00 0.00 C**

**ATOM 599 O PHE A 147 157.578 164.297 145.224 1.00 0.00 O**

**ATOM 600 N PHE A 147 159.059 163.518 142.037 1.00 0.00 N**

**ATOM 601 CB PHE A 147 157.715 161.904 143.320 1.00 0.00 C**

**ATOM 602 CG PHE A 147 156.776 161.340 142.288 1.00 0.00 C**

**ATOM 603 CD1 PHE A 147 155.424 161.641 142.335 1.00 0.00 C**

**ATOM 604 CE1 PHE A 147 154.552 161.129 141.394 1.00 0.00 C**

**ATOM 605 CZ PHE A 147 155.025 160.301 140.396 1.00 0.00 C**

**ATOM 606 CE2 PHE A 147 156.368 159.990 140.340 1.00 0.00 C**

**ATOM 607 CD2 PHE A 147 157.234 160.505 141.284 1.00 0.00 C**

**ATOM 608 CA PHE A 169 150.115 167.516 158.594 1.00 0.00 C**

**ATOM 609 C PHE A 169 151.608 167.643 158.331 1.00 0.00 C**

**ATOM 610 O PHE A 169 152.151 166.827 157.582 1.00 0.00 O**

**ATOM 611 N PHE A 169 149.656 168.193 159.796 1.00 0.00 N**

**ATOM 612 CB PHE A 169 149.360 168.041 157.366 1.00 0.00 C**

**ATOM 613 CG PHE A 169 149.150 167.009 156.299 1.00 0.00 C**

**ATOM 614 CD1 PHE A 169 148.095 166.115 156.382 1.00 0.00 C**

**ATOM 615 CE1 PHE A 169 147.897 165.160 155.400 1.00 0.00 C**

**ATOM 616 CZ PHE A 169 148.759 165.091 154.323 1.00 0.00 C**

**ATOM 617 CE2 PHE A 169 149.816 165.976 154.229 1.00 0.00 C**

**ATOM 618 CD2 PHE A 169 150.008 166.928 155.214 1.00 0.00 C**

**ATOM 619 CA PHE A 214 160.816 145.329 124.746 1.00 0.00 C**

**ATOM 620 C PHE A 214 160.748 144.779 123.330 1.00 0.00 C**

**ATOM 621 O PHE A 214 161.034 145.522 122.367 1.00 0.00 O**

**ATOM 622 N PHE A 214 162.226 145.498 125.092 1.00 0.00 N**

**ATOM 623 CB PHE A 214 160.054 146.646 124.862 1.00 0.00 C**

**ATOM 624 CA PHE A 225 151.470 152.889 126.360 1.00 0.00 C**

**ATOM 625 C PHE A 225 152.226 152.961 127.679 1.00 0.00 C**

**ATOM 626 O PHE A 225 151.627 153.307 128.703 1.00 0.00 O**

**ATOM 627 N PHE A 225 152.359 152.906 125.202 1.00 0.00 N**

**ATOM 628 CB PHE A 225 150.576 151.651 126.329 1.00 0.00 C**

**ATOM 629 CG PHE A 225 149.392 151.782 125.412 1.00 0.00 C**

**ATOM 630 CD1 PHE A 225 148.957 153.031 124.996 1.00 0.00 C**

**ATOM 631 CE1 PHE A 225 147.865 153.157 124.158 1.00 0.00 C**

**ATOM 632 CZ PHE A 225 147.197 152.029 123.723 1.00 0.00 C**

**ATOM 633 CE2 PHE A 225 147.620 150.778 124.130 1.00 0.00 C**

**ATOM 634 CD2 PHE A 225 148.713 150.659 124.971 1.00 0.00 C**

**ATOM 635 CA PHE A 231 155.216 161.228 130.233 1.00 0.00 C**

**ATOM 636 C PHE A 231 154.173 162.058 130.969 1.00 0.00 C**

**ATOM 637 O PHE A 231 154.481 163.140 131.477 1.00 0.00 O**

**ATOM 638 N PHE A 231 155.152 159.826 130.628 1.00 0.00 N**

**ATOM 639 CB PHE A 231 155.034 161.359 128.720 1.00 0.00 C**

**ATOM 640 CG PHE A 231 154.971 162.783 128.237 1.00 0.00 C**

**ATOM 641 CD1 PHE A 231 156.129 163.532 128.098 1.00 0.00 C**

**ATOM 642 CE1 PHE A 231 156.075 164.842 127.655 1.00 0.00 C**

**ATOM 643 CZ PHE A 231 154.856 165.414 127.342 1.00 0.00 C**

**ATOM 644 CE2 PHE A 231 153.694 164.678 127.474 1.00 0.00 C**

**ATOM 645 CD2 PHE A 231 153.755 163.371 127.917 1.00 0.00 C**

**ATOM 646 CA PHE A 233 152.863 161.104 135.233 1.00 0.00 C**

**ATOM 647 C PHE A 233 154.174 161.753 135.656 1.00 0.00 C**

**ATOM 648 O PHE A 233 154.433 161.845 136.860 1.00 0.00 O**

**ATOM 649 N PHE A 233 152.610 161.188 133.800 1.00 0.00 N**

**ATOM 650 CB PHE A 233 152.851 159.642 135.688 1.00 0.00 C**

**ATOM 651 CG PHE A 233 151.472 159.078 135.903 1.00 0.00 C**

**ATOM 652 CD1 PHE A 233 150.342 159.853 135.687 1.00 0.00 C**

**ATOM 653 CE1 PHE A 233 149.077 159.331 135.887 1.00 0.00 C**

**ATOM 654 CZ PHE A 233 148.929 158.023 136.309 1.00 0.00 C**

**ATOM 655 CE2 PHE A 233 150.044 157.241 136.529 1.00 0.00 C**

**ATOM 656 CD2 PHE A 233 151.308 157.768 136.326 1.00 0.00 C**

**ATOM 657 CA PHE A 235 155.385 166.125 133.171 1.00 0.00 C**

**ATOM 658 C PHE A 235 154.014 166.771 133.028 1.00 0.00 C**

**ATOM 659 O PHE A 235 153.958 167.993 132.857 1.00 0.00 O**

**ATOM 660 N PHE A 235 155.370 164.751 133.659 1.00 0.00 N**

**ATOM 661 CB PHE A 235 156.111 166.196 131.823 1.00 0.00 C**

**ATOM 662 CG PHE A 235 156.711 167.535 131.532 1.00 0.00 C**

**ATOM 663 CD1 PHE A 235 157.847 167.955 132.201 1.00 0.00 C**

**ATOM 664 CE1 PHE A 235 158.403 169.186 131.939 1.00 0.00 C**

**ATOM 665 CZ PHE A 235 157.826 170.011 130.996 1.00 0.00 C**

**ATOM 666 CE2 PHE A 235 156.697 169.604 130.318 1.00 0.00 C**

**ATOM 667 CD2 PHE A 235 156.145 168.371 130.585 1.00 0.00 C**

**ATOM 668 CA PHE A 283 162.701 173.058 136.236 1.00 0.00 C**

**ATOM 669 C PHE A 283 162.037 172.845 134.880 1.00 0.00 C**

**ATOM 670 O PHE A 283 162.243 171.806 134.234 1.00 0.00 O**

**ATOM 671 N PHE A 283 163.244 174.410 136.354 1.00 0.00 N**

**ATOM 672 CB PHE A 283 161.710 172.794 137.370 1.00 0.00 C**

**ATOM 673 CG PHE A 283 160.812 171.610 137.136 1.00 0.00 C**

**ATOM 674 CD1 PHE A 283 161.294 170.321 137.286 1.00 0.00 C**

**ATOM 675 CE1 PHE A 283 160.471 169.228 137.079 1.00 0.00 C**

**ATOM 676 CZ PHE A 283 159.150 169.417 136.727 1.00 0.00 C**

**ATOM 677 CE2 PHE A 283 158.653 170.697 136.580 1.00 0.00 C**

**ATOM 678 CD2 PHE A 283 159.480 171.786 136.789 1.00 0.00 C**

**ATOM 679 CA PHE A 293 162.142 165.699 122.004 1.00 0.00 C**

**ATOM 680 C PHE A 293 162.021 164.183 121.893 1.00 0.00 C**

**ATOM 681 O PHE A 293 161.903 163.643 120.785 1.00 0.00 O**

**ATOM 682 N PHE A 293 161.280 166.238 123.053 1.00 0.00 N**

**ATOM 683 CB PHE A 293 163.592 166.102 122.269 1.00 0.00 C**

**ATOM 684 CG PHE A 293 164.533 165.732 121.163 1.00 0.00 C**

**ATOM 685 CD1 PHE A 293 164.511 166.417 119.958 1.00 0.00 C**

**ATOM 686 CE1 PHE A 293 165.380 166.079 118.935 1.00 0.00 C**

**ATOM 687 CZ PHE A 293 166.283 165.047 119.110 1.00 0.00 C**

**ATOM 688 CE2 PHE A 293 166.315 164.356 120.308 1.00 0.00 C**

**ATOM 689 CD2 PHE A 293 165.442 164.699 121.326 1.00 0.00 C**

**ATOM 690 CA PHE A 314 173.467 169.595 124.265 1.00 0.00 C**

**ATOM 691 C PHE A 314 172.122 169.642 124.978 1.00 0.00 C**

**ATOM 692 O PHE A 314 171.838 170.632 125.659 1.00 0.00 O**

**ATOM 693 N PHE A 314 173.797 168.267 123.757 1.00 0.00 N**

**ATOM 694 CB PHE A 314 173.480 170.614 123.125 1.00 0.00 C**

**ATOM 695 CG PHE A 314 174.837 170.827 122.522 1.00 0.00 C**

**ATOM 696 CD1 PHE A 314 175.967 170.864 123.325 1.00 0.00 C**

**ATOM 697 CE1 PHE A 314 177.221 171.058 122.774 1.00 0.00 C**

**ATOM 698 CZ PHE A 314 177.355 171.222 121.408 1.00 0.00 C**

**ATOM 699 CE2 PHE A 314 176.236 171.190 120.597 1.00 0.00 C**

**ATOM 700 CD2 PHE A 314 174.985 170.994 121.155 1.00 0.00 C**

**ATOM 701 CA PHE A 332 173.251 178.795 148.612 1.00 0.00 C**

**ATOM 702 C PHE A 332 172.251 179.943 148.716 1.00 0.00 C**

**ATOM 703 O PHE A 332 172.592 181.019 149.221 1.00 0.00 O**

**ATOM 704 N PHE A 332 172.574 177.507 148.559 1.00 0.00 N**

**ATOM 705 CB PHE A 332 174.156 178.944 147.386 1.00 0.00 C**

**ATOM 706 CG PHE A 332 175.035 180.167 147.408 1.00 0.00 C**

**ATOM 707 CD1 PHE A 332 174.627 181.355 146.818 1.00 0.00 C**

**ATOM 708 CE1 PHE A 332 175.443 182.473 146.843 1.00 0.00 C**

**ATOM 709 CZ PHE A 332 176.679 182.412 147.458 1.00 0.00 C**

**ATOM 710 CE2 PHE A 332 177.097 181.234 148.048 1.00 0.00 C**

**ATOM 711 CD2 PHE A 332 176.278 180.121 148.020 1.00 0.00 C**

**ATOM 712 CA PHE A 337 171.811 177.572 155.708 1.00 0.00 C**

**ATOM 713 C PHE A 337 172.948 178.588 155.716 1.00 0.00 C**

**ATOM 714 O PHE A 337 174.026 178.315 156.256 1.00 0.00 O**

**ATOM 715 N PHE A 337 170.520 178.230 155.873 1.00 0.00 N**

**ATOM 716 CB PHE A 337 171.821 176.755 154.415 1.00 0.00 C**

**ATOM 717 CG PHE A 337 170.900 175.560 154.438 1.00 0.00 C**

**ATOM 718 CD1 PHE A 337 170.487 175.000 155.639 1.00 0.00 C**

**ATOM 719 CE1 PHE A 337 169.639 173.903 155.658 1.00 0.00 C**

**ATOM 720 CZ PHE A 337 169.196 173.354 154.471 1.00 0.00 C**

**ATOM 721 CE2 PHE A 337 169.598 173.900 153.267 1.00 0.00 C**

**ATOM 722 CD2 PHE A 337 170.446 174.998 153.254 1.00 0.00 C**

**ATOM 723 CA PHE A 341 177.127 178.566 157.922 1.00 0.00 C**

**ATOM 724 C PHE A 341 178.223 179.258 158.723 1.00 0.00 C**

**ATOM 725 O PHE A 341 179.335 178.729 158.832 1.00 0.00 O**

**ATOM 726 N PHE A 341 175.804 178.815 158.484 1.00 0.00 N**

**ATOM 727 CB PHE A 341 177.171 179.021 156.463 1.00 0.00 C**

**ATOM 728 CA MET A 81 175.920 166.795 152.115 1.00 0.00 C**

**ATOM 729 C MET A 81 176.762 167.013 153.369 1.00 0.00 C**

**ATOM 730 O MET A 81 176.325 166.685 154.479 1.00 0.00 O**

**ATOM 731 N MET A 81 176.015 167.928 151.200 1.00 0.00 N**

**ATOM 732 CB MET A 81 176.335 165.510 151.402 1.00 0.00 C**

**ATOM 733 CG MET A 81 175.167 164.726 150.831 1.00 0.00 C**

**ATOM 734 SD MET A 81 173.844 164.491 152.034 1.00 0.00 S**

**ATOM 735 CE MET A 81 174.674 163.502 153.278 1.00 0.00 C**

**ATOM 736 CA MET A 90 167.085 166.213 160.471 1.00 0.00 C**

**ATOM 737 C MET A 90 165.837 166.582 161.269 1.00 0.00 C**

**ATOM 738 O MET A 90 165.057 165.722 161.673 1.00 0.00 O**

**ATOM 739 N MET A 90 168.296 166.715 161.118 1.00 0.00 N**

**ATOM 740 CB MET A 90 167.199 164.703 160.297 1.00 0.00 C**

**ATOM 741 CG MET A 90 166.432 164.136 159.121 1.00 0.00 C**

**ATOM 742 SD MET A 90 167.242 164.438 157.539 1.00 0.00 S**

**ATOM 743 CE MET A 90 168.577 163.247 157.612 1.00 0.00 C**

**ATOM 744 CA MET A 112 176.992 161.494 134.268 1.00 0.00 C**

**ATOM 745 C MET A 112 178.267 161.033 133.560 1.00 0.00 C**

**ATOM 746 O MET A 112 178.252 160.969 132.319 1.00 0.00 O**

**ATOM 747 N MET A 112 176.617 160.659 135.410 1.00 0.00 N**

**ATOM 748 CB MET A 112 177.076 162.965 134.702 1.00 0.00 C**

**ATOM 749 CG MET A 112 177.986 163.810 133.823 1.00 0.00 C**

**ATOM 750 SD MET A 112 177.676 165.580 133.892 1.00 0.00 S**

**ATOM 751 CE MET A 112 179.033 166.175 132.885 1.00 0.00 C**

**ATOM 752 CA MET A 121 183.226 155.859 121.444 1.00 0.00 C**

**ATOM 753 C MET A 121 181.969 155.026 121.154 1.00 0.00 C**

**ATOM 754 O MET A 121 182.057 153.901 120.649 1.00 0.00 O**

**ATOM 755 N MET A 121 182.873 157.181 121.977 1.00 0.00 N**

**ATOM 756 CB MET A 121 184.207 155.144 122.399 1.00 0.00 C**

**ATOM 757 CG MET A 121 184.901 153.884 121.883 1.00 0.00 C**

**ATOM 758 SD MET A 121 186.023 154.217 120.514 1.00 0.00 S**

**ATOM 759 CE MET A 121 186.088 152.606 119.734 1.00 0.00 C**

**ATOM 760 CA MET A 142 164.573 161.057 137.833 1.00 0.00 C**

**ATOM 761 C MET A 142 163.154 161.076 138.398 1.00 0.00 C**

**ATOM 762 O MET A 142 162.604 162.147 138.704 1.00 0.00 O**

**ATOM 763 N MET A 142 165.370 159.999 138.449 1.00 0.00 N**

**ATOM 764 CB MET A 142 164.573 160.884 136.312 1.00 0.00 C**

**ATOM 765 CG MET A 142 163.869 161.989 135.539 1.00 0.00 C**

**ATOM 766 SD MET A 142 162.151 161.617 135.145 1.00 0.00 S**

**ATOM 767 CE MET A 142 162.376 160.493 133.770 1.00 0.00 C**

**ATOM 768 CA MET A 151 155.596 166.791 147.255 1.00 0.00 C**

**ATOM 769 C MET A 151 155.996 167.967 148.133 1.00 0.00 C**

**ATOM 770 O MET A 151 155.226 168.379 149.007 1.00 0.00 O**

**ATOM 771 N MET A 151 156.091 166.953 145.891 1.00 0.00 N**

**ATOM 772 CB MET A 151 156.090 165.477 147.854 1.00 0.00 C**

**ATOM 773 CG MET A 151 155.207 164.287 147.518 1.00 0.00 C**

**ATOM 774 SD MET A 151 153.534 164.474 148.164 1.00 0.00 S**

**ATOM 775 CE MET A 151 152.736 163.034 147.458 1.00 0.00 C**

**ATOM 776 CA MET A 152 157.534 169.739 148.665 1.00 0.00 C**

**ATOM 777 C MET A 152 156.714 170.949 148.224 1.00 0.00 C**

**ATOM 778 O MET A 152 156.428 171.828 149.042 1.00 0.00 O**

**ATOM 779 N MET A 152 157.178 168.547 147.904 1.00 0.00 N**

**ATOM 780 CB MET A 152 159.040 170.008 148.565 1.00 0.00 C**

**ATOM 781 CG MET A 152 159.517 170.671 147.291 1.00 0.00 C**

**ATOM 782 SD MET A 152 161.226 171.222 147.428 1.00 0.00 S**

**ATOM 783 CE MET A 152 162.082 169.651 147.463 1.00 0.00 C**

**ATOM 784 CA MET A 226 154.329 152.967 128.849 1.00 0.00 C**

**ATOM 785 C MET A 226 154.900 154.379 128.766 1.00 0.00 C**

**ATOM 786 O MET A 226 154.955 155.085 129.780 1.00 0.00 O**

**ATOM 787 N MET A 226 153.518 152.644 127.684 1.00 0.00 N**

**ATOM 788 CB MET A 226 155.464 151.951 129.011 1.00 0.00 C**

**ATOM 789 CG MET A 226 156.290 152.156 130.273 1.00 0.00 C**

**ATOM 790 SD MET A 226 157.621 150.961 130.473 1.00 0.00 S**

**ATOM 791 CE MET A 226 158.404 151.599 131.951 1.00 0.00 C**

**ATOM 792 CA MET A 249 150.075 179.917 147.428 1.00 0.00 C**

**ATOM 793 C MET A 249 150.333 181.415 147.319 1.00 0.00 C**

**ATOM 794 O MET A 249 150.221 182.142 148.310 1.00 0.00 O**

**ATOM 795 N MET A 249 149.267 179.440 146.312 1.00 0.00 N**

**ATOM 796 CB MET A 249 151.394 179.147 147.482 1.00 0.00 C**

**ATOM 797 CG MET A 249 152.303 179.556 148.624 1.00 0.00 C**

**ATOM 798 SD MET A 249 153.877 178.680 148.644 1.00 0.00 S**

**ATOM 799 CE MET A 249 154.764 179.559 147.364 1.00 0.00 C**

**ATOM 800 CA GLU A 203 172.304 144.880 113.548 1.00 0.00 C**

**ATOM 801 C GLU A 203 172.882 145.720 112.417 1.00 0.00 C**

**ATOM 802 O GLU A 203 173.241 145.164 111.374 1.00 0.00 O**

**ATOM 803 N GLU A 203 172.065 145.644 114.764 1.00 0.00 N**

**ATOM 804 CB GLU A 203 171.005 144.215 113.081 1.00 0.00 C**

**ATOM 805 CA GLU A 209 173.882 148.662 122.628 1.00 0.00 C**

**ATOM 806 C GLU A 209 172.743 148.394 123.608 1.00 0.00 C**

**ATOM 807 O GLU A 209 171.622 148.079 123.200 1.00 0.00 O**

**ATOM 808 N GLU A 209 174.415 147.361 122.217 1.00 0.00 N**

**ATOM 809 CB GLU A 209 173.408 149.466 121.425 1.00 0.00 C**

**ATOM 810 CG GLU A 209 174.532 149.921 120.518 1.00 0.00 C**

**ATOM 811 CD GLU A 209 175.433 150.940 121.170 1.00 0.00 C**

**ATOM 812 OE1 GLU A 209 174.929 151.798 121.924 1.00 0.00 O**

**ATOM 813 OE2 GLU A 209 176.657 150.860 120.946 1.00 0.00 O**

**ATOM 814 CA GLU A 264 160.012 196.130 152.961 1.00 0.00 C**

**ATOM 815 C GLU A 264 159.307 194.792 152.776 1.00 0.00 C**

**ATOM 816 O GLU A 264 159.870 193.873 152.172 1.00 0.00 O**

**ATOM 817 N GLU A 264 159.075 197.247 152.968 1.00 0.00 N**

**ATOM 818 CB GLU A 264 160.828 196.111 154.255 1.00 0.00 C**

**ATOM 819 CG GLU A 264 161.837 197.241 154.377 1.00 0.00 C**

**ATOM 820 CD GLU A 264 163.037 197.050 153.472 1.00 0.00 C**

**ATOM 821 OE1 GLU A 264 163.474 195.892 153.302 1.00 0.00 O**

**ATOM 822 OE2 GLU A 264 163.543 198.055 152.931 1.00 0.00 O**

**ATOM 823 CA GLU A 297 161.185 160.588 118.465 1.00 0.00 C**

**ATOM 824 C GLU A 297 160.293 159.361 118.634 1.00 0.00 C**

**ATOM 825 O GLU A 297 160.395 158.395 117.869 1.00 0.00 O**

**ATOM 826 N GLU A 297 160.522 161.801 118.945 1.00 0.00 N**

**ATOM 827 CB GLU A 297 162.513 160.392 119.196 1.00 0.00 C**

**ATOM 828 CA GLU A 335 169.387 181.756 153.892 1.00 0.00 C**

**ATOM 829 C GLU A 335 169.456 181.267 155.333 1.00 0.00 C**

**ATOM 830 O GLU A 335 170.252 181.810 156.105 1.00 0.00 O**

**ATOM 831 N GLU A 335 168.572 180.914 153.023 1.00 0.00 N**

**ATOM 832 CB GLU A 335 168.885 183.203 153.859 1.00 0.00 C**

**ATOM 833 CG GLU A 335 167.664 183.479 154.712 1.00 0.00 C**

**ATOM 834 CD GLU A 335 166.391 183.530 153.897 1.00 0.00 C**

**ATOM 835 OE1 GLU A 335 166.384 182.996 152.768 1.00 0.00 O**

**ATOM 836 OE2 GLU A 335 165.399 184.111 154.381 1.00 0.00 O**

**ATOM 837 CA ARG A 86 177.800 168.716 160.255 1.00 0.00 C**

**ATOM 838 C ARG A 86 177.486 170.196 160.464 1.00 0.00 C**

**ATOM 839 O ARG A 86 178.294 170.913 161.063 1.00 0.00 O**

**ATOM 840 N ARG A 86 177.061 168.124 159.144 1.00 0.00 N**

**ATOM 841 CB ARG A 86 179.304 168.524 160.050 1.00 0.00 C**

**ATOM 842 CA ARG A 156 154.362 174.626 150.687 1.00 0.00 C**

**ATOM 843 C ARG A 156 152.934 175.147 150.556 1.00 0.00 C**

**ATOM 844 O ARG A 156 152.541 176.085 151.264 1.00 0.00 O**

**ATOM 845 N ARG A 156 154.407 173.166 150.627 1.00 0.00 N**

**ATOM 846 CB ARG A 156 155.249 175.223 149.597 1.00 0.00 C**

**ATOM 847 CG ARG A 156 156.725 174.944 149.781 1.00 0.00 C**

**ATOM 848 CD ARG A 156 157.317 175.836 150.843 1.00 0.00 C**

**ATOM 849 NE ARG A 156 156.905 177.220 150.655 1.00 0.00 N**

**ATOM 850 CZ ARG A 156 157.669 178.151 150.104 1.00 0.00 C**

**ATOM 851 NH2 ARG A 156 157.205 179.393 149.997 1.00 0.00 N**

**ATOM 852 NH1 ARG A 156 158.879 177.869 149.650 1.00 0.00 N**

**ATOM 853 CA ARG A 170 153.705 168.848 158.653 1.00 0.00 C**

**ATOM 854 C ARG A 170 154.597 168.349 159.784 1.00 0.00 C**

**ATOM 855 O ARG A 170 155.657 168.931 160.040 1.00 0.00 O**

**ATOM 856 N ARG A 170 152.288 168.630 158.918 1.00 0.00 N**

**ATOM 857 CB ARG A 170 153.975 170.326 158.376 1.00 0.00 C**

**ATOM 858 CG ARG A 170 153.329 170.839 157.103 1.00 0.00 C**

**ATOM 859 CD ARG A 170 153.788 172.249 156.779 1.00 0.00 C**

**ATOM 860 NE ARG A 170 155.118 172.272 156.181 1.00 0.00 N**

**ATOM 861 CZ ARG A 170 156.186 172.818 156.745 1.00 0.00 C**

**ATOM 862 NH2 ARG A 170 157.348 172.787 156.100 1.00 0.00 N**

**ATOM 863 NH1 ARG A 170 156.122 173.395 157.933 1.00 0.00 N**

**ATOM 864 CA ARG A 202 172.515 146.503 116.979 1.00 0.00 C**

**ATOM 865 C ARG A 202 172.946 145.695 115.760 1.00 0.00 C**

**ATOM 866 O ARG A 202 174.040 145.126 115.717 1.00 0.00 O**

**ATOM 867 N ARG A 202 172.999 145.884 118.204 1.00 0.00 N**

**ATOM 868 CB ARG A 202 173.024 147.940 116.863 1.00 0.00 C**

**ATOM 869 CG ARG A 202 172.050 148.985 117.382 1.00 0.00 C**

**ATOM 870 CD ARG A 202 172.653 150.378 117.333 1.00 0.00 C**

**ATOM 871 NE ARG A 202 172.086 151.260 118.347 1.00 0.00 N**

**ATOM 872 CZ ARG A 202 172.661 152.376 118.774 1.00 0.00 C**

**ATOM 873 NH2 ARG A 202 172.051 153.105 119.704 1.00 0.00 N**

**ATOM 874 NH1 ARG A 202 173.826 152.781 118.296 1.00 0.00 N**

**ATOM 875 CA ARG A 252 146.546 183.425 149.354 1.00 0.00 C**

**ATOM 876 C ARG A 252 147.657 184.011 150.213 1.00 0.00 C**

**ATOM 877 O ARG A 252 147.450 184.216 151.413 1.00 0.00 O**

**ATOM 878 N ARG A 252 146.908 183.333 147.945 1.00 0.00 N**

**ATOM 879 CB ARG A 252 146.128 182.051 149.885 1.00 0.00 C**

**ATOM 880 CG ARG A 252 144.894 181.480 149.189 1.00 0.00 C**

**ATOM 881 CD ARG A 252 143.812 182.540 148.992 1.00 0.00 C**

**ATOM 882 NE ARG A 252 143.708 182.962 147.600 1.00 0.00 N**

**ATOM 883 CZ ARG A 252 142.965 183.976 147.177 1.00 0.00 C**

**ATOM 884 NH2 ARG A 252 142.949 184.271 145.880 1.00 0.00 N**

**ATOM 885 NH1 ARG A 252 142.244 184.702 148.015 1.00 0.00 N**

**ATOM 886 CA ARG A 257 146.671 191.166 155.988 1.00 0.00 C**

**ATOM 887 C ARG A 257 148.174 191.339 156.172 1.00 0.00 C**

**ATOM 888 O ARG A 257 148.754 192.328 155.710 1.00 0.00 O**

**ATOM 889 N ARG A 257 146.329 189.791 155.628 1.00 0.00 N**

**ATOM 890 CB ARG A 257 145.924 191.572 157.259 1.00 0.00 C**

**ATOM 891 CG ARG A 257 146.379 192.890 157.859 1.00 0.00 C**

**ATOM 892 CD ARG A 257 145.502 193.293 159.026 1.00 0.00 C**

**ATOM 893 NE ARG A 257 145.829 194.625 159.518 1.00 0.00 N**

**ATOM 894 CZ ARG A 257 145.006 195.382 160.229 1.00 0.00 C**

**ATOM 895 NH2 ARG A 257 145.415 196.580 160.636 1.00 0.00 N**

**ATOM 896 NH1 ARG A 257 143.788 194.972 160.542 1.00 0.00 N**

**ATOM 897 CA ARG A 263 158.257 199.458 152.277 1.00 0.00 C**

**ATOM 898 C ARG A 263 159.255 198.310 152.183 1.00 0.00 C**

**ATOM 899 O ARG A 263 160.198 198.393 151.389 1.00 0.00 O**

**ATOM 900 N ARG A 263 156.982 199.046 152.851 1.00 0.00 N**

**ATOM 901 CB ARG A 263 158.856 200.599 153.100 1.00 0.00 C**

**ATOM 902 CA ARG A 267 159.920 193.815 148.388 1.00 0.00 C**

**ATOM 903 C ARG A 267 160.355 192.393 148.716 1.00 0.00 C**

**ATOM 904 O ARG A 267 160.789 191.649 147.827 1.00 0.00 O**

**ATOM 905 N ARG A 267 158.677 194.166 149.073 1.00 0.00 N**

**ATOM 906 CB ARG A 267 161.022 194.806 148.758 1.00 0.00 C**

**ATOM 907 CG ARG A 267 162.204 194.806 147.803 1.00 0.00 C**

**ATOM 908 CD ARG A 267 163.415 194.115 148.414 1.00 0.00 C**

**ATOM 909 NE ARG A 267 163.779 194.679 149.709 1.00 0.00 N**

**ATOM 910 CZ ARG A 267 164.623 194.114 150.561 1.00 0.00 C**

**ATOM 911 NH2 ARG A 267 164.880 194.718 151.718 1.00 0.00 N**

**ATOM 912 NH1 ARG A 267 165.215 192.963 150.287 1.00 0.00 N**

**ATOM 913 CA ARG A 270 158.440 189.990 145.468 1.00 0.00 C**

**ATOM 914 C ARG A 270 159.688 189.144 145.257 1.00 0.00 C**

**ATOM 915 O ARG A 270 159.781 188.410 144.269 1.00 0.00 O**

**ATOM 916 N ARG A 270 158.062 190.032 146.877 1.00 0.00 N**

**ATOM 917 CB ARG A 270 158.659 191.399 144.915 1.00 0.00 C**

**ATOM 918 CG ARG A 270 157.400 192.231 144.758 1.00 0.00 C**

**ATOM 919 CD ARG A 270 157.736 193.605 144.204 1.00 0.00 C**

**ATOM 920 NE ARG A 270 158.511 194.401 145.148 1.00 0.00 N**

**ATOM 921 CZ ARG A 270 159.074 195.566 144.860 1.00 0.00 C**

**ATOM 922 NH2 ARG A 270 159.748 196.212 145.807 1.00 0.00 N**

**ATOM 923 NH1 ARG A 270 158.982 196.099 143.653 1.00 0.00 N**

**ATOM 924 CA ARG A 271 161.841 188.378 146.074 1.00 0.00 C**

**ATOM 925 C ARG A 271 161.481 186.905 146.246 1.00 0.00 C**

**ATOM 926 O ARG A 271 162.030 186.043 145.550 1.00 0.00 O**

**ATOM 927 N ARG A 271 160.658 189.232 146.173 1.00 0.00 N**

**ATOM 928 CB ARG A 271 162.891 188.799 147.101 1.00 0.00 C**

**ATOM 929 CG ARG A 271 163.503 190.159 146.824 1.00 0.00 C**

**ATOM 930 CD ARG A 271 164.224 190.175 145.488 1.00 0.00 C**

**ATOM 931 NE ARG A 271 164.369 191.527 144.963 1.00 0.00 N**

**ATOM 932 CZ ARG A 271 163.541 192.082 144.088 1.00 0.00 C**

**ATOM 933 NH2 ARG A 271 163.770 193.327 143.677 1.00 0.00 N**

**ATOM 934 NH1 ARG A 271 162.493 191.427 143.615 1.00 0.00 N**

**ATOM 935 CA ARG A 274 160.781 185.684 141.946 1.00 0.00 C**

**ATOM 936 C ARG A 274 161.631 184.437 142.150 1.00 0.00 C**

**ATOM 937 O ARG A 274 161.990 183.765 141.178 1.00 0.00 O**

**ATOM 938 N ARG A 274 159.796 185.835 143.011 1.00 0.00 N**

**ATOM 939 CB ARG A 274 161.662 186.930 141.856 1.00 0.00 C**

**ATOM 940 CG ARG A 274 160.957 188.142 141.262 1.00 0.00 C**

**ATOM 941 CD ARG A 274 160.307 187.818 139.924 1.00 0.00 C**

**ATOM 942 NE ARG A 274 160.133 189.009 139.101 1.00 0.00 N**

**ATOM 943 CZ ARG A 274 159.092 189.826 139.179 1.00 0.00 C**

**ATOM 944 NH2 ARG A 274 159.040 190.888 138.380 1.00 0.00 N**

**ATOM 945 NH1 ARG A 274 158.104 189.609 140.031 1.00 0.00 N**

**ATOM 946 CA ARG A 339 172.897 181.916 158.747 1.00 0.00 C**

**ATOM 947 C ARG A 339 173.633 180.918 159.631 1.00 0.00 C**

**ATOM 948 O ARG A 339 174.437 181.313 160.484 1.00 0.00 O**

**ATOM 949 N ARG A 339 172.812 181.447 157.368 1.00 0.00 N**

**ATOM 950 CB ARG A 339 171.495 182.186 159.294 1.00 0.00 C**

**ATOM 951 CA ARG A 342 178.916 181.157 160.078 1.00 0.00 C**

**ATOM 952 C ARG A 342 179.052 180.546 161.469 1.00 0.00 C**

**ATOM 953 O ARG A 342 180.133 180.107 161.861 1.00 0.00 O**

**ATOM 954 N ARG A 342 177.935 180.428 159.283 1.00 0.00 N**

**ATOM 955 CB ARG A 342 178.530 182.634 160.186 1.00 0.00 C**

**ATOM 956 C N.A X -1 170.109 153.042 123.099 1.00 0.00 C**

**ATOM 957 C N.A X -1 169.508 152.139 122.011 1.00 0.00 C**

**ATOM 958 N N.A X -1 170.217 150.894 121.816 1.00 0.00 N**

**ATOM 959 C N.A X -1 170.068 150.113 120.718 1.00 0.00 C**

**ATOM 960 N N.A X -1 169.915 150.566 119.514 1.00 0.00 N**

**ATOM 961 N N.A X -1 170.106 148.795 121.009 1.00 0.00 N**

**ATOM 962 C N.A X -1 171.336 152.533 123.863 1.00 0.00 C**

**ATOM 963 C N.A X -1 171.887 153.468 124.972 1.00 0.00 C**

**ATOM 964 N N.A X -1 170.853 154.142 125.733 1.00 0.00 N**

**ATOM 965 C N.A X -1 171.139 154.920 126.840 1.00 0.00 C**

**ATOM 966 O N.A X -1 172.273 155.090 127.285 1.00 0.00 O**

**ATOM 967 C N.A X -1 169.921 155.757 127.275 1.00 0.00 C**

**ATOM 968 N N.A X -1 168.706 154.946 127.417 1.00 0.00 N**

**ATOM 969 C N.A X -1 167.551 154.941 126.681 1.00 0.00 C**

**ATOM 970 O N.A X -1 166.640 154.176 127.013 1.00 0.00 O**

**ATOM 971 C N.A X -1 167.454 155.855 125.441 1.00 0.00 C**

**ATOM 972 N N.A X -1 166.250 155.476 124.718 1.00 0.00 N**

**ATOM 973 C N.A X -1 166.155 154.399 123.858 1.00 0.00 C**

**ATOM 974 O N.A X -1 167.087 153.670 123.535 1.00 0.00 O**

**ATOM 975 C N.A X -1 164.725 154.202 123.348 1.00 0.00 C**

**ATOM 976 N N.A X -1 163.972 154.184 124.607 1.00 0.00 N**

**ATOM 977 C N.A X -1 162.627 154.397 124.834 1.00 0.00 C**

**ATOM 978 O N.A X -1 161.829 154.786 123.986 1.00 0.00 O**

**ATOM 979 C N.A X -1 162.283 154.117 126.324 1.00 0.00 C**

**ATOM 980 N N.A X -1 163.113 154.977 127.150 1.00 0.00 N**

**ATOM 981 C N.A X -1 163.079 156.347 126.977 1.00 0.00 C**

**ATOM 982 O N.A X -1 163.076 156.909 125.882 1.00 0.00 O**

**ATOM 983 C N.A X -1 162.871 157.069 128.296 1.00 0.00 C**

**ATOM 984 N N.A X -1 163.901 158.057 128.492 1.00 0.00 N**

**ATOM 985 C N.A X -1 165.159 157.659 128.893 1.00 0.00 C**

**ATOM 986 O N.A X -1 165.484 156.476 128.983 1.00 0.00 O**

**ATOM 987 C N.A X -1 166.064 158.772 129.407 1.00 0.00 C**

**ATOM 988 N N.A X -1 165.753 160.088 128.859 1.00 0.00 N**

**ATOM 989 C N.A X -1 166.163 160.405 127.583 1.00 0.00 C**

**ATOM 990 O N.A X -1 165.675 159.824 126.612 1.00 0.00 O**

**ATOM 991 C N.A X -1 167.197 161.516 127.392 1.00 0.00 C**

**ATOM 992 N N.A X -1 167.996 161.688 128.585 1.00 0.00 N**

**ATOM 993 C N.A X -1 169.003 160.783 128.874 1.00 0.00 C**

**ATOM 994 O N.A X -1 169.511 160.064 128.014 1.00 0.00 O**

**ATOM 995 C N.A X -1 169.352 160.721 130.375 1.00 0.00 C**

**ATOM 996 N N.A X -1 169.999 159.436 130.640 1.00 0.00 N**

**ATOM 997 C N.A X -1 170.216 161.933 130.749 1.00 0.00 C**

**ATOM 998 C N.A X -1 169.913 162.595 132.070 1.00 0.00 C**

**ATOM 999 C N.A X -1 170.449 163.869 132.286 1.00 0.00 C**

**ATOM 1000 C N.A X -1 170.212 164.543 133.479 1.00 0.00 C**

**ATOM 1001 C N.A X -1 169.400 163.986 134.446 1.00 0.00 C**

**ATOM 1002 O N.A X -1 169.080 164.673 135.581 1.00 0.00 O**

**ATOM 1003 C N.A X -1 168.906 162.700 134.278 1.00 0.00 C**

**ATOM 1004 C N.A X -1 169.176 161.999 133.100 1.00 0.00 C**

**ATOM 1005 C N.A X -1 165.243 160.993 129.876 1.00 0.00 C**

**ATOM 1006 C N.A X -1 164.889 160.088 131.043 1.00 0.00 C**

**ATOM 1007 C N.A X -1 165.886 158.941 130.918 1.00 0.00 C**

**ATOM 1008 C N.A X -1 161.443 157.617 128.211 1.00 0.00 C**

**ATOM 1009 C N.A X -1 160.874 158.296 129.410 1.00 0.00 C**

**ATOM 1010 C N.A X -1 161.261 157.911 130.682 1.00 0.00 C**

**ATOM 1011 C N.A X -1 160.689 158.526 131.774 1.00 0.00 C**

**ATOM 1012 C N.A X -1 159.745 159.545 131.627 1.00 0.00 C**

**ATOM 1013 C N.A X -1 159.326 159.911 130.361 1.00 0.00 C**

**ATOM 1014 C N.A X -1 159.866 159.263 129.258 1.00 0.00 C**

**ATOM 1015 C N.A X -1 160.803 154.205 126.712 1.00 0.00 C**

**ATOM 1016 C N.A X -1 160.520 153.987 128.224 1.00 0.00 C**

**ATOM 1017 C N.A X -1 160.854 152.587 128.722 1.00 0.00 C**

**ATOM 1018 C N.A X -1 159.061 154.287 128.552 1.00 0.00 C**

**ATOM 1019 C N.A X -1 164.359 155.329 122.378 1.00 0.00 C**

**ATOM 1020 C N.A X -1 163.410 154.851 121.272 1.00 0.00 C**

**ATOM 1021 C N.A X -1 162.514 155.990 120.781 1.00 0.00 C**

**ATOM 1022 N N.A X -1 161.326 155.511 120.123 1.00 0.00 N**

**ATOM 1023 C N.A X -1 160.352 154.691 120.580 1.00 0.00 C**

**ATOM 1024 N N.A X -1 159.460 154.224 119.765 1.00 0.00 N**

**ATOM 1025 N N.A X -1 160.309 154.368 121.885 1.00 0.00 N**

**ATOM 1026 C N.A X -1 167.365 157.338 125.818 1.00 0.00 C**

**ATOM 1027 C N.A X -1 167.627 158.292 124.641 1.00 0.00 C**

**ATOM 1028 C N.A X -1 166.463 158.343 123.658 1.00 0.00 C**

**ATOM 1029 N N.A X -1 166.636 159.387 122.672 1.00 0.00 N**

**ATOM 1030 C N.A X -1 165.720 159.715 121.726 1.00 0.00 C**

**ATOM 1031 N N.A X -1 164.526 159.236 121.622 1.00 0.00 N**

**ATOM 1032 N N.A X -1 166.159 160.637 120.850 1.00 0.00 N**

**ATOM 1033 C N.A X -1 170.202 156.619 128.508 1.00 0.00 C**

**ATOM 1034 C N.A X -1 170.333 155.820 129.799 1.00 0.00 C**

**ATOM 1035 C N.A X -1 171.593 156.113 130.610 1.00 0.00 C**

**ATOM 1036 N N.A X -1 172.741 155.513 129.953 1.00 0.00 N**

**ATOM 1037 C N.A X -1 173.935 155.312 130.559 1.00 0.00 C**

**ATOM 1038 N N.A X -1 174.244 155.725 131.739 1.00 0.00 N**

**ATOM 1039 N N.A X -1 174.788 154.606 129.790 1.00 0.00 N**

**ATOM 1040 C N.A X -1 172.940 154.367 124.299 1.00 0.00 C**

**ATOM 1041 O N.A X -1 174.080 153.911 124.174 1.00 0.00 O**

**ATOM 1042 N N.A X -1 172.553 155.490 123.581 1.00 0.00 N**

**ATOM 1043 C N.A X -1 172.709 155.428 122.138 1.00 0.00 C**

**ATOM 1044 C N.A X -1 174.033 155.346 121.361 1.00 0.00 C**

**ATOM 1045 O N.A X -1 173.986 154.864 120.213 1.00 0.00 O**

**ATOM 1046 N N.A X -1 175.205 155.872 121.795 1.00 0.00 N**

**ATOM 1047 C N.A X -1 175.454 157.168 122.436 1.00 0.00 C**

**ATOM 1048 C N.A X -1 174.973 157.242 123.878 1.00 0.00 C**

**ATOM 1049 O N.A X -1 174.332 158.134 124.420 1.00 0.00 O**

**ATOM 1050 O N.A X -1 175.386 156.182 124.585 1.00 0.00 O**

**ATOM 1051 C N.A X -1 174.981 158.332 121.565 1.00 0.00 C**

**ATOM 1052 C N.A X -1 175.684 158.358 120.206 1.00 0.00 C**

**ATOM 1053 C N.A X -1 174.984 159.243 119.178 1.00 0.00 C**

**ATOM 1054 C N.A X -1 173.588 158.778 118.746 1.00 0.00 C**

**ATOM 1055 N N.A X -1 173.572 157.439 118.168 1.00 0.00 N**

**ATOM 1056 C N.A X -1 171.751 156.485 121.574 1.00 0.00 C**

**ATOM 1057 C N.A X -1 171.441 157.388 122.758 1.00 0.00 C**

**ATOM 1058 C N.A X -1 171.503 156.431 123.933 1.00 0.00 C**

**ATOM 1059 CA ALA A 57 182.842 171.465 117.380 1.00 0.00 C**

**ATOM 1060 C ALA A 57 182.513 170.372 118.388 1.00 0.00 C**

**ATOM 1061 O ALA A 57 182.454 170.619 119.591 1.00 0.00 O**

**ATOM 1062 N ALA A 57 182.886 170.925 116.026 1.00 0.00 N**

**ATOM 1063 CB ALA A 57 181.827 172.591 117.468 1.00 0.00 C**

**ATOM 1064 CA ALA A 64 185.066 170.319 128.448 1.00 0.00 C**

**ATOM 1065 C ALA A 64 183.813 170.188 129.308 1.00 0.00 C**

**ATOM 1066 O ALA A 64 183.830 170.534 130.497 1.00 0.00 O**

**ATOM 1067 N ALA A 64 185.316 169.106 127.675 1.00 0.00 N**

**ATOM 1068 CB ALA A 64 184.956 171.522 127.512 1.00 0.00 C**

**ATOM 1069 CA ALA A 93 159.882 168.613 157.450 1.00 0.00 C**

**ATOM 1070 C ALA A 93 160.460 168.872 156.066 1.00 0.00 C**

**ATOM 1071 O ALA A 93 160.256 168.054 155.163 1.00 0.00 O**

**ATOM 1072 N ALA A 93 160.482 169.450 158.479 1.00 0.00 N**

**ATOM 1073 CB ALA A 93 158.368 168.823 157.420 1.00 0.00 C**

**ATOM 1074 CA ALA A 102 170.890 166.433 147.395 1.00 0.00 C**

**ATOM 1075 C ALA A 102 171.446 165.058 147.047 1.00 0.00 C**

**ATOM 1076 O ALA A 102 172.347 164.943 146.207 1.00 0.00 O**

**ATOM 1077 N ALA A 102 169.436 166.405 147.514 1.00 0.00 N**

**ATOM 1078 CB ALA A 102 171.510 166.948 148.692 1.00 0.00 C**

**ATOM 1079 CA ALA A 104 169.366 162.410 144.093 1.00 0.00 C**

**ATOM 1080 C ALA A 104 170.268 163.110 143.083 1.00 0.00 C**

**ATOM 1081 O ALA A 104 170.667 162.514 142.069 1.00 0.00 O**

**ATOM 1082 N ALA A 104 169.791 162.679 145.463 1.00 0.00 N**

**ATOM 1083 CB ALA A 104 167.915 162.851 143.917 1.00 0.00 C**

**ATOM 1084 CA ALA A 106 174.673 163.360 143.708 1.00 0.00 C**

**ATOM 1085 C ALA A 106 174.693 162.076 142.888 1.00 0.00 C**

**ATOM 1086 O ALA A 106 175.667 161.801 142.178 1.00 0.00 O**

**ATOM 1087 N ALA A 106 173.372 164.020 143.638 1.00 0.00 N**

**ATOM 1088 CB ALA A 106 175.035 163.070 145.163 1.00 0.00 C**

**ATOM 1089 CA ALA A 159 151.677 175.284 154.760 1.00 0.00 C**

**ATOM 1090 C ALA A 159 151.159 176.714 154.717 1.00 0.00 C**

**ATOM 1091 O ALA A 159 150.800 177.275 155.757 1.00 0.00 O**

**ATOM 1092 N ALA A 159 151.140 174.482 153.670 1.00 0.00 N**

**ATOM 1093 CB ALA A 159 153.204 175.277 154.714 1.00 0.00 C**

**ATOM 1094 CA ALA A 166 149.484 172.350 158.465 1.00 0.00 C**

**ATOM 1095 C ALA A 166 150.091 171.865 159.772 1.00 0.00 C**

**ATOM 1096 O ALA A 166 151.101 171.151 159.754 1.00 0.00 O**

**ATOM 1097 N ALA A 166 148.027 172.417 158.530 1.00 0.00 N**

**ATOM 1098 CB ALA A 166 150.058 173.718 158.096 1.00 0.00 C**

**ATOM 1099 CA ALA A 175 156.823 164.076 156.165 1.00 0.00 C**

**ATOM 1100 C ALA A 175 157.686 162.902 155.717 1.00 0.00 C**

**ATOM 1101 O ALA A 175 157.785 162.609 154.517 1.00 0.00 O**

**ATOM 1102 N ALA A 175 155.957 163.700 157.278 1.00 0.00 N**

**ATOM 1103 CB ALA A 175 157.693 165.266 156.563 1.00 0.00 C**

**ATOM 1104 CA ALA A 193 162.366 148.656 134.729 1.00 0.00 C**

**ATOM 1105 C ALA A 193 163.446 147.579 134.723 1.00 0.00 C**

**ATOM 1106 O ALA A 193 163.903 147.157 133.655 1.00 0.00 O**

**ATOM 1107 N ALA A 193 161.338 148.383 135.728 1.00 0.00 N**

**ATOM 1108 CB ALA A 193 162.987 150.026 134.983 1.00 0.00 C**

**ATOM 1109 CA ALA A 234 156.230 162.905 135.050 1.00 0.00 C**

**ATOM 1110 C ALA A 234 156.258 164.349 134.571 1.00 0.00 C**

**ATOM 1111 O ALA A 234 157.110 165.114 135.037 1.00 0.00 O**

**ATOM 1112 N ALA A 234 155.013 162.188 134.713 1.00 0.00 N**

**ATOM 1113 CB ALA A 234 157.456 162.172 134.483 1.00 0.00 C**

**ATOM 1114 CA ALA A 281 161.429 178.245 135.325 1.00 0.00 C**

**ATOM 1115 C ALA A 281 162.593 177.325 134.981 1.00 0.00 C**

**ATOM 1116 O ALA A 281 162.651 176.783 133.873 1.00 0.00 O**

**ATOM 1117 N ALA A 281 160.905 177.979 136.660 1.00 0.00 N**

**ATOM 1118 CB ALA A 281 161.851 179.710 135.212 1.00 0.00 C**

**ATOM 1119 CA ALA A 298 158.378 158.382 119.837 1.00 0.00 C**

**ATOM 1120 C ALA A 298 157.166 158.688 118.966 1.00 0.00 C**

**ATOM 1121 O ALA A 298 157.288 159.399 117.962 1.00 0.00 O**

**ATOM 1122 N ALA A 298 159.409 159.388 119.629 1.00 0.00 N**

**ATOM 1123 CB ALA A 298 157.981 158.311 121.313 1.00 0.00 C**

**ATOM 1124 CA ALA A 307 171.626 167.247 114.037 1.00 0.00 C**

**ATOM 1125 C ALA A 307 171.285 166.539 115.344 1.00 0.00 C**

**ATOM 1126 O ALA A 307 171.502 167.090 116.426 1.00 0.00 O**

**ATOM 1127 N ALA A 307 172.057 166.306 113.013 1.00 0.00 N**

**ATOM 1128 CB ALA A 307 170.423 168.054 113.540 1.00 0.00 C**

**ATOM 1129 CA ALA A 308 170.292 164.596 116.434 1.00 0.00 C**

**ATOM 1130 C ALA A 308 171.409 164.013 117.290 1.00 0.00 C**

**ATOM 1131 O ALA A 308 171.126 163.532 118.392 1.00 0.00 O**

**ATOM 1132 N ALA A 308 170.770 165.312 115.258 1.00 0.00 N**

**ATOM 1133 CB ALA A 308 169.337 163.477 116.013 1.00 0.00 C**

**ATOM 1134 CA ALA A 317 173.671 168.756 129.174 1.00 0.00 C**

**ATOM 1135 C ALA A 317 172.794 169.720 129.958 1.00 0.00 C**

**ATOM 1136 O ALA A 317 173.210 170.202 131.013 1.00 0.00 O**

**ATOM 1137 N ALA A 317 172.872 167.812 128.407 1.00 0.00 N**

**ATOM 1138 CB ALA A 317 174.612 169.526 128.248 1.00 0.00 C**

**ATOM 1139 CA ALA A 331 171.496 175.563 149.603 1.00 0.00 C**

**ATOM 1140 C ALA A 331 172.060 176.977 149.675 1.00 0.00 C**

**ATOM 1141 O ALA A 331 172.040 177.599 150.744 1.00 0.00 O**

**ATOM 1142 N ALA A 331 170.732 175.346 148.382 1.00 0.00 N**

**ATOM 1143 CB ALA A 331 172.619 174.534 149.709 1.00 0.00 C**

**ATOM 1144 CA GLN A 115 176.698 158.234 130.229 1.00 0.00 C**

**ATOM 1145 C GLN A 115 177.647 158.943 129.265 1.00 0.00 C**

**ATOM 1146 O GLN A 115 177.493 158.822 128.046 1.00 0.00 O**

**ATOM 1147 N GLN A 115 177.333 157.964 131.517 1.00 0.00 N**

**ATOM 1148 CB GLN A 115 175.420 159.049 130.426 1.00 0.00 C**

**ATOM 1149 CG GLN A 115 174.439 158.930 129.269 1.00 0.00 C**

**ATOM 1150 CD GLN A 115 173.034 159.351 129.647 1.00 0.00 C**

**ATOM 1151 OE1 GLN A 115 172.763 159.687 130.799 1.00 0.00 O**

**ATOM 1152 NE2 GLN A 115 172.130 159.331 128.675 1.00 0.00 N**

**ATOM 1153 CA GLN A 213 164.279 145.167 126.320 1.00 0.00 C**

**ATOM 1154 C GLN A 213 162.780 144.932 126.157 1.00 0.00 C**

**ATOM 1155 O GLN A 213 162.139 144.255 126.960 1.00 0.00 O**

**ATOM 1156 N GLN A 213 164.462 146.444 127.003 1.00 0.00 N**

**ATOM 1157 CB GLN A 213 164.956 144.034 127.096 1.00 0.00 C**

**ATOM 1158 CG GLN A 213 165.116 142.746 126.307 1.00 0.00 C**

**ATOM 1159 CD GLN A 213 164.039 141.730 126.623 1.00 0.00 C**

**ATOM 1160 OE1 GLN A 213 163.547 141.661 127.748 1.00 0.00 O**

**ATOM 1161 NE2 GLN A 213 163.670 140.930 125.629 1.00 0.00 N**

**ATOM 1162 CA PRO A 59 185.134 166.203 119.832 1.00 0.00 C**

**ATOM 1163 C PRO A 59 185.958 166.985 120.844 1.00 0.00 C**

**ATOM 1164 O PRO A 59 186.620 166.360 121.684 1.00 0.00 O**

**ATOM 1165 N PRO A 59 184.225 167.017 119.009 1.00 0.00 N**

**ATOM 1166 CD PRO A 59 184.869 167.346 117.716 1.00 0.00 C**

**ATOM 1167 CG PRO A 59 186.147 166.543 117.722 1.00 0.00 C**

**ATOM 1168 CB PRO A 59 186.039 165.540 118.791 1.00 0.00 C**

**ATOM 1169 CA PRO A 113 180.511 160.125 133.489 1.00 0.00 C**

**ATOM 1170 C PRO A 113 180.194 158.758 132.914 1.00 0.00 C**

**ATOM 1171 O PRO A 113 180.775 158.387 131.887 1.00 0.00 O**

**ATOM 1172 N PRO A 113 179.388 160.718 134.238 1.00 0.00 N**

**ATOM 1173 CD PRO A 113 179.785 160.945 135.642 1.00 0.00 C**

**ATOM 1174 CG PRO A 113 180.943 160.021 135.822 1.00 0.00 C**

**ATOM 1175 CB PRO A 113 181.637 160.055 134.529 1.00 0.00 C**

**ATOM 1176 CA PRO A 125 182.929 148.392 127.731 1.00 0.00 C**

**ATOM 1177 C PRO A 125 182.746 148.115 129.215 1.00 0.00 C**

**ATOM 1178 O PRO A 125 183.734 148.034 129.952 1.00 0.00 O**

**ATOM 1179 N PRO A 125 182.133 149.543 127.288 1.00 0.00 N**

**ATOM 1180 CD PRO A 125 182.928 150.781 127.216 1.00 0.00 C**

**ATOM 1181 CG PRO A 125 184.340 150.314 127.467 1.00 0.00 C**

**ATOM 1182 CB PRO A 125 184.367 148.817 127.418 1.00 0.00 C**

**ATOM 1183 CA PRO A 163 146.689 177.295 158.234 1.00 0.00 C**

**ATOM 1184 C PRO A 163 146.296 176.254 159.271 1.00 0.00 C**

**ATOM 1185 O PRO A 163 147.028 176.024 160.238 1.00 0.00 O**

**ATOM 1186 N PRO A 163 146.116 177.048 156.905 1.00 0.00 N**

**ATOM 1187 CD PRO A 163 144.936 177.889 156.636 1.00 0.00 C**

**ATOM 1188 CG PRO A 163 144.805 178.721 157.883 1.00 0.00 C**

**ATOM 1189 CB PRO A 163 146.125 178.672 158.593 1.00 0.00 C**

**ATOM 1190 CA PRO A 172 158.438 164.986 160.733 1.00 0.00 C**

**ATOM 1191 C PRO A 172 158.066 163.545 160.439 1.00 0.00 C**

**ATOM 1192 O PRO A 172 158.531 162.998 159.432 1.00 0.00 O**

**ATOM 1193 N PRO A 172 157.354 165.763 161.359 1.00 0.00 N**

**ATOM 1194 CD PRO A 172 157.820 166.470 162.565 1.00 0.00 C**

**ATOM 1195 CG PRO A 172 159.317 166.353 162.473 1.00 0.00 C**

**ATOM 1196 CB PRO A 172 159.571 165.078 161.762 1.00 0.00 C**

**ATOM 1197 CA PRO A 215 160.455 142.909 121.810 1.00 0.00 C**

**ATOM 1198 C PRO A 215 159.290 143.298 120.914 1.00 0.00 C**

**ATOM 1199 O PRO A 215 158.446 144.116 121.293 1.00 0.00 O**

**ATOM 1200 N PRO A 215 160.383 143.511 123.147 1.00 0.00 N**

**ATOM 1201 CD PRO A 215 159.801 142.596 124.143 1.00 0.00 C**

**ATOM 1202 CG PRO A 215 159.747 141.269 123.422 1.00 0.00 C**

**ATOM 1203 CB PRO A 215 160.459 141.405 122.107 1.00 0.00 C**

**ATOM 1204 CA PRO A 238 153.669 167.082 138.214 1.00 0.00 C**

**ATOM 1205 C PRO A 238 153.665 168.578 137.942 1.00 0.00 C**

**ATOM 1206 O PRO A 238 153.580 169.372 138.891 1.00 0.00 O**

**ATOM 1207 N PRO A 238 152.771 166.295 137.344 1.00 0.00 N**

**ATOM 1208 CD PRO A 238 153.534 165.181 136.739 1.00 0.00 C**

**ATOM 1209 CG PRO A 238 154.722 165.069 137.636 1.00 0.00 C**

**ATOM 1210 CB PRO A 238 155.038 166.457 137.931 1.00 0.00 C**

**ATOM 1211 CA PRO A 289 163.168 169.707 125.810 1.00 0.00 C**

**ATOM 1212 C PRO A 289 163.437 168.220 125.706 1.00 0.00 C**

**ATOM 1213 O PRO A 289 163.176 167.639 124.650 1.00 0.00 O**

**ATOM 1214 N PRO A 289 162.710 170.138 127.143 1.00 0.00 N**

**ATOM 1215 CD PRO A 289 163.749 170.993 127.755 1.00 0.00 C**

**ATOM 1216 CG PRO A 289 164.370 171.637 126.571 1.00 0.00 C**

**ATOM 1217 CB PRO A 289 164.450 170.530 125.596 1.00 0.00 C**

**ATOM 1218 CA PRO A 327 172.007 171.904 145.246 1.00 0.00 C**

**ATOM 1219 C PRO A 327 172.606 173.281 145.470 1.00 0.00 C**

**ATOM 1220 O PRO A 327 172.424 173.865 146.542 1.00 0.00 O**

**ATOM 1221 N PRO A 327 171.554 171.718 143.860 1.00 0.00 N**

**ATOM 1222 CD PRO A 327 172.324 170.635 143.211 1.00 0.00 C**

**ATOM 1223 CG PRO A 327 173.520 170.482 144.087 1.00 0.00 C**

**ATOM 1224 CB PRO A 327 173.051 170.797 145.436 1.00 0.00 C**

**ATOM 1225 CA ASP A 105 171.515 165.109 142.475 1.00 0.00 C**

**ATOM 1226 C ASP A 105 172.908 164.492 142.475 1.00 0.00 C**

**ATOM 1227 O ASP A 105 173.564 164.432 141.428 1.00 0.00 O**

**ATOM 1228 N ASP A 105 170.610 164.374 143.352 1.00 0.00 N**

**ATOM 1229 CB ASP A 105 171.573 166.578 142.890 1.00 0.00 C**

**ATOM 1230 CG ASP A 105 170.292 167.323 142.570 1.00 0.00 C**

**ATOM 1231 OD1 ASP A 105 169.628 166.965 141.574 1.00 0.00 O**

**ATOM 1232 OD2 ASP A 105 169.948 168.265 143.314 1.00 0.00 O**

**ATOM 1233 CA ASP A 128 175.142 144.139 131.058 1.00 0.00 C**

**ATOM 1234 C ASP A 128 174.527 144.333 132.440 1.00 0.00 C**

**ATOM 1235 O ASP A 128 173.501 145.012 132.573 1.00 0.00 O**

**ATOM 1236 N ASP A 128 176.598 144.038 131.115 1.00 0.00 N**

**ATOM 1237 CB ASP A 128 174.562 142.903 130.369 1.00 0.00 C**

**ATOM 1238 CG ASP A 128 174.998 141.602 131.023 1.00 0.00 C**

**ATOM 1239 OD1 ASP A 128 175.834 141.636 131.950 1.00 0.00 O**

**ATOM 1240 OD2 ASP A 128 174.499 140.536 130.606 1.00 0.00 O**

**ATOM 1241 CA ASP A 138 168.511 156.735 135.990 1.00 0.00 C**

**ATOM 1242 C ASP A 138 167.003 156.971 136.082 1.00 0.00 C**

**ATOM 1243 O ASP A 138 166.552 158.069 136.449 1.00 0.00 O**

**ATOM 1244 N ASP A 138 168.922 155.568 136.761 1.00 0.00 N**

**ATOM 1245 CB ASP A 138 168.951 156.556 134.534 1.00 0.00 C**

**ATOM 1246 CG ASP A 138 169.138 157.871 133.819 1.00 0.00 C**

**ATOM 1247 OD1 ASP A 138 169.041 158.922 134.485 1.00 0.00 O**

**ATOM 1248 OD2 ASP A 138 169.382 157.859 132.594 1.00 0.00 O**

**ATOM 1249 CA ASP A 155 153.866 170.915 151.412 1.00 0.00 C**

**ATOM 1250 C ASP A 155 153.831 172.429 151.579 1.00 0.00 C**

**ATOM 1251 O ASP A 155 153.283 172.936 152.562 1.00 0.00 O**

**ATOM 1252 N ASP A 155 153.592 170.521 150.033 1.00 0.00 N**

**ATOM 1253 CB ASP A 155 155.221 170.365 151.854 1.00 0.00 C**

**ATOM 1254 CG ASP A 155 155.642 170.882 153.214 1.00 0.00 C**

**ATOM 1255 OD1 ASP A 155 155.088 170.412 154.229 1.00 0.00 O**

**ATOM 1256 OD2 ASP A 155 156.525 171.762 153.267 1.00 0.00 O**

**ATOM 1257 CA ASP A 168 148.705 168.278 162.061 1.00 0.00 C**

**ATOM 1258 C ASP A 168 149.219 167.506 160.854 1.00 0.00 C**

**ATOM 1259 O ASP A 168 149.221 166.271 160.886 1.00 0.00 O**

**ATOM 1260 N ASP A 168 148.871 169.717 161.927 1.00 0.00 N**

**ATOM 1261 CB ASP A 168 147.229 167.940 162.296 1.00 0.00 C**

**ATOM 1262 CG ASP A 168 147.044 166.697 163.141 1.00 0.00 C**

**ATOM 1263 OD1 ASP A 168 148.012 166.278 163.808 1.00 0.00 O**

**ATOM 1264 OD2 ASP A 168 145.927 166.139 163.141 1.00 0.00 O**

**ATOM 1265 CA ASP A 204 173.544 147.923 111.580 1.00 0.00 C**

**ATOM 1266 C ASP A 204 174.984 148.318 111.860 1.00 0.00 C**

**ATOM 1267 O ASP A 204 175.780 148.438 110.927 1.00 0.00 O**

**ATOM 1268 N ASP A 204 172.984 147.035 112.593 1.00 0.00 N**

**ATOM 1269 CB ASP A 204 172.686 149.188 111.451 1.00 0.00 C**

**ATOM 1270 CG ASP A 204 172.482 149.897 112.778 1.00 0.00 C**

**ATOM 1271 OD1 ASP A 204 172.766 149.288 113.832 1.00 0.00 O**

**ATOM 1272 OD2 ASP A 204 172.039 151.064 112.767 1.00 0.00 O**

**ATOM 1273 CA ASP A 206 177.261 145.244 114.815 1.00 0.00 C**

**ATOM 1274 C ASP A 206 178.024 145.486 116.114 1.00 0.00 C**

**ATOM 1275 O ASP A 206 179.072 144.888 116.367 1.00 0.00 O**

**ATOM 1276 N ASP A 206 176.717 146.496 114.274 1.00 0.00 N**

**ATOM 1277 CB ASP A 206 178.130 144.504 113.791 1.00 0.00 C**

**ATOM 1278 CG ASP A 206 177.314 143.904 112.664 1.00 0.00 C**

**ATOM 1279 OD1 ASP A 206 176.153 143.515 112.910 1.00 0.00 O**

**ATOM 1280 OD2 ASP A 206 177.832 143.823 111.530 1.00 0.00 O**

**ATOM 1281 CA ASP A 216 158.191 143.020 118.753 1.00 0.00 C**

**ATOM 1282 C ASP A 216 156.833 142.455 119.150 1.00 0.00 C**

**ATOM 1283 O ASP A 216 155.823 142.860 118.564 1.00 0.00 O**

**ATOM 1284 N ASP A 216 159.242 142.713 119.715 1.00 0.00 N**

**ATOM 1285 CB ASP A 216 158.583 142.500 117.368 1.00 0.00 C**

**ATOM 1286 CA ASP A 217 155.503 141.041 120.612 1.00 0.00 C**

**ATOM 1287 C ASP A 217 154.746 142.152 121.328 1.00 0.00 C**

**ATOM 1288 O ASP A 217 153.530 142.297 121.157 1.00 0.00 O**

**ATOM 1289 N ASP A 217 156.786 141.522 120.106 1.00 0.00 N**

**ATOM 1290 CB ASP A 217 155.721 139.850 121.544 1.00 0.00 C**

**ATOM 1291 CA ASP A 218 154.862 144.138 122.734 1.00 0.00 C**

**ATOM 1292 C ASP A 218 154.726 145.276 121.733 1.00 0.00 C**

**ATOM 1293 O ASP A 218 153.985 146.236 122.000 1.00 0.00 O**

**ATOM 1294 N ASP A 218 155.450 142.947 122.136 1.00 0.00 N**

**ATOM 1295 CB ASP A 218 155.701 144.595 123.928 1.00 0.00 C**

**ATOM 1296 CA ASP A 223 156.230 151.033 124.221 1.00 0.00 C**

**ATOM 1297 C ASP A 223 155.605 152.414 124.394 1.00 0.00 C**

**ATOM 1298 O ASP A 223 155.945 153.143 125.331 1.00 0.00 O**

**ATOM 1299 N ASP A 223 155.204 150.013 124.021 1.00 0.00 N**

**ATOM 1300 CB ASP A 223 157.213 151.024 123.050 1.00 0.00 C**

**ATOM 1301 CG ASP A 223 158.391 151.957 123.268 1.00 0.00 C**

**ATOM 1302 OD1 ASP A 223 158.679 152.292 124.437 1.00 0.00 O**

**ATOM 1303 OD2 ASP A 223 159.027 152.360 122.273 1.00 0.00 O**

**ATOM 1304 CA ASP A 266 156.292 193.940 149.570 1.00 0.00 C**

**ATOM 1305 C ASP A 266 157.533 193.536 148.786 1.00 0.00 C**

**ATOM 1306 O ASP A 266 157.464 192.649 147.930 1.00 0.00 O**

**ATOM 1307 N ASP A 266 156.594 194.132 150.984 1.00 0.00 N**

**ATOM 1308 CB ASP A 266 155.681 195.211 148.986 1.00 0.00 C**

**ATOM 1309 CG ASP A 266 154.592 194.920 147.972 1.00 0.00 C**

**ATOM 1310 OD1 ASP A 266 154.315 193.728 147.725 1.00 0.00 O**

**ATOM 1311 OD2 ASP A 266 154.011 195.881 147.427 1.00 0.00 O**

**ATOM 1312 CA ASP A 334 168.309 179.118 151.390 1.00 0.00 C**

**ATOM 1313 C ASP A 334 169.045 179.751 152.565 1.00 0.00 C**

**ATOM 1314 O ASP A 334 170.033 179.197 153.056 1.00 0.00 O**

**ATOM 1315 N ASP A 334 169.070 179.366 150.173 1.00 0.00 N**

**ATOM 1316 CB ASP A 334 168.114 177.613 151.595 1.00 0.00 C**

**ATOM 1317 CG ASP A 334 166.920 177.292 152.465 1.00 0.00 C**

**ATOM 1318 OD1 ASP A 334 166.258 178.237 152.937 1.00 0.00 O**

**ATOM 1319 OD2 ASP A 334 166.641 176.093 152.678 1.00 0.00 O**

**ATOM 1320 CA ASN A 77 176.562 167.875 146.193 1.00 0.00 C**

**ATOM 1321 C ASN A 77 177.354 167.418 147.412 1.00 0.00 C**

**ATOM 1322 O ASN A 77 176.774 167.195 148.482 1.00 0.00 O**

**ATOM 1323 N ASN A 77 177.286 168.883 145.426 1.00 0.00 N**

**ATOM 1324 CB ASN A 77 176.212 166.693 145.293 1.00 0.00 C**

**ATOM 1325 CG ASN A 77 174.965 166.946 144.476 1.00 0.00 C**

**ATOM 1326 OD1 ASN A 77 173.935 167.354 145.012 1.00 0.00 O**

**ATOM 1327 ND2 ASN A 77 175.058 166.731 143.169 1.00 0.00 N**

**ATOM 1328 CA ASN A 95 165.305 169.331 155.739 1.00 0.00 C**

**ATOM 1329 C ASN A 95 165.464 167.938 155.144 1.00 0.00 C**

**ATOM 1330 O ASN A 95 166.451 167.659 154.451 1.00 0.00 O**

**ATOM 1331 N ASN A 95 163.909 169.756 155.712 1.00 0.00 N**

**ATOM 1332 CB ASN A 95 165.837 169.370 157.169 1.00 0.00 C**

**ATOM 1333 CG ASN A 95 166.493 170.690 157.509 1.00 0.00 C**

**ATOM 1334 OD1 ASN A 95 166.593 171.581 156.665 1.00 0.00 O**

**ATOM 1335 ND2 ASN A 95 166.945 170.825 158.750 1.00 0.00 N**

**ATOM 1336 CA ASN A 100 166.824 163.528 149.522 1.00 0.00 C**

**ATOM 1337 C ASN A 100 167.138 164.124 148.154 1.00 0.00 C**

**ATOM 1338 O ASN A 100 167.641 163.420 147.271 1.00 0.00 O**

**ATOM 1339 N ASN A 100 167.133 164.462 150.604 1.00 0.00 N**

**ATOM 1340 CB ASN A 100 165.360 163.087 149.583 1.00 0.00 C**

**ATOM 1341 CG ASN A 100 165.070 162.183 150.764 1.00 0.00 C**

**ATOM 1342 OD1 ASN A 100 164.261 162.508 151.628 1.00 0.00 O**

**ATOM 1343 ND2 ASN A 100 165.738 161.037 150.805 1.00 0.00 N**

**ATOM 1344 CA ASN A 122 179.466 155.106 121.078 1.00 0.00 C**

**ATOM 1345 C ASN A 122 179.063 153.761 121.678 1.00 0.00 C**

**ATOM 1346 O ASN A 122 177.946 153.298 121.437 1.00 0.00 O**

**ATOM 1347 N ASN A 122 180.792 155.578 121.476 1.00 0.00 N**

**ATOM 1348 CB ASN A 122 179.356 155.045 119.549 1.00 0.00 C**

**ATOM 1349 CG ASN A 122 179.363 156.420 118.910 1.00 0.00 C**

**ATOM 1350 OD1 ASN A 122 178.701 157.341 119.387 1.00 0.00 O**

**ATOM 1351 ND2 ASN A 122 180.117 156.566 117.827 1.00 0.00 N**

**ATOM 1352 CA ASN A 141 166.457 158.904 140.347 1.00 0.00 C**

**ATOM 1353 C ASN A 141 165.649 160.053 139.757 1.00 0.00 C**

**ATOM 1354 O ASN A 141 165.267 160.985 140.475 1.00 0.00 O**

**ATOM 1355 N ASN A 141 165.967 157.615 139.897 1.00 0.00 N**

**ATOM 1356 CB ASN A 141 167.937 159.003 139.971 1.00 0.00 C**

**ATOM 1357 CG ASN A 141 168.646 160.129 140.668 1.00 0.00 C**

**ATOM 1358 OD1 ASN A 141 168.039 160.925 141.375 1.00 0.00 O**

**ATOM 1359 ND2 ASN A 141 169.953 160.176 140.507 1.00 0.00 N**

**ATOM 1360 CA ASN A 179 158.865 160.549 151.516 1.00 0.00 C**

**ATOM 1361 C ASN A 179 159.142 159.069 151.252 1.00 0.00 C**

**ATOM 1362 O ASN A 179 159.545 158.691 150.145 1.00 0.00 O**

**ATOM 1363 N ASN A 179 157.611 160.723 152.247 1.00 0.00 N**

**ATOM 1364 CB ASN A 179 160.001 161.212 152.308 1.00 0.00 C**

**ATOM 1365 CG ASN A 179 161.326 161.258 151.552 1.00 0.00 C**

**ATOM 1366 OD1 ASN A 179 161.870 160.240 151.130 1.00 0.00 O**

**ATOM 1367 ND2 ASN A 179 161.853 162.464 151.387 1.00 0.00 N**

**ATOM 1368 CA ASN A 268 160.595 190.636 150.375 1.00 0.00 C**

**ATOM 1369 C ASN A 268 159.673 189.610 149.731 1.00 0.00 C**

**ATOM 1370 O ASN A 268 160.131 188.550 149.289 1.00 0.00 O**

**ATOM 1371 N ASN A 268 160.234 191.994 149.985 1.00 0.00 N**

**ATOM 1372 CB ASN A 268 160.571 190.505 151.897 1.00 0.00 C**

**ATOM 1373 CG ASN A 268 161.616 189.539 152.414 1.00 0.00 C**

**ATOM 1374 OD1 ASN A 268 162.315 188.890 151.637 1.00 0.00 O**

**ATOM 1375 ND2 ASN A 268 161.731 189.442 153.732 1.00 0.00 N**

**ATOM 1376 CA ASN A 322 170.192 171.269 136.334 1.00 0.00 C**

**ATOM 1377 C ASN A 322 170.629 170.505 137.575 1.00 0.00 C**

**ATOM 1378 O ASN A 322 170.489 170.992 138.702 1.00 0.00 O**

**ATOM 1379 N ASN A 322 171.215 171.202 135.293 1.00 0.00 N**

**ATOM 1380 CB ASN A 322 168.877 170.696 135.805 1.00 0.00 C**

**ATOM 1381 CG ASN A 322 167.724 170.865 136.778 1.00 0.00 C**

**ATOM 1382 OD1 ASN A 322 167.792 171.661 137.715 1.00 0.00 O**

**ATOM 1383 ND2 ASN A 322 166.660 170.100 136.566 1.00 0.00 N**

**ATOM 1384 CA ASN A 326 170.099 172.479 141.919 1.00 0.00 C**

**ATOM 1385 C ASN A 326 170.536 172.516 143.385 1.00 0.00 C**

**ATOM 1386 O ASN A 326 169.936 173.292 144.155 1.00 0.00 O**

**ATOM 1387 N ASN A 326 171.218 172.619 140.986 1.00 0.00 N**

**ATOM 1388 CB ASN A 326 169.299 171.217 141.548 1.00 0.00 C**

**ATOM 1389 CG ASN A 326 168.083 170.982 142.440 1.00 0.00 C**

**ATOM 1390 OD1 ASN A 326 168.199 170.731 143.641 1.00 0.00 O**

**ATOM 1391 ND2 ASN A 326 166.902 171.056 141.840 1.00 0.00 N**

**ATOM 1392 CA ASN A 336 168.892 179.635 157.019 1.00 0.00 C**

**ATOM 1393 C ASN A 336 170.207 178.866 157.006 1.00 0.00 C**

**ATOM 1394 O ASN A 336 170.944 178.856 158.001 1.00 0.00 O**

**ATOM 1395 N ASN A 336 168.655 180.272 155.725 1.00 0.00 N**

**ATOM 1396 CB ASN A 336 167.722 178.721 157.390 1.00 0.00 C**

**ATOM 1397 CG ASN A 336 167.417 177.683 156.323 1.00 0.00 C**

**ATOM 1398 OD1 ASN A 336 168.068 177.630 155.281 1.00 0.00 O**

**ATOM 1399 ND2 ASN A 336 166.419 176.847 156.585 1.00 0.00 N**

**ATOM 1400 CA LYS A 89 170.115 168.214 161.615 1.00 0.00 C**

**ATOM 1401 C LYS A 89 168.833 167.902 160.855 1.00 0.00 C**

**ATOM 1402 O LYS A 89 168.339 168.705 160.059 1.00 0.00 O**

**ATOM 1403 N LYS A 89 170.608 169.525 161.221 1.00 0.00 N**

**ATOM 1404 CB LYS A 89 169.888 168.141 163.130 1.00 0.00 C**

**ATOM 1405 CA LYS A 91 164.573 168.335 162.391 1.00 0.00 C**

**ATOM 1406 C LYS A 91 163.293 168.683 161.634 1.00 0.00 C**

**ATOM 1407 O LYS A 91 162.238 168.089 161.880 1.00 0.00 O**

**ATOM 1408 N LYS A 91 165.639 167.879 161.504 1.00 0.00 N**

**ATOM 1409 CB LYS A 91 165.057 169.541 163.204 1.00 0.00 C**

**ATOM 1410 CA LYS A 132 170.871 147.423 133.893 1.00 0.00 C**

**ATOM 1411 C LYS A 132 170.803 148.256 135.165 1.00 0.00 C**

**ATOM 1412 O LYS A 132 169.805 148.944 135.419 1.00 0.00 O**

**ATOM 1413 N LYS A 132 172.196 147.516 133.283 1.00 0.00 N**

**ATOM 1414 CB LYS A 132 170.539 145.963 134.204 1.00 0.00 C**

**ATOM 1415 CG LYS A 132 170.147 145.117 133.007 1.00 0.00 C**

**ATOM 1416 CD LYS A 132 170.481 143.653 133.259 1.00 0.00 C**

**ATOM 1417 CE LYS A 132 169.508 142.720 132.557 1.00 0.00 C**

**ATOM 1418 NZ LYS A 132 168.224 142.580 133.296 1.00 0.00 N**

**ATOM 1419 CA LYS A 165 145.778 171.440 158.630 1.00 0.00 C**

**ATOM 1420 C LYS A 165 147.294 171.306 158.630 1.00 0.00 C**

**ATOM 1421 O LYS A 165 147.797 170.180 158.725 1.00 0.00 O**

**ATOM 1422 N LYS A 165 145.324 172.812 158.807 1.00 0.00 N**

**ATOM 1423 CB LYS A 165 145.216 170.872 157.325 1.00 0.00 C**

**ATOM 1424 CG LYS A 165 143.743 171.175 157.085 1.00 0.00 C**

**ATOM 1425 CD LYS A 165 142.860 170.684 158.223 1.00 0.00 C**

**ATOM 1426 CE LYS A 165 141.395 170.684 157.820 1.00 0.00 C**

**ATOM 1427 NZ LYS A 165 141.119 171.643 156.715 1.00 0.00 N**

**ATOM 1428 CA LYS A 174 154.197 162.389 158.351 1.00 0.00 C**

**ATOM 1429 C LYS A 174 155.026 162.755 157.126 1.00 0.00 C**

**ATOM 1430 O LYS A 174 154.843 162.171 156.049 1.00 0.00 O**

**ATOM 1431 N LYS A 174 155.012 162.389 159.563 1.00 0.00 N**

**ATOM 1432 CB LYS A 174 153.013 163.342 158.510 1.00 0.00 C**

**ATOM 1433 CG LYS A 174 152.076 163.374 157.313 1.00 0.00 C**

**ATOM 1434 CD LYS A 174 151.440 162.016 157.068 1.00 0.00 C**

**ATOM 1435 CE LYS A 174 150.398 161.691 158.124 1.00 0.00 C**

**ATOM 1436 NZ LYS A 174 149.163 162.506 157.961 1.00 0.00 N**

**ATOM 1437 CA LYS A 176 159.121 161.054 156.303 1.00 0.00 C**

**ATOM 1438 C LYS A 176 158.298 159.870 155.812 1.00 0.00 C**

**ATOM 1439 O LYS A 176 158.777 159.116 154.965 1.00 0.00 O**

**ATOM 1440 N LYS A 176 158.280 162.182 156.673 1.00 0.00 N**

**ATOM 1441 CB LYS A 176 160.032 160.651 157.468 1.00 0.00 C**

**ATOM 1442 CG LYS A 176 159.360 159.914 158.611 1.00 0.00 C**

**ATOM 1443 CD LYS A 176 160.312 159.716 159.778 1.00 0.00 C**

**ATOM 1444 CE LYS A 176 161.372 158.676 159.451 1.00 0.00 C**

**ATOM 1445 NZ LYS A 176 162.232 158.371 160.626 1.00 0.00 N**

**ATOM 1446 CA LYS A 200 170.738 144.304 122.868 1.00 0.00 C**

**ATOM 1447 C LYS A 200 171.940 144.640 121.998 1.00 0.00 C**

**ATOM 1448 O LYS A 200 172.757 145.499 122.339 1.00 0.00 O**

**ATOM 1449 N LYS A 200 171.006 144.724 124.236 1.00 0.00 N**

**ATOM 1450 CB LYS A 200 169.472 144.967 122.314 1.00 0.00 C**

**ATOM 1451 CA LYS A 227 156.020 156.077 127.435 1.00 0.00 C**

**ATOM 1452 C LYS A 227 155.109 157.285 127.621 1.00 0.00 C**

**ATOM 1453 O LYS A 227 155.490 158.248 128.288 1.00 0.00 O**

**ATOM 1454 N LYS A 227 155.291 154.820 127.566 1.00 0.00 N**

**ATOM 1455 CB LYS A 227 156.724 156.129 126.080 1.00 0.00 C**

**ATOM 1456 CG LYS A 227 157.931 157.051 126.045 1.00 0.00 C**

**ATOM 1457 CD LYS A 227 158.748 156.829 124.785 1.00 0.00 C**

**ATOM 1458 CE LYS A 227 160.002 157.682 124.779 1.00 0.00 C**

**ATOM 1459 NZ LYS A 227 160.824 157.428 123.567 1.00 0.00 N**

**ATOM 1460 CA LYS A 254 148.260 188.307 148.750 1.00 0.00 C**

**ATOM 1461 C LYS A 254 147.053 188.614 149.627 1.00 0.00 C**

**ATOM 1462 O LYS A 254 146.857 189.767 150.027 1.00 0.00 O**

**ATOM 1463 N LYS A 254 148.752 186.949 148.955 1.00 0.00 N**

**ATOM 1464 CB LYS A 254 147.901 188.525 147.278 1.00 0.00 C**

**ATOM 1465 CG LYS A 254 149.042 188.374 146.259 1.00 0.00 C**

**ATOM 1466 CD LYS A 254 150.065 189.517 146.287 1.00 0.00 C**

**ATOM 1467 CE LYS A 254 151.303 189.199 147.120 1.00 0.00 C**

**ATOM 1468 NZ LYS A 254 152.320 190.281 147.065 1.00 0.00 N**

**ATOM 1469 CA LYS A 265 157.340 193.416 153.194 1.00 0.00 C**

**ATOM 1470 C LYS A 265 156.993 193.108 151.744 1.00 0.00 C**

**ATOM 1471 O LYS A 265 157.090 191.954 151.307 1.00 0.00 O**

**ATOM 1472 N LYS A 265 158.086 194.667 153.311 1.00 0.00 N**

**ATOM 1473 CB LYS A 265 156.068 193.480 154.039 1.00 0.00 C**

**ATOM 1474 CG LYS A 265 156.288 193.902 155.479 1.00 0.00 C**

**ATOM 1475 CD LYS A 265 154.980 194.338 156.120 1.00 0.00 C**

**ATOM 1476 CE LYS A 265 154.067 193.158 156.390 1.00 0.00 C**

**ATOM 1477 NZ LYS A 265 152.772 193.587 156.986 1.00 0.00 N**

**ATOM 1478 CA LYS A 338 173.715 180.835 155.182 1.00 0.00 C**

**ATOM 1479 C LYS A 338 173.906 181.338 156.608 1.00 0.00 C**

**ATOM 1480 O LYS A 338 175.031 181.643 157.019 1.00 0.00 O**

**ATOM 1481 N LYS A 338 172.723 179.764 155.123 1.00 0.00 N**

**ATOM 1482 CB LYS A 338 173.298 181.987 154.269 1.00 0.00 C**

**ATOM 1483 CG LYS A 338 173.211 181.627 152.800 1.00 0.00 C**

**ATOM 1484 CD LYS A 338 172.525 182.732 152.017 1.00 0.00 C**

**ATOM 1485 CE LYS A 338 173.407 183.964 151.910 1.00 0.00 C**

**ATOM 1486 NZ LYS A 338 174.577 183.735 151.020 1.00 0.00 N**

**ATOM 1487 CA CYS A 131 174.140 148.722 132.413 1.00 0.00 C**

**ATOM 1488 C CYS A 131 172.689 148.696 132.888 1.00 0.00 C**

**ATOM 1489 O CYS A 131 172.018 149.736 132.915 1.00 0.00 O**

**ATOM 1490 N CYS A 131 174.993 147.921 133.290 1.00 0.00 N**

**ATOM 1491 CB CYS A 131 174.225 148.249 130.963 1.00 0.00 C**

**ATOM 1492 SG CYS A 131 172.951 149.045 129.942 1.00 0.00 S**

**ATOM 1493 CA CYS A 161 147.259 178.359 151.941 1.00 0.00 C**

**ATOM 1494 C CYS A 161 146.398 177.717 153.022 1.00 0.00 C**

**ATOM 1495 O CYS A 161 145.290 178.195 153.293 1.00 0.00 O**

**ATOM 1496 N CYS A 161 148.682 178.191 152.211 1.00 0.00 N**

**ATOM 1497 CB CYS A 161 146.912 177.780 150.571 1.00 0.00 C**

**ATOM 1498 SG CYS A 161 145.180 177.970 150.082 1.00 0.00 S**

**ATOM 1499 CA CYS A 181 155.828 156.242 150.127 1.00 0.00 C**

**ATOM 1500 C CYS A 181 156.204 156.595 148.692 1.00 0.00 C**

**ATOM 1501 O CYS A 181 156.130 155.738 147.801 1.00 0.00 O**

**ATOM 1502 N CYS A 181 156.828 156.713 151.087 1.00 0.00 N**

**ATOM 1503 CB CYS A 181 154.449 156.803 150.476 1.00 0.00 C**

**ATOM 1504 SG CYS A 181 153.695 156.061 151.939 1.00 0.00 S**

**ATOM 1505 CA CYS A 210 172.054 148.164 125.912 1.00 0.00 C**

**ATOM 1506 C CYS A 210 171.038 149.282 126.116 1.00 0.00 C**

**ATOM 1507 O CYS A 210 171.393 150.454 126.272 1.00 0.00 O**

**ATOM 1508 N CYS A 210 173.032 148.523 124.898 1.00 0.00 N**

**ATOM 1509 CB CYS A 210 172.745 147.810 127.231 1.00 0.00 C**

**ATOM 1510 SG CYS A 210 173.689 149.103 128.055 1.00 0.00 S**

**ATOM 1511 CA CYS A 229 151.892 157.147 130.719 1.00 0.00 C**

**ATOM 1512 C CYS A 229 152.949 157.734 131.645 1.00 0.00 C**

**ATOM 1513 O CYS A 229 152.634 158.516 132.550 1.00 0.00 O**

**ATOM 1514 N CYS A 229 152.330 157.198 129.329 1.00 0.00 N**

**ATOM 1515 CB CYS A 229 151.565 155.707 131.114 1.00 0.00 C**

**ATOM 1516 SG CYS A 229 151.108 155.493 132.850 1.00 0.00 S**

**ATOM 1517 CA CYS A 245 149.861 174.796 144.161 1.00 0.00 C**

**ATOM 1518 C CYS A 245 150.551 176.135 144.387 1.00 0.00 C**

**ATOM 1519 O CYS A 245 150.174 176.880 145.296 1.00 0.00 O**

**ATOM 1520 N CYS A 245 149.507 174.612 142.764 1.00 0.00 N**

**ATOM 1521 CB CYS A 245 150.755 173.647 144.635 1.00 0.00 C**

**ATOM 1522 SG CYS A 245 149.847 172.170 145.145 1.00 0.00 S**

**ATOM 1523 CA CYS A 286 165.530 171.708 132.081 1.00 0.00 C**

**ATOM 1524 C CYS A 286 164.877 170.343 131.912 1.00 0.00 C**

**ATOM 1525 O CYS A 286 165.583 169.380 131.593 1.00 0.00 O**

**ATOM 1526 N CYS A 286 164.594 172.820 132.021 1.00 0.00 N**

**ATOM 1527 CB CYS A 286 166.302 171.748 133.402 1.00 0.00 C**

**ATOM 1528 SG CYS A 286 167.563 173.045 133.439 1.00 0.00 S**

**ATOM 1529 CA CYS A 315 170.014 168.538 125.518 1.00 0.00 C**

**ATOM 1530 C CYS A 315 170.078 167.747 126.815 1.00 0.00 C**

**ATOM 1531 O CYS A 315 169.371 168.091 127.767 1.00 0.00 O**

**ATOM 1532 N CYS A 315 171.295 168.609 124.837 1.00 0.00 N**

**ATOM 1533 CB CYS A 315 168.967 167.916 124.589 1.00 0.00 C**

**ATOM 1534 SG CYS A 315 167.270 168.381 124.955 1.00 0.00 S**

**ATOM 1535 CA CYS A 340 174.079 178.611 160.224 1.00 0.00 C**

**ATOM 1536 C CYS A 340 175.443 178.259 159.644 1.00 0.00 C**

**ATOM 1537 O CYS A 340 176.170 177.469 160.257 1.00 0.00 O**

**ATOM 1538 N CYS A 340 173.377 179.624 159.445 1.00 0.00 N**

**ATOM 1539 CB CYS A 340 173.227 177.344 160.347 1.00 0.00 C**

**ATOM 1540 SG CYS A 340 173.142 176.336 158.854 1.00 0.00 S**

**ATOM 1541 CA SER A 67 185.087 168.074 133.158 1.00 0.00 C**

**ATOM 1542 C SER A 67 184.221 168.997 134.005 1.00 0.00 C**

**ATOM 1543 O SER A 67 183.947 168.706 135.177 1.00 0.00 O**

**ATOM 1544 N SER A 67 184.319 167.522 132.052 1.00 0.00 N**

**ATOM 1545 CB SER A 67 186.314 168.817 132.625 1.00 0.00 C**

**ATOM 1546 OG SER A 67 187.217 167.927 131.993 1.00 0.00 O**

**ATOM 1547 CA SER A 78 179.510 166.925 148.422 1.00 0.00 C**

**ATOM 1548 C SER A 78 179.486 168.005 149.497 1.00 0.00 C**

**ATOM 1549 O SER A 78 179.386 167.694 150.690 1.00 0.00 O**

**ATOM 1550 N SER A 78 178.680 167.291 147.279 1.00 0.00 N**

**ATOM 1551 CB SER A 78 180.944 166.657 147.965 1.00 0.00 C**
[truncated: 192,400 more chars]
