# Supplementary material for: Development and Biological Characterization of Fluorescent Dynorphins for the Visualization of Kappa Opioid Receptors
Source: J Med Chem. 2026 Jun 5;69(12):14098–113. doi: 10.1021/acs.jmedchem.5c03072 (PMC13312453; doi:10.1021/acs.jmedchem.5c03072)
Supplement: Supplementary file 3 [file jm5c03072_si_003.pdf]

Supporting Information

Development and Biological Characterization of Fluorescent Dynorphins for the Visualization of Kappa Opioid Receptors

Predrag Kalaba[‡,1], Monika Perisic Böhm[‡,1,2], Filip Đikić[‡,3], Nataša Tomašević[4], Ruth Drdla-Schutting[5], Simon Hasinger[4], Dylan M. Ines[1], Alexandra Wolf[3], Mireia Belil-Catalina[1], Sharon D. Bryant[6], Mariana Spetea[7], Christian W. Gruber[#,4], Markus Muttenthaler[#,1,8], Erik Keimpema[#,3]

[1]*Institute of Biological Chemistry, Faculty of Chemistry, University of Vienna, Vienna, 1090, Austria*
[2]*Vienna Doctoral School in Chemistry, University of Vienna, Vienna, 1090, Austria*
[3]*Department of Molecular Neurosciences, Center for Brain Research, Medical University of Vienna, Vienna, 1090, Austria*
[4]*Institute of Pharmacology, Center for Pharmacology and Physiology, Medical University of Vienna, Vienna, 1090, Austria*
[5]*Department of Neurophysiology, Center for Brain Research, Medical University of Vienna, Vienna, 1090, Austria*
[6]*Inte:Ligand GmbH, Vienna, 1070, Austria*
[7]*Department of Pharmaceutical Chemistry, Institute of Pharmacy and Center for Molecular Biosciences Innsbruck (CMBI), University of Innsbruck, Innsbruck, 6020, Austria*
[8]*Institute for Molecular Bioscience, The University of Queensland, Brisbane, Queensland, 4067, Australia*

‡ contributed equally

[#] *Address correspondence to:* Ap. Prof. Dr. Erik Keimpema, *Email:* erik.keimpema@meduniwien.ac.at

or

Prof. Dr. Markus Muttenthaler, *Email:* markus.muttenthaler@univie.ac.at

or

Assoc. Prof. Dr. Christian W. Gruber, *Email:* christian.w.gruber@meduniwien.ac.at

Contents:

- Supporting Table 1 – Page S2
- Supporting Figure 1 – Page S3-S14
- Supporting Figure 2 – Page S15
- Supporting Figure 3 – Page S16
- Supporting Figure 4 – Page S16
- Supporting Figure 5 – Page S17
- Supporting Figure 6 – Page S18
- Supporting Figure 7 – Page S18
- Supporting Figure 8 – Page S19
- Supporting Figure 9 – Page S20
- Supporting Figure 10 – Page S21
- Supporting Figure 11 – Page S22
- Supporting Figure 12 – Page S23
- Supporting Figure 13 – Page S24
- Supporting Figure 14 – Page S24

Table S1. Compound Summary.

Tracer	Yield	Purity	Conc.	Ret. time	Calc. [m/z]	Obs. [m/z]	Charge
**1** [$Y^1(C_2H_4$-$Cy3_s)$,P^3,R^8]Dyn A(1-8)-NH_2	9%	99%	901 µM	17.2 min	595.9658	595.9662	$[M+3H]^{3+}$
**2** [$Y^1(C_2H_4$-$Cy3_s)$,P^3,R^8]Dyn A(1-8)-OH	4%	99%	706 µM	17.3 min	596.2938	596.2943	$[M+3H]^{3+}$
**3** [$Y^1(C_2H_4$-$Cy3_s)$,P^3,R^8]Dyn A(1-11)-OH	10%	99%	649 µM	17.0 min	542.7844	542.7850	$[M+4H]^{4+}$
**4** [$Y^1(C_2H_4$-$Cy3_s)$,P^3,R^8]Dyn A(1-11)-NH_2	3%	99%	676 µM	16.9 min	542.5384	542.5388	$[M+4H]^{4+}$
**5** [P^3,R^8,K^{13}(AEEA-$Cy3_s$)]Dyn A(1-13)-NH_2	2%	98%	565 µM	17.6 min	628.3411	628.3416	$[M+4H]^{4+}$
**6** [P^3,R^8,K^{11}(AEEA-$Cy3_s$)]Dyn A(1-13)-NH_2	4%	97%	605 µM	17.5 min	628.3411	628.3414	$[M+4H]^{4+}$
**7** [P^3,R^8,K^{11}(AEEA-$Cy3_s$)]Dyn A(1-11)-OH	1%	96%	517 µM	17.2 min	568.0463	568.0472	$[M+4H]^{4+}$
**8** [P^3,R^8,K^{11}(AEEA-$Cy3_s$)]Dyn A(1-11)-NH_2	1%	95%	569 µM	17.3 min	568.2923	568.2930	$[M+4H]^{4+}$
**9** [P^3,R^8,K^{13}(AEEA-$Cy3_s$)]Dyn A(1-13)-OH	12%	99%	368 µM	17.2 min	628.5871	628.5876	$[M+4H]^{4+}$
**10** [P^3,R^8,K^{11}(AEEA-$Cy3_s$)]Dyn A(1-13)-OH	10%	99%	574 µM	17.0 min	628.5871	628.5875	$[M+4H]^{4+}$
[P^3,R^8]Dyn A(1-11)-OH	79%	99%	1491 µM	15.8 min	362.2205	362.2211	$[M+4H]^{4+}$

Abbreviations: AEEA = (2-(2-azidoethoxy)ethyloxy)acetic acid; C_2H_4 = ethylene linker; $Cy3_s$ = sulfonated Cyanine 3.

1: H_2N-Y(C_2H_4-Cy3$_s$)GPFLRRR-$CONH_2$

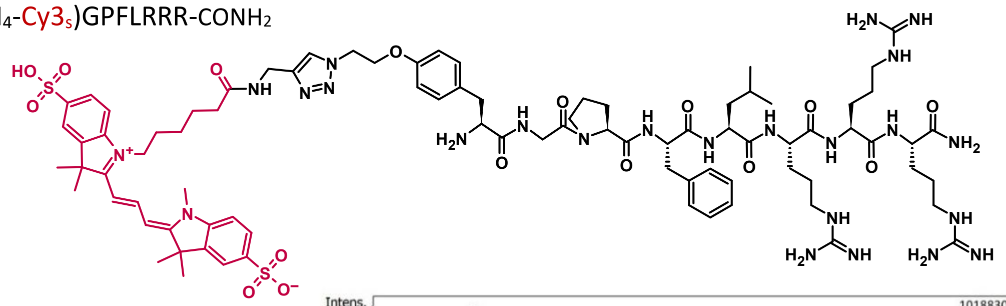

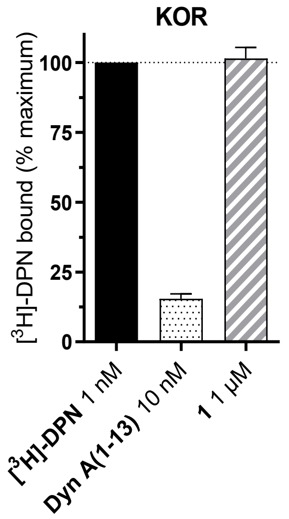

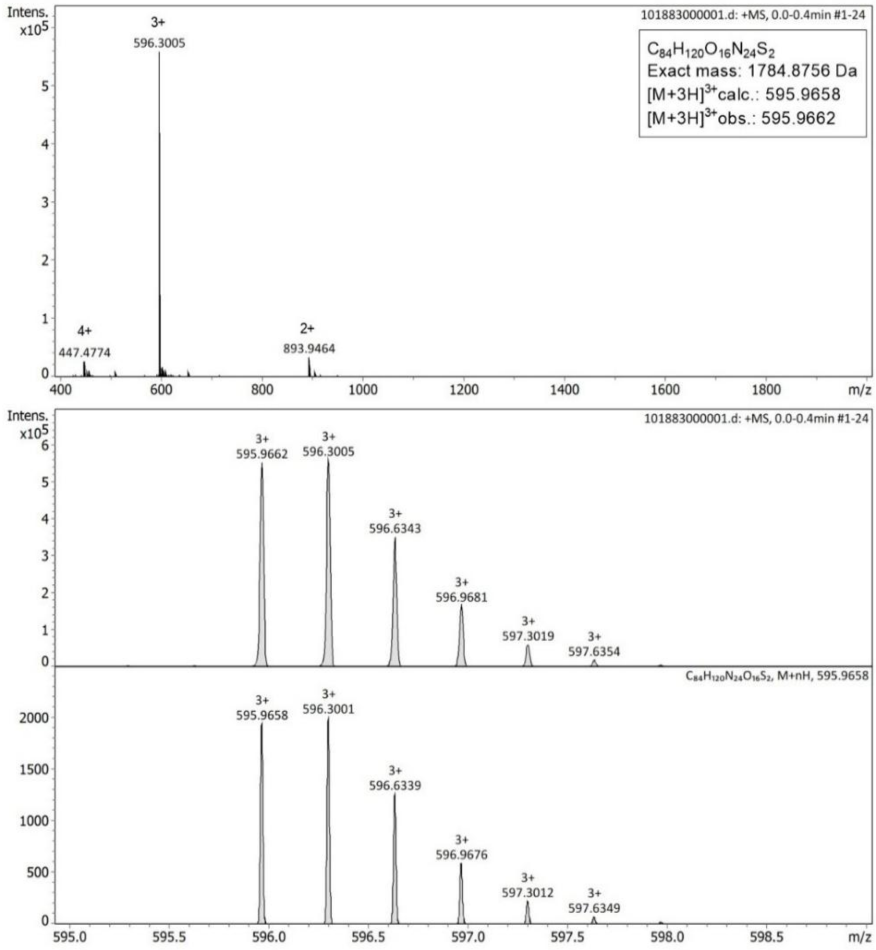

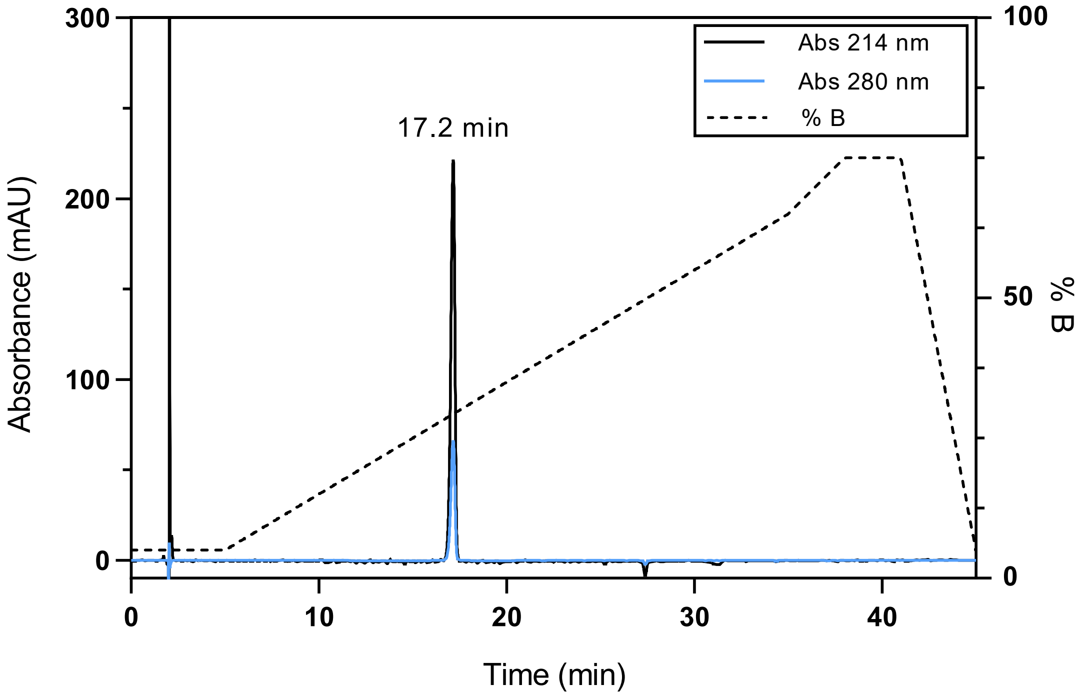

2: H_2N-Y(C_2H_4-Cy3$_s$)GPFLRRR-COOH

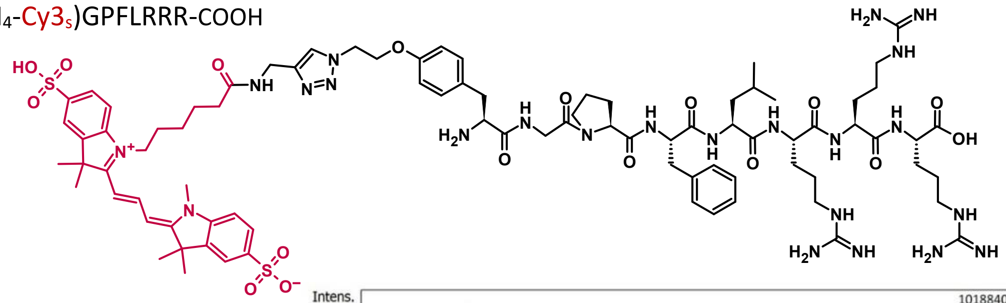

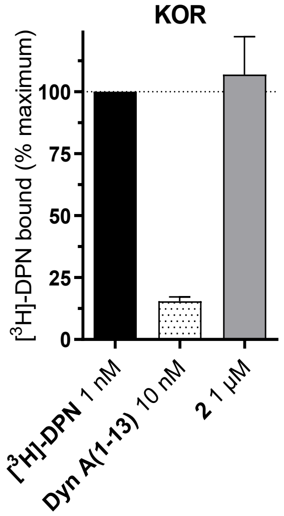

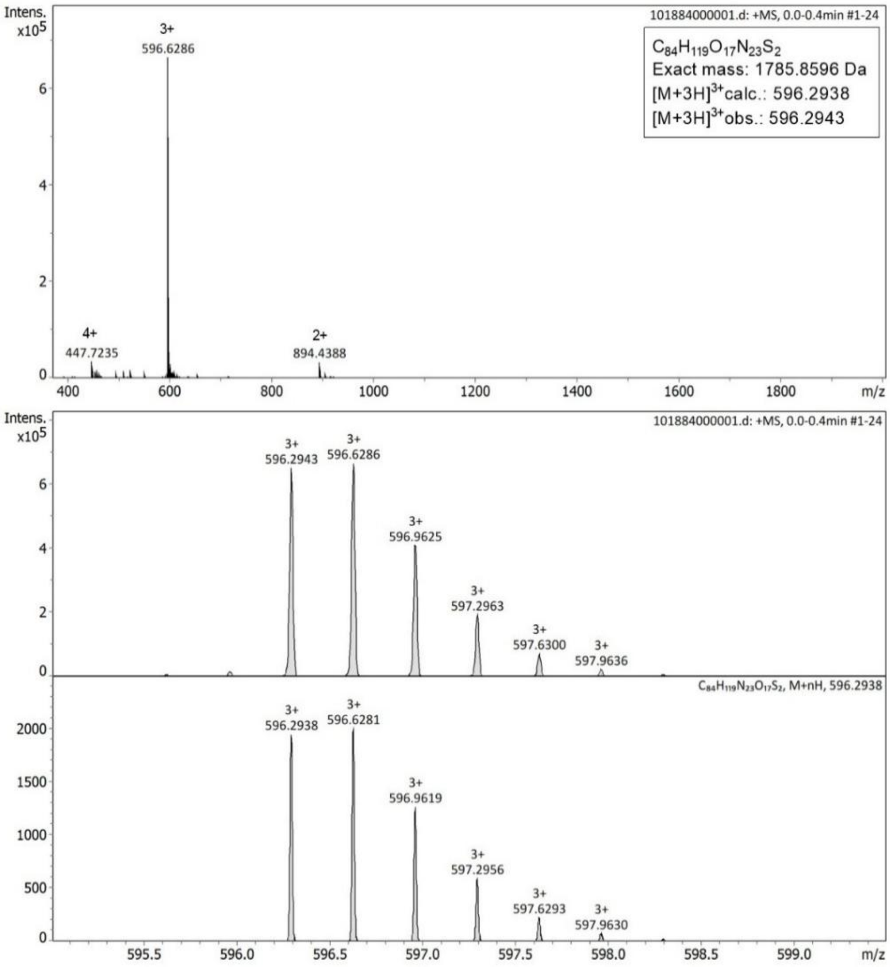

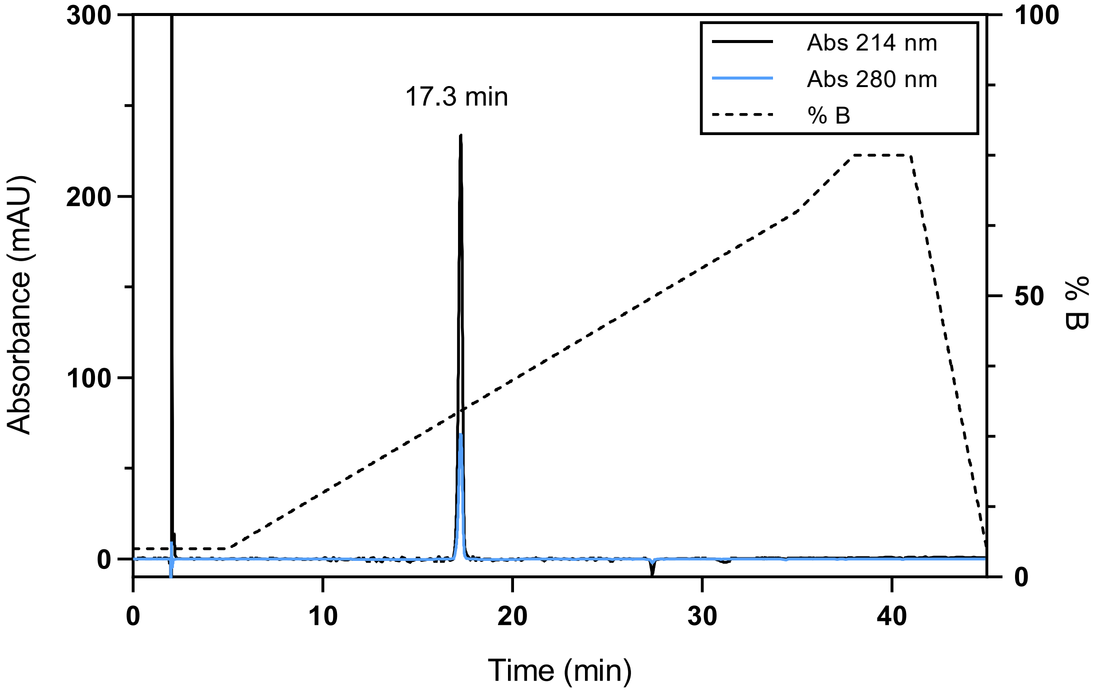

3: H_2N-Y(C_2H_4-Cy3$_s$)GPFLRRRRPK-COOH

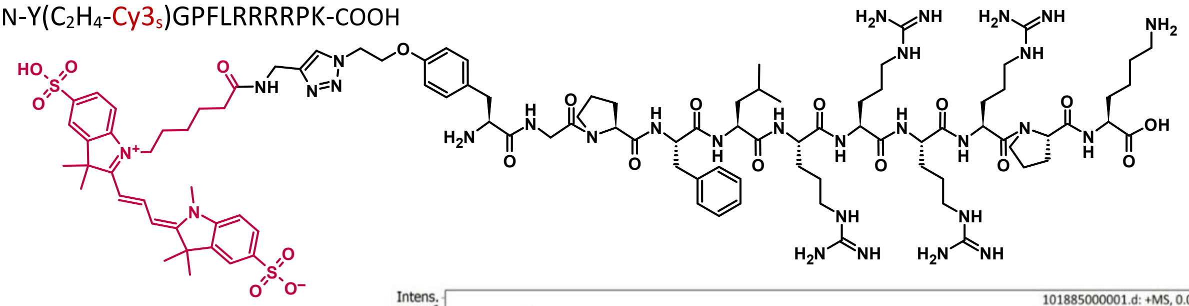

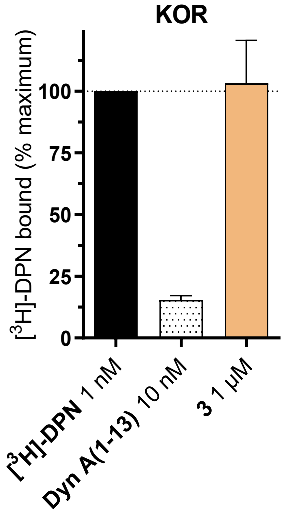

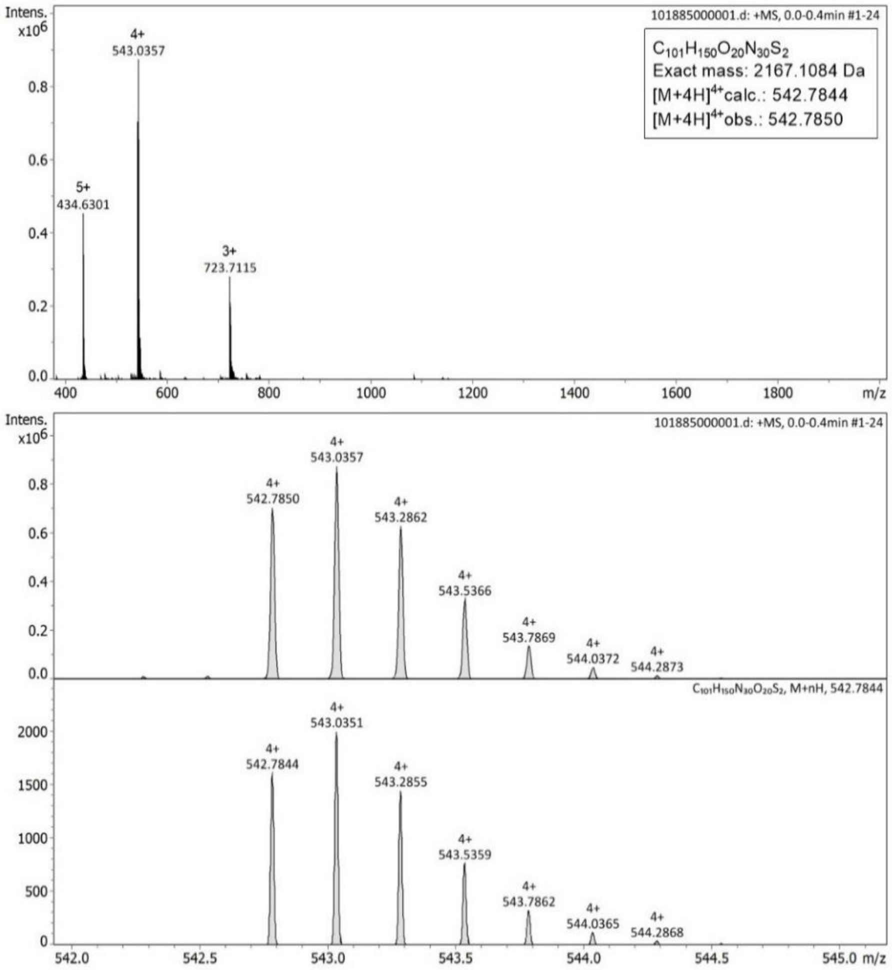

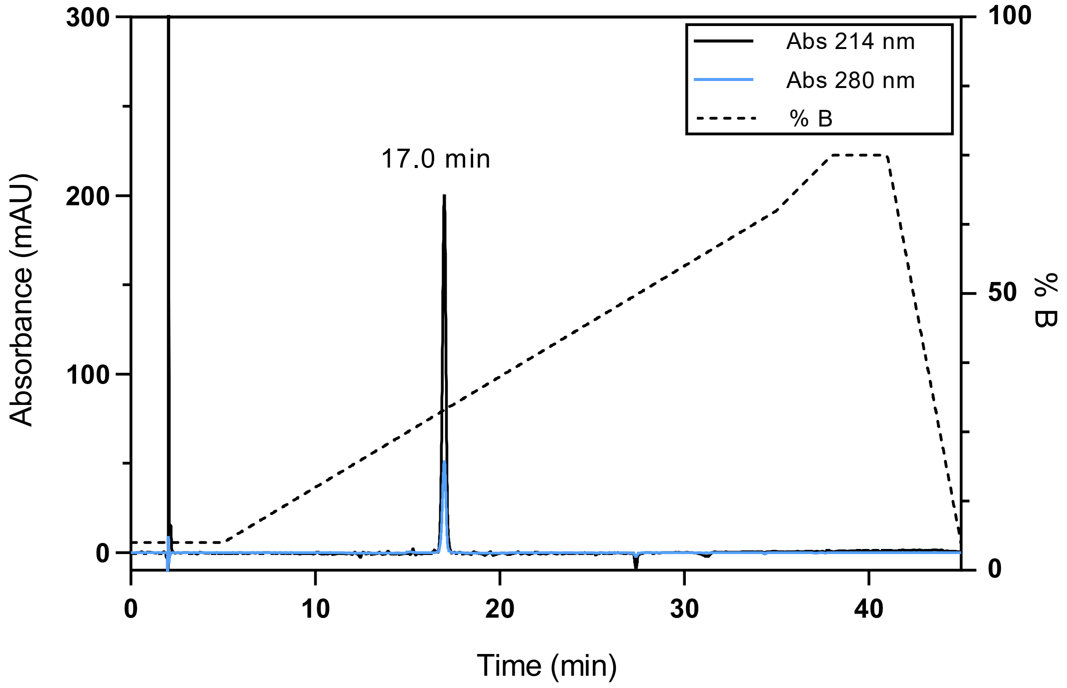

4: H_2N-Y(C_2H_4-Cy3$_s$)GPFLRRRRPK-$CONH_2$

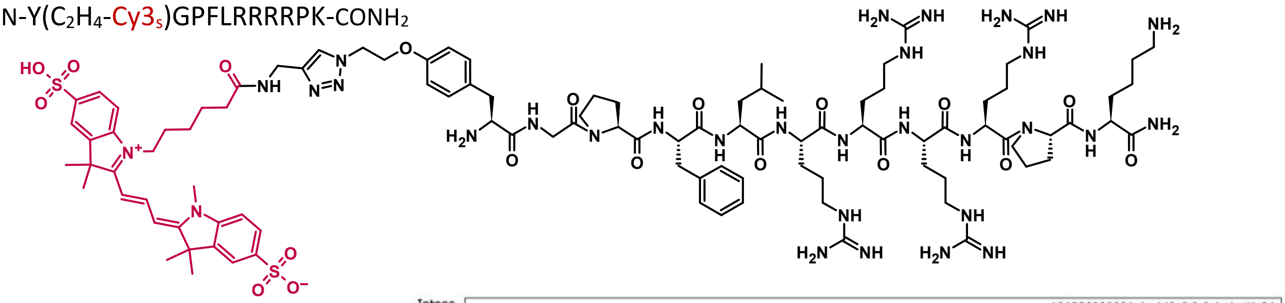

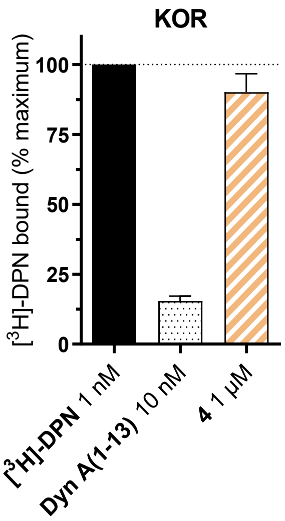

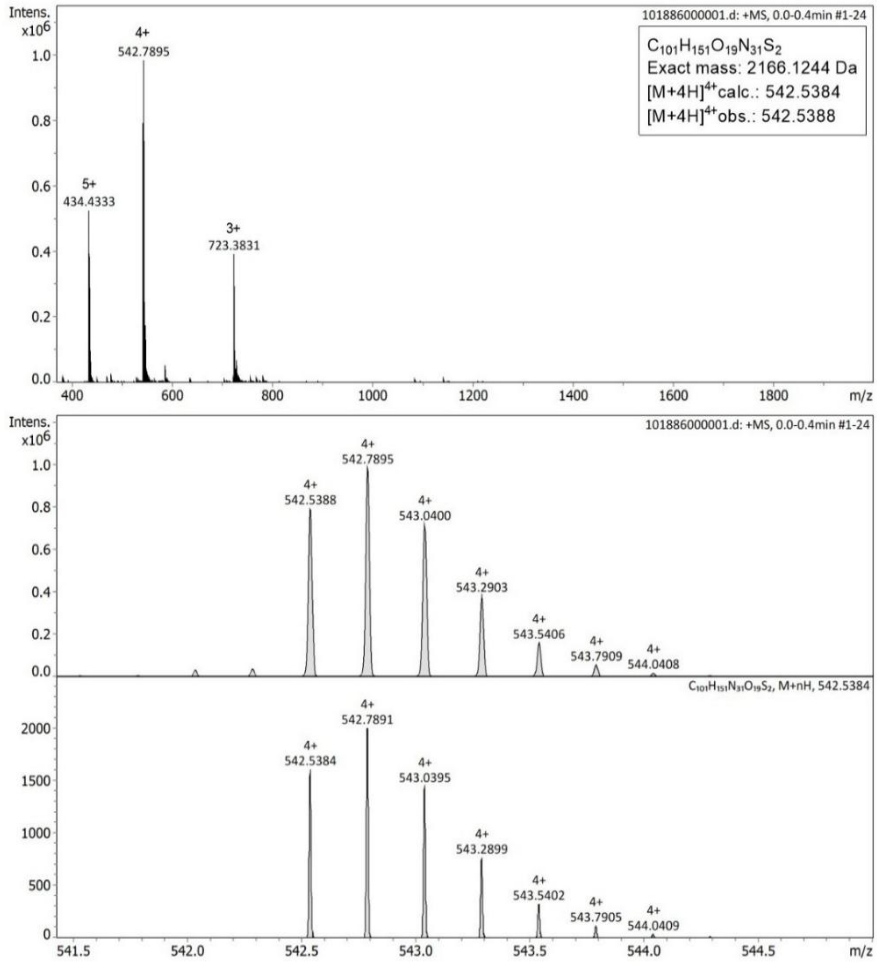

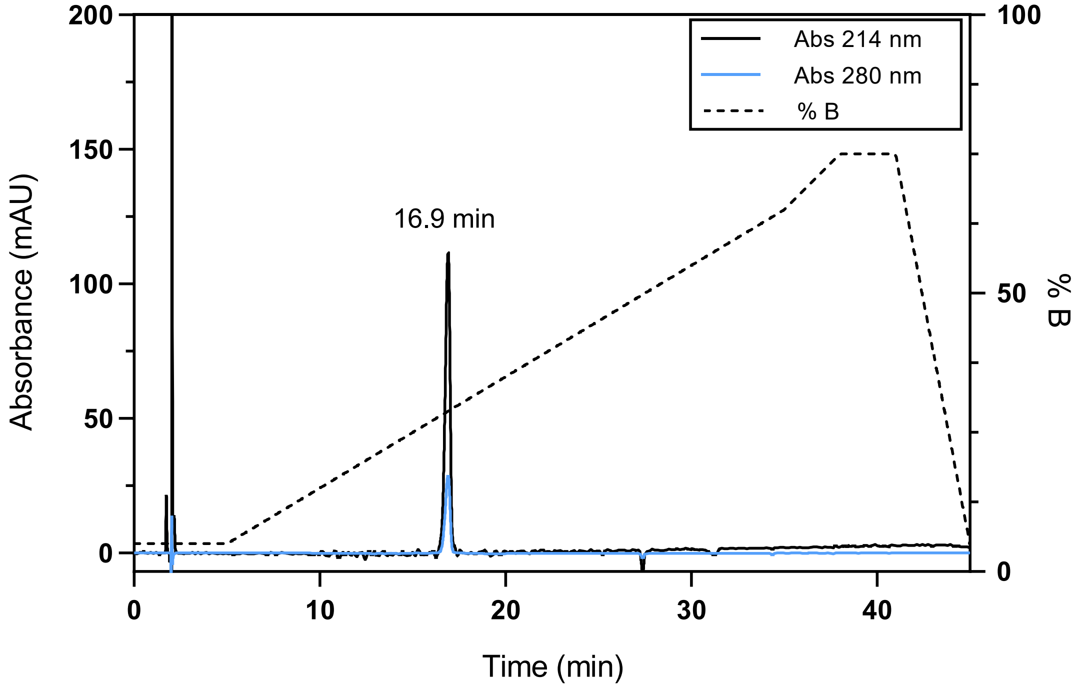

5: H_2N-YGPFLRRRRPKLK(AEEA-Cy3$_S$)-$CONH_2$

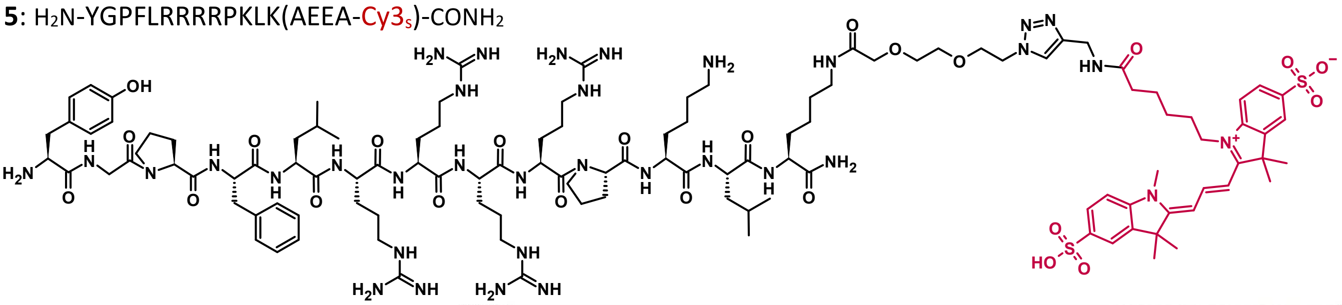

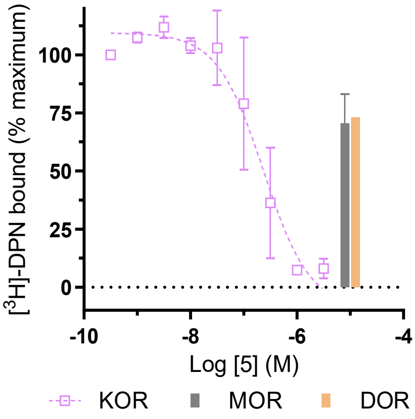

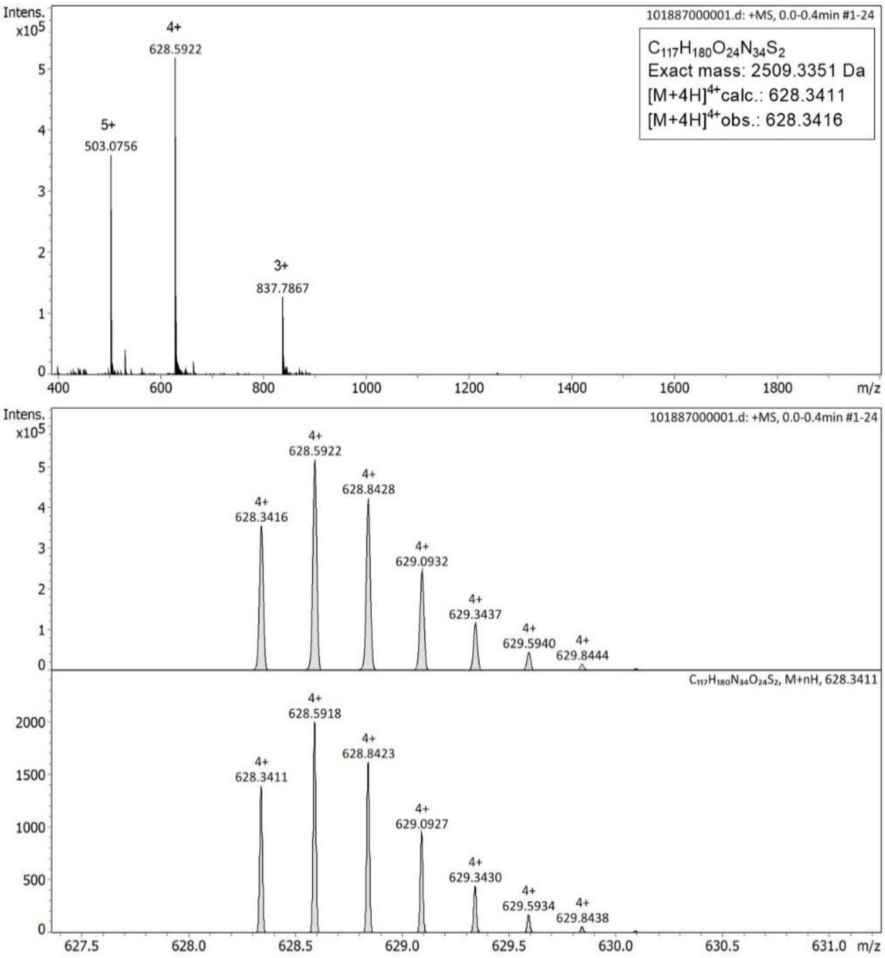

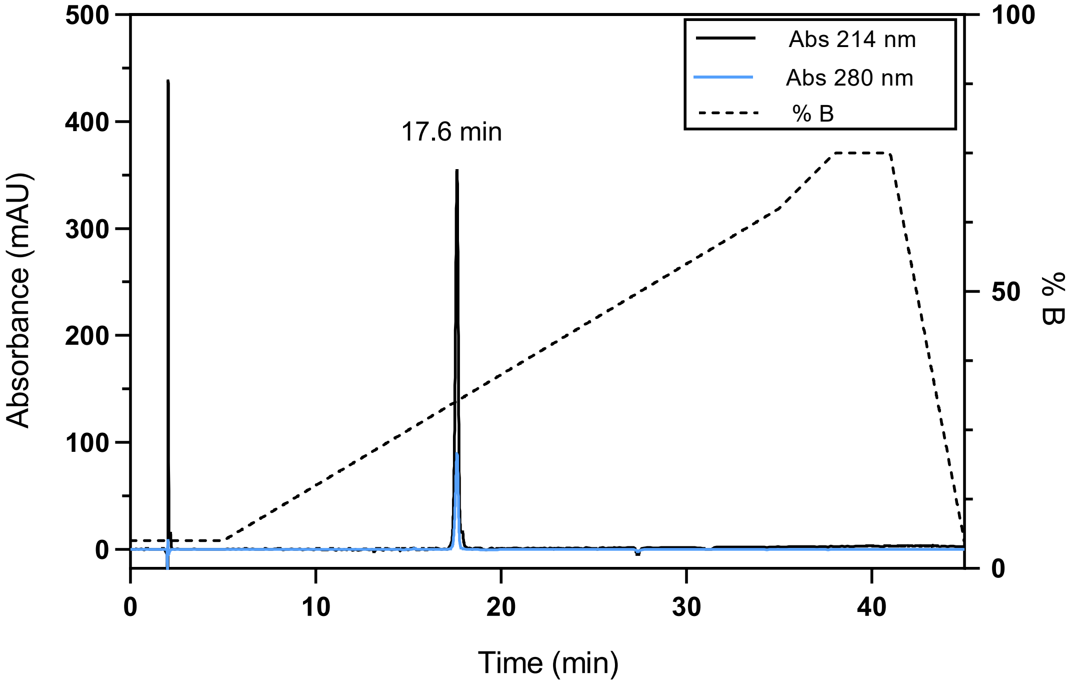

6: H2N-YGPFLRRRRPK(AEEA-Cy3s)LK-CONH2

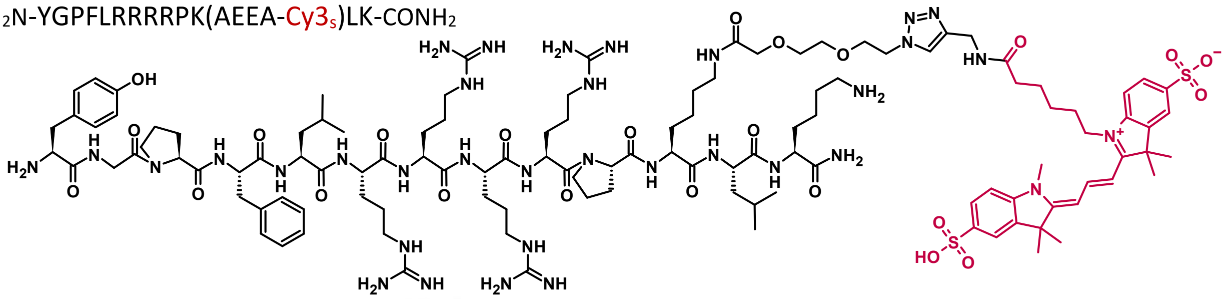

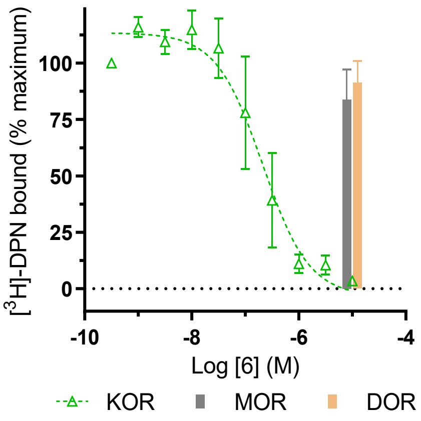

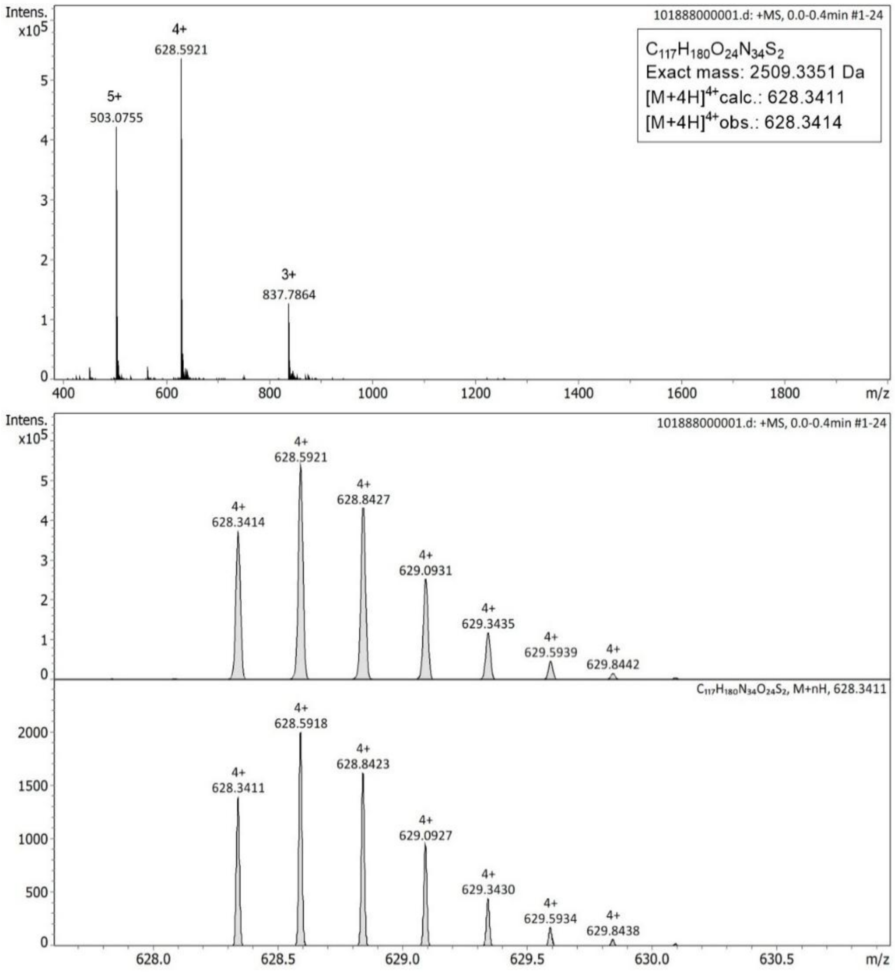

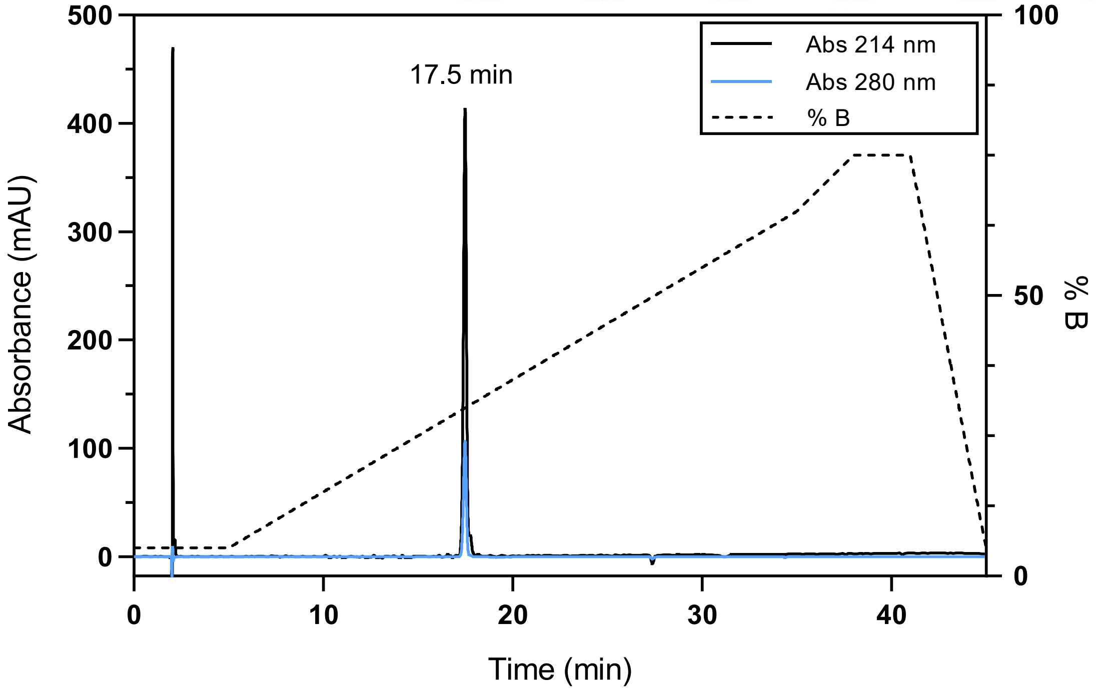

7: H_2N-YGPFLRRRRPK(AEEA-Cy3$_s$)-COOH

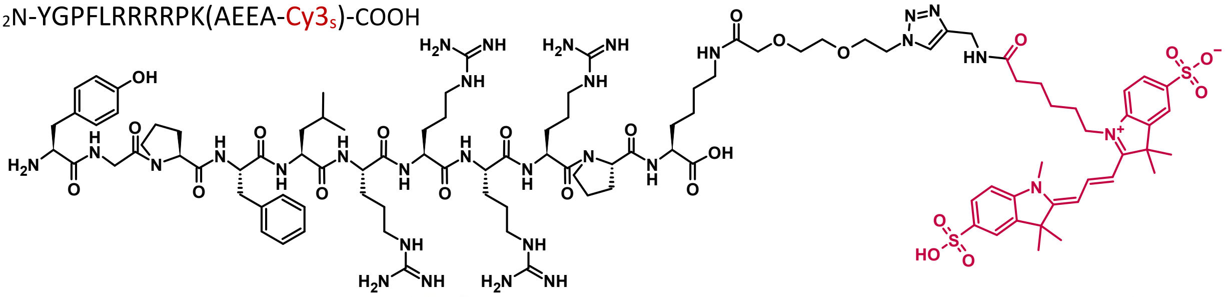

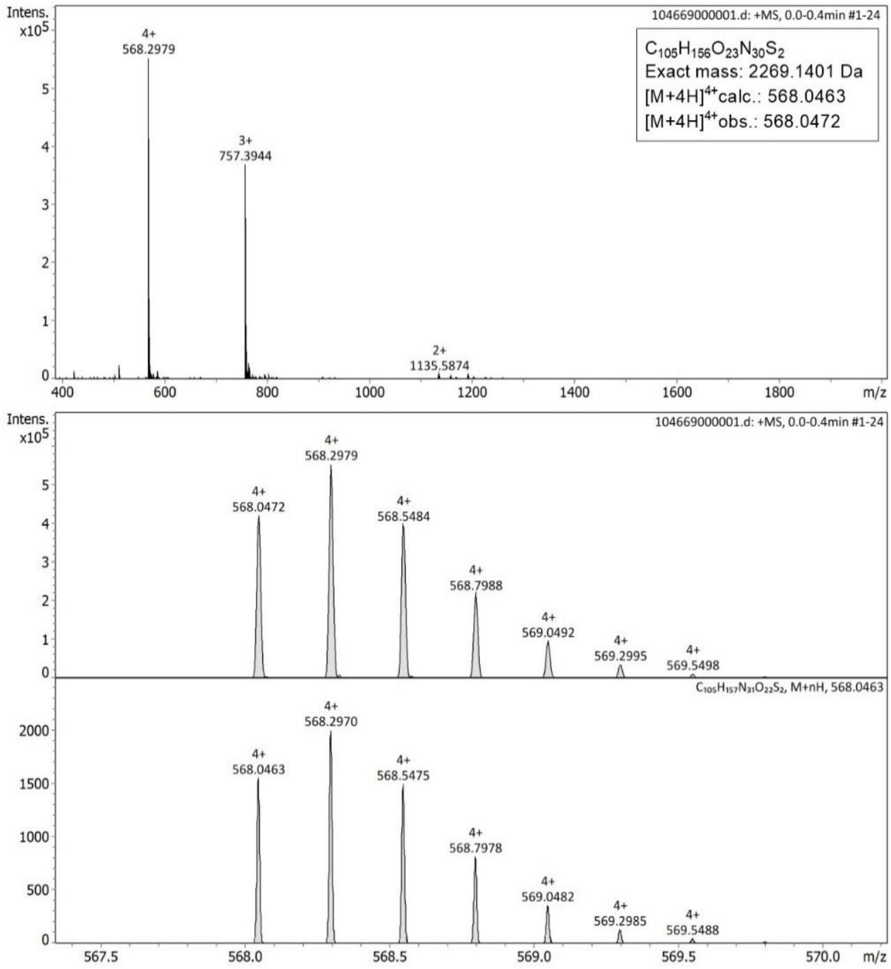

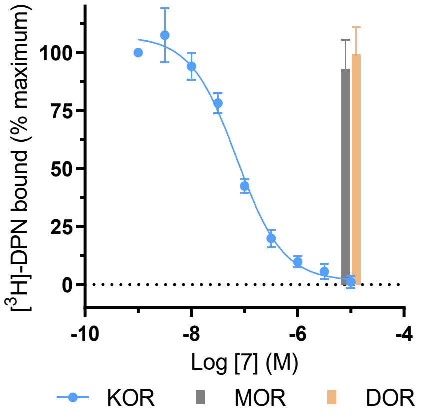

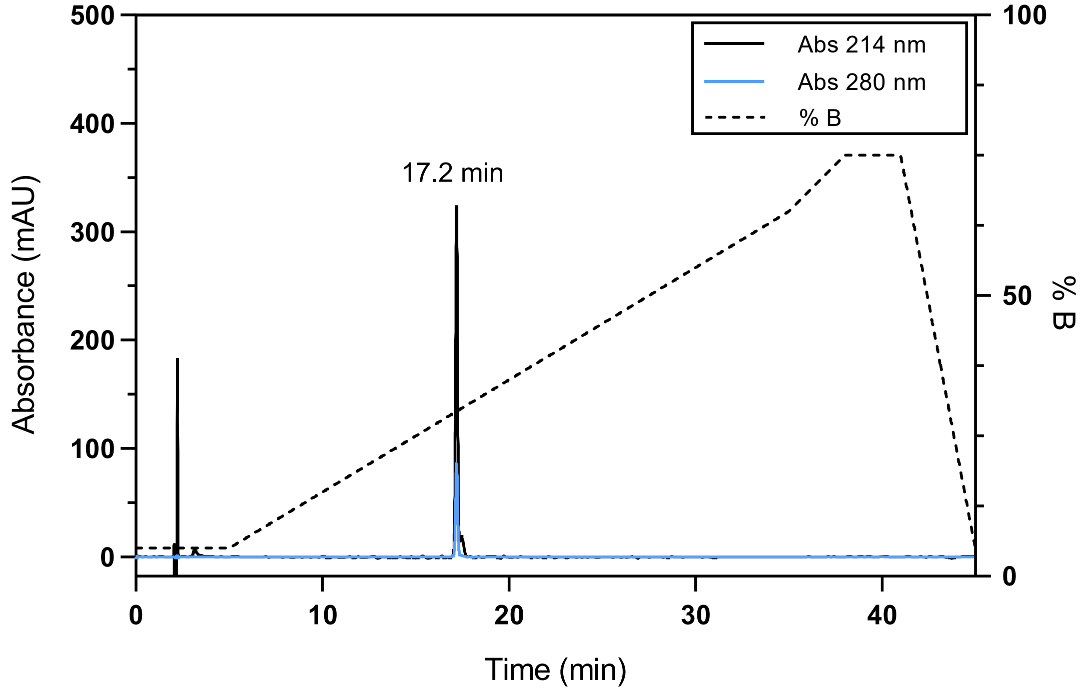

8: H_2N-YGPFLRRRRPK(AEEA-Cy3$_s$)-$CONH_2$

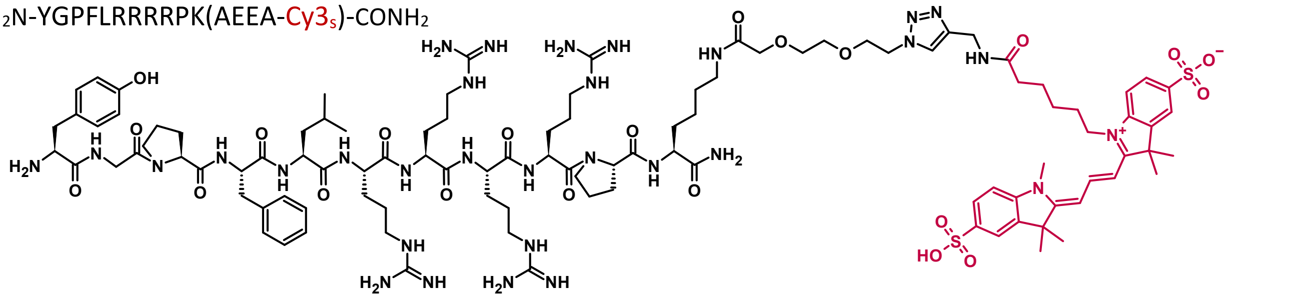

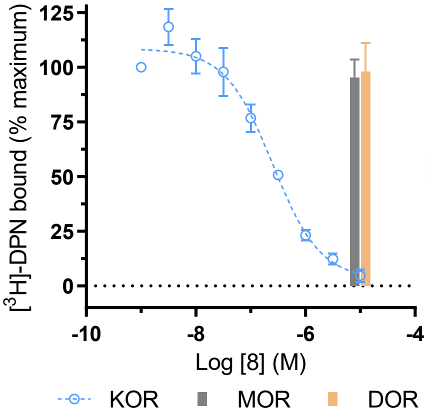

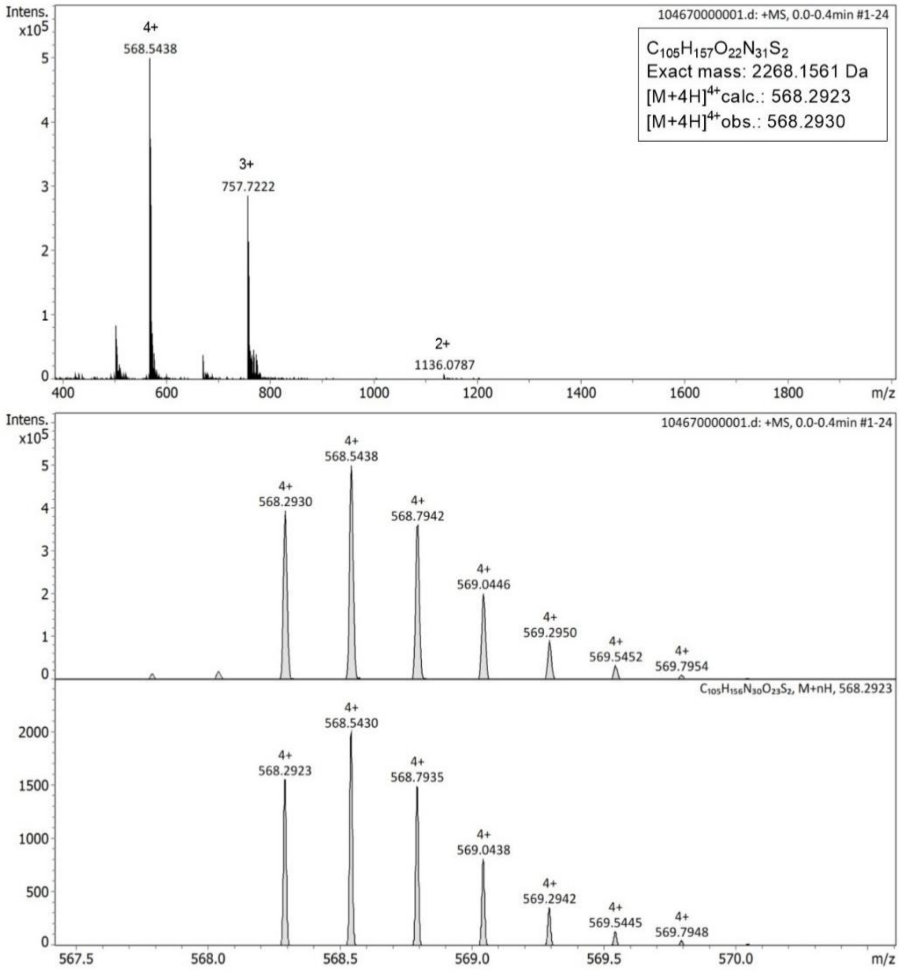

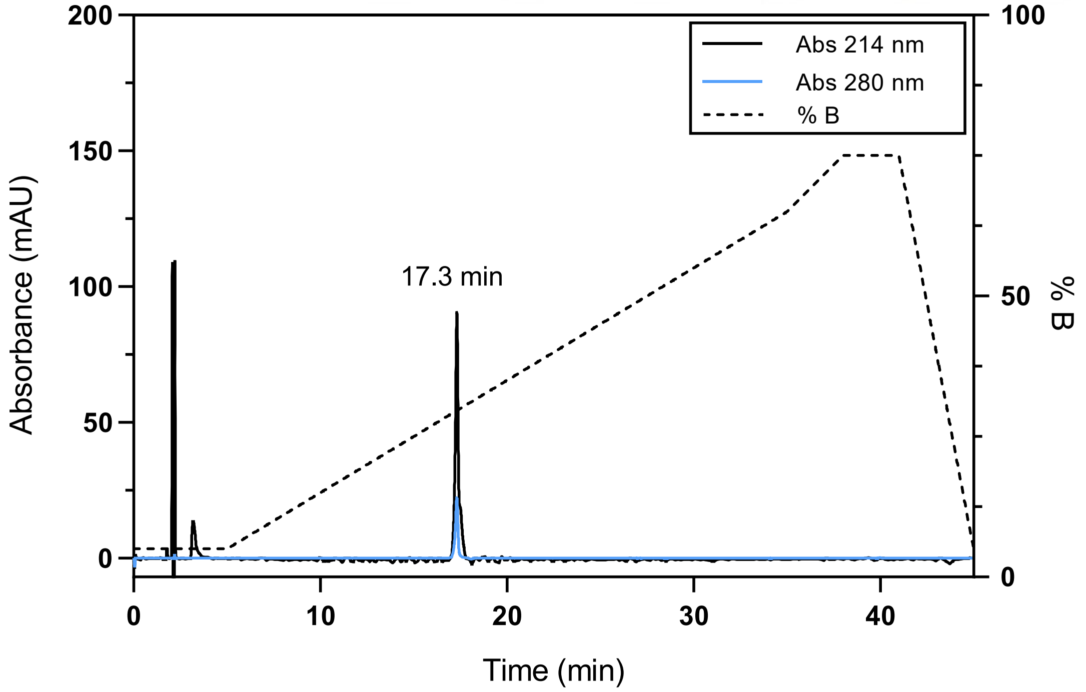

9: H$_2$N-YGPFLRRRRPKLK(AEEA-Cy3$_S$)-COOH

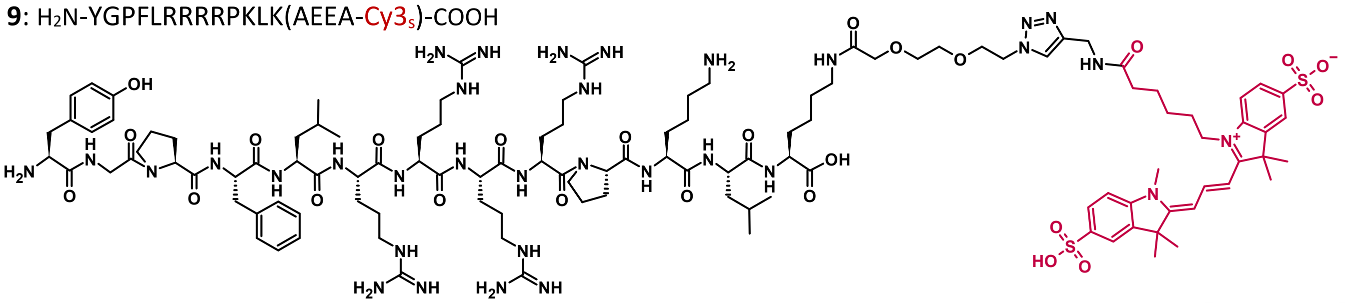

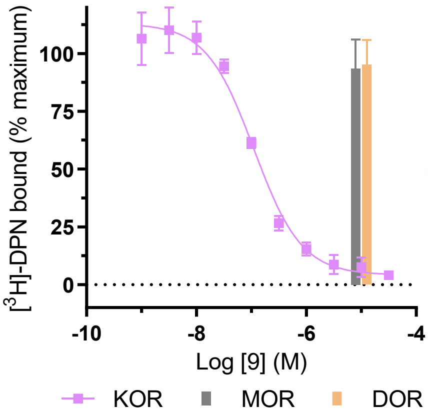

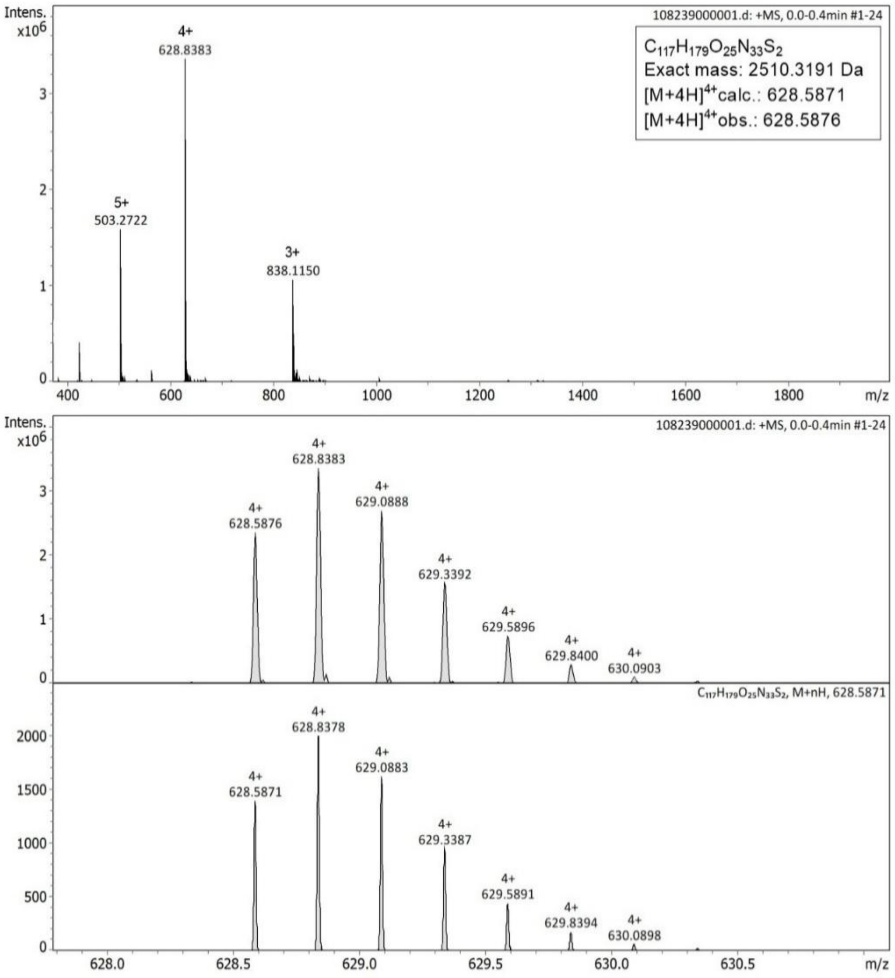

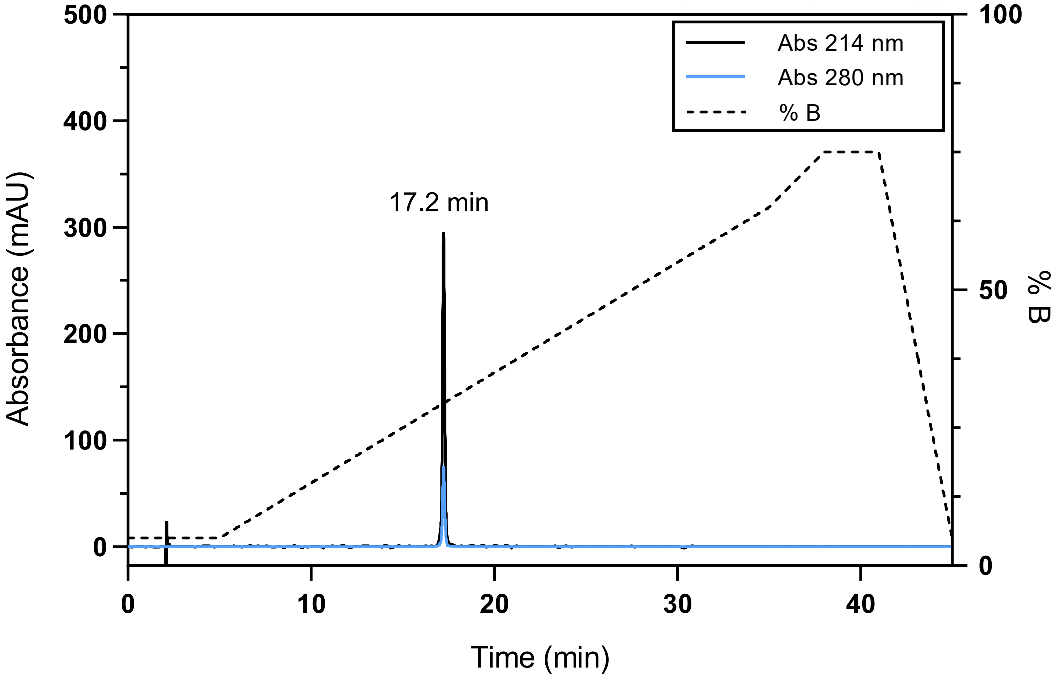

10: H_2N-YGPFLRRRRPK(AEEA-Cy3$_s$)LK-COOH

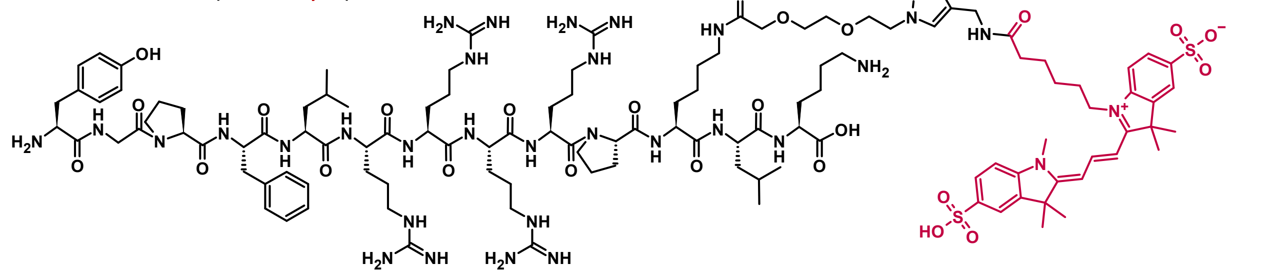

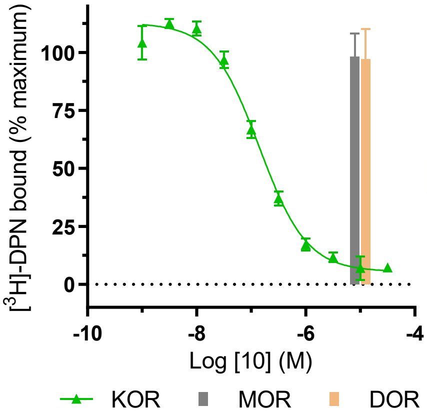

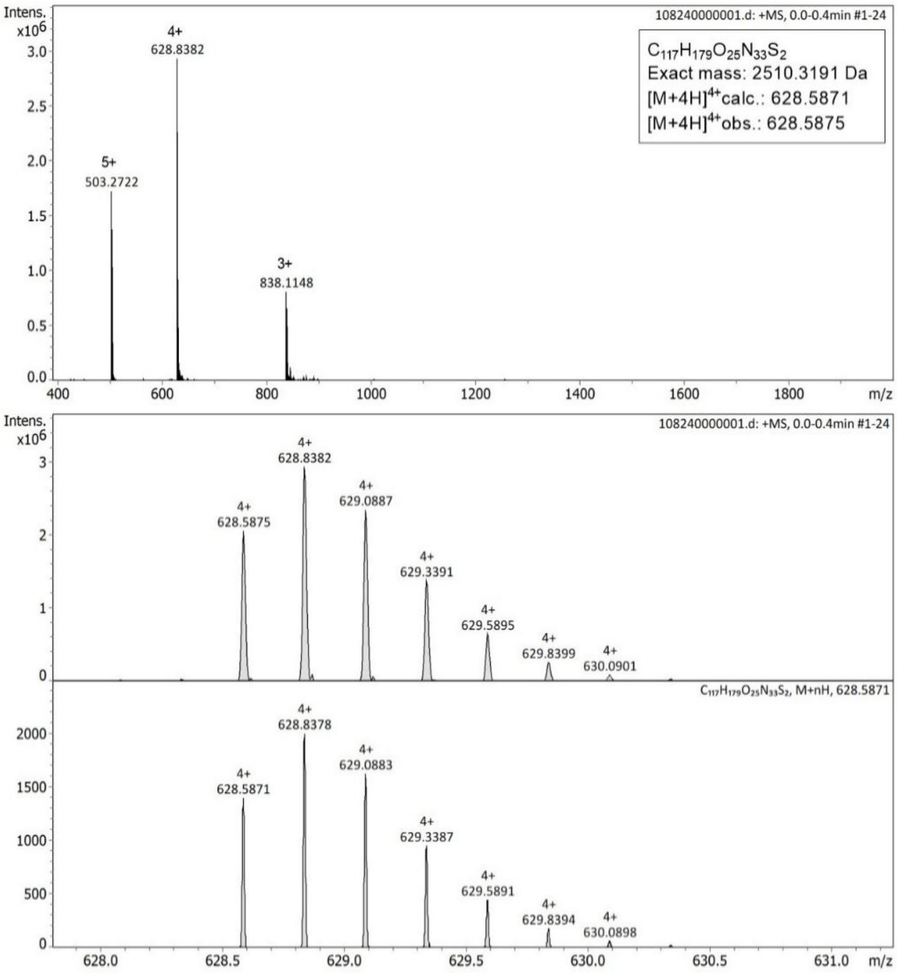

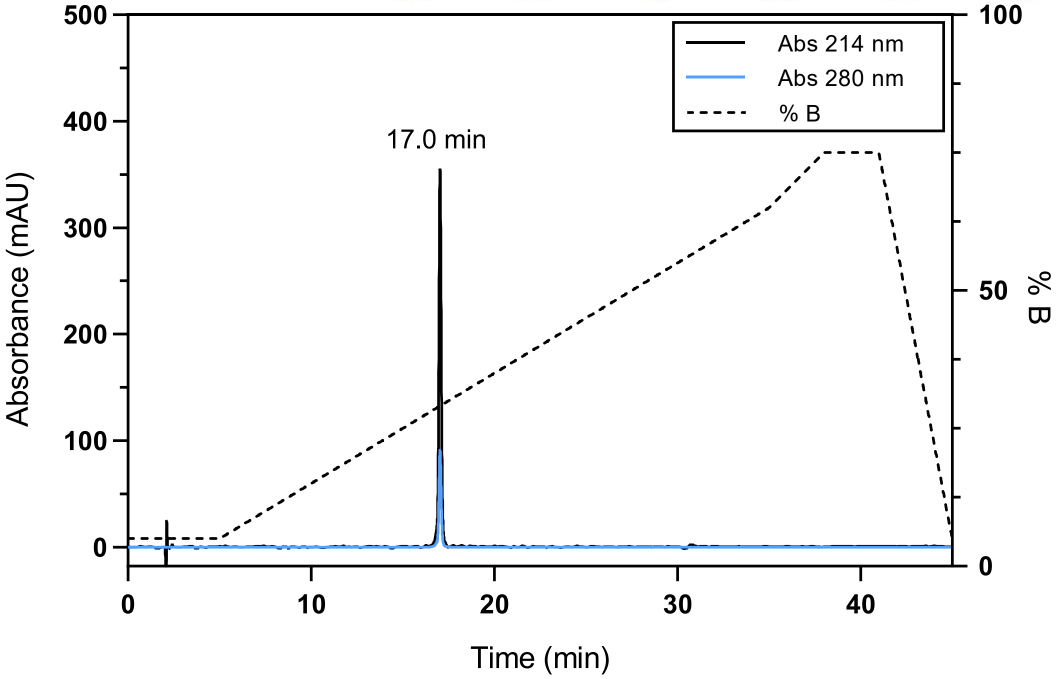

[P^3,R^8]Dyn A(1-11)-OH: H_2N-YGPFLRRRRPK-COOH

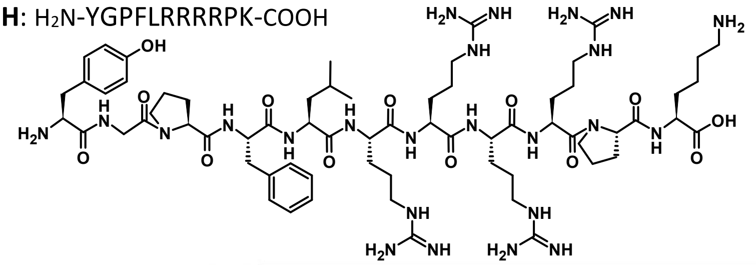

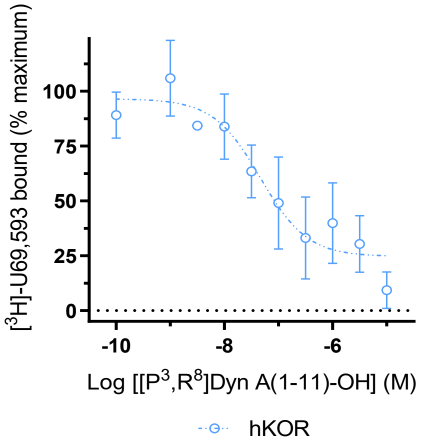

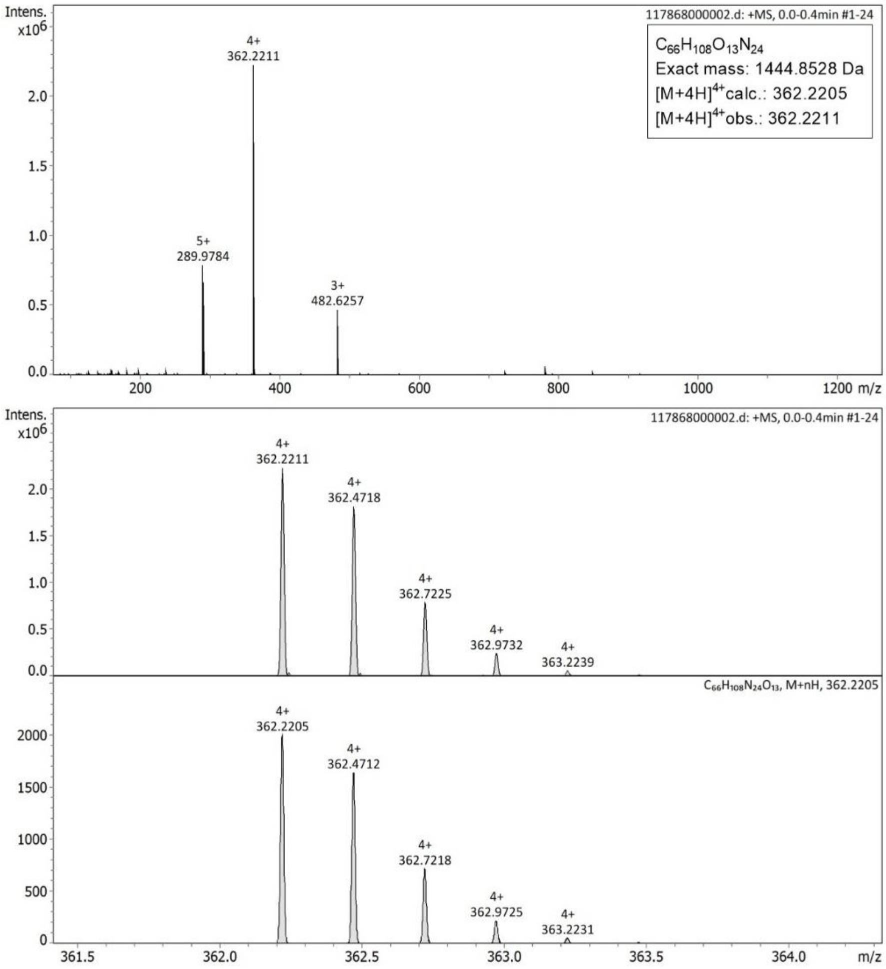

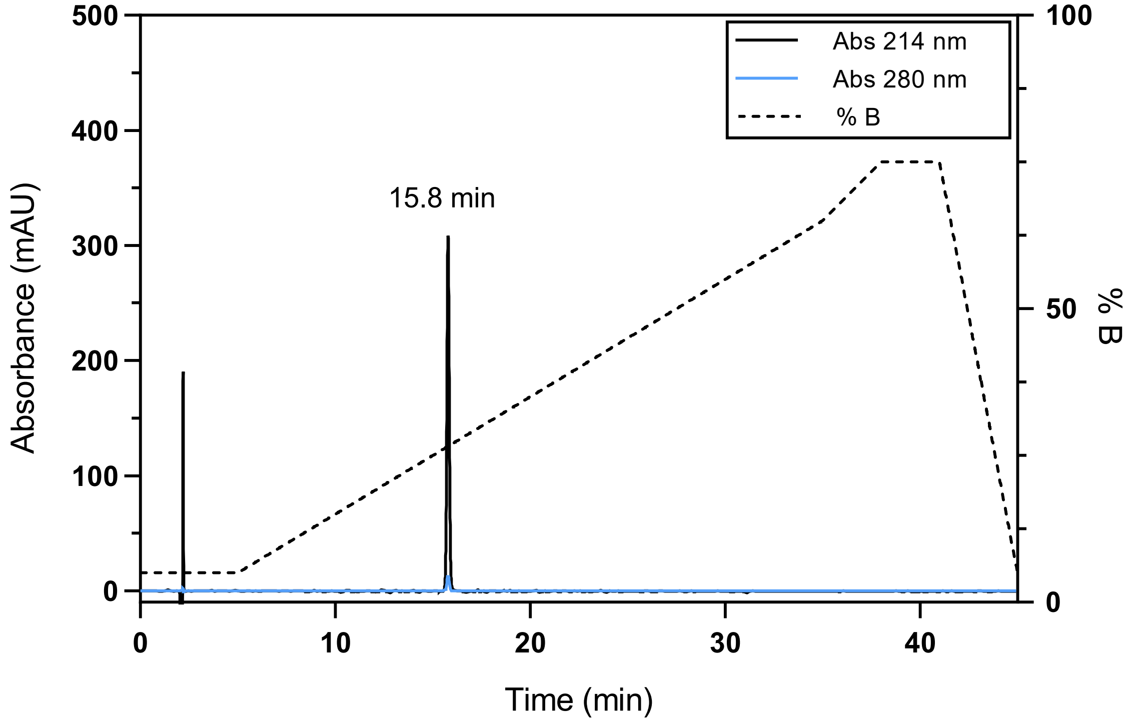

Figure S1. Overview of structures, purities, radioligand displacement assays, and HRMS. Retention times and product purities were determined by analytical C_{18}-RP-HPLC and UV detection at 214 nm. Solvent A (ddH_2O + 0.1% TFA) and B (ACN + 0.08% TFA) were used as eluents at a 1 mL/min flow rate and a linear gradient of 5-65% B in 30 min. All final products had a purity of ≥95%. Radioligand displacement assays were carried out with [^{3}H]-DPN using membrane preparations of HEK293 cell lines stably expressing murine KOR-GFP, MOR-GFP, or DOR-GFP, or [^{3}H]-U69,593 using membrane preparations of CHO cells stably expressing the human KOR. Specific binding was obtained by subtraction of non-specific binding at 10 µM naloxone, or 10 µM U69,593 from total binding, and values were normalized to the percentage of maximum binding (i.e., no competing ligand present). The K_i values were derived from one-site-fitted competition binding curves. In cases where compounds did not displace [^{3}H]-DPN to more than 25% at concentrations of 10 µM (shown as one-point binding), the K_i value was denoted as >10 µM. Experiments were carried out in duplicates, and data are presented as mean ± SD from at least three independent experiments. Final products were confirmed through HR-ESI-MS direct injections on a maXis HD ESI-Qq-TOF mass spectrometer.

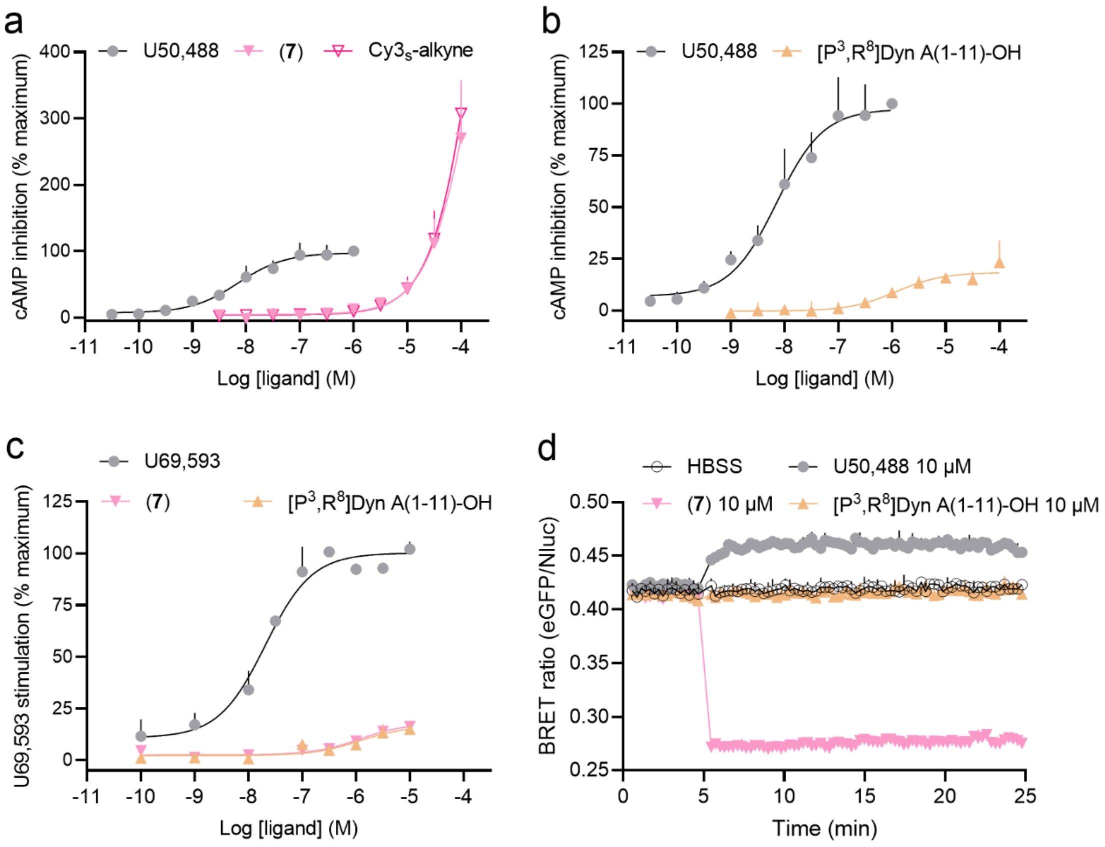

Figure S2. KOR-mediated downstream signaling and β-arrestin recruitment. (**a**) Measurements of cAMP inhibition by U50,488, [P^3,R^8,K^{11}(AEEA-Cy3$_s$)]Dyn A(1-11)-OH (**7**), and the fluorophore Cy3$_s$ alone. Note the interference of the fluorescent moiety with the readout of the cAMP assay from 1 μM onwards, creating exponential curves for tracer (**7**) and Cy3$_s$-alkyne. (**b**) cAMP measurements on the non-fluorescent counterpart of (**7**) show weak partial agonism around 1 μM (*see Table 2*). (**c**) Circumventing fluorescent readouts, a [^{35}S]-GTPγS binding assay on hKOR expressing CHO membrane fractions similarly revealed weak partial agonism by both lead tracer [P^3,R^8,K^{11}(AEEA-Cy3$_s$)]Dyn A(1-11)-OH (**7**) and its non-fluorescent parent peptide [P^3,R^8]Dyn A(1-11)-OH. U69,593, a selective KOR agonist, was used as a positive control. (**d**) Despite its partial agonism, [P^3,R^8]Dyn A(1-11)-OH did not show β-arrestin-2 recruitment in HEK293 cells transiently expressing mKOR, while selective KOR agonist U50,488 was used as a positive control. Note that tracer (**7**) again interfered with the assay readout due to its fluorescent moiety, resulting in a drop in BRET ratio upon compound addition at 5 min. Data represent mean ± SD of triplicates (n=3, cAMP assay) or duplicates (n=2, β-arrestin), carried out in at least three independent experiments (N≥3).

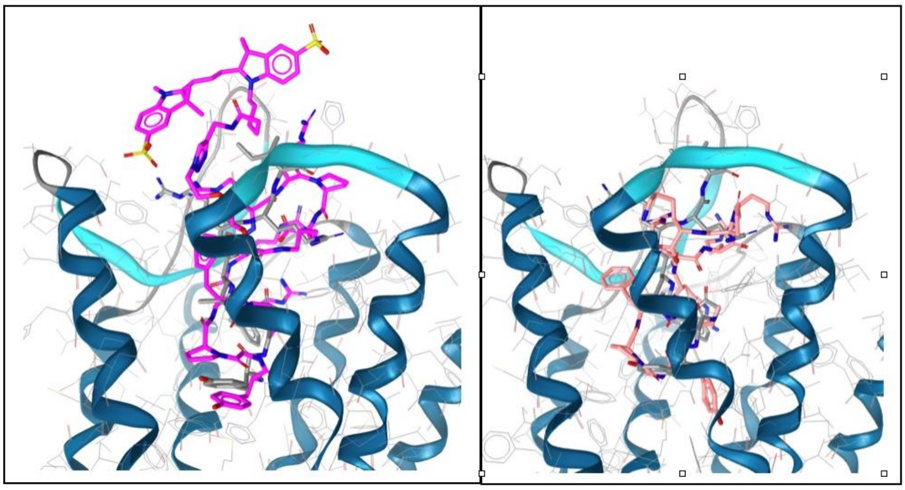

Figure S3. Comparison of Dyn A(1-8), tracer [P[3],R[8],K[11](AEEA-Cy3$_s$)]Dyn A(1-11)-OH (7), and parent peptide [P[3],R[8]]Dyn A(1-11)-OH in the KOR-Dyn A(1-8) agonist binding site. (**a**) Depictions of the prioritized docked poses of tracer (**7**) (*magenta*) and parent peptide (*peach*) in the binding site of the cryo-EM resolved KOR with Dyn A(1-8) (*gray*) (PDB ID 8F7W). Hydrogen atoms not displayed.

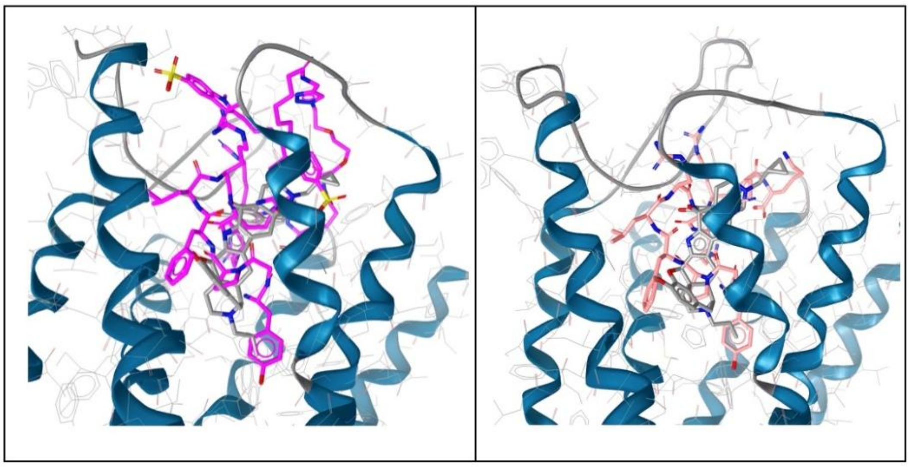

Figure S4. Comparison of NorBNI, tracer [P[3],R[8],K[11](AEEA-Cy3$_s$)]Dyn A(1-11)-OH (7), and parent peptide [P[3],R[8]]Dyn A(1-11)-OH in the KOR-NorBNI antagonist binding site. (**a**) Depictions of the prioritized docked poses of tracer (**7**) (*magenta*) and parent peptide (*peach*) in the binding site of the cryo-EM resolved KOR with NorBNI (*gray*) (PDB ID 8VVE). Hydrogen atoms not displayed.

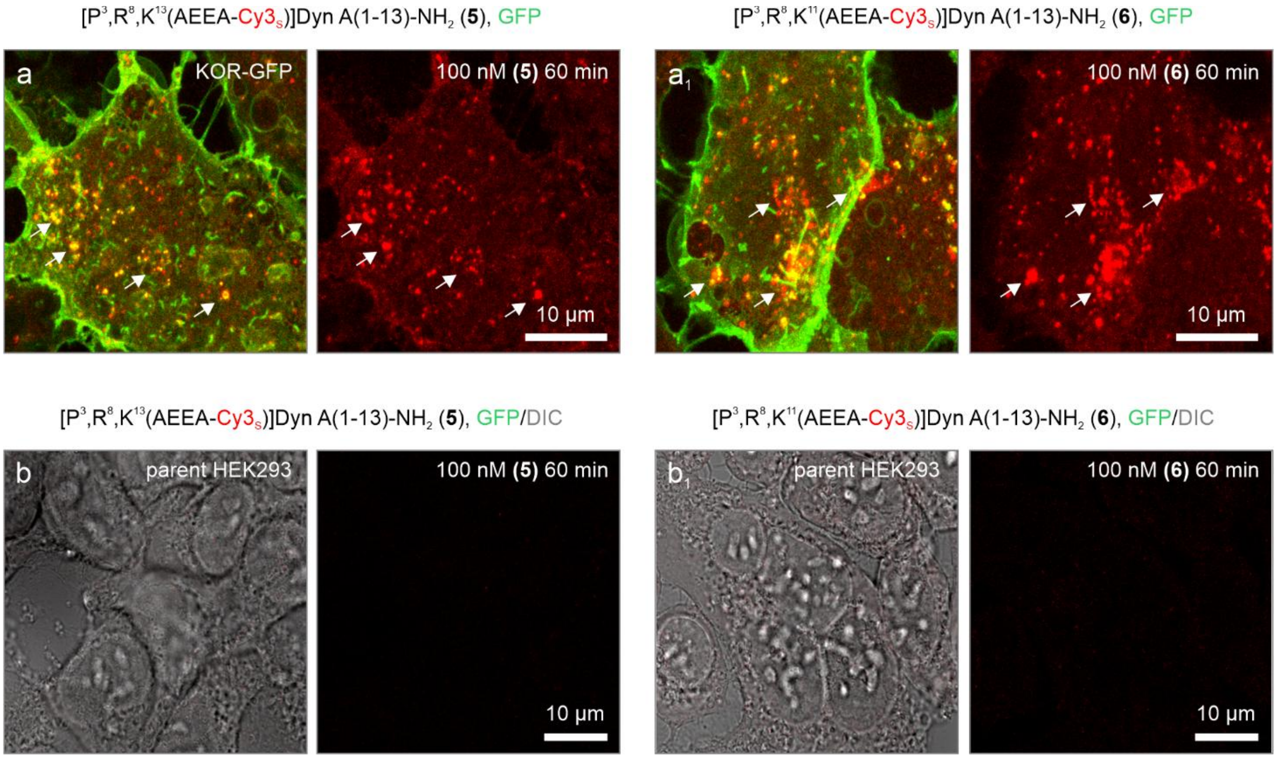

Figure S5. Validation of tracers [P^3,R^8,K^{13}(AEEA-$Cy3_s$)]Dyn A(1-13)-NH_2 (5) and [P^3,R^8,K^{11}(AEEA-$Cy3_s$)]Dyn A(1-13)-NH_2 (6) in mKOR-GFP overexpressing or parent HEK293 cells. (**a-b_1**) Confocal microscopy, after a brief wash with PBS for 5 min, a subsequent 30 min PFA fixation step and two additional 5 min PBS washing steps, revealed maintained $Cy3_s$ signals (*red*, excitation 560 nm) co-localized with GFP in mKOR-GFP HEK293 cells (*green*, excitation 480 nm) (a,a_1), but not in parent HEK293 cells (b,b_1), supporting KOR-dependent cellular labeling with both tracers (**5**) and (**6**) at 100 nM. *Arrows* are marking sites of high accumulation and overlap of tracer and KOR-GFP signals (a,a_1; creating a *yellow* signal in the overlaid images on the left side). Whole cells were visualized with differential interference contrast (DIC; left figure panels, b,b_1) to reveal cellular structures in the absence of any fluorescent signal.

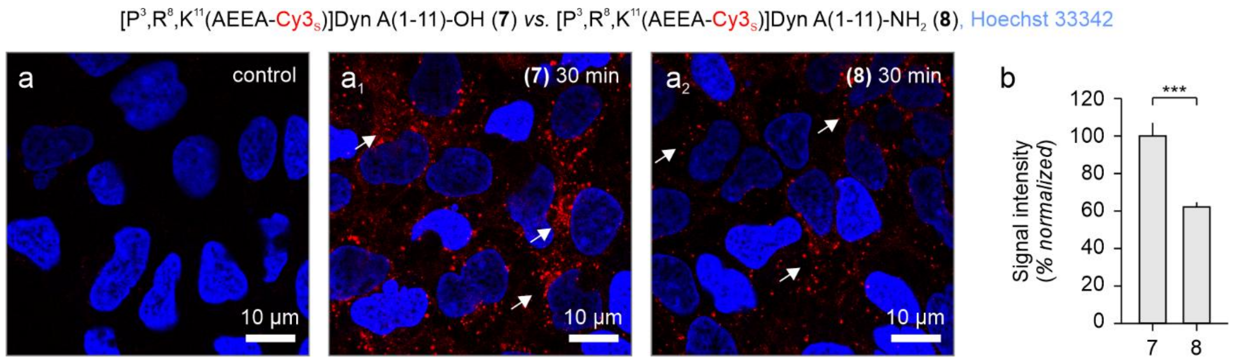

Figure S6. Quantitative analysis of signal-to-noise ratios between lead tracer [P^3,R^8,K^{11}(AEEA-Cy3$_s$)]Dyn A(1-11)-OH (7) and its amide counterpart [P^3,R^8,K^{11}(AEEA-Cy3$_s$)]Dyn A(1-11)-NH$_2$ (8). (**a-b**) Fluorescent intensity measurements between tracers (**7**) and (**8**). *Arrows* mark sites of high densities of tracer accumulation (n=10 cells per condition). Data are presented as means ± SEM. ***$p < 0.001$

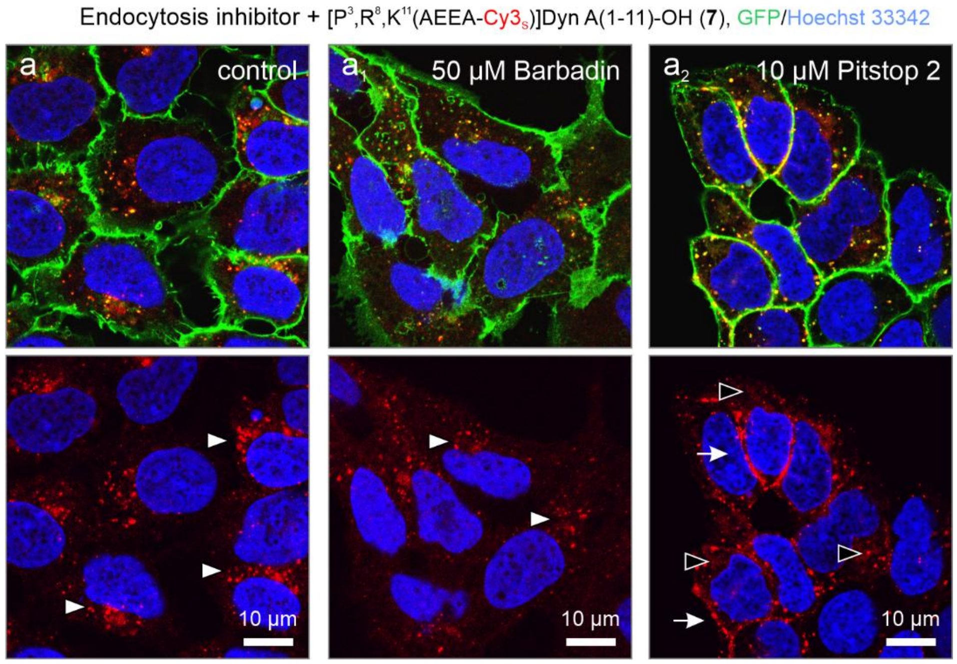

Figure S7. Inhibition of endocytosis limits compound internalization. (**a-a$_2$**) Pretreatment with the selective β-arrestin/β2-adaptin inhibitor barbadin revealed diminished [P^3,R^8,K^{11}(AEEA-Cy3$_s$)]Dyn A(1-11)-OH (**7**) uptake (*arrowheads*, a$_1$), while the clathrin inhibitor pitstop 2 both diminished uptake (*open arrowheads*) and arrested compound (**7**) at the membranes (*arrows*).

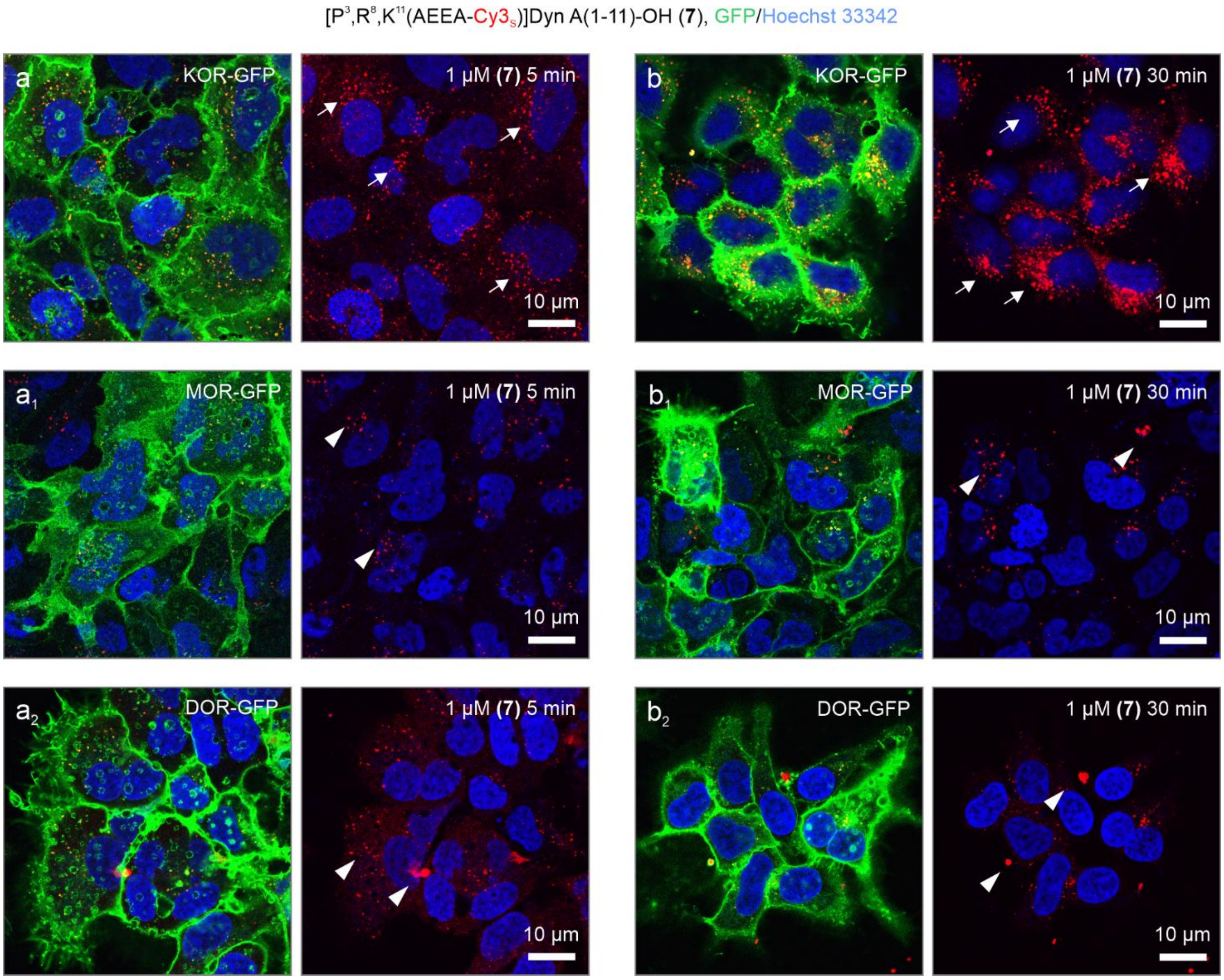

Figure S8. Selectivity study of [P^3,R^8,K^{11}(AEEA-Cy3$_s$)]Dyn A(1-11)-OH (7) at 1 µM on mKOR-GFP, mMOR-GFP, or mDOR-GFP overexpressing cells. (**a-b$_2$**) Treatment with 1 µM of lead tracer (**7**) revealed labeling as early as 5 min with robust signals after 30 min of stimulation (*arrows*, *red*, excitation 560 nm). Note the co-localization of (**7**) and mKOR-GFP (*green*, excitation 480 nm, resulting in *yellow*). Minor compound accumulation, mostly non-overlapping with green signals, was observed in mDOR-GFP and mMOR-GFP-containing cells (*arrowheads*), which we attributed to binding to cellular debris *via* a non-opiate receptor-dependent mechanism. Hoechst 33342 was used as a nuclear counterstain (*blue*, excitation 405 nm).

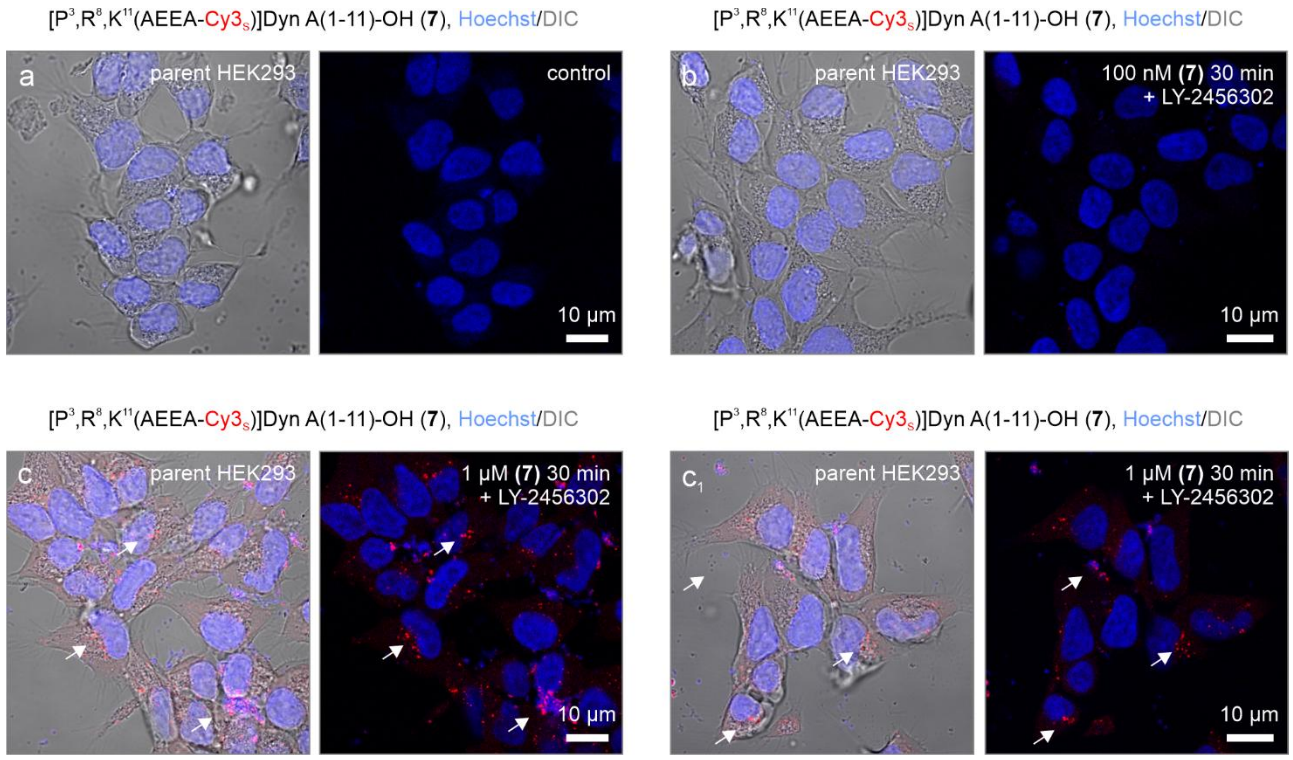

Figure S9. Background labeling of [P^3,R^8,K^{11}(AEEA-Cy3$_s$)]Dyn A(1-11)-OH (7) on parent HEK293 cells. (**a,b**) Exposure of up to 100 nM (**7**) for 30 min did not result in any labeling in parent HEK293 cells (*see also Fig. 4b,b$_1$*). (**c,c$_1$**) However, at concentrations ≥1 µM, there was minor tracer labeling, as well as strong binding to dead cells and debris (*arrows*, *red*, excitation 560 nm). The KOR antagonist LY-2456302, also blocking MOR and DOR at these concentrations (10 µM), did not prevent this binding, suggesting it is non-specific to opioid receptors. Hoechst 33342 was used as a nuclear counterstain (*blue*, excitation 405 nm).

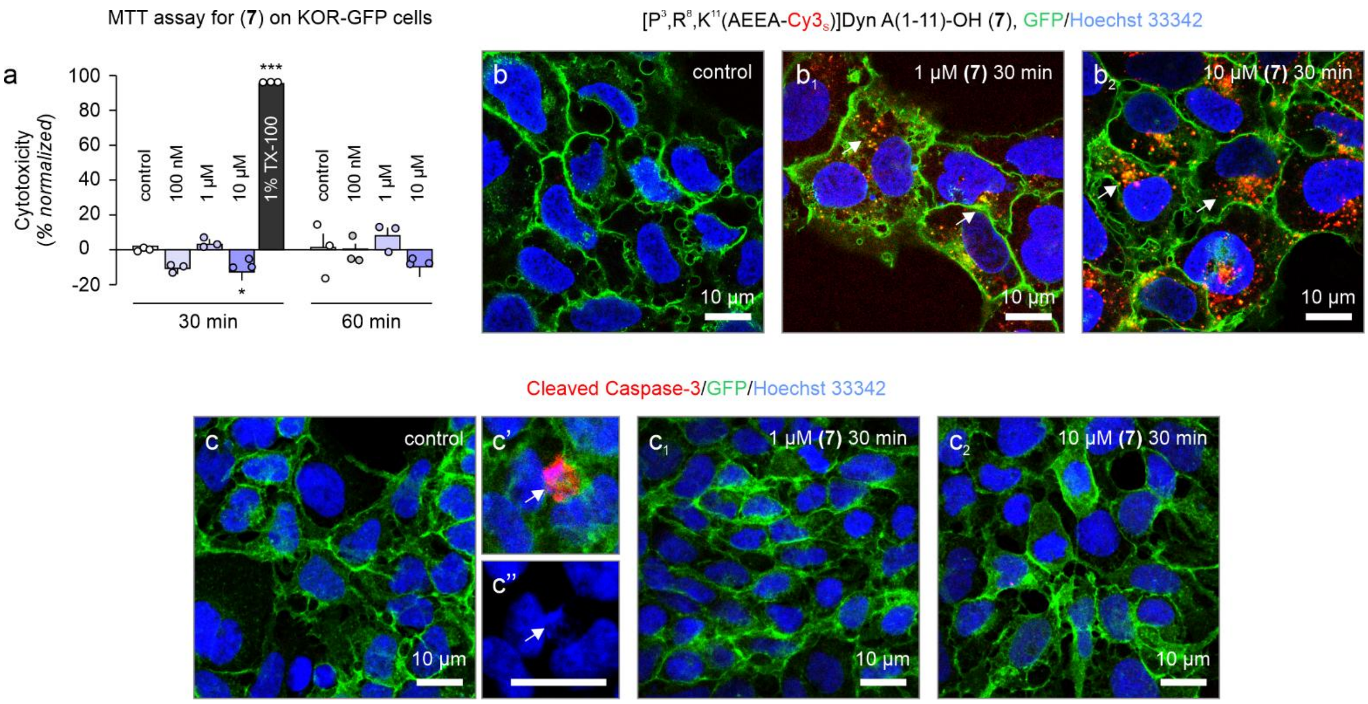

Figure S10. Cytotoxicity measurements in HEK293 cells overexpressing KOR-GFP. (**a**) MTT assays reveal no cytotoxicity in cells exposed to up to 10 µM [P^3,R^8,K^{11}(AEEA-$Cy3_s$)]Dyn A(1-11)-OH (**7**) for 30 or 60 min, as compared to positive control 1% Triton X-100 (n=3 cells per condition). Note the small but significant negative cytotoxicity with 10 µM tracer (**7**), indicating possible interaction between the $Cy3_S$ fluorophore and the MTT readout. (**b-c$_2$**) Treatment with tracer (**7**) up to 10 µM reveals no nuclear fragmentation (b-b_2; Hoechst$^+$) or accumulation of the apoptotic marker cleaved caspase-3 (c-c_2; *arrow*). Data is presented as means ± SEM. *p < 0.05; ***p < 0.001.

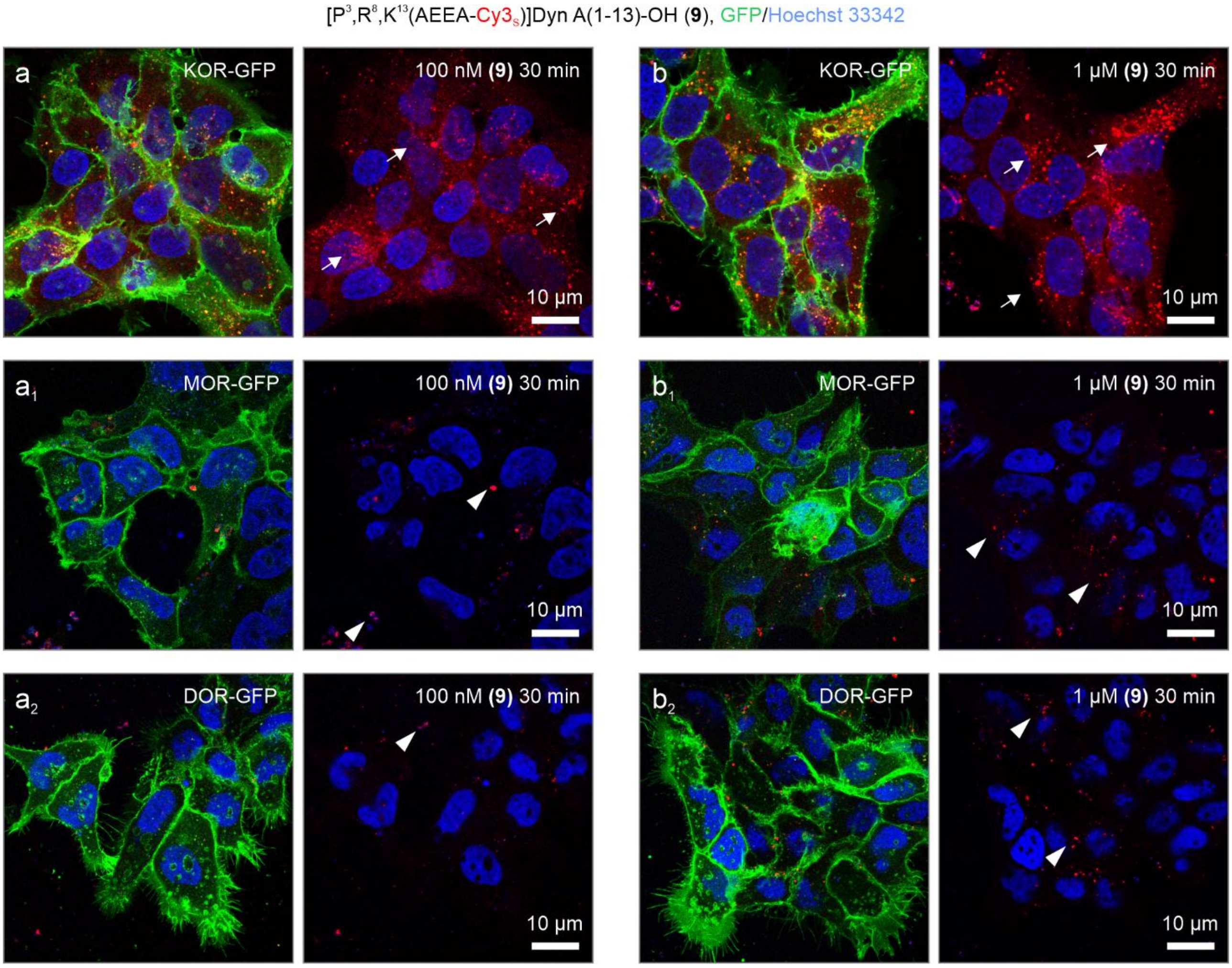

Figure S11. Selectivity study of [P^3,R^8,K^{13}(AEEA-$Cy3_s$)]Dyn A(1-13)-OH (9) on mKOR-GFP, mMOR-GFP, and mDOR-GFP overexpressing cells. (**a,b**) Stimulation with 100 nM or 1 µM of (**9**) revealed cellular labeling at 30 min (*arrows*, *red*, excitation 560 nm), but not as strong as its amide counterpart (**5**). Note the co-localization of (**9**) and KOR-GFP (*green*, excitation 480 nm, resulting in *yellow*). (**a_1-b_2**) Minor compound accumulation, mostly non-overlapping with green signals, was present in mDOR-GFP and mMOR-GFP (*arrowheads*), which we attribute to binding to cellular debris in a non-opiate receptor-dependent mechanism (*see Fig. S8*).

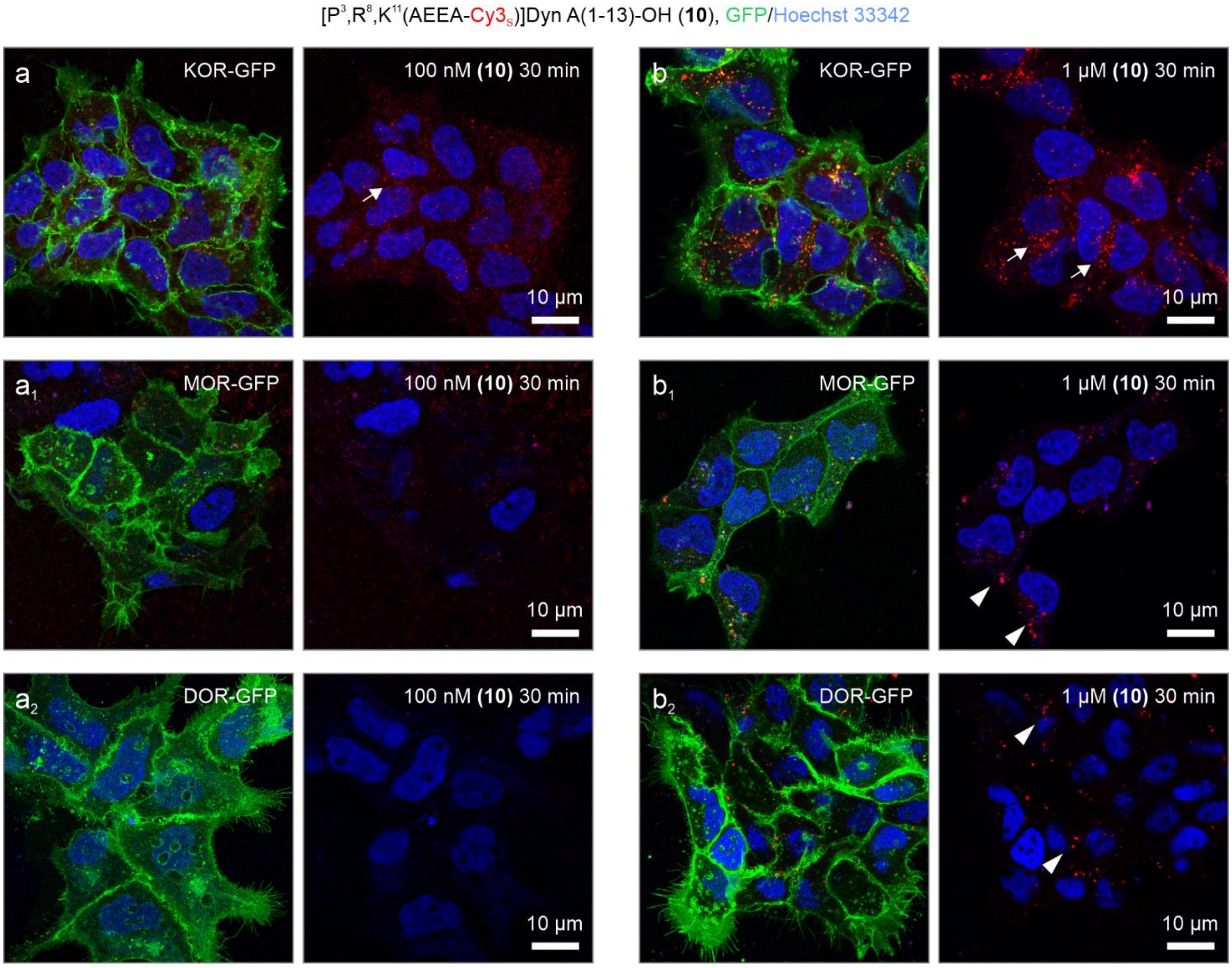

Figure S12. Selectivity of [P^3,R^8,K^{11}(AEEA-$Cy3_s$)]Dyn A(1-13)-OH (10) on mKOR-GFP, mMOR-GFP, and mDOR-GFP overexpressing cells. (**a,b**) Treatment with both 100 nM and 1 µM of tracer (**10**) revealed weak labeling at 30 min of stimulation (*arrows*, *red*, excitation 560 nm), as compared to its parent tracer (**6**). Note the co-localization of tracer (**10**) and KOR-GFP (*green*, excitation 480 nm, resulting in *yellow*). (**a_1,b_2**) Minor compound accumulation, mostly non-overlapping with green signals, was present in DOR-GFP and MOR-GFP (*arrowheads*), which we attributed to binding to cellular debris in a non-opiate receptor-dependent mechanism (*see Fig. S8*).

Primary mouse cortical neurons (**7**), Hoechst/DIC

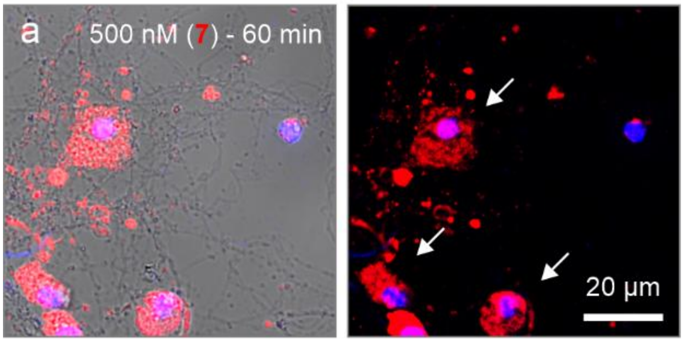

Figure S13. Debris labeling in primary cortical neuronal cultures. (**a**) Similar to HEK293 cultures (*Fig. S9*), stimulation with 500 nM [P[3],R[8],K[11](AEEA-Cy3s)]Dyn A(1-11)-OH (**7**) labeled cellular debris (*red*, excitation 560 nm), including fragmented nuclei (*blue spheres*, excitation 405 nm) and damaged somas (*arrows*). Cells were visualized using DIC (left figure panel) to reveal cellular structures.

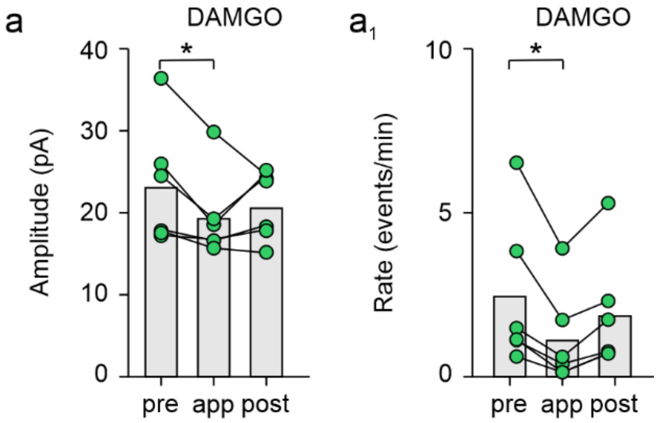

Figure S14. Measurements of spontaneous excitatory postsynaptic currents (sEPSCs) in superficial laminae neurons of the dorsal horn. (**a,a$_1$**) sEPSCs were recorded in neurons from laminae I and II of the spinal cord dorsal horn. Bath application of the selective MOR agonist DAMGO (10 µM), a positive control for our experimental setup, decreased both the amplitude and event rate of sEPSCs (n=6 cells per condition). *P<0.05.
